# Mitochondrial methylation is linked to sexually dimorphic growth in Nile tilapia (*Oreochromis niloticus*)

**DOI:** 10.3389/fcell.2025.1643817

**Published:** 2025-08-05

**Authors:** Partha Sarathi Tripathy, Prabhugouda Siriyappagouder, Artem Valeryevich Nedoluzhko, Ioannis Konstantinidis, Soumya Shephalika Dash, Bijay Kumar Behera, Janmejay Parhi, Kaja Skjærven, Francesc Piferrer, Jorge Manuel de Oliveira Fernandes

**Affiliations:** ^1^ Faculty of Biosciences and Aquaculture, Nord University, Bodø, Norway; ^2^ College of Fisheries, Rani Lakshmi Bai Central Agricultural University, Jhansi, Uttar Pradesh, India; ^3^ Paleogenomics Laboratory, European University at St Petersburg, St Petersburg, Russia; ^4^ Palli Shiksha Bhavana, Visva Bharati, Sriniketan, West Bengal, India; ^5^ Fish Genetics and Biotechnology Division, ICAR-Central Institute of Freshwater Aquaculture, Bhubaneswar, Odisha, India; ^6^ Feed and Nutrition, Institute of Marine Research, Bergen, Norway; ^7^ Department of Renewable Marine Resources, Instituto de Ciencias del Mar (ICM)-CSIC, Barcelona, Spain

**Keywords:** epigenetics, growth, methylation, mitogenome, nanopore sequencing, sexual dimorphism

## Abstract

For aquaculture to be sustainable, it is very important to improve the growth rates of farmed fish by choosing the right species and their management. However, the integration of epigenetic markers in selective breeding programs remains underdeveloped, mainly due to limited understanding, particularly regarding DNA methylation’s heritability and its functional impact on growth traits. This gap is even more pronounced in mitochondrial epigenetics, despite mitochondria’s critical role in energy production and growth regulation, making it an important but underexplored area in aquaculture breeding strategies. The present study aimed to explore the relationship between differential mitogenome methylation and its role in growth rates and sexual dimorphism in Nile tilapia (*Oreochromis niloticus*). Nanopore sequencing was employed to compare mtDNA methylation patterns between fast- and slow-growing individuals, as well as between sexes. We found significant differences in mtDNA methylation, with males exhibiting higher growth rates and distinct methylation patterns in genes related to the electron transport chain, such as *ND5*, *ATP6* and *CYTB*. This suggests a link between mitochondrial function and growth. Moreover, several differentially methylated sites were identified, including hypomethylation in genes associated with oxidative phosphorylation, which correlated with increased growth. Notably, larger individuals showed significant hypomethylation in *ND5*, *ND6* and *COX1*, potentially enhancing ATP production. The differentially methylated positions across mitogenome may drive enhanced growth by optimizing mitochondrial function for higher energy output. Our study provides valuable insights for selective breeding programs to enhance growth traits, emphasizing the need for future research on the functional role of these epigenetic changes in sustainable aquaculture.

## 1 Introduction

Optimizing fish growth is crucial for aquaculture sustainability, directly influencing food security and ecosystem management. Sustainable practices, including genetic selection, tailored nutrition, and environmental optimization, are essential for maximizing growth efficiency and production yields (Naylor et al., 2021). Additionally, research explores the effects of disease, stress, and climate change on fish growth, guiding conservation efforts and efficient fish farming practices (Marcos-López et al., 2010). Latest research on this important trait aims to understand the genes and genetic variations that influence growth rates in fish species. Scientists use techniques like QTL mapping, marker-assisted selection, and genome sequencing to identify growth-related genes (Li and He, 2014; Sciara et al., 2018; Ji et al., 2019). Moreover, researchers have also explored transgenics and gene editing methods to enhance growth traits (Cui et al., 2017; Wang et al., 2022). However, ethical and environmental considerations are important when applying these findings to ensure long-term sustainability. Moreover, understanding the molecular mechanisms of fish growth provides valuable insights that can be applied to improve aquaculture practices.

*Oreochromis niloticus*, commonly known as Nile tilapia, is a freshwater fish species belonging to the Cichlidae family. It is native to the Nile River basin in Africa but has been introduced and widely distributed in various freshwater ecosystems around the world due to its adaptability and commercial value (Canonico et al., 2005). Nile tilapia is known for its economic importance in aquaculture and serves as a valuable food source in many countries. This fish species exhibits a sexually dimorphic pattern, meaning that males and females of this species display distinct physical characteristics and reproductive behaviours (Lind et al., 2015; Mehrim et al., 2019). The sexual dimorphism in Nile tilapia also extends to various physiological and molecular aspects. There have been observations of differential growth rates between males and females, with males typically growing faster and larger than females. Interestingly, in mixed-sex culture of *Oreochromis niloticus*, it has been observed that the fish attain sexual maturity before reaching market size, leading to uncontrolled reproduction in grow-out ponds or tanks (Chen et al., 2018). This results in a portion of the stock being unsuitable for marketing purposes. In addition to this, the irregular growth pattern in male and female *O. niloticus* has been discussed recently (Lind et al., 2015). In captivity, male *O. niloticus* size ranged from 18 to 414 g at 6 months of age, while females ranged from 13 to 291 g. Understanding the underlying mechanisms responsible for sexual dimorphism in growth can provide valuable insights into the biology and reproductive strategies of this species. In recent years, researchers have begun investigating the role of epigenetic modifications, such as DNA methylation, in mediating sexual dimorphism and growth-related traits in various organisms including Nile tilapia (Nedoluzhko et al., 2021). Moreover, growth is also linked with mitochondrial efficiency (Salin et al., 2019). This is why we hypothesize that, mtDNA methylation plays a vital role in fish growth.

Fish growth is regulated by complex cellular pathways driven by hormones like growth hormone and insulin, which coordinate key processes such as nutrient uptake, metabolism, nutrient sensing, growth factor signalling, and gene expression regulation (Carmona-Antoñanzas et al., 2014). These molecular mechanisms rely on ATP, which supplies the energy necessary for processes such as enzymatic reactions, active transport, muscle contraction, signal transduction, as well as DNA, RNA synthesis, and cellular respiration (Dunn and Grider, 2020). The energy currency, i.e., ATP is readily accessible and fuels various cellular processes, making it vital for growth and development in organisms like fish. Since ATP synthesis primarily occurs in mitochondria, their efficiency directly determines the availability of ATP for essential functions.

Mitochondria, known as the cell’s powerhouse, are crucial for ATP production through oxidative phosphorylation and play a key role in growth. They have their own DNA, i.e., mitochondrial DNA (mtDNA) that is distinct from the cell’s nuclear DNA, encoding genes necessary for this process and for producing antioxidant enzymes to manage reactive oxygen species (ROS), preventing oxidative damage (Jarmuszkiewicz et al., 2015). Mitochondria also regulate intracellular calcium, essential for cellular signaling (El-Hattab and Scaglia, 2016). However, mtDNA mutations can disrupt these functions, leading to mitochondrial disorders affecting various organs due to impaired energy production (Haas et al., 2007). The specific regulation of mtDNA gene expression and its impact on cellular processes and disorders remains an area of ongoing research. This research is particularly significant as it explores how variations in mtDNA expression could affect not just mitochondrial function but also broader biological processes that govern fish growth.

Fish growth is influenced not only by molecular mechanisms, hormones, and mitochondria but also by environmental factors and epigenetic processes (Konstantinidis et al., 2023a; Konstantinidis et al., 2023b). Environmental conditions such as temperature, water quality, and food availability significantly impact fish growth. Epigenetic modifications, including DNA methylation and histone modifications, are key in regulating genes associated with growth (Bestor et al., 1994). DNA methylation, the addition of a methyl group to cytosine bases, can either silence or activate genes, affecting cellular identity and enabling genomic imprinting and transgenerational inheritance. These epigenetic modifications allow cells to respond to environmental shifts, highlighting the importance of both genetic and environmental factors in understanding growth mechanisms.

Methylation patterns in nuclear DNA are more extensive and often linked to gene regulation, particularly in promoter regions, whereas mtDNA typically shows more limited methylation, primarily in non-coding regions (Stoccoro and Coppedè, 2021). Strand-specific methylation has been observed in both nuclear and mtDNA, with potential regulatory effects on gene expression in nuclear DNA (Stoccoro and Coppedè, 2021). For mtDNA, strand-specific methylation, particularly on the heavy strand, could influence mitochondrial function, though this remains an area of ongoing research (Dou et al., 2019). However, the existence and significance of mtDNA methylation are still debated, with minimal evidence for its presence (Bicci et al., 2021; Guitton et al., 2022), many research have also found the existence of mtDNA methylation in both CpG and non-CpG context (Liao et al., 2015; Liao et al., 2016; Wang et al., 2020; Nedoluzhko et al., 2021). This controversy may stem from structural differences between nuclear and mitochondrial genomes and the limitations of techniques optimized for nuclear DNA methylation analysis (Leroux et al., 2021). Notably, mitochondria contain both 5-methylcytosine (5 mC) and 5-hydroxymethylcytosine (5hmC) at CpG dinucleotides (Shock et al., 2011). A mitochondria-targeted variant of DNA methyltransferase 1 (*DNMT1*), known as *mtDNMT1*, has been identified, suggesting a role in mtDNA methylation (Lopes, 2020). However, the exact functions of mtDNMT1 in mitochondrial gene regulation and biogenesis are not yet fully understood (Kikuchi et al., 2022). Further research is needed to elucidate the mechanisms and implications of mtDNA methylation in mitochondrial function and biogenesis.

Bisulfite sequencing, while a standard for DNA methylation analysis, faces limitations with human mtDNA due to its unique closed-circular-covalent topology, leading to inaccurate CpG methylation detection and substantial false positives, as demonstrated by Liu et al. (2016). The study of mtDNA is further complicated by its low abundance, the absence of specific detection tools, and isolation challenges (Low et al., 2003). Moreover, the relevance of mtDNA methylation and its evolutionary variations remain challenging areas (Stimpfel et al., 2018; Breton et al., 2021). Nanopore sequencing, as highlighted by Gouil and Keniry (2019), offers a superior alternative, enabling direct, real-time detection of multiple DNA modifications without the drawbacks of bisulfite conversion, thus preserving DNA integrity and avoiding PCR biases. Given the challenges associated with traditional bisulfite sequencing for analyzing mtDNA methylation, and the importance of understanding epigenetic mechanisms in growth and development, the analysis of mtDNA methylation in Nile tilapia presents an opportunity to apply nanopore sequencing techniques. This approach could provide crucial insights into the molecular mechanisms behind sexual dimorphism and growth variations in this economically important species.

Thus, the present study aims to explore the relationship between differential mitogenome methylation in growth of *O. niloticus*, shedding light on the potential influence of epigenetic factors on this important biological process.

## 2 Materials and methods

### 2.1 Ethical statement

The current research was granted approval by Nord University’s (Bodø, Norway) ethics board and was also authorized by the Norwegian Animal Research Authority under FOTS ID 1042. All animal-related procedures strictly adhered to the guidelines set forth in the EU Directive 2010/63 concerning the use of animals for scientific purposes.

### 2.2 Sampling

The Nile tilapia used in this study belong to the third generation (F3) of our in-house domestication program (Konstantinidis et al., 2020). The base population (F0) was established from wild Nile tilapia females captured at the River Nile in Luxor, Egypt. The fertilized eggs were collected via traditional fishing methods and transported to our research facilities in Bodø, Norway. Fertilized eggs were identified by the visible presence of developing embryos in the female’s buccal cavity, accompanied by swelling of the mouth and altered feeding behaviour, ensuring that only viable eggs were collected for transportation. Successive generations (F1, F2, and F3) were reared under standardized conditions in a recirculating aquaculture system (pH = 7.6, oxygen saturation = 100%, temperature = 28°C, and photoperiod of 11:13 dark). The fish were fed *ad libitum* with 0.15–0.8 mm Amber Neptun pellets (Skretting, Norway). This consistent environment ensured minimal external influences on the selection process. For each generation, the fish were measured for total length (cm) and body weight (g) using a digital calliper and electronic balance, respectively, to monitor growth performance and ensure standardized data collection. The full-sib fish groups were marked based on size as slow-growing male (SM) (size between 14 and 17 cm), slow-growing female (SF) (size between 10 and 14.3 cm), fast-growing male (BM) (size between 24.5 and 31 cm) and fast-growing female (BF) (size between 21.1 and 26.4 cm). There were 10 fish specimens per group. Prior to sampling, six fish from each group were euthanized with clove oil (Sigma Aldrich, UNITED STATES) using a 1:10 mix of 15 mL clove oil in 95% ethanol diluted in 10 L of freshwater. Fast muscle (white muscle) samples were collected from the specimens left dorsal quadrant, followed by snap-frozen in liquid nitrogen and stored at −80°С until DNA extraction.

### 2.3 DNA isolation

The genomic DNA was extracted from the samples (six per group) using the DNeasy Blood and Tissue kit (Qiagen, Germany), following the manufacturer’s instructions. To assess DNA purity, a NanoDrop ND-1000 spectrophotometer (Thermofisher Scientific, United States), was used, while its quantity and quality were determined using Qubit (Thermofisher Scientific, United States) and Tape Station Genomic DNA ScreenTape Assay (Agilent Technologies, United States). Though we initially sampled ten fish to account for biological variation and ensure the reliability of our results. However, we selected six fish based on the quality and quantity of extracted DNA, ensuring that only high-quality samples were used for the mtDNA methylation study to maintain the accuracy and reproducibility of the findings.

### 2.4 Restriction enzyme digestion

All the DNA samples from experimental groups were digested with *DraI* (NEB, United States) and *EcoRV-HF* (NEB, United States) restriction enzymes according to manufacturer’s protocols. *DraI* that cuts at 5′ TTT↓AAA 3′, has four restriction sites on mtDNA. Similarly, *EcoRV* cuts at 5′ GAT↓ATC 3′ and has two restriction sites on mtDNA. In a 50 µL reaction, 10X rCutSmart Buffer 5 μL, *DraI* enzyme 1 µL (4 units), *EcoRV* enzyme 1 µL (4 units), DNA template 1 µg and nuclease-free water was mixed. The reaction mix was incubated at 37°C for 30 min followed by heat inactivation at 65°C for 20 min, for complete restriction enzyme digestion of DNA samples.

### 2.5 Library preparation and nanopore sequencing

Genomic DNA libraries for Oxford Nanopore Technologies (ONT) sequencing were constructed for each specimen using Ligation Sequencing Kit SQK-LSK109 (ONT, United Kingdom), following the standard ONT protocol and adaptive sampling. For the purpose of adaptive sampling Nile tilapia mitogenome (NCBI accession number MW149239.1) sequence fasta file from our previous study was used (Nedoluzhko et al., 2021). Eight DNA samples (two from each group) were sequenced on a single R9.4.1 FLO-MIN-106 flow cell. A total of three flow cells were used to sequence all 24 samples from all the groups. Each sample utilized 1 μg of initial DNA for ONT sequencing library construction. Barcoding of samples was performed using the ONT kit EXP-NBD104, and the pooled libraries were subjected to ONT sequencing on a MinION Mk1c device until the end of flow cell life, i.e., usually set to 72 h by default on the sequencer.

### 2.6 Basecalling and adapter trimming

To process the raw sequencing reads obtained from the four groups of Nile tilapia samples (SM, SF, BM, BF), basecalling was performed using Dorado v0.7.2 (https://github.com/nanoporetech/dorado). Dorado, developed by Oxford Nanopore Technologies, utilizes deep learning algorithms to convert raw electrical signals (in.pod5 file) from the MinION and other nanopore devices into nucleotide sequences. The basecalling process was carried out using the pretrained model specifically optimized for R9.4.1 flow cells, which corresponds to the chemistry used in the sequencing runs, i.e., dna_r9.4.1_e8_sup@v3.3. For basecalling the raw reads, stored in the Pod5 file format, were taken directly into Dorado. The basecalling was executed using the following command-line parameters, dorado basecaller sup,5mCG pod5s/> calls.bam. This command automatically trims barcodes and adapters from raw reads.

### 2.7 Methylation calls extraction and analysis

**FIGURE 1:**
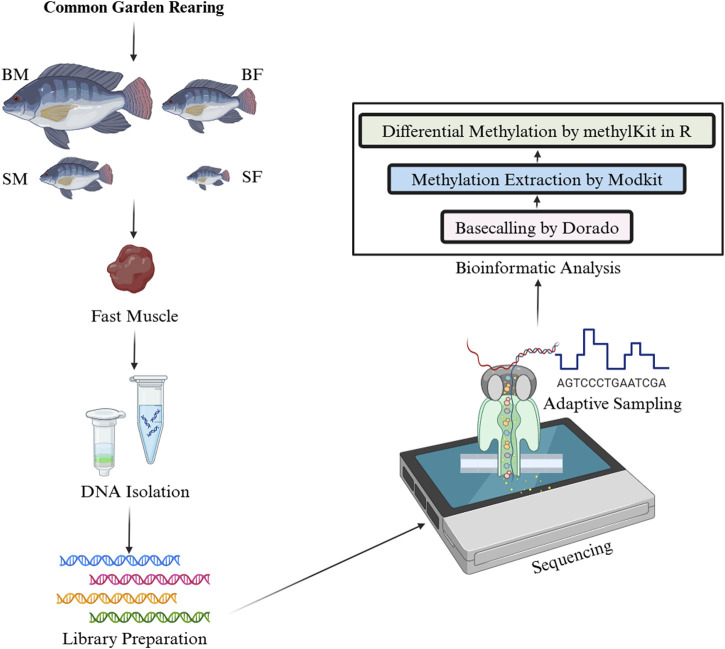
Workflow for the analysis of differential DNA methylation in Nile tilapia from a common garden rearing. This infographic illustrates the experimental and analytical steps involved in studying the mtDNA methylation patterns in four groups: slow-growing male (SM), slow-growing female (SF), fast-growing male (BM) and fast-growing female (BF). First, DNA was isolated from fast muscle samples, followed by ligation-based library preparation for Nanopore sequencing. The sequence data were then processed using Dorado for basecalling, with subsequent methylation extraction performed by ModKit. Finally, differential methylation analysis was conducted using methylKit in R.

The bam files generated from Dorado v0.7.2 contains information for modified bases in MM/ML tags. These bam files were used in Modkit v0.3.1 (https://github.com/nanoporetech/modkit) to extract methylation data in bed format. The bed files were later converted to a format suitable for methylKit v1.26.0 in excel. The per-site methylation was calculated in methylKit as the ratio of total number of sites counted as methylated to that of total number of sites in that position or the coverage. Then the differential methylation was analysed in CpG context using methylKit v1.26.0 in R at adjusted *p-value* ≤ 0.05. The differential methylation was analysed between SM vs. BM and SF vs. BF. The methodology and bioinformatics analysis followed has been outlined in Figure 1.

### 2.8 Regression analysis

To assess the relationship between site-specific mtDNA methylation and somatic growth, we conducted a series of linear regressions using R v2024.12.1. For each methylation site, a separate linear model was fitted with fish weight as the dependent variable and methylation percentage as the independent variable: Weight∼Methylation_*i*_, where Methylation_*i*_ refers to the methylation level at position *i*. Only numeric methylation data were used for modelling, excluding group identifiers. The regression loop iterated over all methylation positions, extracting the slope (estimate), p-value, and coefficient of determination (*R* ^2^) from each model. Multiple testing correction was applied to the p-values using the Benjamini–Hochberg false discovery rate (FDR) method. Methylation positions with an FDR below 0.05 were considered statistically significant. The strength and direction of associations were interpreted based on regression slope estimates and corresponding *R* ^2^ values. For each significant methylation site, scatter plots with regression lines were generated using the ggplot2 package. Plots depicted methylation percentage *versus* fish weight, with samples colored by group. Regression trends were overlaid with 95% confidence intervals.

### 2.9 Principal component analysis (PCA)

To explore patterns of variation in mtDNA methylation and assess whether epigenetic signatures were more strongly associate with sex or growth rate, we performed a PCA using R v2024.12.1 with packages tidyverse v2.0.0, ggfortify v0.4.17 and vegan v2.6-10. Methylation percentage data were first extracted from the dataset, excluding metadata columns (e.g., sample group and weight). The methylation matrix was then mean-centered and scaled before applying PCA via the prcomp () function. Sample group identifiers were parsed to classify individuals by sex (Male: SM, BM; Female: SF, BF) and by growth phenotype (Slow-growing: SM, SF; Fast-growing: BM, BF). The resulting principal component scores were visualized using ggplot2, with 95% confidence ellipses fitted to each grouping (SexGroup and GrowthGroup) to assess clustering tendencies. Variance explained by the first two principal components was included on axis labels. To statistically assess the influence of sex and growth rate on overall methylation profiles, we conducted permutational multivariate analysis of variance (PERMANOVA) using the adonis2 () function from the vegan package. The analysis used Euclidean distance and 999 permutations to test for significant group-level separation in the methylation matrix.

### 2.10 Statistical analysis

The statistical significance of differences in growth measurements across the four fish groups (SM, SF, BM, BF) was assessed using one-way ANOVA, followed by Post-hoc pairwise comparisons using Tukey’s Honest Significant Difference (HSD) test, which controls the family-wise error rate to account for multiple comparisons among groups. Visual representations of methylation data were created using ggplot2 (Wickham, 2011) in R, providing a clear visualization of the methylation landscape across the mitogenome. Circos plot was generated, using Circa v1.2.2 (http://omgenomics.com/circa) to visualize the distribution of methylation across different mitochondrial genes.

## 3 Results

### 3.1 Adaptive sampling on the enrichment of mitogenome

The adaptive sampling after fragmenting mtDNA through restriction enzymes greatly improved the enrichment of mitogenome. On an average 8,346,306 reads were found per sample, out of which 6,856,653 reads were mapped to the reference mitogenome. So, on an average 82.15% reads were mapped to the mitogenome. The mean length of reads was found to be 2,356 bp. By utilizing adaptive sampling, we were able to selectively sequence mtDNA, enabling real-time enrichment by actively rejecting nuclear DNA (including NUMTs) during sequencing. This approach effectively minimized the impact of nuclear sequences on the methylation analysis.

### 3.2 Fish growth across groups

From the present study the growth variation was measured among different sex and size classes of Nile tilapia (*O. niloticus*) in a mixed-sex culture system. The growth was measured in terms of weight, of six replicates each from four distinct groups: slow-growing male (SM), slow-growing female (SF), fast-growing male (BM) and fast-growing female (BF) (Supplementary Material: Supplementary_Table.docx- Supplementary Table S1). The weight measurements of the fish revealed a significant variation between the groups. The BM group showed the highest average weight, indicating a more rapid growth rate compared to other groups. The data recorded for this group ranged from 268 g to 382 g, with an average weight significantly higher than the other groups. In contrast, the SF group showed the lowest weight rate.

**FIGURE 2:**
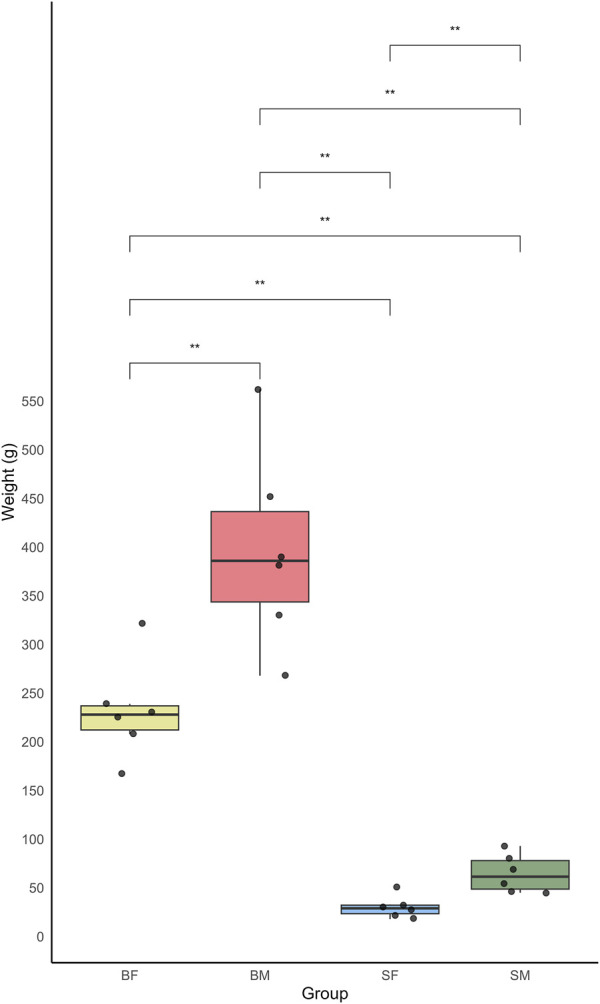
Boxplot showing the weight distribution of four groups of Nile tilapia: slow-growing male (SM), slow-growing female (SF), fast-growing male (BM) and fast-growing female (BF). The y-axis represents the weight in grams, while the x-axis indicates the different fish groups. Each boxplot displays the median weight and interquartile range, with whiskers extending to 1.5 times the interquartile range. Outliers are represented by individual points.

The boxplot analysis of the weights further underscored these differences, clearly depicting the upper and lower quartiles, thus highlighting the broader range of weights in the BM compared to the relatively narrow weight range observed in the SF (Figure 2). The mean weights for each group were as follows: BM > BF > SM > SF.

### 3.3 Methylated positions across mitogenome

**FIGURE 3:**
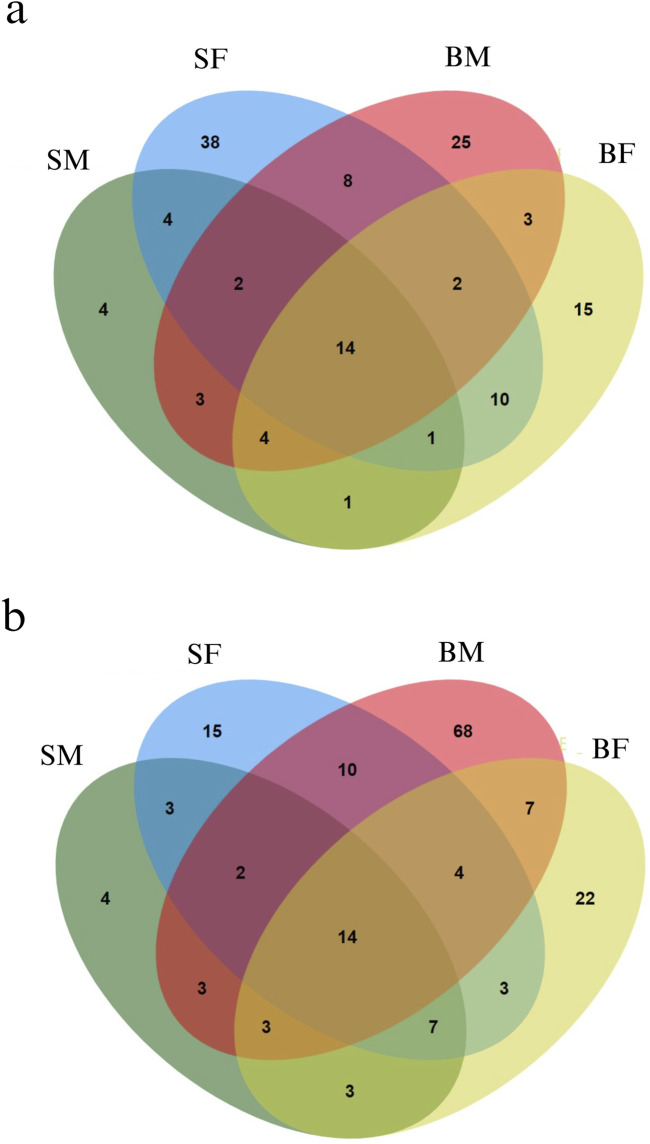
Venn diagrams representing the distribution of methylated positions across the mitogenome in Nile tilapia growth groups. **(a)** Methylated positions across the plus strand of the mitogenome. Each ellipse represents a group with the number indicating unique and shared methylated positions among slow-growing male (SM), slow-growing female (SF), fast-growing male (BM) and fast-growing female (BF) groups. **(b)** Minus strand methylated positions across the mitogenome. Similar to the plus strand, each ellipse corresponds to one of the growth groups, illustrating the overlap and unique methylated positions within each group.

From the methylKit analysis, sites in the mitogenome with methylation levels greater than 25% were considered as methylated positions, while those with methylation levels below 25% were classified as unmethylated (Supplementary Material: Per_Site_Methylation.xlsx). From the analysis we identified several methylated positions across mitogenome unique to each group, reflecting potential group-specific regulatory mechanisms of gene expression influenced by methylation (Figure 3). In addition to this, our study also uncovered positions that were commonly methylated across all four groups. Moreover, the strand specific methylated positions were also analysed for plus (heavy) and minus (light) strands as shown in Figures 3a,b, respectively.

#### 3.3.1 Complex I genes

The genes linked to Complex I of the electron transport chain (ETC) i.e., *ND1*, *ND2*, *ND3*, *ND4*, *ND4L*, *ND5* and *ND6* were found to be methylated in CpG context across mitogenomes of SM, SF, BM and BF. A total of 16, 31, 25 and 16 positions were found to be methylated in the plus strand of SM, SF, BM and BF, respectively. Similarly, 16, 22, 47 and 20 positions were found to be methylated in the minus strand of SM, SF, BM and BF, respectively. On the plus strand positions 4360, 9848, 12,053, 13,220, 13,505 and 13,883 were found to be methylated in all the four groups across the mitogenome.

#### 3.3.2 Complex III genes

The *CYTB* gene linked to Complex III of the ETC was found to be methylated across mitogenomes of SM, SF, BM and BF. A total of 2, 6, 2 and 4 positions were methylated in the plus strand of SM, SF, BM and BF, respectively. Similarly, 4, 5, 9 and 5 positions were found to be methylated in the minus strand of SM, SF, BM and BF, respectively. On the plus strand positions 14,946, 15,524 and in the minus strand position 14,500 were methylated in all the four groups across the mitogenome.

#### 3.3.3 Complex IV genes

The *COX1, COX2 and COX3* genes linked to Complex IV of the ETC were methylated in CpG context across mitogenomes of SM, SF, BM and BF. A total of 7, 14, 14 and 9 positions were found to be methylated in the plus strand of SM, SF, BM and BF, respectively. Similarly, 9, 12, 26 and 18 positions were found to be methylated in the minus strand of SM, SF, BM and BF, respectively. On the plus strand positions 6809, 7312, 7754 and in the minus strand position 6034, 6796 were found to be methylated in all the four groups across the mitogenome.

#### 3.3.4 Complex V genes

The *ATP6 and ATP8* genes linked to Complex V of the ETC were found to be methylated in CpG context across mitogenomes of SM, SF, BM and BF. A total of 3, 6, 3 and 3 positions were found to be methylated in the plus strand of SM, SF, BM and BF, respectively. Similarly, 3, 3, 7 and 3 positions were found to be methylated in the minus strand of SM, SF, BM and BF, respectively. On the plus strand positions 8365, 8520, 8603 and in the minus strand position 8602, 8676 were found to be methylated in all the four groups across the mitogenome.

#### 3.3.5 rRNA genes

Two rRNA genes, i.e. *16s rRNA* and *12s rRNA* were found to be methylated in CpG context across mitogenomes of SM, SF, BM and BF. In case of *12s rRNA* gene, a total of 1, 6, 6 and 3 positions were found to be methylated in the plus strand and 2, 4, 4 and 4 positions were found to be methylated in the minus strand of SM, SF, BM and BF, respectively across mitogenomes. Similarly, in case of *16s rRNA* gene, a total of 1, 8, 5 and 7 positions were found to be methylated in the plus strand and 1, 7, 8 and 2 positions were found to be methylated in the minus strand of SM, SF, BM and BF, respectively across mitogenomes.

#### 3.3.6 tRNA genes

The tRNA genes were found to be methylated in CpG context across mitogenomes of SM, SF, BM and BF. A total of 2, 5, 5 and 3 positions were found to be methylated in the plus strand of SM, SF, BM and BF, respectively. Similarly, 3, 4, 8 and 5 positions were found to be methylated in the minus strand of SM, SF, BM and BF, respectively. On the plus strand positions 10,066 and in the minus strand position 5278 were found to be methylated in all the four groups across the mitogenome.

#### 3.3.7 D-loop

The D-loop region was also found to be methylated in CpG context across mitogenomes of SM, SF, BM and BF. A total of 1, 3, 1 and 1 positions were found to be methylated in the plus strand of SM, SF, BM and BF, respectively. But in case of minus strand, position 16,518 in SM and position 16,425 in BF were found to be methylated only.

#### 3.3.8 Methylated positions specific to a particular group

Interestingly, it was found that, 4, 38, 25 and 15 positions were methylated exclusively in SM, SF, BM and BF, respectively in plus strand. Similarly, 4, 15, 68 and 22 positions were methylated exclusively in SM, SF, BM and BF, respectively in minus strand.

#### 3.3.9 Commonly methylated positions across the four groups

**FIGURE 4:**
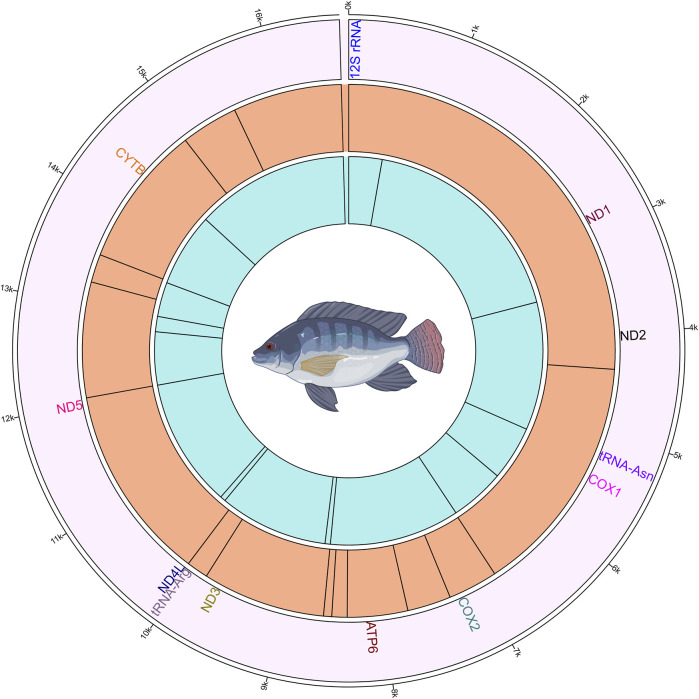
Circos plot showing the mitogenome and the methylated positions in common to all four Nile tilapia growth groups. The outermost ring (first ring from the outside) represents the mitogenome. The second ring shows the gene names those have commonly methylated positions. The third ring illustrates the methylated positions on the plus strand, indicated by black lines. The innermost (fourth) ring depicts the methylated positions on the minus strand, indicated by black lines.

The methylation analysis revealed several positions in the mtDNA that were commonly methylated across the different groups of fish. These positions and their corresponding genes are shown in Figure 4. In the plus strand, these methylated positions were found in *ND2, COX1, COX2, ATP6, ND3, tRNA-Arg, ND5* and *CYTB* genes. In the minus strand, these methylated positions were found in *12S rRNA, ND1, tRNA-Asn, COX1, ATP6, ND4L, ND5* and *CYTB* genes.

### 3.4 Differentially methylated positions across mitogenome

#### 3.4.1 Differential methylation based on size

##### 3.4.1.1 Slow-growing male (SM) vs. fast-growing male (BM)

**FIGURE 5:**
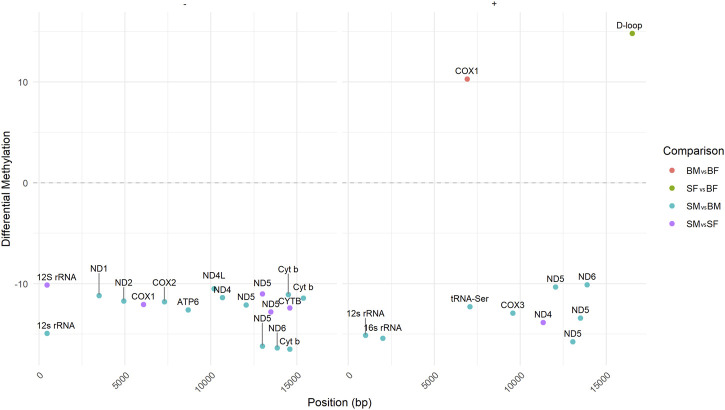
Differentially Methylated Positions (DMPs) across the mitogenome in Nile tilapia. The figure illustrates the hypo- and hypermethylated positions in the mitochondrial genome for four comparisons: SM vs. BM (SMBM) (in blue color), SF vs. BF (SFBF) (in green color), SM vs. SF (SMSF) (in purple color) and BM vs. BF (BMBF) (in orange color). DMPs are indicated for both the plus and minus strands of the mitogenome in different genes.

In the differential methylation analysis between SM and BM fish groups (taking BM as control) across different genes in the mitogenome, several significant differences were observed. The analysis showed specific cytosine positions that exhibited statistically significant changes in methylation levels (Table 1). Significant differential methylation was noted at multiple sites across various mitochondrial genes (Figure 5). The differential methylation analysis between SM and BM Nile tilapia revealed significant epigenetic changes in several mitochondrial genes across both the plus and minus strands. In the plus strand, notable hypomethylation was observed in the *12s rRNA* gene (−15.12%), *16s rRNA* gene (−15.41%), and *ND5* gene at multiple positions (−10.31% to −15.75%). Additionally, hypomethylation was detected in *tRNA-Ser* (−12.28%), *COX3* (−12.92%), and *ND6* (−10.09%).

**TABLE 1:** Comparative analysis of methylation differences between SM vs. BM, SF vs. BF, SM vs. SF and BM vs. BF across various mitochondrial genes, from methylKit in R. The table details the specific methylated positions, gene name, the strand orientation (+ or -), the methylation difference expressed in percentage, and the adjusted p-values highlighting the statistical significance of each difference. SM: slow-growing male, SF: slow-growing female, BM: fast-growing male (BM) and BF: fast-growing female.

Position	Gene name	Methylation difference	Adj. *p-value*	Comparison
Plus strand				
11,329	*ND4*	−13.84	6.69E-06	SMSF
993	*12s rRNA*	−15.12	2.30E-08	SMBM
2007	*16s rRNA*	−15.41	3.29E-08	SMBM
7071	*tRNA-Ser*	−12.28	1.07E-05	SMBM
9567	*COX3*	−12.92	2.99E-06	SMBM
12,053	*ND5*	−10.31	1.15E-04	SMBM
13,061	*ND5*	−15.75	1.23E-10	SMBM
13,505	*ND5*	−13.39	3.07E-08	SMBM
13,883	*ND6*	−10.09	2.49E-05	SMBM
16,519	*D-loop*	14.81	5.67E-05	SFBF
6912	*COX1*	10.28	1.45E-02	BMBF
461	*12s rRNA*	−10.13	7.50E-05	SMSF
6073	*COX1*	−12.05	1.24E-05	SMSF
13,000	*ND5*	−11.00	3.84E-07	SMSF
13,479	*ND5*	−12.78	1.19E-09	SMSF
14,587	*CYTB*	−12.41	1.18E-08	SMSF
Minus Strand				
461	*12s rRNA*	−14.92	1.18E-09	SMBM
3488	*ND1*	−11.17	2.49E-05	SMBM
4921	*ND2*	−11.71	1.07E-05	SMBM
7299	*COX2*	−11.78	1.07E-05	SMBM
8679	*ATP6*	−12.60	1.96E-06	SMBM
10,186	*ND4L*	−10.48	3.56E-05	SMBM
10,672	*ND4*	−11.37	8.00E-06	SMBM
12,052	*ND5*	−12.10	1.27E-06	SMBM
13,000	*ND5*	−16.19	3.69E-13	SMBM
13,856	*ND6*	−16.35	5.06E-14	SMBM
14,500	*CYTB*	−11.09	8.15E-07	SMBM
14,587	*CYTB*	−16.49	5.06E-14	SMBM
15,368	*CYTB*	−11.43	3.74E-07	SMBM

On the minus strand, significant hypomethylation was identified in the *12s rRNA* gene (−14.92%), *ND1* (−11.17%), *ND2* (−11.71%), *COX2* (−11.78%), *ATP6* (−12.60%), *ND4L* (−10.48%), *ND4* (−11.37%), *ND5* (−12.10% to −16.19%), *ND6* (−16.35%), and *Cyt b* at multiple positions (−11.09% to −16.49%) (Table 1).

##### 3.4.1.2 Slow-growing female (SF) vs. fast-growing female (BF)

The comparison between SF and BF (taking BF as control) revealed significant methylation differences in the mitochondrial *D-loop* region on the plus strand, with a hypermethylation of 14.81% (Figure 5). Additionally, in the BF group, *COX1* showed hypermethylation at one position with a difference of 10.28% (Table 1).

#### 3.4.2 Differential methylation based on sex

##### 3.4.2.1 Slow-growing male (SM) vs. slow-growing female (SF)

When comparing SM and SF (taking SF as control), several significant methylation differences were observed. In the plus strand, hypomethylation was noted in the *12s rRNA* gene (−10.13%), *COX1* (−12.05%), *ND5* at multiple positions (−11.00% to −12.78%), and *CYTB* (−12.41%) (Figure 5). Additionally, in the minus strand, the *12s rRNA* gene showed significant hypomethylation (−14.92%).

##### 3.4.2.2 Fast-growing male (BM) vs. fast-growing female (BF)

The differential methylation analysis between BM and BF (taking BF as control) highlighted significant changes in several mitochondrial genes (Figure 5). In the plus strand, *COX1* exhibited hypermethylation in BM with a difference of 10.28% (adj. *p-value* = 1.45E-02) (Table 1).

### 3.5 Regression analysis

#### 3.5.1 Heavy strand methylation

**FIGURE 6:**
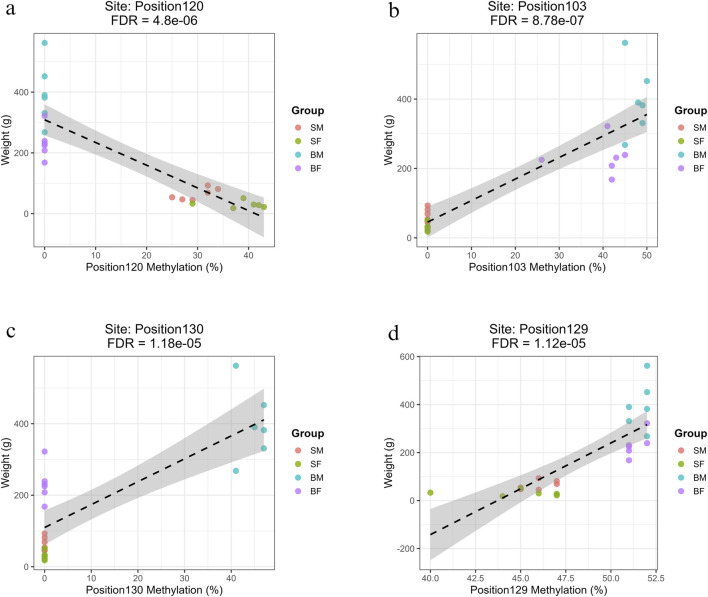
Regression plots showing the relationship between methylation percentage and fish body weight at mitochondrial positions on the forward strand with the highest *R* ^2^ values (>0.7) **(a)** Position120 (*ND5*) shows a strong negative correlation with weight in slow-growing fish (SM, SF). **(b)** Position103 (*ND4*) shows a strong positive correlation with weight in fast-growing fish (BM, BF). **(c)** Position130 (D-loop) shows a strong positive association specific to fast-growing males (BM). **(d)** Position129 (*CYTB*), a site common to all groups, shows a significant positive correlation with weight.

The sites with highest *R* ^2^ value (>0.7) have been shown for slow-growing, fast-growing, fast-growing males and common position in Figures 6a–d, respectively. The figures for the remaining significant positions have been given in Supplementary Material: Regression_Plus_Strand.zip.

##### 3.5.1.1 Methylation sites associated with slow growth (SM, SF)

Four methylation sites were significantly and negatively associated with weight specifically in slow-growing individuals (SM, SF). These were Position47 (*ND2*, Base 4273), Position80 (*COX3*, Base 8898), Position101 (*ND4*, Base 11,329) and Position120 (*ND5*, Base 13,311) (Supplementary Material: Regression.xlsx: Sheet Name- Plus_Strand). These sites were filtered based on the R value greater than 0.7 and represent epigenetic modifications localized in key oxidative phosphorylation genes. Among slow-growing fishes, several methylation sites exhibited strong negative slopes (ranging from −5.69 to −7.48), indicating that for every 1% increase in methylation at these loci, there was an associated decrease of approximately 5.7–7.5 g in fish weight.

##### 3.5.1.2 Methylation sites associated with fast growth (BM, BF)

Three sites were found to be significantly associated with increased weight in fast-growing males and females (BM, BF). These were Position103 (*ND4*, Base 11,414), Position114 (*ND5*, Base 12,730) and Position45 (*ND2*, Base 4126) (Supplementary Material: Regression.xlsx: Sheet Name- Plus_Strand). The strong positive slopes observed (ranging from 5.62 to 6.20) indicate that for every 1% increase in methylation at these loci, there was an associated increase of approximately 5–6 g in fish weight.

##### 3.5.1.3 Methylation sites specific to fast-growing males (BM)

A total of 25 methylated positions associated with higher weight specifically in BM individuals, exhibiting strong positive correlations (slopes 5.53–6.4) and high *R* ^2^ values (∼0.64–0.65). The affected genes include *ND1–ND4*, *ND2*, *ND3*, *COX1–COX3*, *CYTB*, and multiple tRNAs (e.g., *tRNA-Phe* and *tRNA-Asn*). The most relevant sites were Position1 (*tRNA-Phe*, Base 63), Position54 (*COX1*), and Position130 (D-loop) (Supplementary Material: Regression.xlsx: Sheet Name- Plus_Strand).

##### 3.5.1.4 Common methylation sites across all groups

Two sites were found to be significantly associated with growth across all fish groups, i.e., Position129 (Base 15,524) and Position125 (Base 14,946) (Supplementary Material: Regression.xlsx: Sheet Name- Plus_Strand). Interestingly, both common sites were within the *CYTB* gene, but with opposing directions of effect, suggesting potentially complex, context-dependent methylation regulation at this locus.

#### 3.5.2 Light strand methylation

**FIGURE 7:**
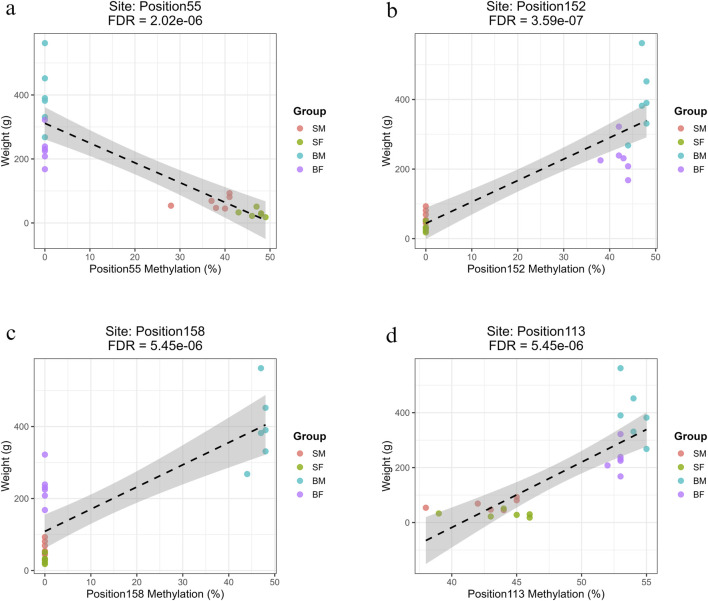
Regression plots showing the relationship between methylation percentage and body weight at mitochondrial positions on the reverse strand with the highest *R* ^2^ values (>0.7). **(a)** Position55 (*COX1*) shows a strong negative correlation with weight in slow-growing fish (SM, SF). **(b)** Position152 (*ND6*) shows a strong positive correlation with weight in fast-growing fish (BM, BF). **(c)** Position158 (*CYTB*) exhibits a strong positive association specifically in fast-growing males (BM). **(d)** Position113 (*ND4L*), a site common to all groups, shows a significant positive correlation with weight.

The positions with highest *R* ^2^ value (>0.7) have been shown for slow-growing, fast-growing, fast-growing males and common position in Figures 7a–d, respectively. The remaining significant correlations are illustrated in Supplementary Material: Regression_Minus_Strand.zip.

##### 3.5.2.1 Methylation sites associated with slow growth (SM, SF)

Three methylation sites showed strong negative associations with fish weight and were specific to slow-growing fish (SM and SF). These sites were in mitochondrial *12s rRNA* (Position4, Base 549), *16s rRNA* (Position22, Base 2442) and *COX1* regions (Position55, Base 5759) (Supplementary Material: Regression.xlsx: Sheet Name- Minus_Strand). The correlations exhibited strong negative slopes (ranging from −6.02 to −6.18), indicating that for every 1% increase in methylation at these loci, there was an associated decrease of approximately 6 g in fish weight.

##### 3.5.2.2 Methylation sites associated with fast growth (BM, BF)

Seven methylation sites showed strong positive correlations with weight in both BM and BF, and had *R* ^2^ values above 0.75, high positive slopes and highly significant FDR values. These were in multiple mitochondrial loci within genes involved in oxidative phosphorylation, namely, Position149 (*ND5*), Position152 (*ND6*), Position29 (*ND1*), Position54 and Position67 (*COX1*), Position81 and Position87 (*COX2*) (Supplementary Material: Regression.xlsx: Sheet Name- Minus_Strand).

##### 3.5.2.3 Methylation sites specific to fast-growing males (BM)

A large set of 68 positions were significantly associated with increased body weight specifically in the BM group. These methylation sites were found across mitochondrial genes involved in respiration and protein synthesis, including *ND1–ND6*, *COX1–COX3*, *CYTB*, *ATP6*, *ATP8*, and various tRNAs. Some of the most significant positions were Position8 (*12S rRNA*), Position124 (*ND4*) and Position158 (*CYTB*) (Supplementary Material: Regression.xlsx: Sheet Name- Minus_Strand). The prevalence of mitochondrial coding and tRNA genes in these significant results implies a potential epigenetic mechanism specific to fast-growing males.

##### 3.5.2.4 Common methylation sites across all groups

Only Position113 (ND4L, Base 10,186) was found to be significantly associated with growth across all groups (SM, SF, BM, BF), with a notably high positive slope of 23.79 and *R* ^2^ = 0.67 (FDR = 5.45 × 10^−6^) (Supplementary Material: Regression.xlsx: Sheet Name- Minus_Strand).

### 3.6 PCA analysis of mitochondrial methylation with sex and growth rate

#### 3.6.1 Heavy strand

**FIGURE 8:**
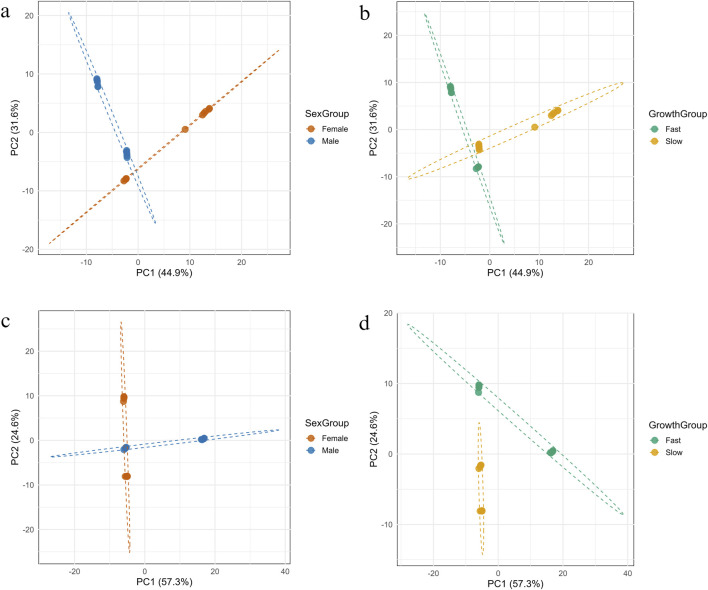
Principal Component Analysis (PCA) of mtDNA methylation patterns on the forward **(a,b)** and reverse **(c,d)** strands, showing separation of fish samples by sex and growth group.

The PCA of heavy strand mtDNA methylation revealed a clear separation among individuals based on both sex and growth rate (Figures 8a,b, respectively). The first two principal components accounted for a cumulative 76.5% of the total variance in the dataset, with PC1 explaining 44.9% and PC2 explaining 31.6%. When grouped by sex, male and female samples formed distinct clusters in the PCA space, separated primarily along PC1. Confidence ellipses showed minimal overlap between groups, indicating sex-specific methylated positions clustered. Similarly, grouping by growth rate (fast vs. slow) also resulted in separation along both PC1 and PC2. Although there was slightly more overlap compared to sex-based clustering, the fast- and slow-growing groups still formed distinguishable clusters. PERMANOVA analysis confirmed that both sex and growth group explained a significant portion of the variance in methylation patterns. Sex accounted for 34.7% of the total variance (*R* ^2^ = 0.347, F = 11.71, p = 0.001), while growth rate accounted for 33.7% (*R* ^2^ = 0.337, F = 11.18, p = 0.001).

#### 3.6.2 Light strand

The PCA of methylation on the light strand of mtDNA revealed distinct separation between sample groups when categorized by both sex and growth rate (Figures 8c,d, respectively). The first two principal components showed a total of 81.9% of the variance, with PC1 accounting for 57.3% and PC2 for 24.6%. When samples were grouped by growth rate, individuals from fast- and slow-growing groups formed clearly distinct clusters. The separation occurred predominantly along PC1, with minimal overlaps between groups, suggesting strong methylation pattern differentiation associated with growth phenotype. In contrast, grouping by sex showed partial separation primarily along PC1, but with greater overlap in PCA space. While male and female samples showed directional trends, the distinction was less pronounced than that observed for growth. These observations were supported by PERMANOVA analysis. Growth rate accounted for a greater proportion of variance (*R* ^2^ = 0.337, F = 11.18, p = 0.001) than sex (*R* ^2^ = 0.301, F = 9.47, p = 0.001).

## 4 Discussion

### 4.1 Growth dynamics in mixed-sex culture system

Culturing fish in a mixed-sex system enhances genetic diversity, improving resilience against environmental stressors and diseases (Bouza et al., 1997; Ye et al., 2022). It also supports natural reproduction, reducing artificial breeding costs (Asturiano, 2020; Clearinghouse, 2022). While mixed-sex cultures can lead to balanced growth and higher yields, studies indicate no significant overall difference compared to mono-sex culture (Dan and Little, 2000). However, challenges such as differential growth patterns persist (Donaldson, 1994; Toguyeni et al., 2002; Chakraborty and Banerjee, 2010).

This study highlights significant growth variations among different sex and size classes of *O. niloticus*, aligning with prior findings (Lind et al., 2015). BM fish exhibited the highest average weights, while SF had the lowest, likely due to sex-specific growth influences. Males typically grow faster due to higher metabolic efficiency and better feed conversion (Schreiber et al., 1998; Toguyeni et al., 2002; Mamun et al., 2007; Lind et al., 2015; De Verdal et al., 2017). Understanding these variations is crucial for optimizing aquaculture practices and selective breeding programs.

Further, differential growth patterns in mixed-sex Nile tilapia may be linked to mtDNA methylation variations affecting energy metabolism and cellular respiration. Genes involved in ATP production exhibit methylation differences, potentially influencing metabolic efficiency (Eya et al., 2015; Mieiro et al., 2015; Maes et al., 2016). These findings underscore the role of mitochondrial epigenetics in growth regulation, presenting opportunities for targeted improvements in commercial fish farming.

### 4.2 Distinct mtDNA methylation sites linked to size variations

The methylation analysis of mtDNA across four groups of fish revealed specific positions of methylation that may be linked to growth in Nile tilapia. On the plus strand of mtDNA, 4, 38, 25, and 15 positions were found to be methylated exclusively in SM, SF, BM, and BF, respectively. On the minus strand of mtDNA, 4, 15, 68, and 22 positions were uniquely methylated in SM, SF, BM, and BF, respectively. These distinct methylation patterns suggest that DNA methylation might play a crucial role in the regulation of mitochondrial genes, contributing to the differential growth observed in these groups. The methylation of genes involved in mitochondrial function, such as those encoding components of the electron transport chain (*ND2, COX1, COX2, ATP6, ND3, ND5, CYTB*) and those involved in protein synthesis (*tRNA-Arg, 12S rRNA*), indicates a potential mechanism by which energy production and metabolic efficiency are regulated. The effect of mtDNA methylation on the regulation of genes encoding protein subunits of the ETC and oxidative phosphorylation system has also been reflected in earlier works (Gao et al., 2017). In particular, the unique methylation sites found in larger individuals (BM and BF) could reflect adaptations that enhance energy production and utilization, supporting increased growth. Conversely, the specific methylation patterns in smaller individuals (SM and SF) might reflect a different regulatory balance that limits growth, potentially as a response to environmental pressures or genetic factors. In an earlier work it has also been revealed that, specific regions in the mitochondrial genome with high levels of methylation, such as the D-Loop and genes like *ND2* and *ATP6* have been associated with various regulatory functions that could influence cellular and organismal phenotypes, including size (Devall et al., 2023). Additionally, the observed methylation differences could affect other indirect pathways related to growth. For example, altered mitochondrial function could impact cellular oxidative stress levels, signalling pathways, and metabolic homeostasis, all of which are critical for growth and development. Methylation changes in mitochondrial genes might also influence the expression of nuclear-encoded mitochondrial proteins, further affecting cellular energy dynamics. These methylation patterns could modulate hormonal signalling pathways by influencing mitochondrial function and energy availability, which are crucial for endocrine regulation. Hypomethylation in key mitochondrial genes, such as *ND5*, *ATP6*, and *COX1*, may enhance ATP production, supporting increased cellular energy demands for growth hormone (*GH*) synthesis and secretion. *GH* stimulates hepatic production of insulin-like growth factor 1 (*IGF-1*), which promotes protein synthesis, cell proliferation, and overall somatic growth. Additionally, altered mitochondrial efficiency due to differential methylation could affect the sensitivity of *GH* and *IGF-1* receptors by modulating intracellular signalling cascades such as the *PI3K/Akt* and *MAPK* pathways. These pathways regulate anabolic processes, including muscle growth and lipid metabolism, further contributing to differences in body size and growth rates.

The commonly methylated positions across all the groups indicates a potential fundamental regulatory mechanism in the mitochondrial genome. These methylated positions in different groups could imply essential roles in mitochondrial function and possibly in the broader physiological processes such as energy production and metabolic regulation. These positions might be playing a crucial role in the regulation of mitochondrial gene expression independent of size or sex. The presence of methylation in the genes in plus strand suggests a potential regulatory role in mitochondrial function and energy production. Methylation in the *ND2*, *COX1*, and *COX2* genes, which are involved in the electron transport chain, could influence the efficiency of oxidative phosphorylation and ATP synthesis. It is because methylation of the *ND2, COX1*, and *COX2* genes, could reduce transcription of these genes, impair RNA processing, and decrease the production of essential mitochondrial proteins. This disruption would hinder the efficiency of oxidative phosphorylation, leading to lower ATP synthesis and cellular energy deficits. Such effects would be particularly detrimental to tissues that rely heavily on mitochondrial function, like muscle and brain cells, ultimately impairing overall cellular metabolism and function. Similarly, *ATP6* is directly involved in ATP production, and its methylation could affect cellular energy dynamics. The alterations in the *ATP6* gene impairing the function of the ATP synthase complex, which is crucial for ATP production in mitochondria has also been studied in earlier works (Dautant et al., 2018). The methylation of *tRNA-Arg* might play a role in the translation of mitochondrial proteins, impacting the overall protein synthesis machinery within the mitochondria. The *ND5* and *CYTB* are also crucial components of the electron transport chain, and their methylation could indicate a broader regulatory mechanism affecting mitochondrial respiration and metabolic processes. On the minus strand, the methylated positions were found in the *12S rRNA*, *ND1*, *tRNA-Asn*, *COX1*, *ATP6*, *ND4L*, *ND5*, and *CYTB* genes. The methylation of *12S rRNA* suggests an influence on ribosomal function and mitochondrial protein synthesis, potentially affecting the overall efficiency of the mitochondrial translation system. *ND1* and *ND4L*, being part of the NADH dehydrogenase complex, indicate that methylation might regulate the initial steps of the electron transport chain. The presence of methylation in *tRNA-Asn* highlights the potential for regulation at the level of mitochondrial tRNA processing and stability. The methylation on the *COX1*, *ATP6*, *ND5*, and *CYTB* genes on both the strands underscores the importance of these genes in mitochondrial function and suggests that methylation could serve as a key regulatory mechanism across different levels of mitochondrial gene expression.

### 4.3 Differential methylation in mitochondrial complex I genes linked to size variation

The analysis of differential methylation between SM and BM showed significant insights into the epigenetic regulation of genes within the mitogenome, which are crucial for energy production and metabolic processes. Notably, several genes within Complex I of the ETC, including *ND1*, *ND2*, *ND4*, *ND4L*, *ND5* and *ND6*, showed changes in methylation patterns that may influence their expression and function. The phenotypic changes in the organism due to methylation in Complex I genes has also been studied previously (Lomba et al., 2010; Lozoya et al., 2018; Gardner et al., 2023). Methylation changes in these genes can affect their transcription and, consequently, the efficiency of ATP production. Although methylation changes in Complex I genes may not occur within the promoter regions and mitochondrial transcripts are polycistronic, these changes can still influence gene expression and mitochondrial function. Methylation in coding or intergenic regions can alter the secondary structure of mRNA, affecting RNA stability, processing, or translation efficiency. This could lead to reduced translation or improper processing of polycistronic transcripts, thereby decreasing the levels of individual Complex I proteins. Methylation might also impact the recruitment of regulatory proteins or RNA-binding factors that influence mRNA stability or translation, further impairing the production of functional Complex I subunits. These changes, even without directly affecting transcription, can disrupt the efficiency of oxidative phosphorylation and ATP synthesis. For instance, hypomethylation generally leads to increased gene expression (Chandler and Jones, 1988; Chen et al., 1998; Wilson et al., 2007; Ehrlich and Lacey, 2013). In the BM group, which exhibited hypomethylation at several sites within these genes, there may be an enhanced capacity for energy production, potentially explaining the observed larger somatic growth compared to SM.

The mitochondrial genes, especially those involved in the ETC, directly impact cellular energy availability. In fish, as in other vertebrates, growth rates are closely linked to energy metabolism. The differential methylation observed may alter the metabolic efficiency of these individuals, thereby influencing growth rates and sizes. Previous studies have highlighted the relationship between mitochondrial dysfunction and varied metabolic rates, which could be linked to differences in growth (Haque et al., 2016; Koch et al., 2021).

The differential methylation patterns observed between SM and BM could indicate adaptive epigenetic responses to environmental or physiological signals. In aquaculture, where growth rate and size at harvest are critical traits, understanding the epigenetic mechanisms that regulate these traits provides valuable insights for selective breeding programs. For example, selectively breeding individuals with methylation profiles conducive to higher growth rates could be a strategic approach to enhance productivity (Guppy et al., 2018; Houston et al., 2020; Rasal and Sundaray, 2020; Podgorniak et al., 2022). Our findings suggest that the methylation status of mitochondrial genes could be a key factor in the observed size differences between SM and BM tilapia. This association between epigenetic modifications and phenotypic traits aligns with studies in other species where DNA methylation has been linked to variations in phenotype, including stress response, aging, and development (Cheng et al., 2021; Liu et al., 2022).

As discussed before, in mixed-sex culture systems, differential growth rates between sexes often pose a significant challenge, with males typically outgrowing females, leading to uneven stock sizes and reduced marketability, as seen in our present study.

### 4.4 Sex-specific mtDNA methylation patterns linked to size and metabolic differences

The differential methylation analysis between SM vs. SF and BM vs. BF Nile tilapia groups has uncovered significant epigenetic variations within the mitochondrial genome. These variations could play a crucial role in understanding sex-specific differences in growth and metabolism, offering potential epigenetic markers for further studies.

In the comparison between SM vs. SF, significant hypomethylation was detected in several mitochondrial genes on both the plus and minus strands. Specifically, the *12s rRNA* gene showed hypomethylation in both plus and minus strand. Additionally, *COX1*, *ND5*, and *CYTB* showed hypomethylation on the plus strand. These genes play pivotal roles in mitochondrial function and energy production. The *12s rRNA* gene is involved in mitochondrial protein synthesis, while *COX1*, *ND5*, and *CYTB* are key components of the ETC and oxidative phosphorylation pathway (Kehrein et al., 2013). The hypomethylation in these genes suggests an increase in their expression levels, potentially enhancing mitochondrial efficiency and energy metabolism in SM compared to SF. Kehrein et al. (2013) discussed the pivotal roles of these genes in mitochondrial function, particularly in the ETC and oxidative phosphorylation, emphasizing their importance in energy production. Additionally, research has shown that hypomethylation can lead to increased gene expression, as demonstrated by Sharma et al. (2019), who found that hypomethylation in mitochondrial genes is associated with higher transcription and protein levels, potentially enhancing mitochondrial function. Studies by Wallace and Fan (2010) further support this by highlighting how epigenetic modifications, including DNA methylation, influence the expression of mitochondrial genes involved in energy metabolism. Moreover, research by Bellizzi et al. (2012) links hypomethylation of mitochondrial genes to improved mitochondrial efficiency and ATP production, aligning with the idea that hypomethylation in genes such as *COX1*, *ND5*, and *CYTB* could upregulate expression and enhance mitochondrial energy metabolism. Enhanced mitochondrial function could lead to higher ATP production, which is crucial for supporting the increased energy demands associated with growth and metabolic activities in males. This is supported by previous studies indicating that hypomethylation can lead to upregulation of mitochondrial genes, improving metabolic capacity and growth performance (Jia et al., 2015).

The observed methylation differences between SM and SF may explain the distinct growth patterns seen in these groups. Males generally grow faster and larger than females, which could be linked to more efficient mitochondrial function driven by specific methylation patterns. Enhanced mitochondrial activity in males, facilitated by hypomethylation, would provide the necessary energy for rapid growth (Castegna et al., 2015; Crouse et al., 2022). The differential methylation patterns also suggest that males and females have adapted their mitochondrial function to meet sex-specific energy demands. For example, females may exhibit hypermethylation in genes involved in oxidative stress response, aligning with their reproductive needs and energy allocation strategies.

In the BM vs. BF comparison, significant hypermethylation was observed in the *COX1* gene on the plus strand in BM. The *COX1* is a crucial component of the mitochondrial electron transport chain, and hypermethylation in BM suggests a potential downregulation of *COX1* expression. This downregulation could lead to decreased mitochondrial efficiency and reduced ATP production, possibly as a metabolic adaptation to the larger body size and lower relative growth rate of BF compared to BM. Larger animals often have lower relative metabolic rates, despite potentially higher absolute metabolic rates (Nagy, 2005). This scaling of metabolic processes can influence mitochondrial function, particularly in animals with larger body sizes or slower growth rates. Slower growth and larger body sizes may lead to adjustments in mitochondrial efficiency to match lower energy demands (Kozłowski et al., 2020). In addition, previous reports showed that hypermethylation can repress gene expression, impacting mitochondrial function and metabolic processes (Minocherhomji et al., 2012; Mposhi et al., 2017).

### 4.5 Regression and PCA analysis for growth and sex-specific mitochondrial methylation

The observed positive association between mtDNA methylation and body weight in fast-growing fish in both heavy and light strands underscores the potential functional importance of mitochondrial epigenetics in growth regulation. Methylation at genes encoding components of Complex I (e.g., *ND2*, *ND4*, *ND5*) and Complex IV (*COX1*–*COX3*) likely enhances mitochondrial efficiency or bioenergetic output, aligning with the central role of these complexes in ATP production. This is consistent with findings in other vertebrates, where increased mitochondrial activity is linked to enhanced growth performance due to greater energy availability (Eya et al., 2011). Among these, most of the positions were linked to *ND* genes. *ND2* and *ND4* are known for their roles in proton translocation and respiratory chain function, both of which are crucial for sustaining high metabolic rates required for rapid growth (Mark et al., 2012). In contrast, methylation sites negatively correlated with growth were predominantly found in slow-growing fish and located in *ND* and *COX* genes. Hypomethylated sites in certain genes in the domestic fish modulates mitochondrial functions such as proteasomal degradation by negatively regulating an anti-apoptotic protein survivin (Podgorniak et al., 2022). It is plausible that methylation of these mitochondrial genes negatively correlating with growth may have a negative impact on mitochondrial function, leading to slower growth.

The PCA analysis of mtDNA methylation patterns revealed distinct clustering based on both sex and growth phenotype, highlighting the biological relevance of epigenetic variation in shaping these traits. On the heavy strand, sex differences were more prominent, with male and female samples forming non-overlapping clusters, suggesting sex-specific methylation signatures that may reflect divergent mitochondrial regulation or metabolic demands between sexes. In contrast, the light strand PCA emphasized growth-related methylation differences, with fast- and slow-growing individuals forming well-separated clusters and minimal overlap. This pattern suggests that methylation plays a critical role in modulating growth through mitochondrial pathways, possibly influencing gene expression or bioenergetic efficiency. Additionally, PERMANOVA confirmed that both sex and growth significantly contributed to methylation variation. These findings support the hypothesis that mitochondrial methylation is not random but structured according to physiological and developmental demands, potentially serving as an adaptive mechanism in fish to regulate energy metabolism and size differences.

## 5 Conclusion

The present study uncovers the possible role of mtDNA methylation in regulating size and sexual dimorphism in Nile tilapia. Using nanopore sequencing, we identified distinct mtDNA methylation patterns across different groups of Nile tilapia, specifically comparing slow and fast-growing males and females. Our findings suggest that these methylation differences may influence the efficiency of mitochondrial energy production and metabolic processes, thereby contributing to the observed variations in growth rates and sizes between the groups. Certain mitochondrial genes, particularly those involved in the ETC, including *ND5*, *ATP6*, *COX1*, and *CYTB*, exhibit differential methylation patterns that could regulate energy metabolism. Specifically, hypomethylation in genes like *ND5* and *COX1* in fast-growing individuals (BM and BF) likely enhances mitochondrial efficiency, supporting increased ATP production necessary for rapid growth. In contrast, specific hypermethylation patterns in slow-growing individuals (SM and SF) might indicate a more energy-conserving metabolic strategy. Moreover, the study highlights sex-specific methylation differences, which could explain the faster growth rates observed in males compared to females, potentially linked to the unique energy demands of each sex. These finding will help future studies for utilizing mitochondrial methylation profiles as epigenetic markers in selective breeding programs to optimize growth traits in aquaculture. By integrating mtDNA methylation data into breeding strategies, it may be possible to enhance productivity in Nile tilapia, improving energy efficiency and growth rates while maintaining genetic diversity and ecological balance in aquaculture systems. Our findings also underscore the importance of further research into the functional roles of these epigenetic modifications in shaping phenotypic traits and their broader implications for sustainable aquaculture practices.

## Data Availability

The sequencing data were deposited into the NCBI-SRA database under accession number SRR32750101- SRR32750124.

## References

[B1] Asturiano J. F. (2020). Chapter 14 improvements on the reproductive control of the european eel. Reproduction aquatic animals basic Biol. Aquac. Technol., 293–320. 10.1007/978-981-15-2290-1_15

[B2] Bellizzi D. D ‘Aquila P. Giordano M. Montesanto A. Passarino G. (2012). Global DNA methylation levels are modulated by mitochondrial DNA variants. Epigenomics 4, 17–27. 10.2217/epi.11.109 22332655

[B3] Bestor T. H. Chandler V. L. Feinberg A. P. (1994). Epigenetic effects in eukaryotic gene expression. Dev. Genet. 15, 458–462. 10.1002/dvg.1020150603 7834904

[B4] Bicci I. Calabrese C. Golder Z. J. Gomez-Duran A. Chinnery P. F. (2021). Single-molecule mitochondrial DNA sequencing shows no evidence of CpG methylation in human cells and tissues. Nucleic Acids Res. 49, 12757–12768. 10.1093/nar/gkab1179 34850165 PMC8682748

[B5] Bouza C. Sanchez L. Martinez P. (1997). Gene diversity analysis in natural populations and cultured stocks of turbot (*Scophthalmus maximus* L.). Anim. Genet. 28, 28–36. 10.1111/j.1365-2052.1997.00070.x

[B6] Breton S. Ghiselli F. Milani L. (2021). Mitochondrial short-term plastic responses and long-term evolutionary dynamics in animal species. Genome Biol. Evol. 13, evab084. 10.1093/gbe/evab084 33892508 PMC8290114

[B7] Canonico G. C. Arthington A. McCrary J. K. Thieme M. L. (2005). The effects of introduced tilapias on native biodiversity. Aquatic Conservation Mar. Freshw. Ecosyst. 15, 463–483. 10.1002/aqc.699

[B8] Carmona-Antoñanzas G. Tocher D. R. Martinez-Rubio L. Leaver M. J. (2014). Conservation of lipid metabolic gene transcriptional regulatory networks in fish and mammals. Gene 534, 1–9. 10.1016/j.gene.2013.10.040 24177230

[B9] Castegna A. Iacobazzi V. Infantino V. (2015). The mitochondrial side of epigenetics. Physiol. genomics 47, 299–307. 10.1152/physiolgenomics.00096.2014 26038395

[B10] Chakraborty S. B. Banerjee S. (2010). Comparative growth performance of mixed-sex and monosex nile tilapia population in freshwater cage culture system under Indian perspective. Int. J. Biol. 2, 44. 10.5539/ijb.v2n1p44

[B11] Chandler L. A. Jones P. A. (1988). “Hypomethylation of DNA in the regulation of gene expression,” in The molecular biology of cell determination and cell differentiation, 335–349. 10.1007/978-1-4615-6817-9_12 2481475

[B12] Chen J. Fan Z. Tan D. Jiang D. Wang D. (2018). A review of genetic advances related to sex control and manipulation in tilapia. J. World Aquac. Soc. 49, 277–291. 10.1111/jwas.12479

[B13] Chen R. Z. Pettersson U. Beard C. Jackson-Grusby L. Jaenisch R. (1998). DNA hypomethylation leads to elevated mutation rates. Nature 395, 89–93. 10.1038/25779 9738504

[B14] Cheng Y. Vechtova P. Fussy Z. Sterba J. Linhartová Z. Rodina M. (2021). Changes in phenotypes and DNA methylation of *in vitro* aging sperm in common carp *Cyprinus carpio* . Int. J. Mol. Sci. 22, 5925. 10.3390/ijms22115925 34073009 PMC8198300

[B15] Clearinghouse T. (2022). Aquaculture technology toolkit catalogue. TAAT clearinghouse. Clearinghouse technical report series 012. Gates Open Res. 6, 104. 10.21955/gatesopenres.1116904.1

[B16] Crouse M. S. Caton J. S. Claycombe-Larson K. J. Diniz W. J. Lindholm-Perry A. K. Reynolds L. P. (2022). Epigenetic modifier supplementation improves mitochondrial respiration and growth rates and alters DNA methylation of bovine embryonic fibroblast cells cultured in divergent energy supply. Front. Genet. 13, 812764. 10.3389/fgene.2022.812764 35281844 PMC8907857

[B17] Cui Z. Liu Y. Wang W. Wang Q. Zhang N. Lin F. (2017). Genome editing reveals dmrt1 as an essential Male sex-determining gene in Chinese tongue sole (*Cynoglossus semilaevis*). Sci. Rep. 7, 42213. 10.1038/srep42213 28205594 PMC5311979

[B18] Dan N. C. Little D. C. (2000). The culture performance of monosex and mixed-sex new-season and overwintered fry in three strains of nile tilapia (*Oreochromis niloticus*) in northern Vietnam. Aquaculture 184, 221–231. 10.1016/s0044-8486(99)00329-4

[B19] Dautant A. Meier T. Hahn A. Tribouillard-Tanvier D. Di Rago J.-P. Kucharczyk R. (2018). ATP synthase diseases of mitochondrial genetic origin. Front. physiology 9, 329. 10.3389/fphys.2018.00329 PMC5893901 29670542

[B20] Devall M. Soanes D. M. Smith A. R. Dempster E. L. Smith R. G. Burrage J. (2023). Genome-wide characterization of mitochondrial DNA methylation in human brain. Front. Endocrinol. 13, 1059120. 10.3389/fendo.2022.1059120 PMC9885148 36726473

[B21] De Verdal H. Mekkawy W. Lind C. E. Vandeputte M. Chatain B. Benzie J. A. (2017). Measuring individual feed efficiency and its correlations with performance traits in nile tilapia, *Oreochromis niloticus* . Aquaculture 468, 489–495. 10.1016/j.aquaculture.2016.11.015

[B22] Donaldson E. M. (1994). “Biotechnology in aquaculture,” in US congress, office of technology assessment.

[B23] Dou X. Boyd-Kirkup J. D. McDermott J. Zhang X. Li F. Rong B. (2019). The strand-biased mitochondrial DNA methylome and its regulation by DNMT3A. Genome Res. 29, 1622–1634. 10.1101/gr.234021.117 31537639 PMC6771398

[B24] Dunn J. Grider M. H. (2020). Physiology, adenosine triphosphate. 31985968

[B25] Ehrlich M. Lacey M. (2013). DNA methylation and differentiation: silencing, upregulation and modulation of gene expression. Epigenomics 5, 553–568. 10.2217/epi.13.43 24059801 PMC3864898

[B26] El-Hattab A. W. Scaglia F. (2016). Mitochondrial cytopathies. Cell Calcium 60, 199–206. 10.1016/j.ceca.2016.03.003 26996063

[B27] Eya J. C. Ashame M. F. Pomeroy C. F. (2011). Association of mitochondrial function with feed efficiency in rainbow trout: diets and family effects. Aquaculture 321, 71–84. 10.1016/j.aquaculture.2011.08.037

[B28] Eya J. C. Ukwuaba V. O. Yossa R. Gannam A. L. (2015). Interactive effects of dietary lipid and phenotypic feed efficiency on the expression of nuclear and mitochondrial genes involved in the mitochondrial electron transport chain in rainbow trout. Int. J. Mol. Sci. 16, 7682–7706. 10.3390/ijms16047682 25853266 PMC4425043

[B29] Gao D. Zhu B. Sun H. Wang X. (2017). Mitochondrial DNA methylation and related disease. Mitochondrial DNA Dis. 1038, 117–132. 10.1007/978-981-10-6674-0_9 29178073

[B30] Gardner S. T. Bertucci E. M. Sutton R. Horcher A. Aubrey D. Parrott B. B. (2023). Development of DNA methylation‐based epigenetic age predictors in loblolly pine (*Pinus taeda*). Mol. Ecol. Resour. 23, 131–144. 10.1111/1755-0998.13698 35957540 PMC10087248

[B31] Gouil Q. Keniry A. (2019). Latest techniques to study DNA methylation. Essays Biochem. 63, 639–648. 10.1042/EBC20190027 31755932 PMC6923321

[B32] Guitton R. Nido G. S. Tzoulis C. (2022). No evidence of extensive non-CpG methylation in mtDNA. Nucleic Acids Res. 50, 9190–9194. 10.1093/nar/gkac701 35979955 PMC9458446

[B33] Guppy J. L. Jones D. B. Jerry D. R. Wade N. M. Raadsma H. W. Huerlimann R. (2018). The state of “omics” research for farmed penaeids: advances in research and impediments to industry utilization. Front. Genet. 9, 282. 10.3389/fgene.2018.00282 30123237 PMC6085479

[B34] Haas R. H. Parikh S. Falk M. J. Saneto R. P. Wolf N. I. Darin N. (2007). Mitochondrial disease: a practical approach for primary care physicians. Pediatrics 120, 1326–1333. 10.1542/peds.2007-0391 18055683

[B35] Haque E. Irfan S. Kamil M. Sheikh S. Hasan A. Ahmad A. (2016). Terpenoids with antifungal activity trigger mitochondrial dysfunction in *Saccharomyces cerevisiae* . Microbiology 85, 436–443. 10.1134/s0026261716040093 28853775

[B36] Houston R. D. Bean T. P. Macqueen D. J. Gundappa M. K. Jin Y. H. Jenkins T. L. (2020). Harnessing genomics to fast-track genetic improvement in aquaculture. Nat. Rev. Genet. 21, 389–409. 10.1038/s41576-020-0227-y 32300217

[B37] Jarmuszkiewicz W. Woyda-Ploszczyca A. Koziel A. Majerczak J. Zoladz J. A. (2015). Temperature controls oxidative phosphorylation and reactive oxygen species production through uncoupling in rat skeletal muscle mitochondria. Free Radic. Biol. Med. 83, 12–20. 10.1016/j.freeradbiomed.2015.02.012 25701433

[B38] Ji J. Yan G. Chen D. Xiao S. Gao J. Zhang Z. (2019). An association study using imputed whole‐genome sequence data identifies novel significant loci for growth‐related traits in a duroc× erhualian F2 population. J. Animal Breed. Genet. 136, 217–228. 10.1111/jbg.12389 30869175

[B39] Jia Y. Song H. Gao G. Cai D. Yang X. Zhao R. (2015). Maternal betaine supplementation during gestation enhances expression of mtDNA-encoded genes through D-loop DNA hypomethylation in the skeletal muscle of newborn piglets. J. Agric. Food Chem. 63, 10152–10160. 10.1021/acs.jafc.5b04418 26527363

[B40] Kehrein K. Bonnefoy N. Ott M. (2013). Mitochondrial protein synthesis: efficiency and accuracy. Antioxidants and Redox Signal. 19, 1928–1939. 10.1089/ars.2012.4896 23088322

[B41] Kikuchi A. Onoda H. Yamaguchi K. Kori S. Matsuzawa S. Chiba Y. (2022). Structural basis for activation of DNMT1. Nat. Commun. 13, 7130. 10.1038/s41467-022-34779-4 36414620 PMC9681727

[B42] Koch R. E. Buchanan K. L. Casagrande S. Crino O. Dowling D. K. Hill G. E. (2021). Integrating mitochondrial aerobic metabolism into ecology and evolution. Trends Ecol. and Evol. 36, 321–332. 10.1016/j.tree.2020.12.006 33436278

[B43] Konstantinidis I. Sætrom P. Brieuc M. S. Jakobsen K. S. Liedtke H. Pohlmann C. (2023a). DNA hydroxymethylation differences underlie phenotypic divergence of somatic growth in nile tilapia reared in common garden. Epigenetics 18, 2282323. 10.1080/15592294.2023.2282323 38010265 PMC10732659

[B44] Konstantinidis I. Sætrom P. Fernandes J. M. (2023b). Genome-wide hydroxymethylation profiles in liver of female nile tilapia with distinct growth performance. Sci. data 10, 114. 10.1038/s41597-023-01996-5 36859394 PMC9977925

[B45] Konstantinidis I. Sætrom P. Mjelle R. Nedoluzhko A. V. Robledo D. Fernandes J. M. (2020). Major gene expression changes and epigenetic remodelling in nile tilapia muscle after just one generation of domestication. Epigenetics 15, 1052–1067. 10.1080/15592294.2020.1748914 32264748 PMC7116051

[B46] Kozłowski J. Konarzewski M. Czarnoleski M. (2020). Coevolution of body size and metabolic rate in vertebrates: a life‐history perspective. Biol. Rev. 95, 1393–1417. 10.1111/brv.12615 32524739 PMC7540708

[B47] Leroux E. Brosseau C. Angers B. Angers A. Breton S. (2021). Mitochondrial DNA methylation: controversies, issues and perspectives. Med. Sci. M/S 37, 258–264. 10.1051/medsci/2021011 33739273

[B48] Li Y. He M. (2014). Genetic mapping and QTL analysis of growth-related traits in Pinctada fucata using restriction-site associated DNA sequencing. PLoS One 9, e111707. 10.1371/journal.pone.0111707 25369421 PMC4219768

[B49] Liao K. Yan J. Mai K. Ai Q. (2015). Dietary olive and perilla oils affect liver mitochondrial DNA methylation in large yellow croakers. J. Nutr. 145, 2479–2485. 10.3945/jn.115.216481 26400965

[B50] Liao K. Yan J. Mai K. Ai Q. (2016). Dietary lipid concentration affects liver mitochondrial DNA copy number, gene expression and DNA methylation in large yellow croaker (*Larimichthys crocea*). Comp. Biochem. Physiology Part B Biochem. Mol. Biol. 193, 25–32. 10.1016/j.cbpb.2015.11.012 26692128

[B51] Lind C. Safari A. Agyakwah S. Attipoe F. El-Naggar G. Hamzah A. (2015). Differences in sexual size dimorphism among farmed tilapia species and strains undergoing genetic improvement for body weight. Aquac. Rep. 1, 20–27. 10.1016/j.aqrep.2015.03.003

[B52] Liu B. Du Q. Chen L. Fu G. Li S. Fu L. (2016). CpG methylation patterns of human mitochondrial DNA. Sci. Rep. 6, 23421. 10.1038/srep23421 26996456 PMC4800444

[B53] Liu Z. Zhou T. Gao D. (2022). Genetic and epigenetic regulation of growth, reproduction, disease resistance and stress responses in aquaculture. Front. Genet. 13, 994471. 10.3389/fgene.2022.994471 36406125 PMC9666392

[B54] Lomba A. Milagro F. I. García-Díaz D. F. Marti A. Campión J. Martínez J. A. (2010). Obesity induced by a pair-fed high fat sucrose diet: methylation and expression pattern of genes related to energy homeostasis. Lipids Health Dis. 9, 1–10. 10.1186/1476-511X-9-60 20534152 PMC2909242

[B55] Lopes A. F. (2020). Mitochondrial metabolism and DNA methylation: a review of the interaction between two genomes. Clin. Epigenetics 12, 182. 10.1186/s13148-020-00976-5 33228792 PMC7684747

[B56] Low R. L. Orton S. Friedman D. B. (2003). A truncated form of DNA topoisomerase IIbeta associates with the mtDNA genome in Mammalian mitochondria. Eur. J. Biochem. 270, 4173–4186. 10.1046/j.1432-1033.2003.03814.x 14519130

[B57] Lozoya O. A. Martinez-Reyes I. Wang T. Grenet D. Bushel P. Li J. (2018). Mitochondrial nicotinamide adenine dinucleotide reduced (NADH) oxidation links the tricarboxylic acid (TCA) cycle with methionine metabolism and nuclear DNA methylation. PLoS Biol. 16, e2005707. 10.1371/journal.pbio.2005707 29668680 PMC5927466

[B58] Maes V. Betoulle S. Jaffal A. Dedourge-Geffard O. Delahaut L. Geffard A. (2016). Juvenile roach (*rutilus Rutilus*) increase their anaerobic metabolism in response to copper exposure in laboratory conditions. Ecotoxicology 25, 900–913. 10.1007/s10646-016-1648-4 27033855

[B59] Mamun S. M. Focken U. Becker K. (2007). Comparison of metabolic rates and feed nutrient digestibility in conventional, genetically improved (GIFT) and genetically Male (GMNT) nile tilapia, *Oreochromis niloticus* (L.). Comp. Biochem. Physiology Part A Mol. and Integr. Physiology 148, 214–222. 10.1016/j.cbpa.2007.04.007 17555997

[B60] Marcos‐López M. Gale P. Oidtmann B. Peeler E. (2010). Assessing the impact of climate change on disease emergence in freshwater fish in the United Kingdom. Transbound. Emerg. Dis. 57, 293–304. 10.1111/j.1865-1682.2010.01150.x 20561287

[B61] Mark F. C. Lucassen M. Strobel A. Barrera-Oro E. Koschnick N. Zane L. (2012). Mitochondrial function in antarctic nototheniids with ND6 translocation. PloS one 7, e31860. 10.1371/journal.pone.0031860 22363756 PMC3283701

[B62] Mehrim A. Khalil F. Hassan M. (2019). Sexual maturity signs and histological alterations of adult *Oreochromis niloticus* (linnaeus, 1758) fed probiotic. Int. J. Anat. Appl. Physiol. 5, 103–110. 10.19070/2572-7451-1900019

[B63] Mieiro C. Pardal M. Duarte A. Pereira E. Palmeira C. (2015). Impairment of mitochondrial energy metabolism of two marine fish by *in vitro* mercuric chloride exposure. Mar. Pollut. Bull. 97, 488–493. 10.1016/j.marpolbul.2015.05.054 26026249

[B64] Minocherhomji S. Tollefsbol T. O. Singh K. K. (2012). Mitochondrial regulation of epigenetics and its role in human diseases. Epigenetics 7, 326–334. 10.4161/epi.19547 22419065 PMC3368816

[B65] Mposhi A. Van der Wijst M. G. Faber K. N. Rots M. G. (2017). Regulation of mitochondrial gene expression, the epigenetic enigma. Front. Biosci. (Landmark Ed) 22, 1099–1113. 10.2741/4535 28199194

[B66] Nagy K. A. (2005). Field metabolic rate and body size. J. Exp. Biol. 208, 1621–1625. 10.1242/jeb.01553 15855393

[B67] Naylor R. L. Hardy R. W. Buschmann A. H. Bush S. R. Cao L. Klinger D. H. (2021). A 20-year retrospective review of global aquaculture. Nature 591, 551–563. 10.1038/s41586-021-03308-6 33762770

[B68] Nedoluzhko A. Mjelle R. Renström M. Skjærven K. H. Piferrer F. Fernandes J. M. (2021). The first mitochondrial 5-methylcytosine map in a non-model teleost (*Oreochromis niloticus*) reveals extensive strand-specific and non-CpG methylation. Genomics 113, 3050–3057. 10.1016/j.ygeno.2021.07.007 34245830

[B69] Podgorniak T. Dhanasiri A. Chen X. Ren X. Kuan P.-F. Fernandes J. (2022). Early fish domestication affects methylation of key genes involved in the rapid onset of the farmed phenotype. Epigenetics 17, 1281–1298. 10.1080/15592294.2021.2017554 35006036 PMC9542679

[B70] Rasal K. D. Sundaray J. K. (2020). Status of genetic and genomic approaches for delineating biological information and improving aquaculture production of farmed rohu, *labeo Rohita* (ham, 1822). Rev. Aquac. 12, 2466–2480. 10.1111/raq.12444

[B71] Salin K. Villasevil E. M. Anderson G. J. Lamarre S. G. Melanson C. A. McCarthy I. (2019). Differences in mitochondrial efficiency explain individual variation in growth performance. Proc. R. Soc. B 286, 20191466. 10.1098/rspb.2019.1466 PMC6732382 31431161

[B72] Schreiber S. Focken U. Becker K. (1998). Individually reared female nile tilapia (*oreochrornis Niloticus*) can grow faster than males. J. Appl. Ichthyology 14, 43–47. 10.1111/j.1439-0426.1998.tb00612.x

[B73] Sciara A. Rodríguez-Ramilo S. T. Hermida M. Gómez-Tato A. Fernández J. Bouza C. (2018). Validation of growth-related quantitative trait loci markers in turbot (*Scophthalmus maximus*) families as a step toward marker assisted selection. Aquaculture 495, 602–610. 10.1016/j.aquaculture.2018.06.010

[B74] Sharma N. Pasala M. S. Prakash A. (2019). Mitochondrial DNA: epigenetics and environment. Environ. Mol. Mutagen. 60, 668–682. 10.1002/em.22319 31335990 PMC6941438

[B75] Shock L. S. Thakkar P. V. Peterson E. J. Moran R. G. Taylor S. M. (2011). DNA methyltransferase 1, cytosine methylation, and cytosine hydroxymethylation in Mammalian mitochondria. Proc. Natl. Acad. Sci. 108, 3630–3635. 10.1073/pnas.1012311108 21321201 PMC3048134

[B76] Stimpfel M. Jancar N. Virant-Klun I. (2018). New challenge: mitochondrial epigenetics? Stem Cell Rev. Rep. 14, 13–26. 10.1007/s12015-017-9771-z 28980199

[B77] Stoccoro A. Coppedè F. (2021). Mitochondrial DNA methylation and human diseases. Int. J. Mol. Sci. 22, 4594. 10.3390/ijms22094594 33925624 PMC8123858

[B78] Toguyeni A. Fauconneau B. t. Fostier A. Abucay J. Mair G. Baroiller J.-F. (2002). Influence of sexual phenotype and genotype, and sex ratio on growth performances in tilapia, *Oreochromis niloticus* . Aquaculture 207, 249–261. 10.1016/s0044-8486(01)00747-5

[B79] Wallace D. C. Fan W. (2010). Energetics, epigenetics, mitochondrial genetics. Mitochondrion 10, 12–31. 10.1016/j.mito.2009.09.006 19796712 PMC3245717

[B80] Wang J. Su B. Dunham R. A. (2022). Genome‐wide identification of catfish antimicrobial peptides: a new perspective to enhance fish disease resistance. Rev. Aquac. 14, 2002–2022. 10.1111/raq.12684

[B81] Wang S. Jiao N. Zhao L. Zhang M. Zhou P. Huang X. (2020). Evidence for the paternal mitochondrial DNA in the crucian carp-like fish lineage with hybrid origin. Sci. China Life Sci. 63, 102–115. 10.1007/s11427-019-9528-1 31728830

[B82] Wickham H. (2011). ggplot2. Wiley Interdiscip. Rev. Comput. Stat. 3, 180–185. 10.1002/wics.147

[B83] Wilson A. S. Power B. E. Molloy P. L. (2007). DNA hypomethylation and human diseases. Biochimica Biophysica Acta (BBA)-Reviews Cancer 1775, 138–162. 10.1016/j.bbcan.2006.08.007 17045745

[B84] Ye Y. Ren W. Zhang S. Zhao L. Tang J. Hu L. (2022). Genetic diversity of fish in aquaculture and of common carp (*Cyprinus carpio*) in traditional rice–fish coculture. Agriculture 12, 997. 10.3390/agriculture12070997

