# Supplementary figures and images for: Mitochondrial methylation is linked to sexually dimorphic growth in Nile tilapia (Oreochromis niloticus) (part 1 of 3)
Source: Front Cell Dev Biol. 2025 Aug 5;13:1643817. doi: 10.3389/fcell.2025.1643817 (PMC12361127; doi:10.3389/fcell.2025.1643817)

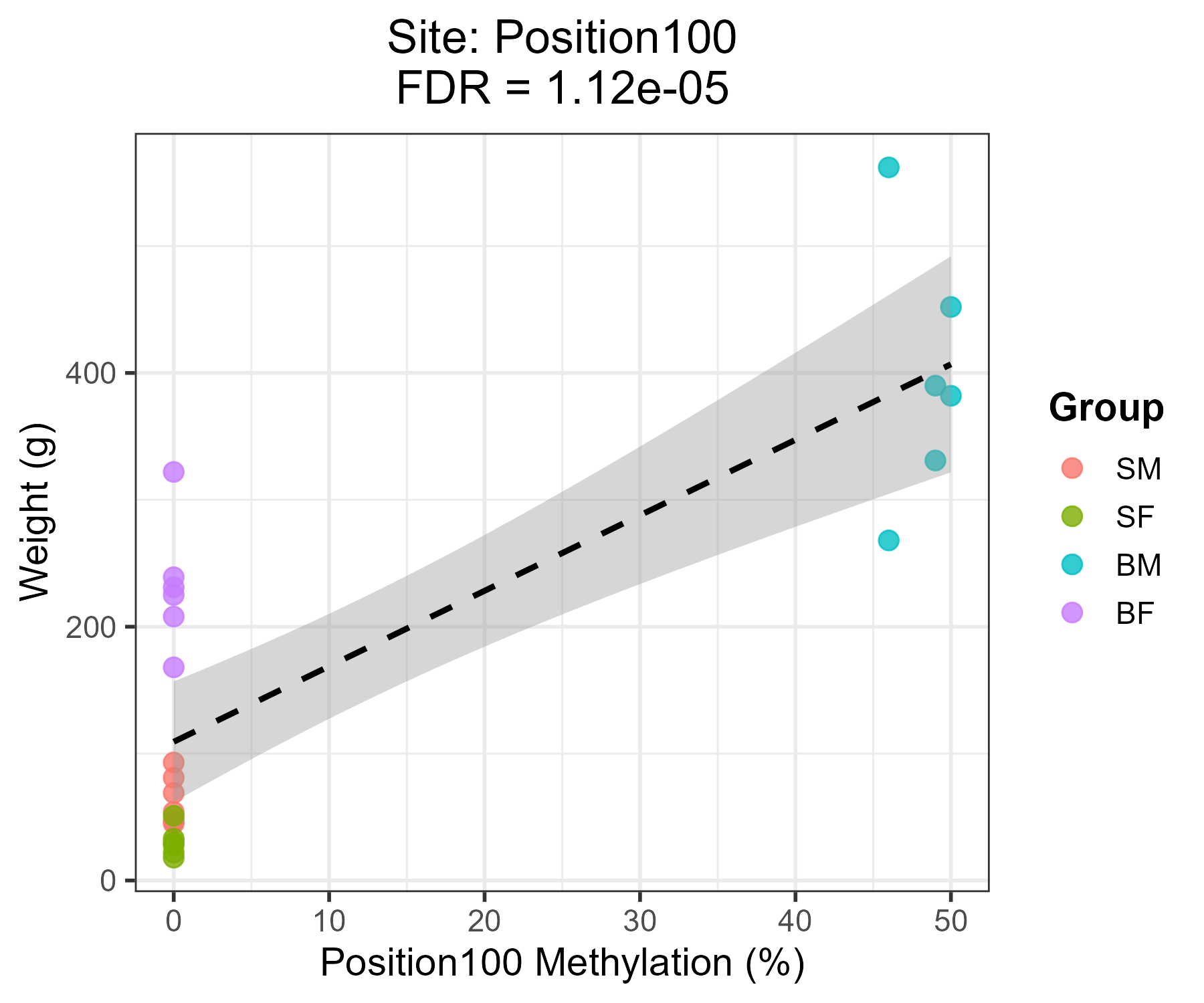

Supplement: Supplementary file 2 [file DataSheet1.zip › Regression_Plus_Strand/Position100_regression.tiff]

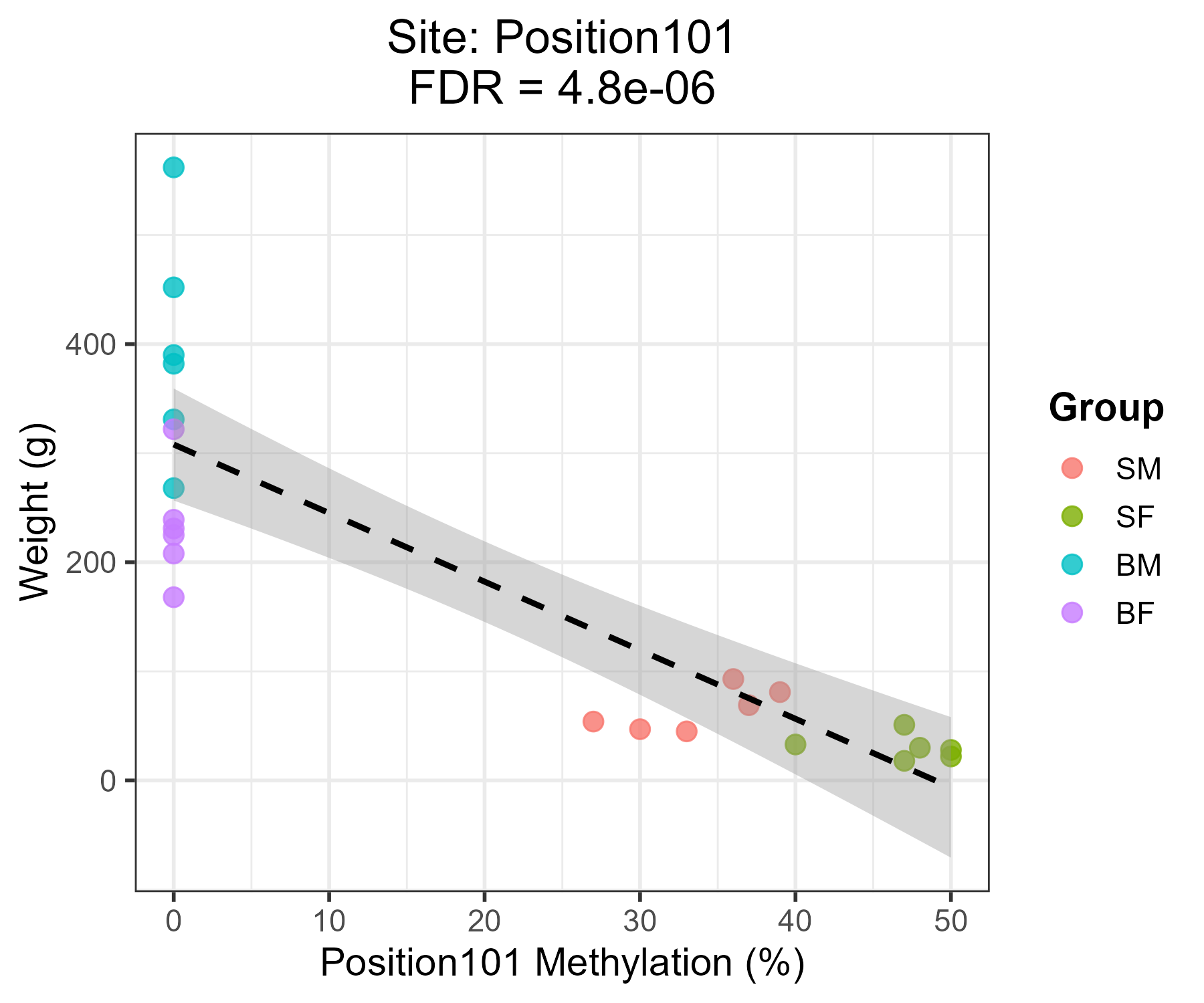

Supplement: Supplementary file 2 [file DataSheet1.zip › Regression_Plus_Strand/Position101_regression.tiff]

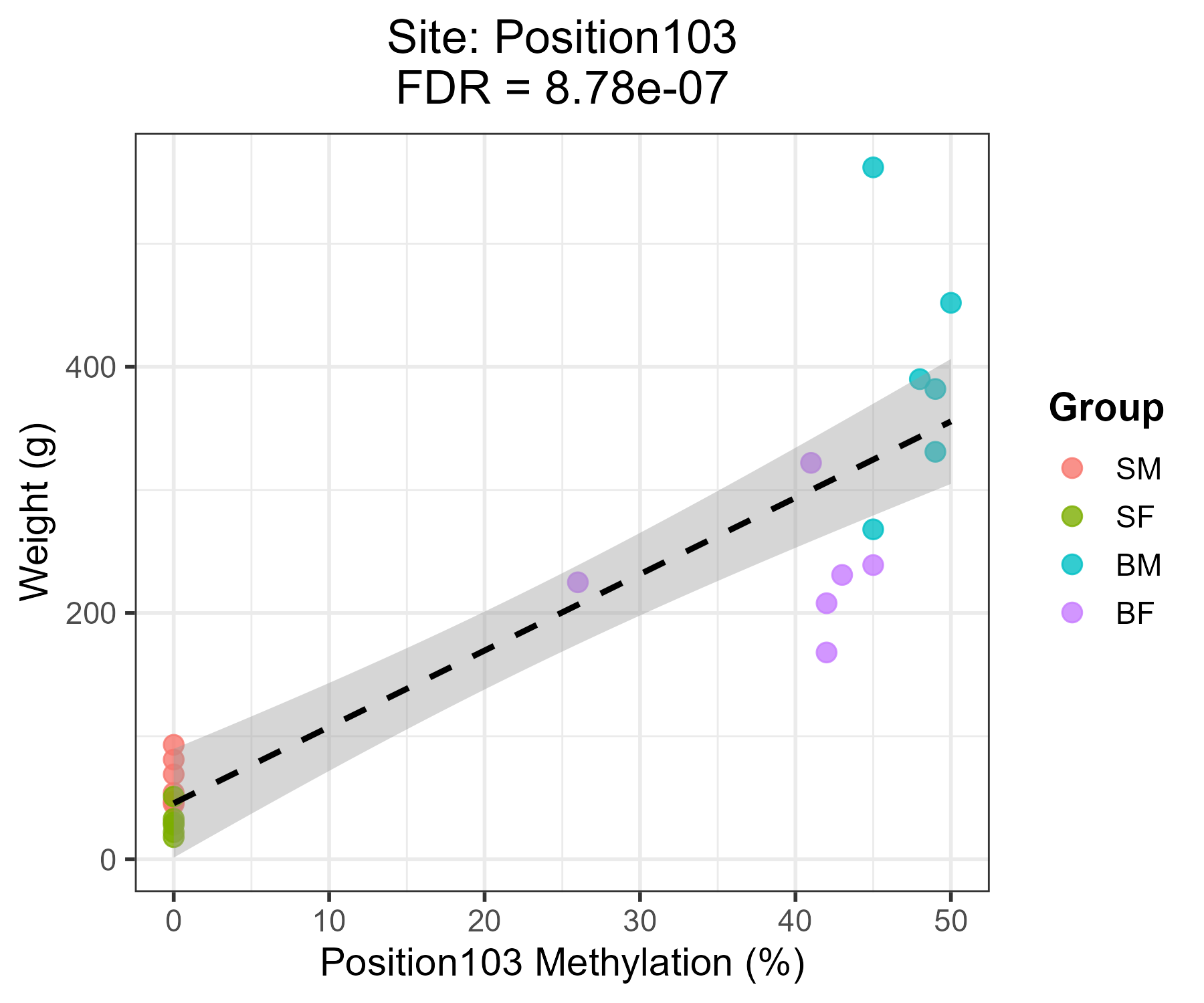

Supplement: Supplementary file 2 [file DataSheet1.zip › Regression_Plus_Strand/Position103_regression.tiff]

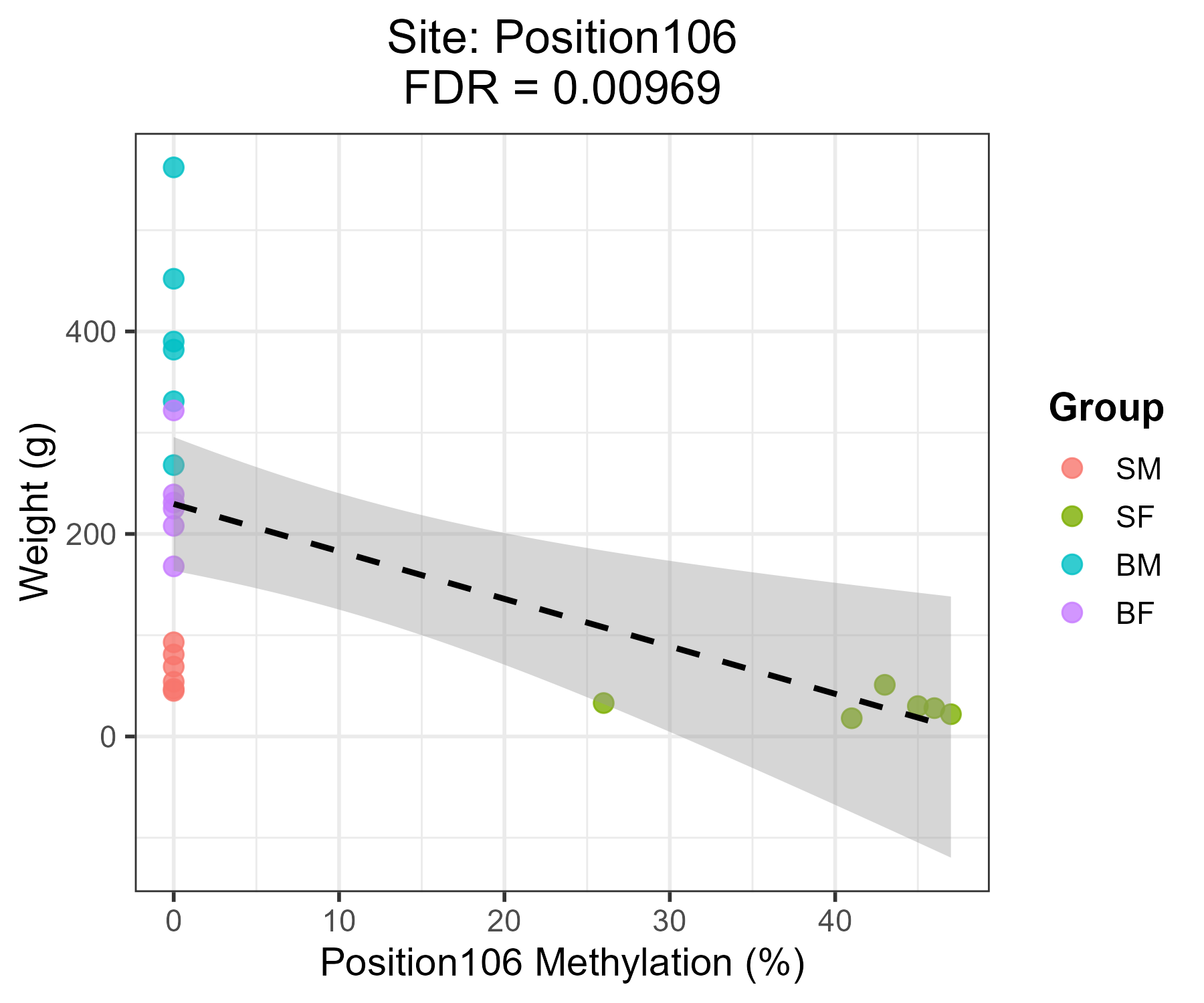

Supplement: Supplementary file 2 [file DataSheet1.zip › Regression_Plus_Strand/Position106_regression.tiff]

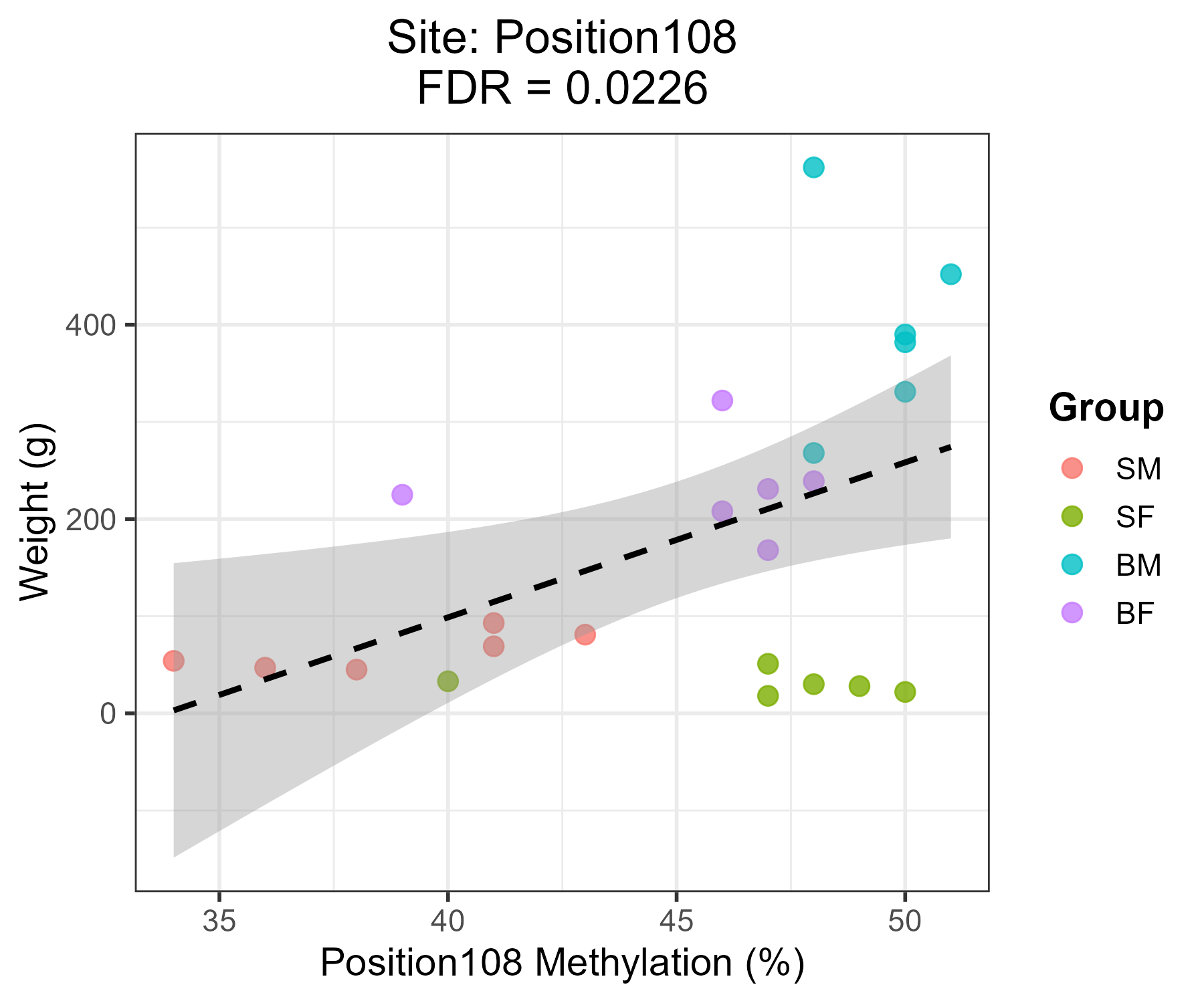

Supplement: Supplementary file 2 [file DataSheet1.zip › Regression_Plus_Strand/Position108_regression.tiff]

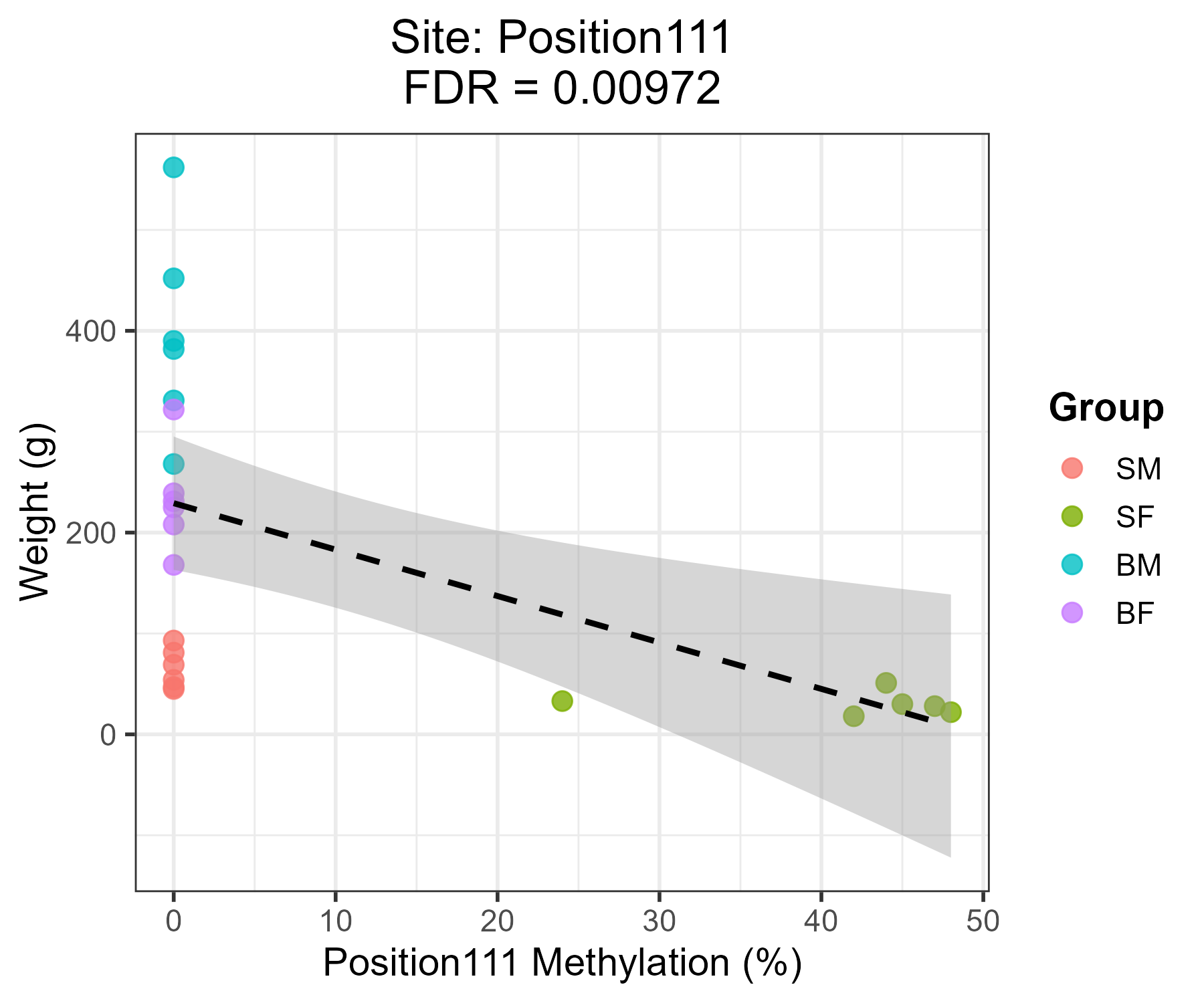

Supplement: Supplementary file 2 [file DataSheet1.zip › Regression_Plus_Strand/Position111_regression.tiff]

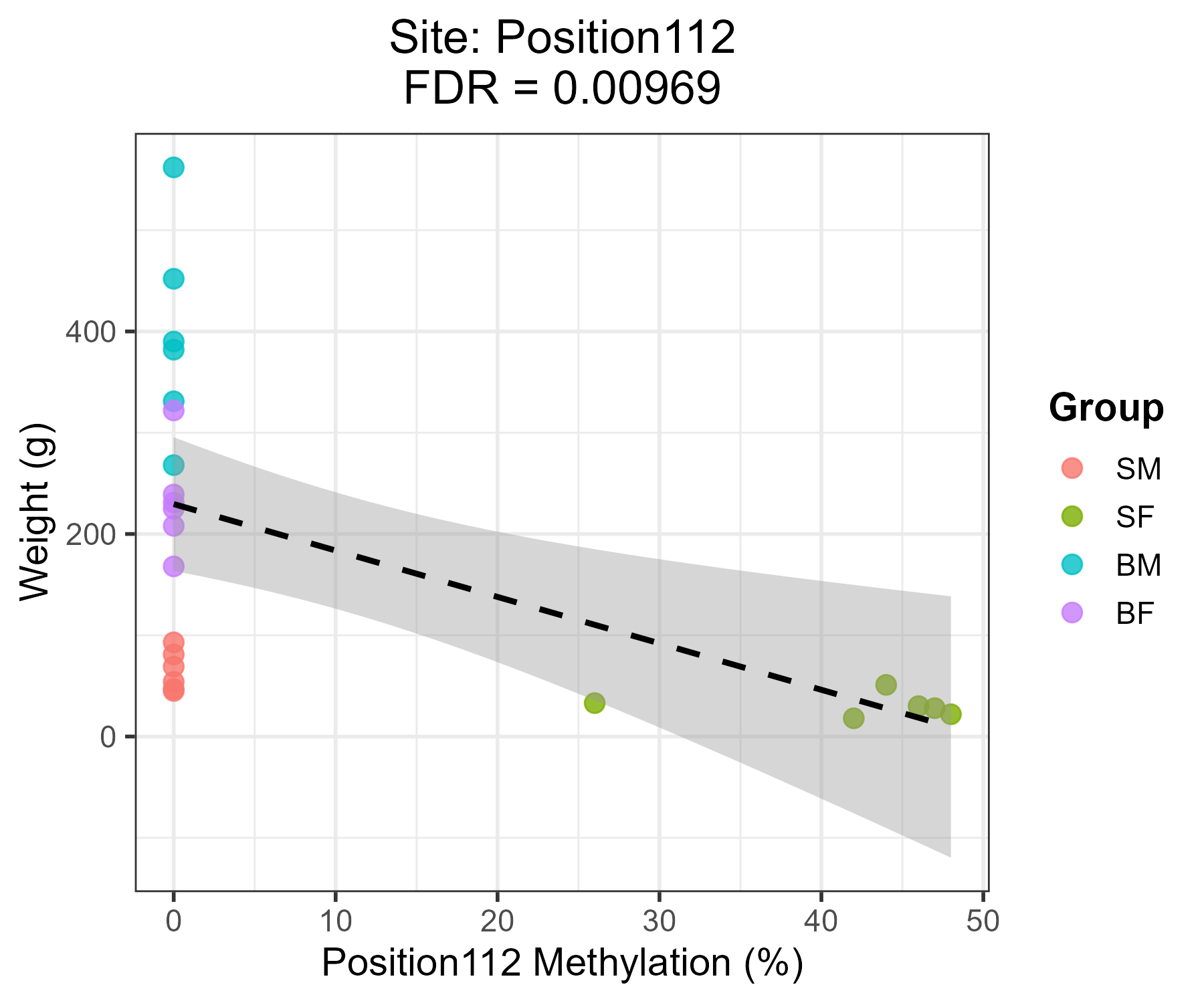

Supplement: Supplementary file 2 [file DataSheet1.zip › Regression_Plus_Strand/Position112_regression.tiff]

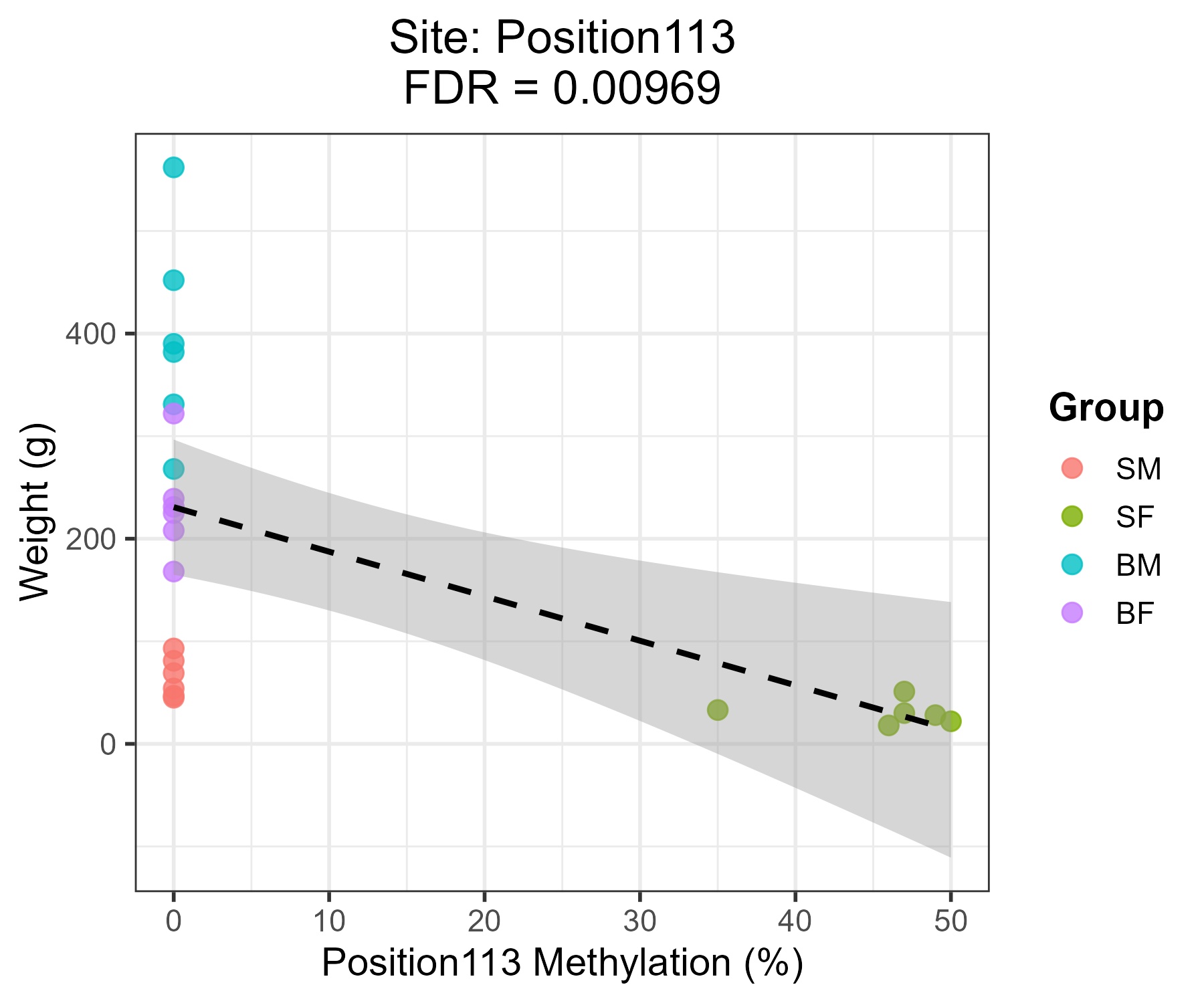

Supplement: Supplementary file 2 [file DataSheet1.zip › Regression_Plus_Strand/Position113_regression.tiff]

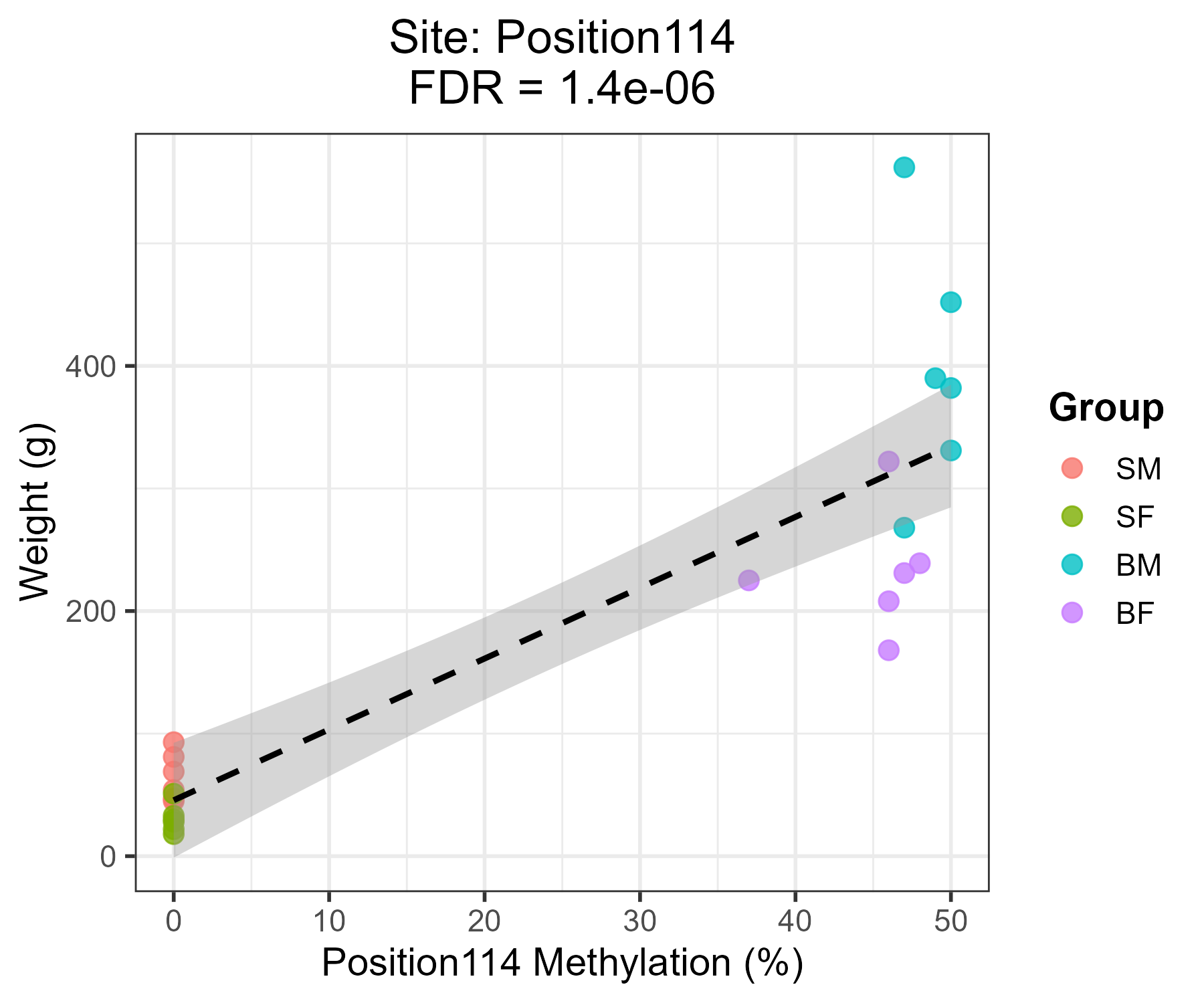

Supplement: Supplementary file 2 [file DataSheet1.zip › Regression_Plus_Strand/Position114_regression.tiff]

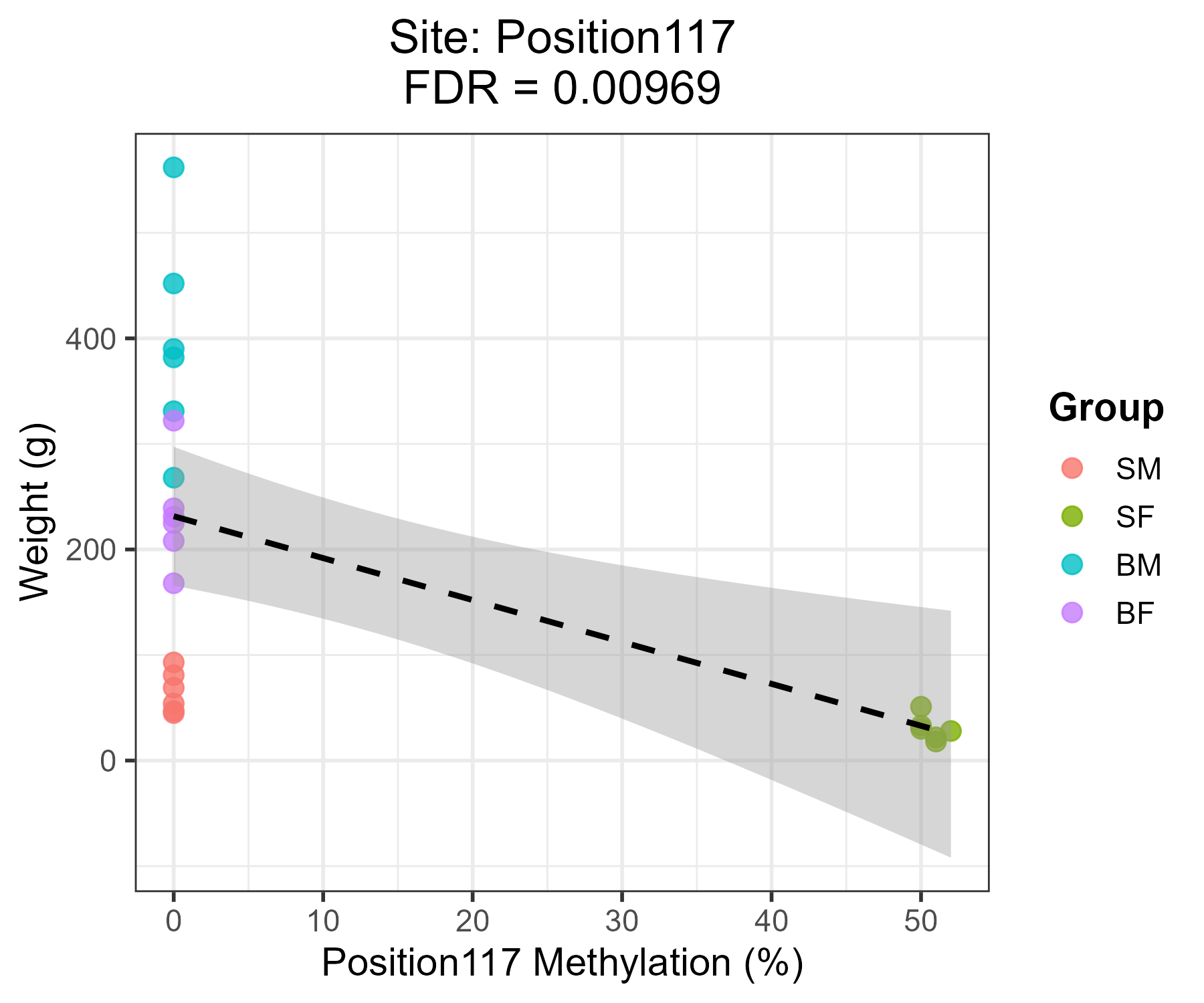

Supplement: Supplementary file 2 [file DataSheet1.zip › Regression_Plus_Strand/Position117_regression.tiff]

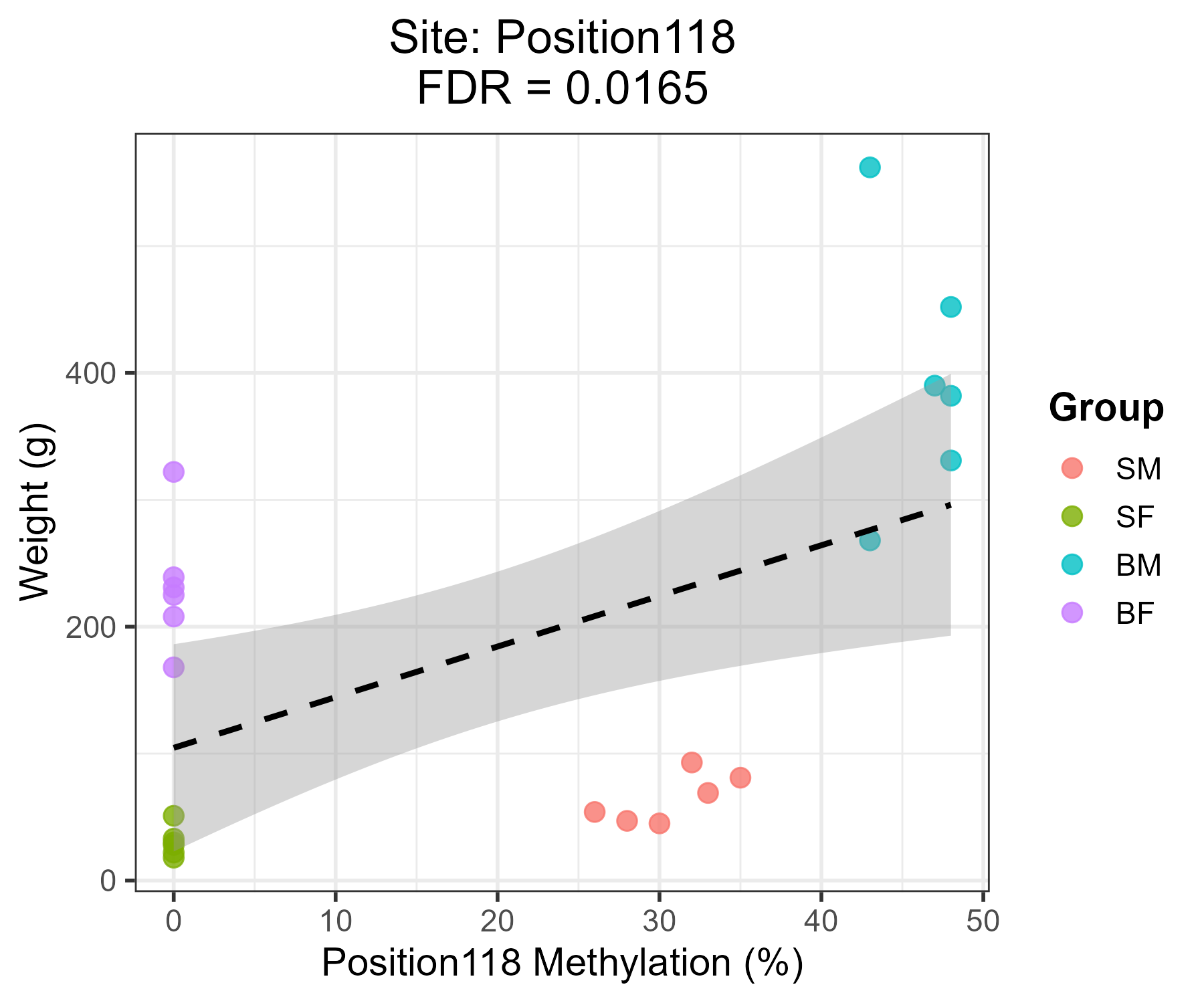

Supplement: Supplementary file 2 [file DataSheet1.zip › Regression_Plus_Strand/Position118_regression.tiff]

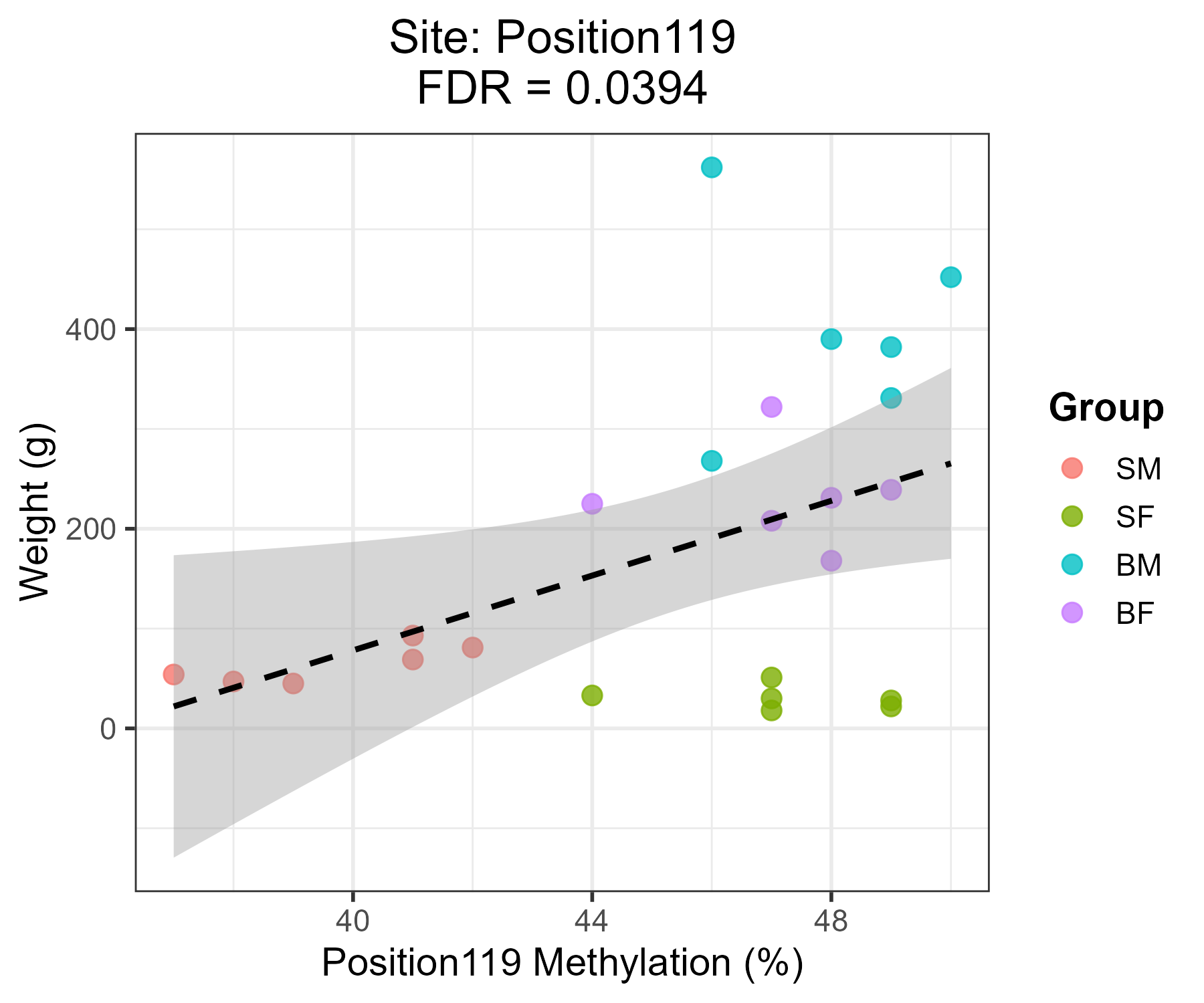

Supplement: Supplementary file 2 [file DataSheet1.zip › Regression_Plus_Strand/Position119_regression.tiff]

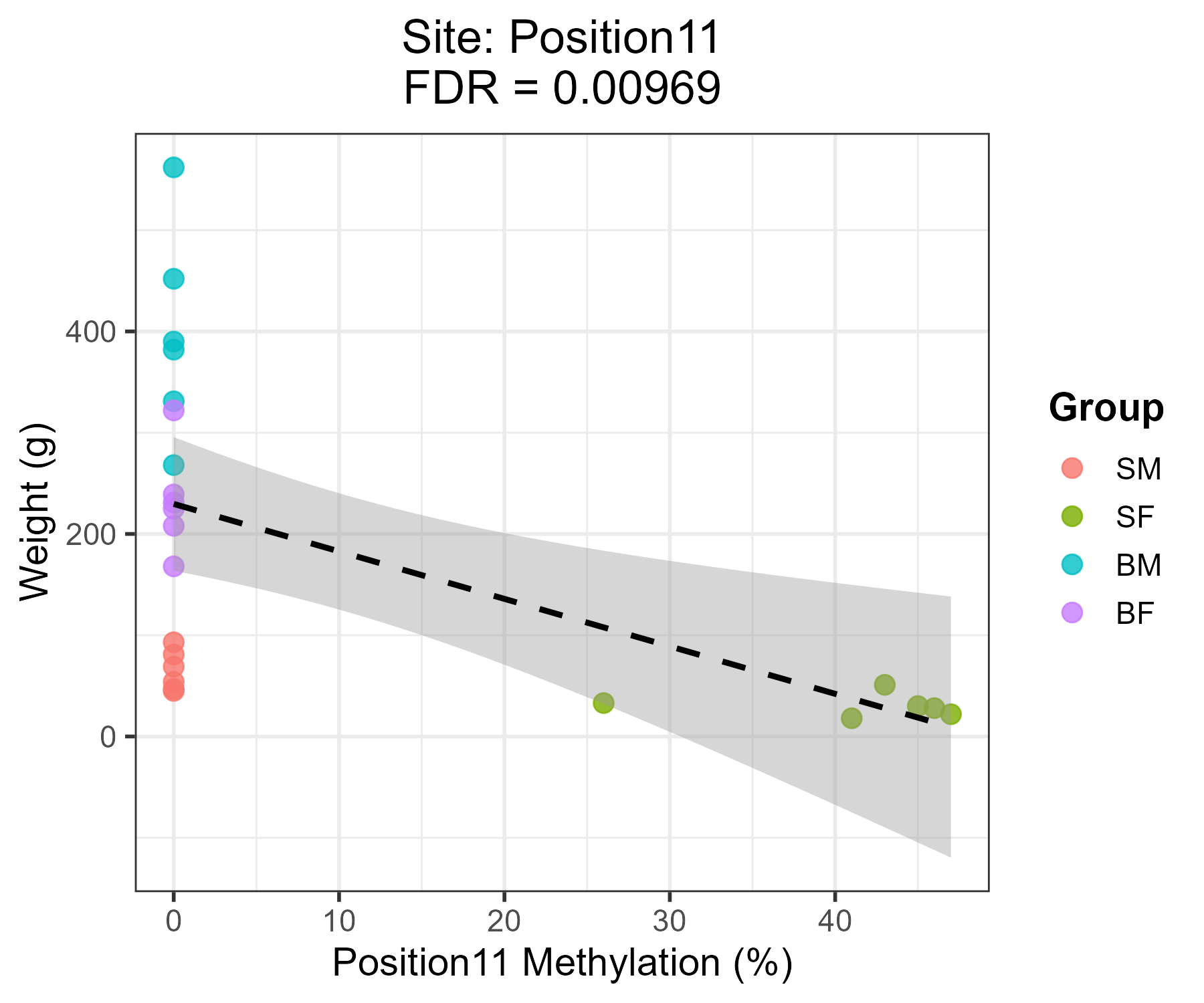

Supplement: Supplementary file 2 [file DataSheet1.zip › Regression_Plus_Strand/Position11_regression.tiff]

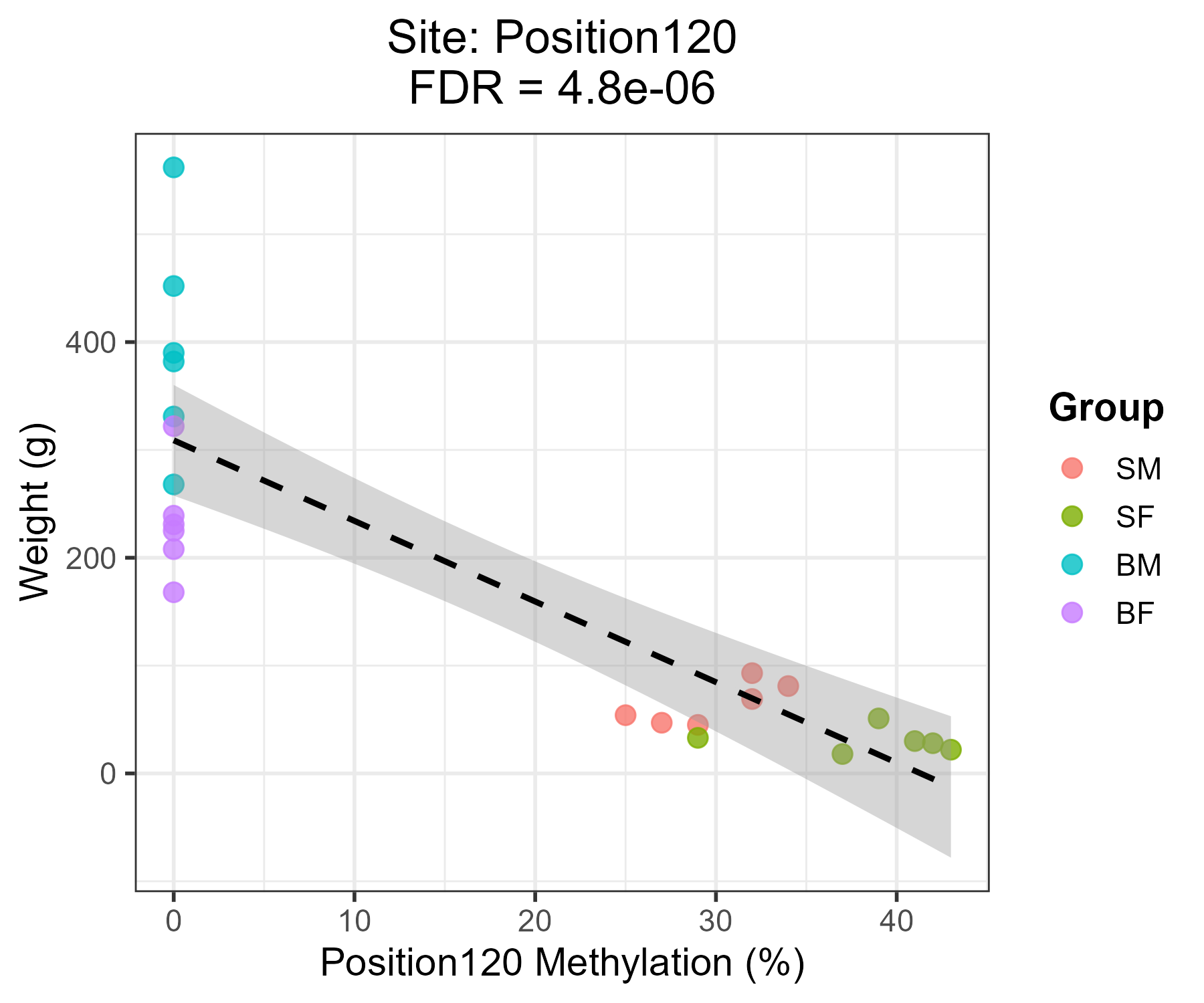

Supplement: Supplementary file 2 [file DataSheet1.zip › Regression_Plus_Strand/Position120_regression.tiff]

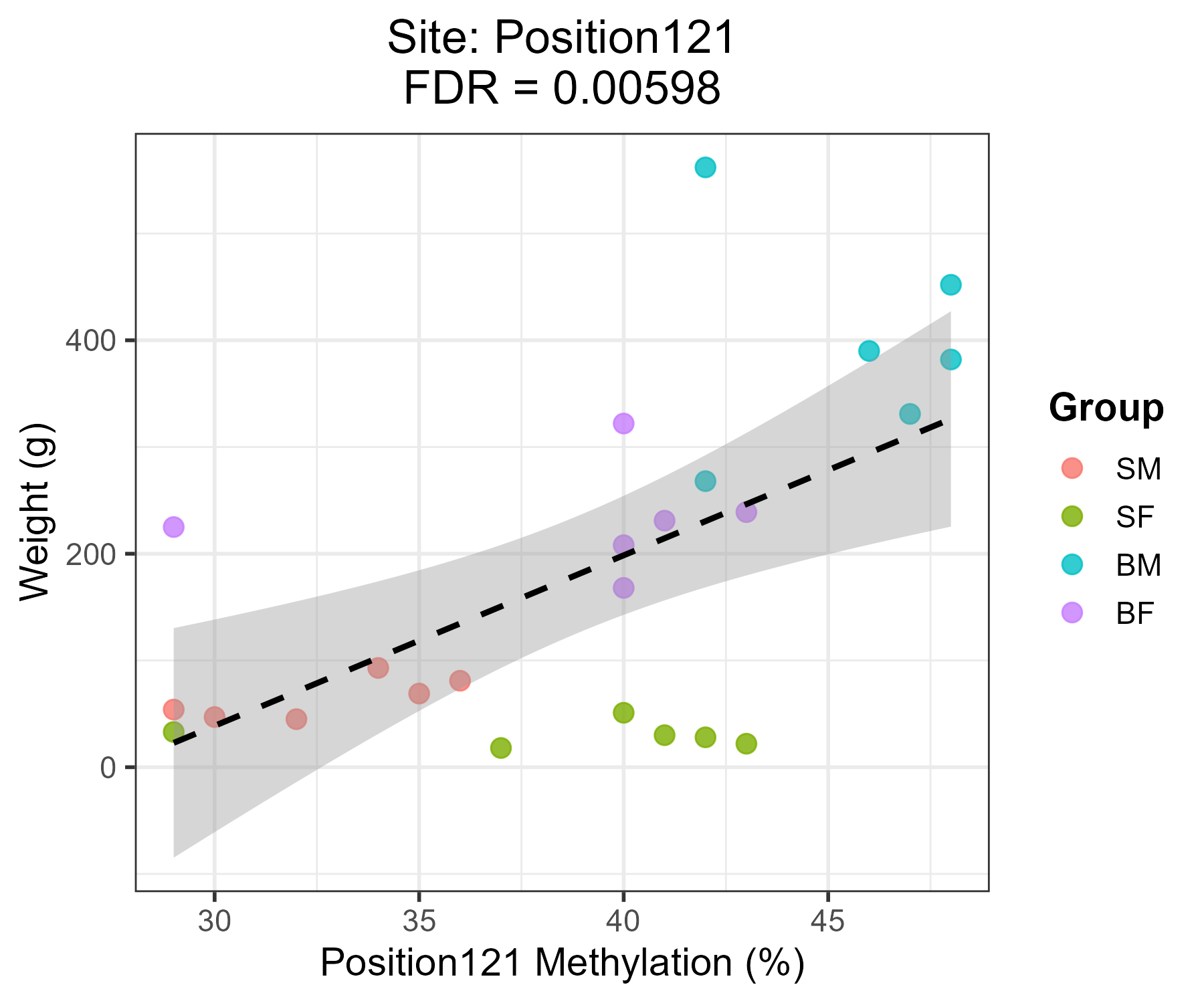

Supplement: Supplementary file 2 [file DataSheet1.zip › Regression_Plus_Strand/Position121_regression.tiff]

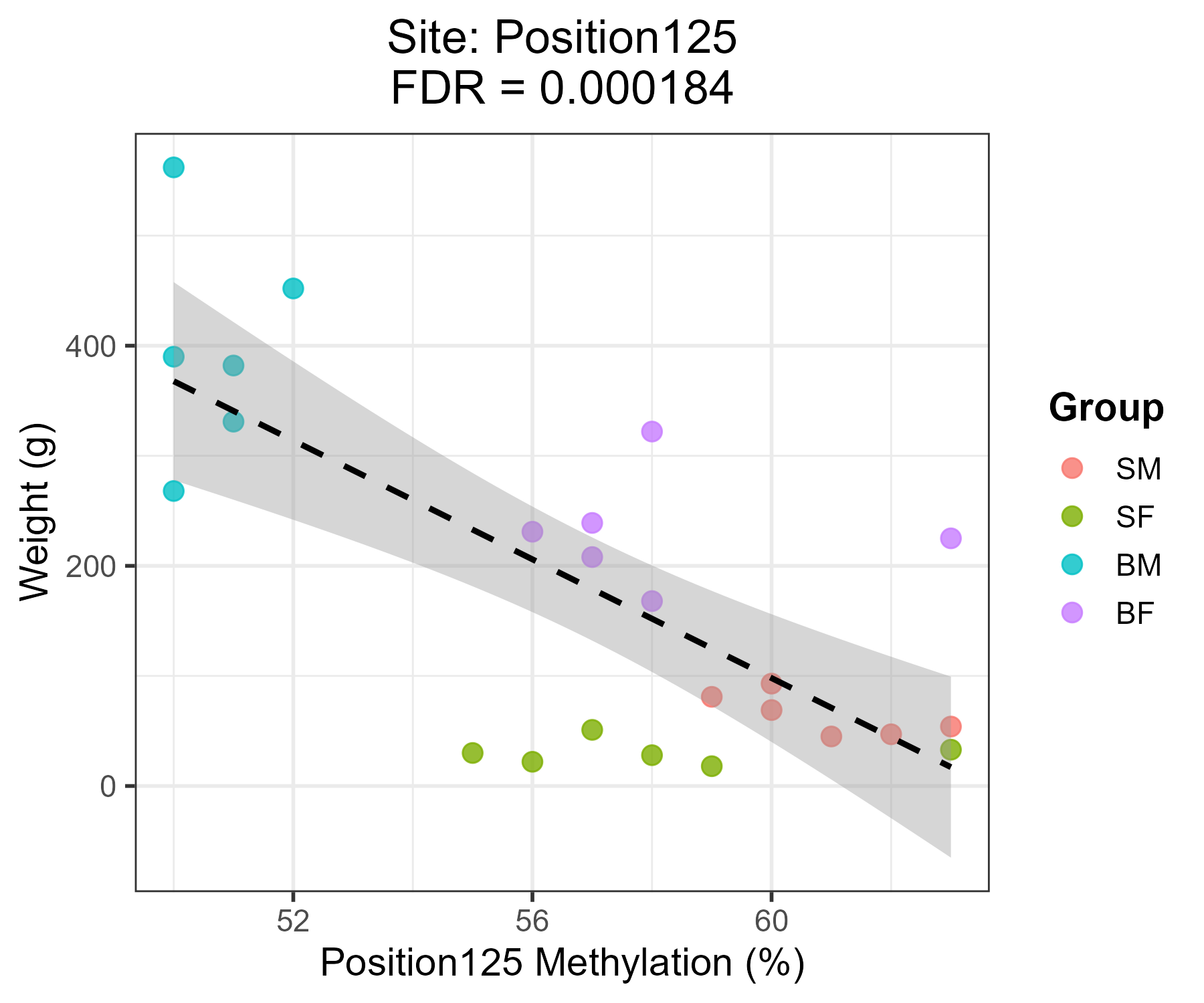

Supplement: Supplementary file 2 [file DataSheet1.zip › Regression_Plus_Strand/Position125_regression.tiff]

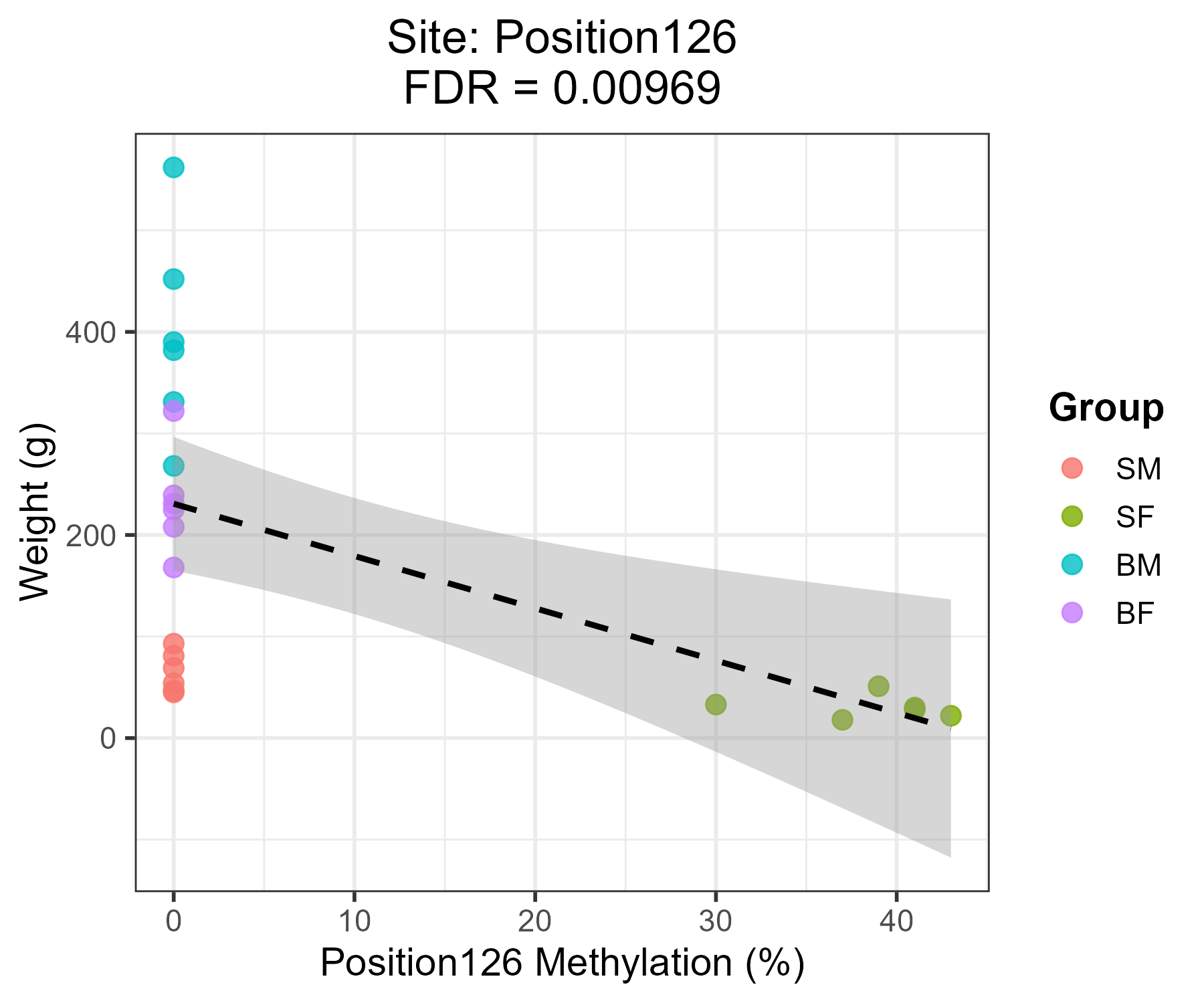

Supplement: Supplementary file 2 [file DataSheet1.zip › Regression_Plus_Strand/Position126_regression.tiff]

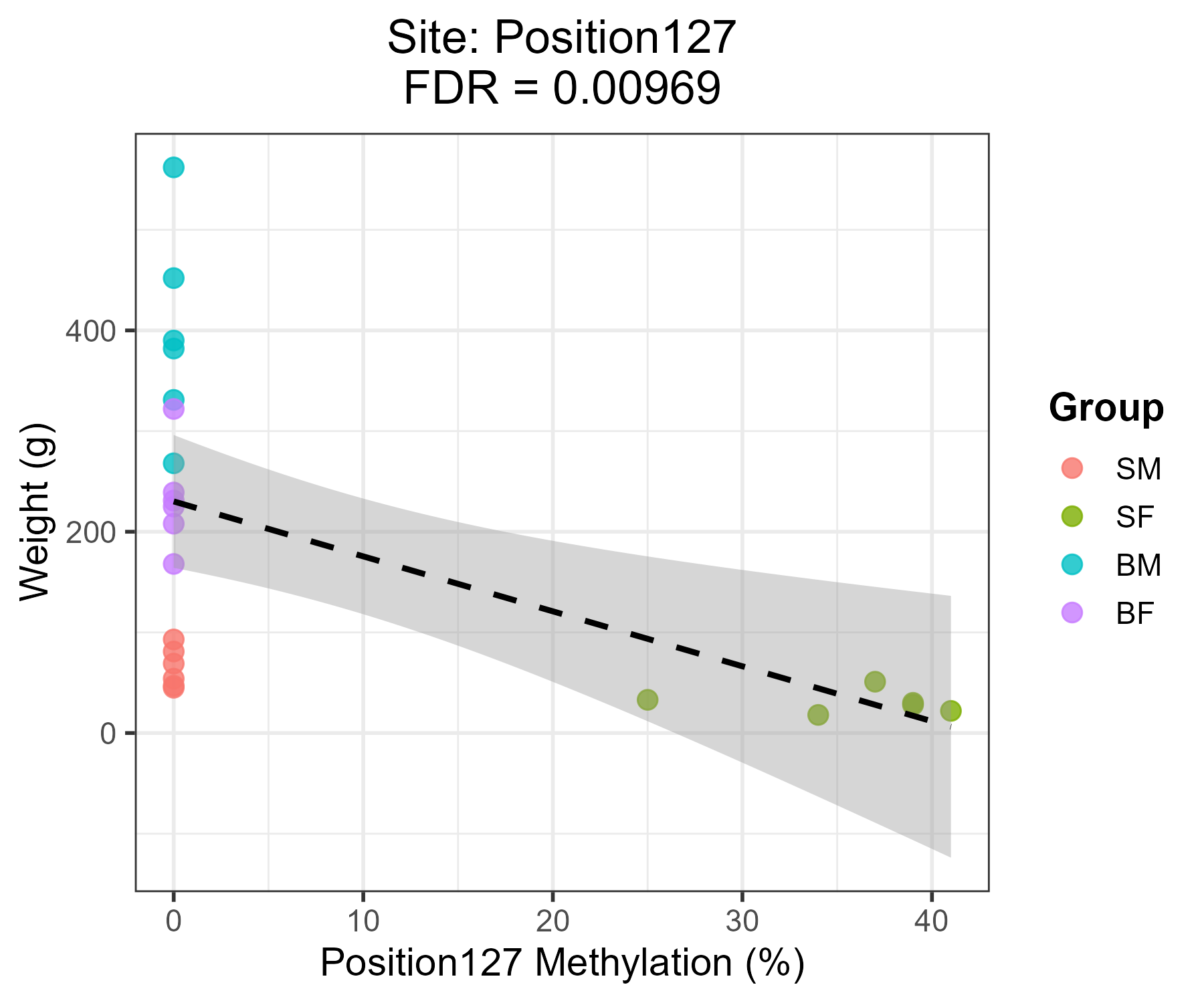

Supplement: Supplementary file 2 [file DataSheet1.zip › Regression_Plus_Strand/Position127_regression.tiff]

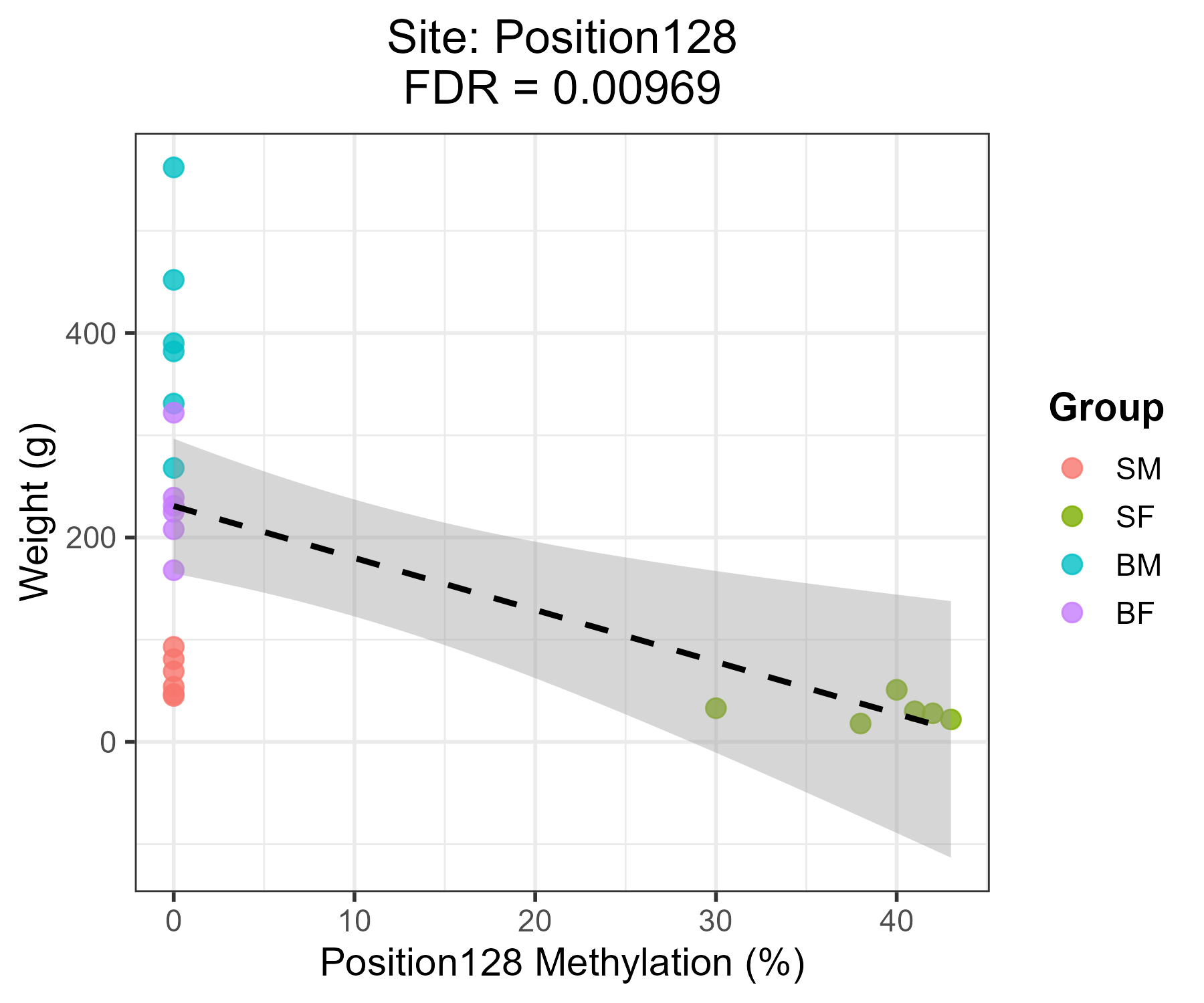

Supplement: Supplementary file 2 [file DataSheet1.zip › Regression_Plus_Strand/Position128_regression.tiff]

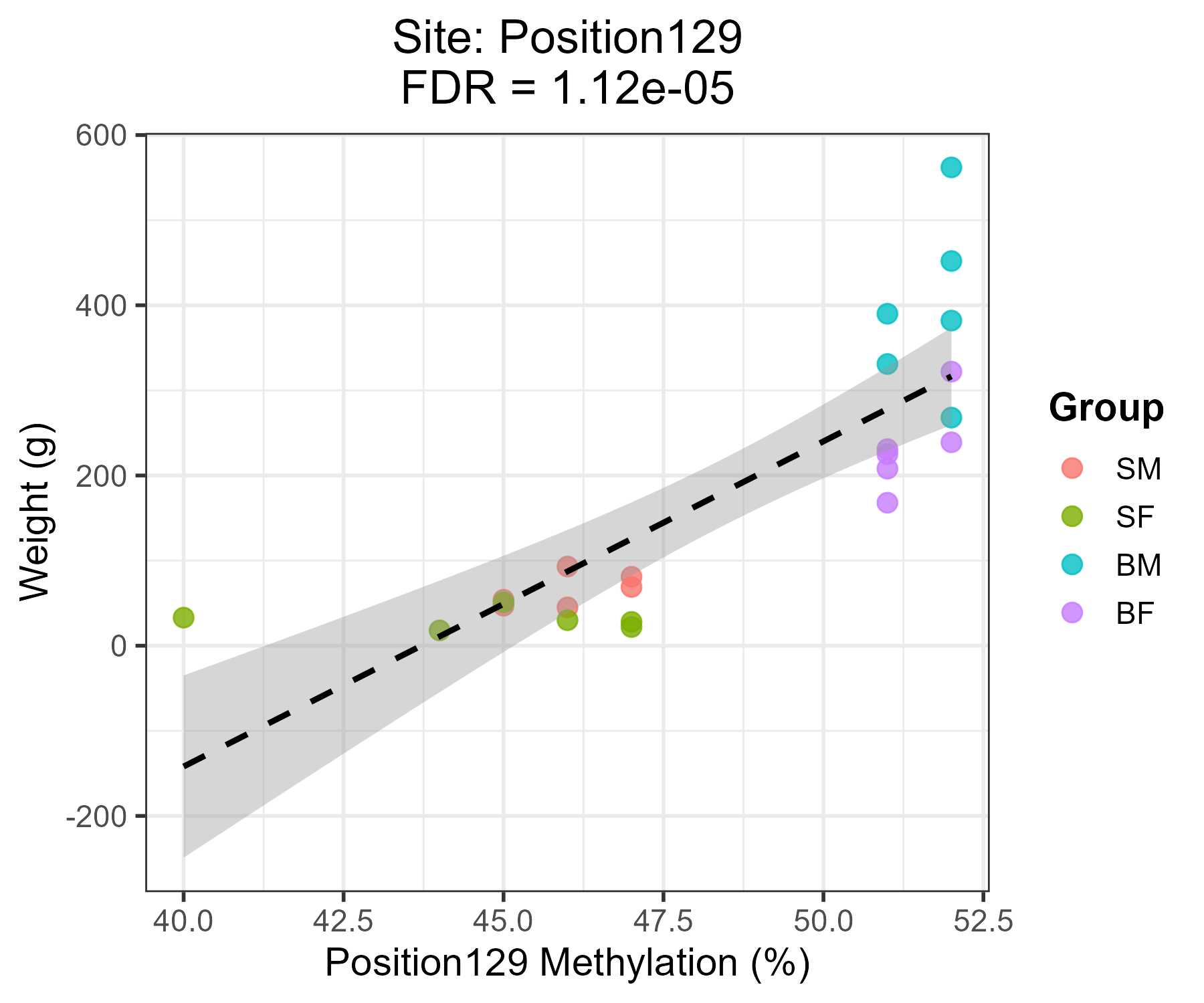

Supplement: Supplementary file 2 [file DataSheet1.zip › Regression_Plus_Strand/Position129_regression.tiff]

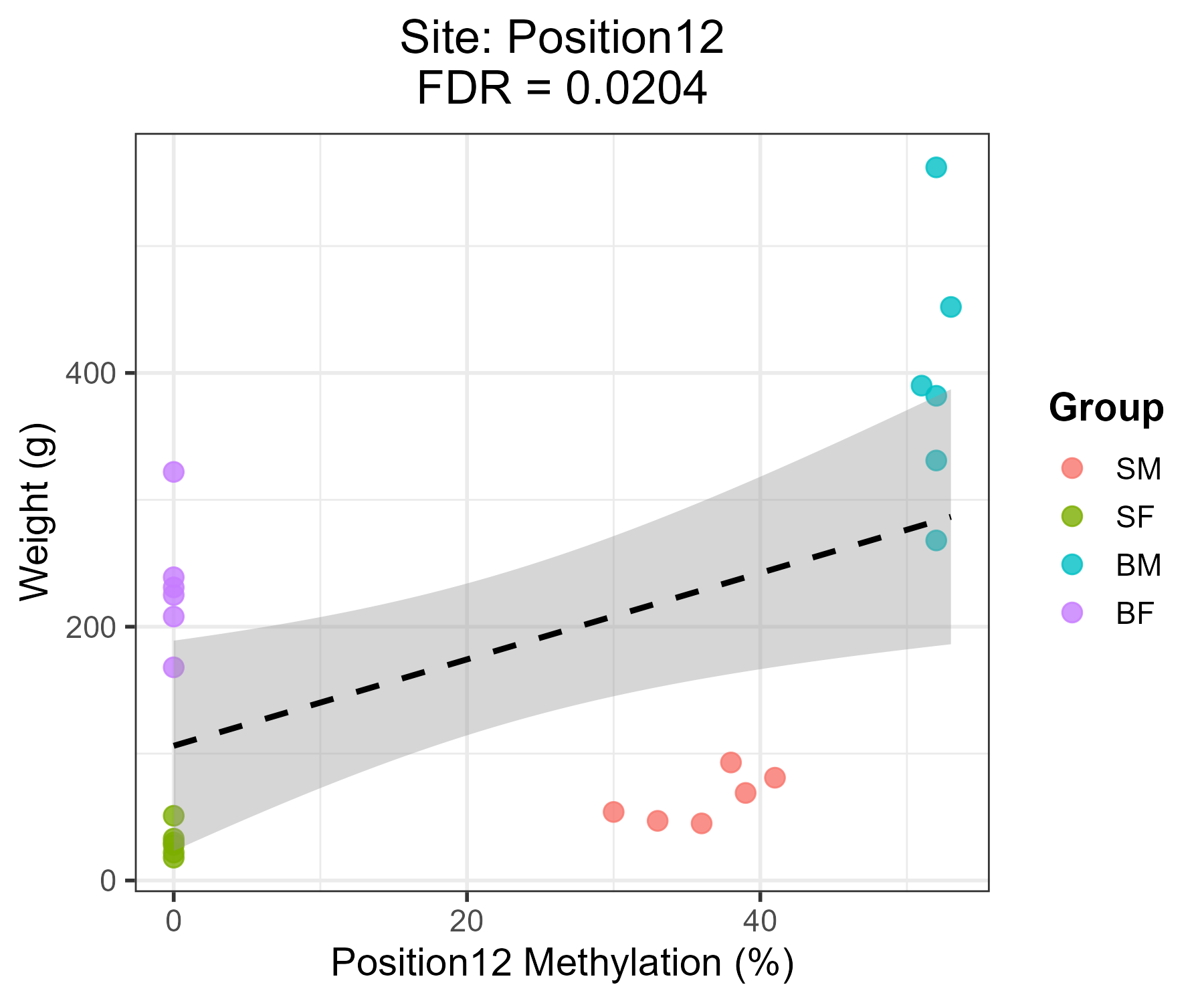

Supplement: Supplementary file 2 [file DataSheet1.zip › Regression_Plus_Strand/Position12_regression.tiff]

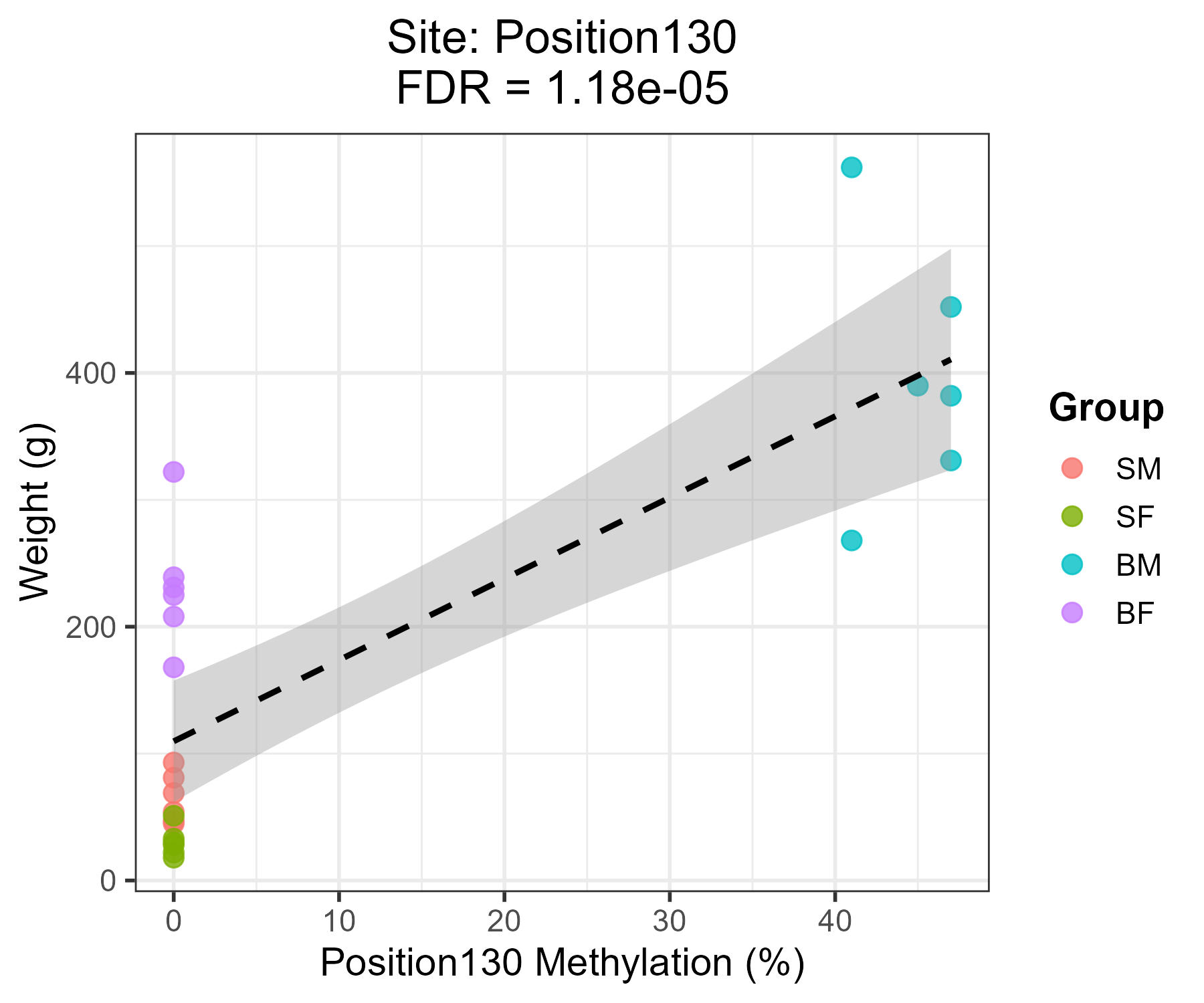

Supplement: Supplementary file 2 [file DataSheet1.zip › Regression_Plus_Strand/Position130_regression.tiff]

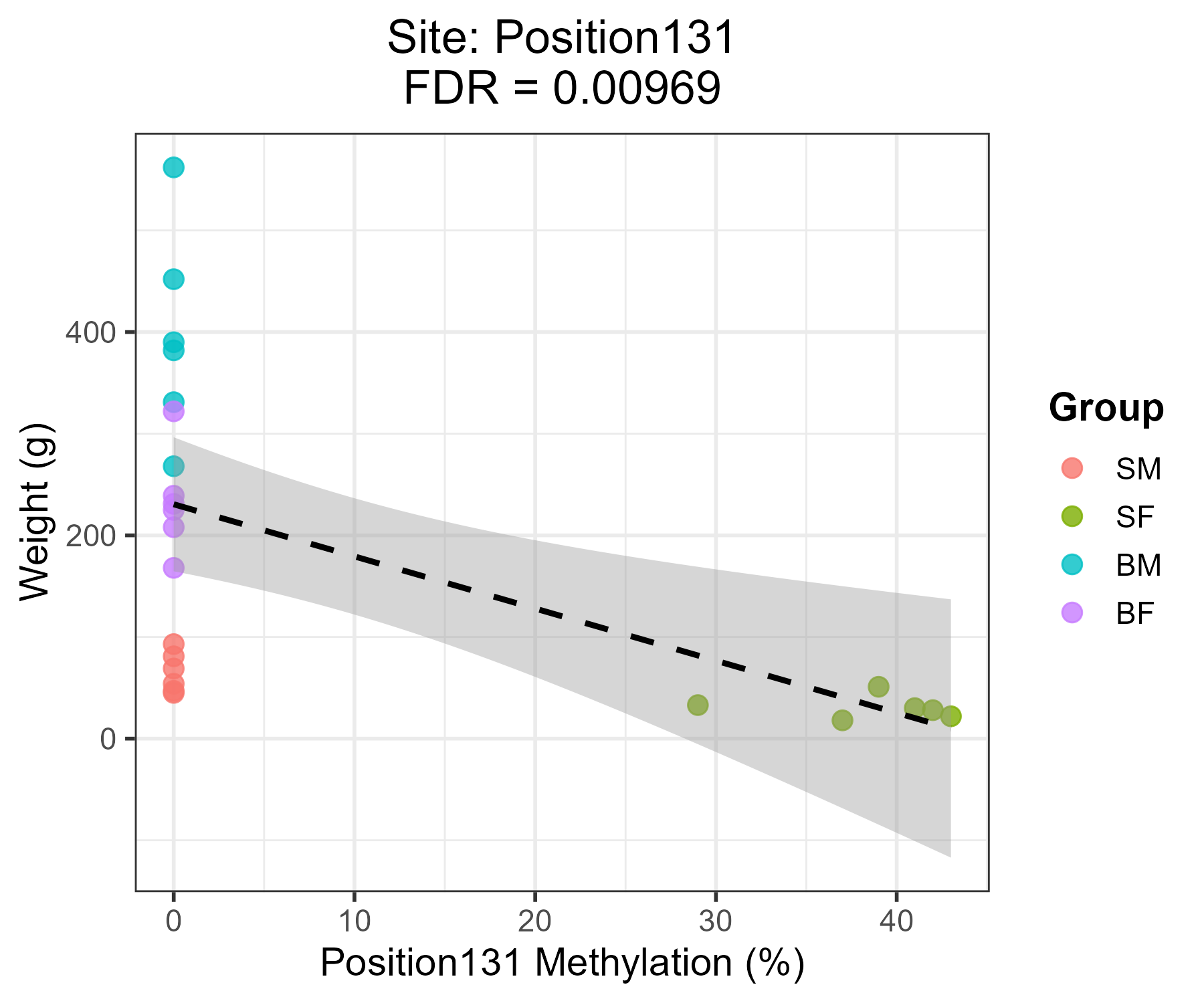

Supplement: Supplementary file 2 [file DataSheet1.zip › Regression_Plus_Strand/Position131_regression.tiff]

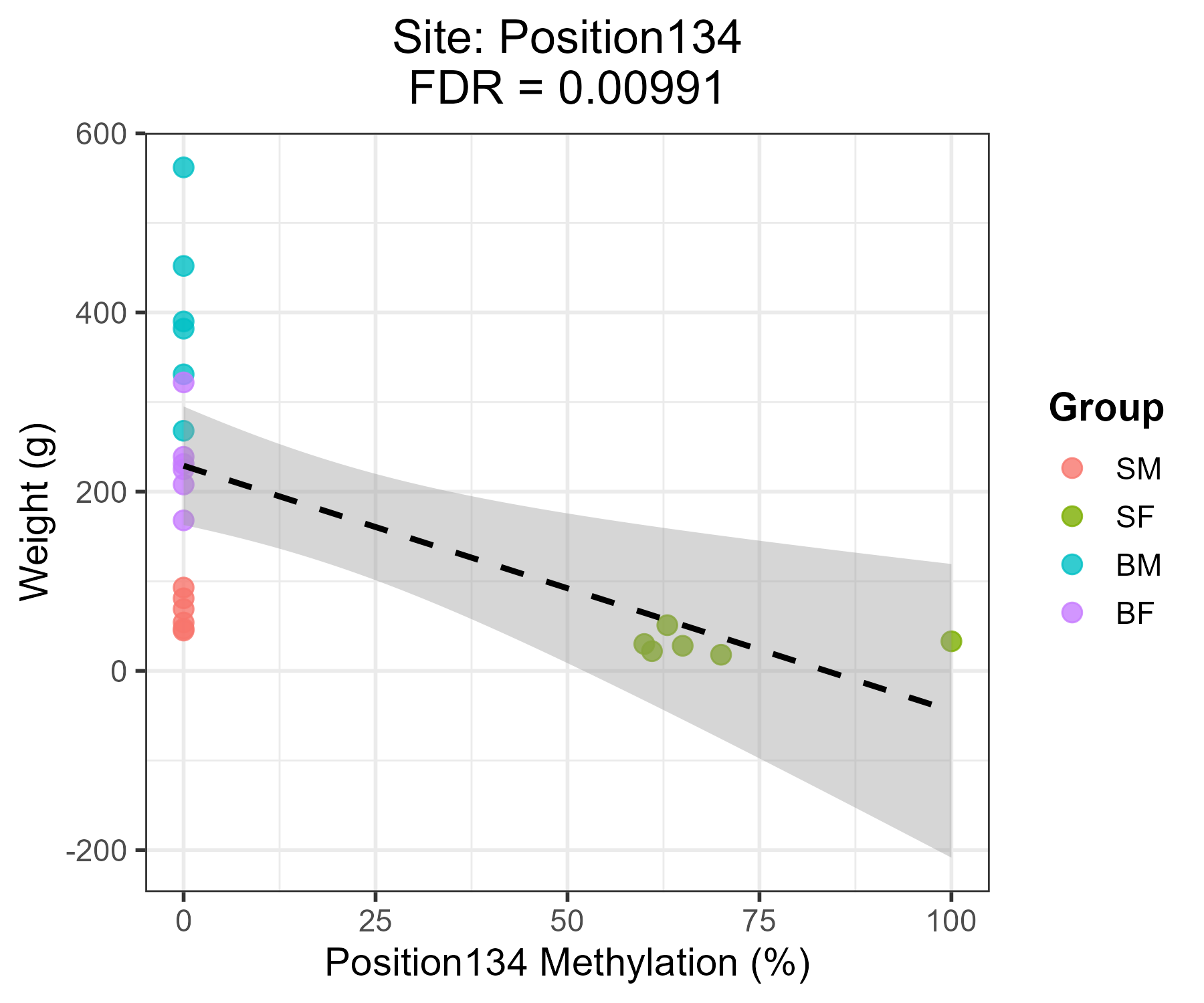

Supplement: Supplementary file 2 [file DataSheet1.zip › Regression_Plus_Strand/Position134_regression.tiff]

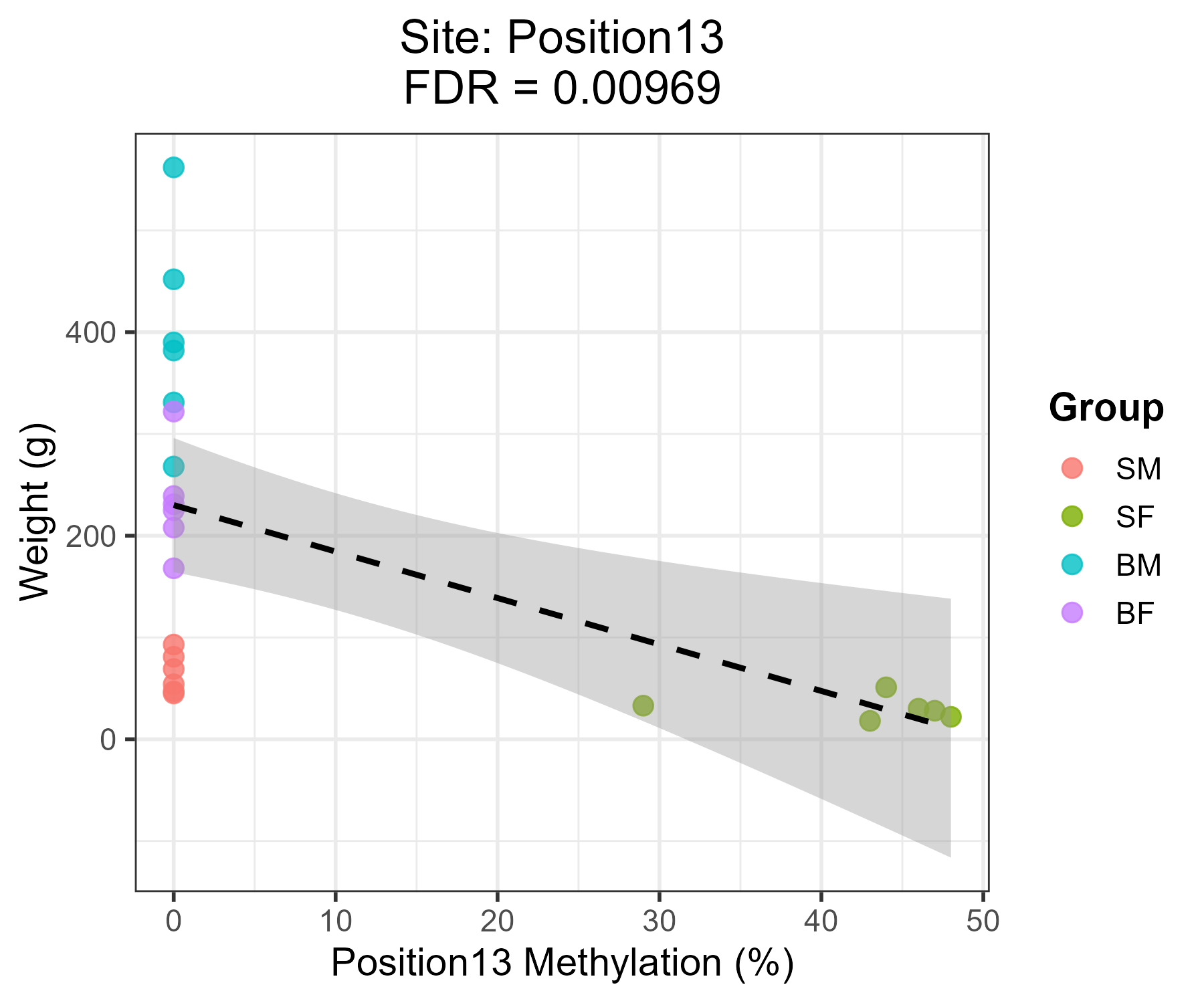

Supplement: Supplementary file 2 [file DataSheet1.zip › Regression_Plus_Strand/Position13_regression.tiff]

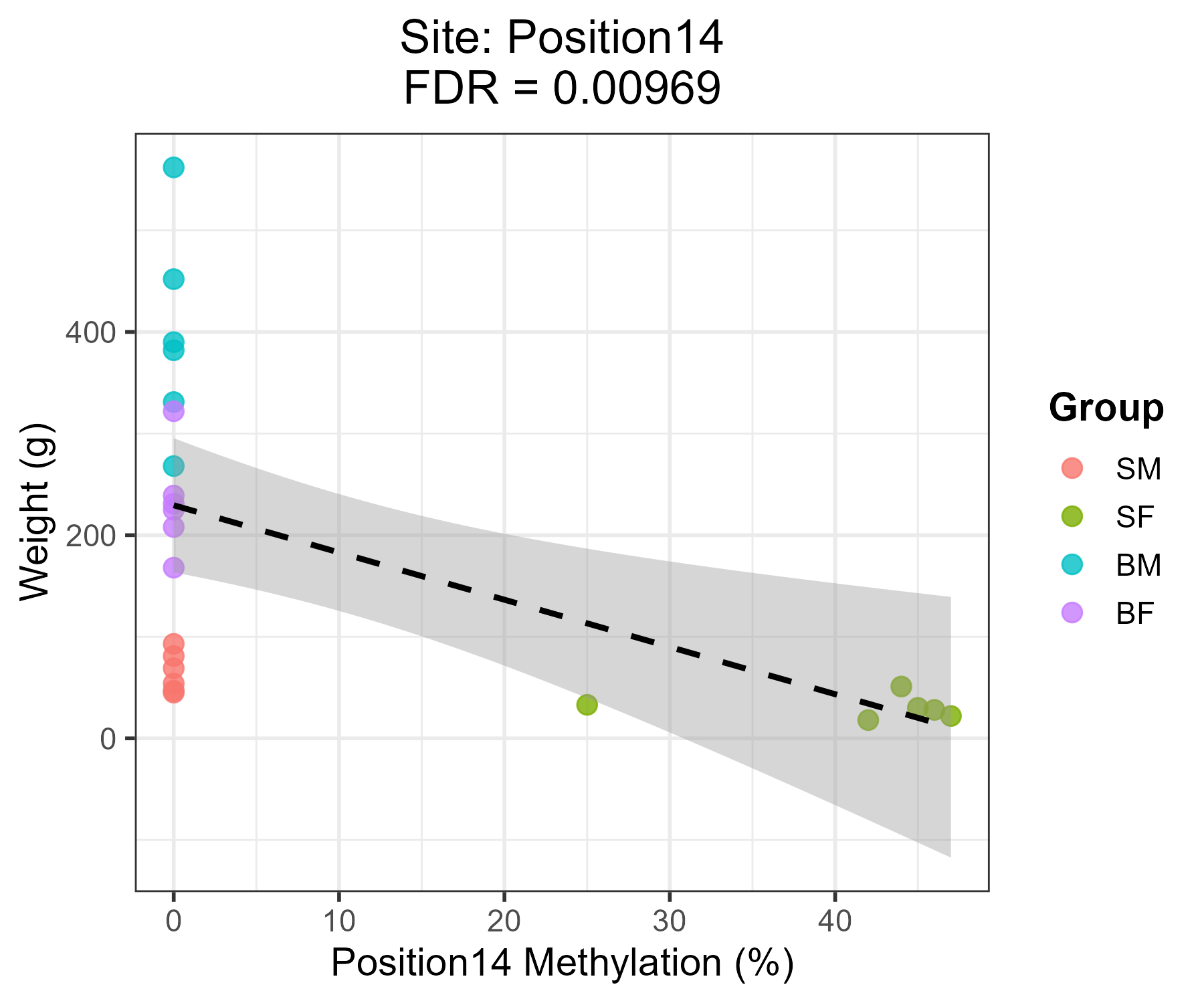

Supplement: Supplementary file 2 [file DataSheet1.zip › Regression_Plus_Strand/Position14_regression.tiff]

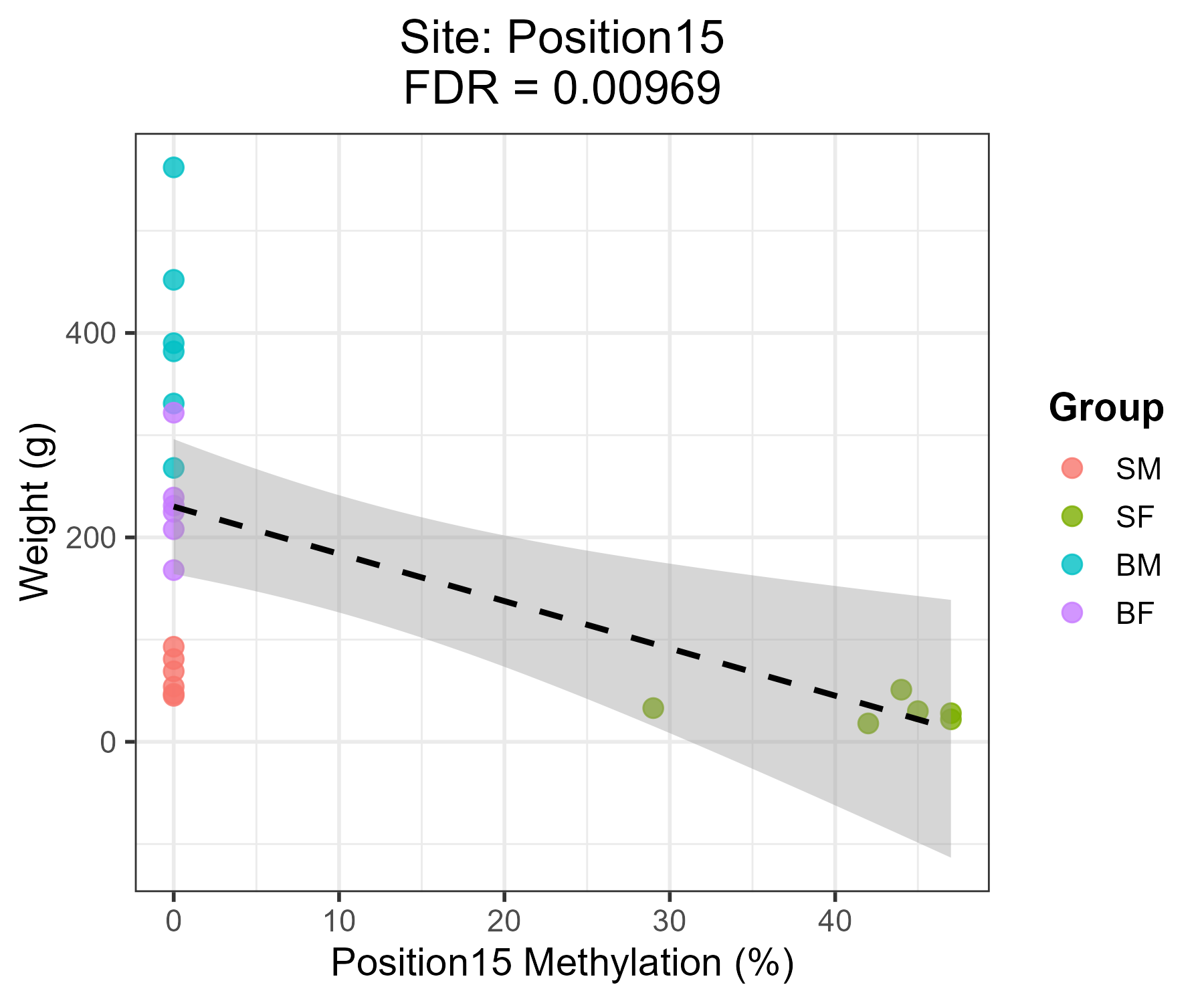

Supplement: Supplementary file 2 [file DataSheet1.zip › Regression_Plus_Strand/Position15_regression.tiff]

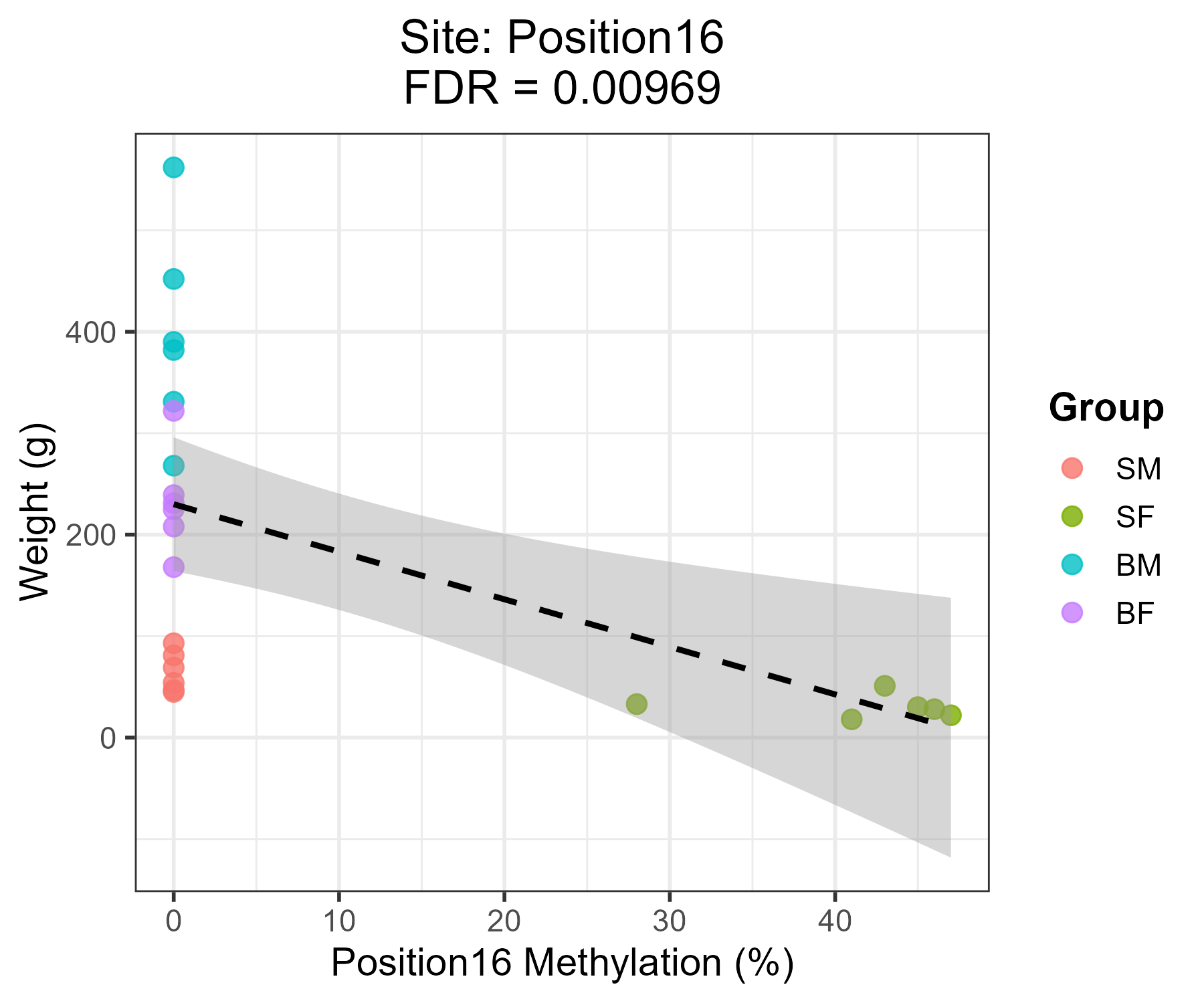

Supplement: Supplementary file 2 [file DataSheet1.zip › Regression_Plus_Strand/Position16_regression.tiff]

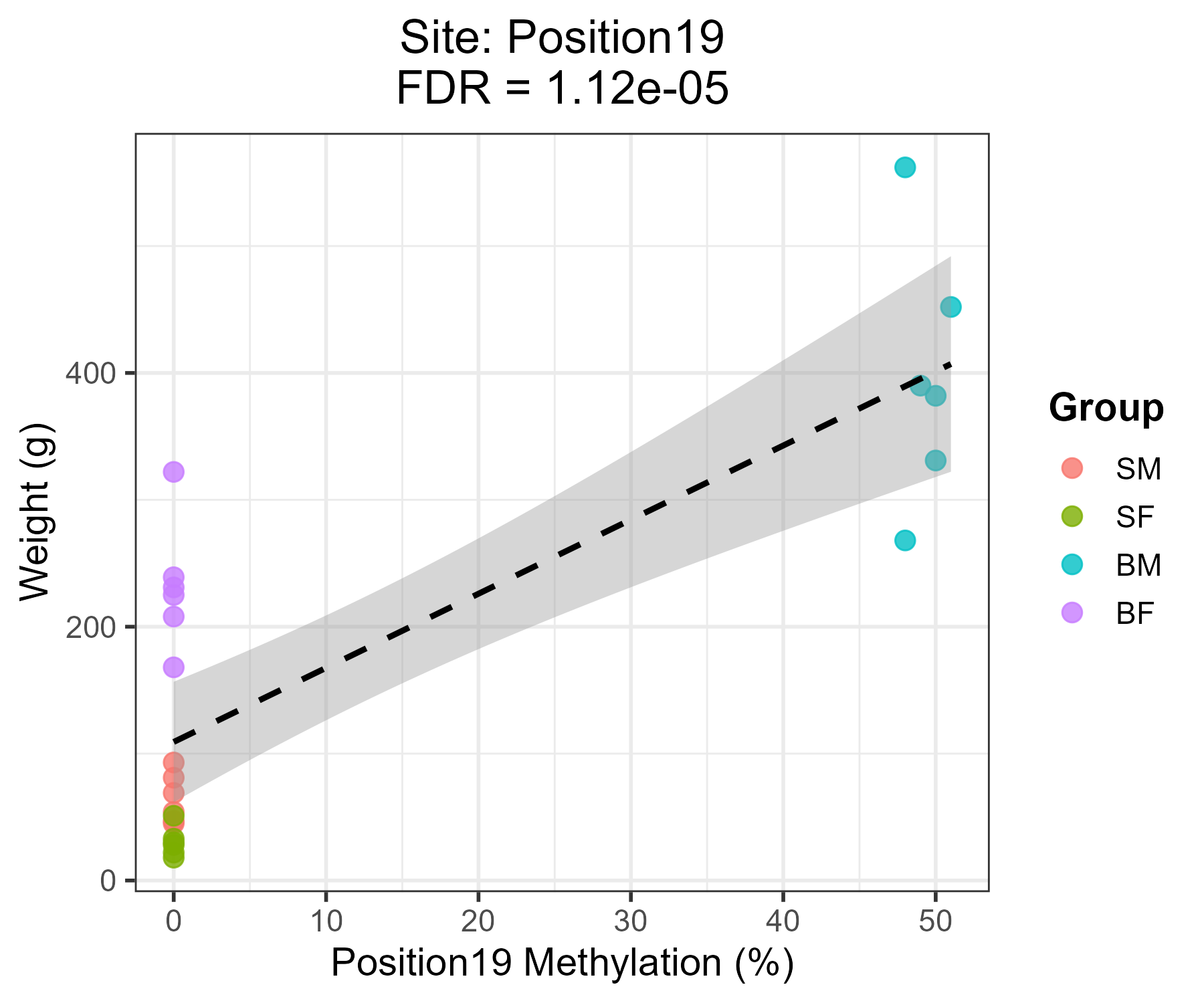

Supplement: Supplementary file 2 [file DataSheet1.zip › Regression_Plus_Strand/Position19_regression.tiff]

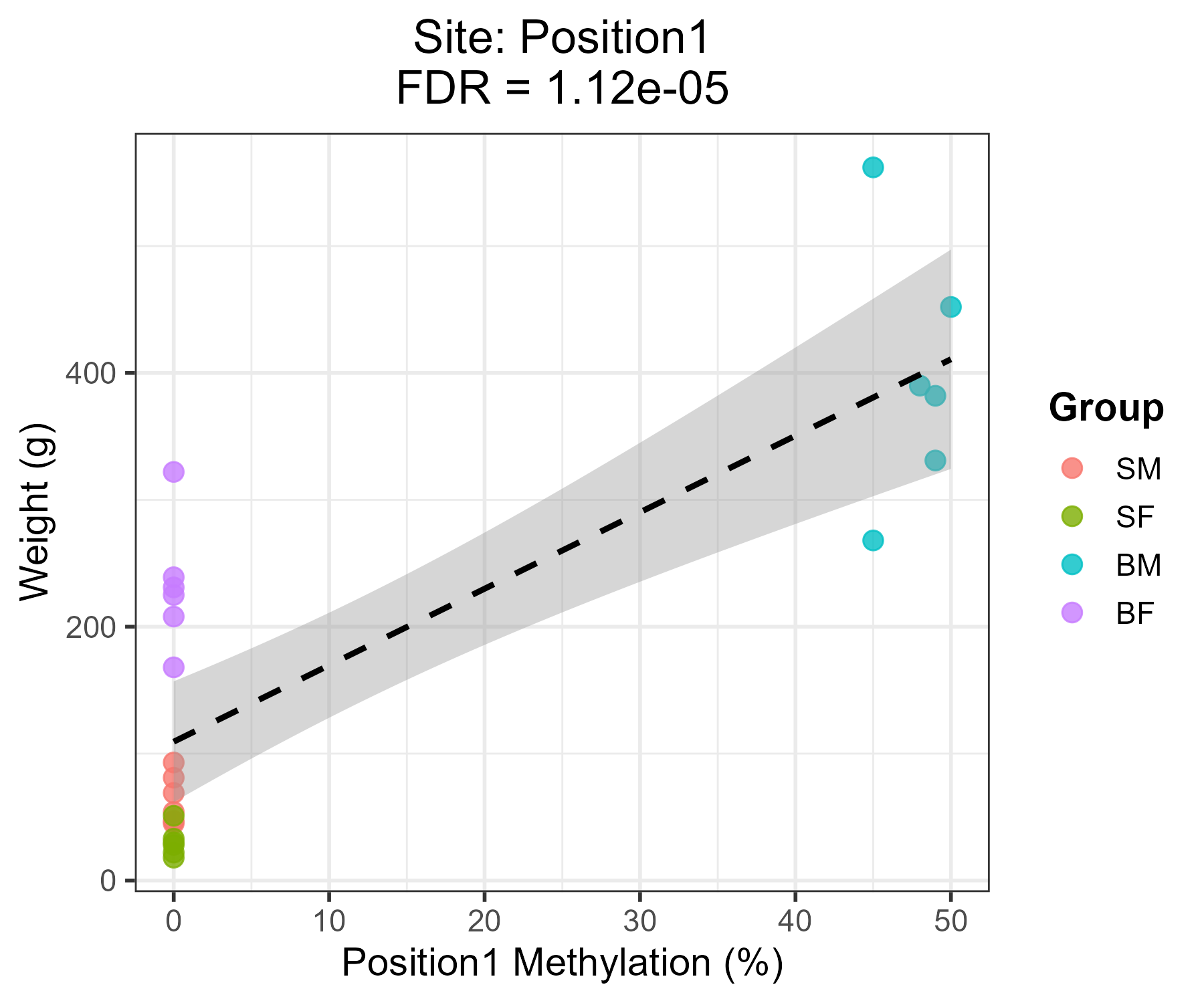

Supplement: Supplementary file 2 [file DataSheet1.zip › Regression_Plus_Strand/Position1_regression.tiff]

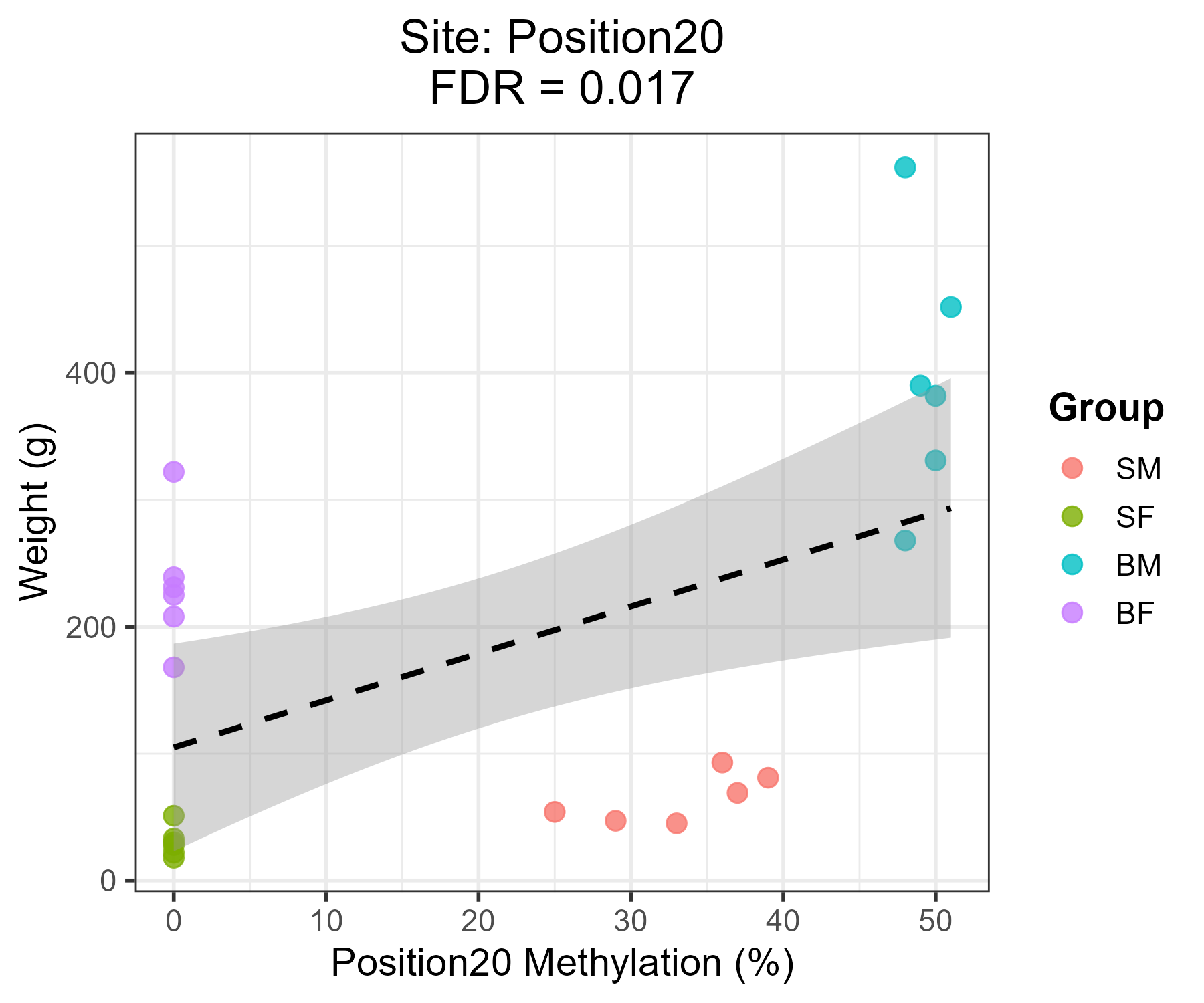

Supplement: Supplementary file 2 [file DataSheet1.zip › Regression_Plus_Strand/Position20_regression.tiff]

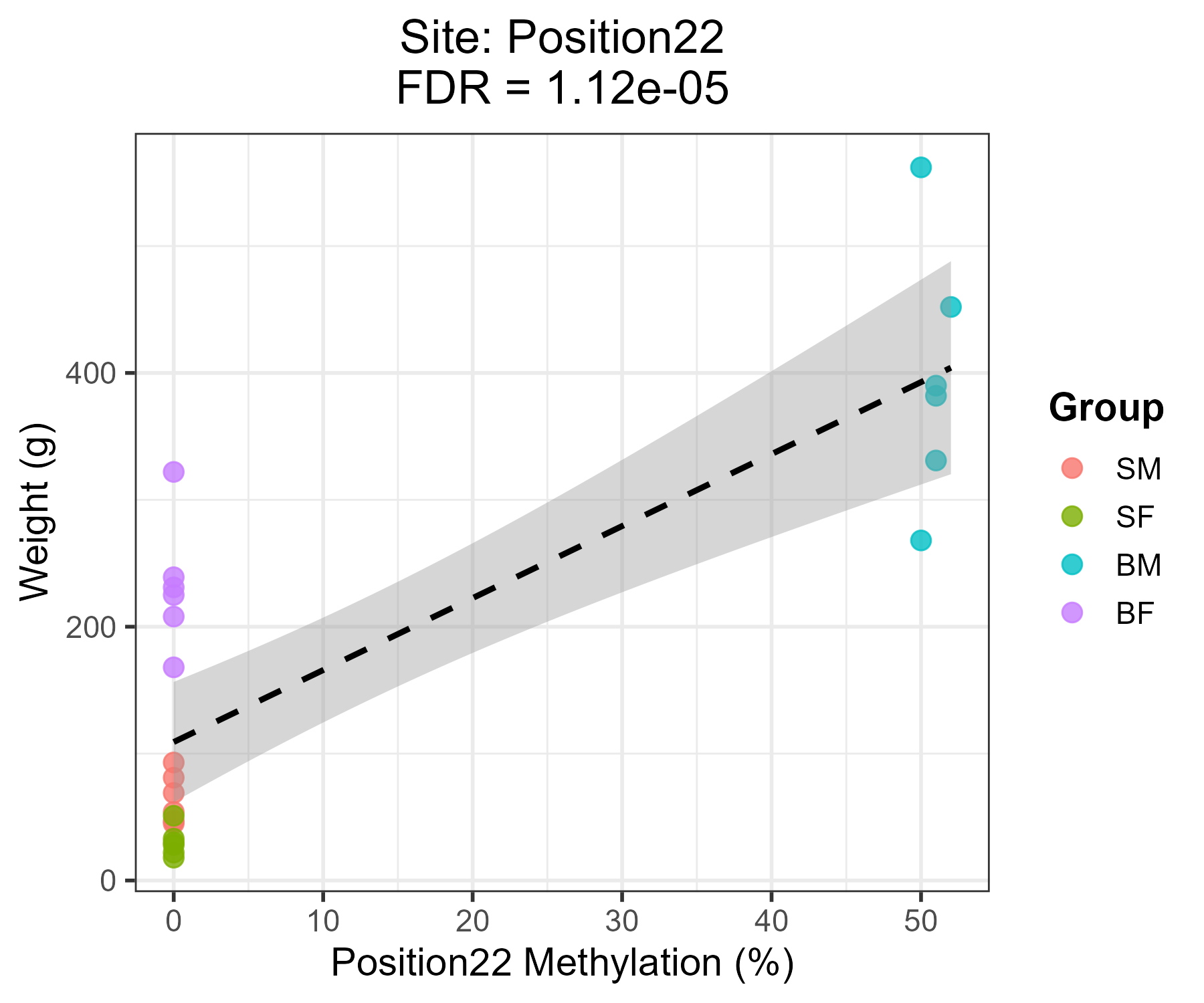

Supplement: Supplementary file 2 [file DataSheet1.zip › Regression_Plus_Strand/Position22_regression.tiff]

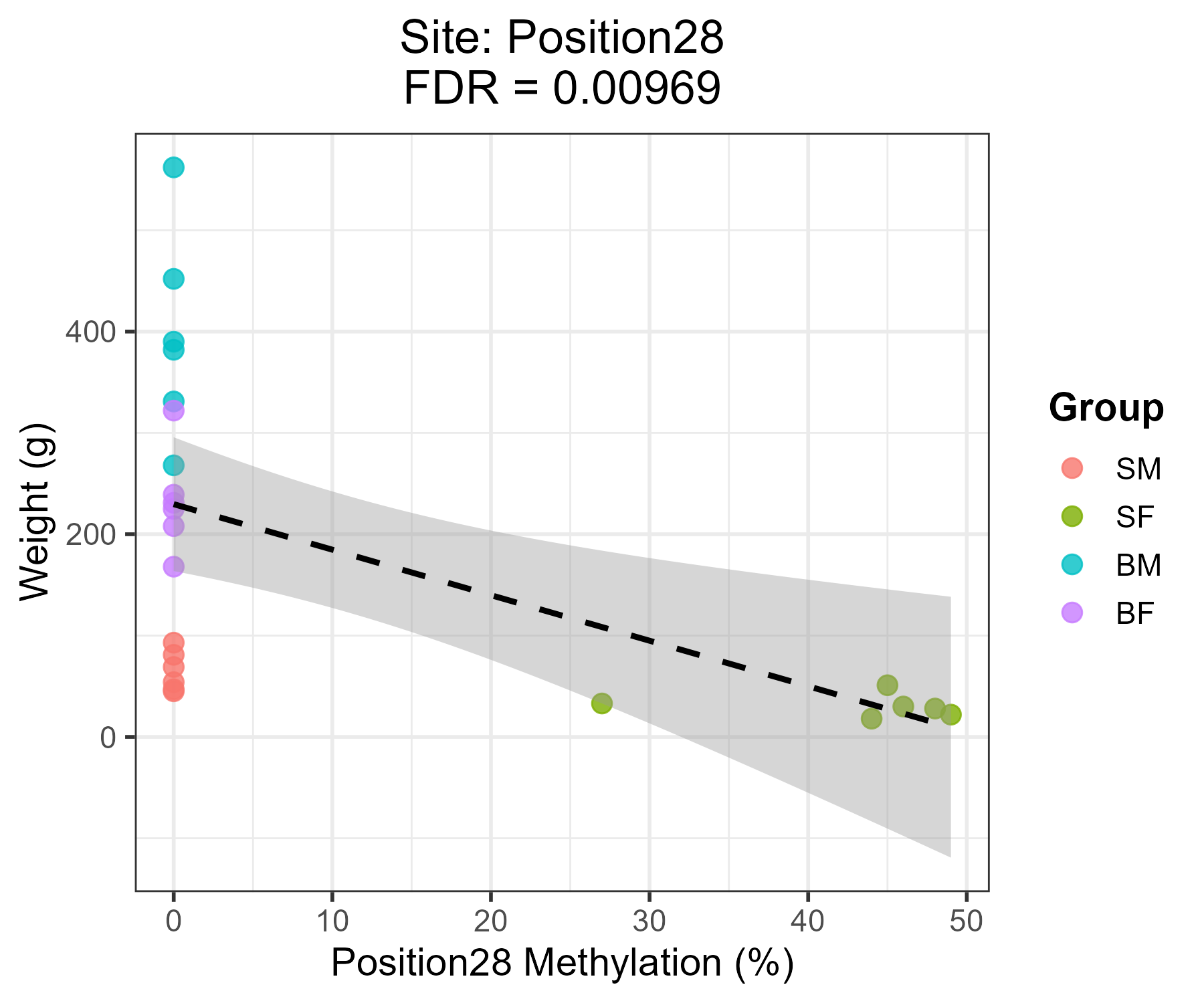

Supplement: Supplementary file 2 [file DataSheet1.zip › Regression_Plus_Strand/Position28_regression.tiff]

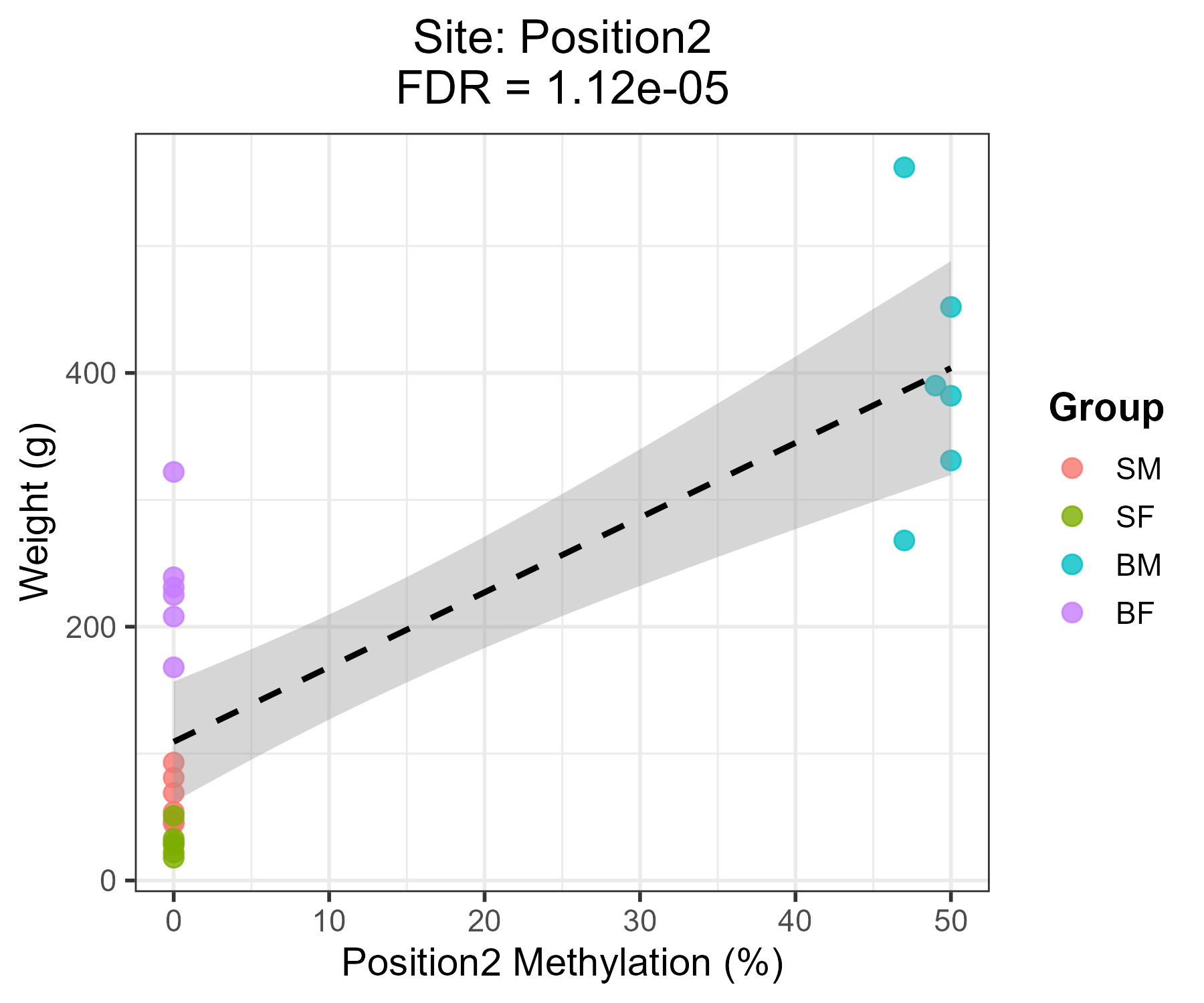

Supplement: Supplementary file 2 [file DataSheet1.zip › Regression_Plus_Strand/Position2_regression.tiff]

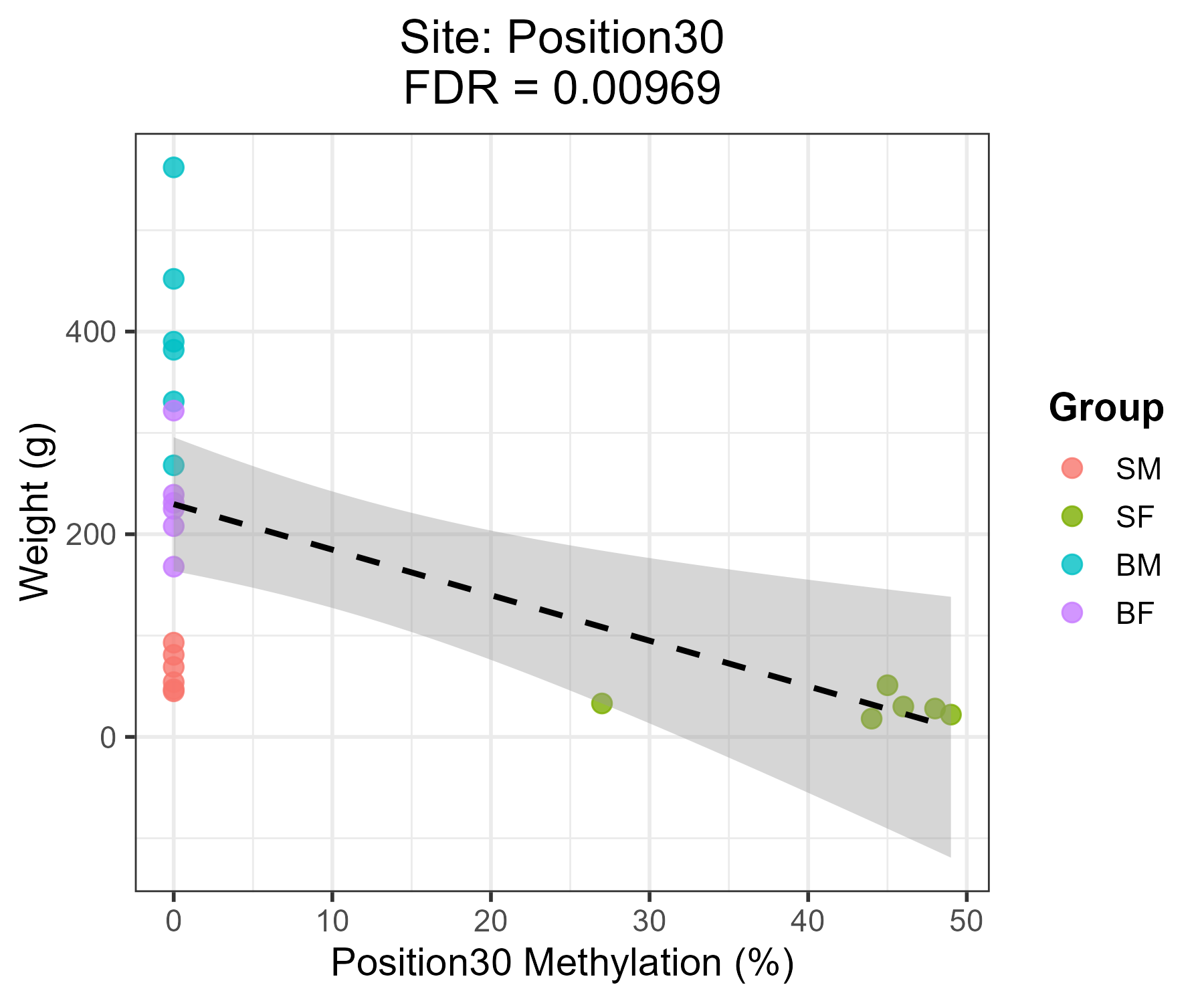

Supplement: Supplementary file 2 [file DataSheet1.zip › Regression_Plus_Strand/Position30_regression.tiff]

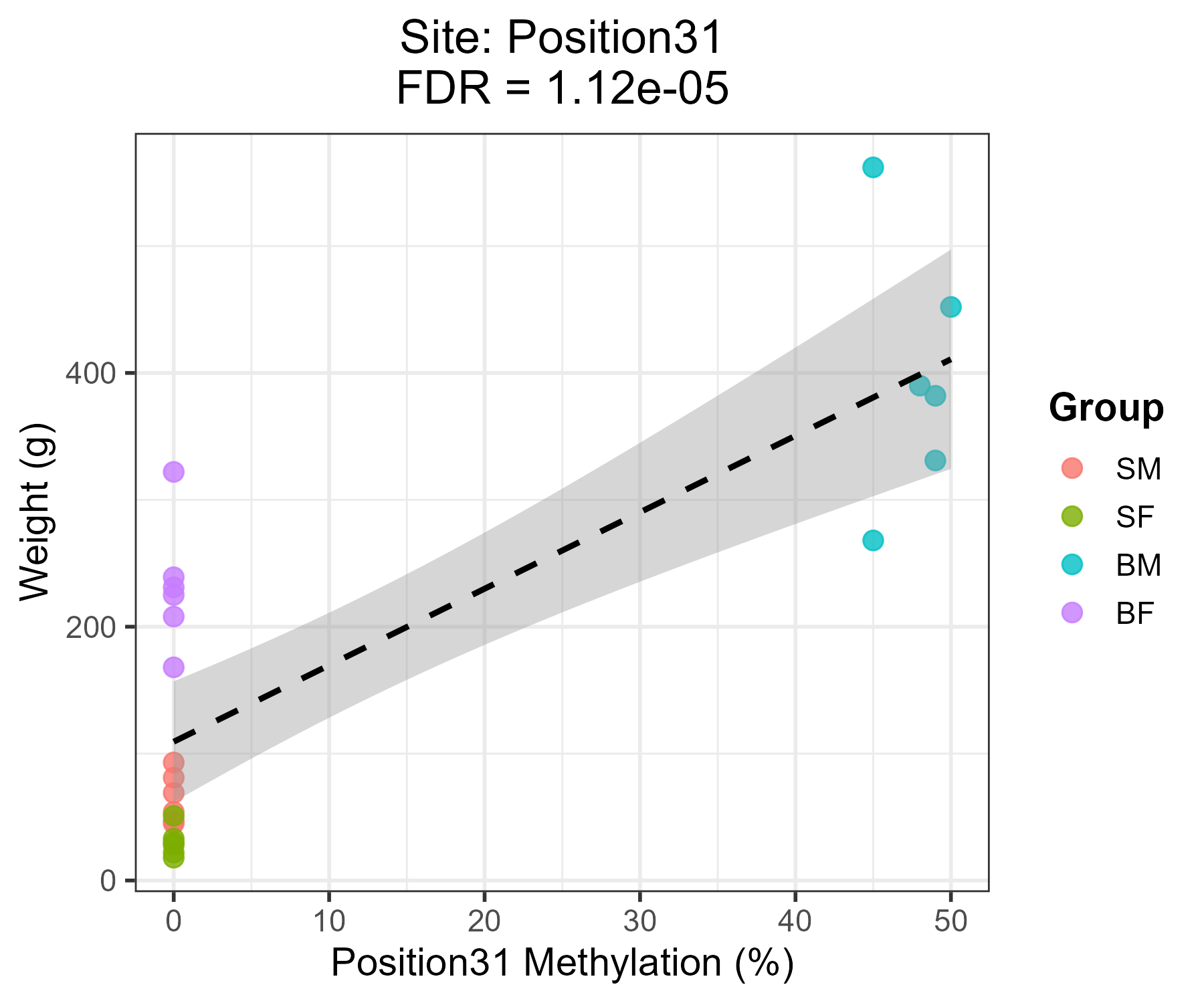

Supplement: Supplementary file 2 [file DataSheet1.zip › Regression_Plus_Strand/Position31_regression.tiff]

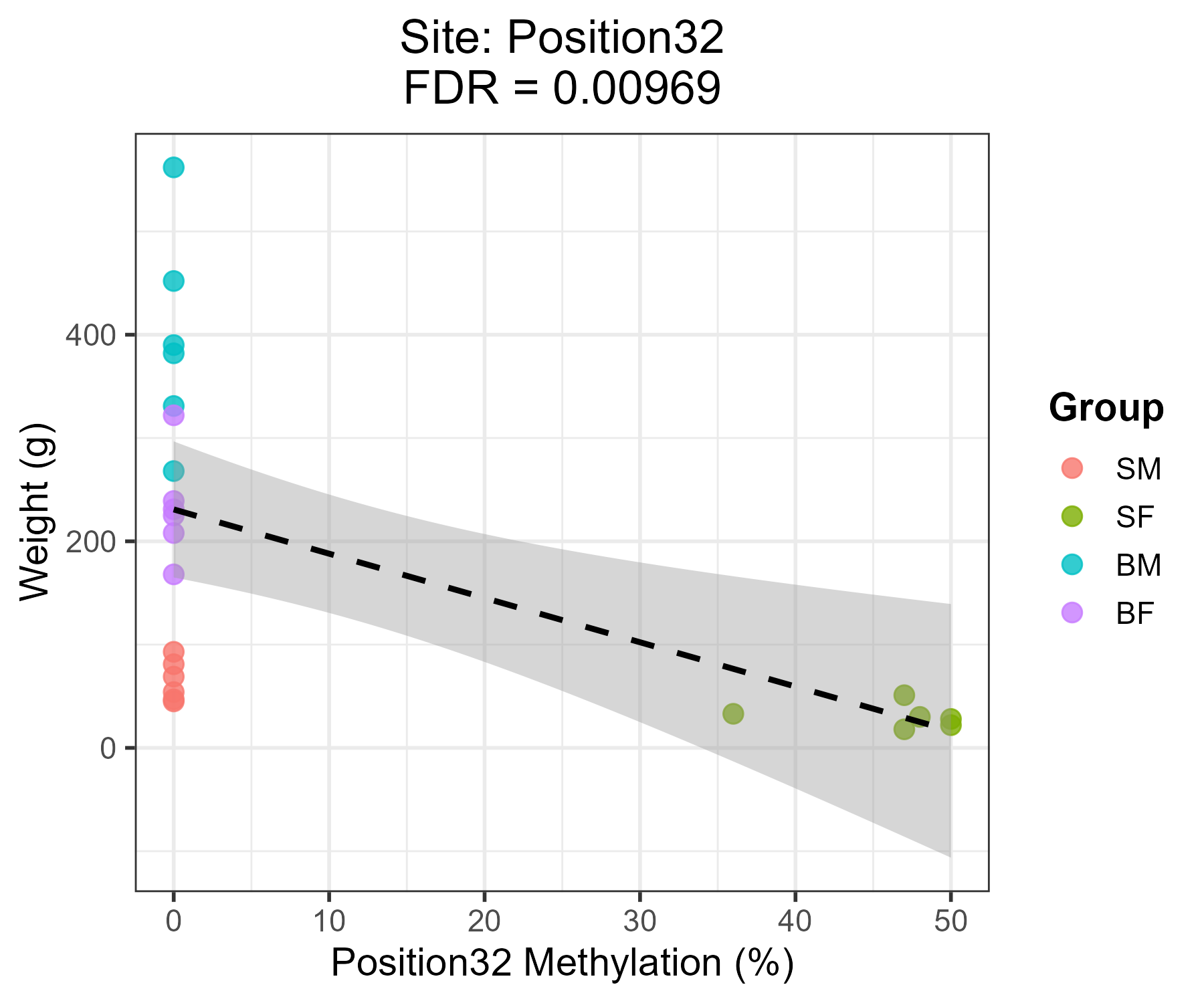

Supplement: Supplementary file 2 [file DataSheet1.zip › Regression_Plus_Strand/Position32_regression.tiff]

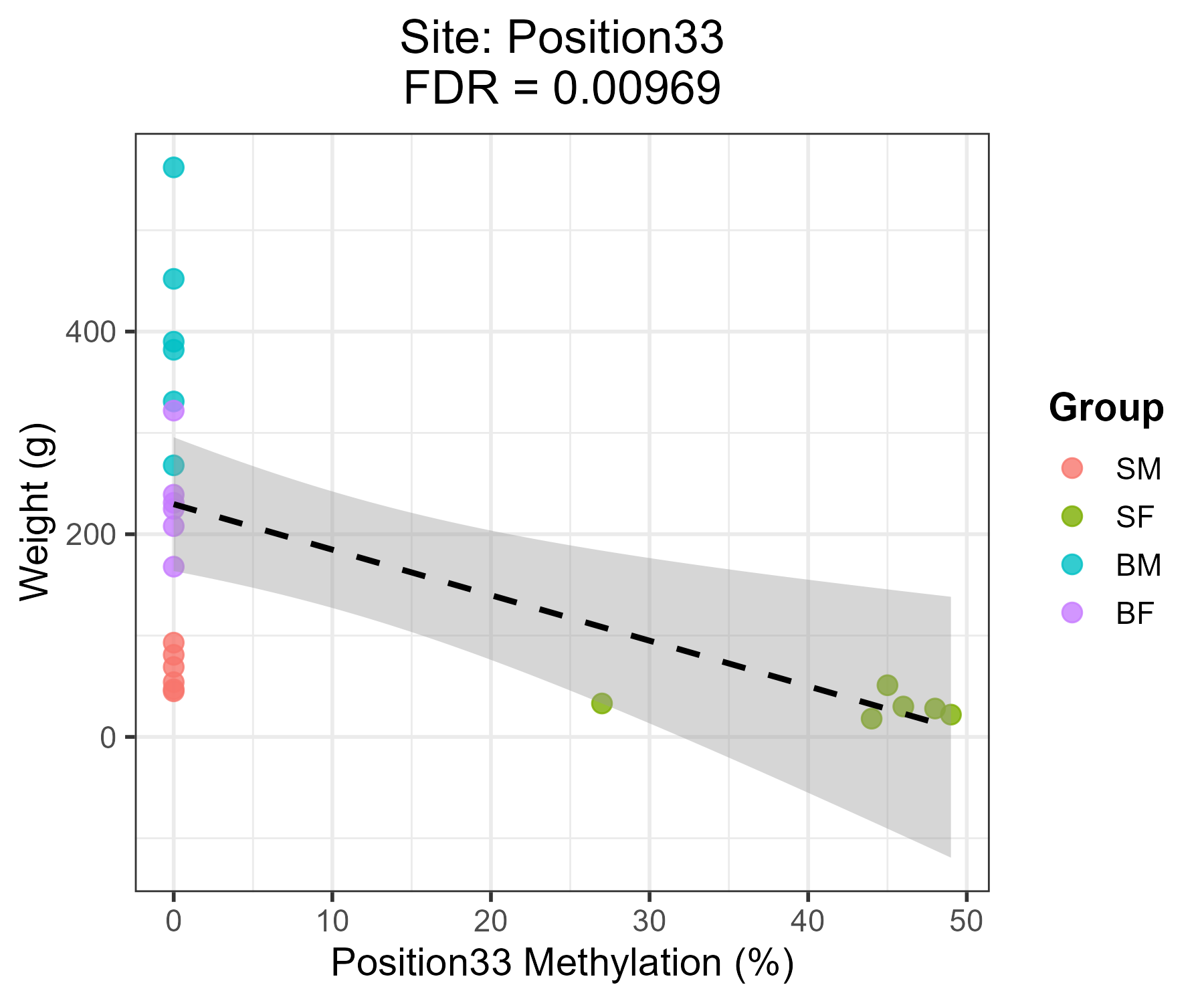

Supplement: Supplementary file 2 [file DataSheet1.zip › Regression_Plus_Strand/Position33_regression.tiff]

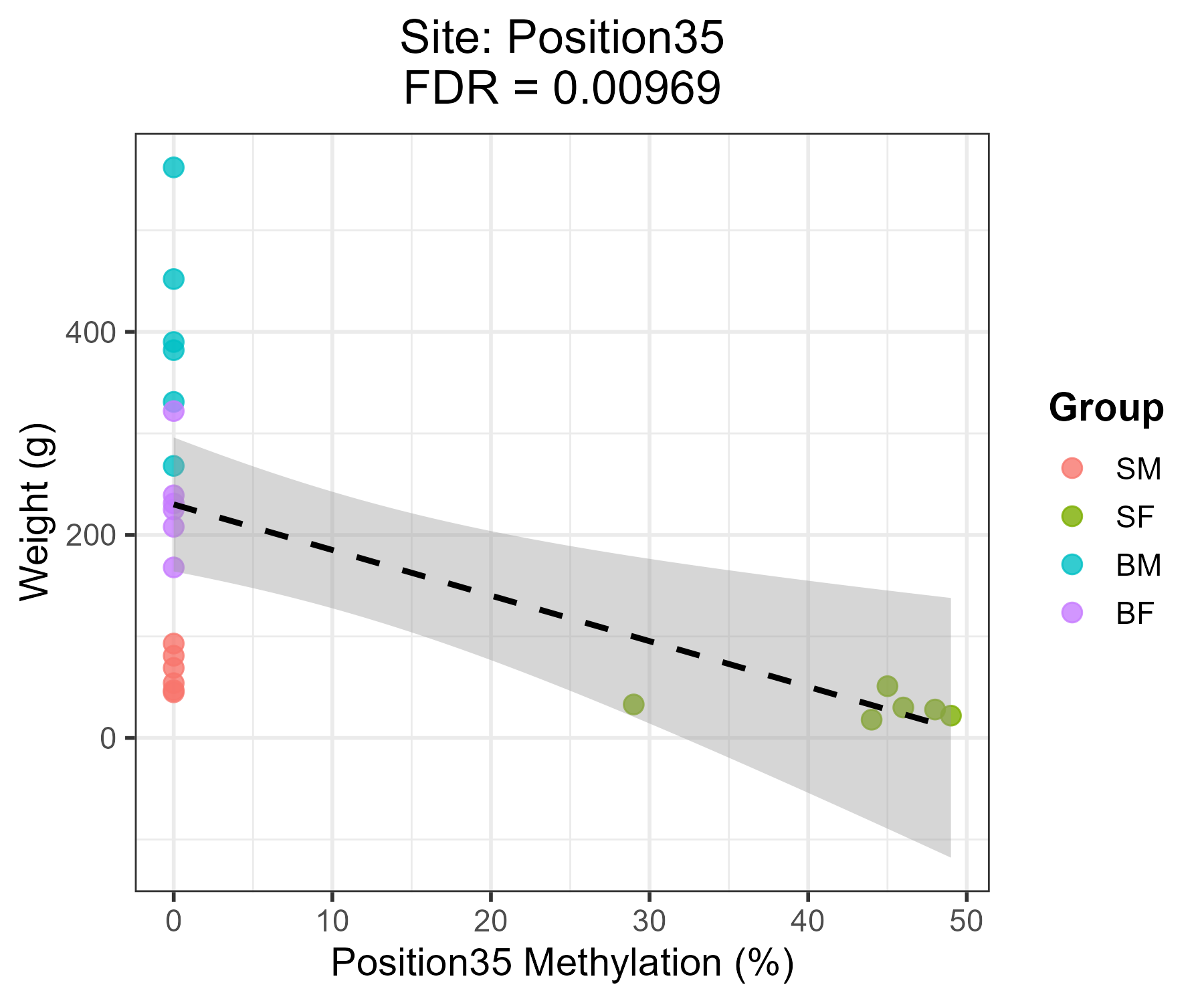

Supplement: Supplementary file 2 [file DataSheet1.zip › Regression_Plus_Strand/Position35_regression.tiff]

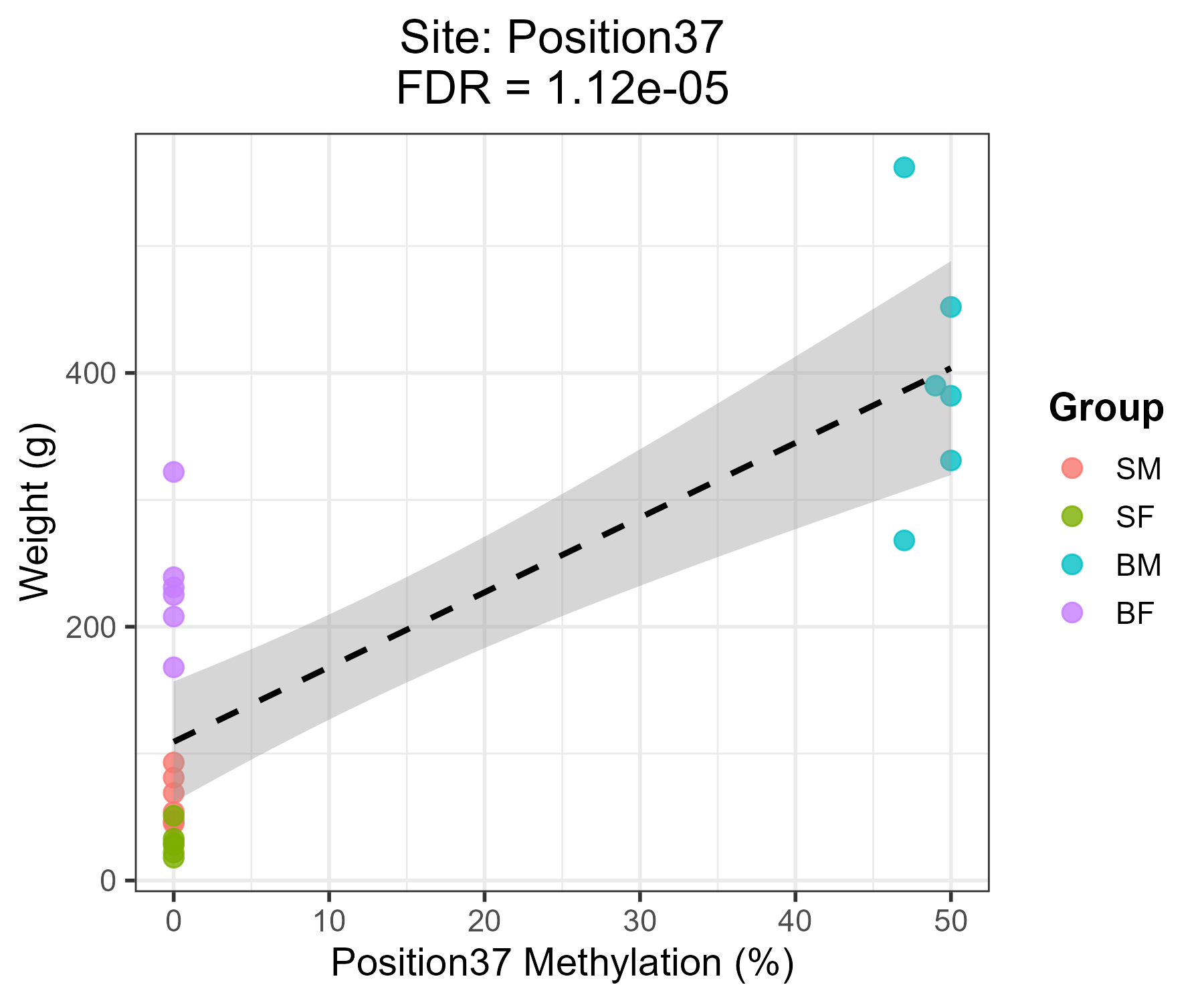

Supplement: Supplementary file 2 [file DataSheet1.zip › Regression_Plus_Strand/Position37_regression.tiff]

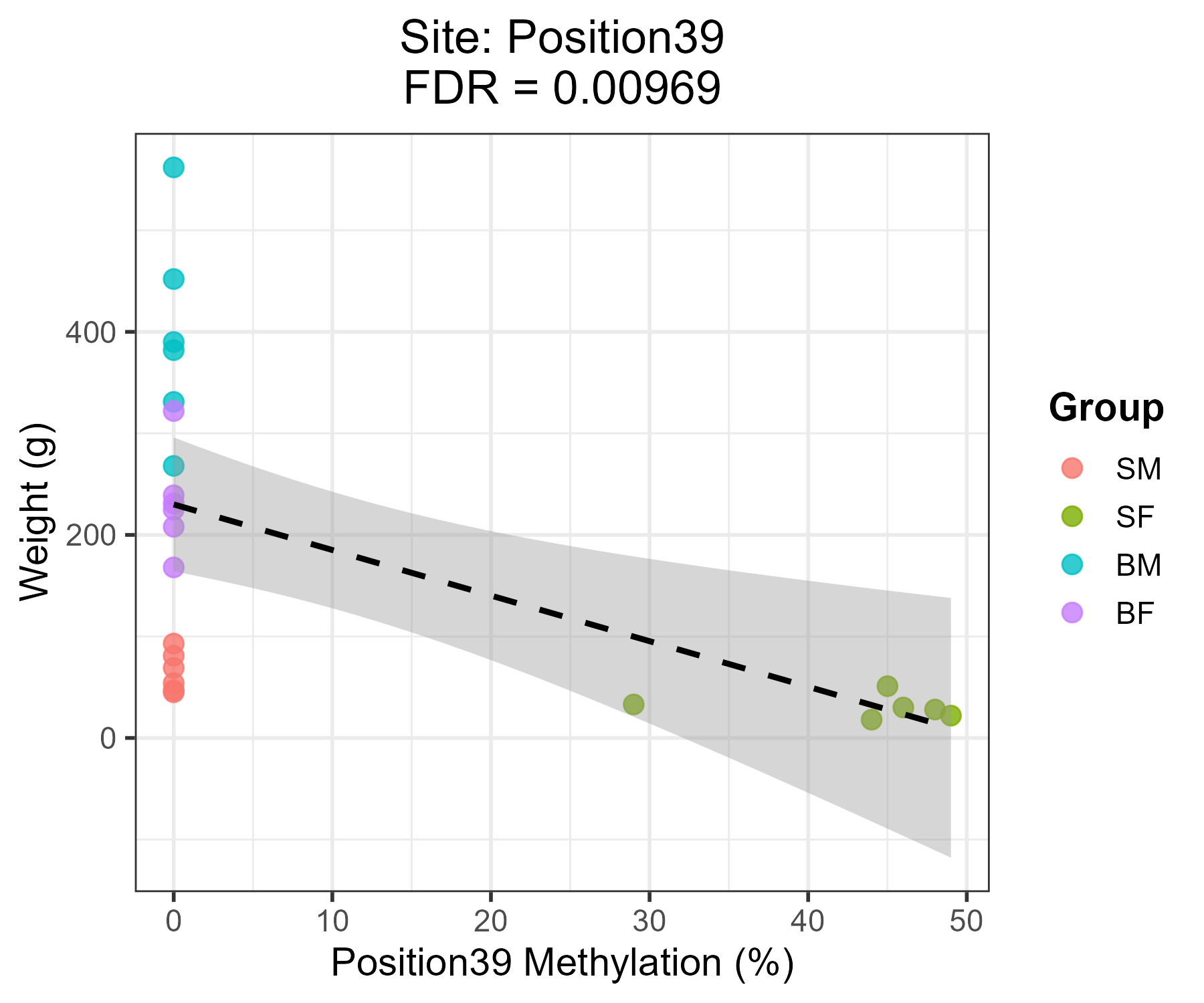

Supplement: Supplementary file 2 [file DataSheet1.zip › Regression_Plus_Strand/Position39_regression.tiff]

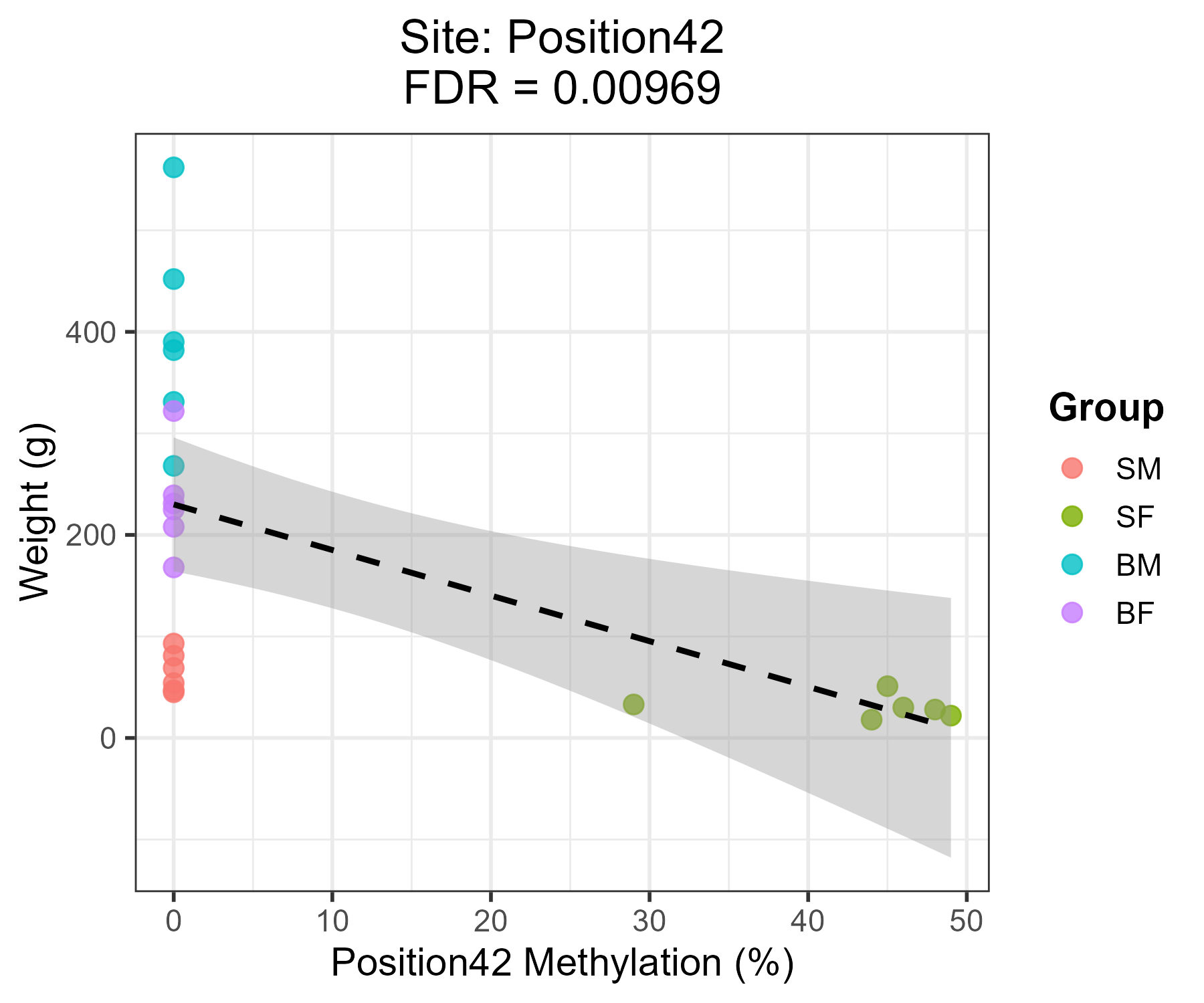

Supplement: Supplementary file 2 [file DataSheet1.zip › Regression_Plus_Strand/Position42_regression.tiff]

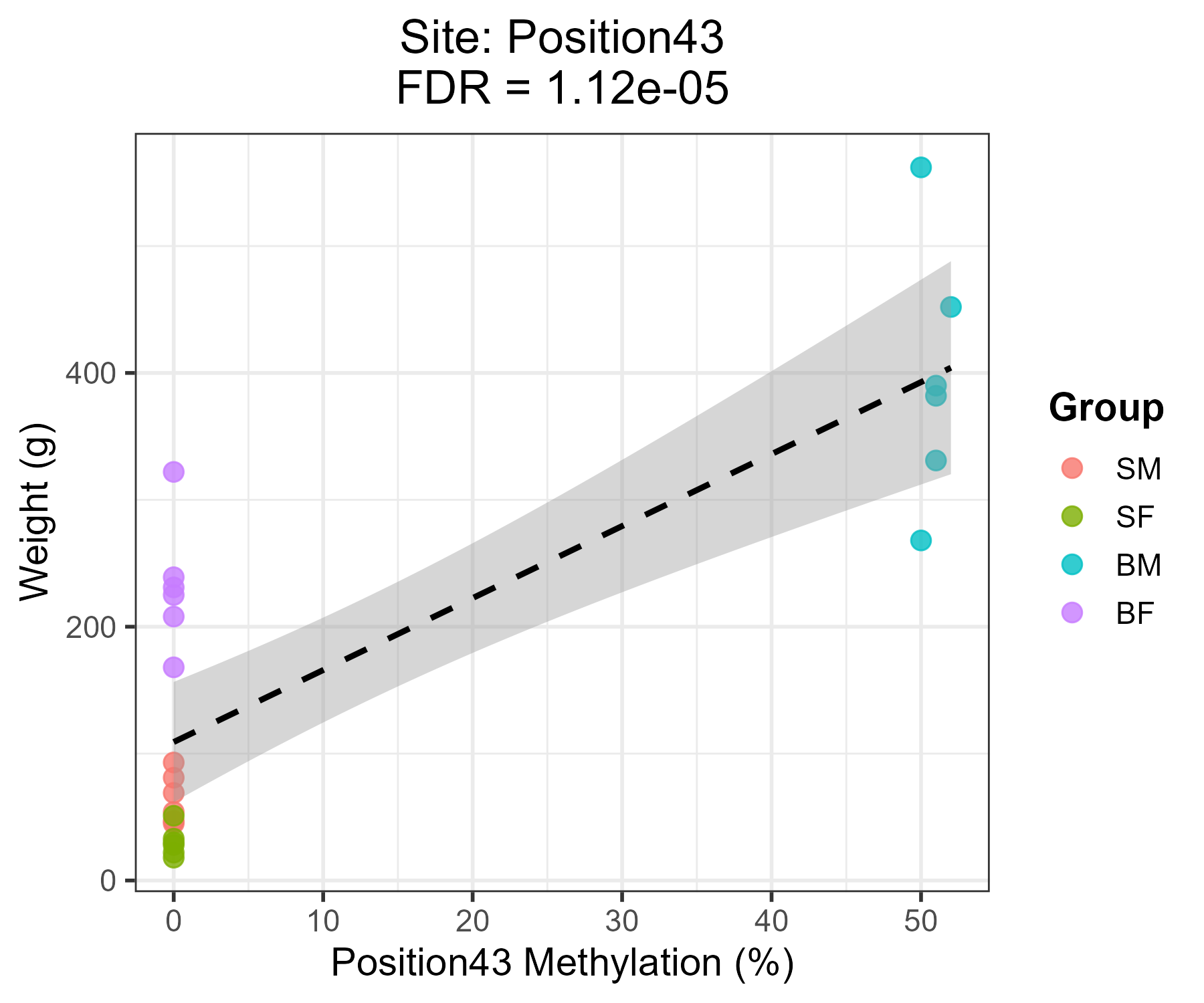

Supplement: Supplementary file 2 [file DataSheet1.zip › Regression_Plus_Strand/Position43_regression.tiff]

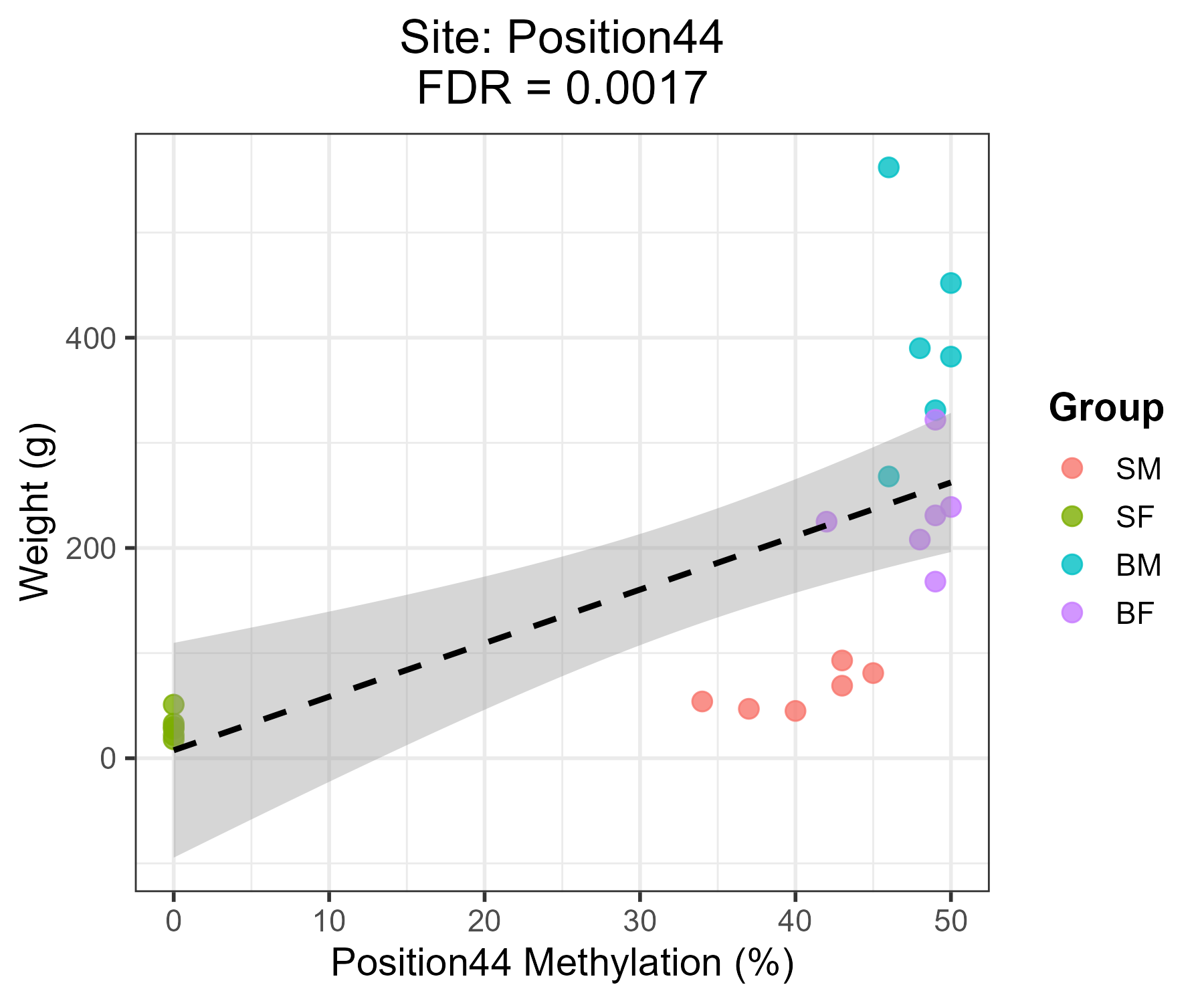

Supplement: Supplementary file 2 [file DataSheet1.zip › Regression_Plus_Strand/Position44_regression.tiff]

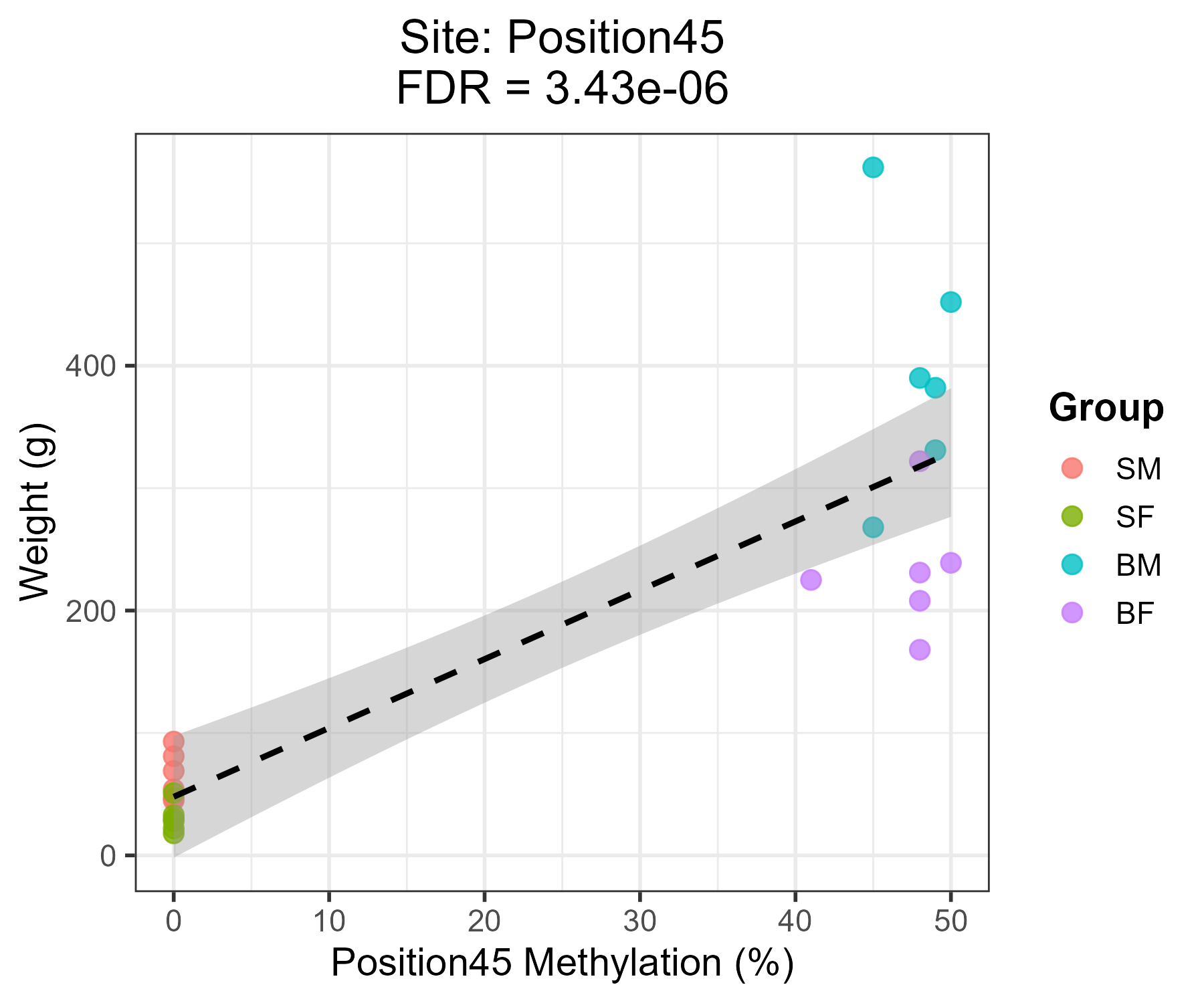

Supplement: Supplementary file 2 [file DataSheet1.zip › Regression_Plus_Strand/Position45_regression.tiff]

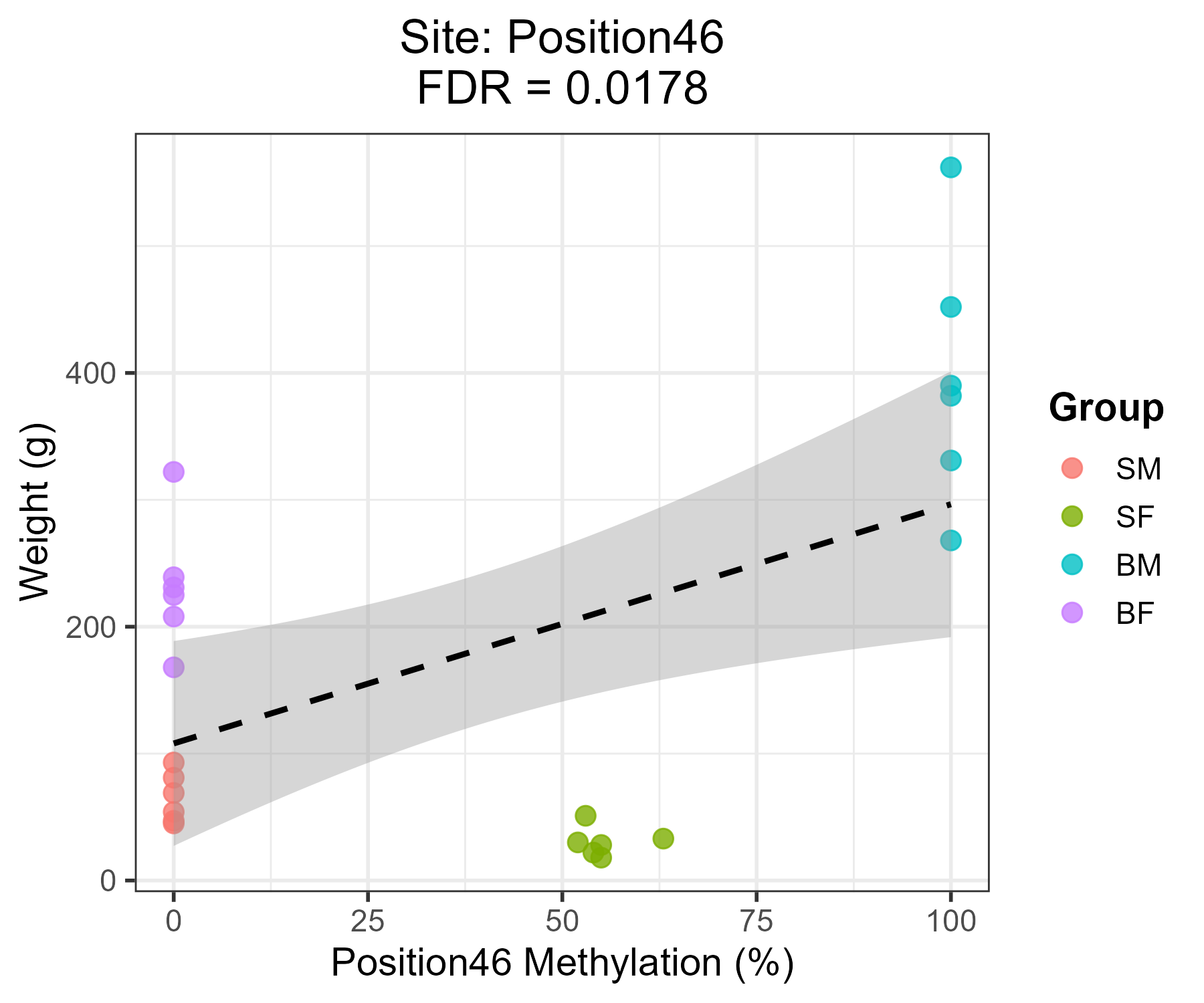

Supplement: Supplementary file 2 [file DataSheet1.zip › Regression_Plus_Strand/Position46_regression.tiff]

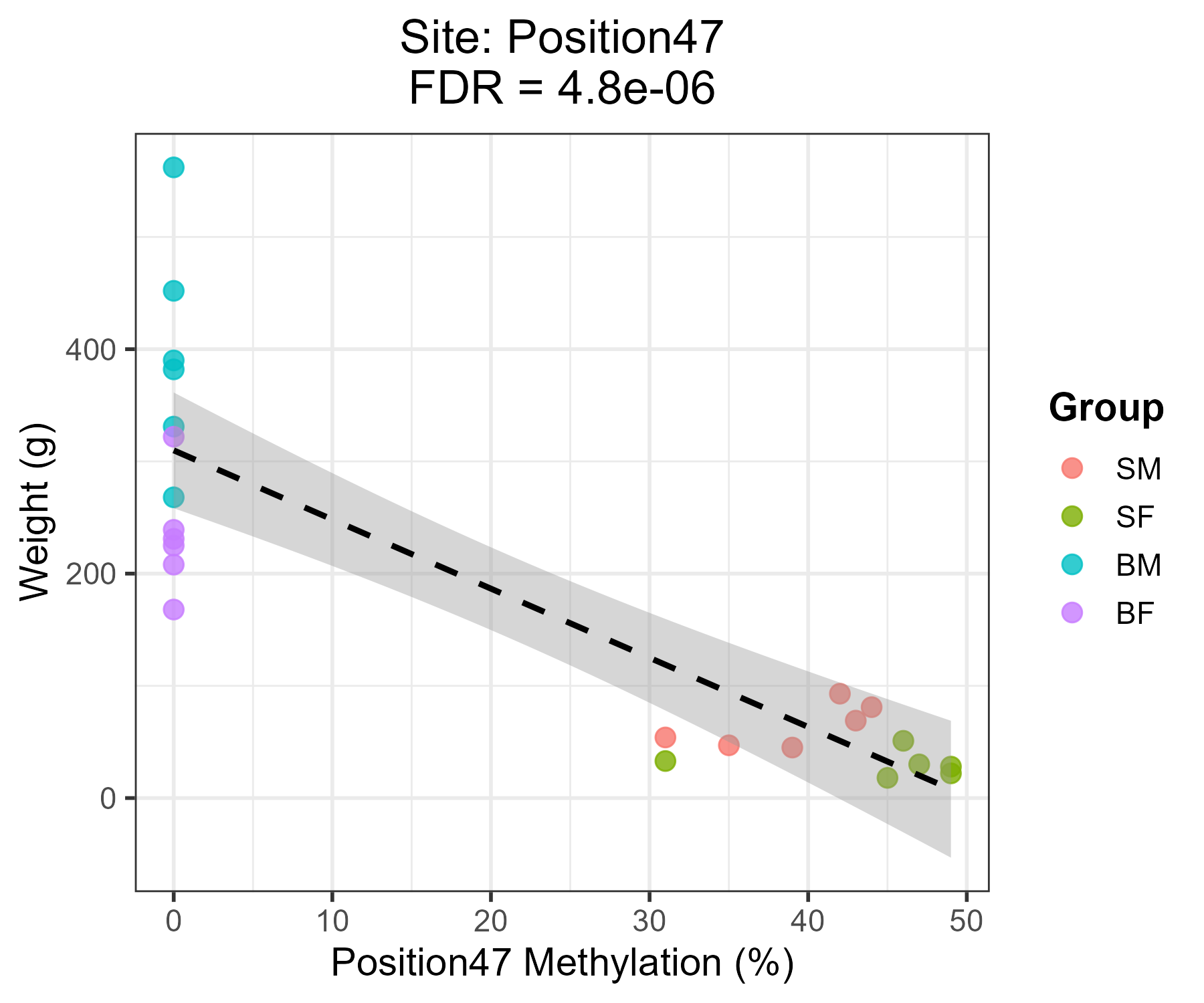

Supplement: Supplementary file 2 [file DataSheet1.zip › Regression_Plus_Strand/Position47_regression.tiff]

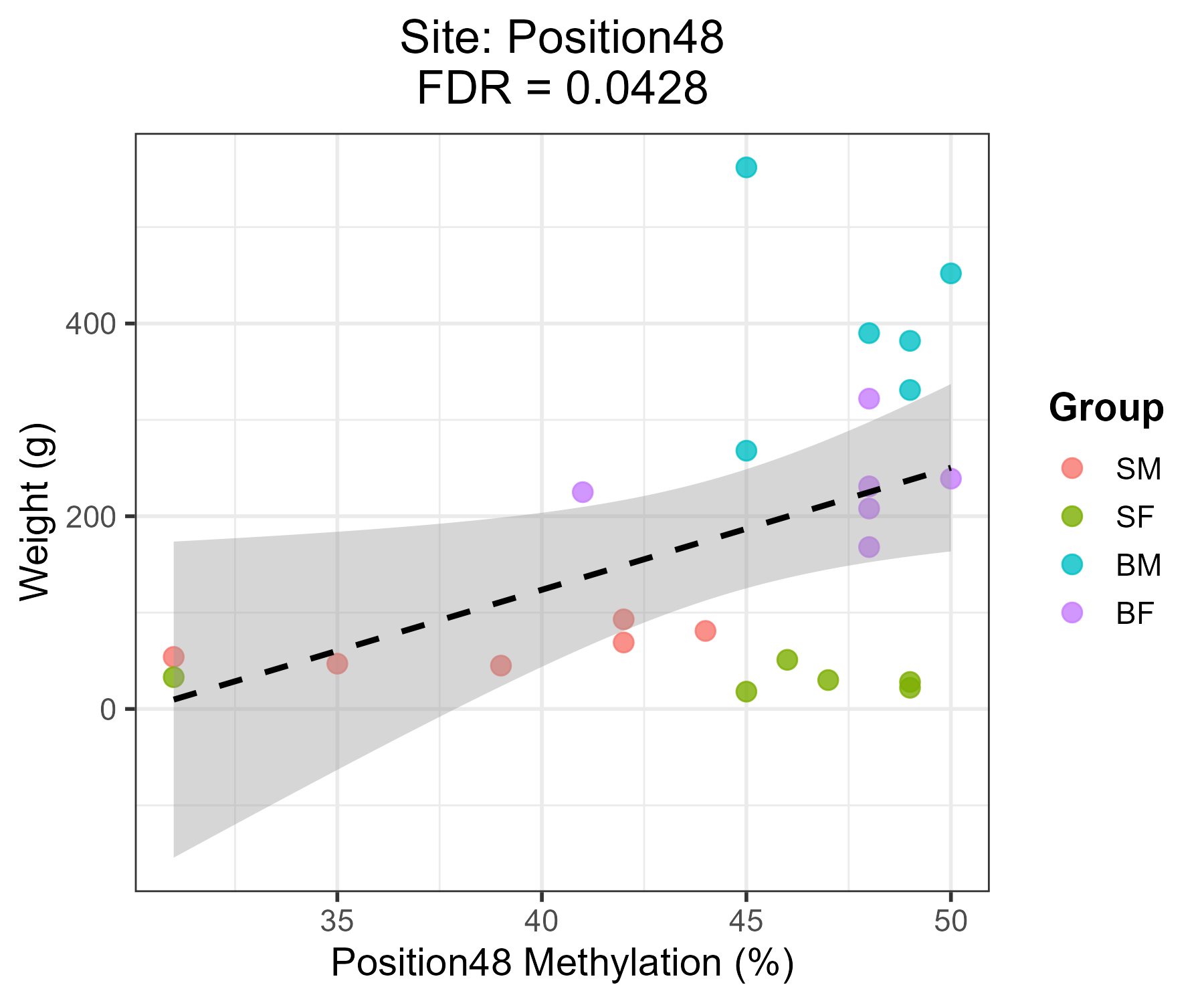

Supplement: Supplementary file 2 [file DataSheet1.zip › Regression_Plus_Strand/Position48_regression.tiff]

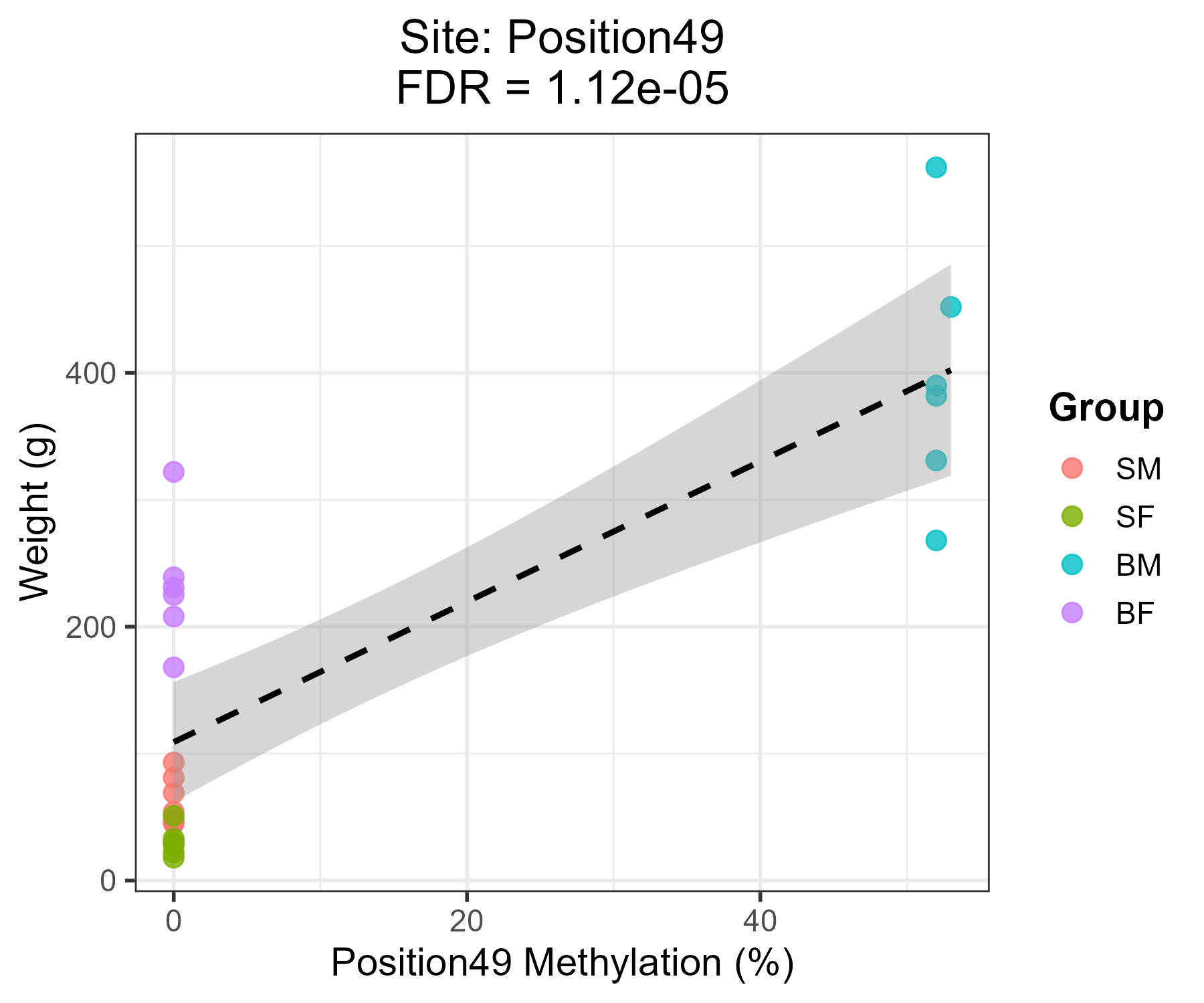

Supplement: Supplementary file 2 [file DataSheet1.zip › Regression_Plus_Strand/Position49_regression.tiff]

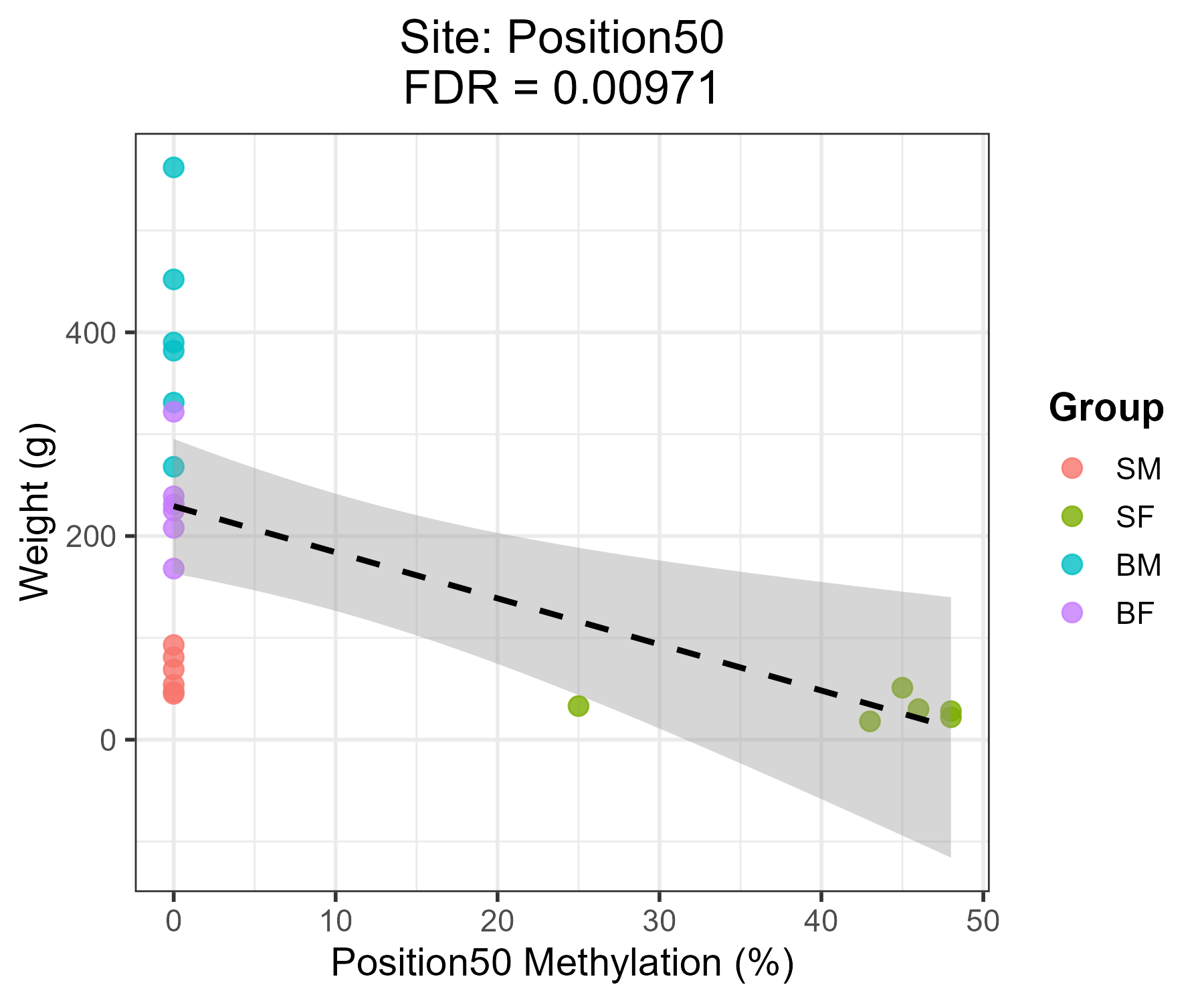

Supplement: Supplementary file 2 [file DataSheet1.zip › Regression_Plus_Strand/Position50_regression.tiff]

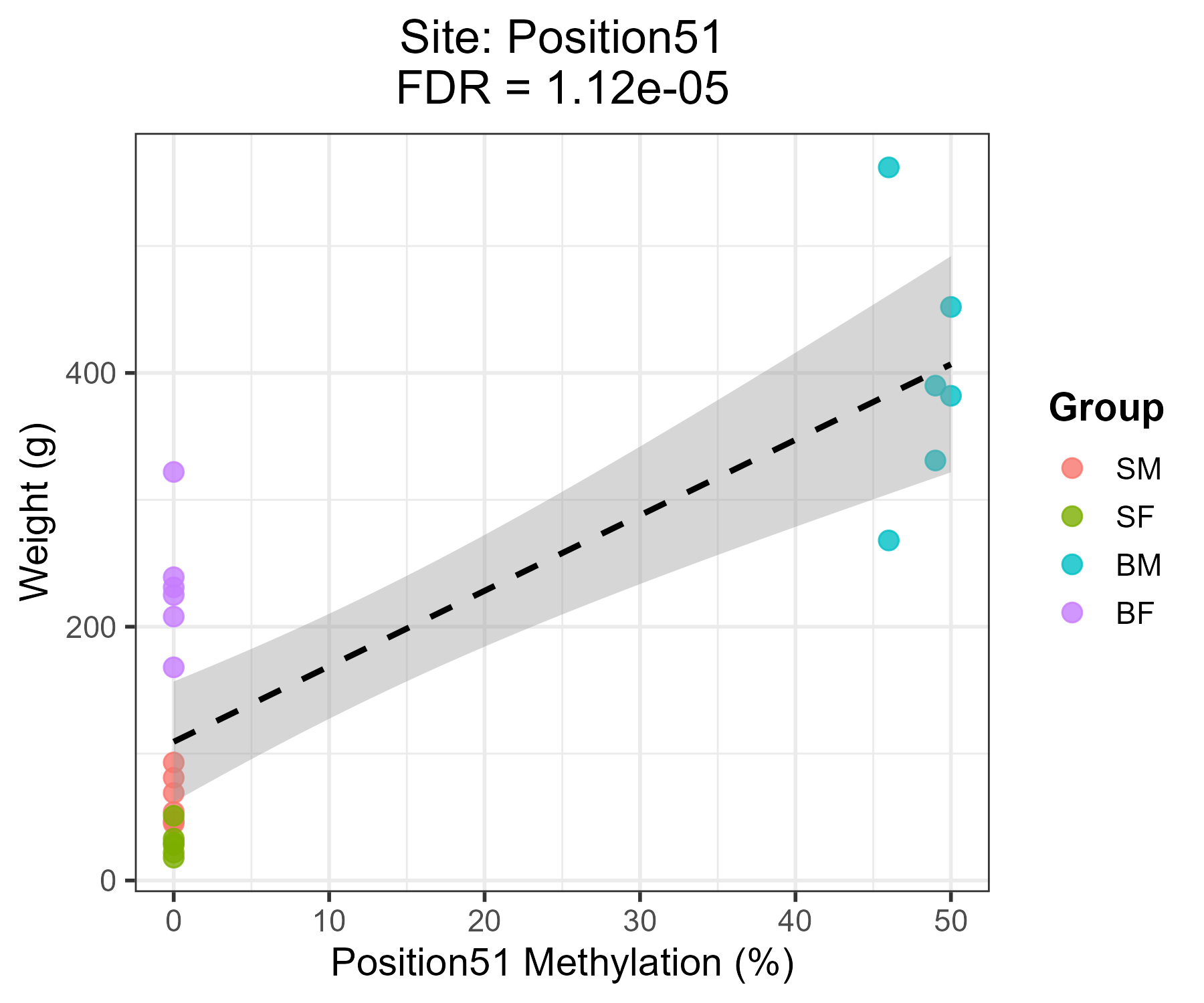

Supplement: Supplementary file 2 [file DataSheet1.zip › Regression_Plus_Strand/Position51_regression.tiff]

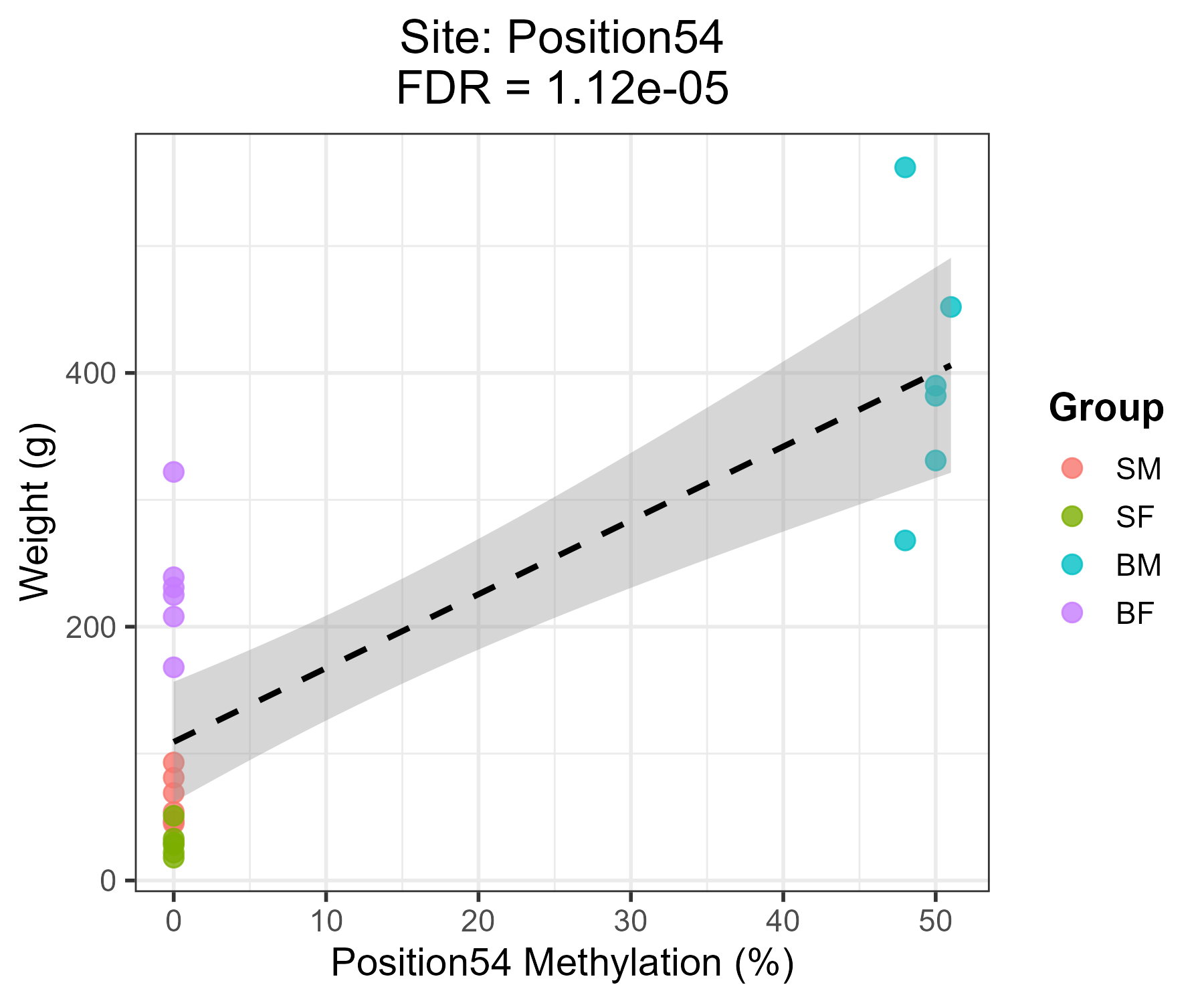

Supplement: Supplementary file 2 [file DataSheet1.zip › Regression_Plus_Strand/Position54_regression.tiff]

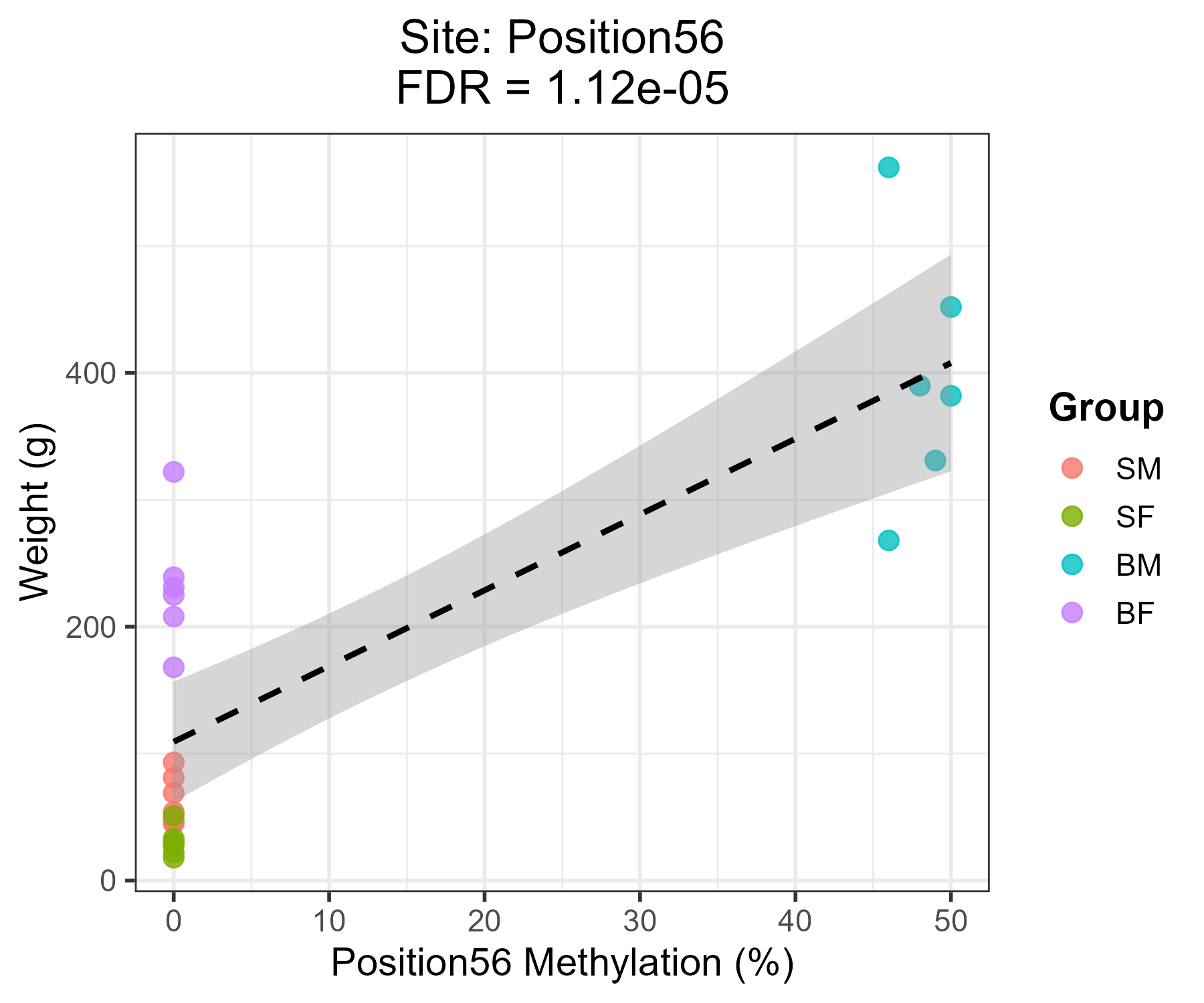

Supplement: Supplementary file 2 [file DataSheet1.zip › Regression_Plus_Strand/Position56_regression.tiff]

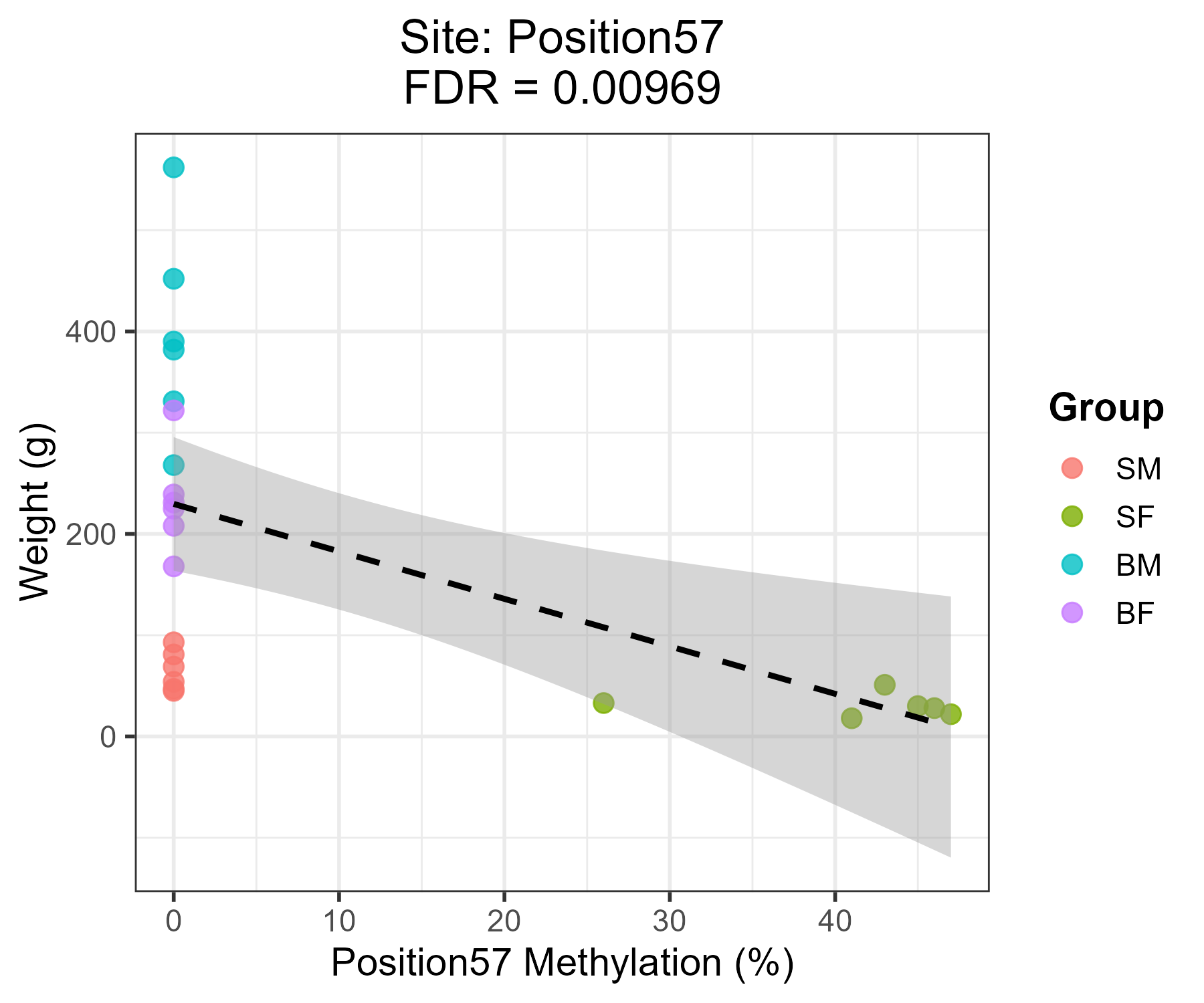

Supplement: Supplementary file 2 [file DataSheet1.zip › Regression_Plus_Strand/Position57_regression.tiff]

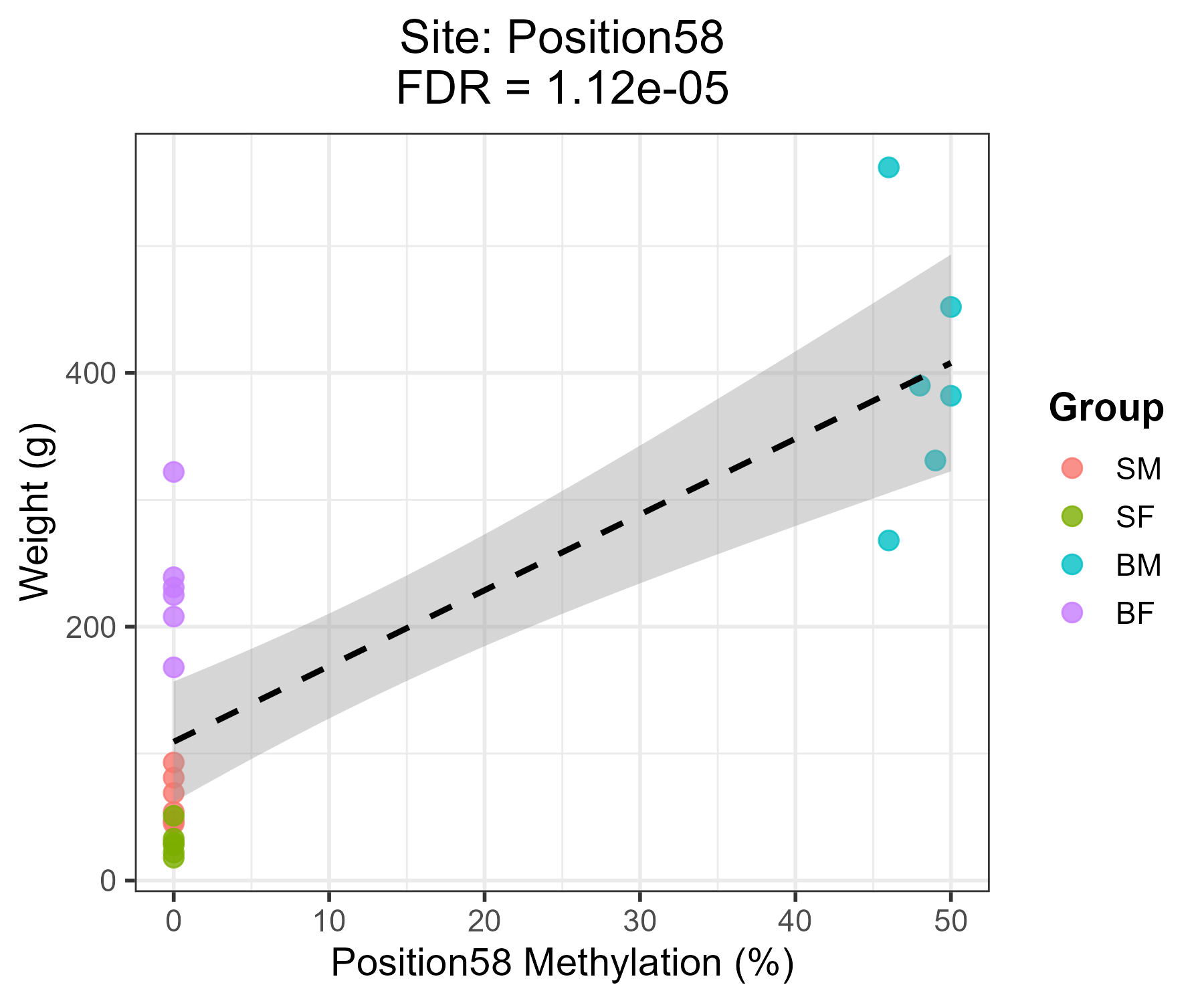

Supplement: Supplementary file 2 [file DataSheet1.zip › Regression_Plus_Strand/Position58_regression.tiff]

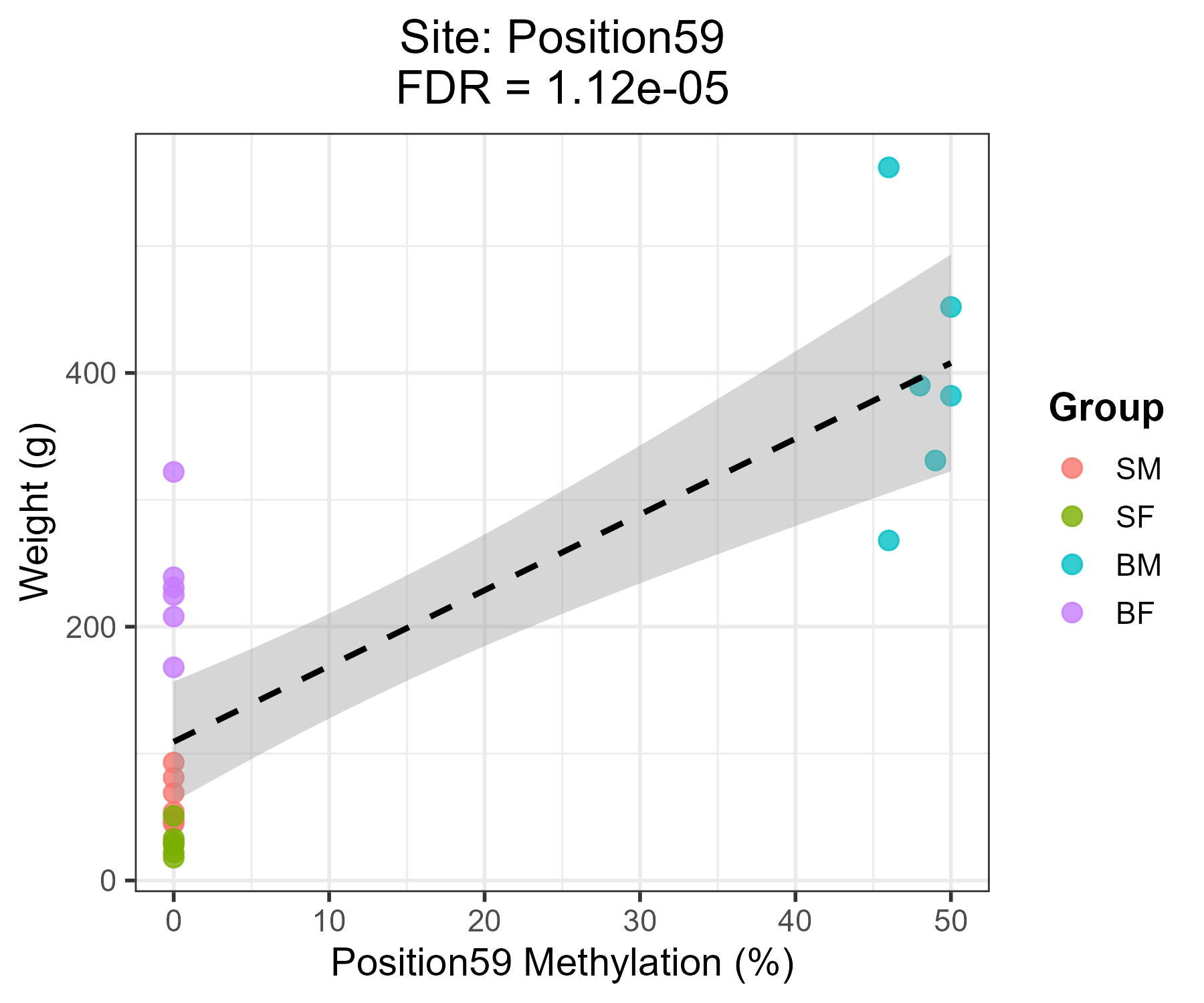

Supplement: Supplementary file 2 [file DataSheet1.zip › Regression_Plus_Strand/Position59_regression.tiff]

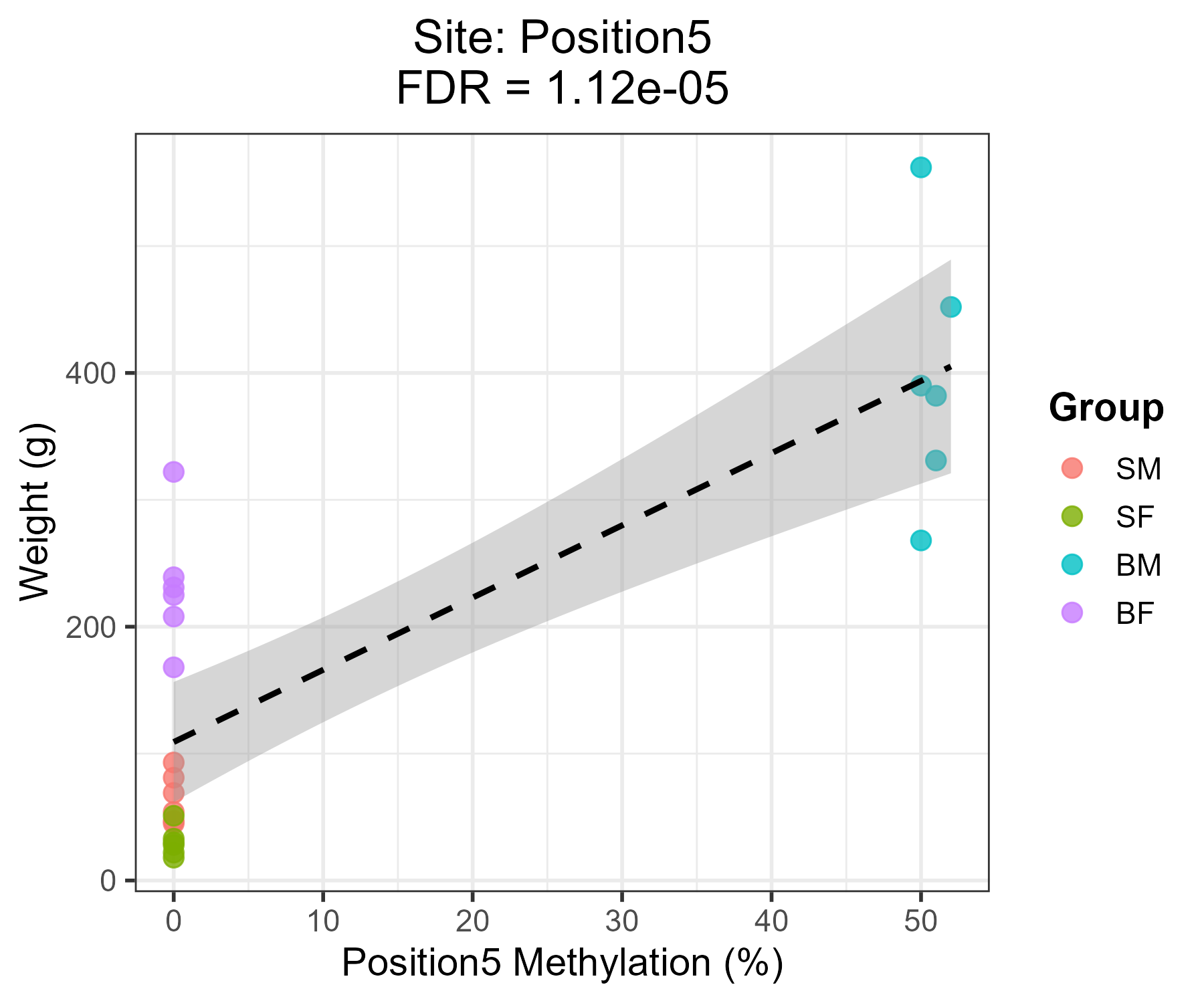

Supplement: Supplementary file 2 [file DataSheet1.zip › Regression_Plus_Strand/Position5_regression.tiff]

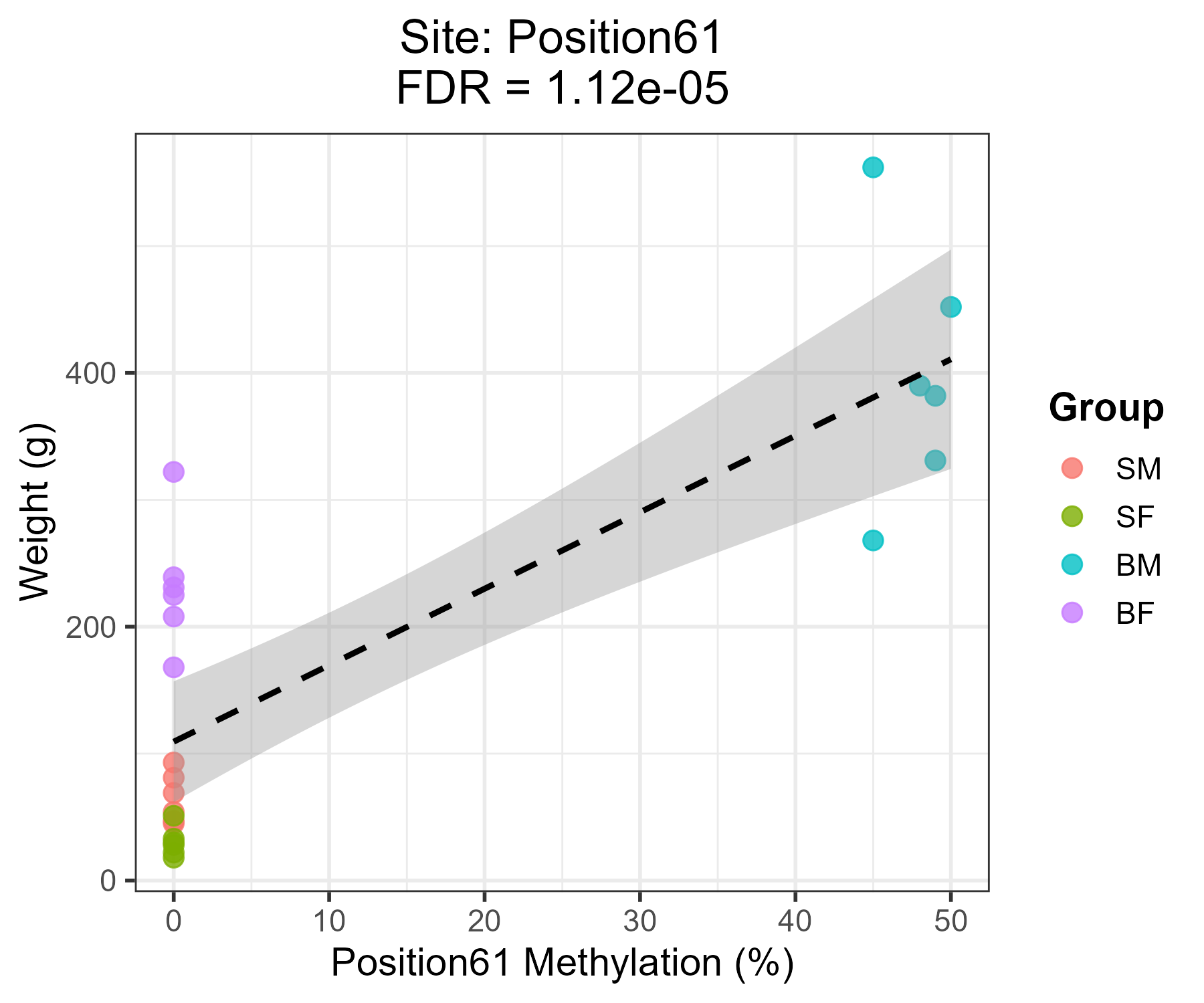

Supplement: Supplementary file 2 [file DataSheet1.zip › Regression_Plus_Strand/Position61_regression.tiff]

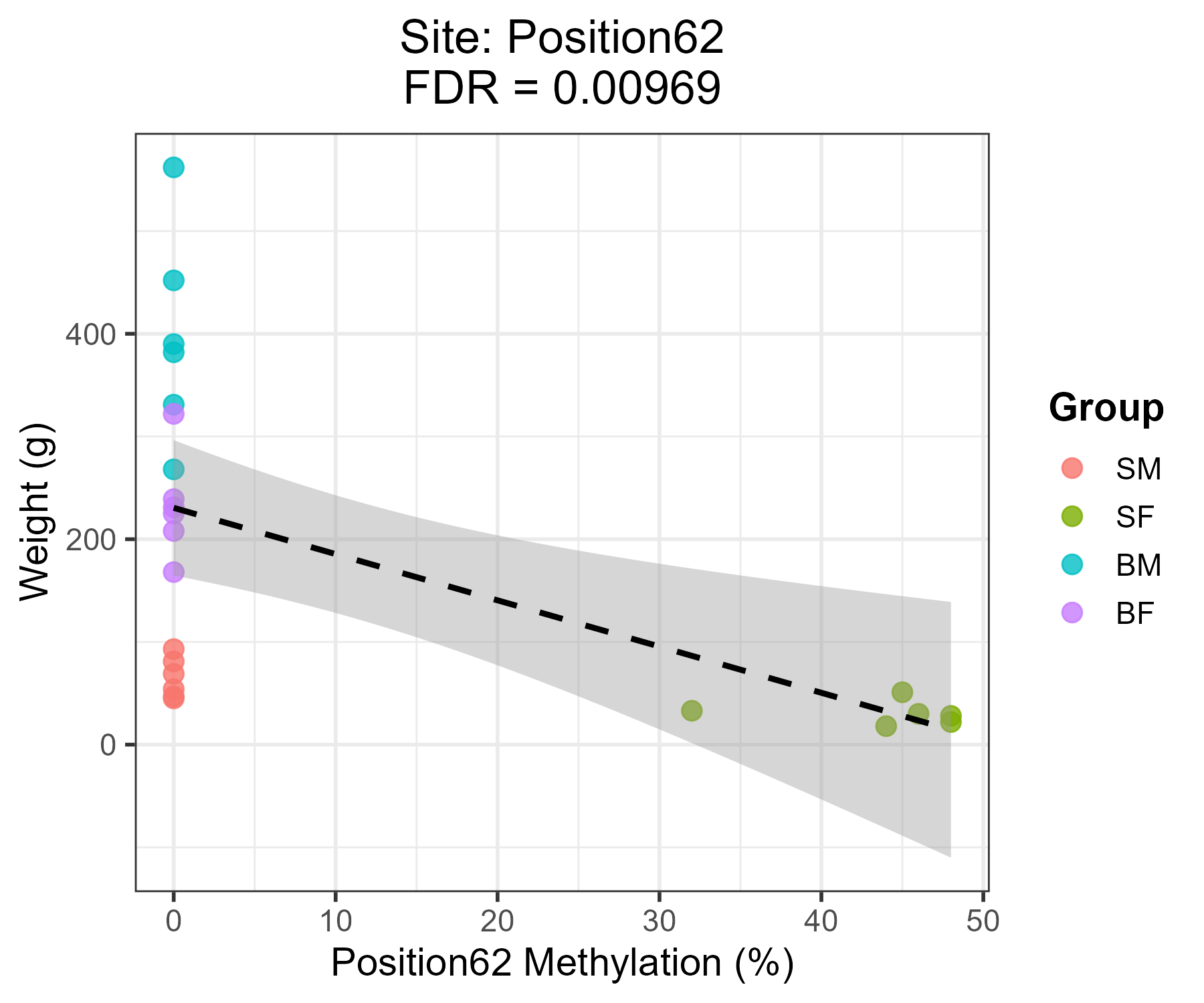

Supplement: Supplementary file 2 [file DataSheet1.zip › Regression_Plus_Strand/Position62_regression.tiff]

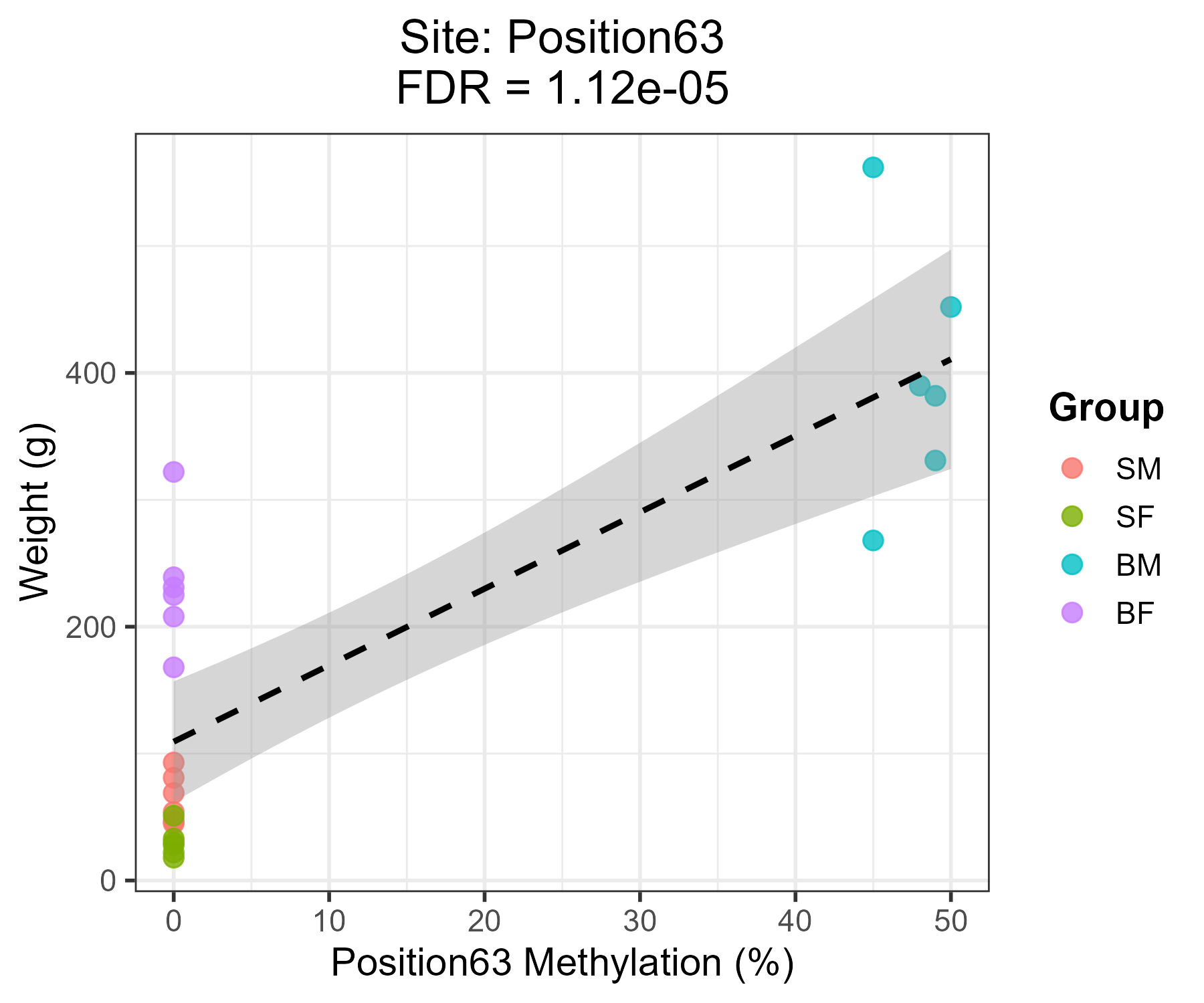

Supplement: Supplementary file 2 [file DataSheet1.zip › Regression_Plus_Strand/Position63_regression.tiff]

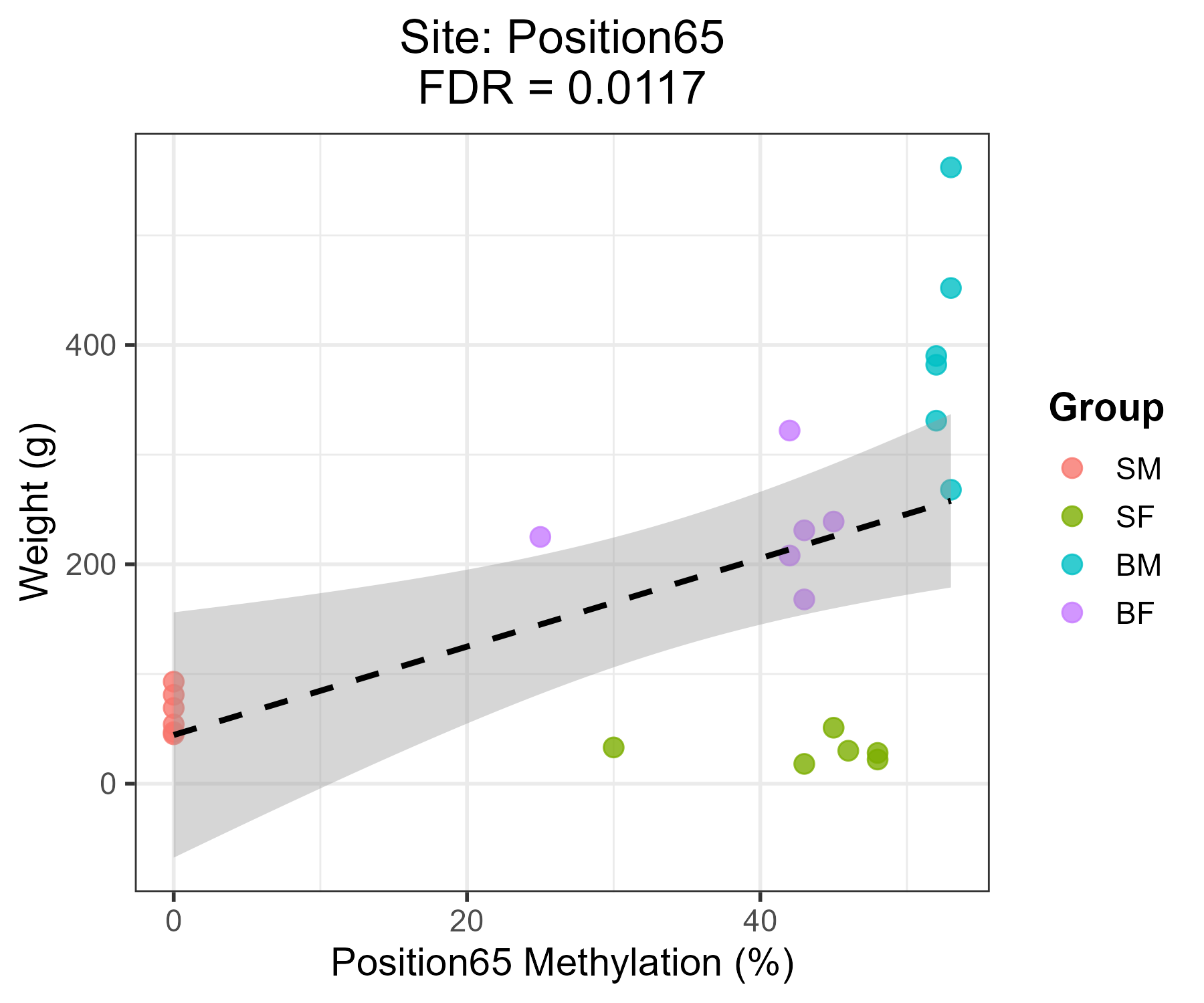

Supplement: Supplementary file 2 [file DataSheet1.zip › Regression_Plus_Strand/Position65_regression.tiff]

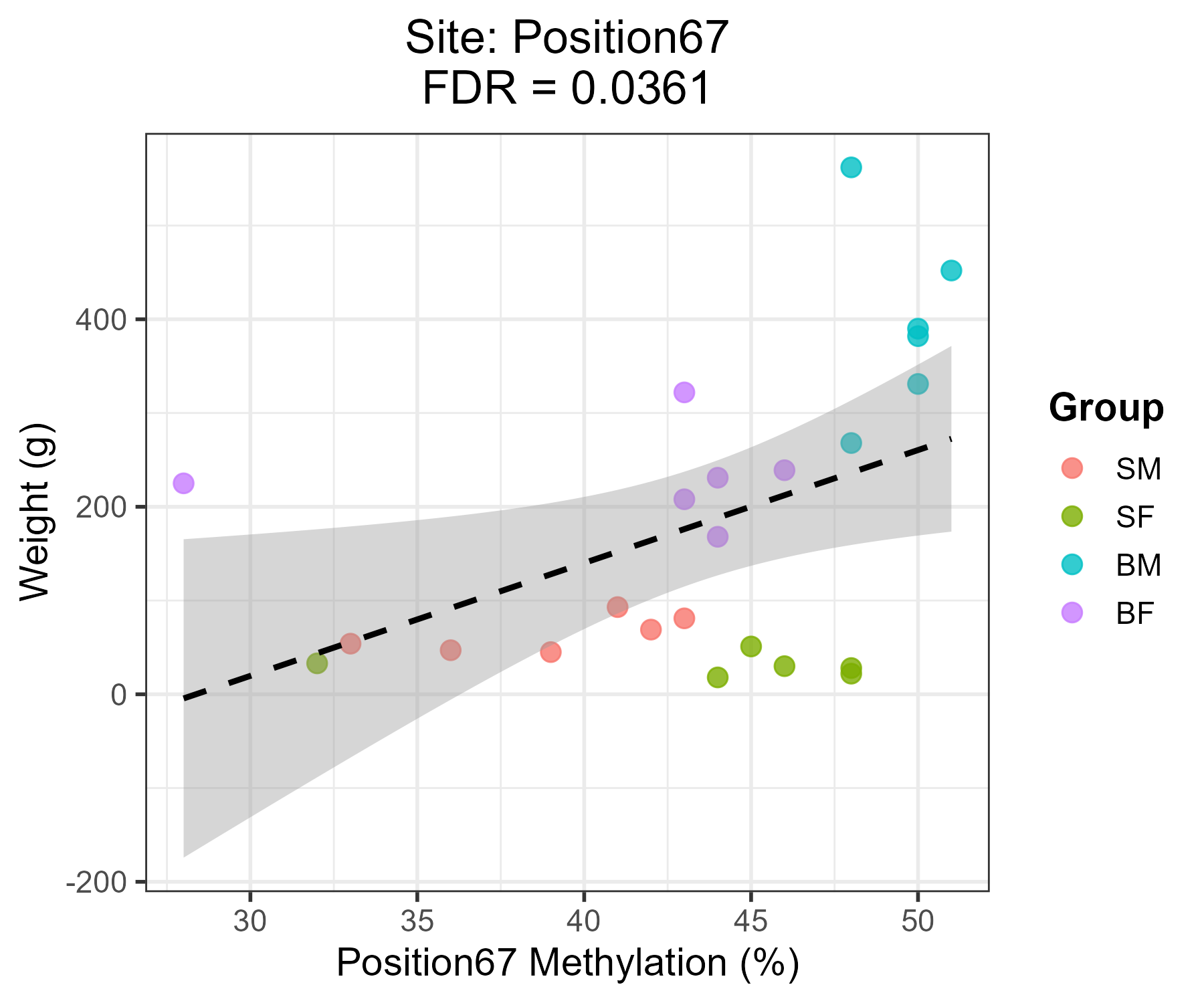

Supplement: Supplementary file 2 [file DataSheet1.zip › Regression_Plus_Strand/Position67_regression.tiff]

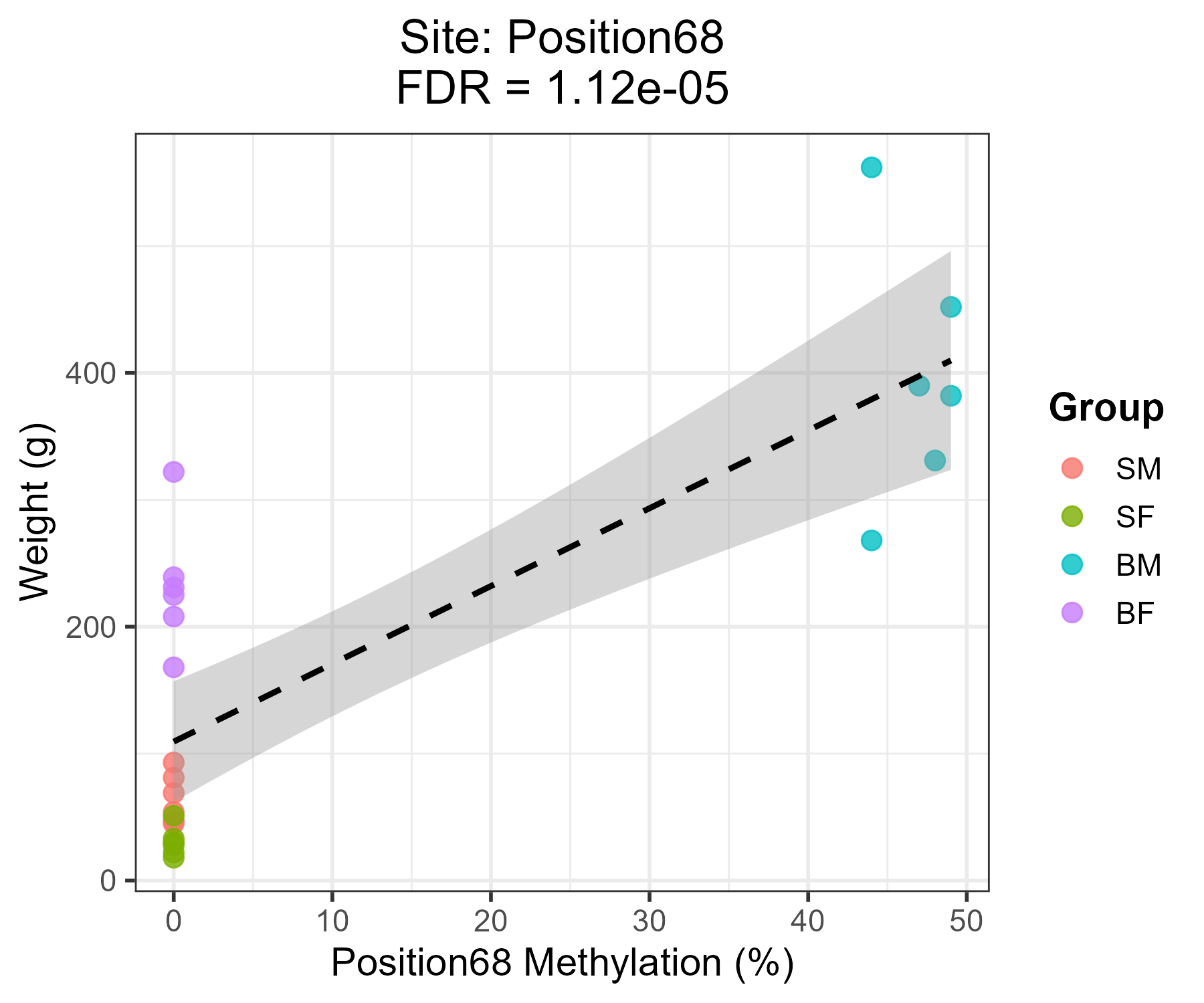

Supplement: Supplementary file 2 [file DataSheet1.zip › Regression_Plus_Strand/Position68_regression.tiff]

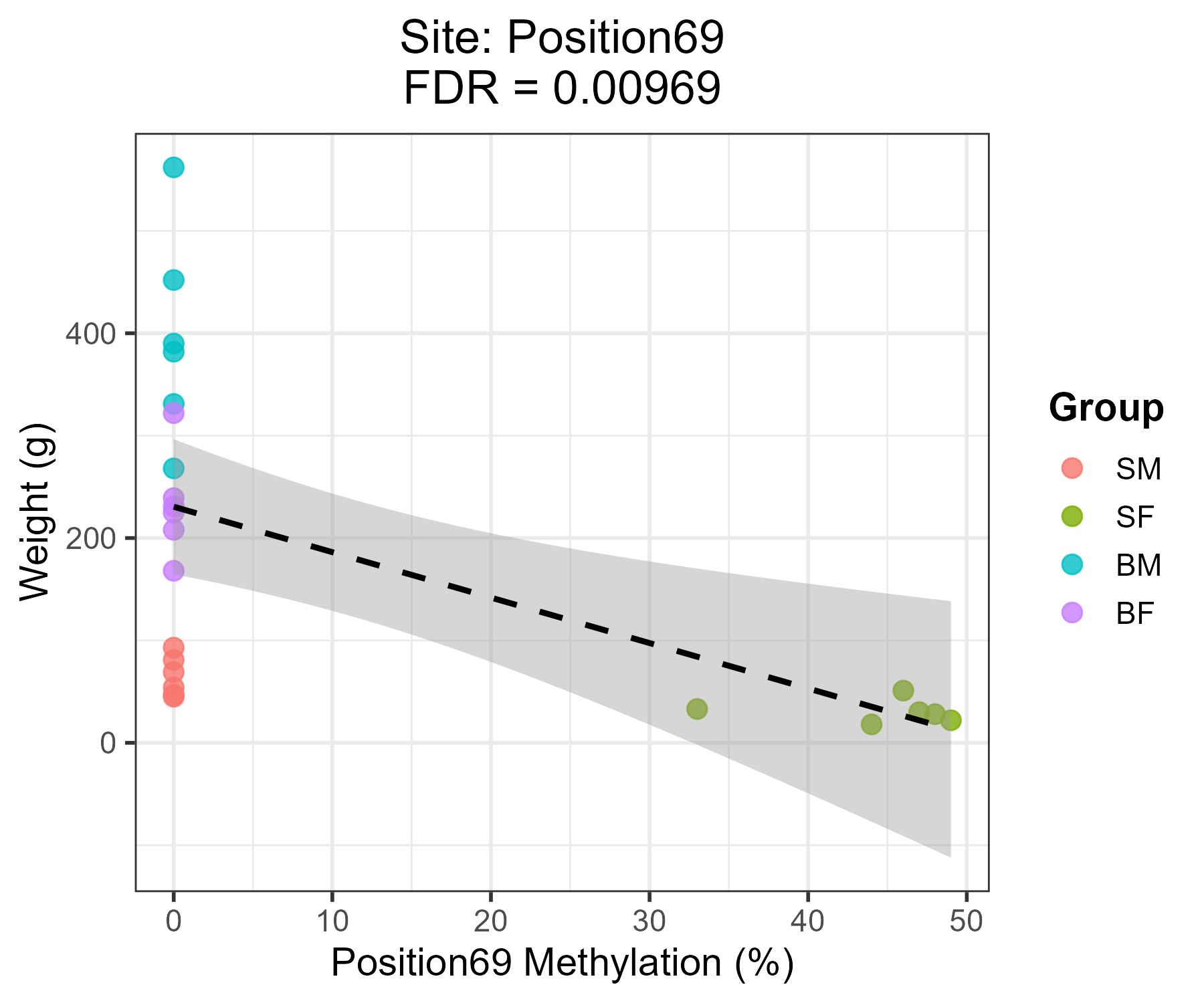

Supplement: Supplementary file 2 [file DataSheet1.zip › Regression_Plus_Strand/Position69_regression.tiff]

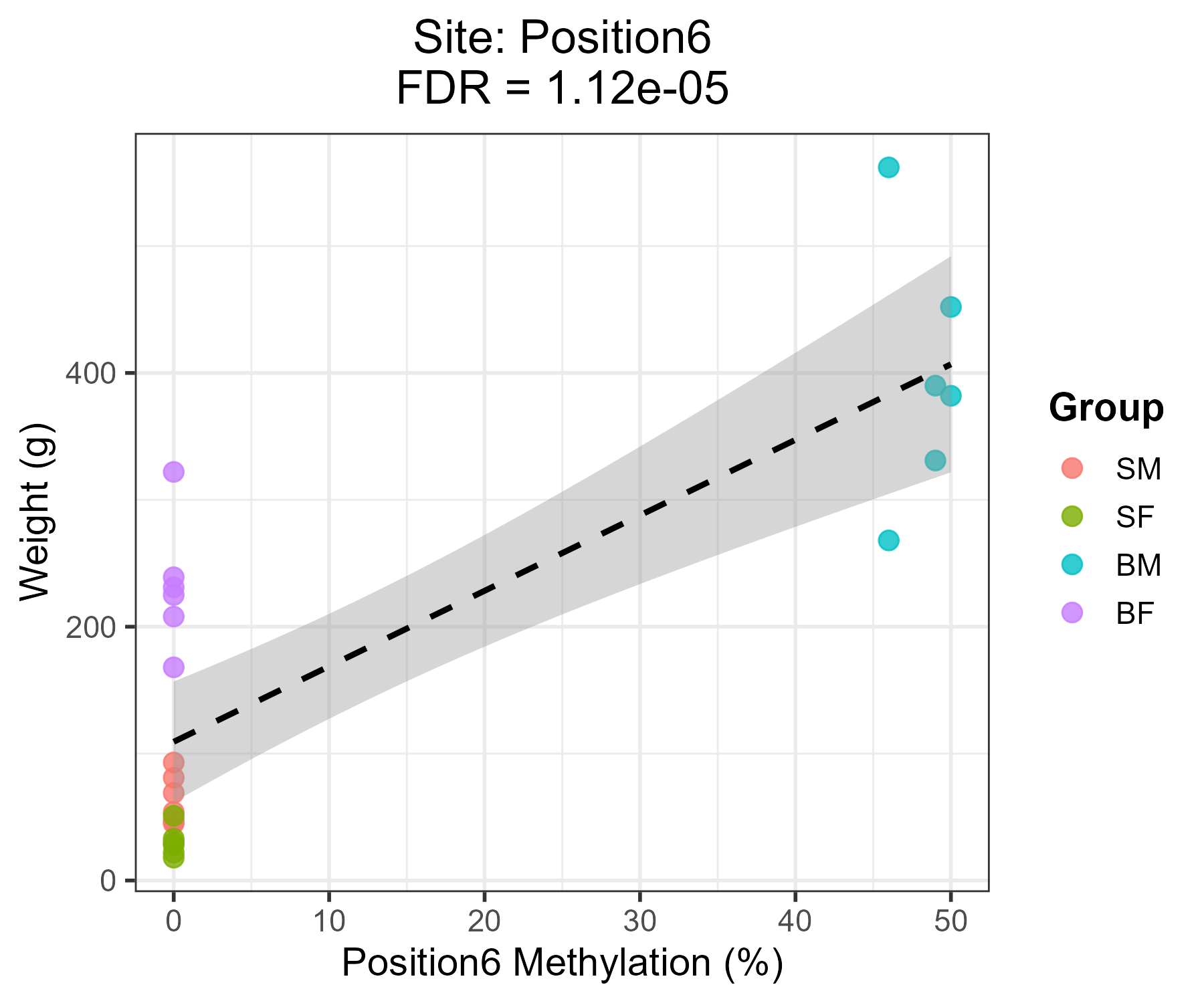

Supplement: Supplementary file 2 [file DataSheet1.zip › Regression_Plus_Strand/Position6_regression.tiff]

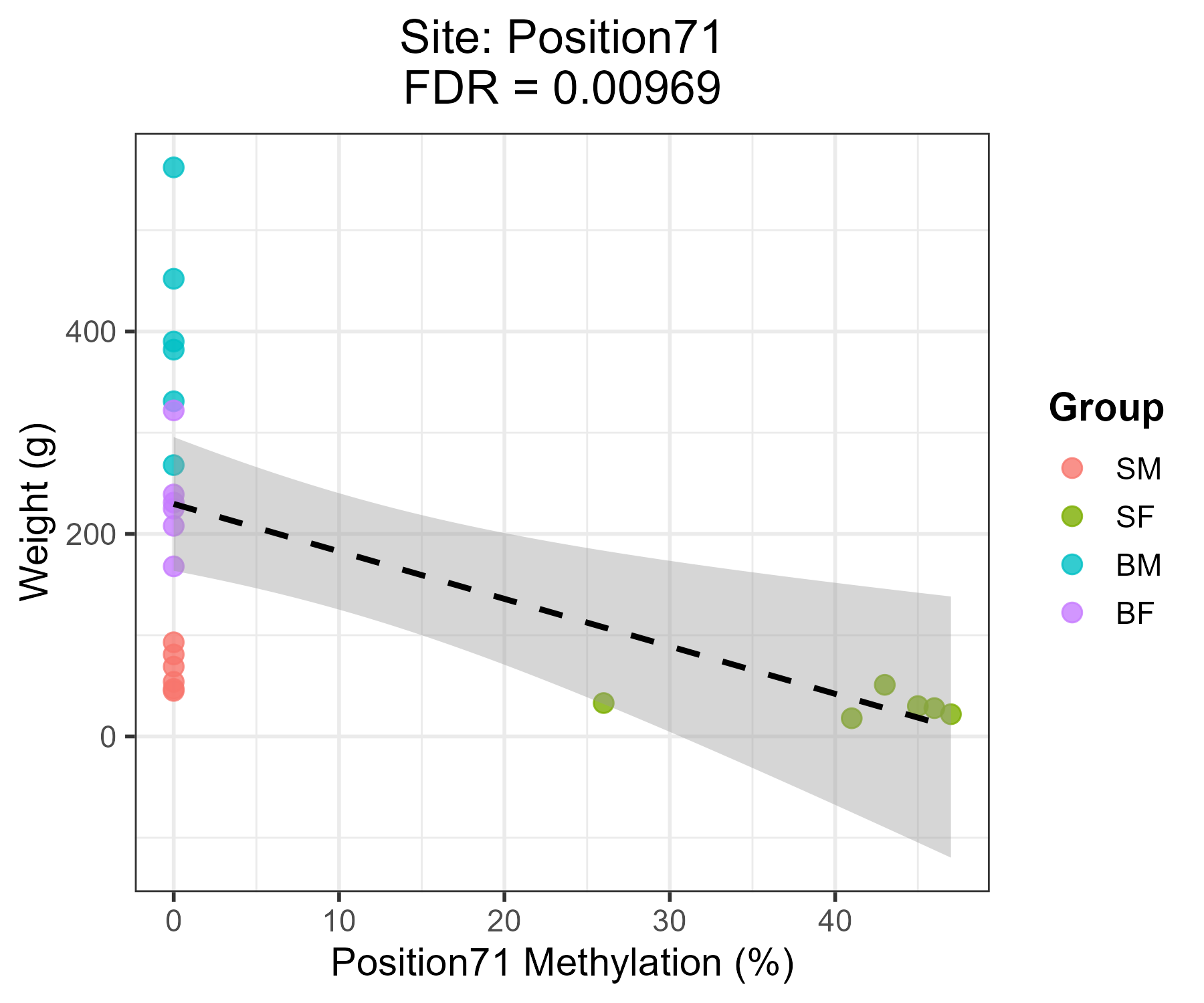

Supplement: Supplementary file 2 [file DataSheet1.zip › Regression_Plus_Strand/Position71_regression.tiff]

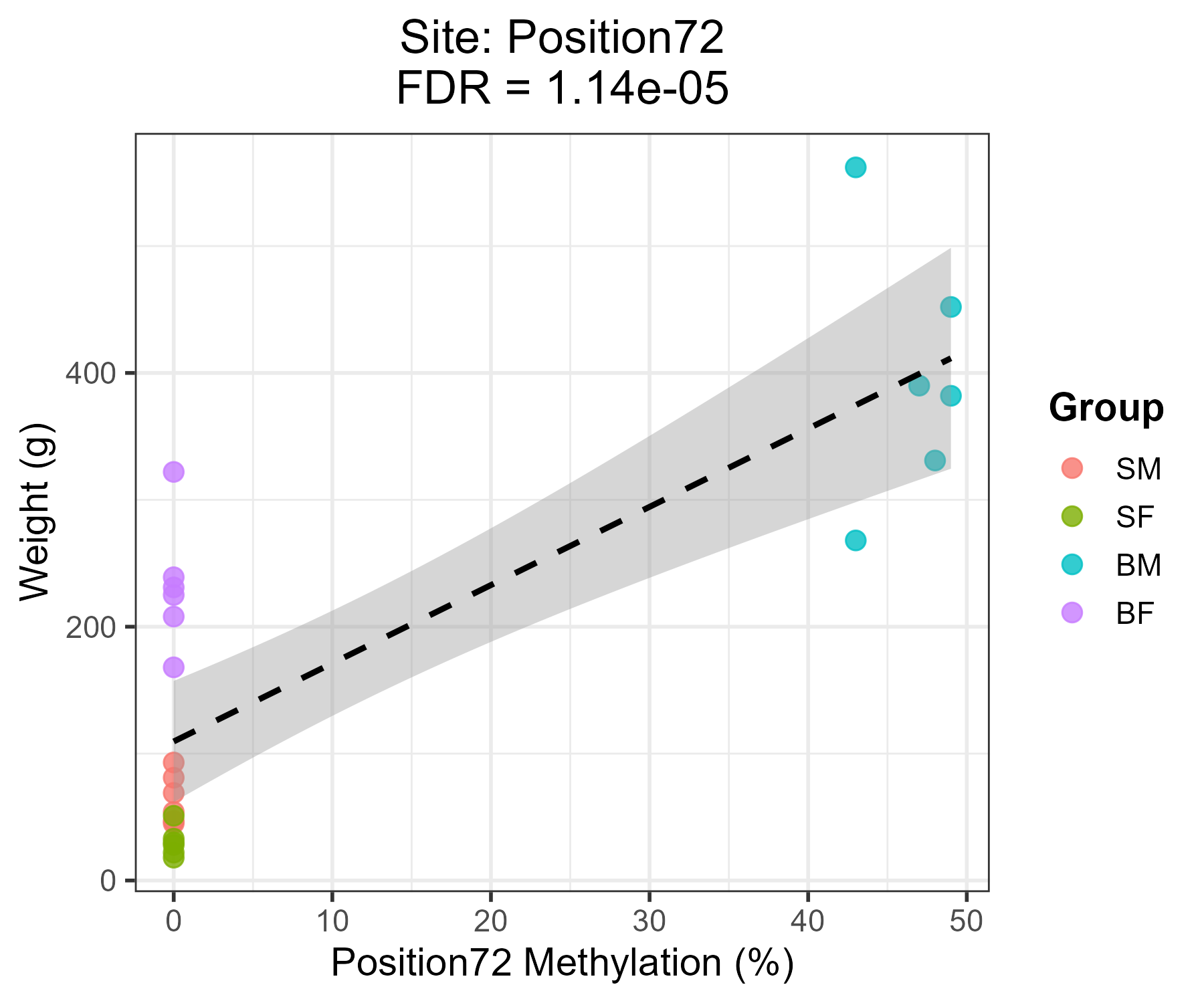

Supplement: Supplementary file 2 [file DataSheet1.zip › Regression_Plus_Strand/Position72_regression.tiff]

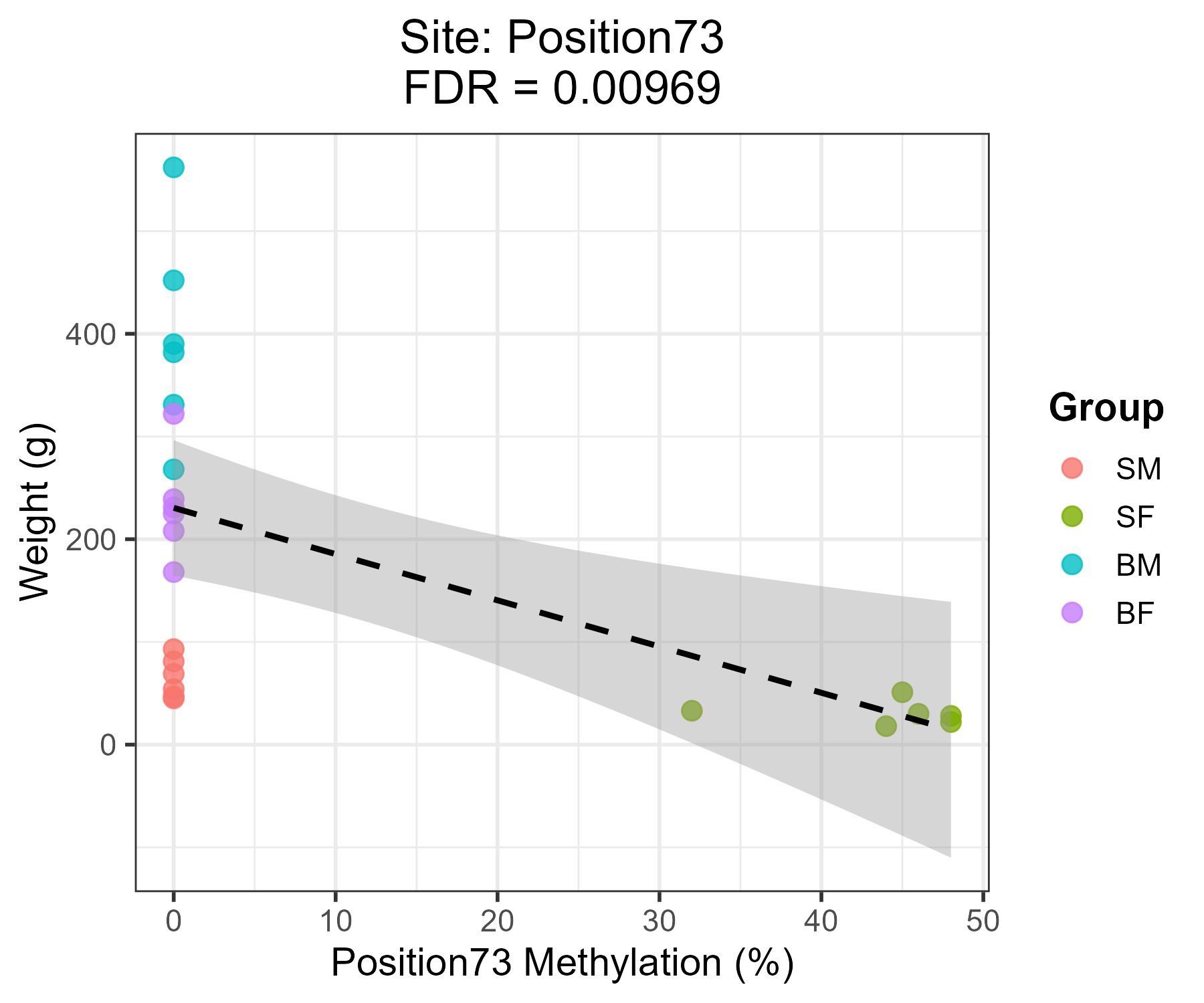

Supplement: Supplementary file 2 [file DataSheet1.zip › Regression_Plus_Strand/Position73_regression.tiff]

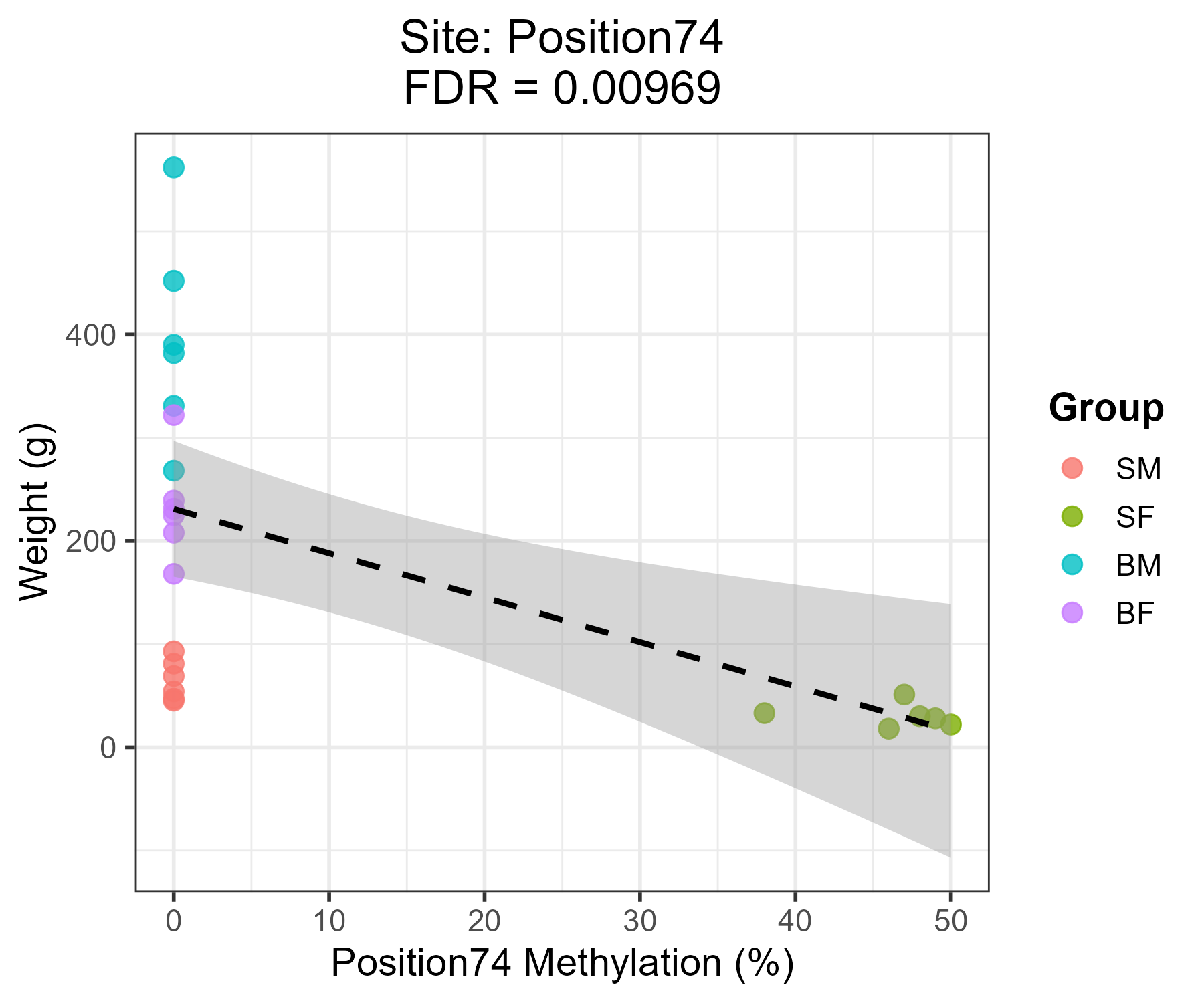

Supplement: Supplementary file 2 [file DataSheet1.zip › Regression_Plus_Strand/Position74_regression.tiff]

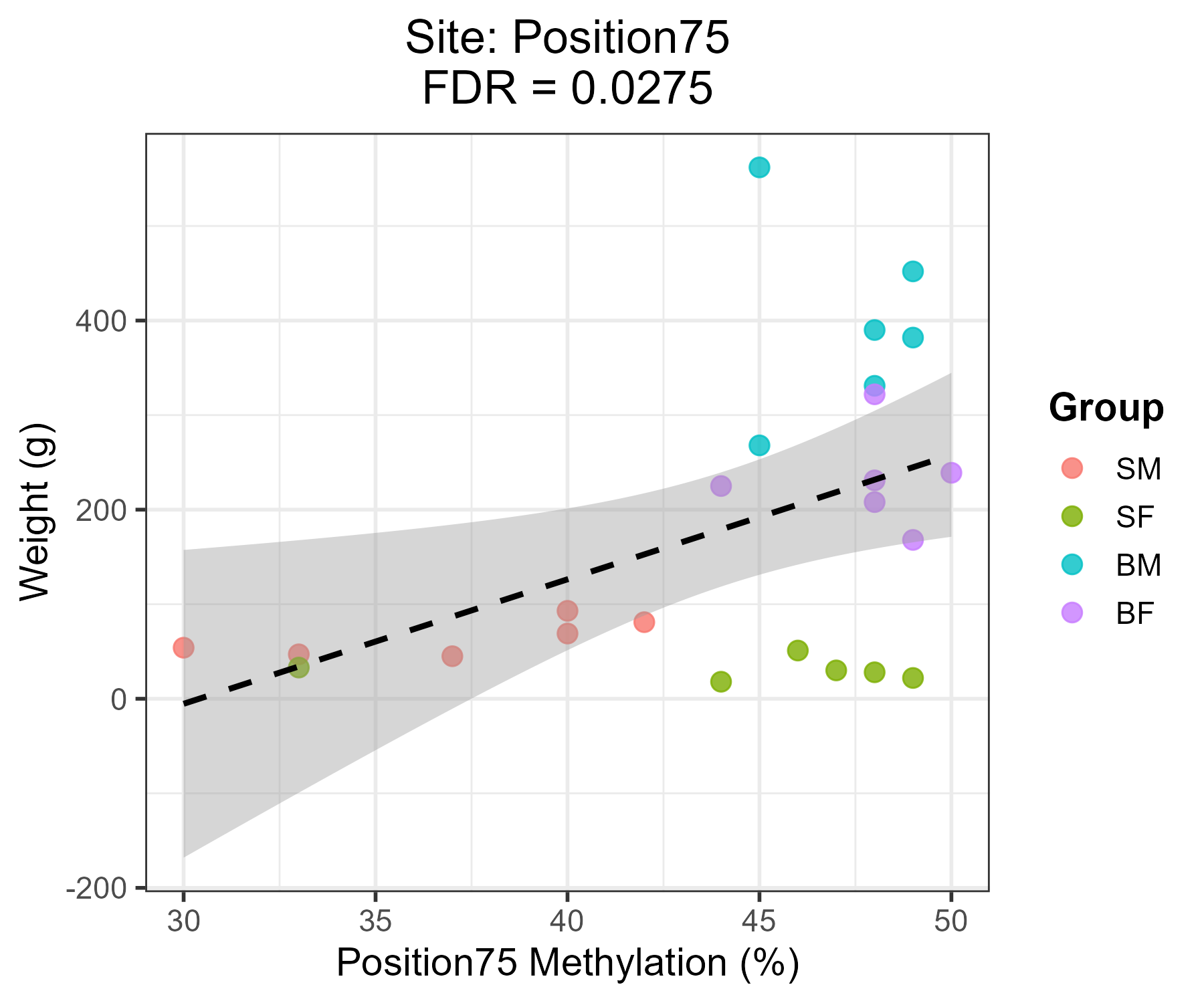

Supplement: Supplementary file 2 [file DataSheet1.zip › Regression_Plus_Strand/Position75_regression.tiff]

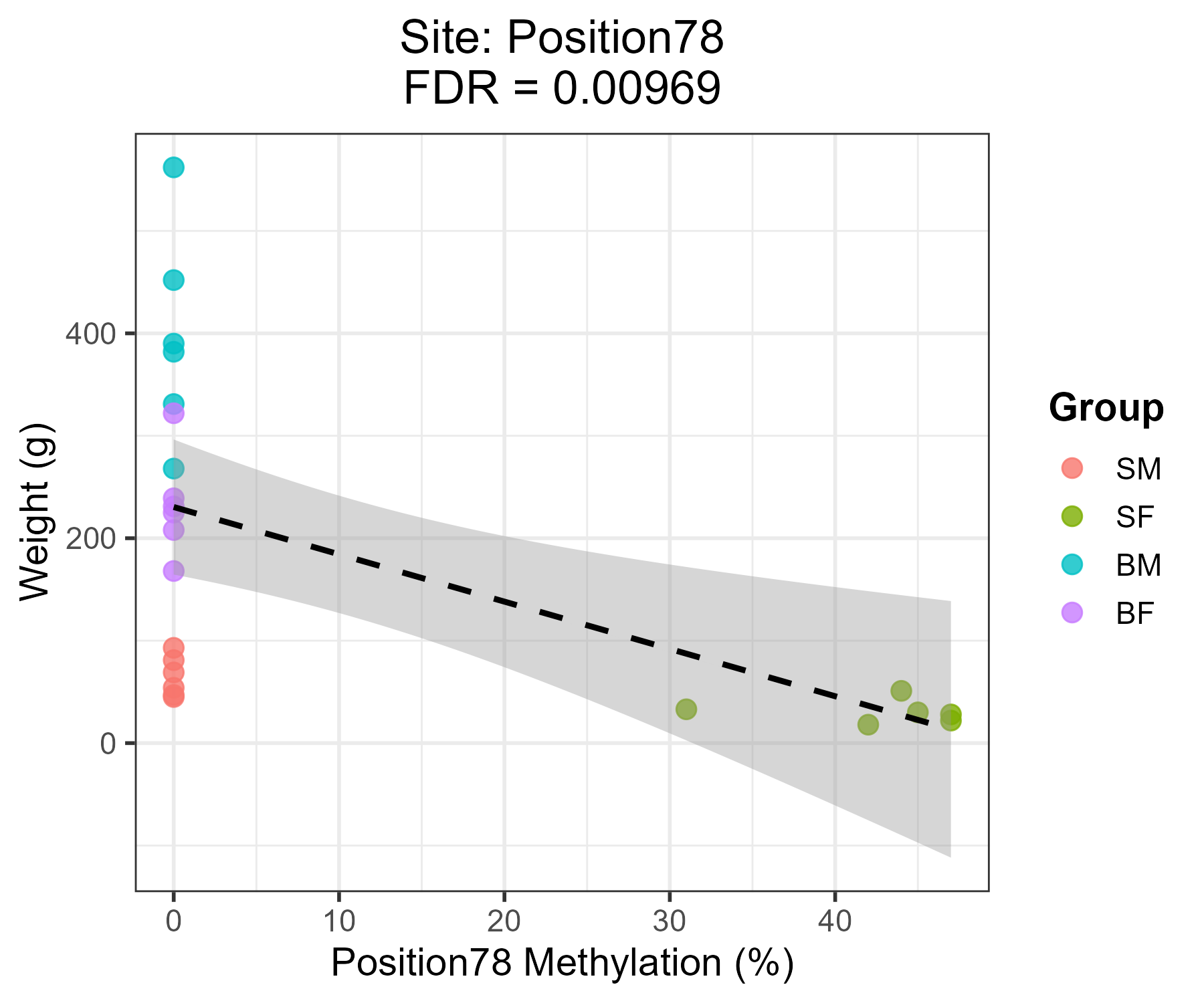

Supplement: Supplementary file 2 [file DataSheet1.zip › Regression_Plus_Strand/Position78_regression.tiff]

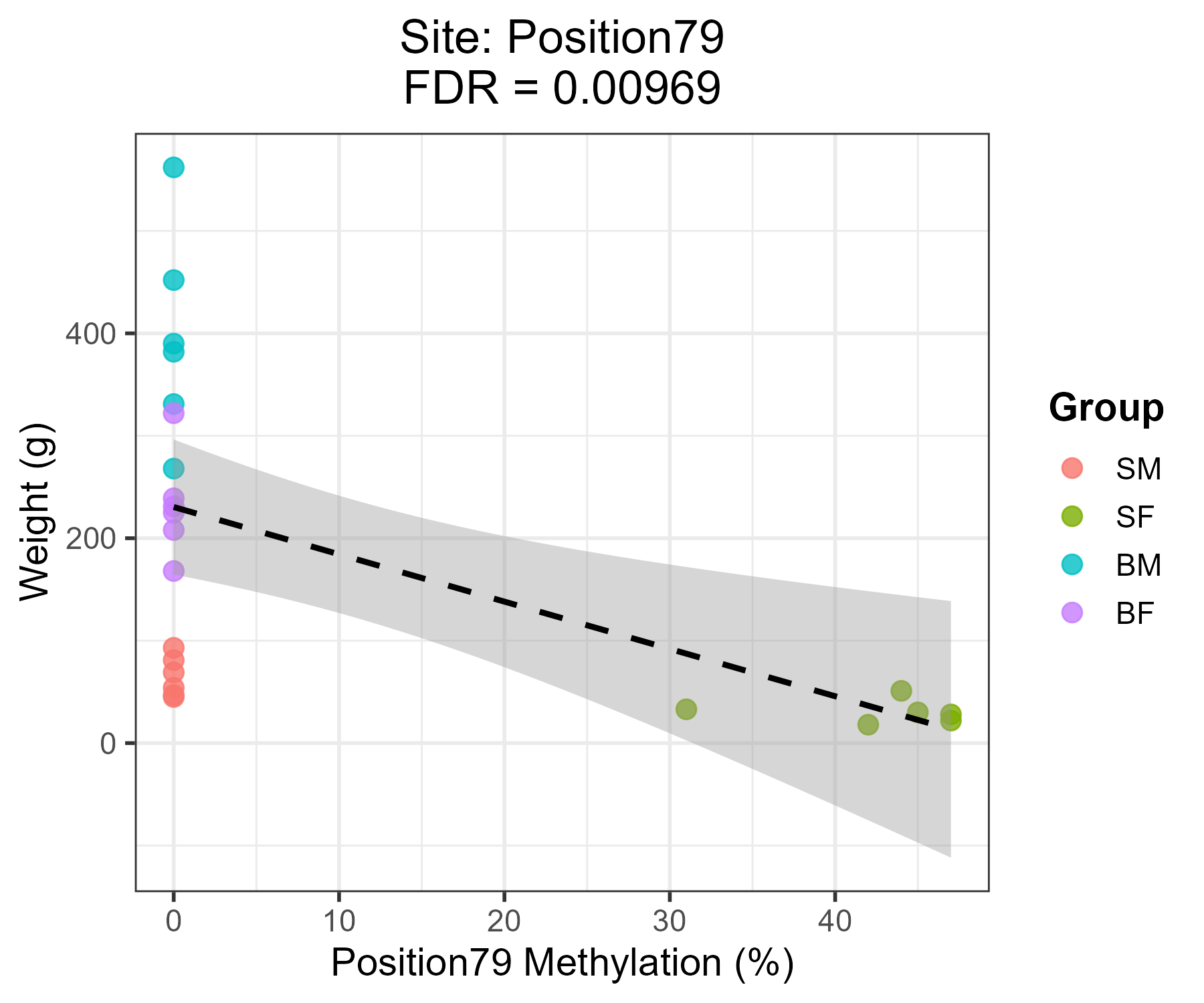

Supplement: Supplementary file 2 [file DataSheet1.zip › Regression_Plus_Strand/Position79_regression.tiff]

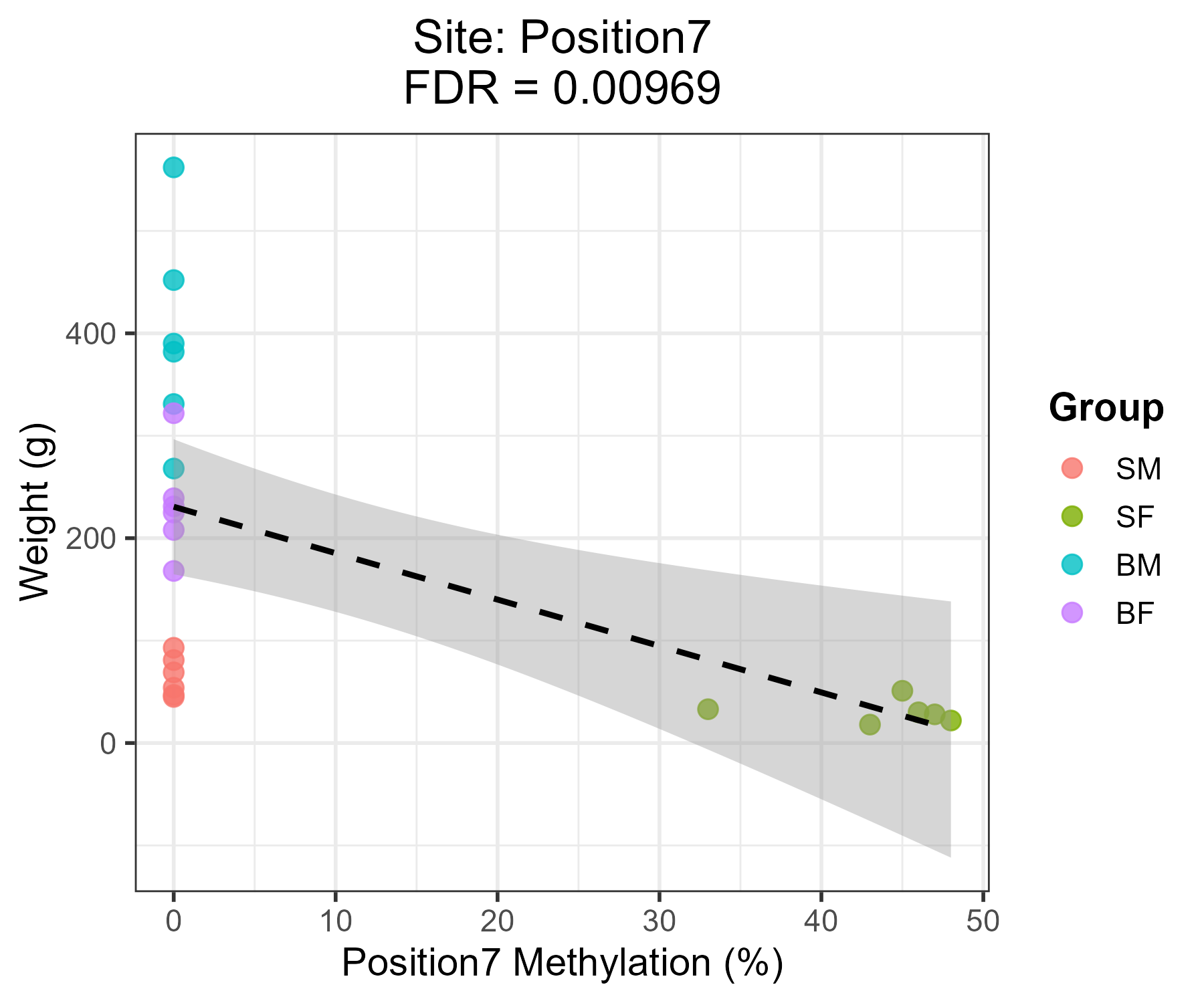

Supplement: Supplementary file 2 [file DataSheet1.zip › Regression_Plus_Strand/Position7_regression.tiff]

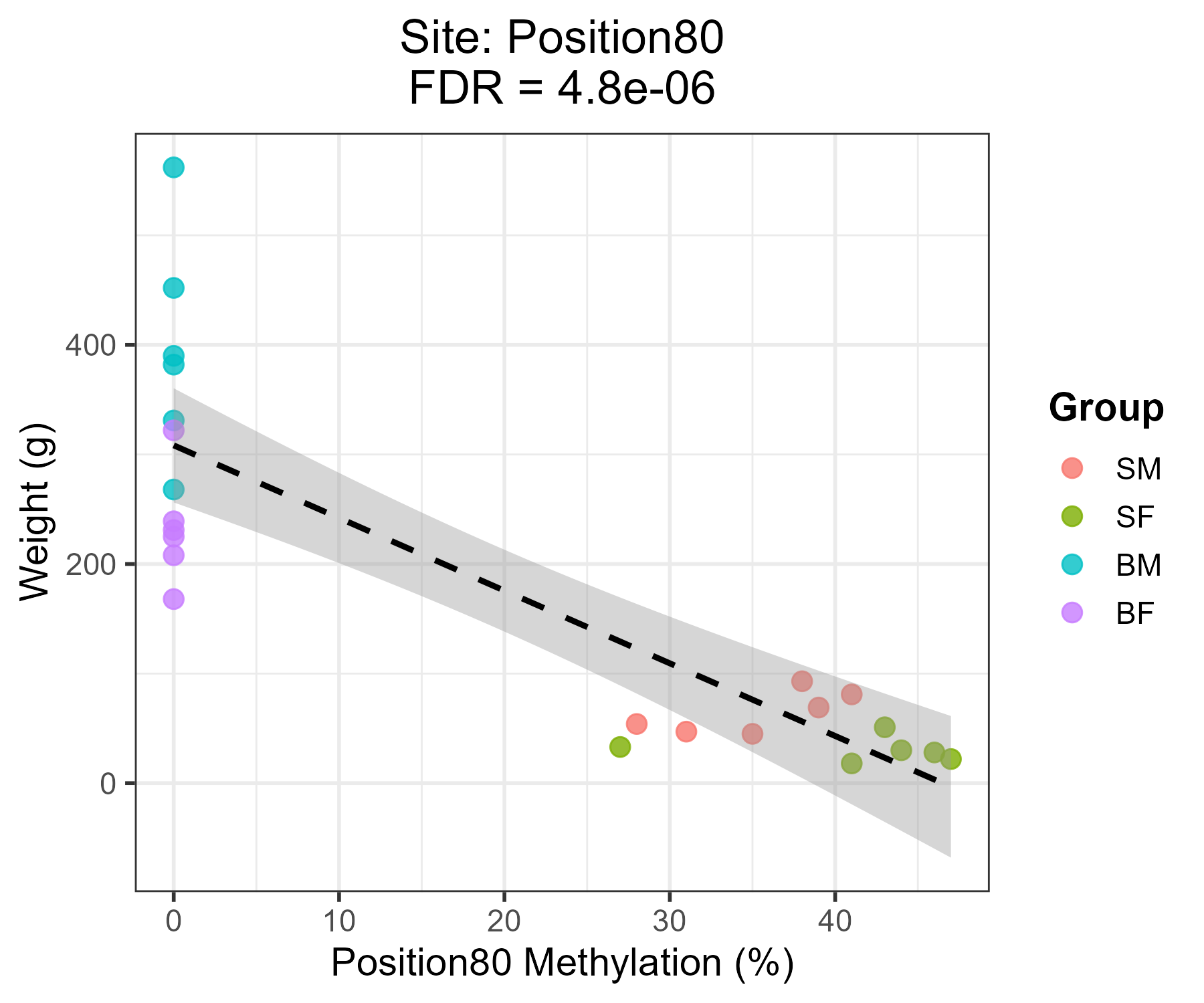

Supplement: Supplementary file 2 [file DataSheet1.zip › Regression_Plus_Strand/Position80_regression.tiff]

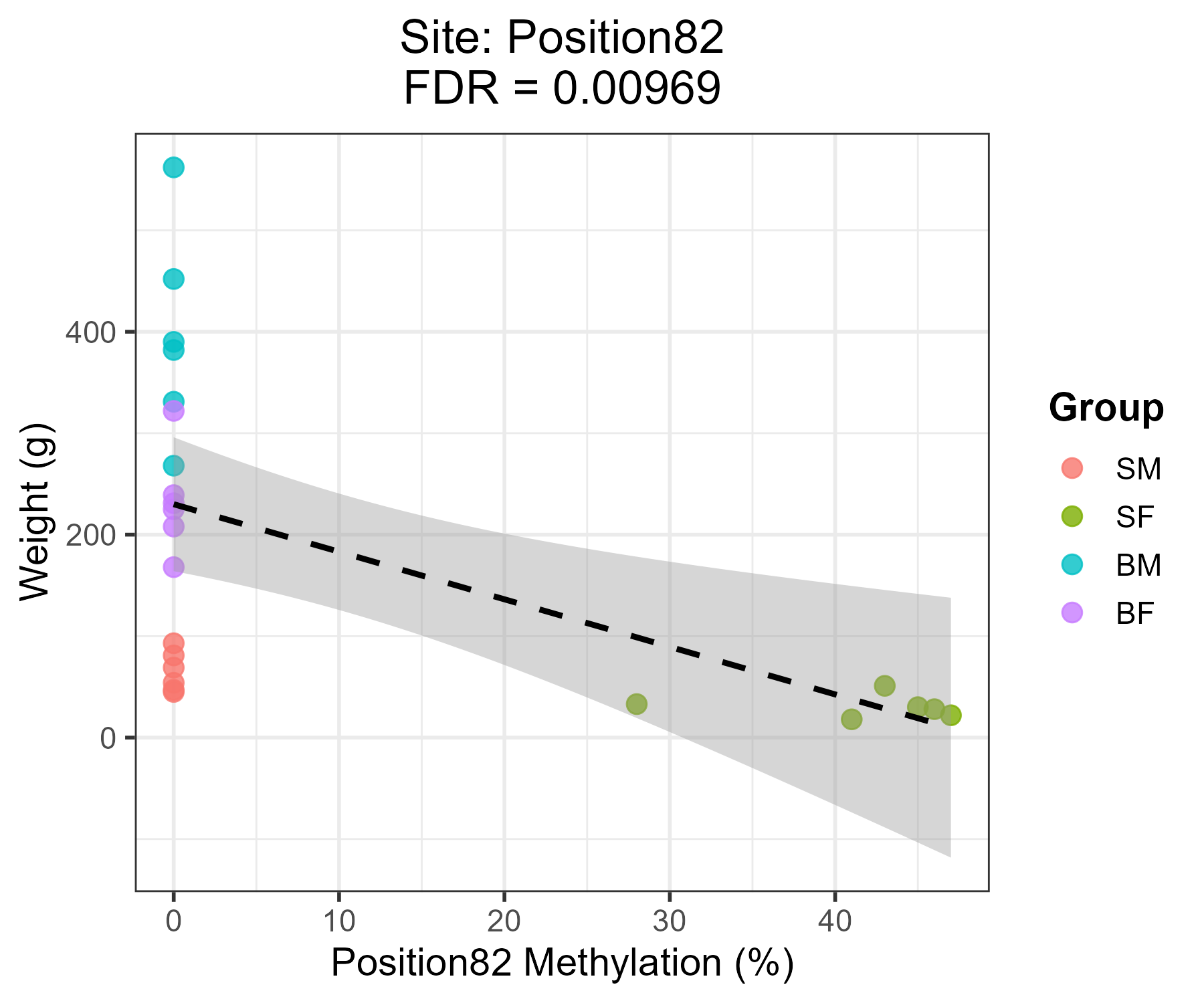

Supplement: Supplementary file 2 [file DataSheet1.zip › Regression_Plus_Strand/Position82_regression.tiff]

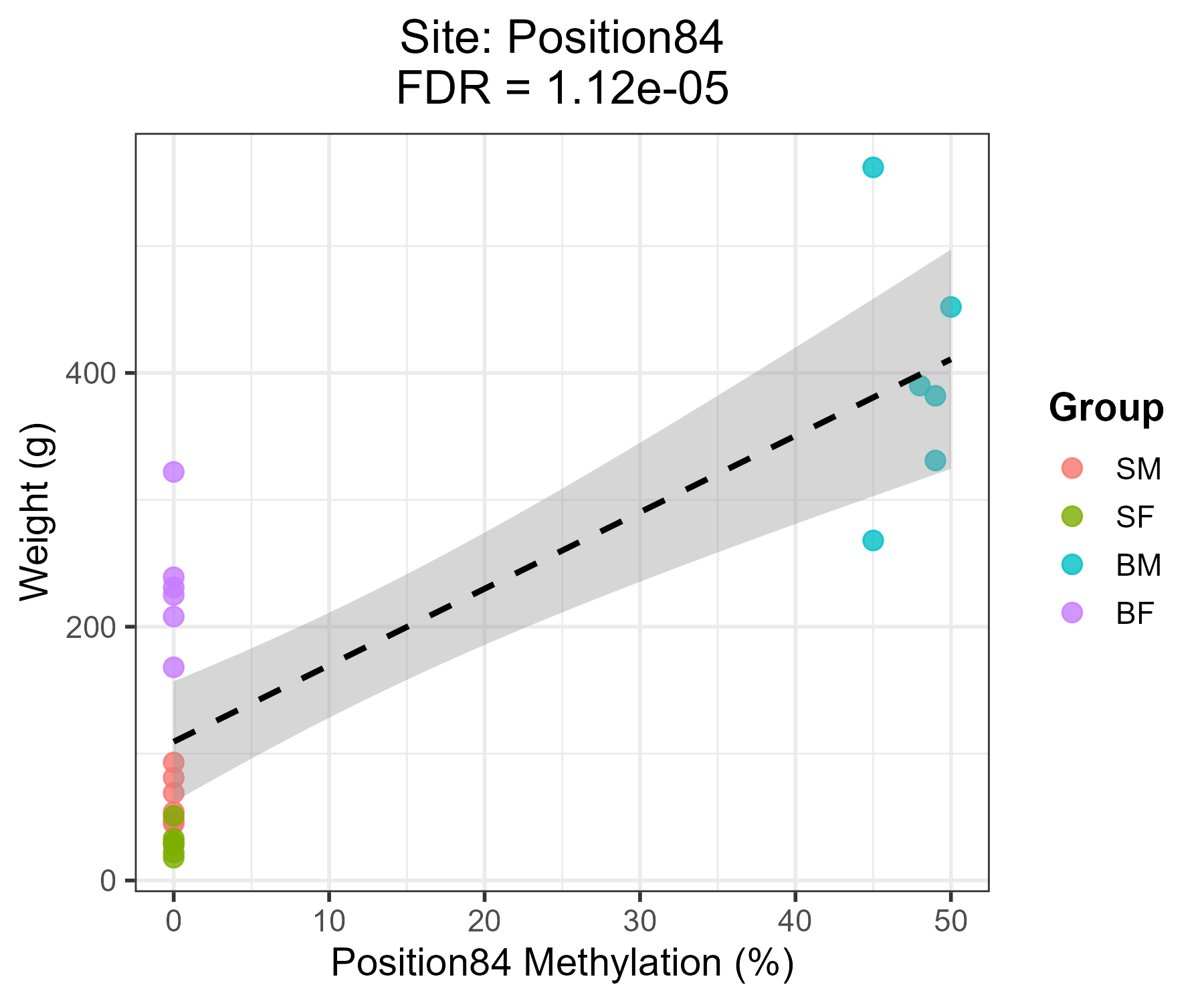

Supplement: Supplementary file 2 [file DataSheet1.zip › Regression_Plus_Strand/Position84_regression.tiff]

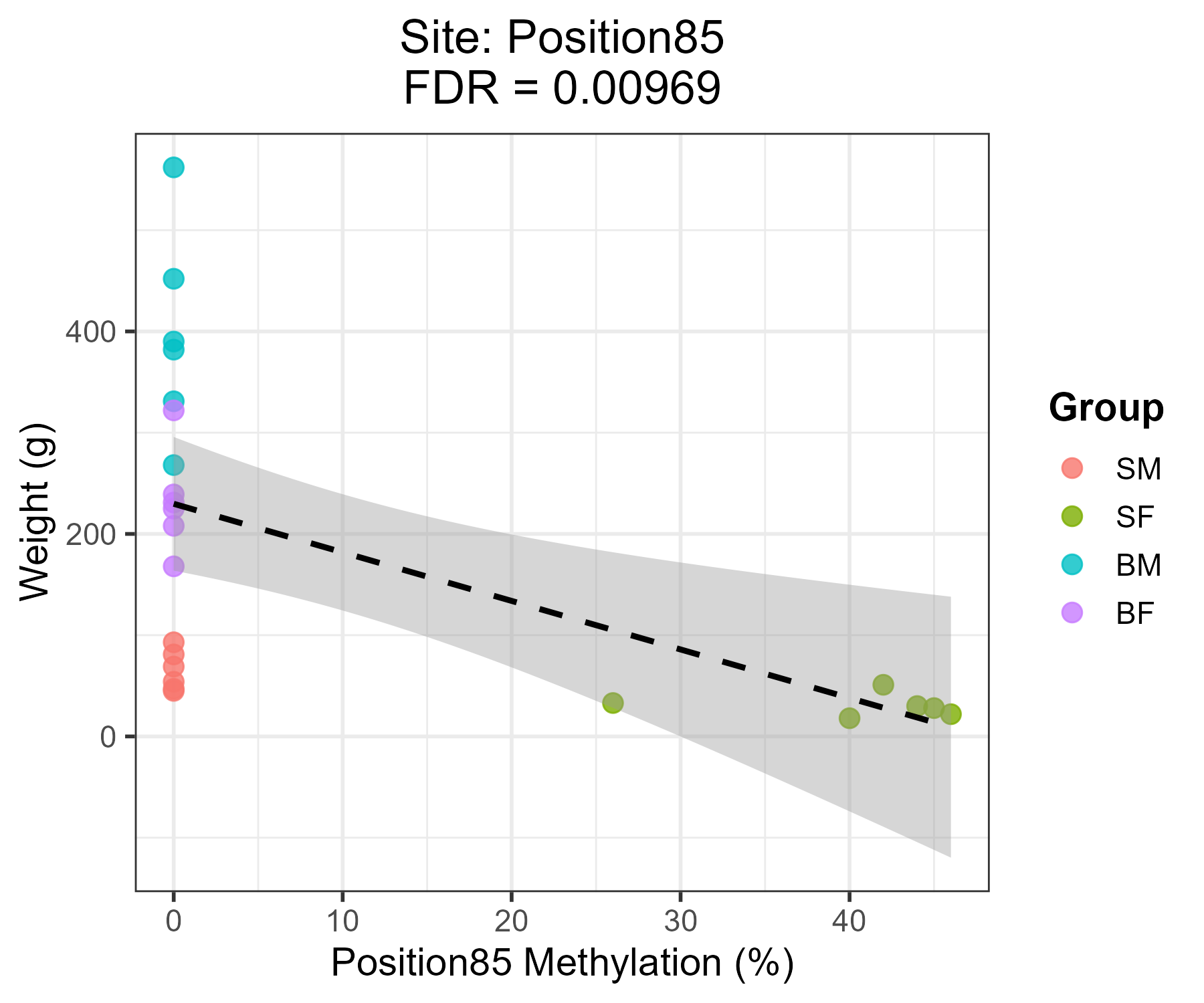

Supplement: Supplementary file 2 [file DataSheet1.zip › Regression_Plus_Strand/Position85_regression.tiff]

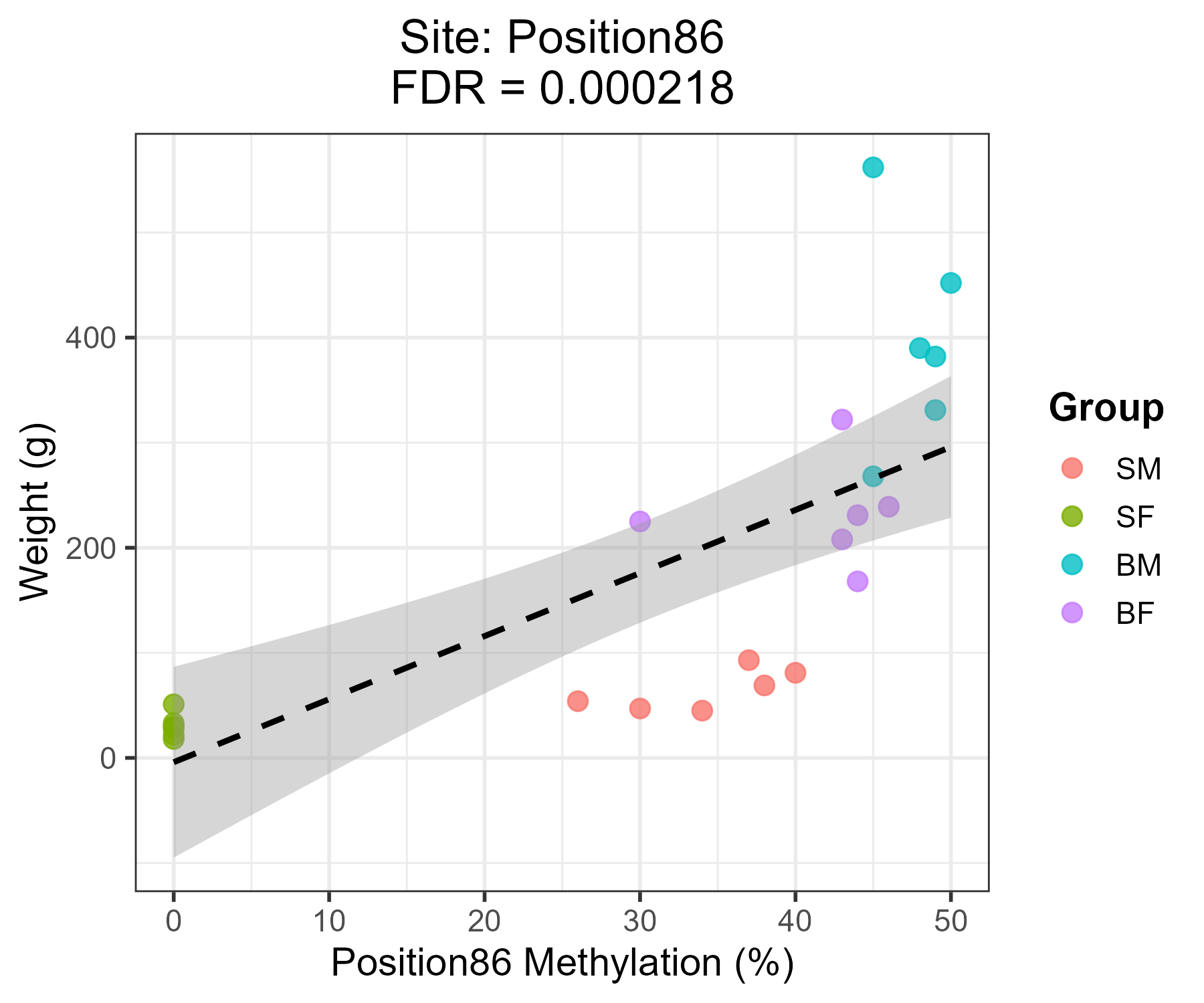

Supplement: Supplementary file 2 [file DataSheet1.zip › Regression_Plus_Strand/Position86_regression.tiff]

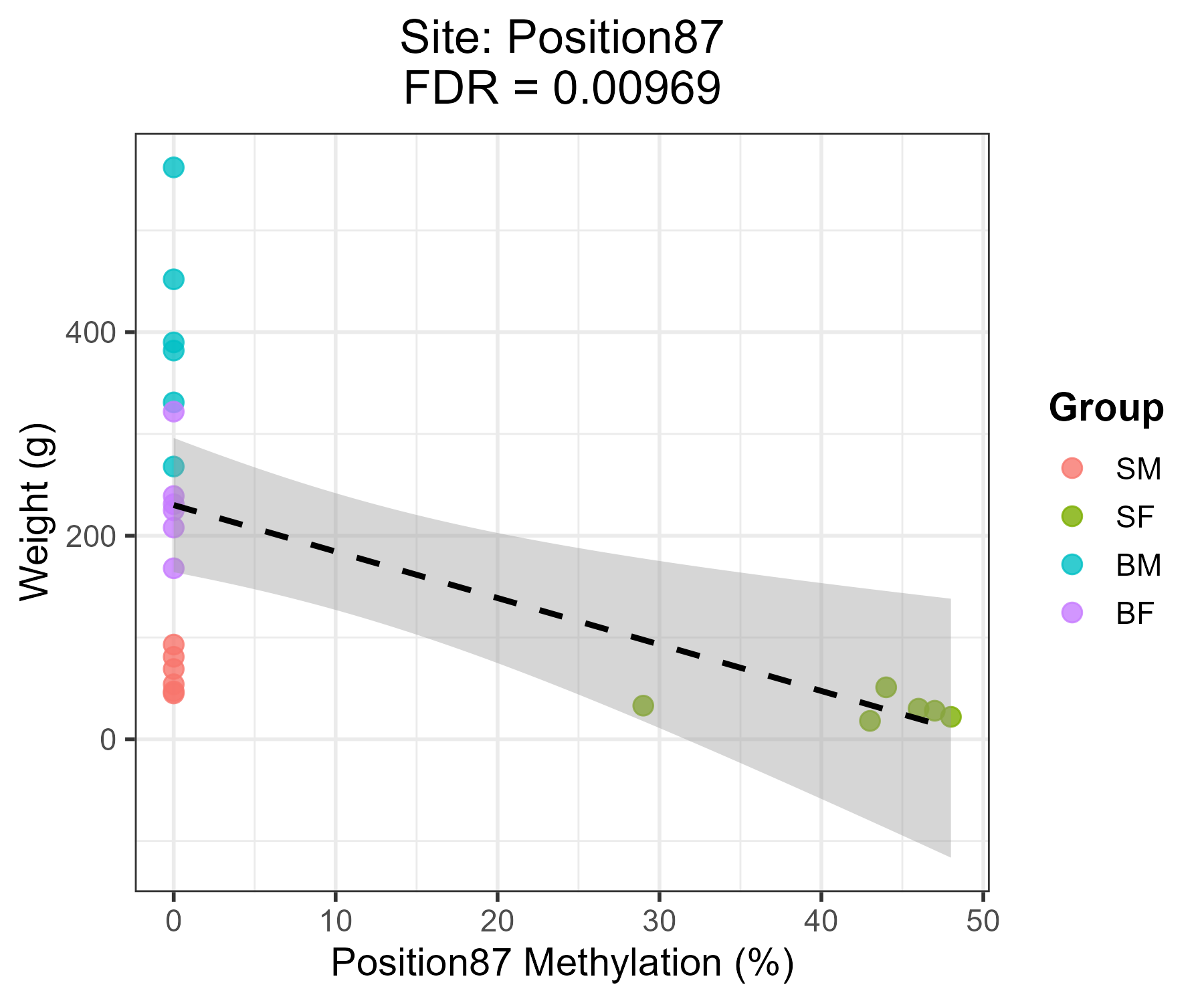

Supplement: Supplementary file 2 [file DataSheet1.zip › Regression_Plus_Strand/Position87_regression.tiff]

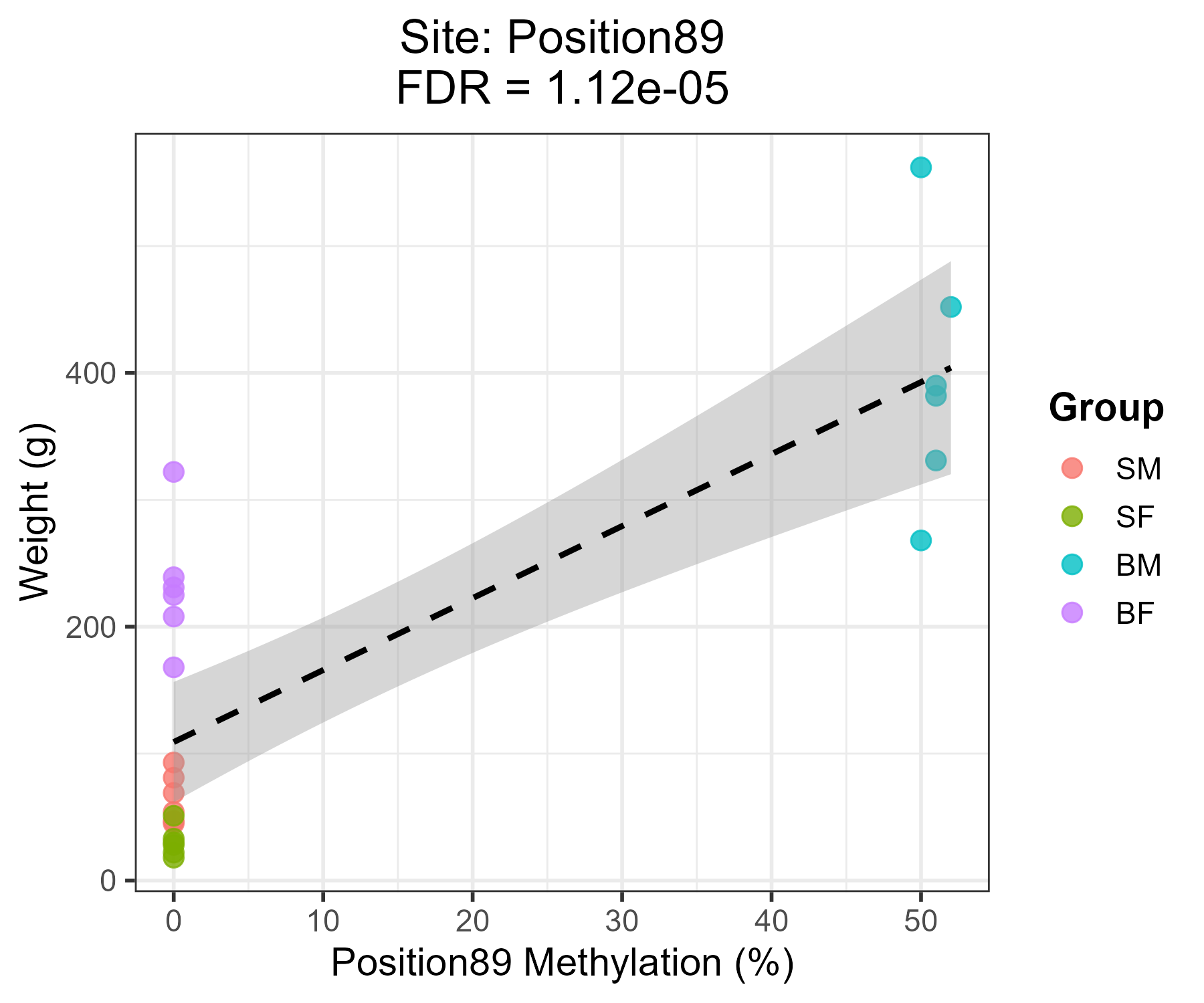

Supplement: Supplementary file 2 [file DataSheet1.zip › Regression_Plus_Strand/Position89_regression.tiff]

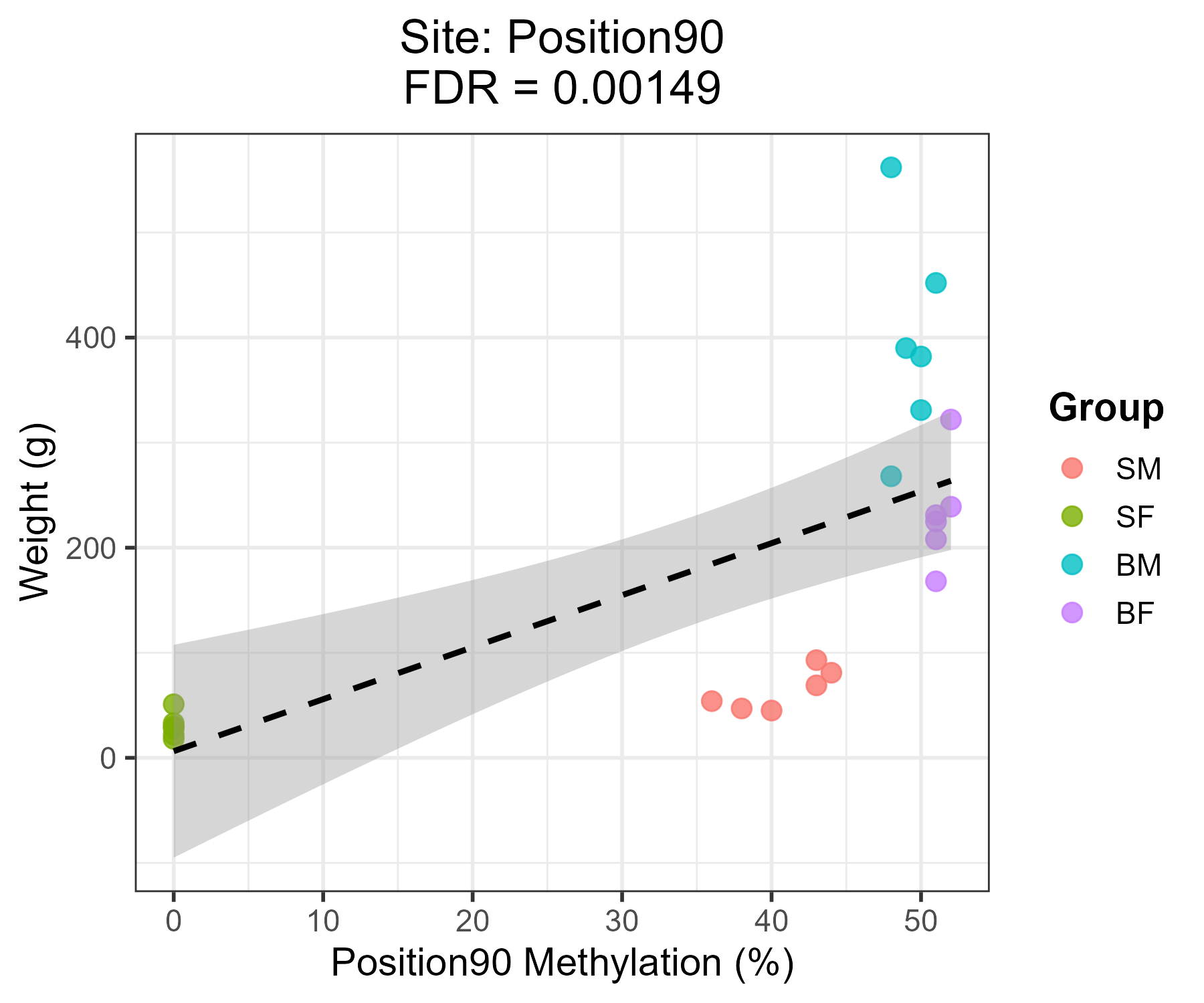

Supplement: Supplementary file 2 [file DataSheet1.zip › Regression_Plus_Strand/Position90_regression.tiff]

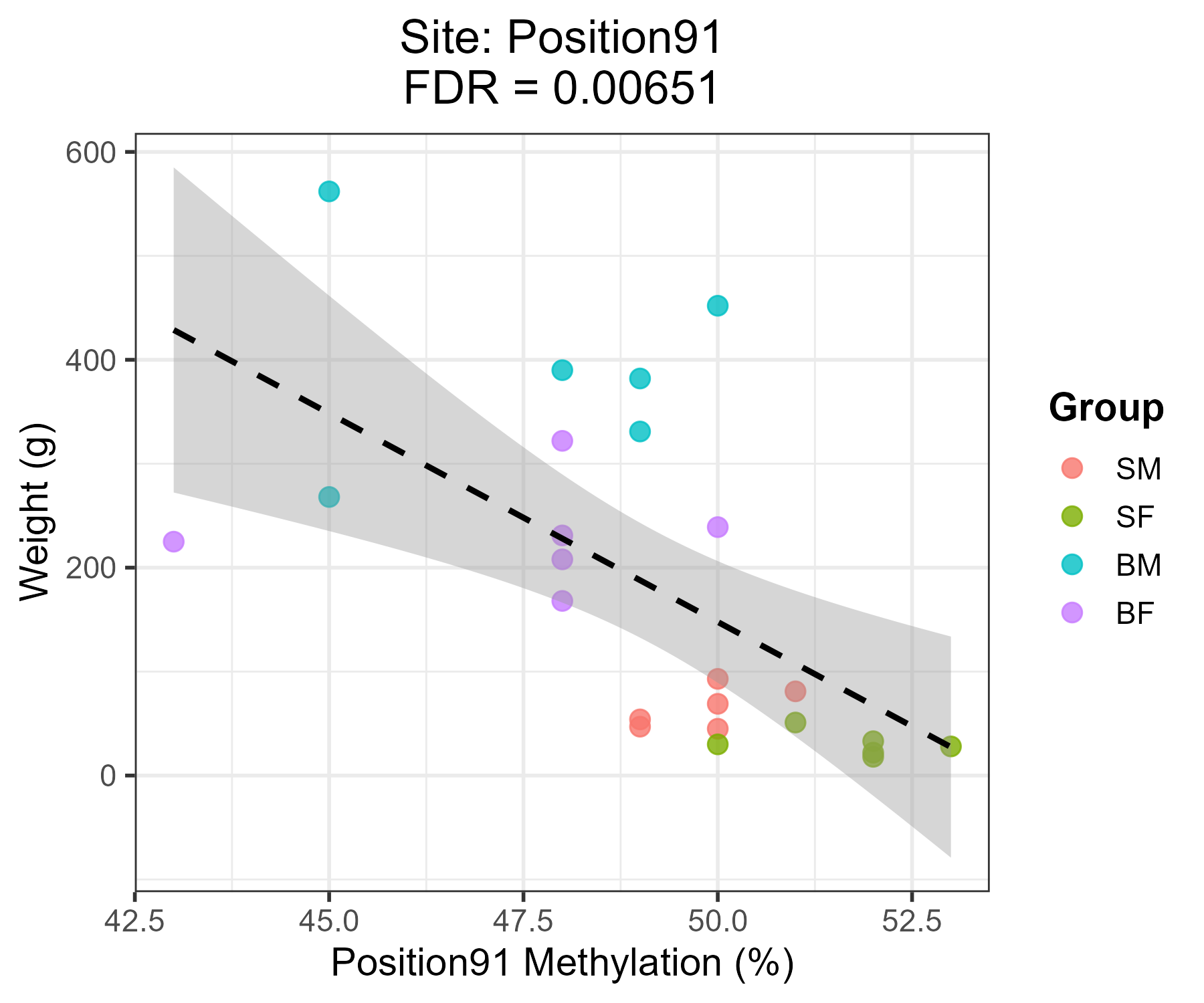

Supplement: Supplementary file 2 [file DataSheet1.zip › Regression_Plus_Strand/Position91_regression.tiff]

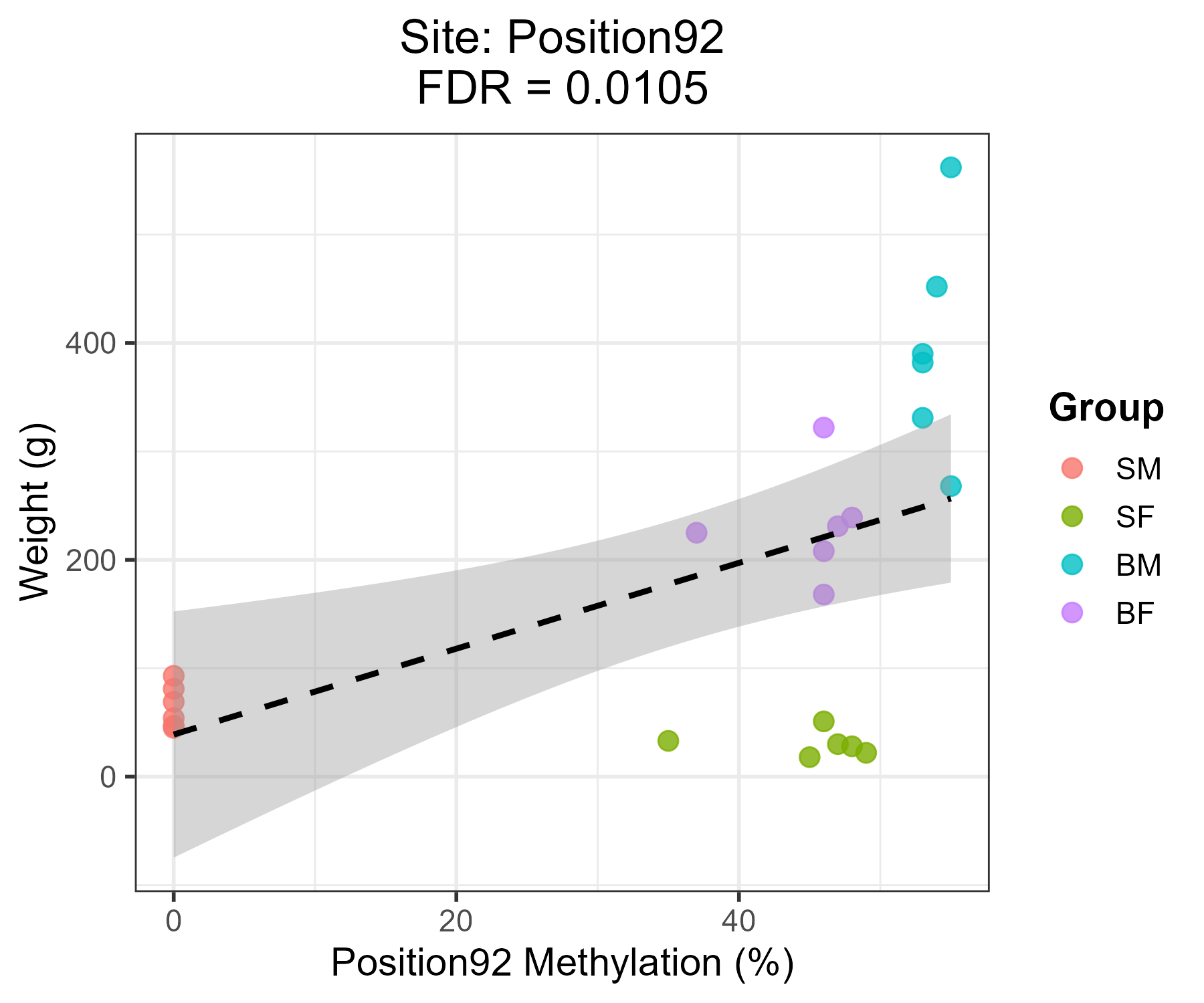

Supplement: Supplementary file 2 [file DataSheet1.zip › Regression_Plus_Strand/Position92_regression.tiff]

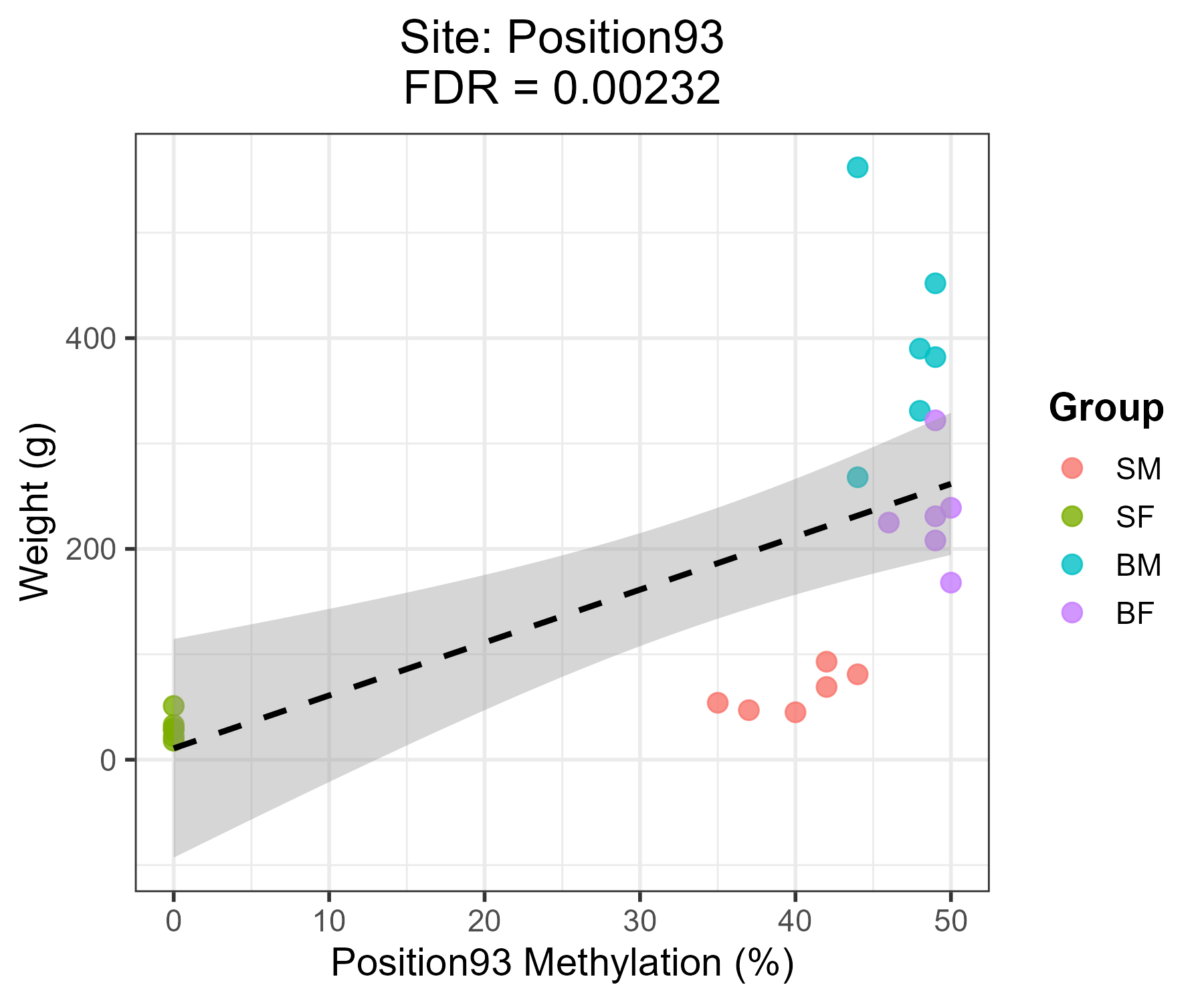

Supplement: Supplementary file 2 [file DataSheet1.zip › Regression_Plus_Strand/Position93_regression.tiff]

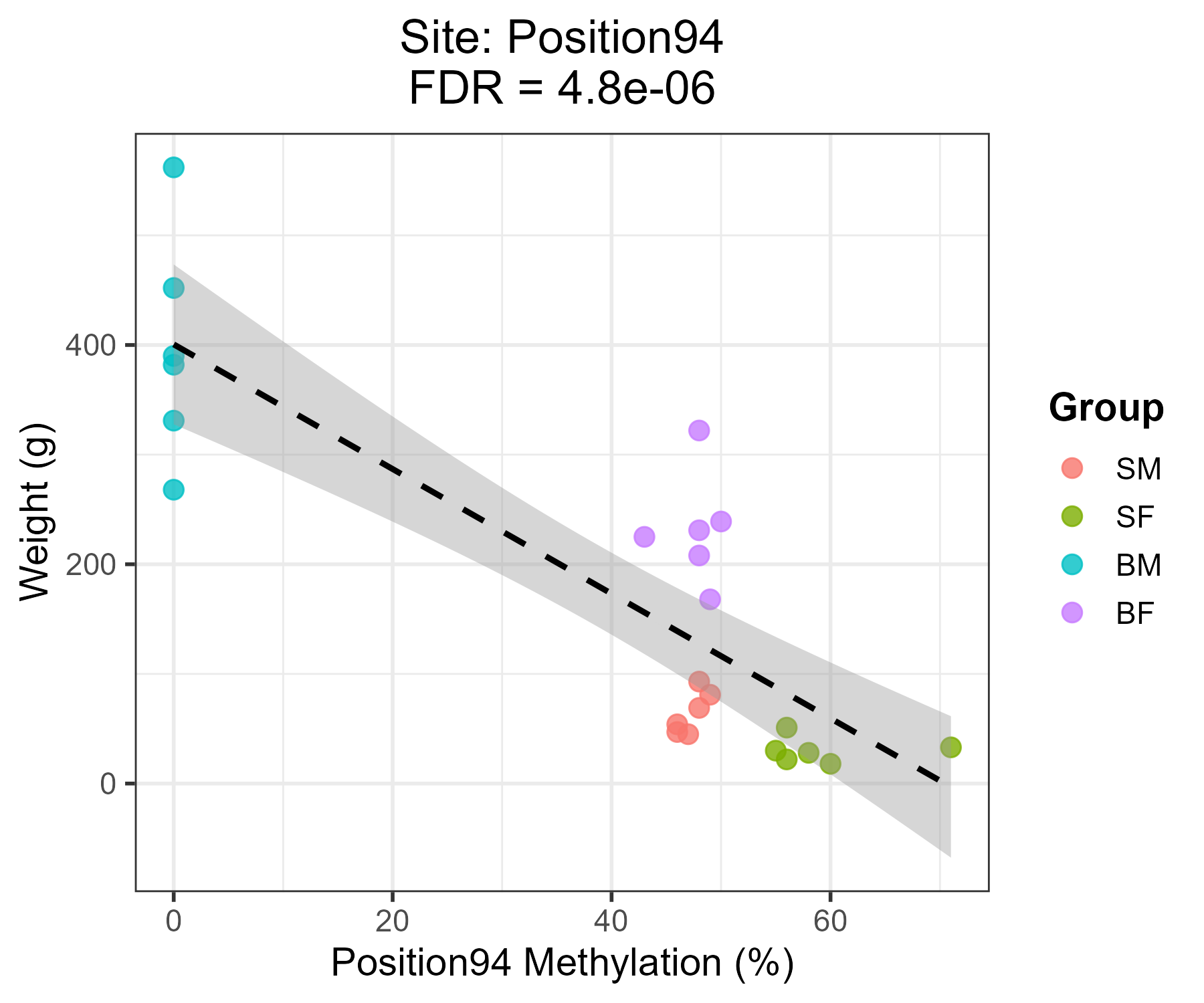

Supplement: Supplementary file 2 [file DataSheet1.zip › Regression_Plus_Strand/Position94_regression.tiff]

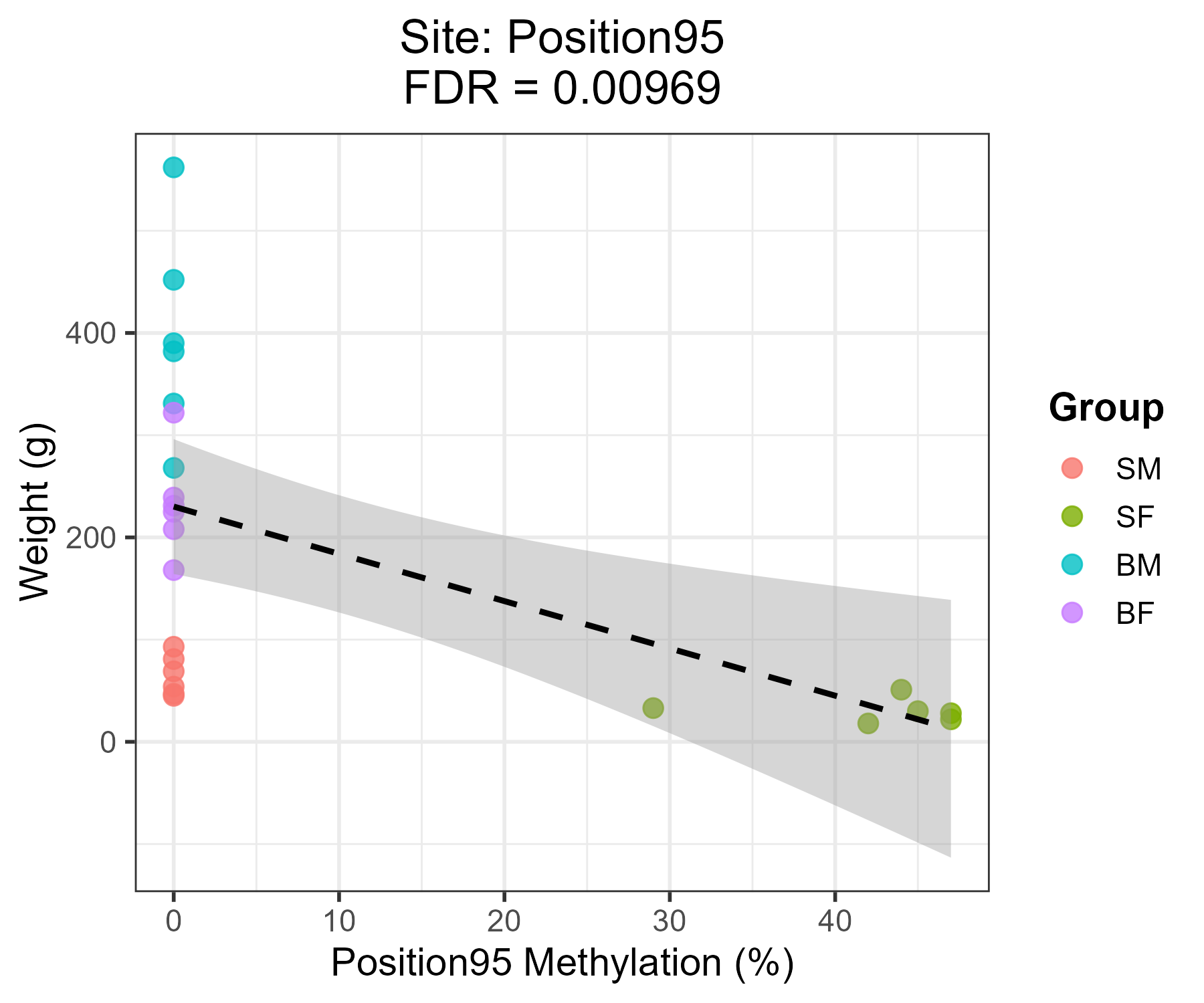

Supplement: Supplementary file 2 [file DataSheet1.zip › Regression_Plus_Strand/Position95_regression.tiff]

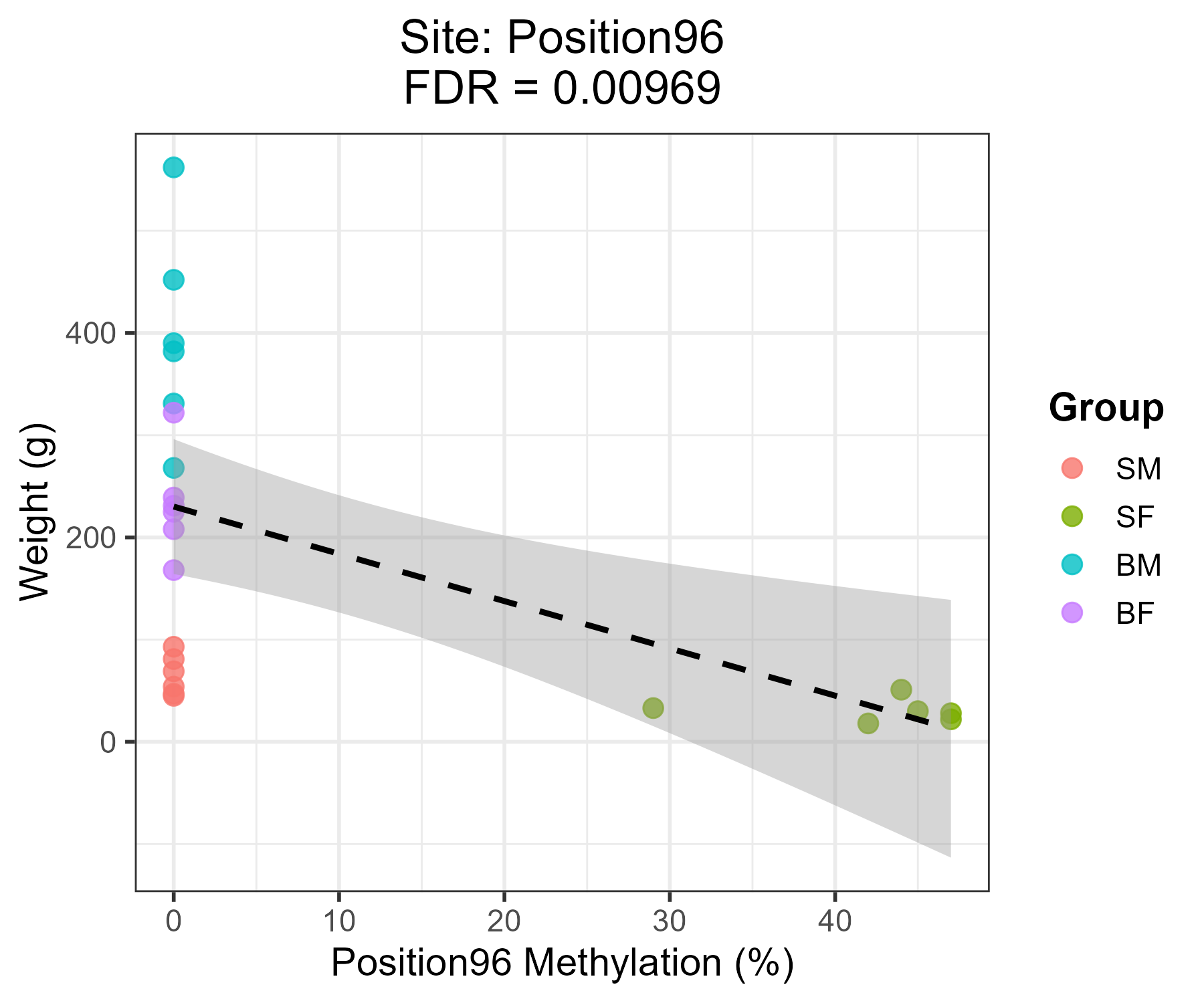

Supplement: Supplementary file 2 [file DataSheet1.zip › Regression_Plus_Strand/Position96_regression.tiff]

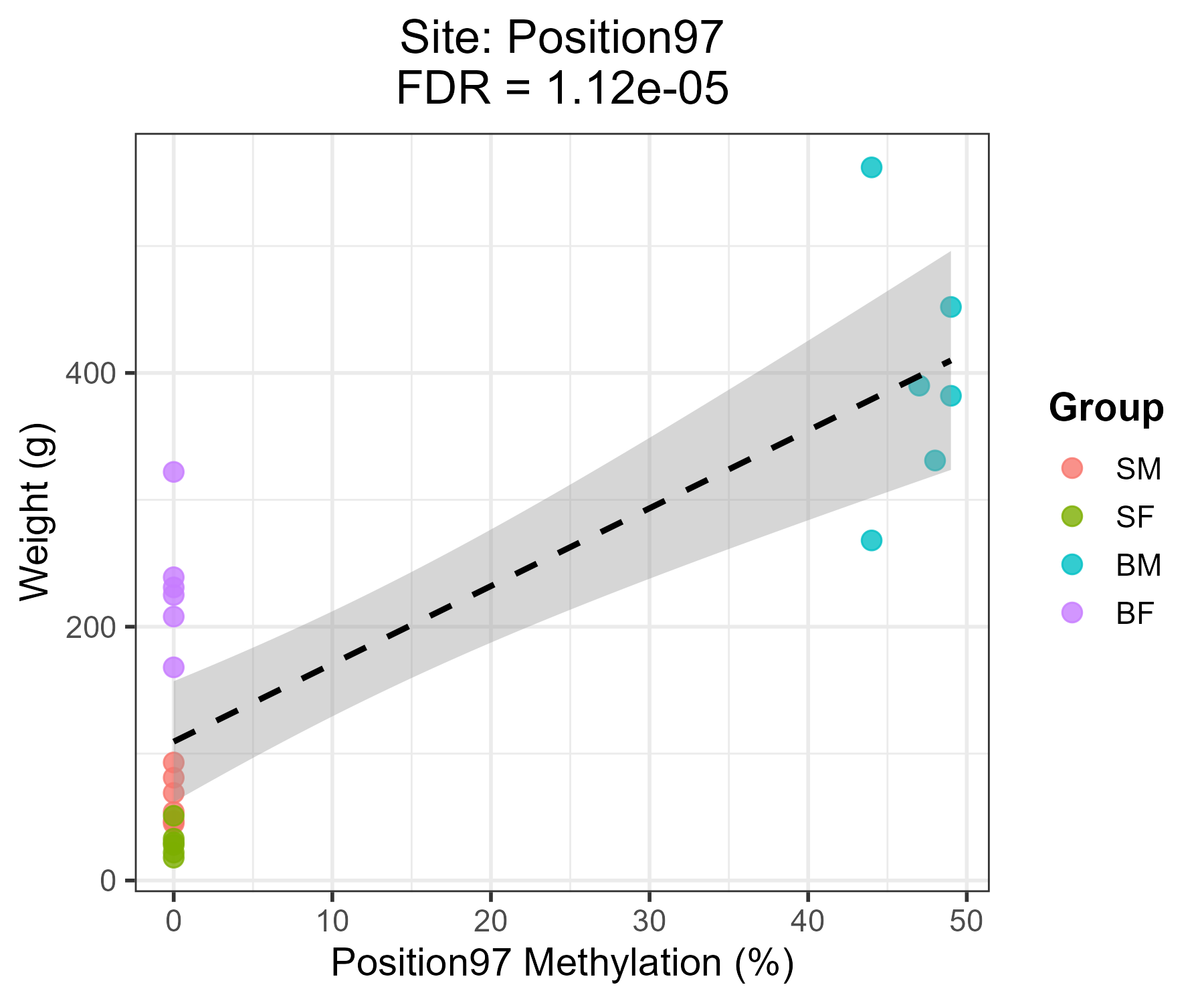

Supplement: Supplementary file 2 [file DataSheet1.zip › Regression_Plus_Strand/Position97_regression.tiff]

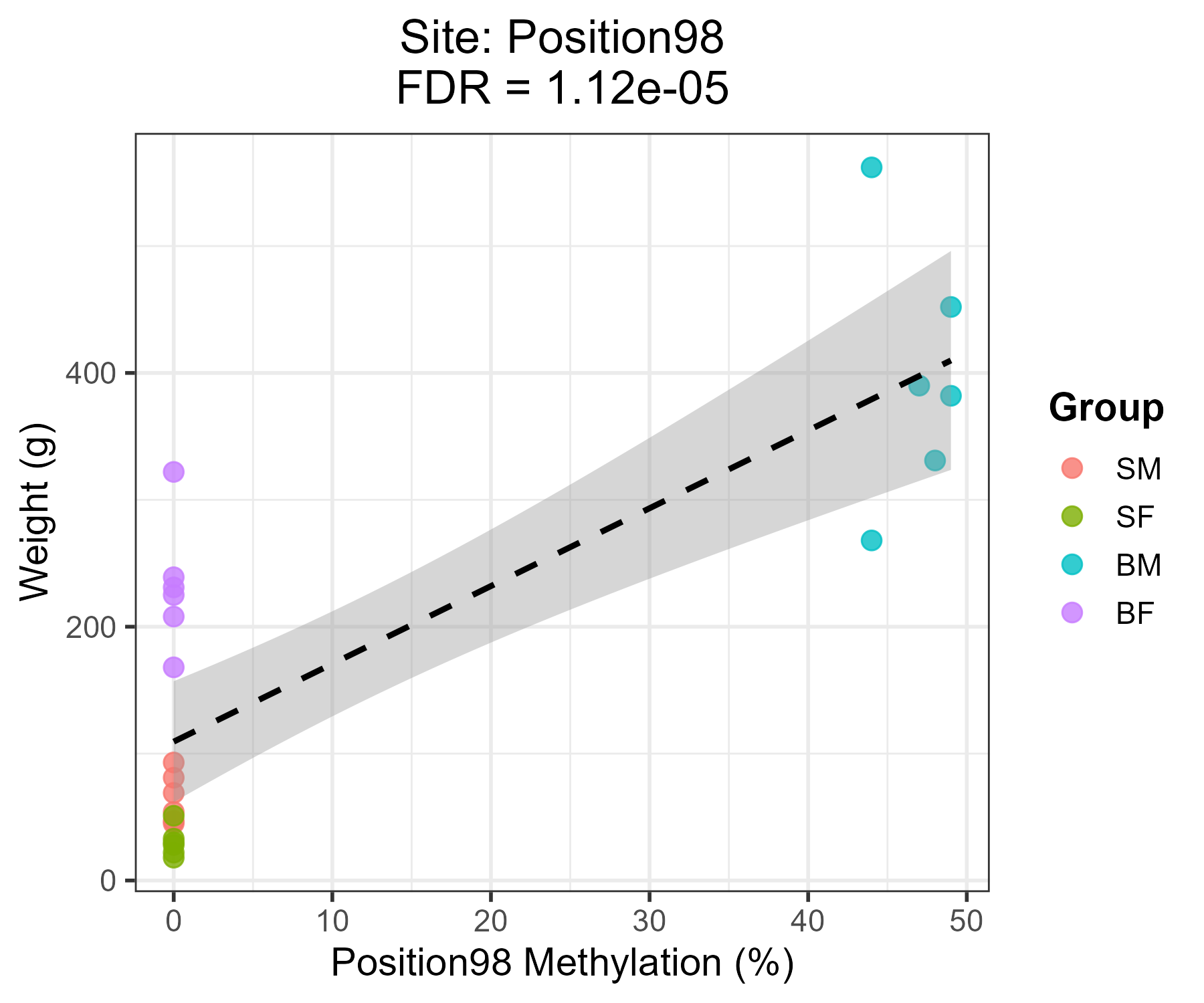

Supplement: Supplementary file 2 [file DataSheet1.zip › Regression_Plus_Strand/Position98_regression.tiff]

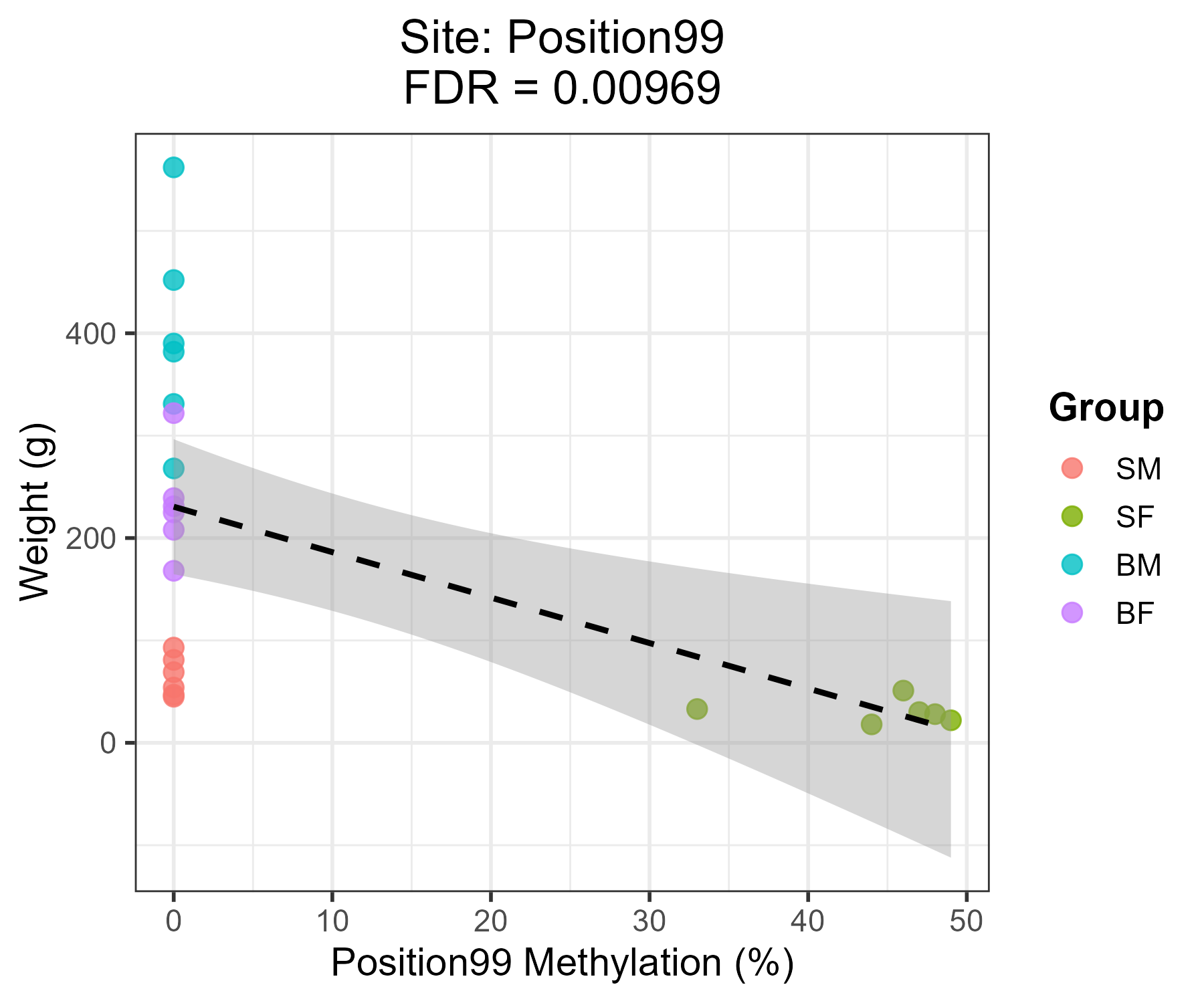

Supplement: Supplementary file 2 [file DataSheet1.zip › Regression_Plus_Strand/Position99_regression.tiff]

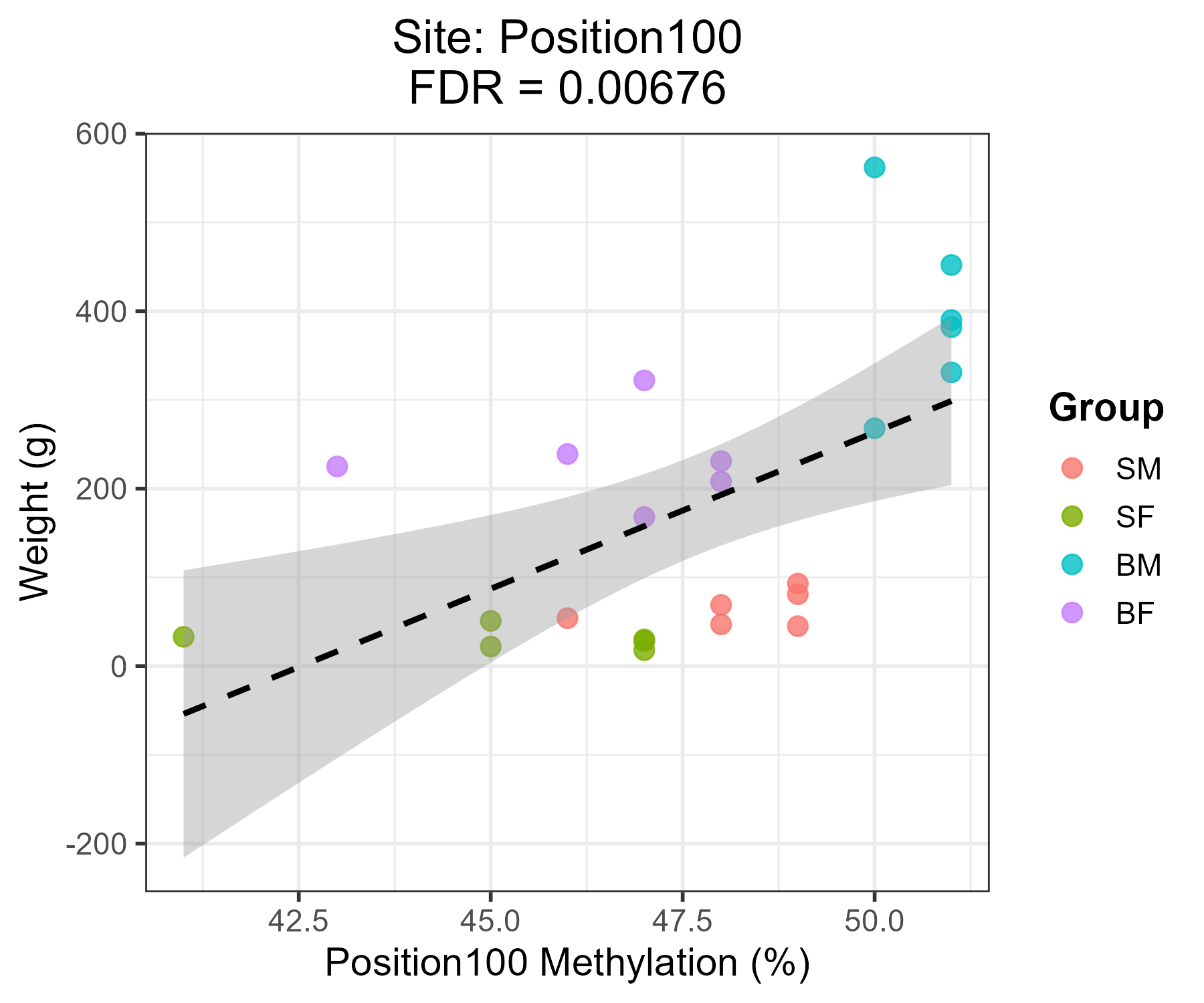

Supplement: Supplementary file 4 [file DataSheet2.zip › Regression_Minus_Strand/Position100_regression.tiff]

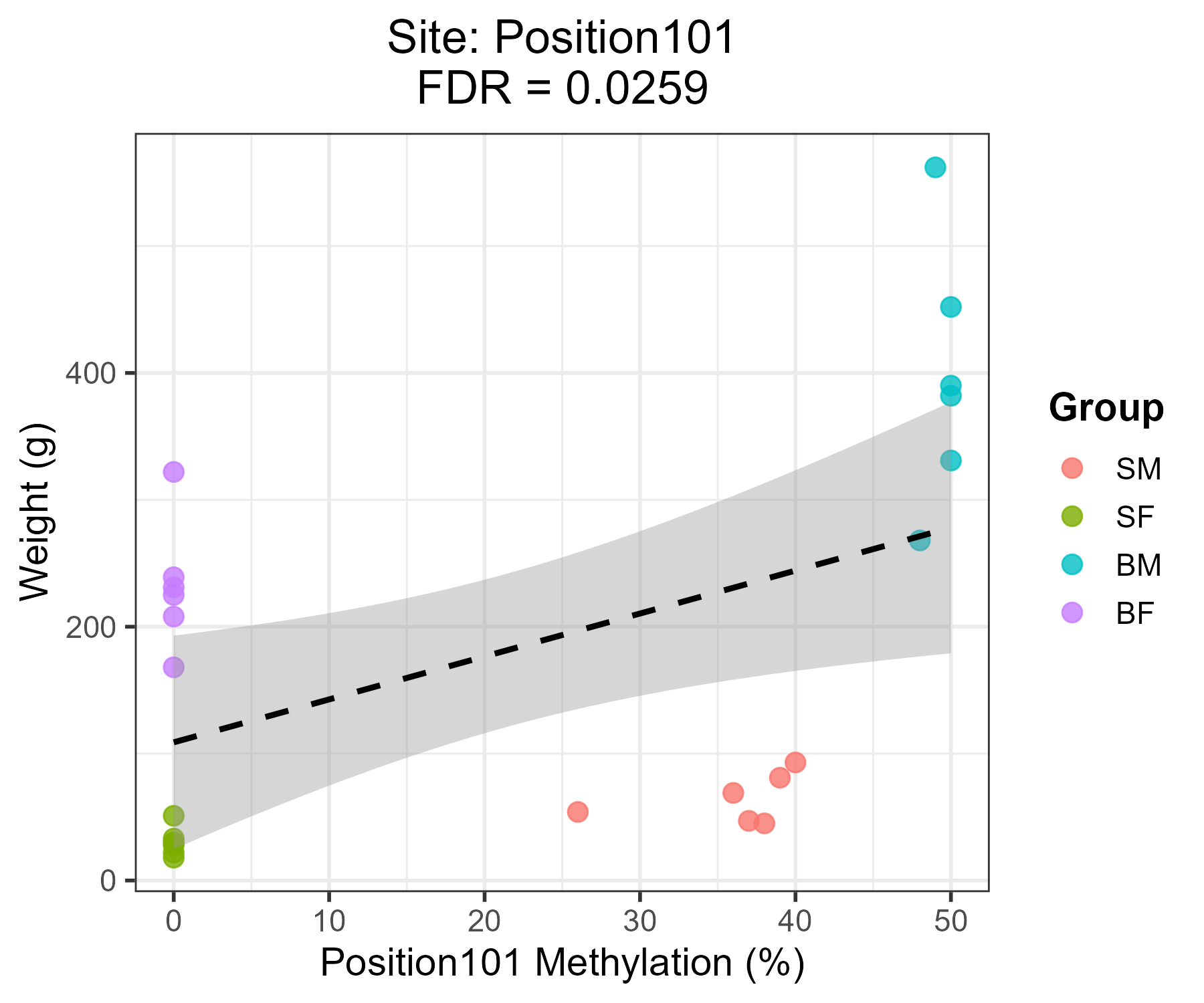

Supplement: Supplementary file 4 [file DataSheet2.zip › Regression_Minus_Strand/Position101_regression.tiff]

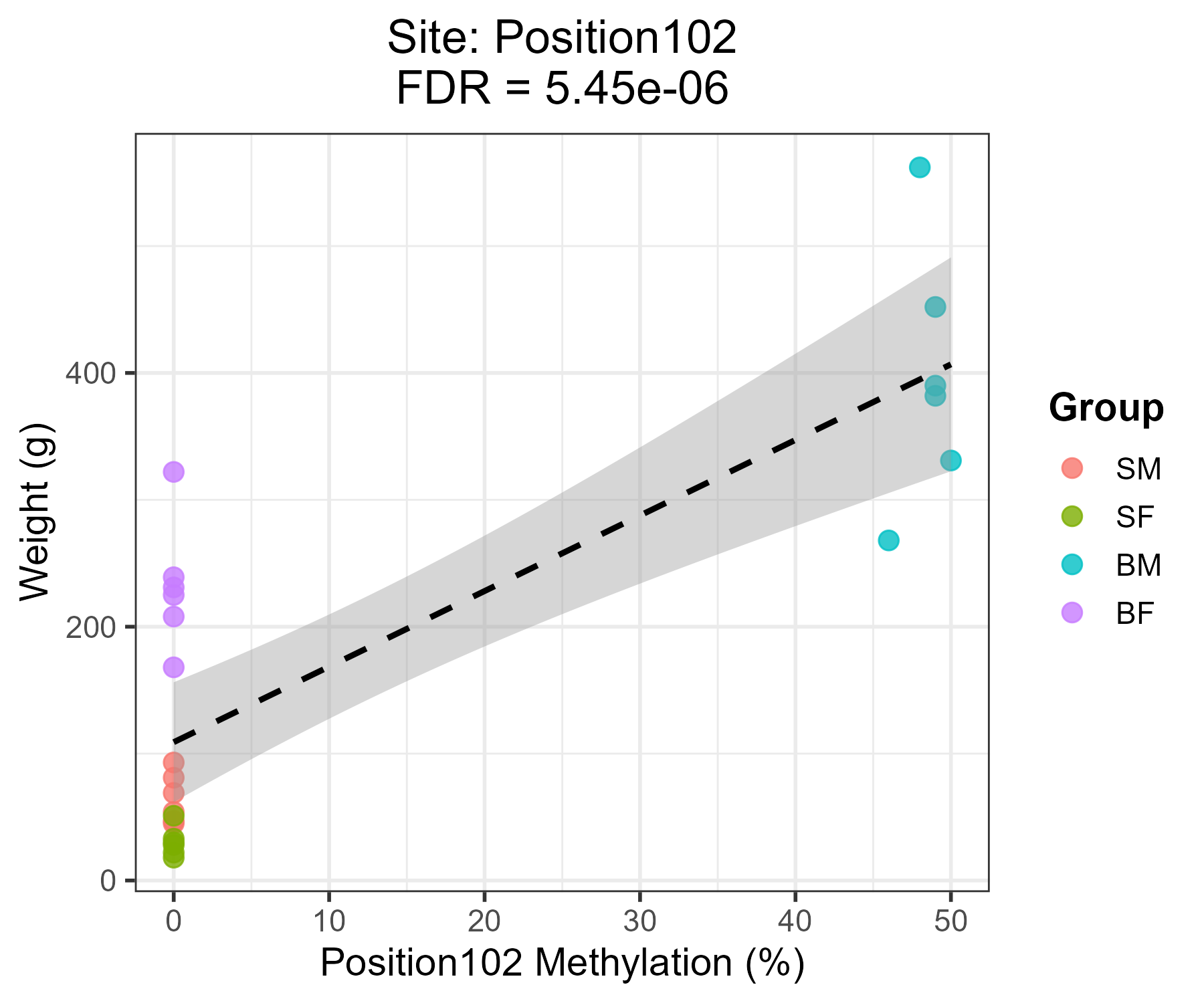

Supplement: Supplementary file 4 [file DataSheet2.zip › Regression_Minus_Strand/Position102_regression.tiff]

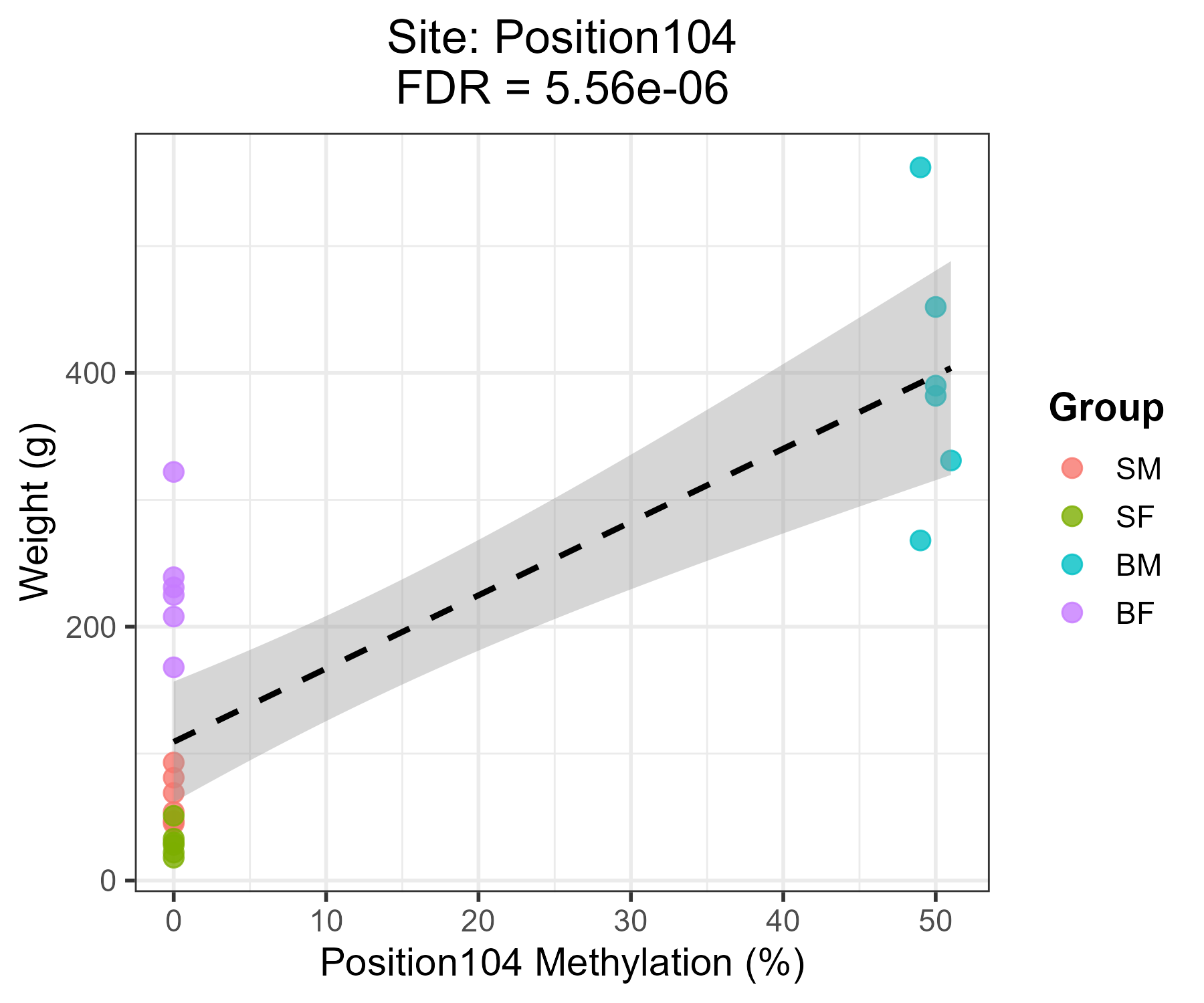

Supplement: Supplementary file 4 [file DataSheet2.zip › Regression_Minus_Strand/Position104_regression.tiff]

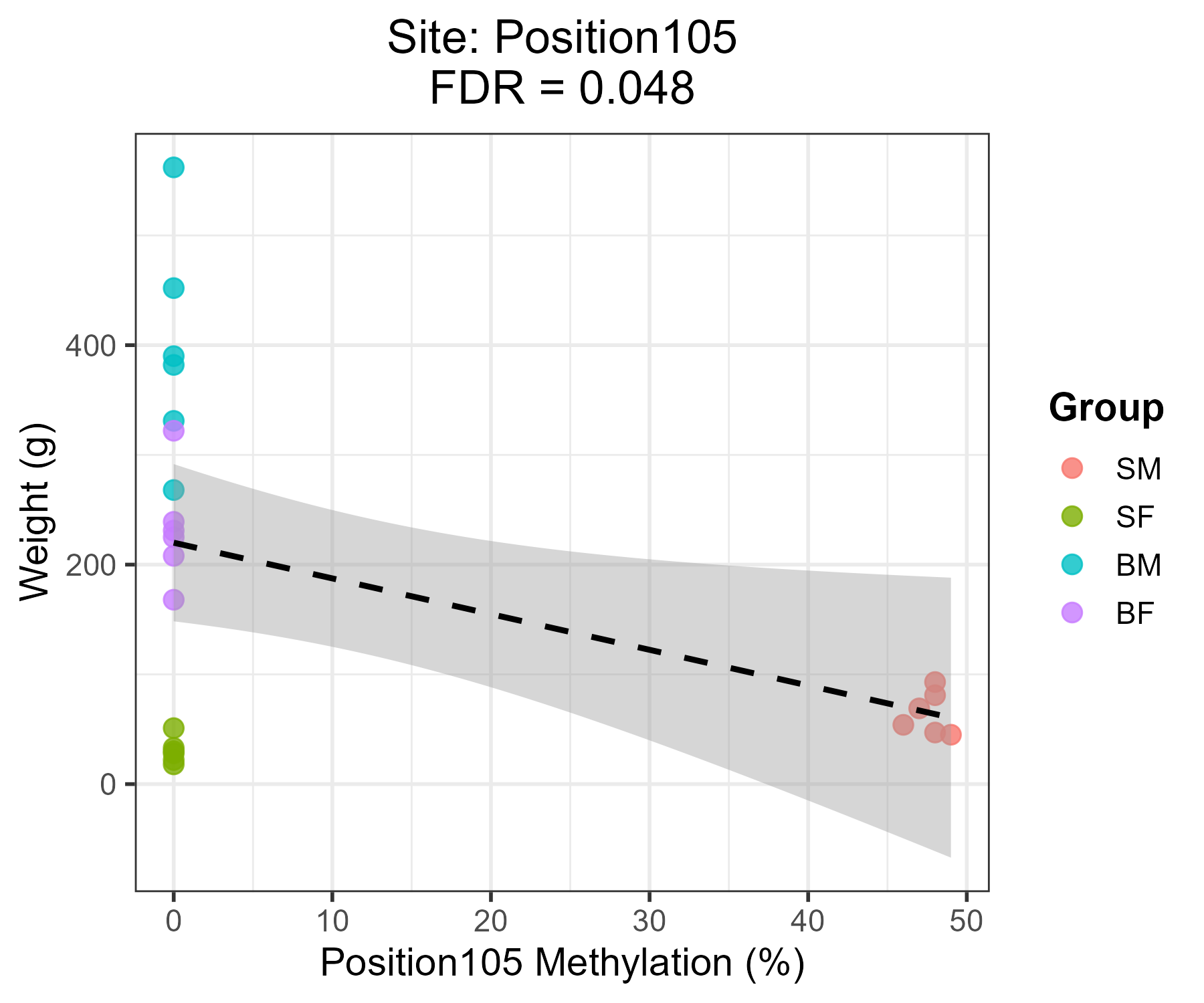

Supplement: Supplementary file 4 [file DataSheet2.zip › Regression_Minus_Strand/Position105_regression.tiff]

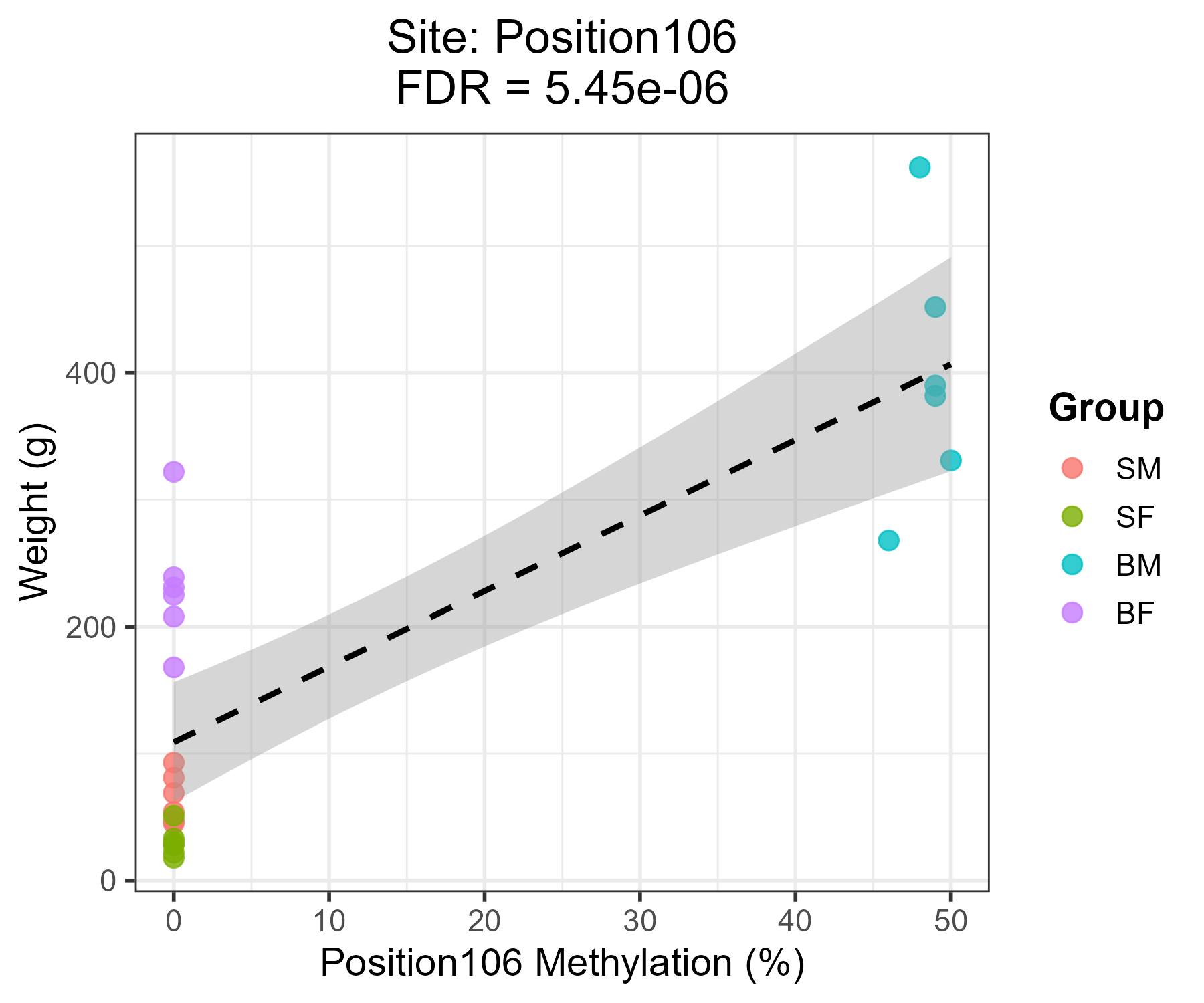

Supplement: Supplementary file 4 [file DataSheet2.zip › Regression_Minus_Strand/Position106_regression.tiff]

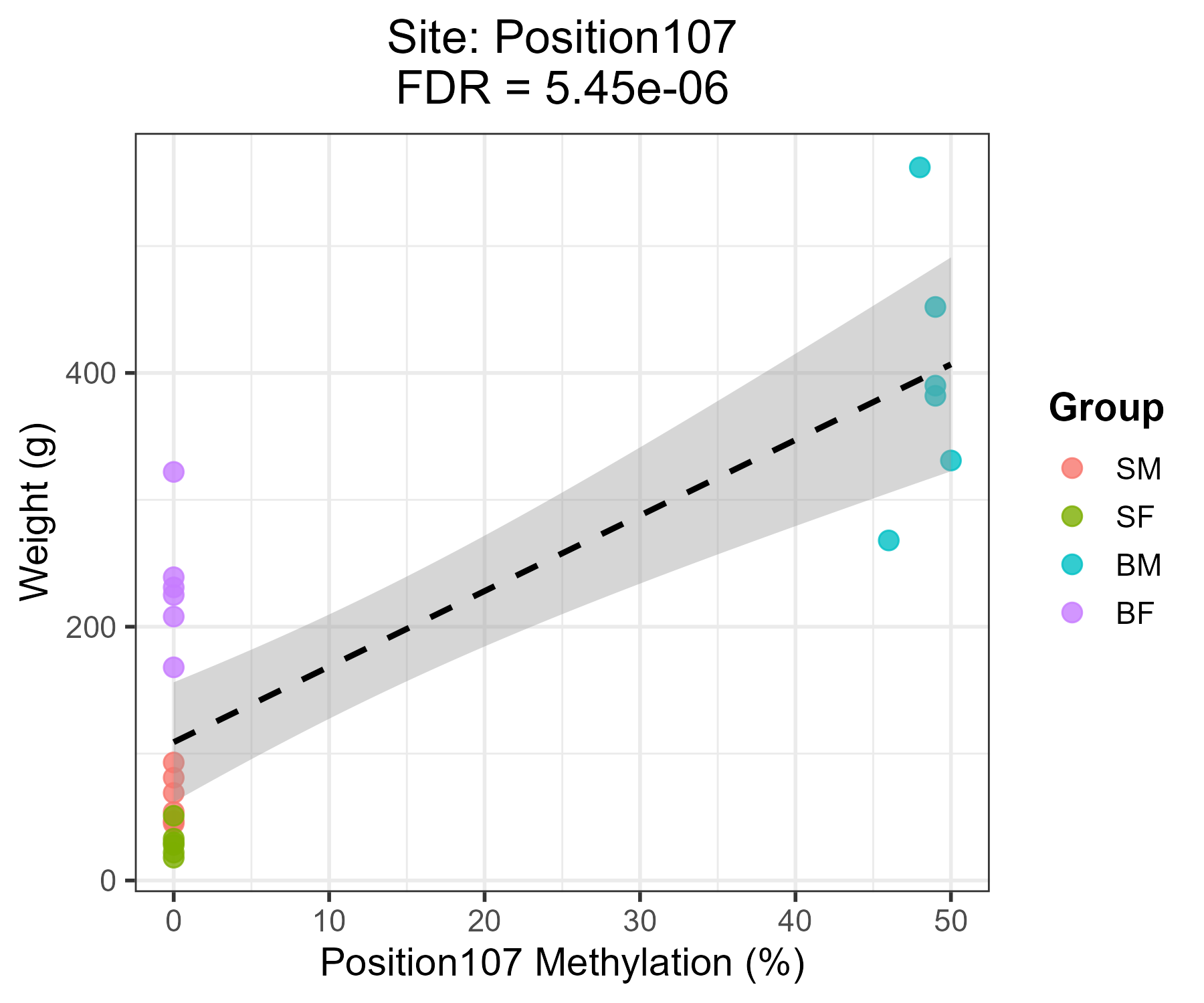

Supplement: Supplementary file 4 [file DataSheet2.zip › Regression_Minus_Strand/Position107_regression.tiff]

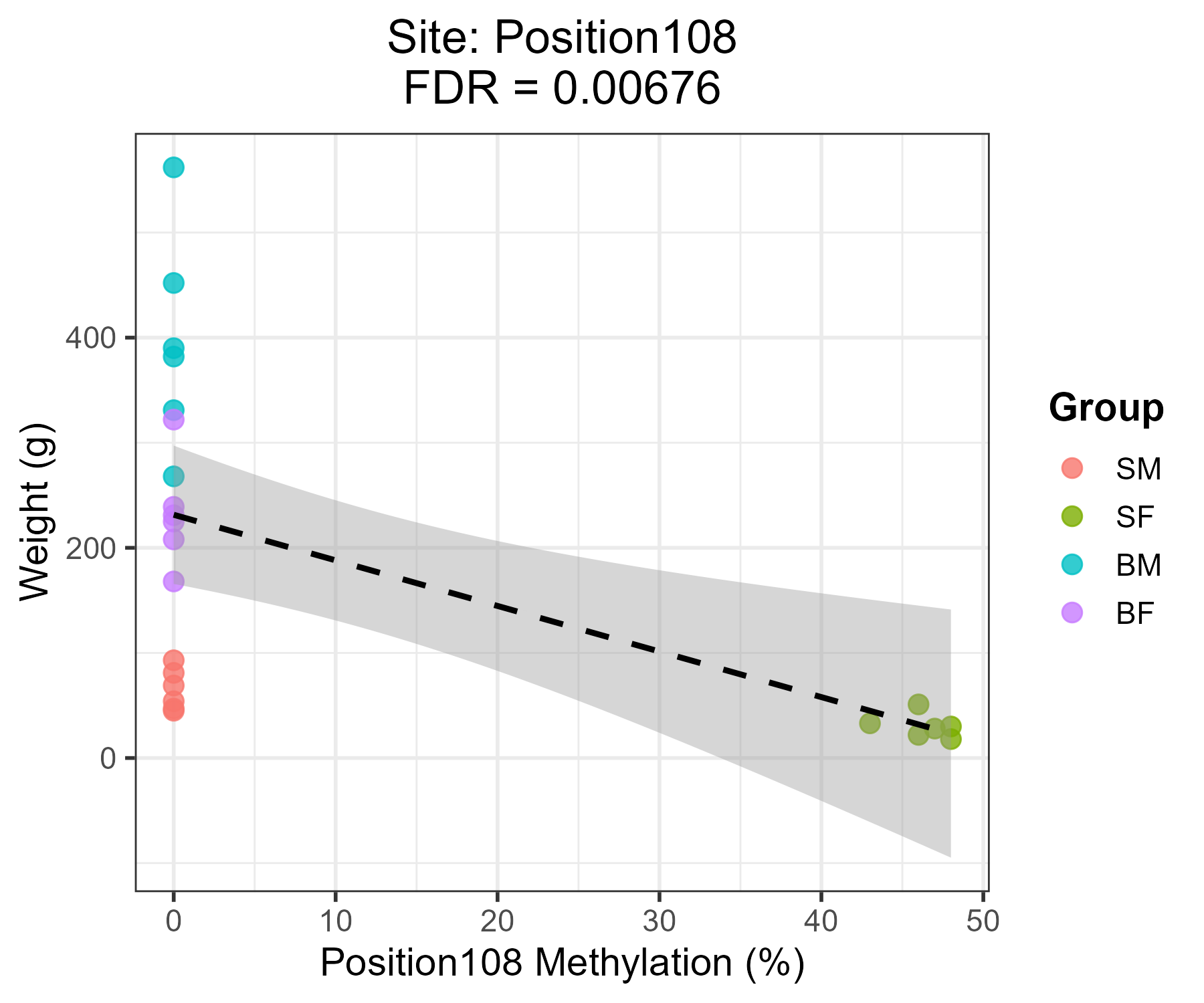

Supplement: Supplementary file 4 [file DataSheet2.zip › Regression_Minus_Strand/Position108_regression.tiff]

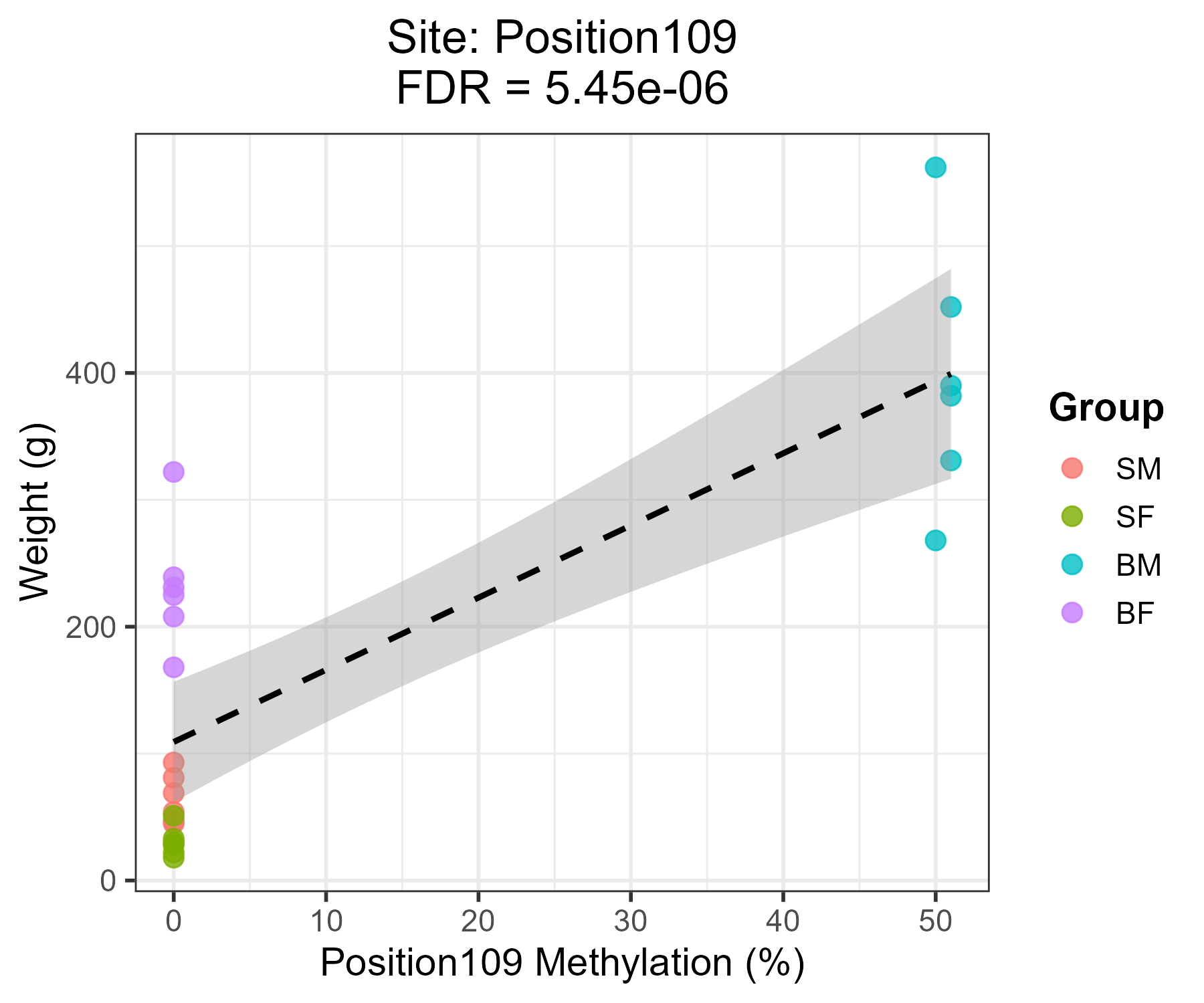

Supplement: Supplementary file 4 [file DataSheet2.zip › Regression_Minus_Strand/Position109_regression.tiff]

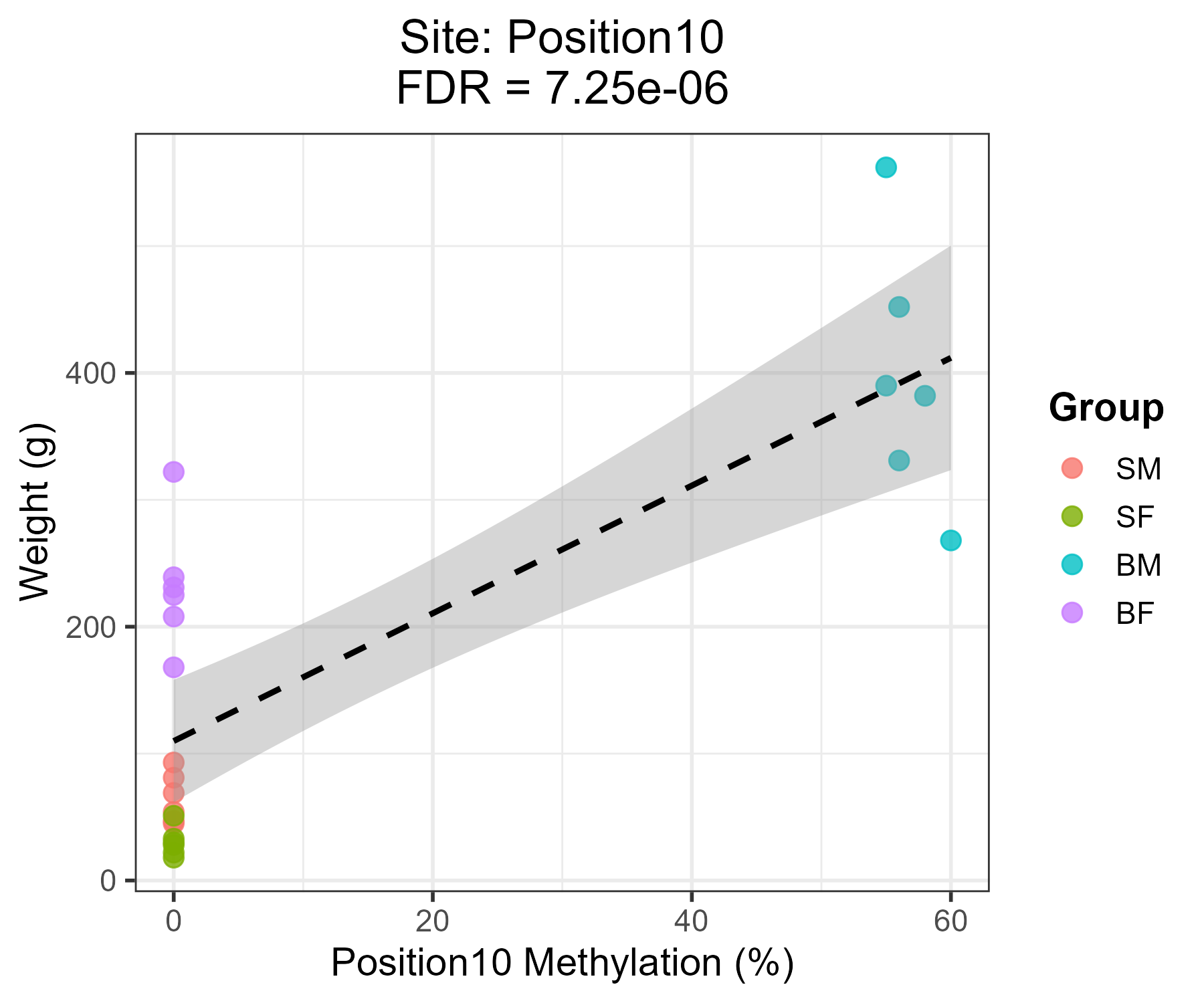

Supplement: Supplementary file 4 [file DataSheet2.zip › Regression_Minus_Strand/Position10_regression.tiff]
