# Supplementary material for: Mitochondrial methylation is linked to sexually dimorphic growth in Nile tilapia (Oreochromis niloticus)
Source: Front Cell Dev Biol. 2025 Aug 5;13:1643817. doi: 10.3389/fcell.2025.1643817 (PMC12361127; doi:10.3389/fcell.2025.1643817)
Supplement: Supplementary file 3 [file Table2.docx]

**Supplementary Table 1:** Individual fish weights distribution by group. This table lists the weight of individual fish categorized into four groups: BF (Fast-growing Female), BM (Fast-growing Male), SF (Slow-growing Female), and SM (Slow-growing Male).

| **Group** | **Weight (in g)** | **Average Weight (in g)** |
| --- | --- | --- |
| BF | 322  231  208  225  239  168 | 232.17 ± 20.71 |
| BM | 268  562  452  390  331  382 | 397.50 ± 41.44 |
| SF | 51  30  28  18  33  22 | 30.33 ± 4.70 |
| SM | 69  81  45  54  93  47 | 64.83 ± 7.96 |
