# Supplementary figures and images for: Mitochondrial methylation is linked to sexually dimorphic growth in Nile tilapia (Oreochromis niloticus) (part 3 of 3)
Source: Front Cell Dev Biol. 2025 Aug 5;13:1643817. doi: 10.3389/fcell.2025.1643817 (PMC12361127; doi:10.3389/fcell.2025.1643817)

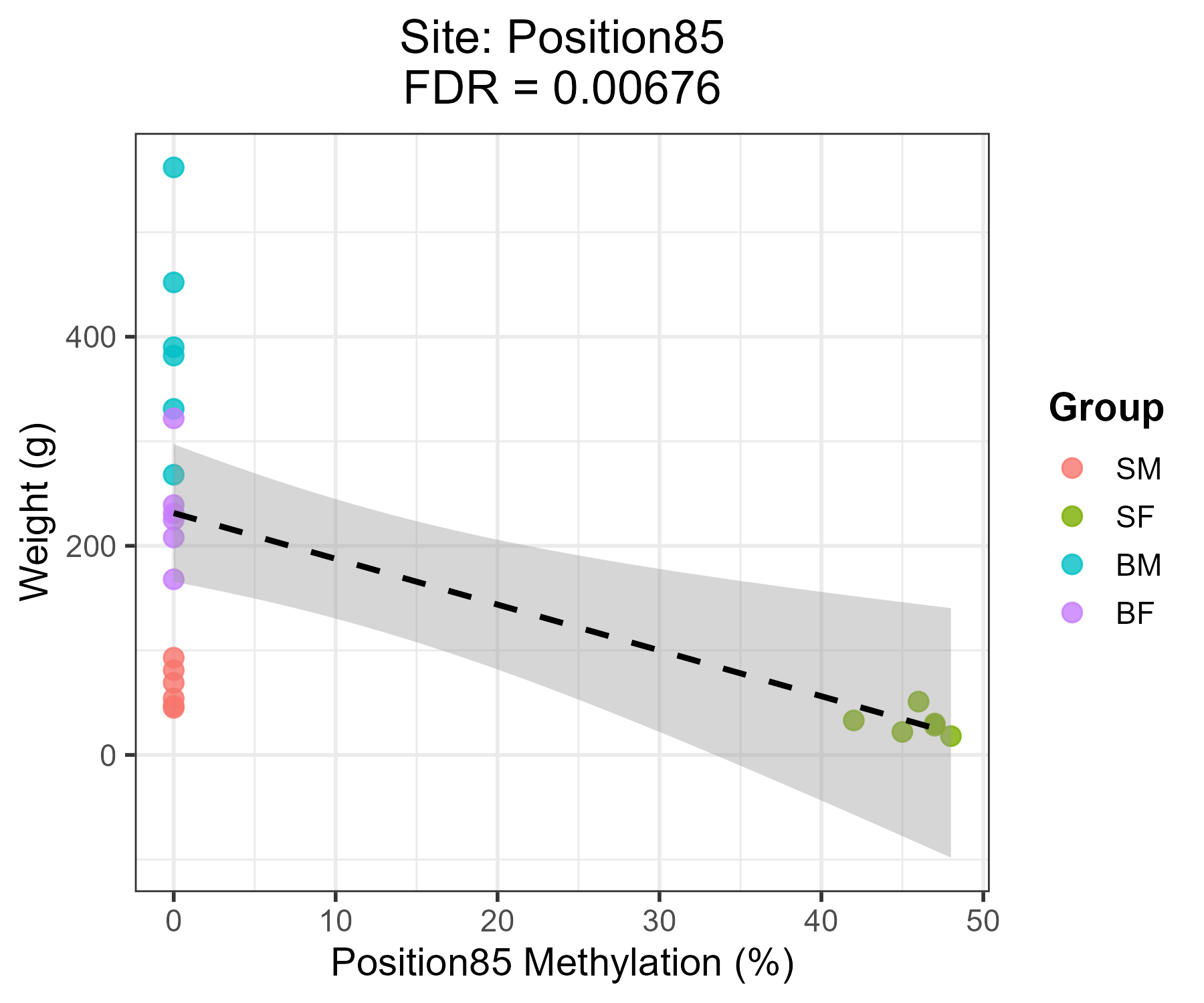

Supplement: Supplementary file 4 [file DataSheet2.zip › Regression_Minus_Strand/Position85_regression.tiff]

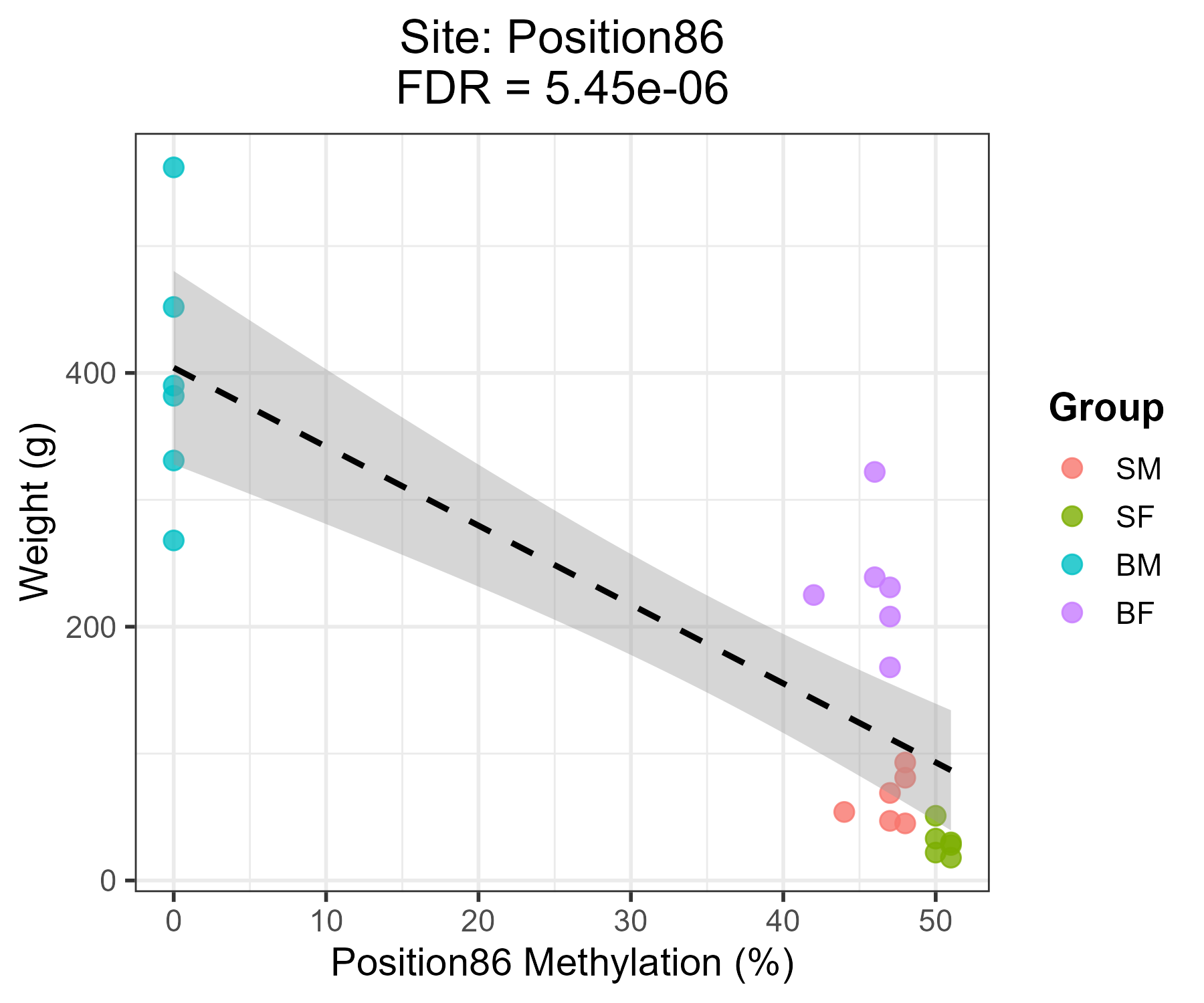

Supplement: Supplementary file 4 [file DataSheet2.zip › Regression_Minus_Strand/Position86_regression.tiff]

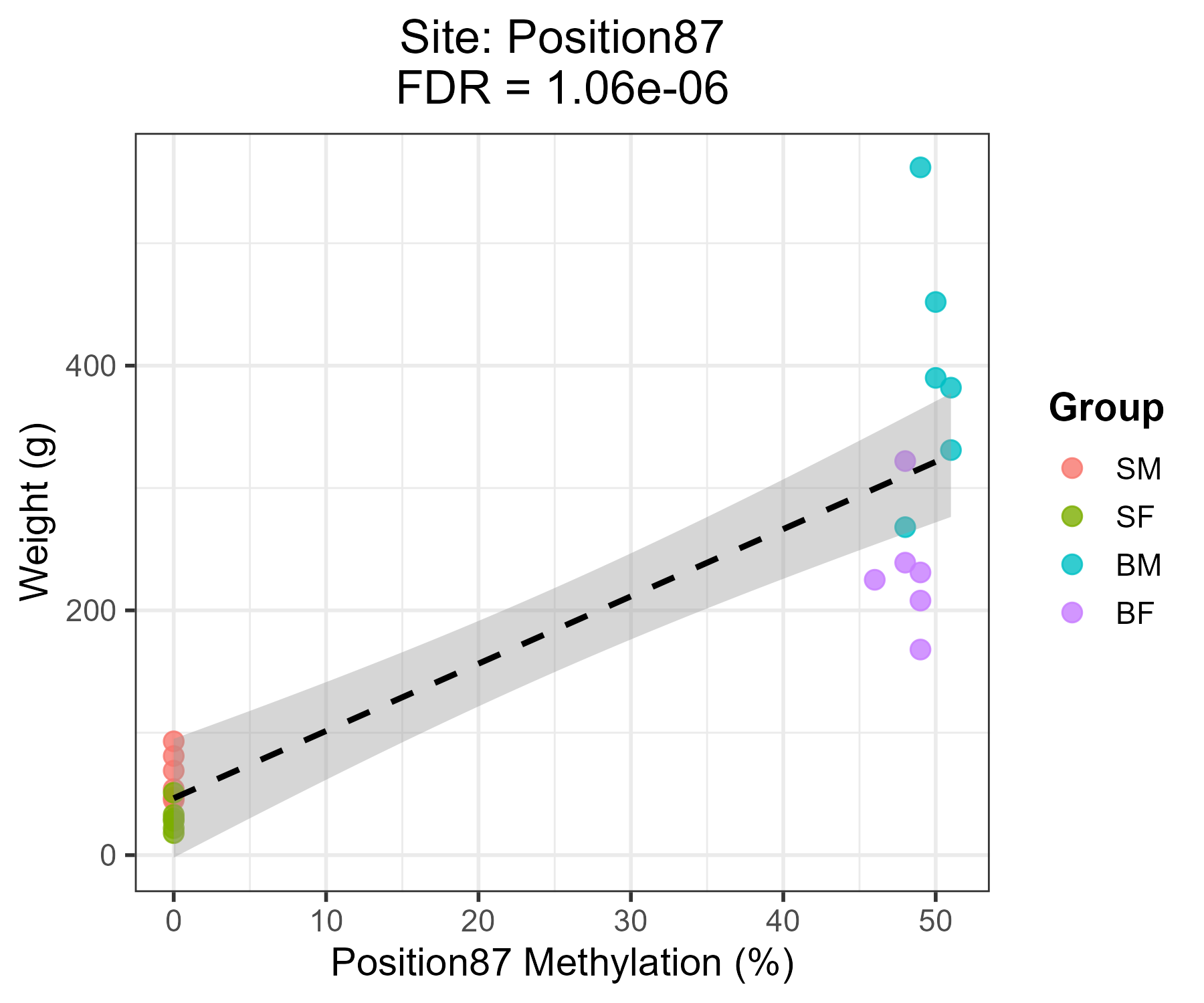

Supplement: Supplementary file 4 [file DataSheet2.zip › Regression_Minus_Strand/Position87_regression.tiff]

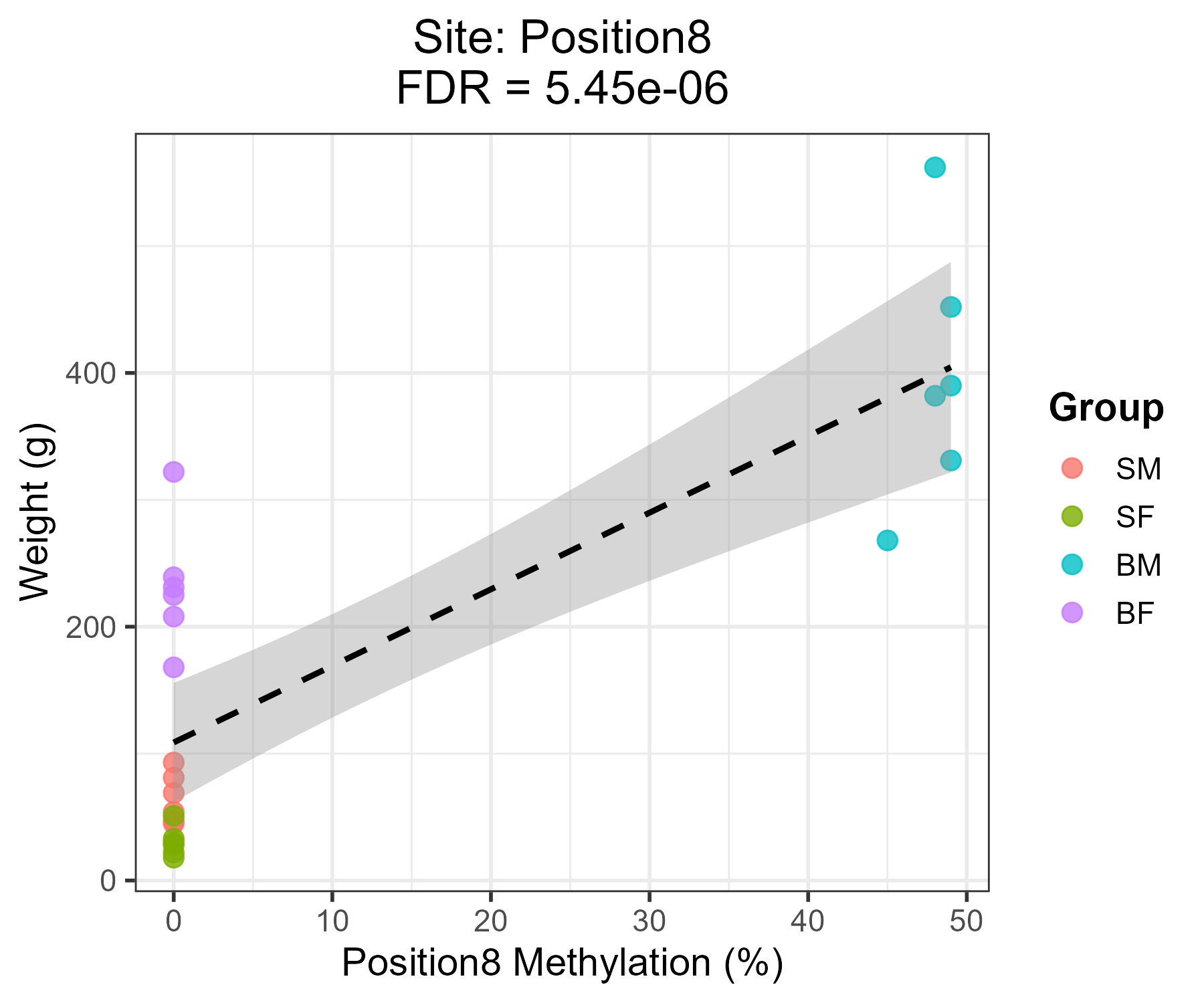

Supplement: Supplementary file 4 [file DataSheet2.zip › Regression_Minus_Strand/Position8_regression.tiff]

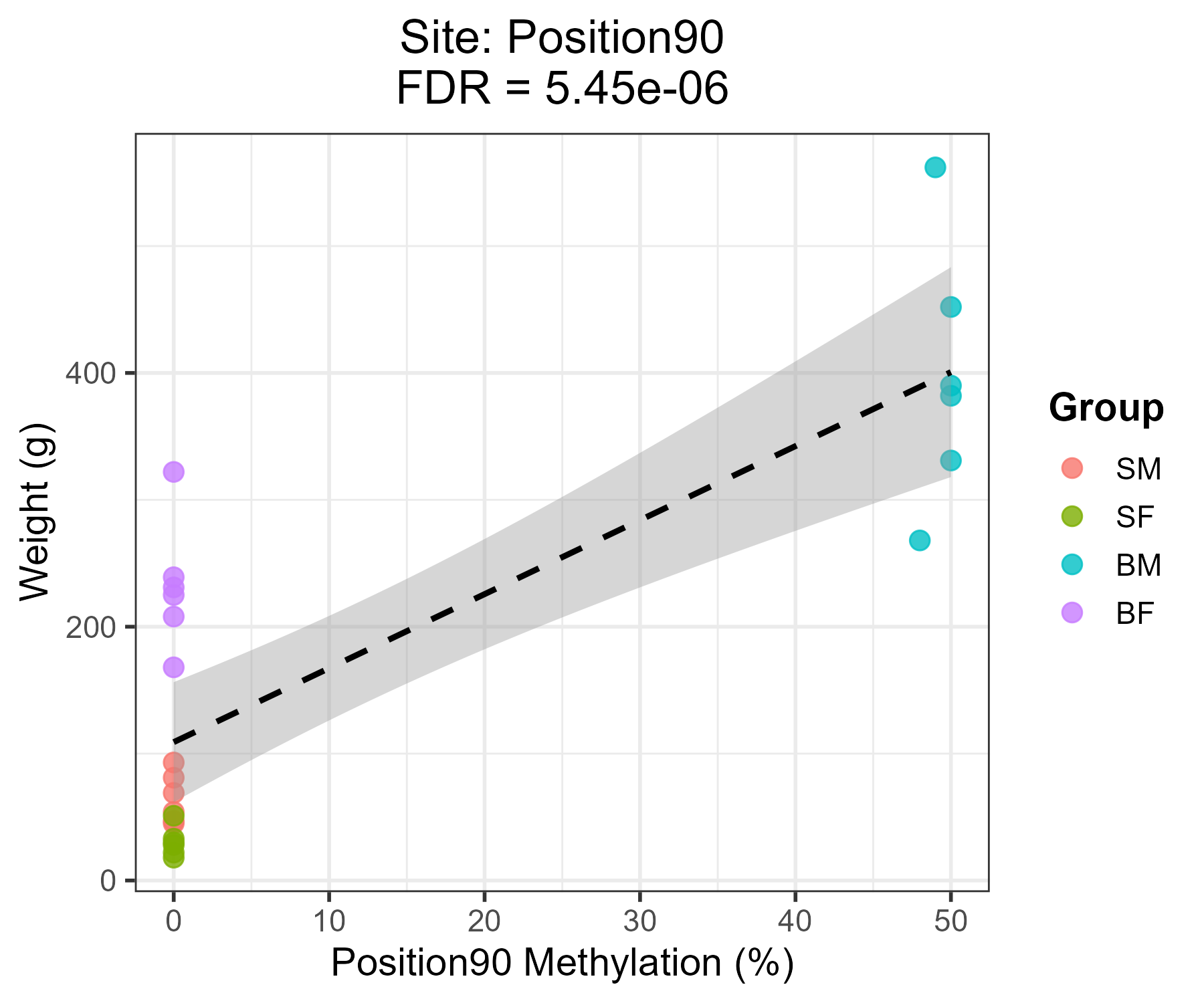

Supplement: Supplementary file 4 [file DataSheet2.zip › Regression_Minus_Strand/Position90_regression.tiff]

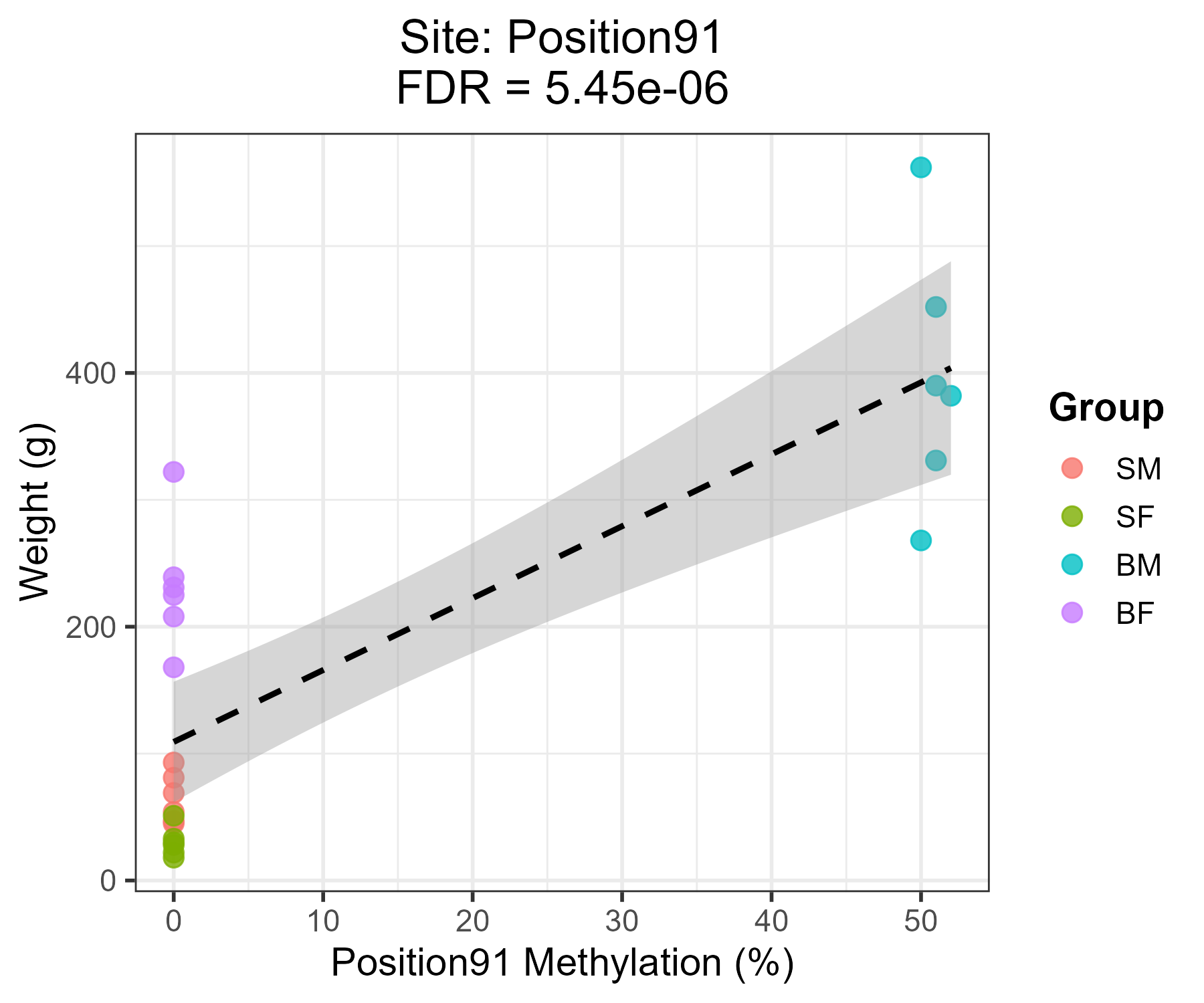

Supplement: Supplementary file 4 [file DataSheet2.zip › Regression_Minus_Strand/Position91_regression.tiff]

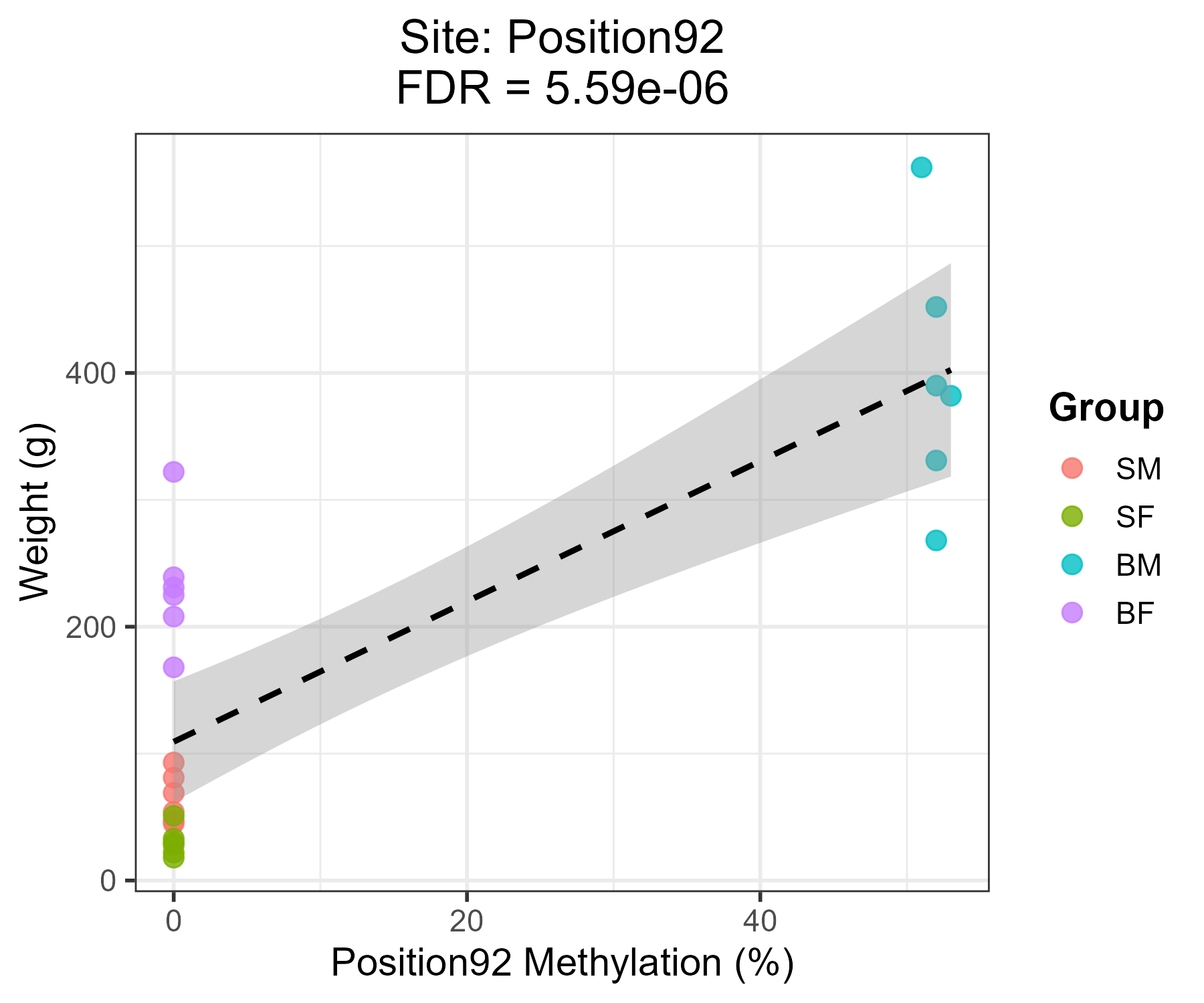

Supplement: Supplementary file 4 [file DataSheet2.zip › Regression_Minus_Strand/Position92_regression.tiff]

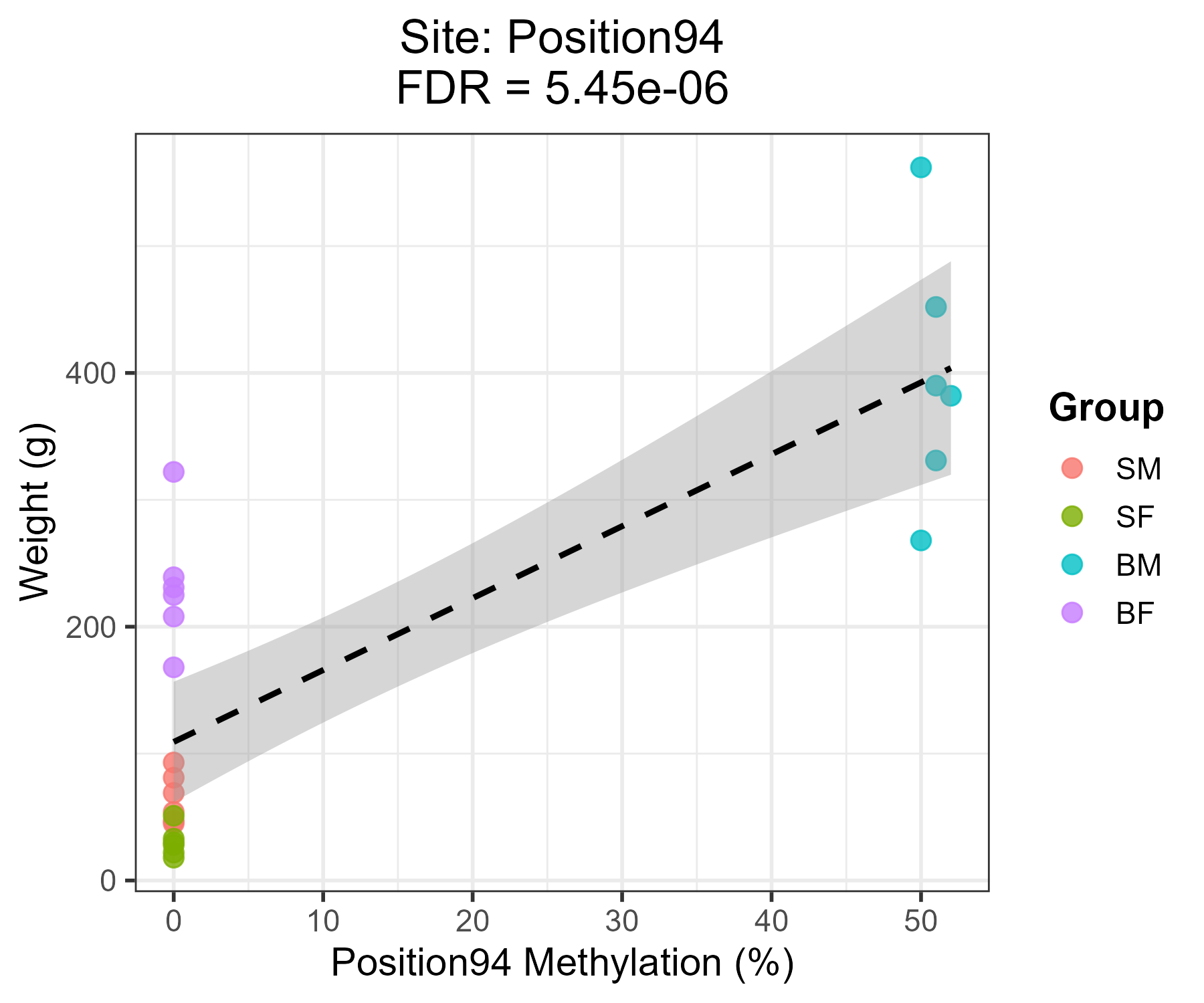

Supplement: Supplementary file 4 [file DataSheet2.zip › Regression_Minus_Strand/Position94_regression.tiff]

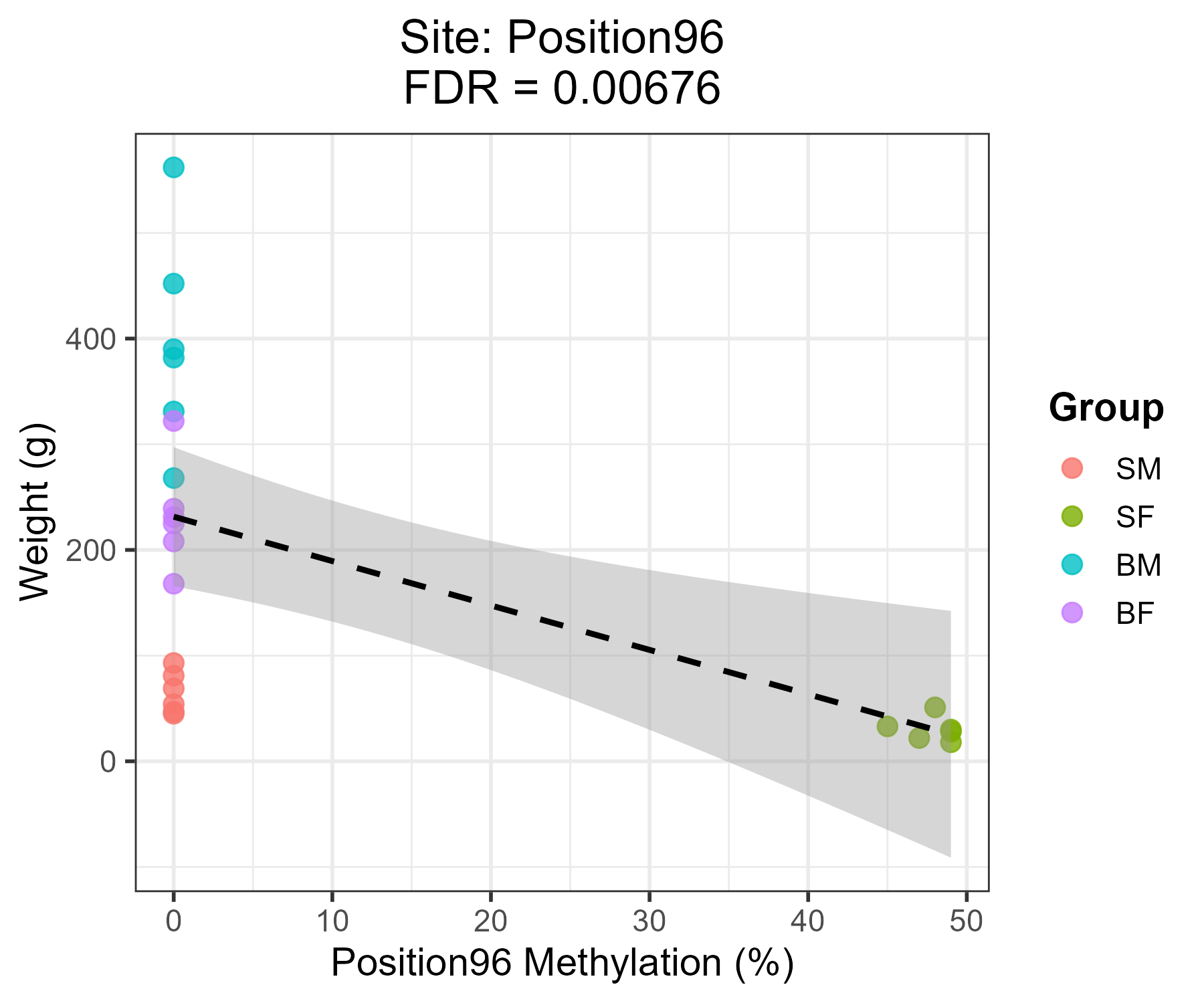

Supplement: Supplementary file 4 [file DataSheet2.zip › Regression_Minus_Strand/Position96_regression.tiff]

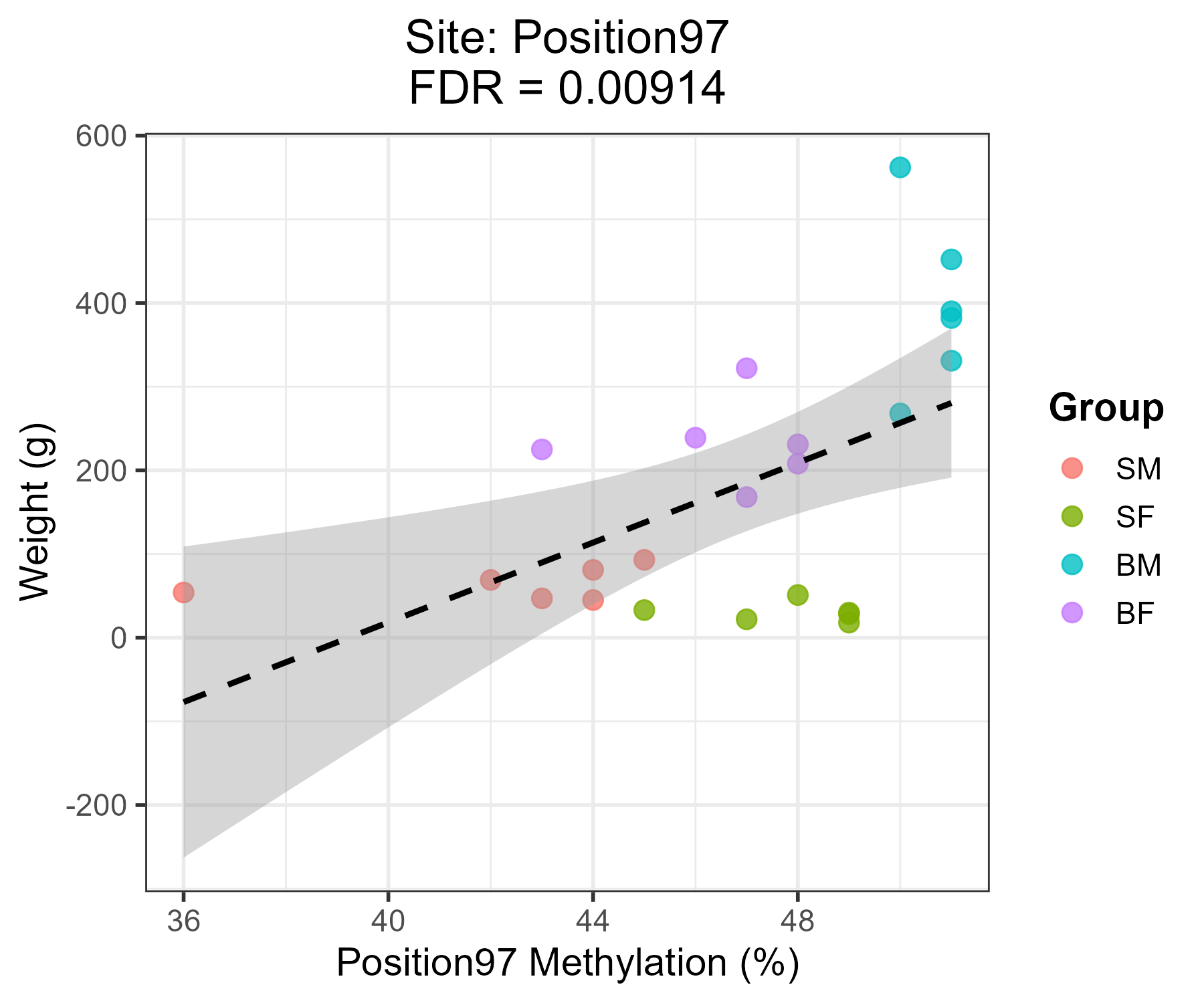

Supplement: Supplementary file 4 [file DataSheet2.zip › Regression_Minus_Strand/Position97_regression.tiff]

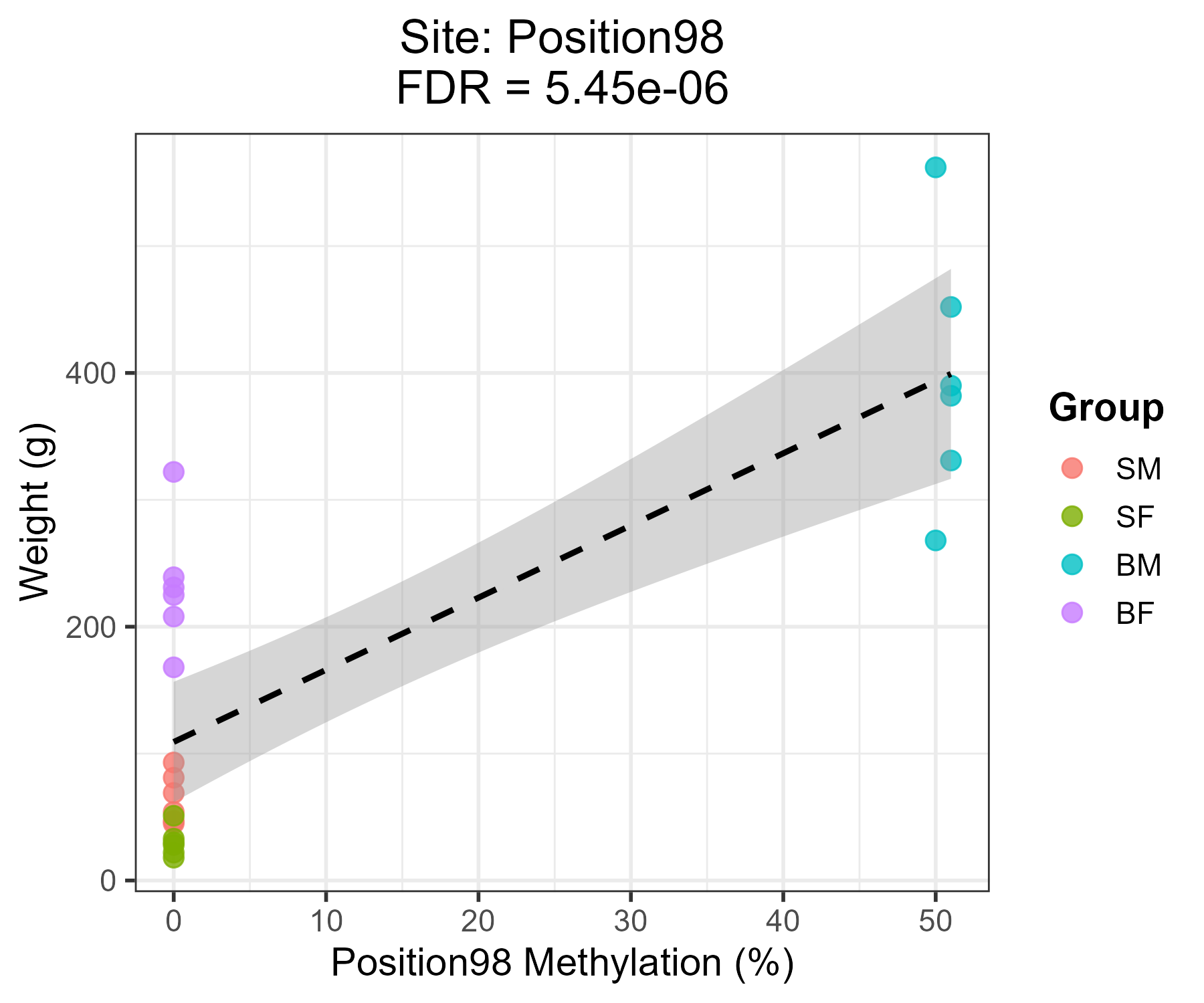

Supplement: Supplementary file 4 [file DataSheet2.zip › Regression_Minus_Strand/Position98_regression.tiff]

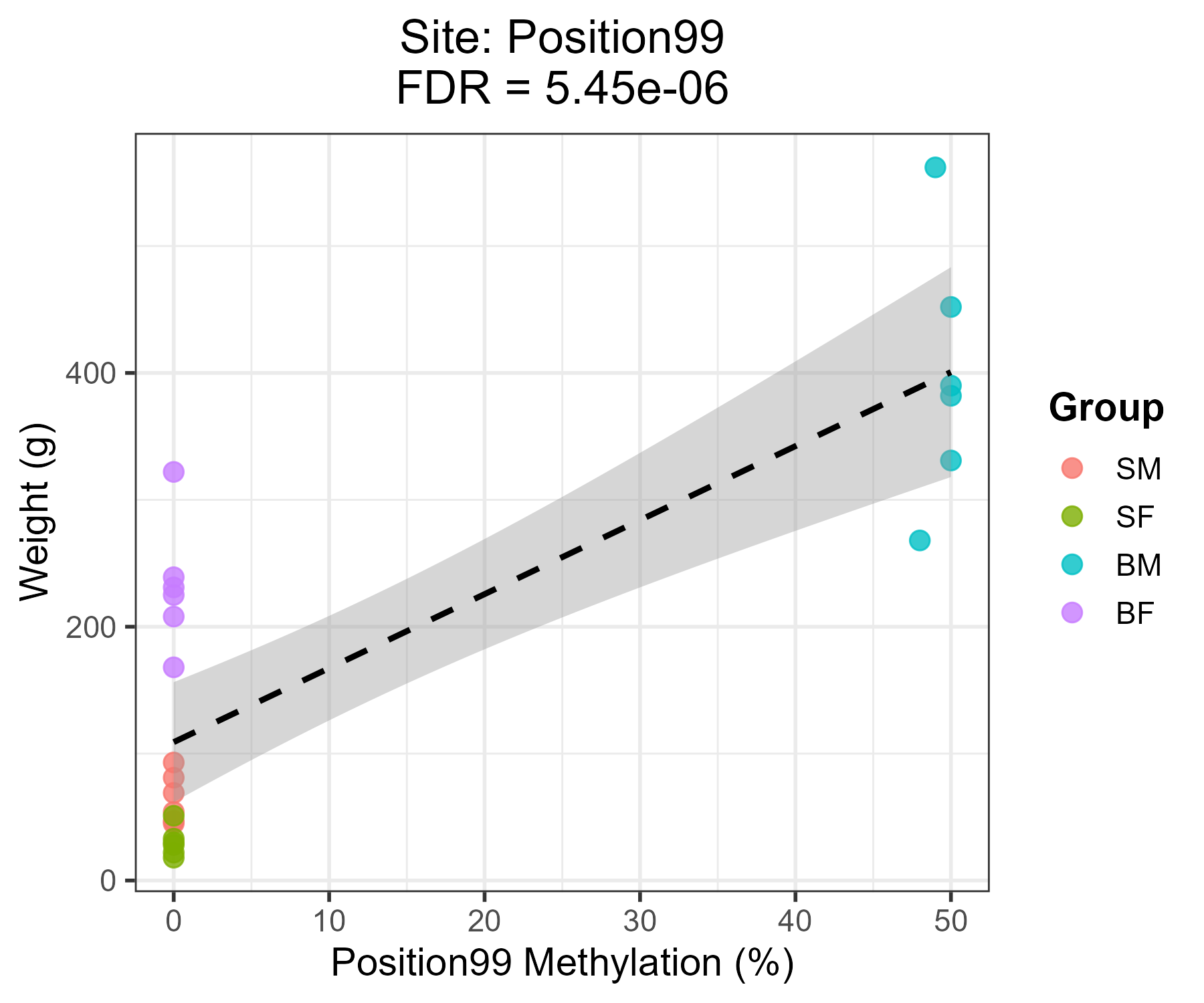

Supplement: Supplementary file 4 [file DataSheet2.zip › Regression_Minus_Strand/Position99_regression.tiff]

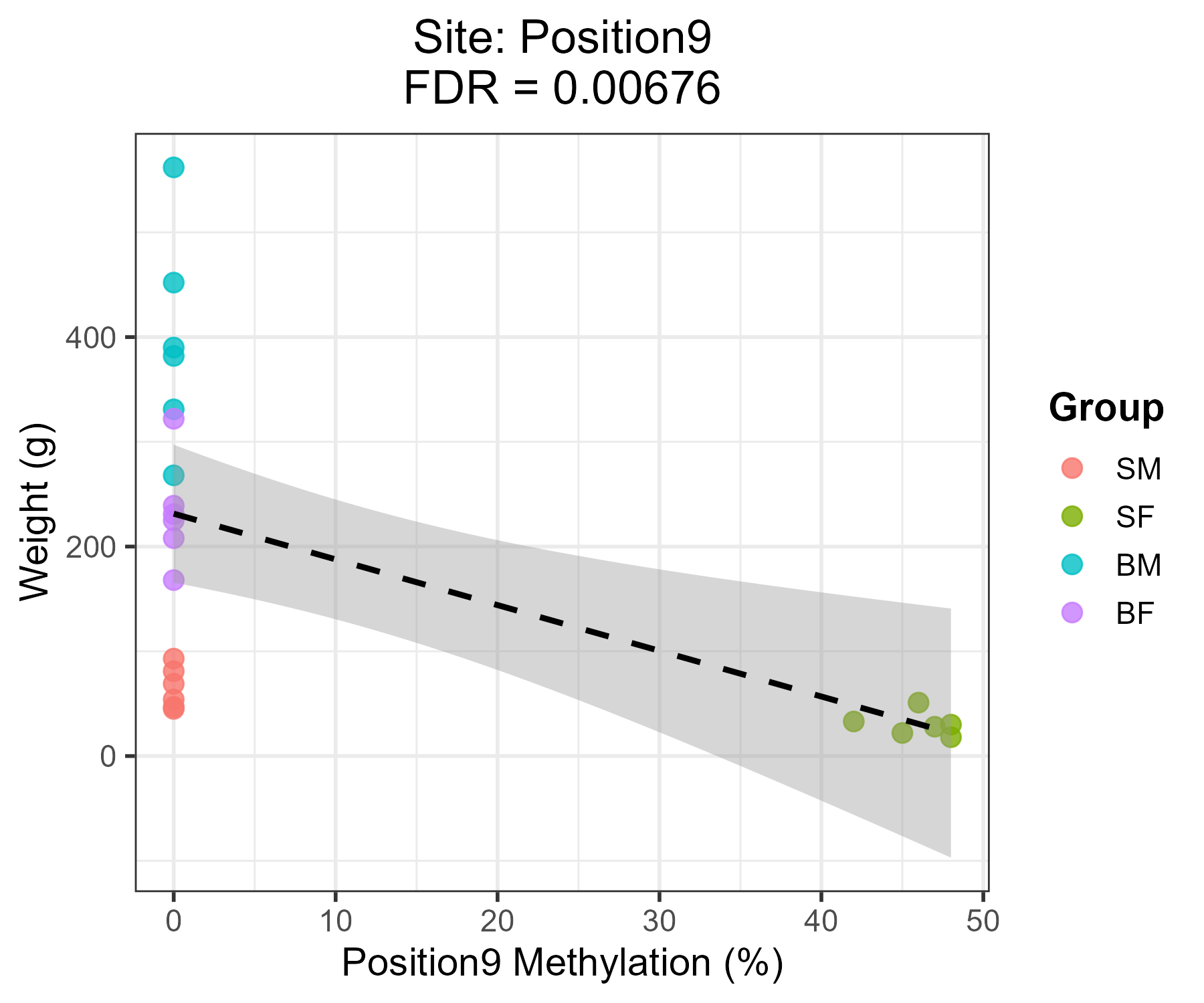

Supplement: Supplementary file 4 [file DataSheet2.zip › Regression_Minus_Strand/Position9_regression.tiff]
