# Supplementary figures and images for: Mitochondrial methylation is linked to sexually dimorphic growth in Nile tilapia (Oreochromis niloticus) (part 2 of 3)
Source: Front Cell Dev Biol. 2025 Aug 5;13:1643817. doi: 10.3389/fcell.2025.1643817 (PMC12361127; doi:10.3389/fcell.2025.1643817)

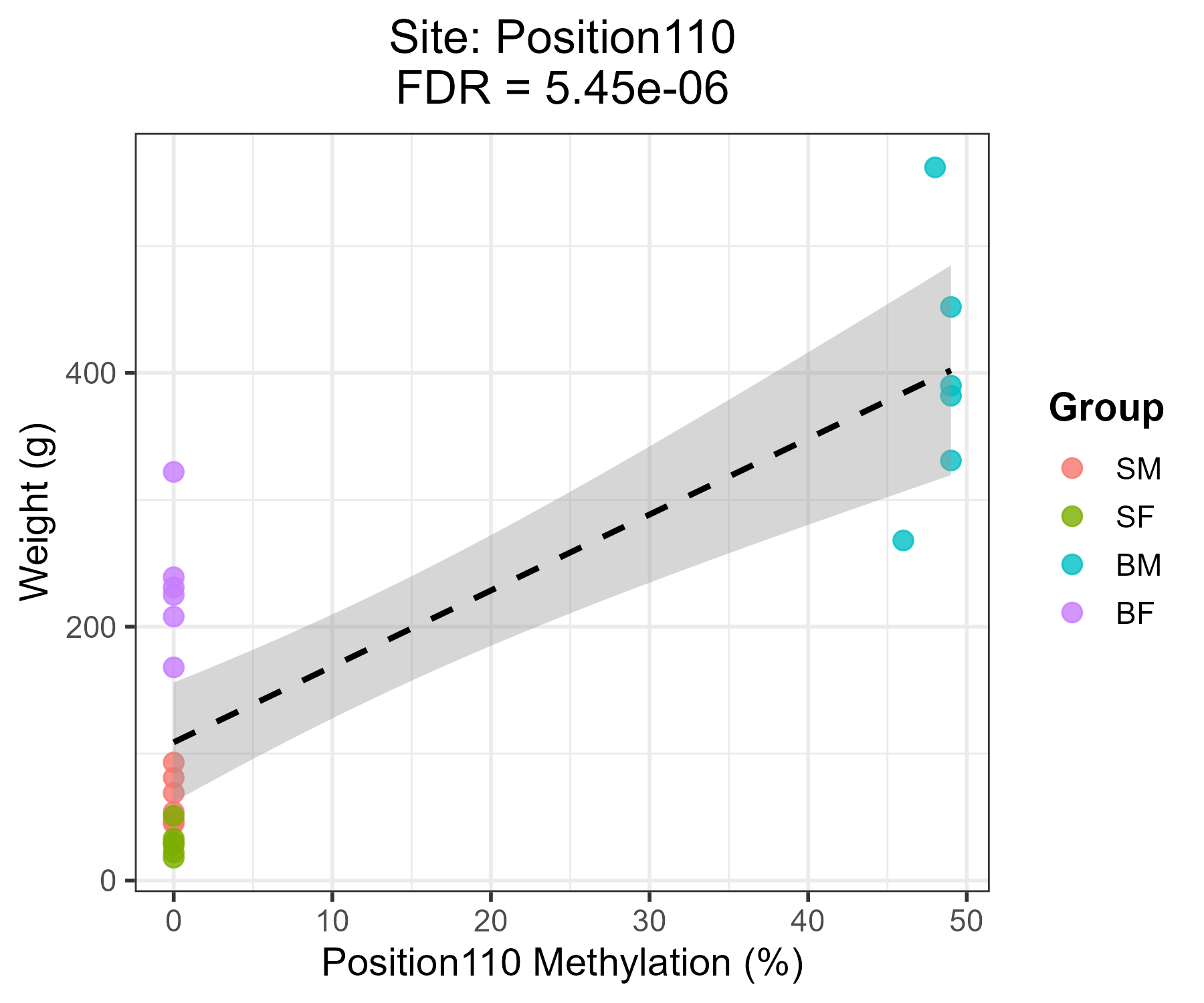

Supplement: Supplementary file 4 [file DataSheet2.zip › Regression_Minus_Strand/Position110_regression.tiff]

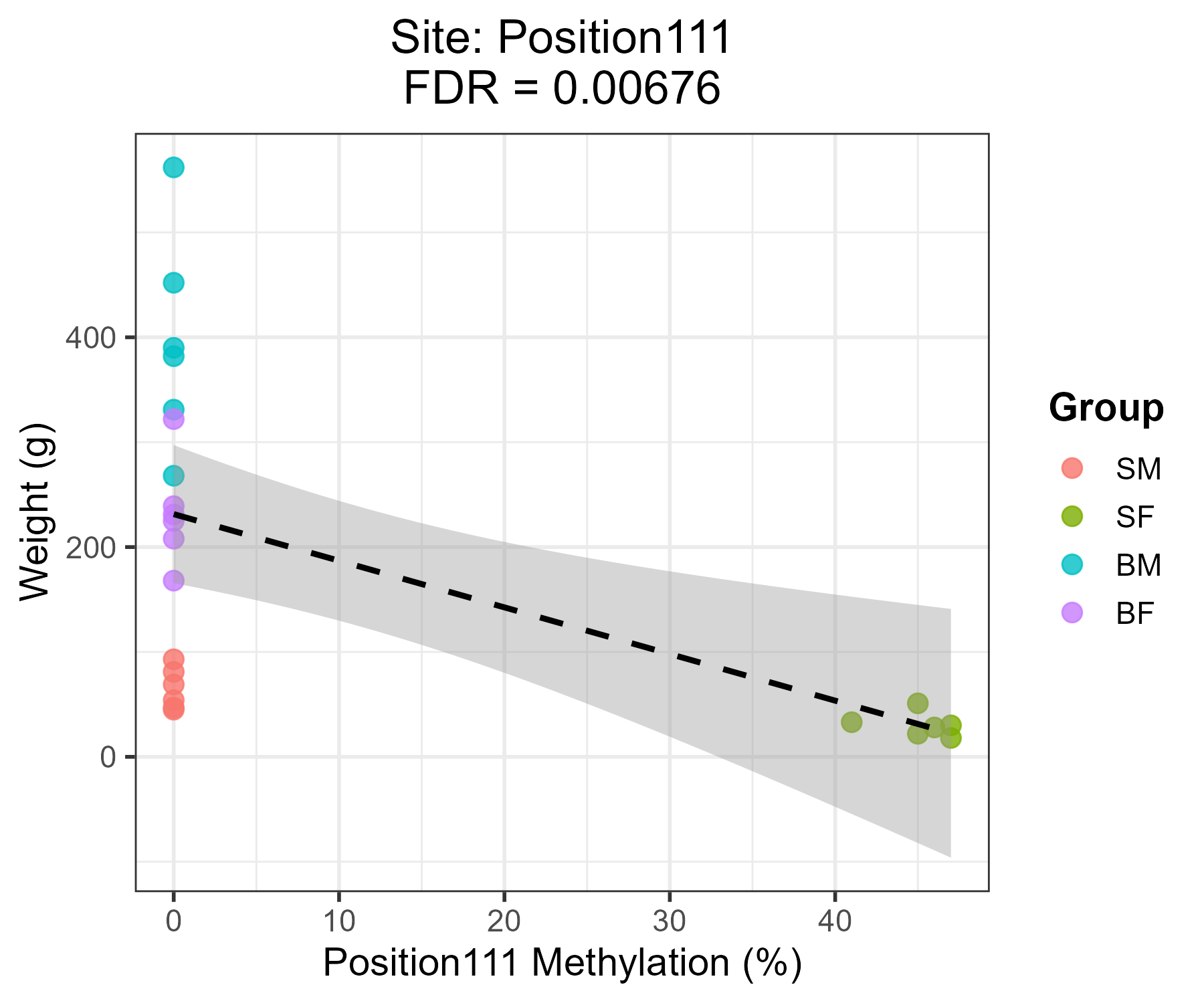

Supplement: Supplementary file 4 [file DataSheet2.zip › Regression_Minus_Strand/Position111_regression.tiff]

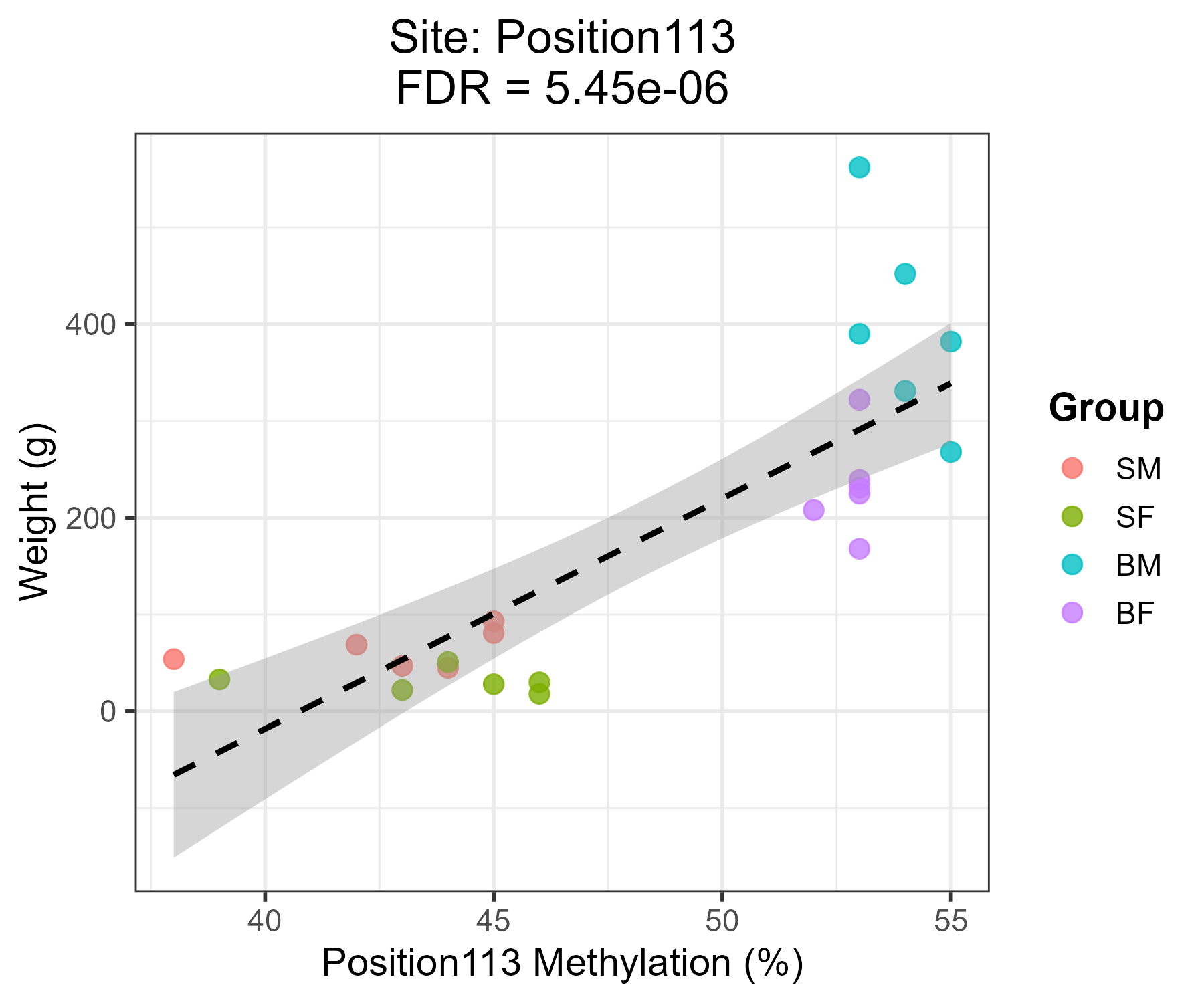

Supplement: Supplementary file 4 [file DataSheet2.zip › Regression_Minus_Strand/Position113_regression.tiff]

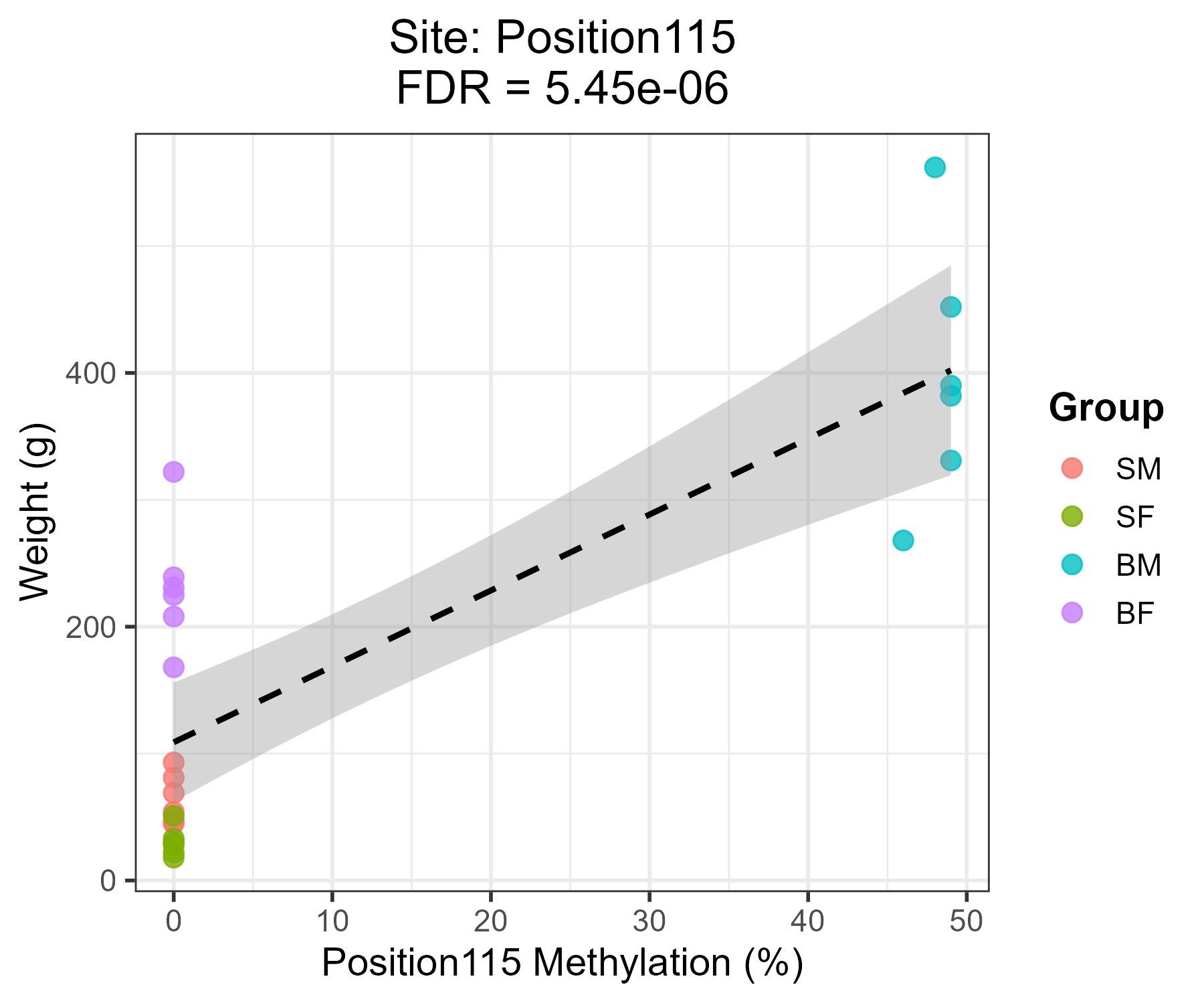

Supplement: Supplementary file 4 [file DataSheet2.zip › Regression_Minus_Strand/Position115_regression.tiff]

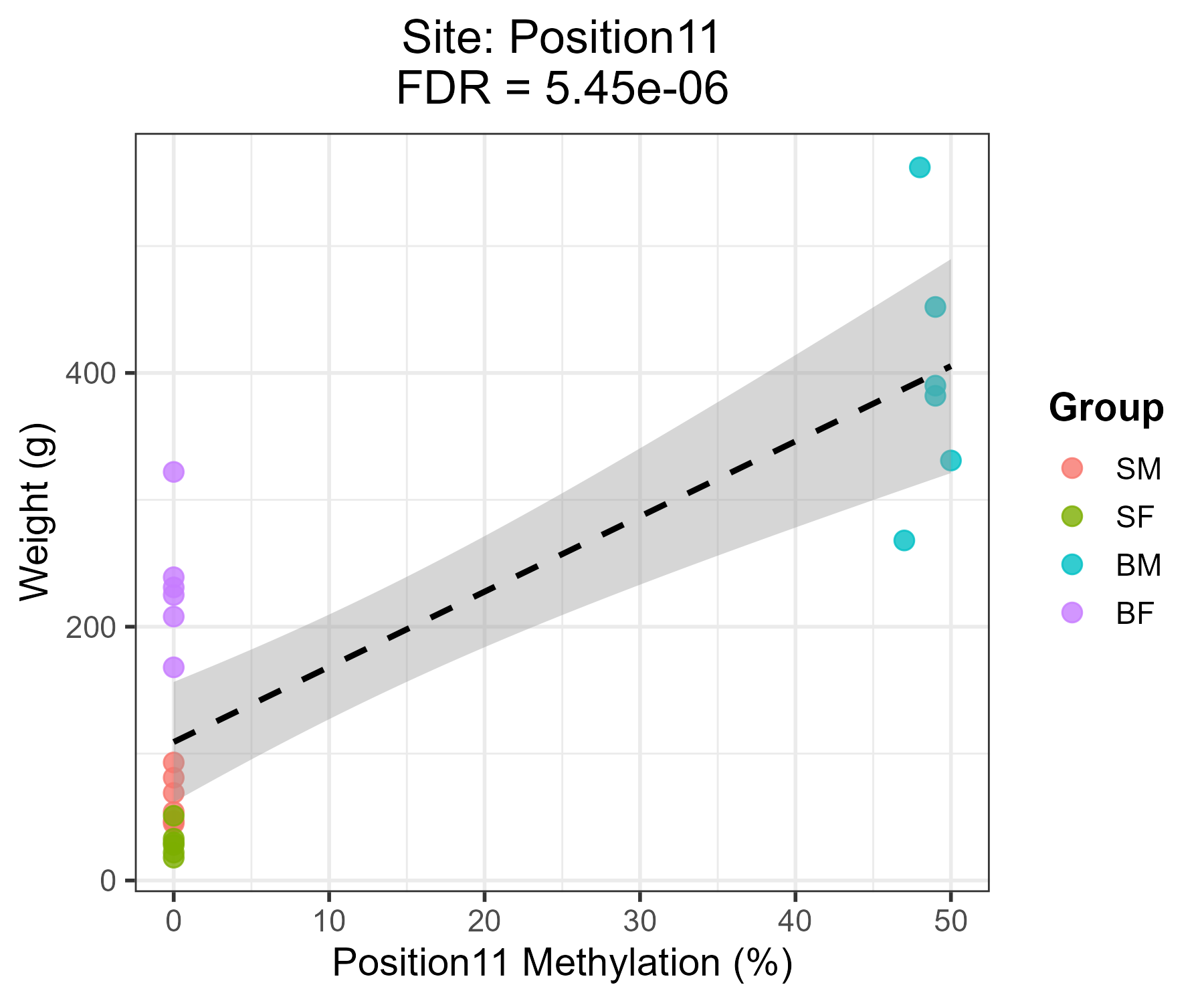

Supplement: Supplementary file 4 [file DataSheet2.zip › Regression_Minus_Strand/Position11_regression.tiff]

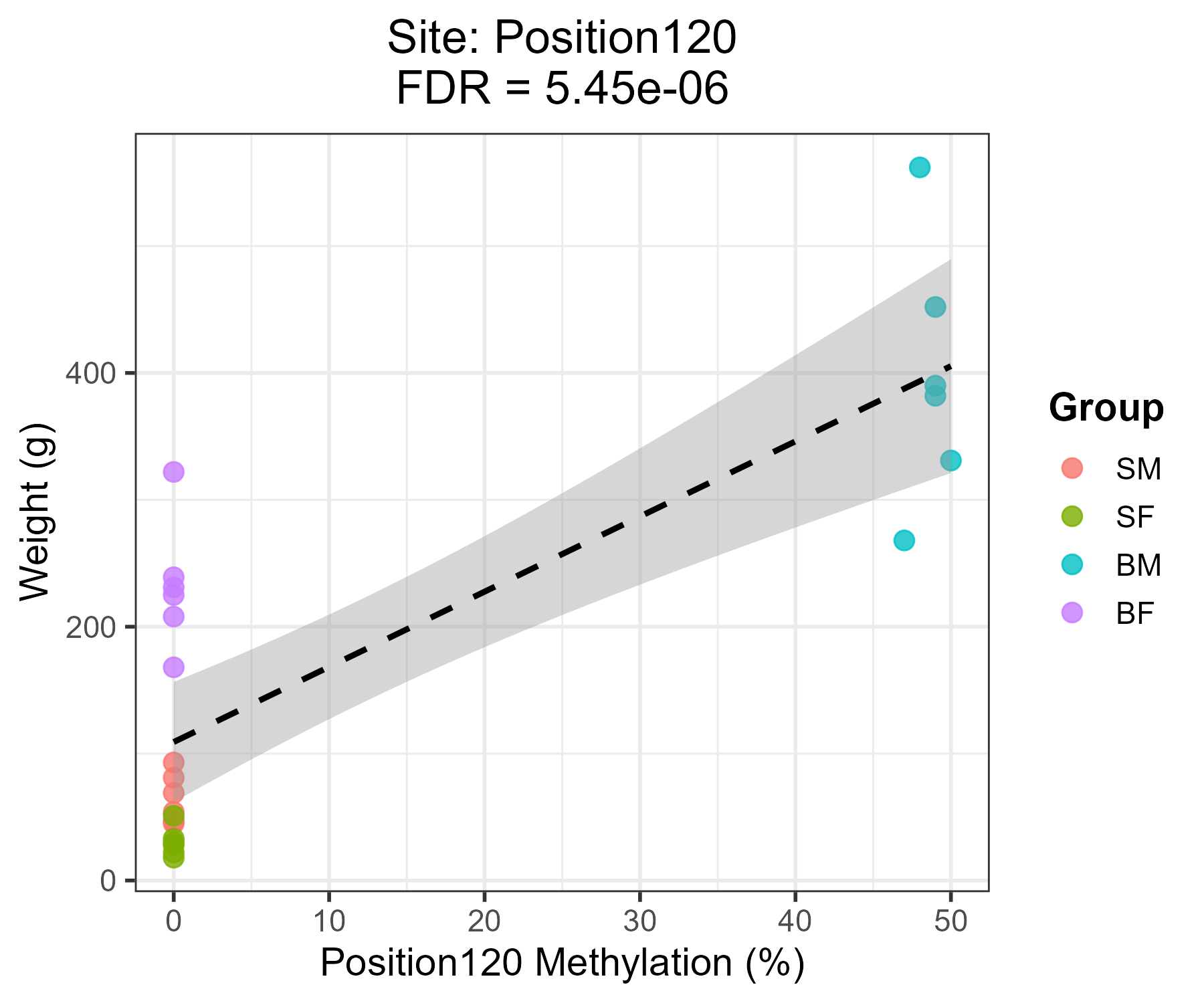

Supplement: Supplementary file 4 [file DataSheet2.zip › Regression_Minus_Strand/Position120_regression.tiff]

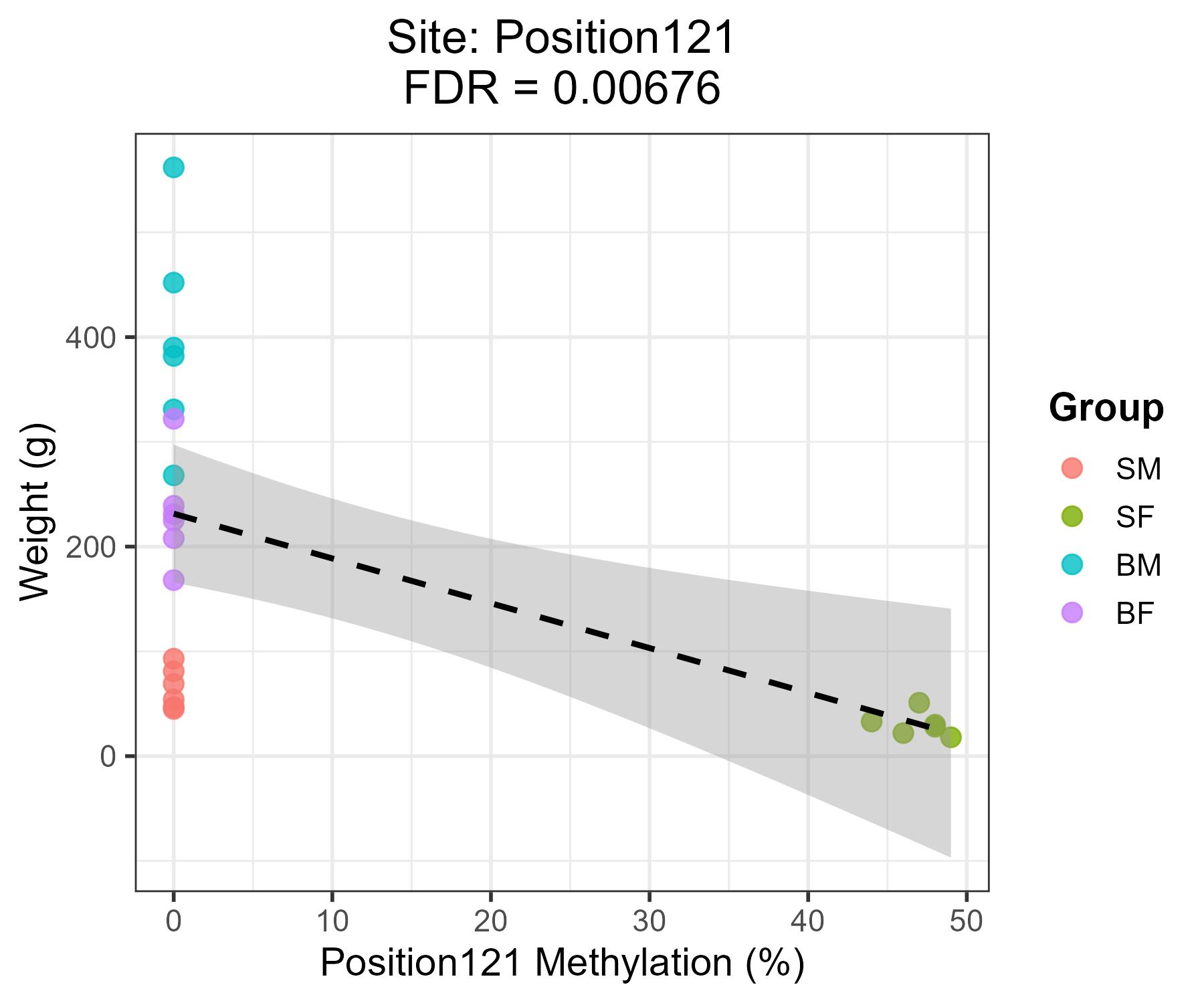

Supplement: Supplementary file 4 [file DataSheet2.zip › Regression_Minus_Strand/Position121_regression.tiff]

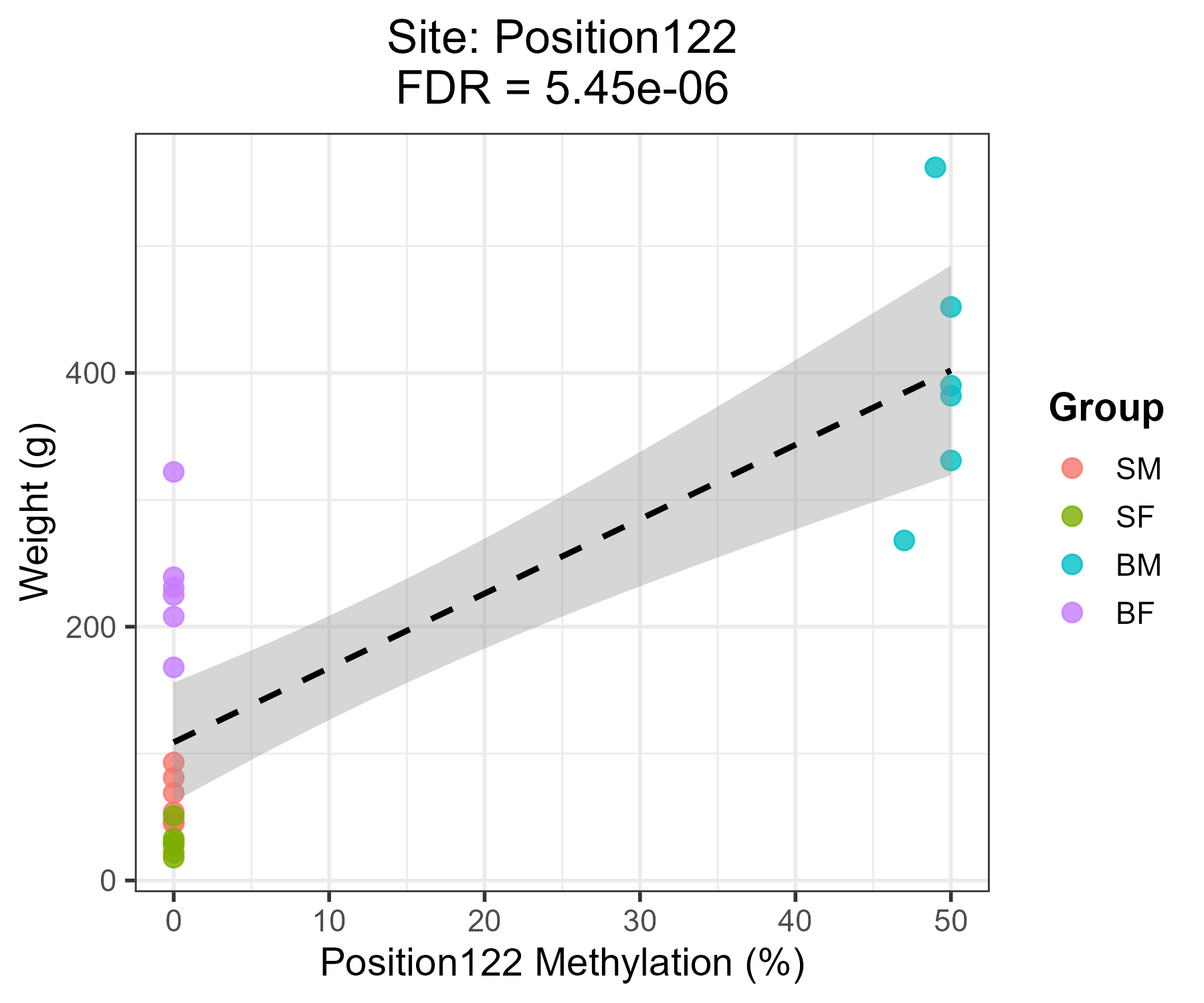

Supplement: Supplementary file 4 [file DataSheet2.zip › Regression_Minus_Strand/Position122_regression.tiff]

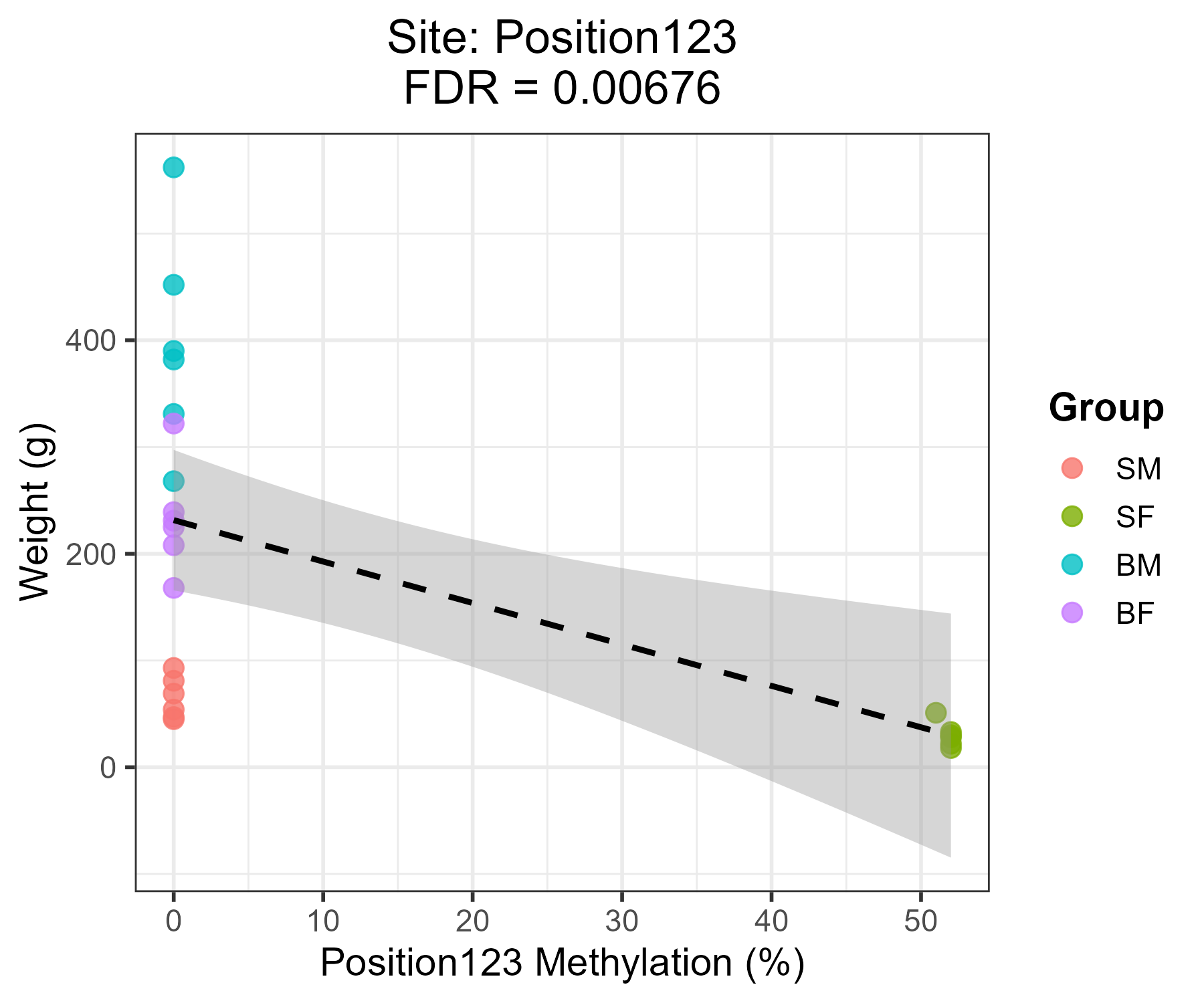

Supplement: Supplementary file 4 [file DataSheet2.zip › Regression_Minus_Strand/Position123_regression.tiff]

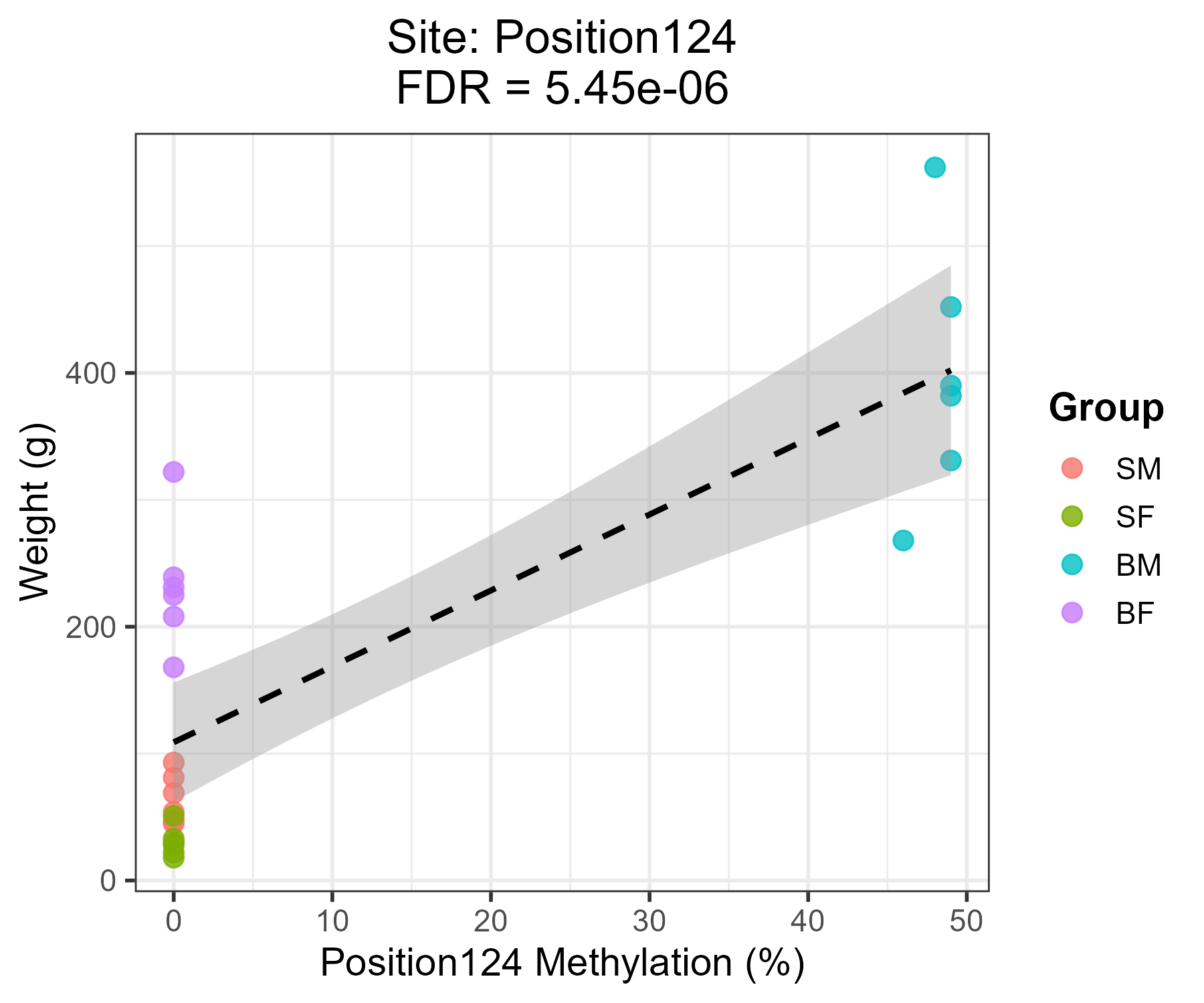

Supplement: Supplementary file 4 [file DataSheet2.zip › Regression_Minus_Strand/Position124_regression.tiff]

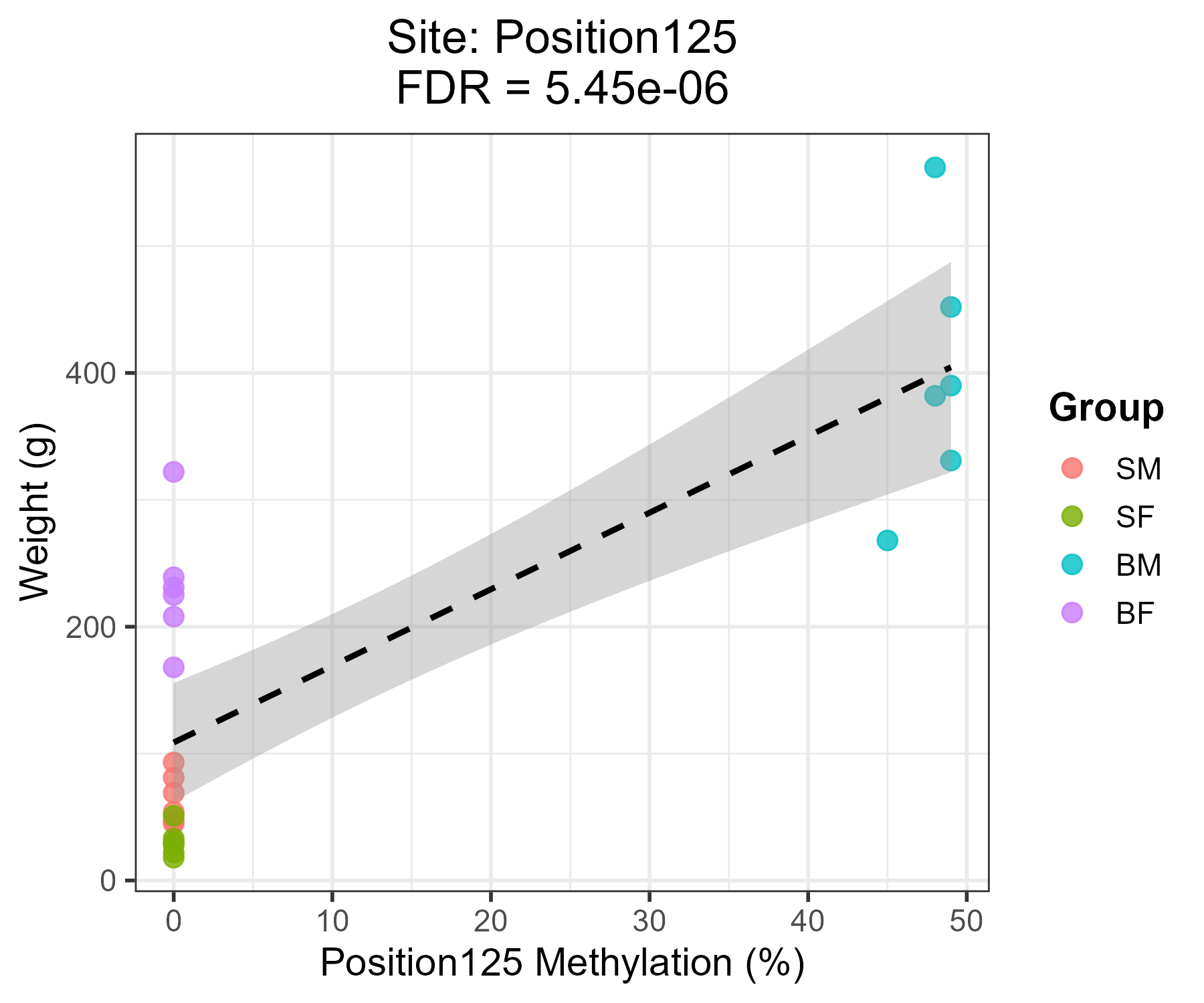

Supplement: Supplementary file 4 [file DataSheet2.zip › Regression_Minus_Strand/Position125_regression.tiff]

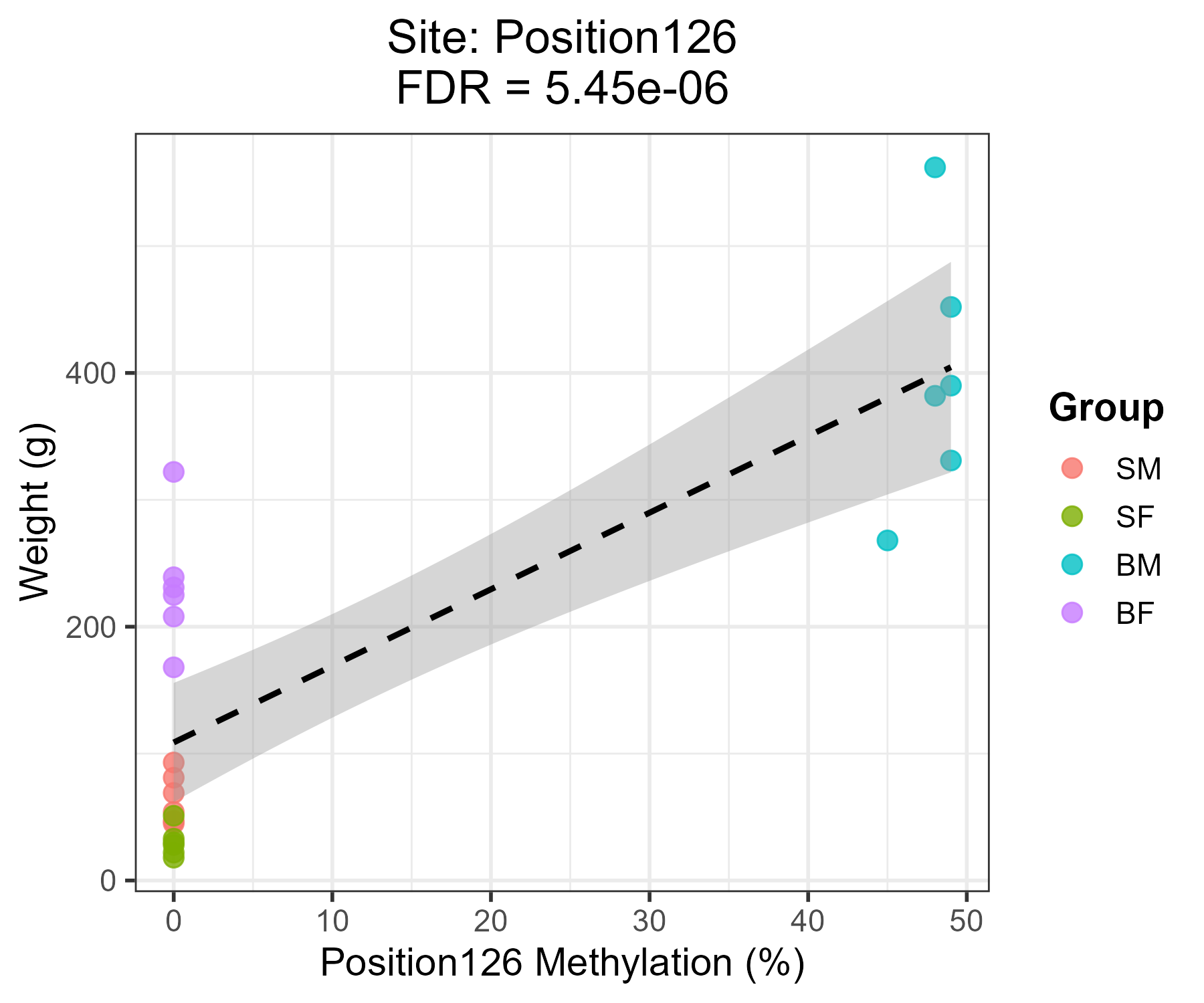

Supplement: Supplementary file 4 [file DataSheet2.zip › Regression_Minus_Strand/Position126_regression.tiff]

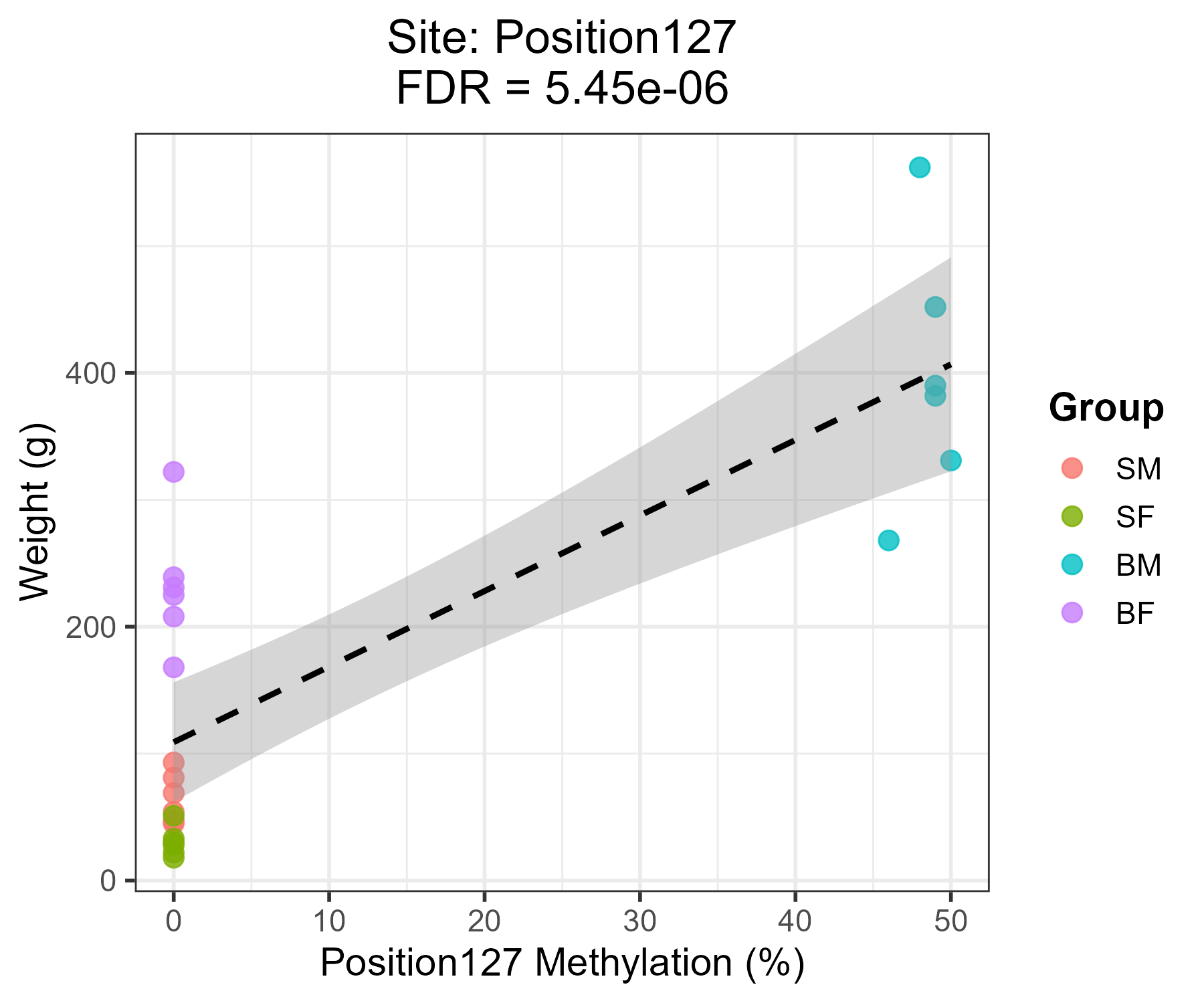

Supplement: Supplementary file 4 [file DataSheet2.zip › Regression_Minus_Strand/Position127_regression.tiff]

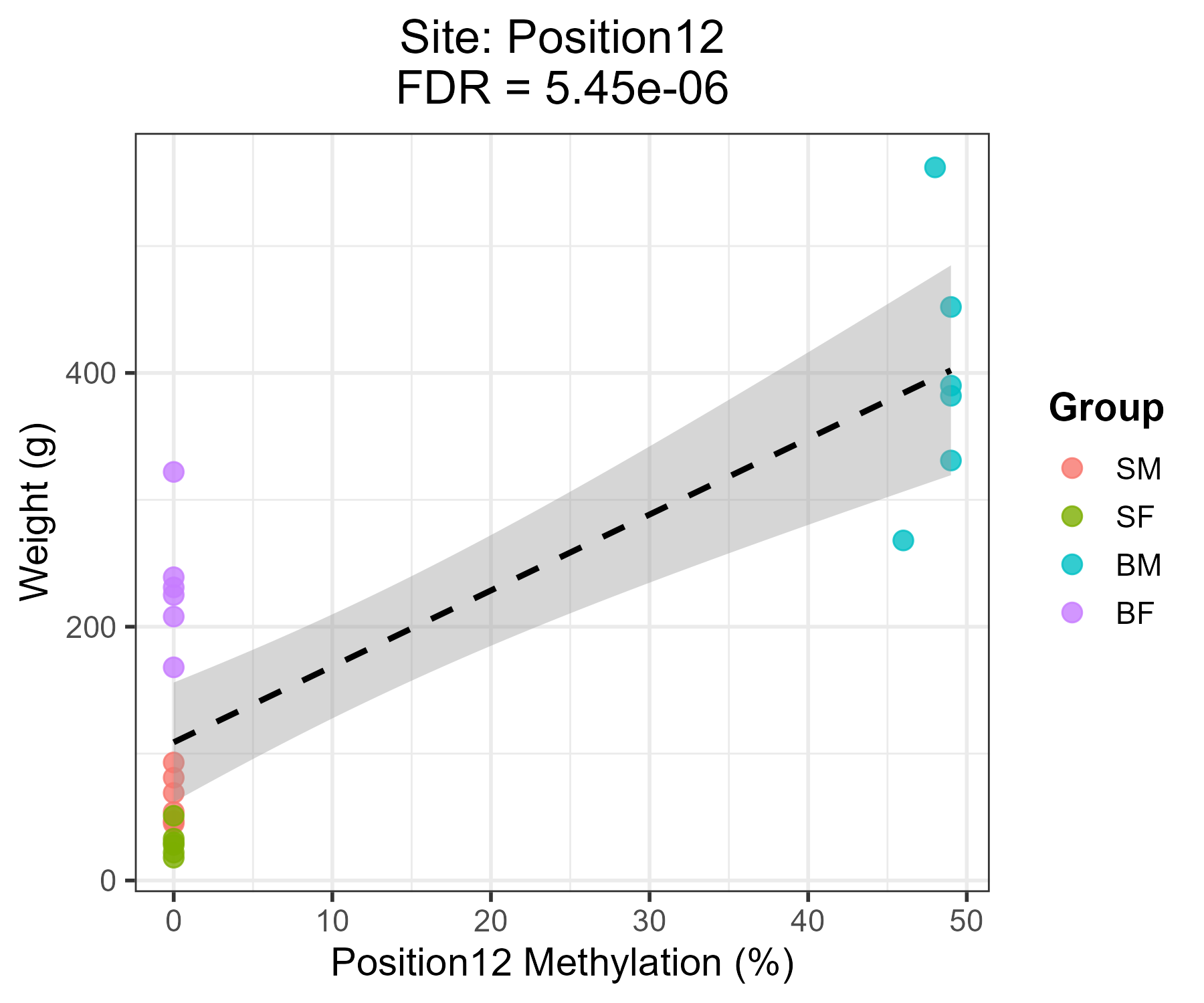

Supplement: Supplementary file 4 [file DataSheet2.zip › Regression_Minus_Strand/Position12_regression.tiff]

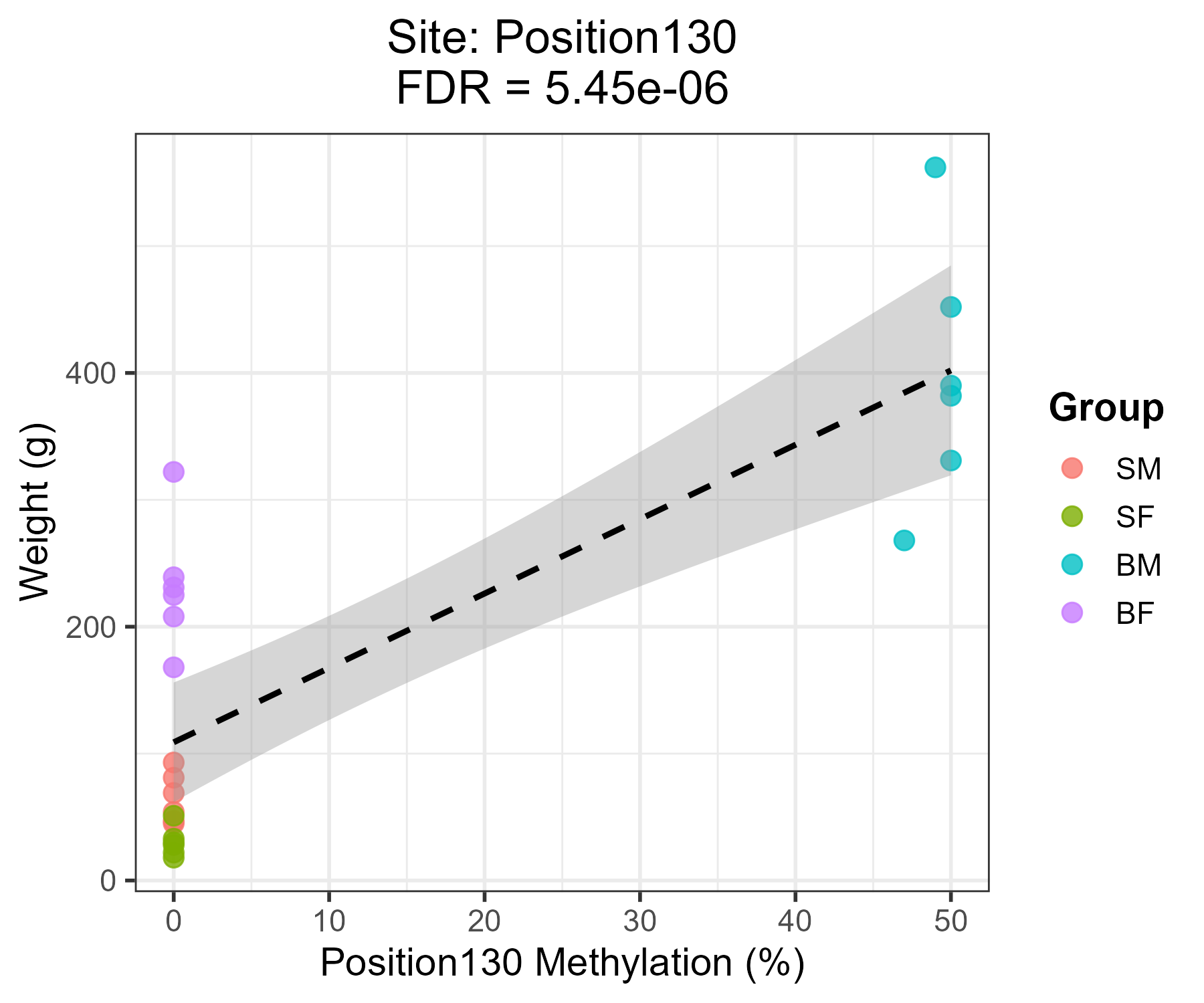

Supplement: Supplementary file 4 [file DataSheet2.zip › Regression_Minus_Strand/Position130_regression.tiff]

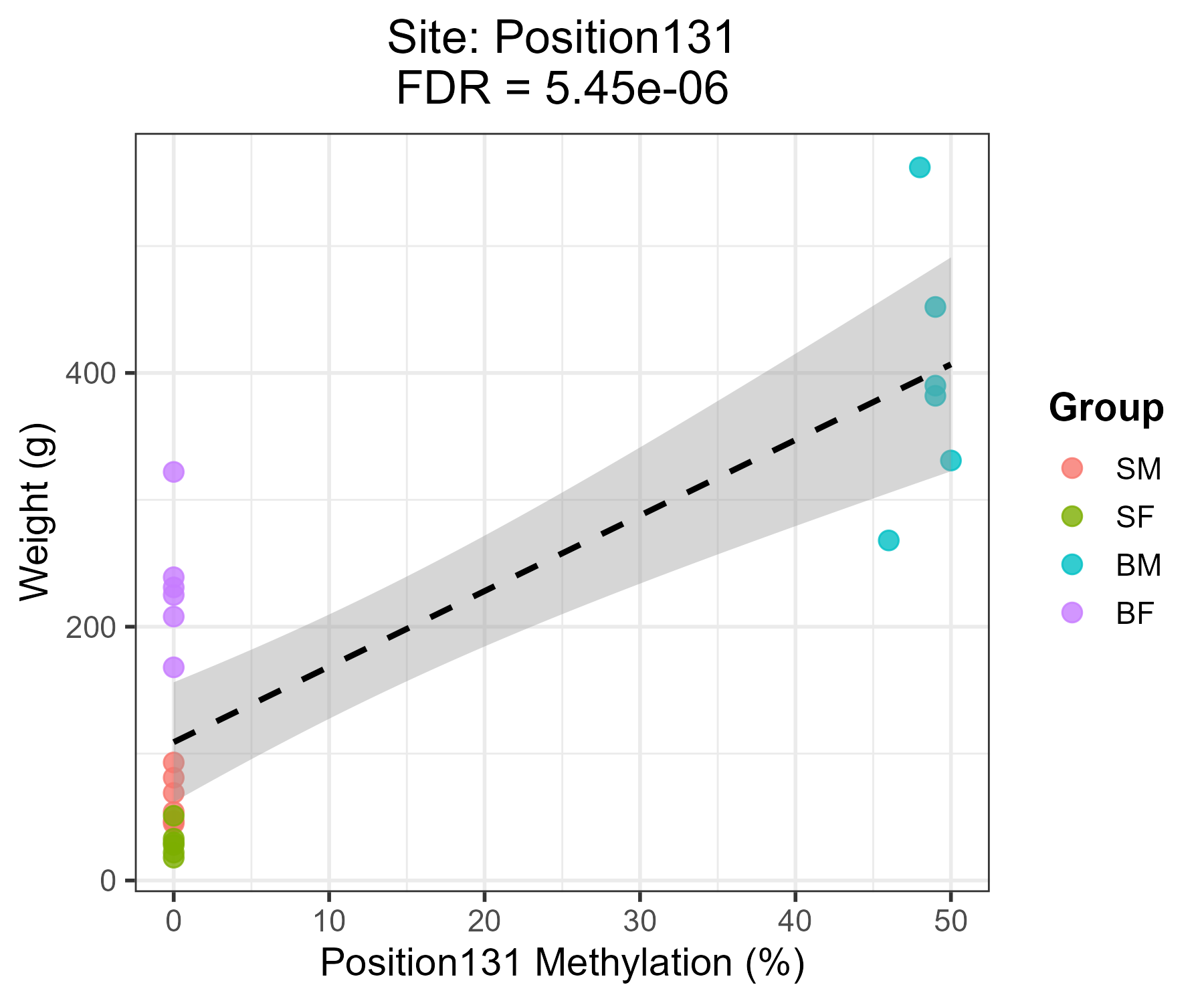

Supplement: Supplementary file 4 [file DataSheet2.zip › Regression_Minus_Strand/Position131_regression.tiff]

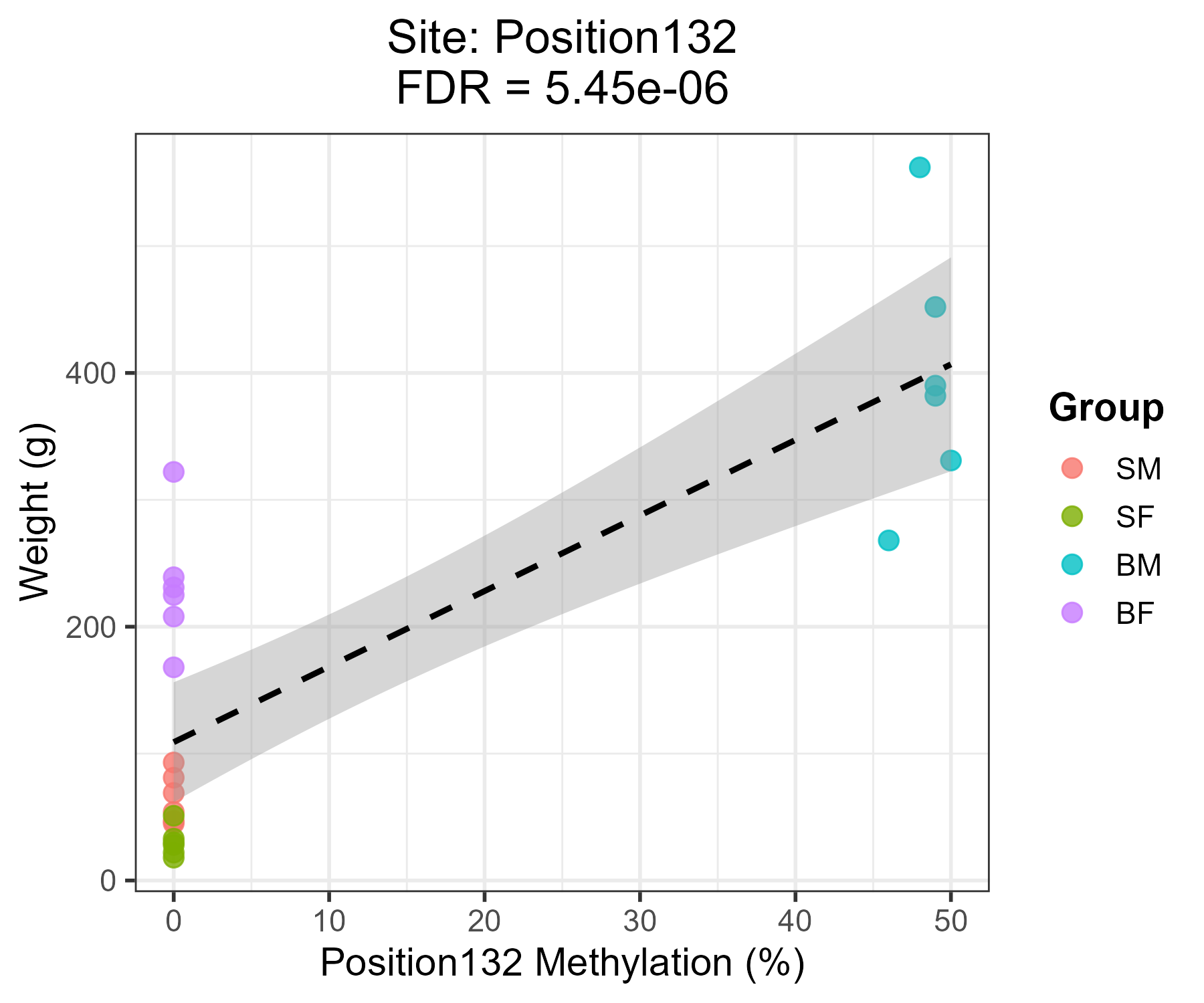

Supplement: Supplementary file 4 [file DataSheet2.zip › Regression_Minus_Strand/Position132_regression.tiff]

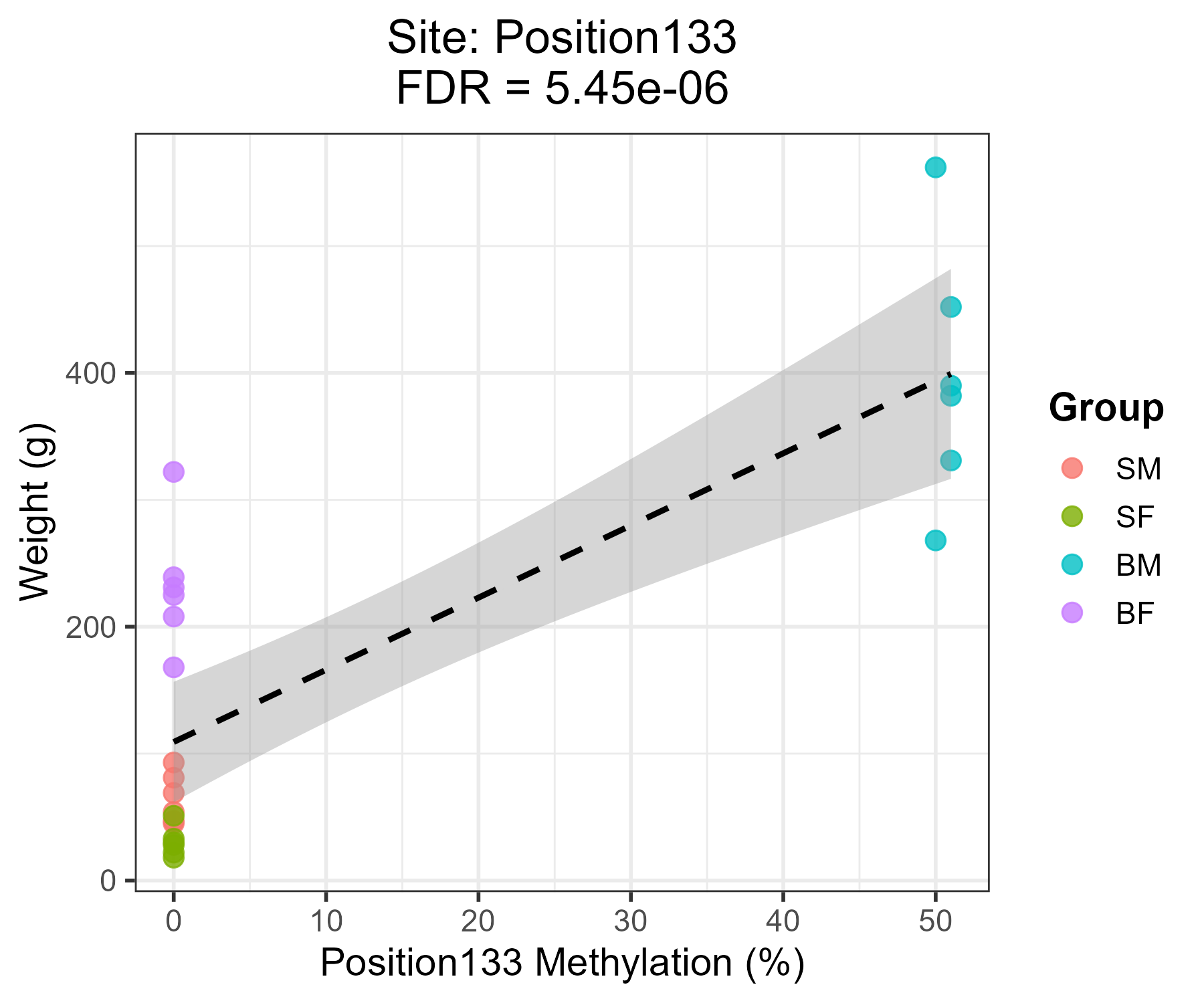

Supplement: Supplementary file 4 [file DataSheet2.zip › Regression_Minus_Strand/Position133_regression.tiff]

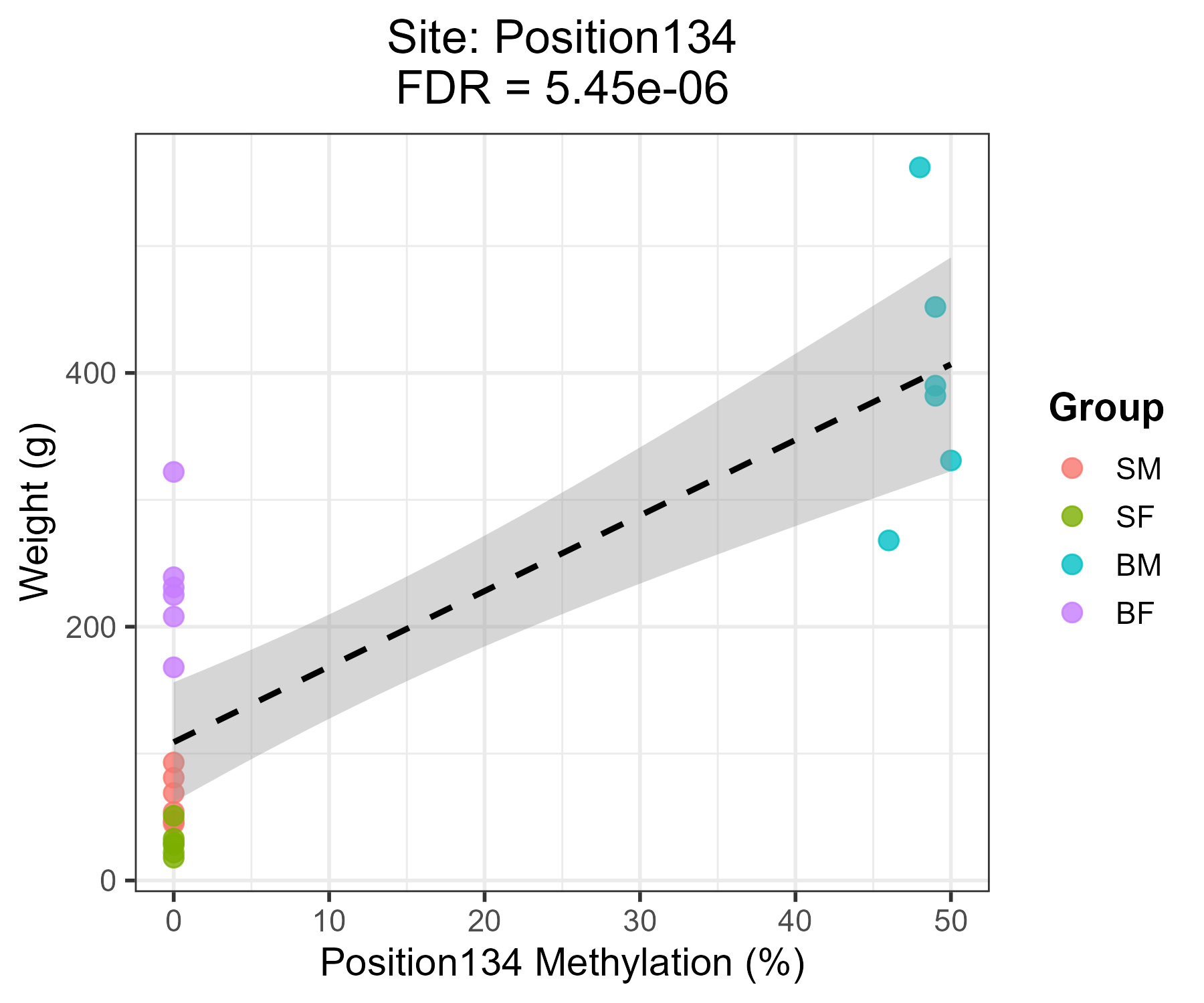

Supplement: Supplementary file 4 [file DataSheet2.zip › Regression_Minus_Strand/Position134_regression.tiff]

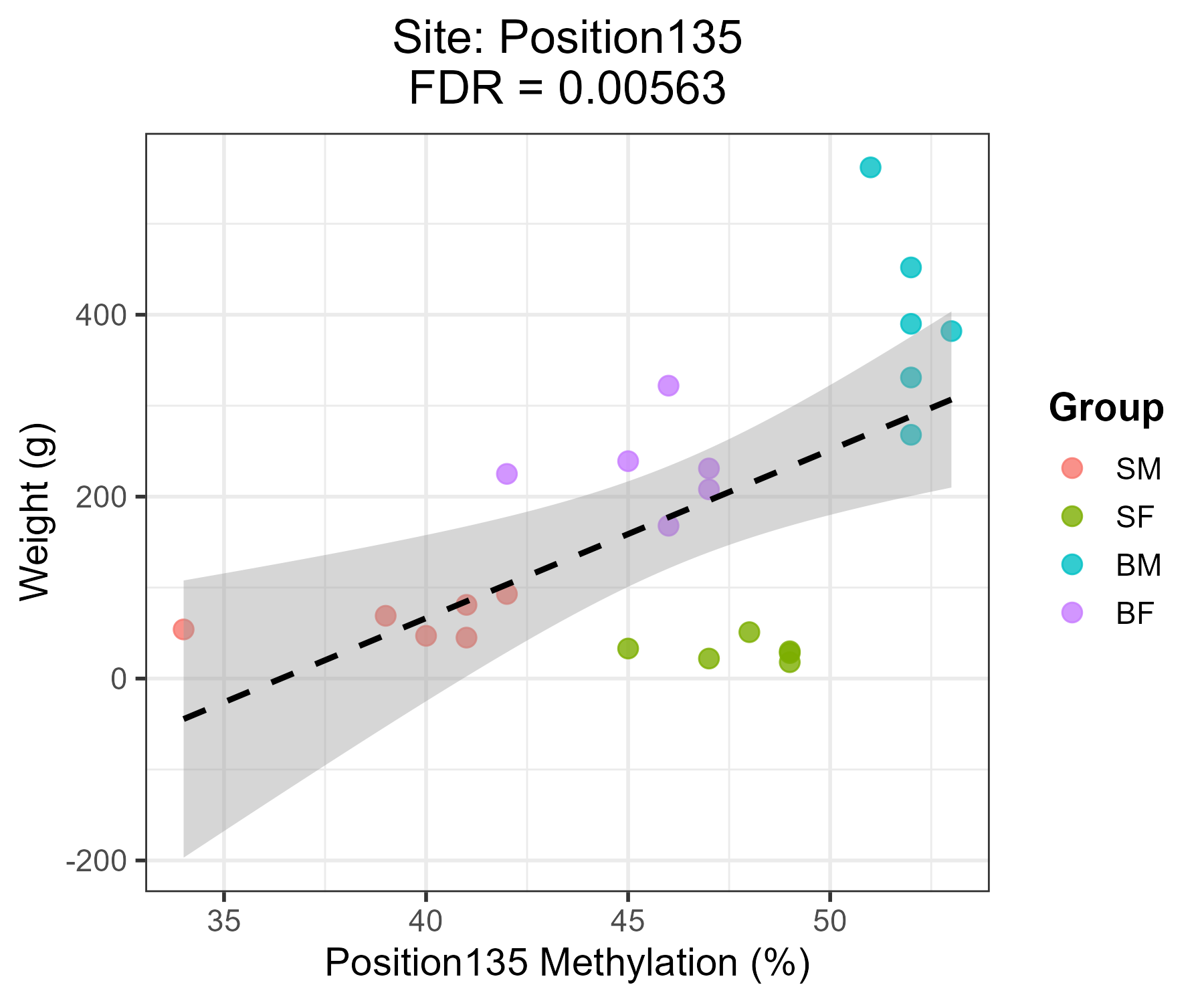

Supplement: Supplementary file 4 [file DataSheet2.zip › Regression_Minus_Strand/Position135_regression.tiff]

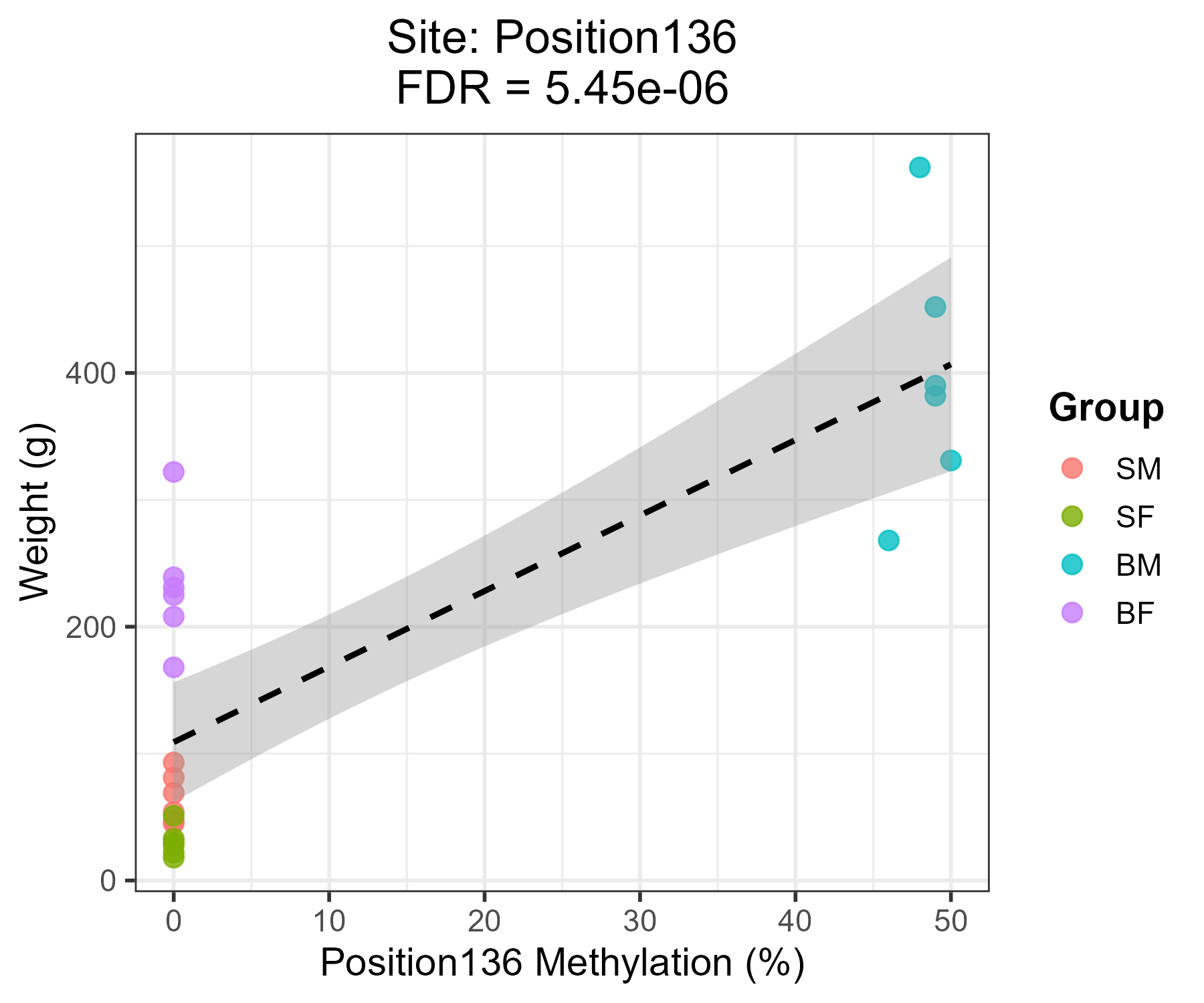

Supplement: Supplementary file 4 [file DataSheet2.zip › Regression_Minus_Strand/Position136_regression.tiff]

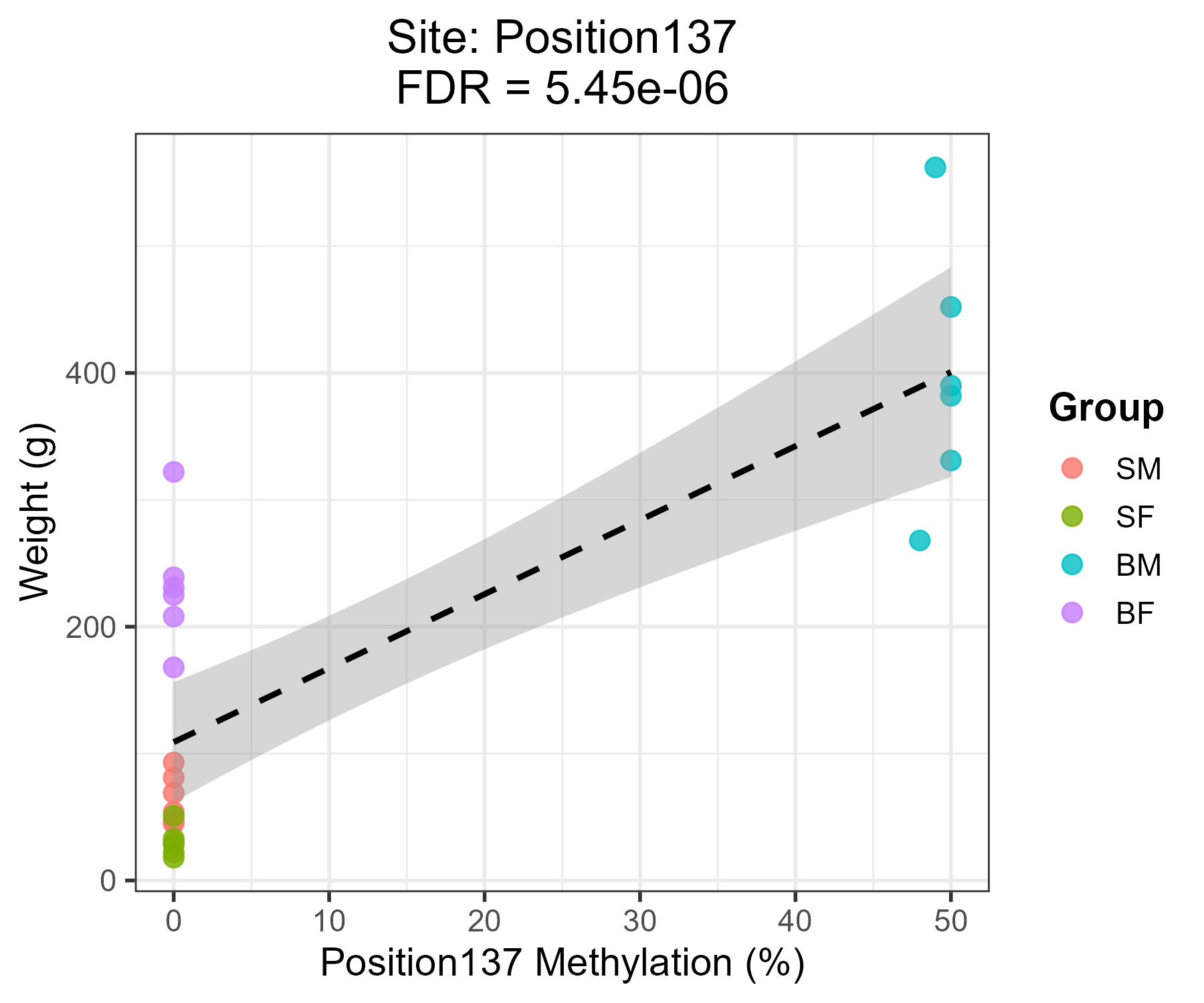

Supplement: Supplementary file 4 [file DataSheet2.zip › Regression_Minus_Strand/Position137_regression.tiff]

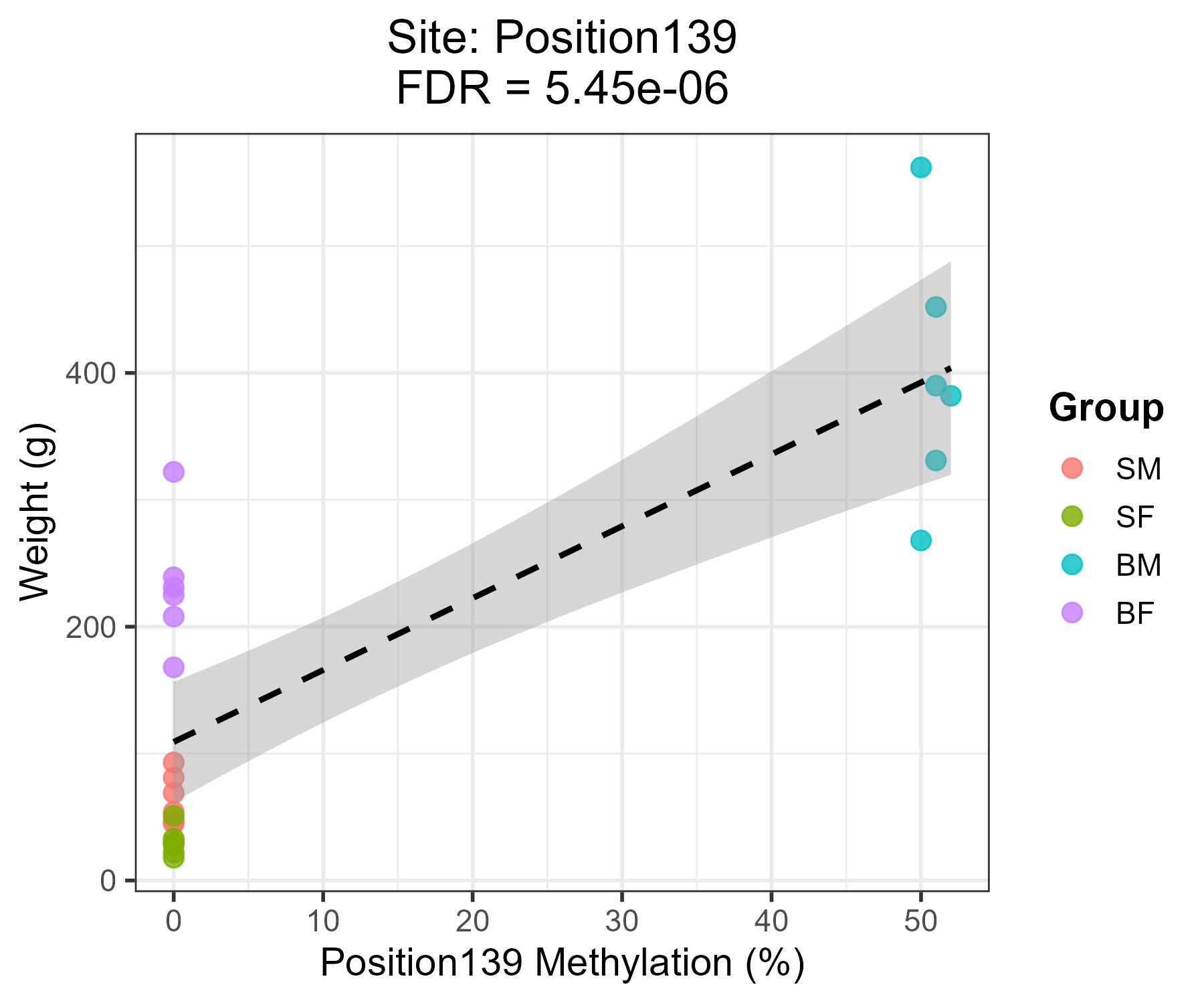

Supplement: Supplementary file 4 [file DataSheet2.zip › Regression_Minus_Strand/Position139_regression.tiff]

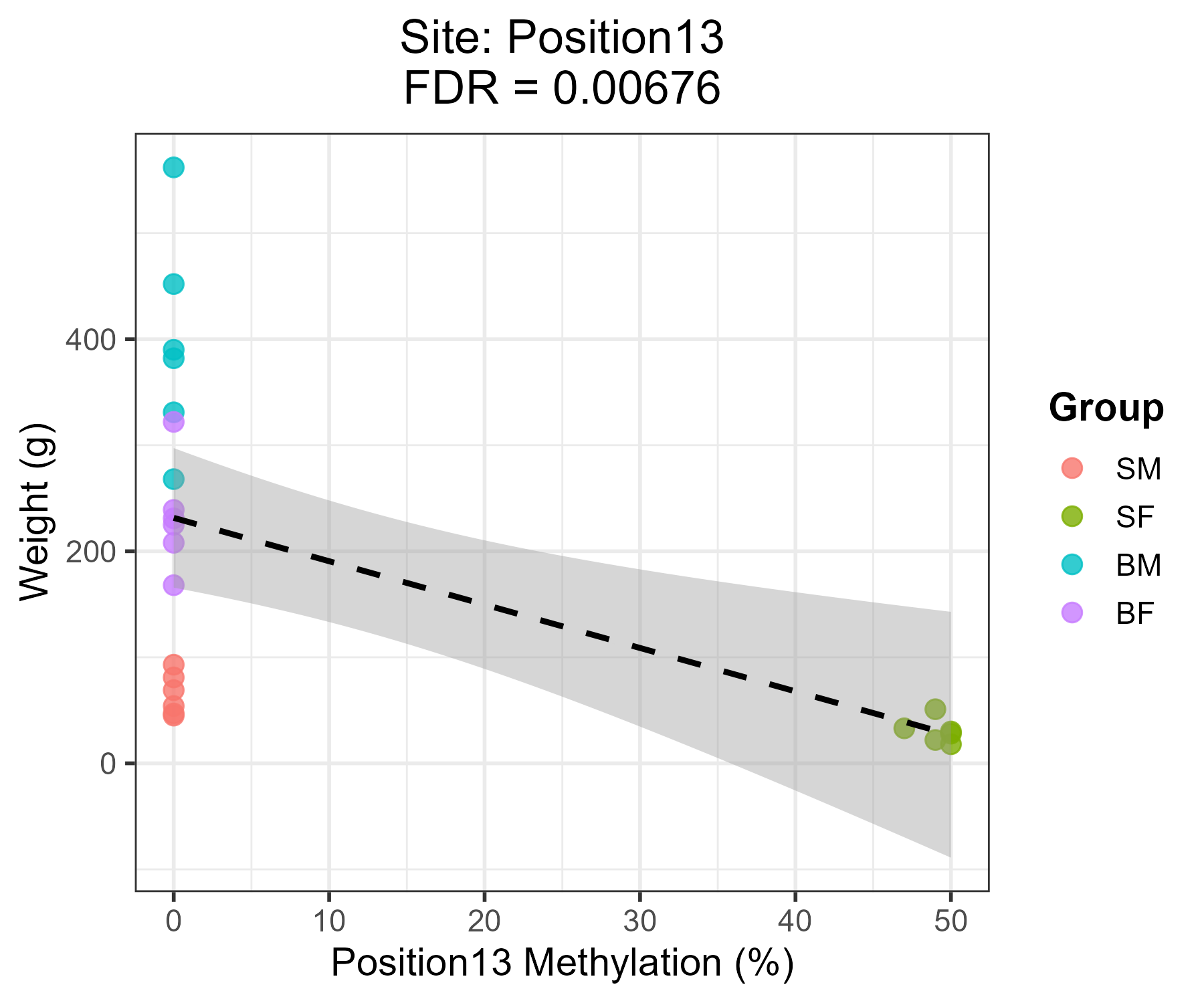

Supplement: Supplementary file 4 [file DataSheet2.zip › Regression_Minus_Strand/Position13_regression.tiff]

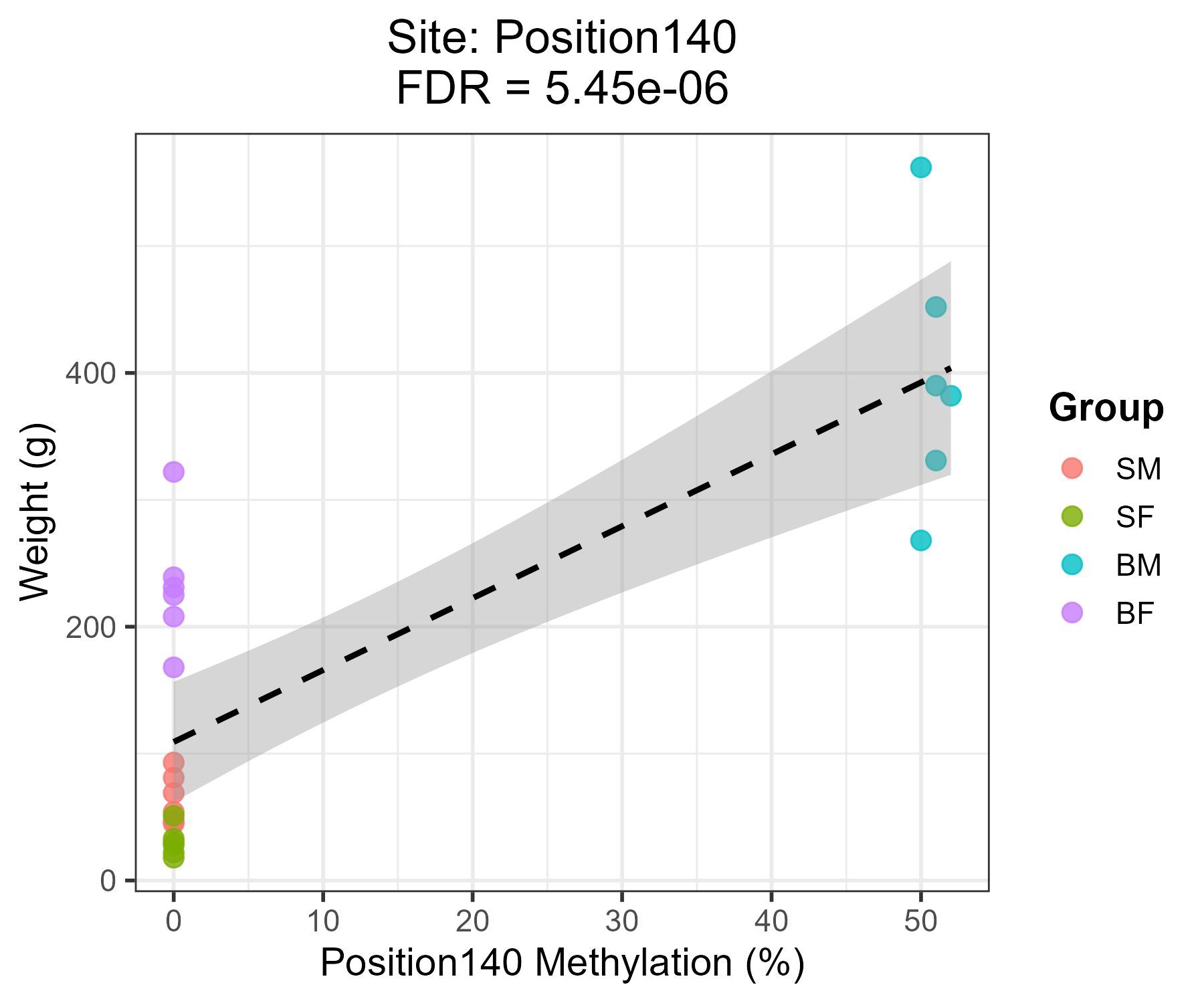

Supplement: Supplementary file 4 [file DataSheet2.zip › Regression_Minus_Strand/Position140_regression.tiff]

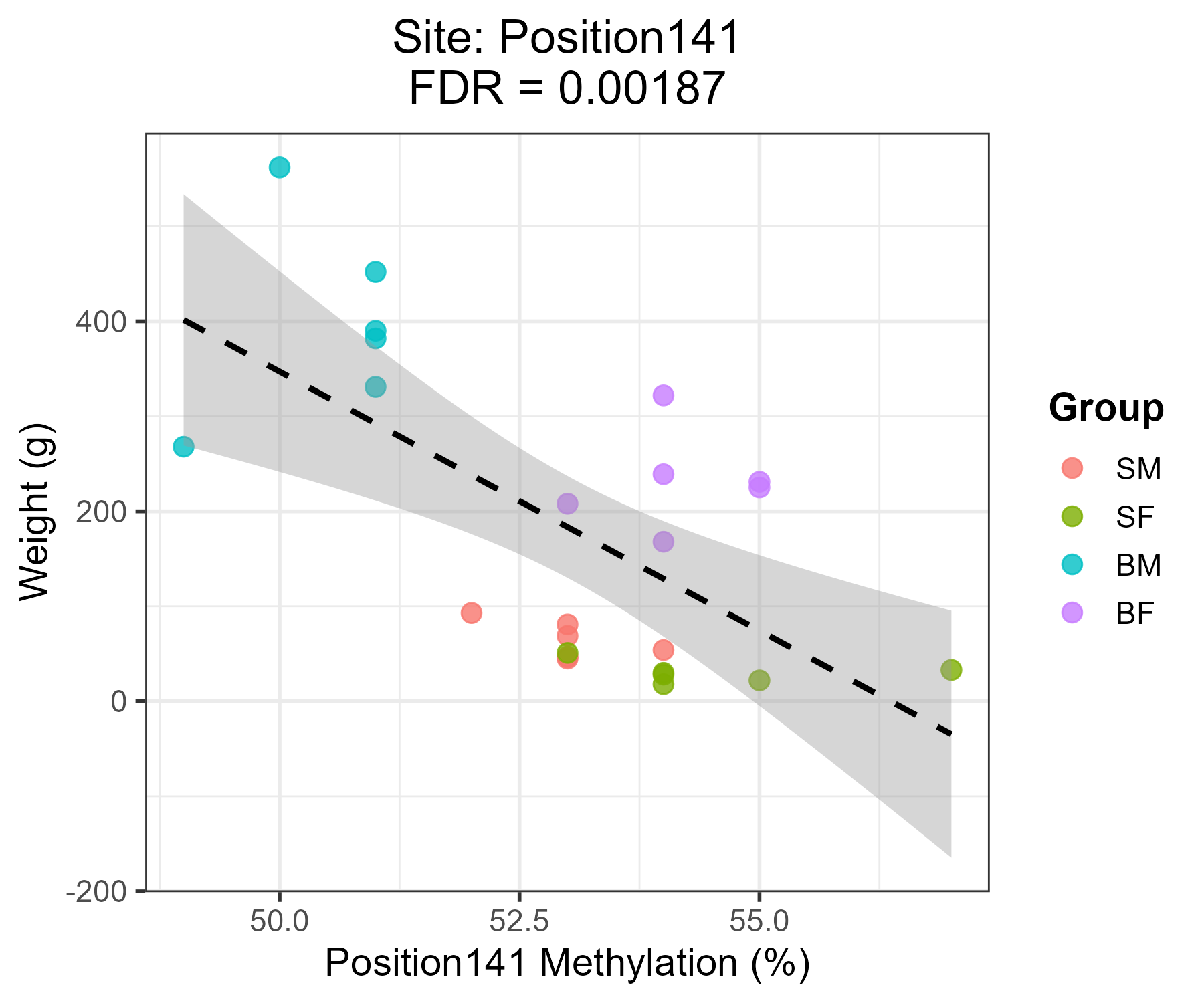

Supplement: Supplementary file 4 [file DataSheet2.zip › Regression_Minus_Strand/Position141_regression.tiff]

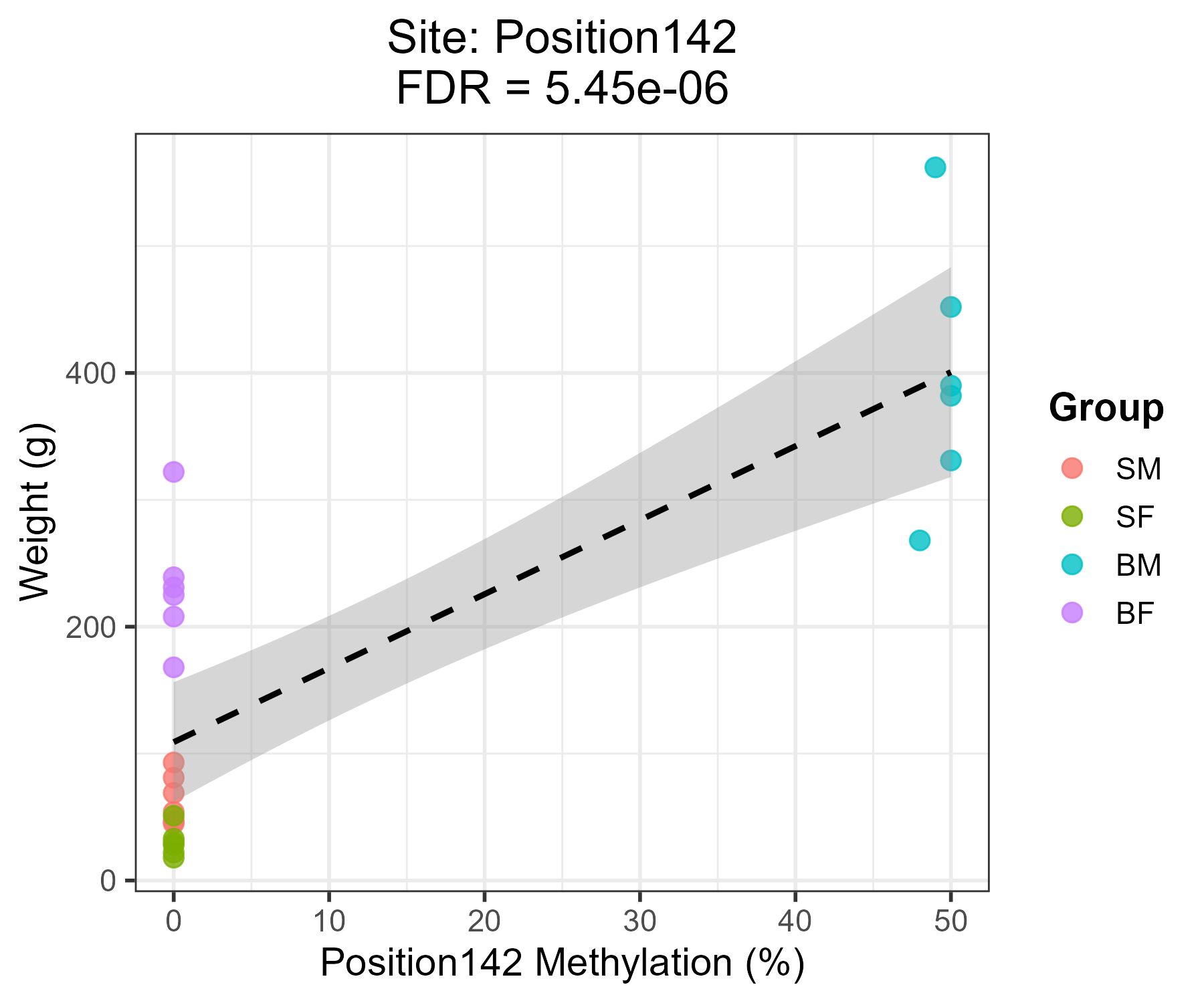

Supplement: Supplementary file 4 [file DataSheet2.zip › Regression_Minus_Strand/Position142_regression.tiff]

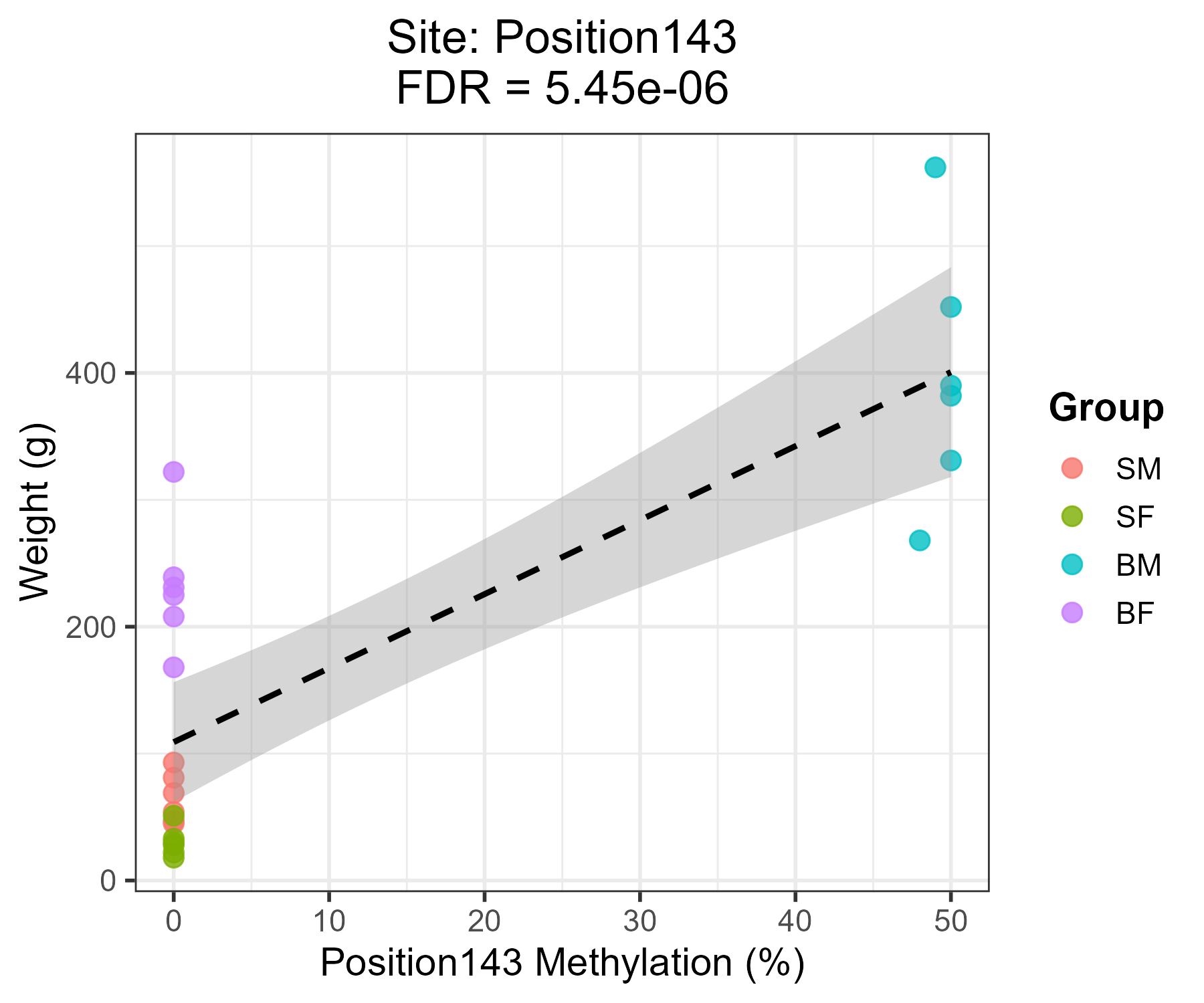

Supplement: Supplementary file 4 [file DataSheet2.zip › Regression_Minus_Strand/Position143_regression.tiff]

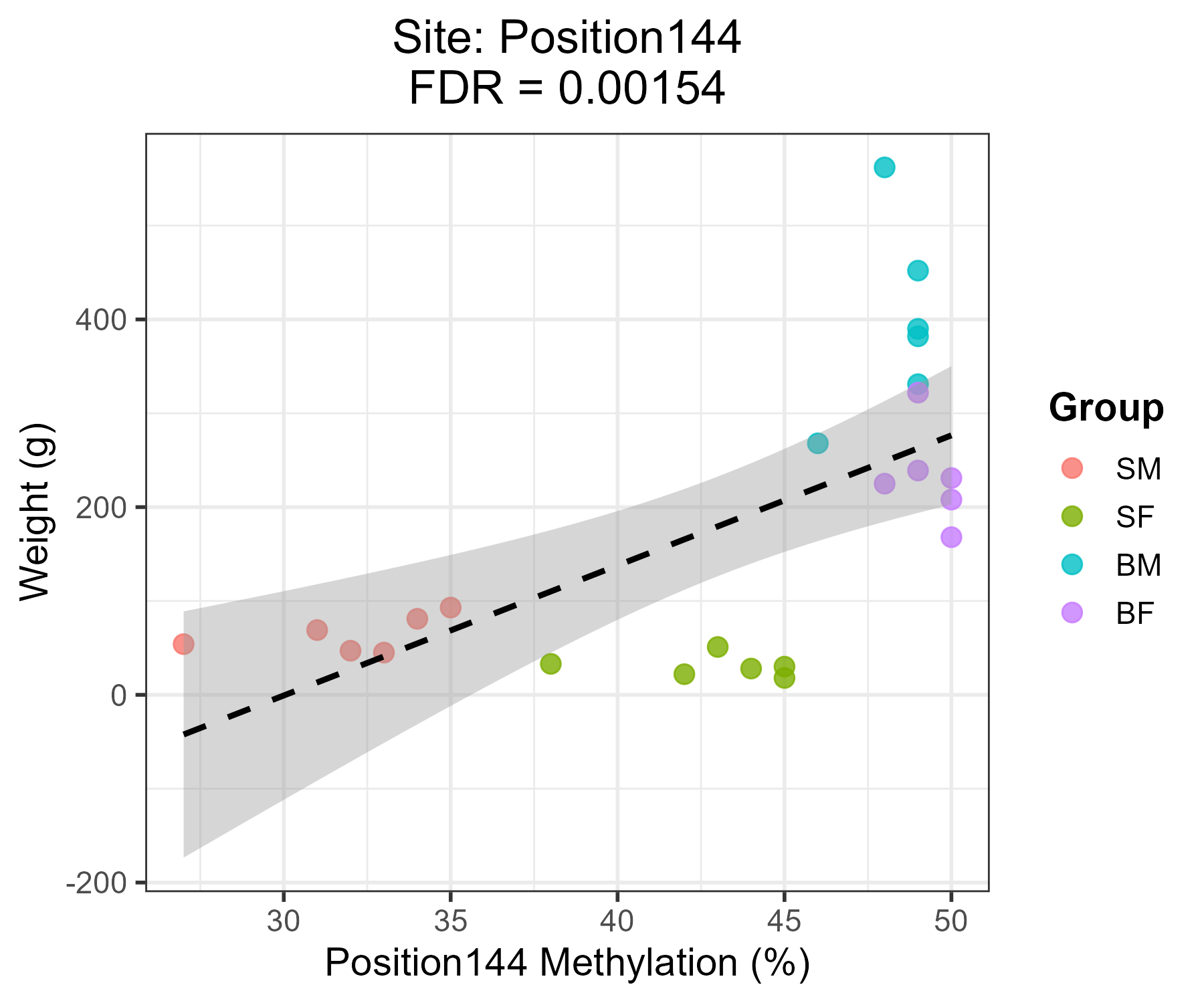

Supplement: Supplementary file 4 [file DataSheet2.zip › Regression_Minus_Strand/Position144_regression.tiff]

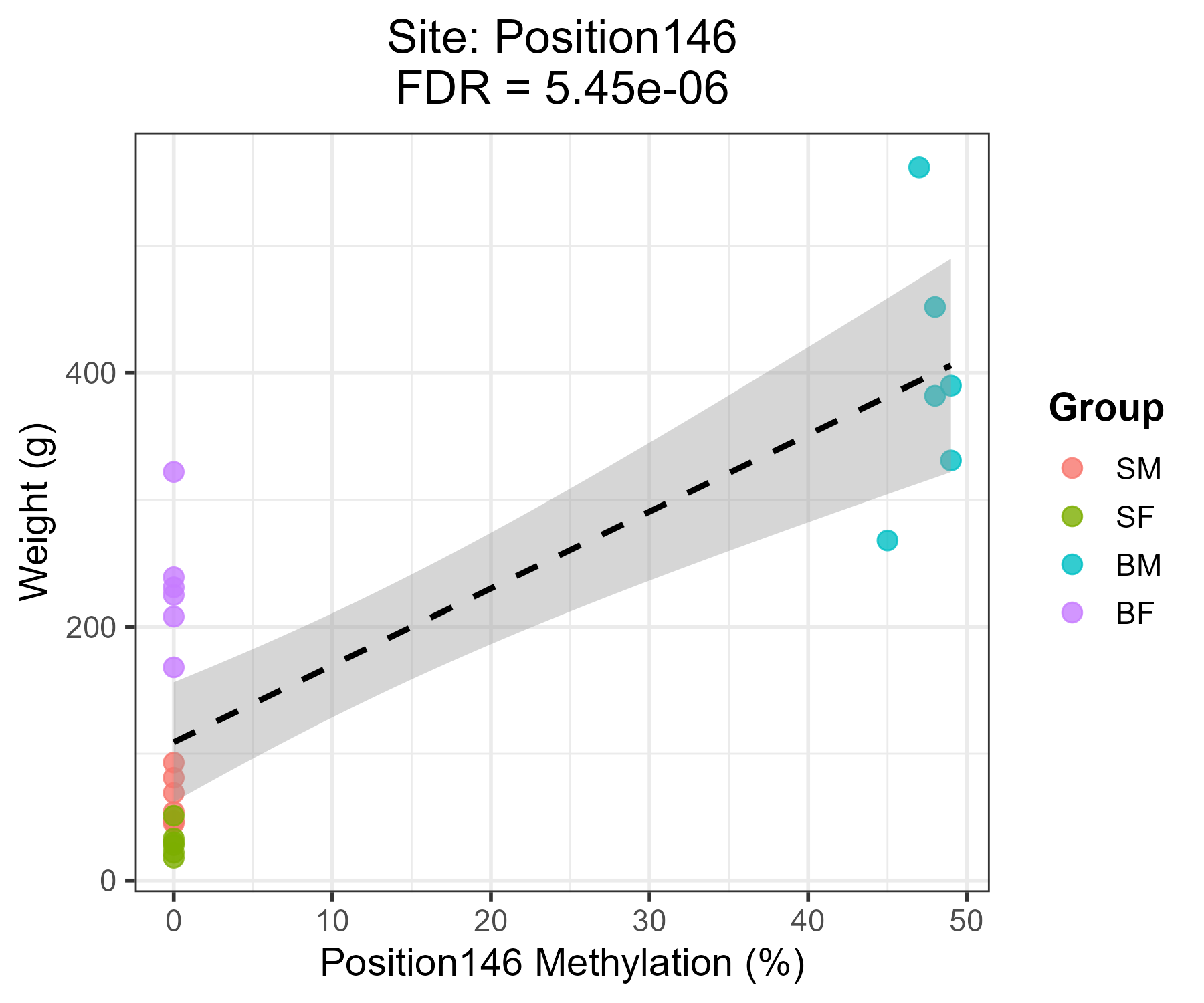

Supplement: Supplementary file 4 [file DataSheet2.zip › Regression_Minus_Strand/Position146_regression.tiff]

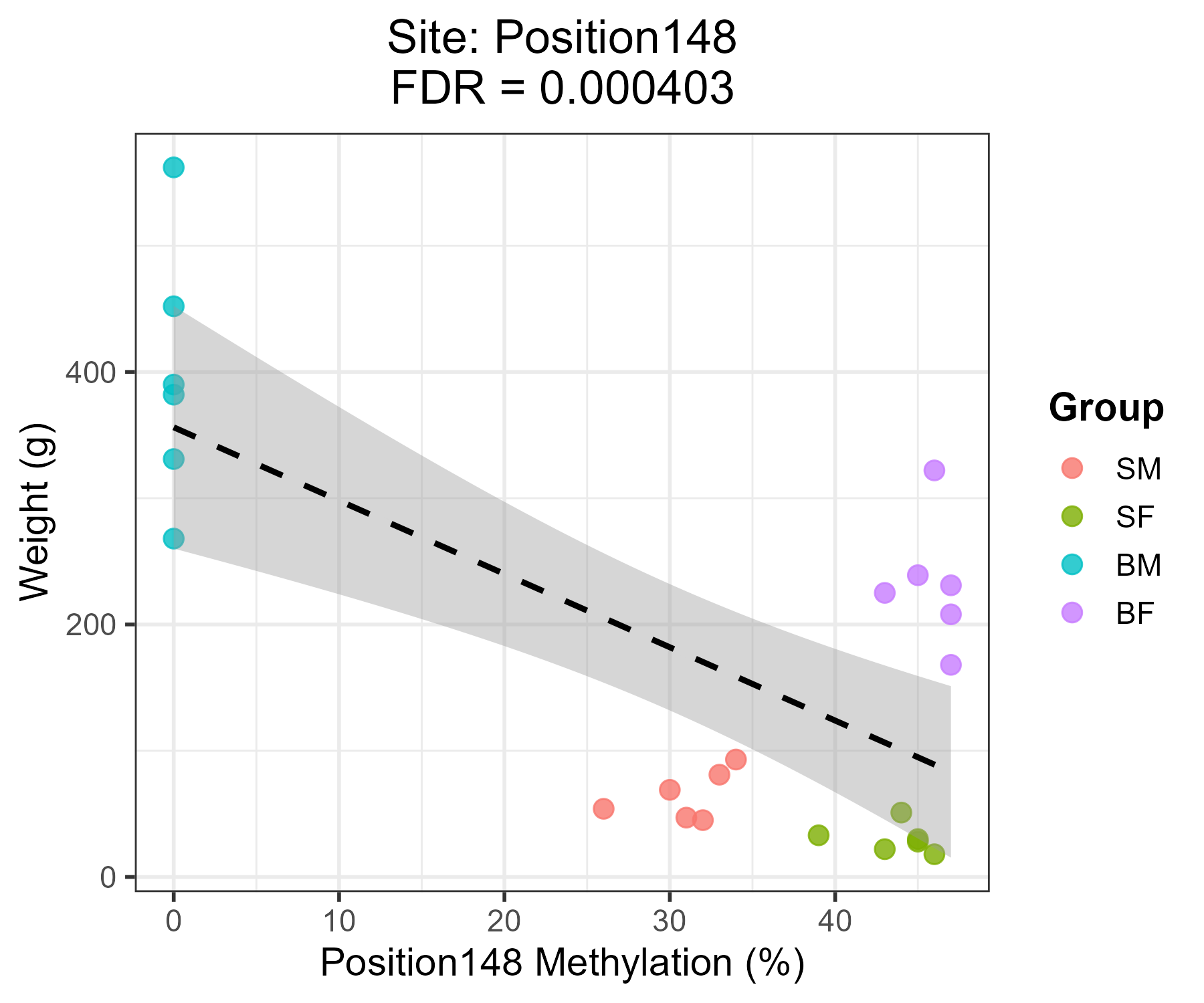

Supplement: Supplementary file 4 [file DataSheet2.zip › Regression_Minus_Strand/Position148_regression.tiff]

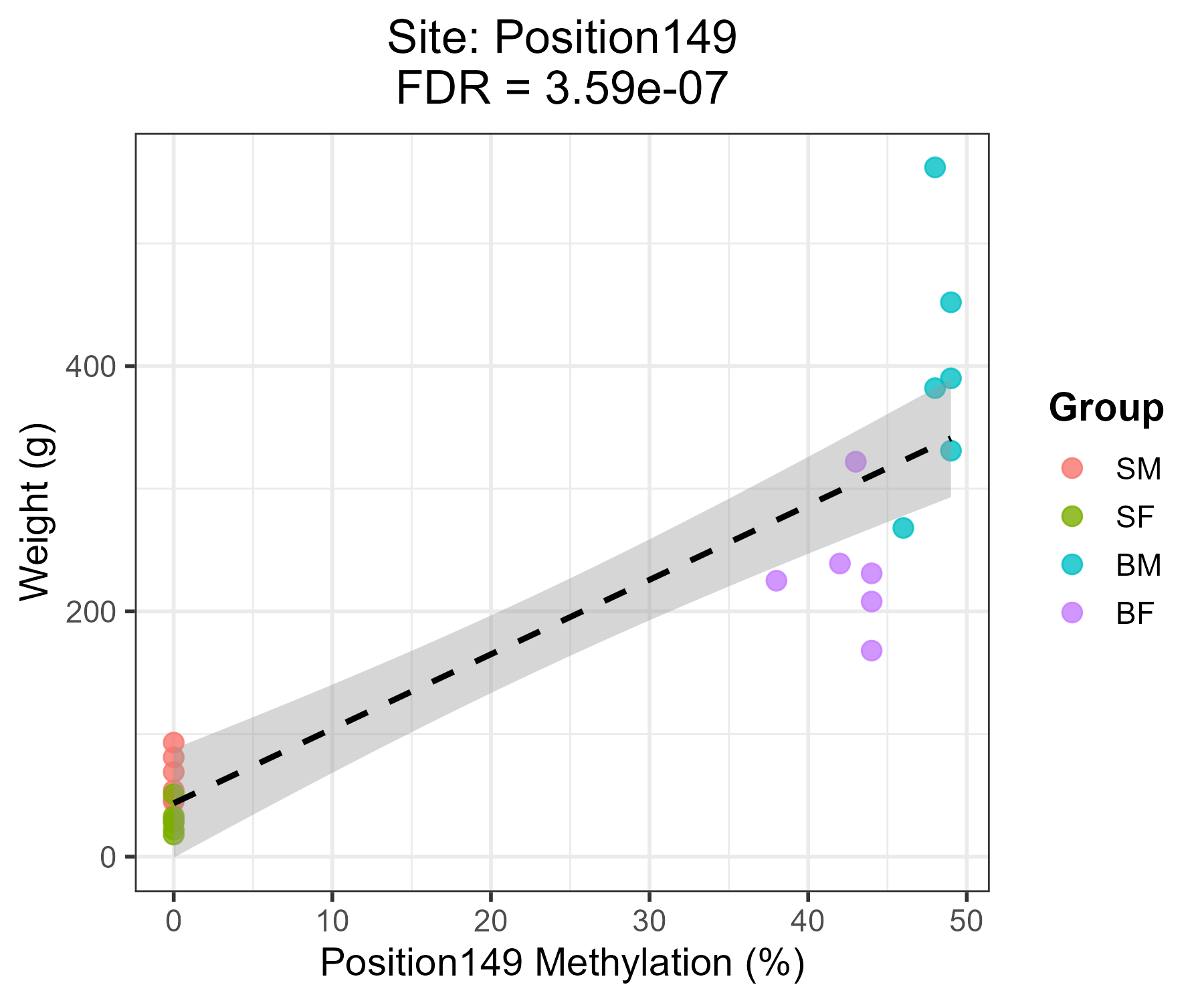

Supplement: Supplementary file 4 [file DataSheet2.zip › Regression_Minus_Strand/Position149_regression.tiff]

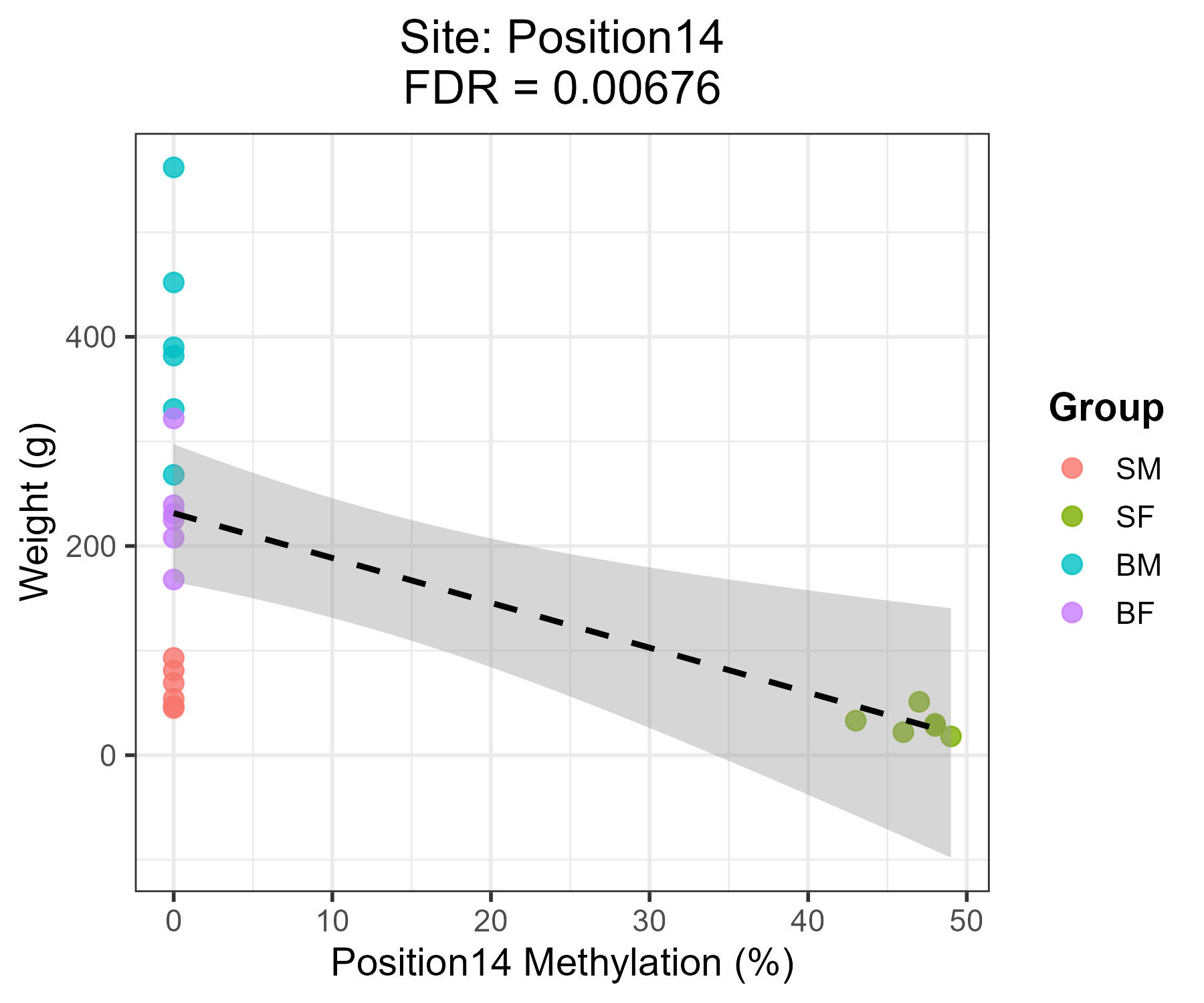

Supplement: Supplementary file 4 [file DataSheet2.zip › Regression_Minus_Strand/Position14_regression.tiff]

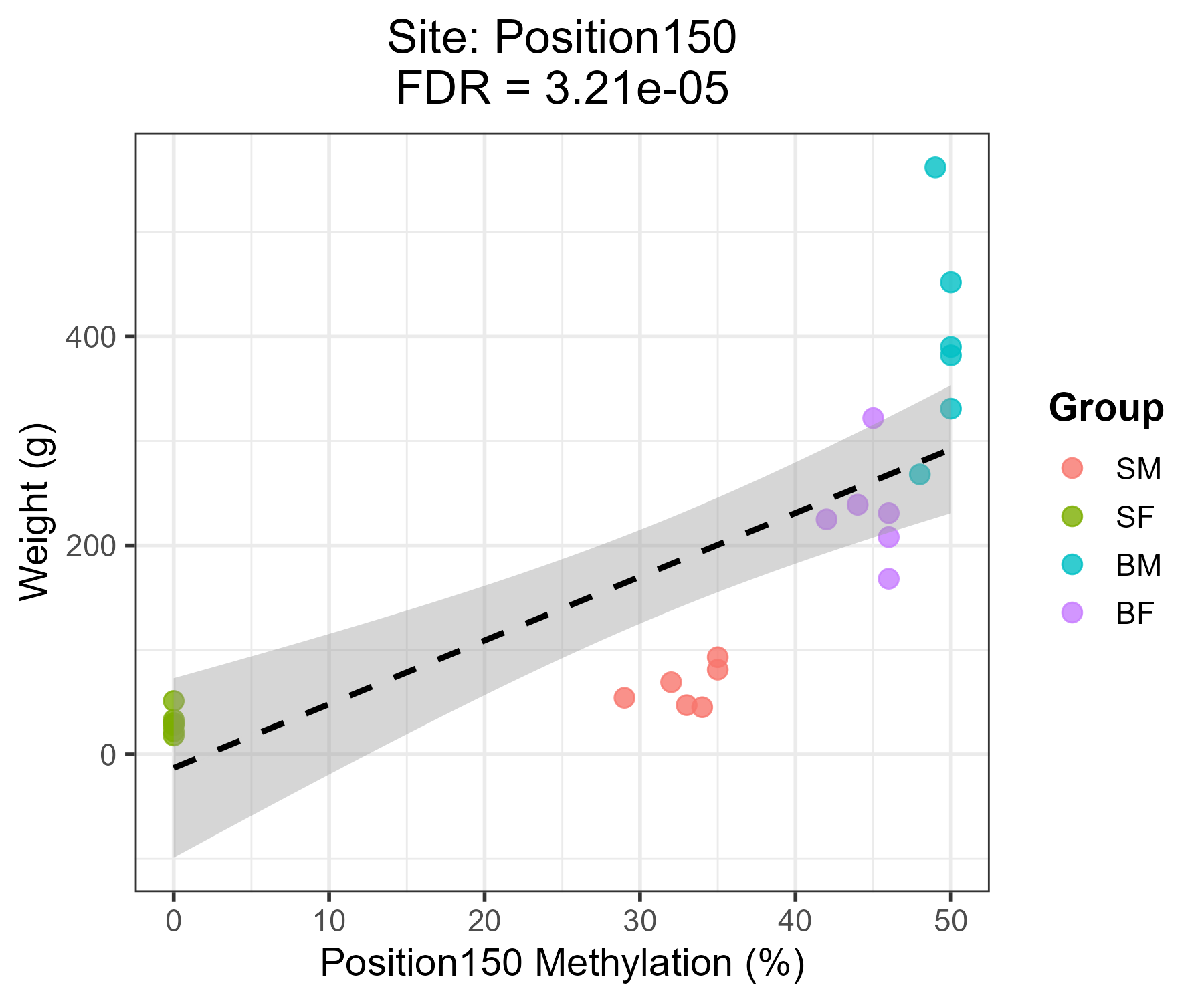

Supplement: Supplementary file 4 [file DataSheet2.zip › Regression_Minus_Strand/Position150_regression.tiff]

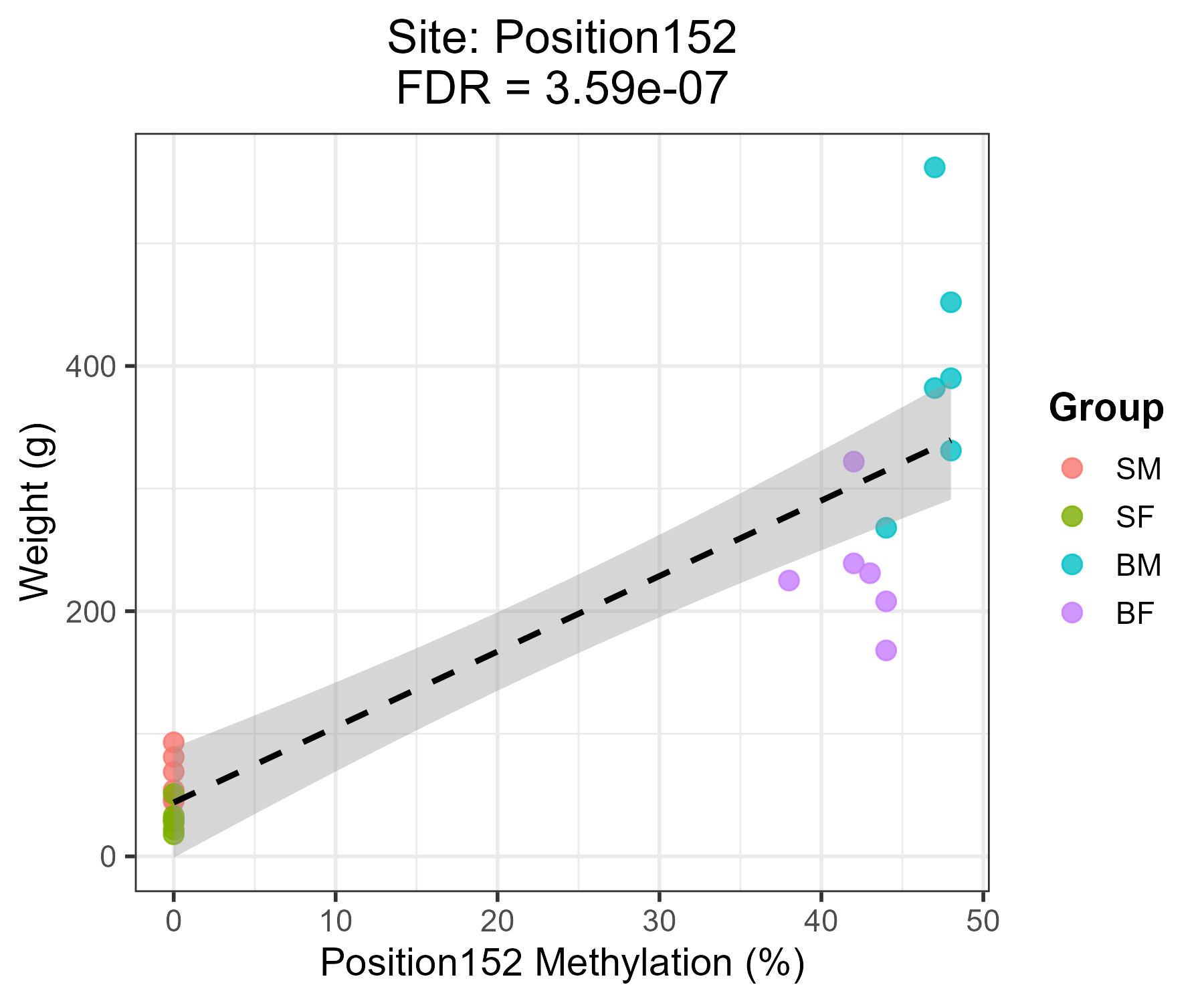

Supplement: Supplementary file 4 [file DataSheet2.zip › Regression_Minus_Strand/Position152_regression.tiff]

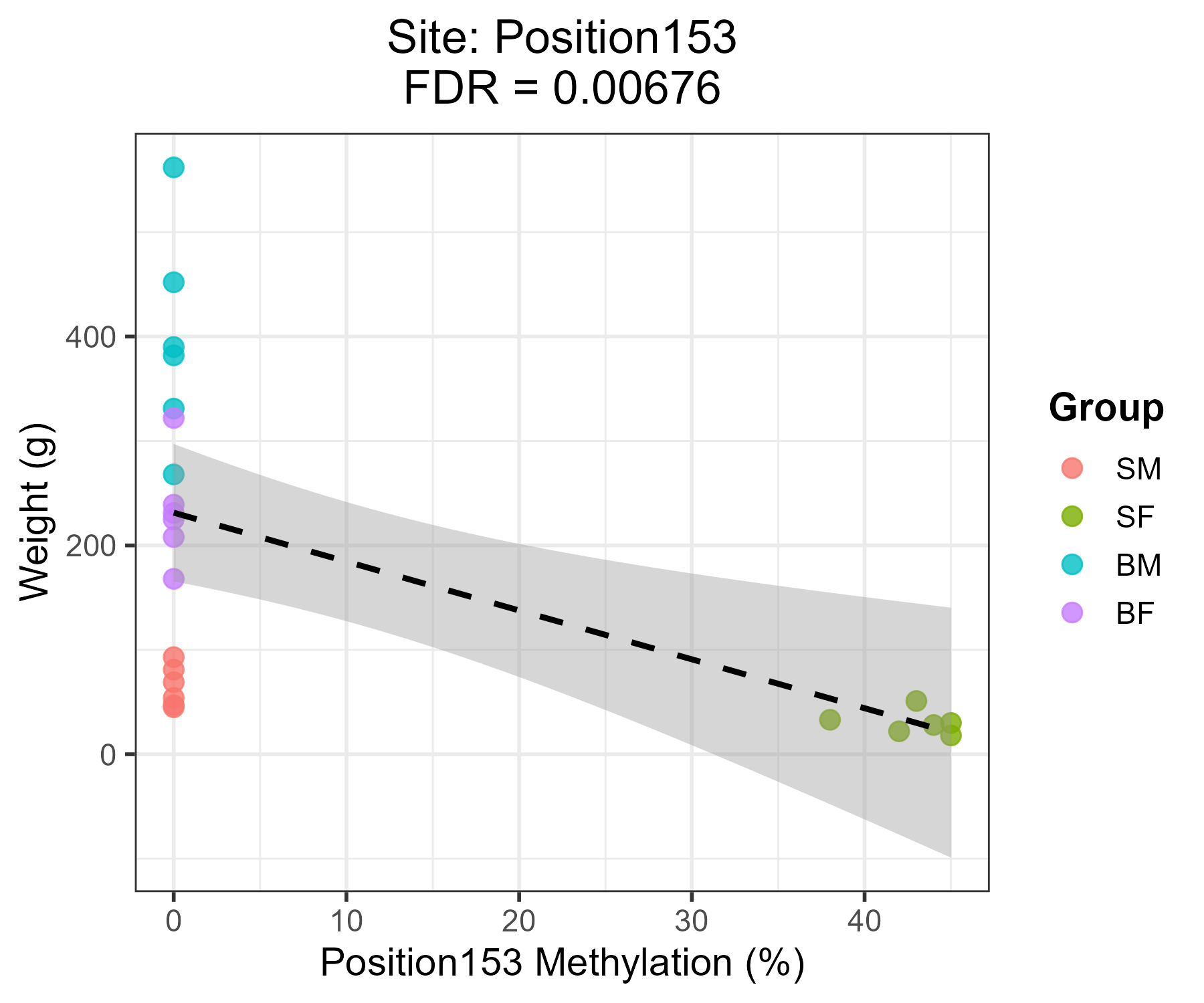

Supplement: Supplementary file 4 [file DataSheet2.zip › Regression_Minus_Strand/Position153_regression.tiff]

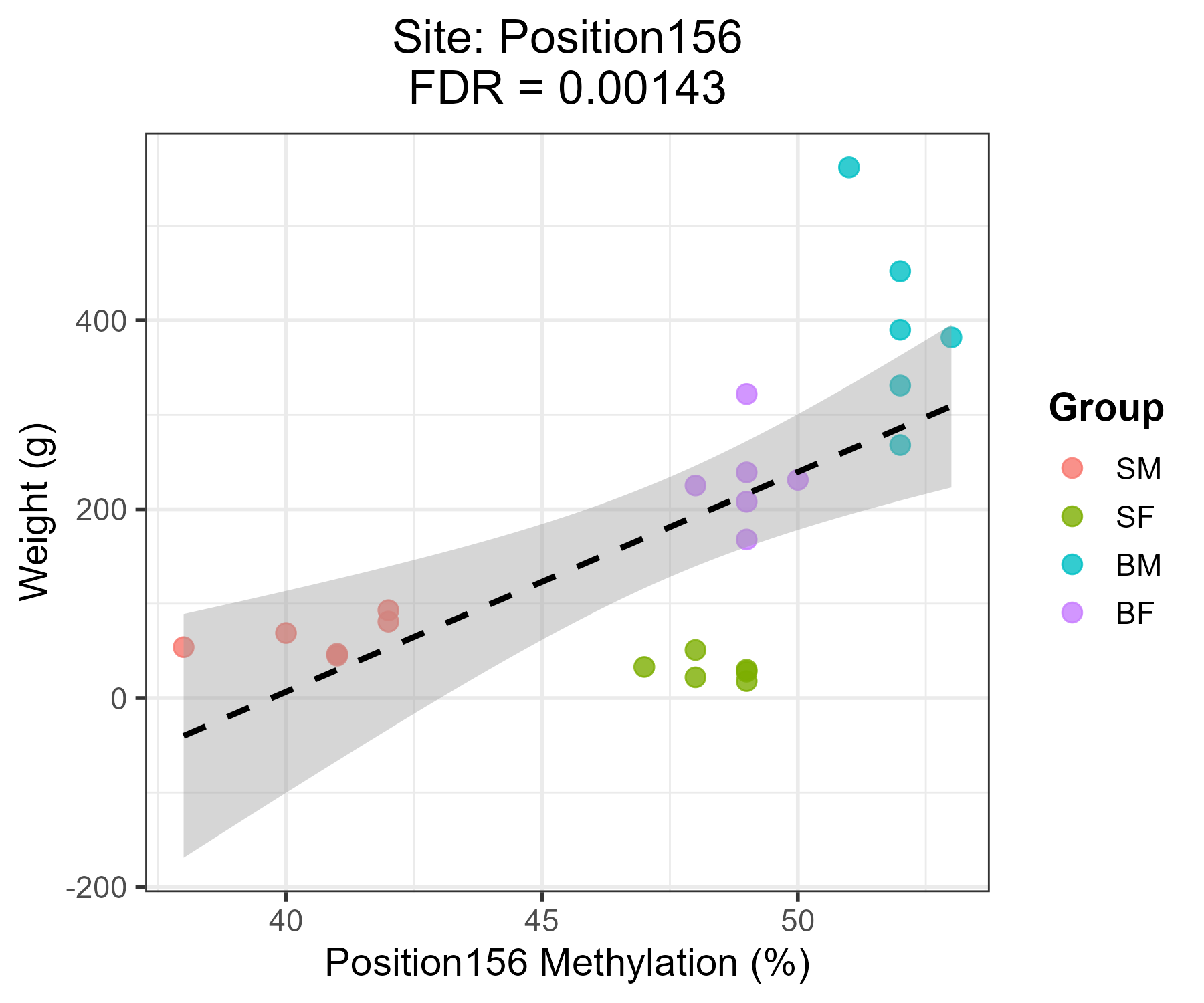

Supplement: Supplementary file 4 [file DataSheet2.zip › Regression_Minus_Strand/Position156_regression.tiff]

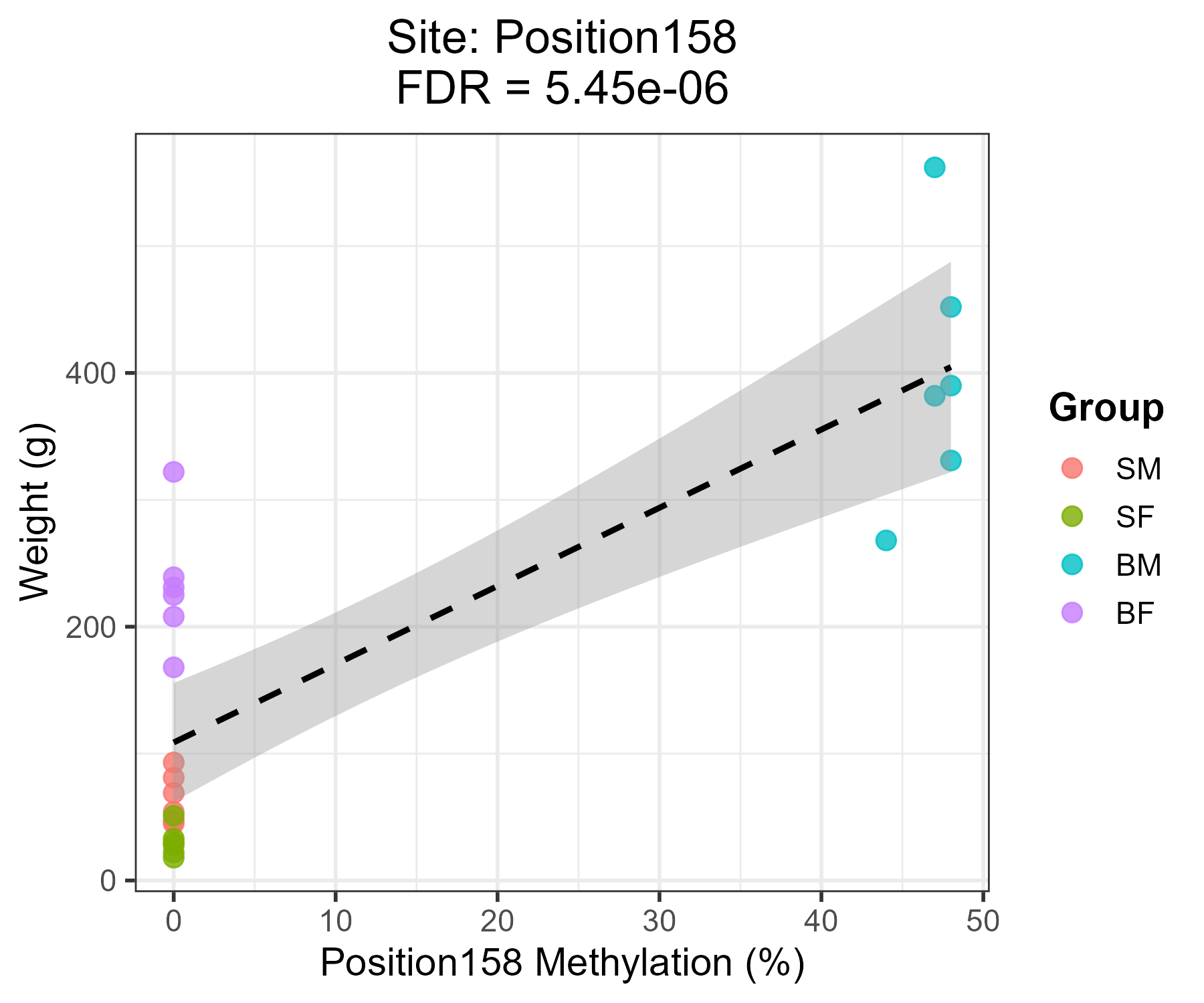

Supplement: Supplementary file 4 [file DataSheet2.zip › Regression_Minus_Strand/Position158_regression.tiff]

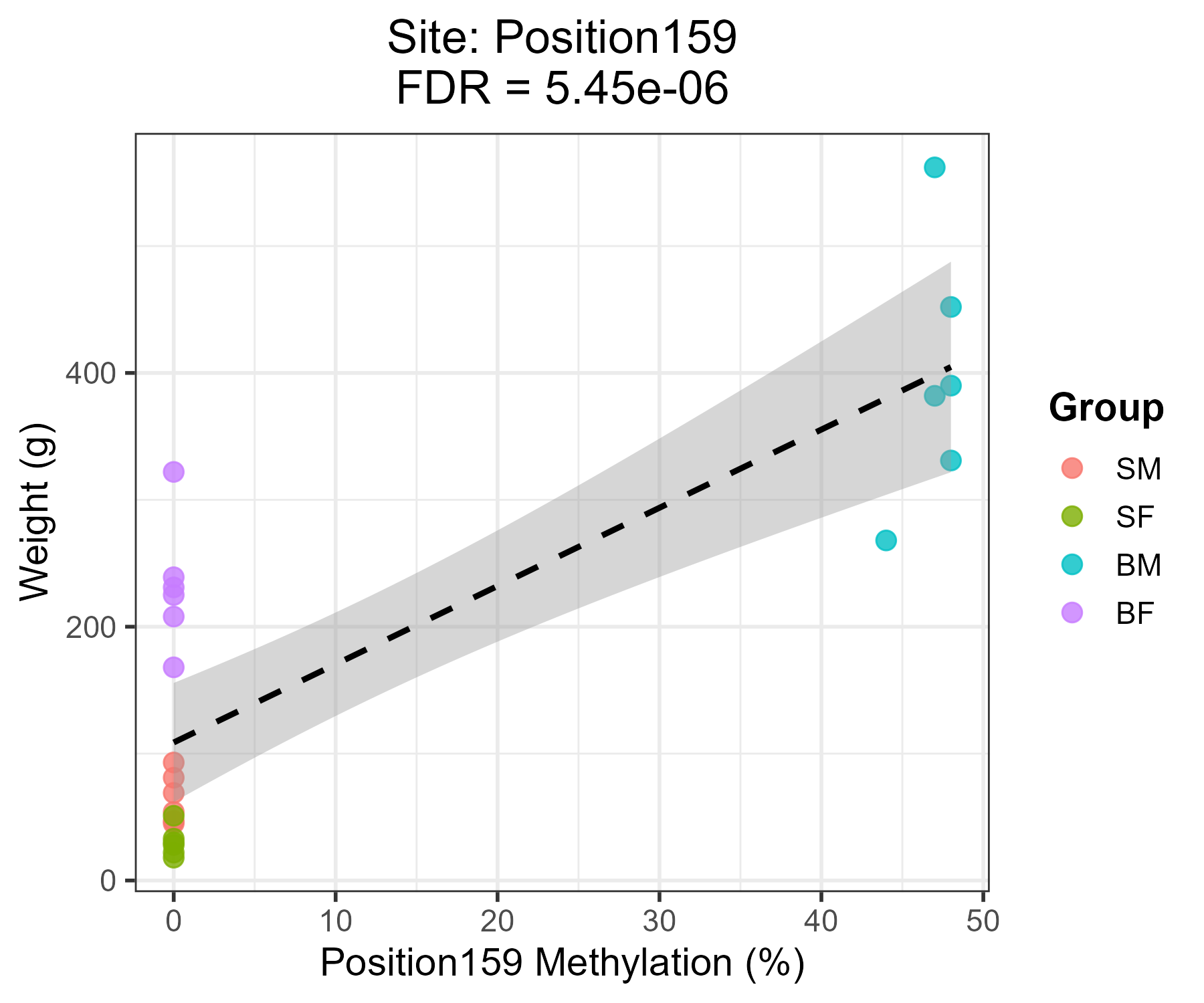

Supplement: Supplementary file 4 [file DataSheet2.zip › Regression_Minus_Strand/Position159_regression.tiff]

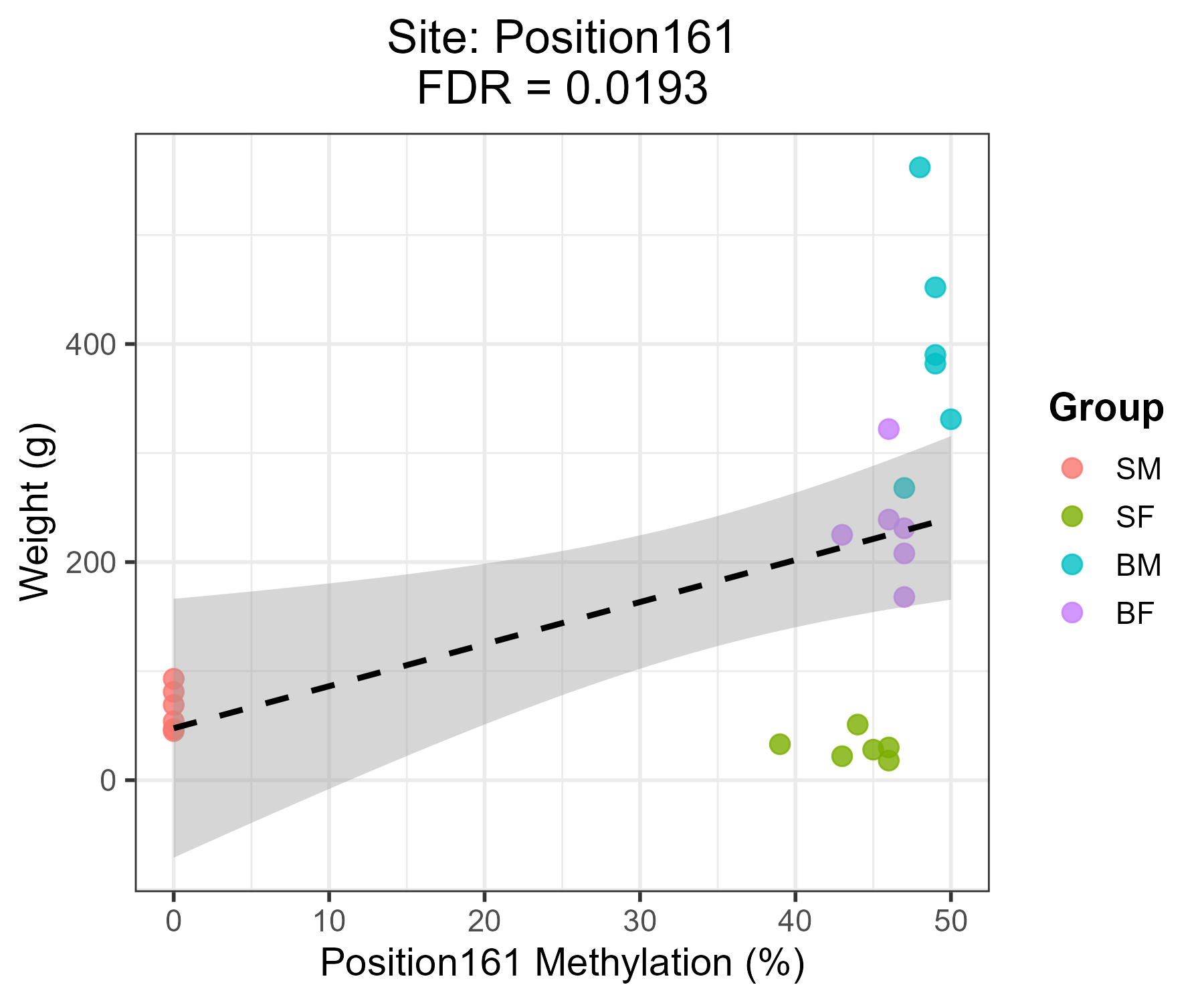

Supplement: Supplementary file 4 [file DataSheet2.zip › Regression_Minus_Strand/Position161_regression.tiff]

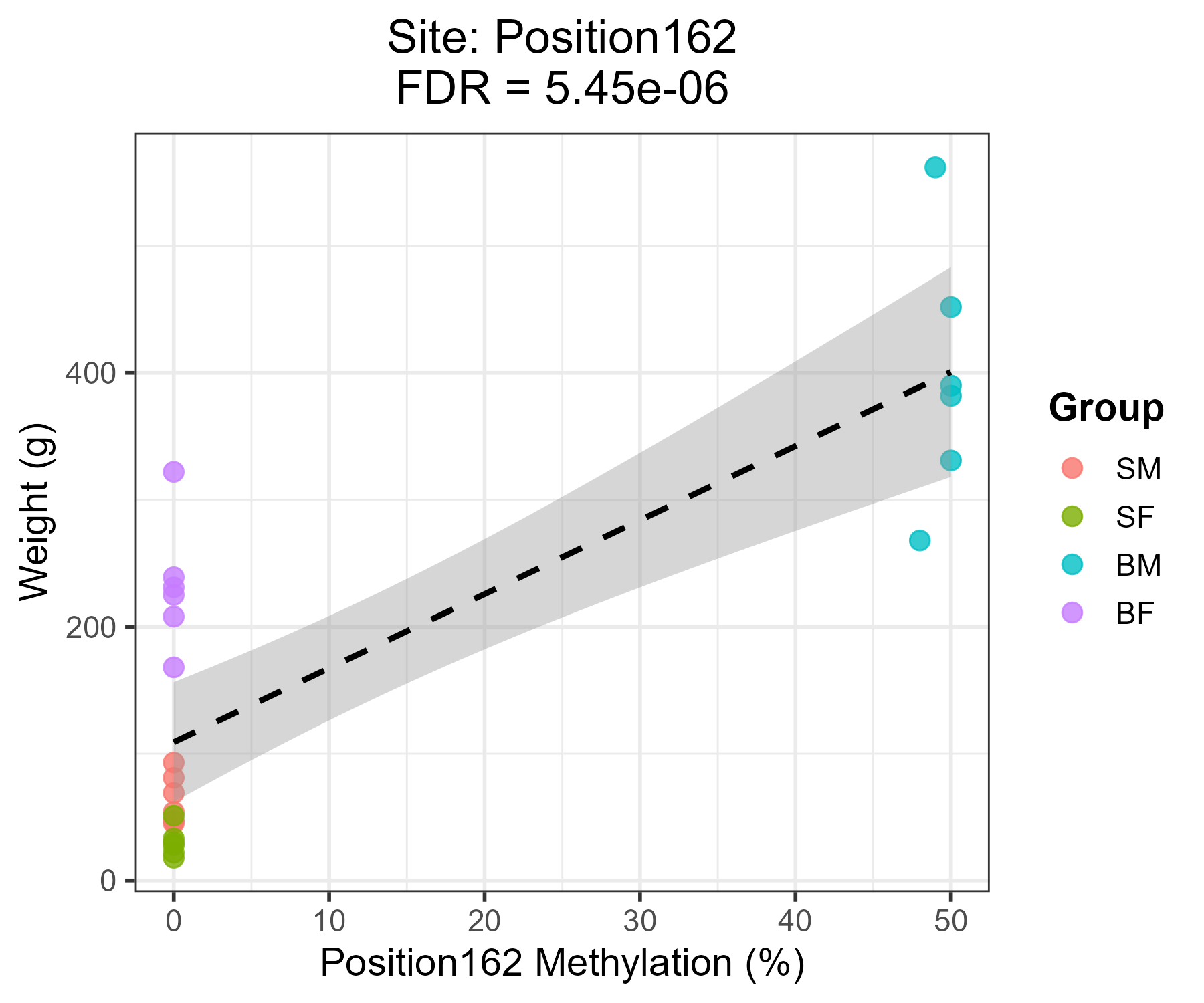

Supplement: Supplementary file 4 [file DataSheet2.zip › Regression_Minus_Strand/Position162_regression.tiff]

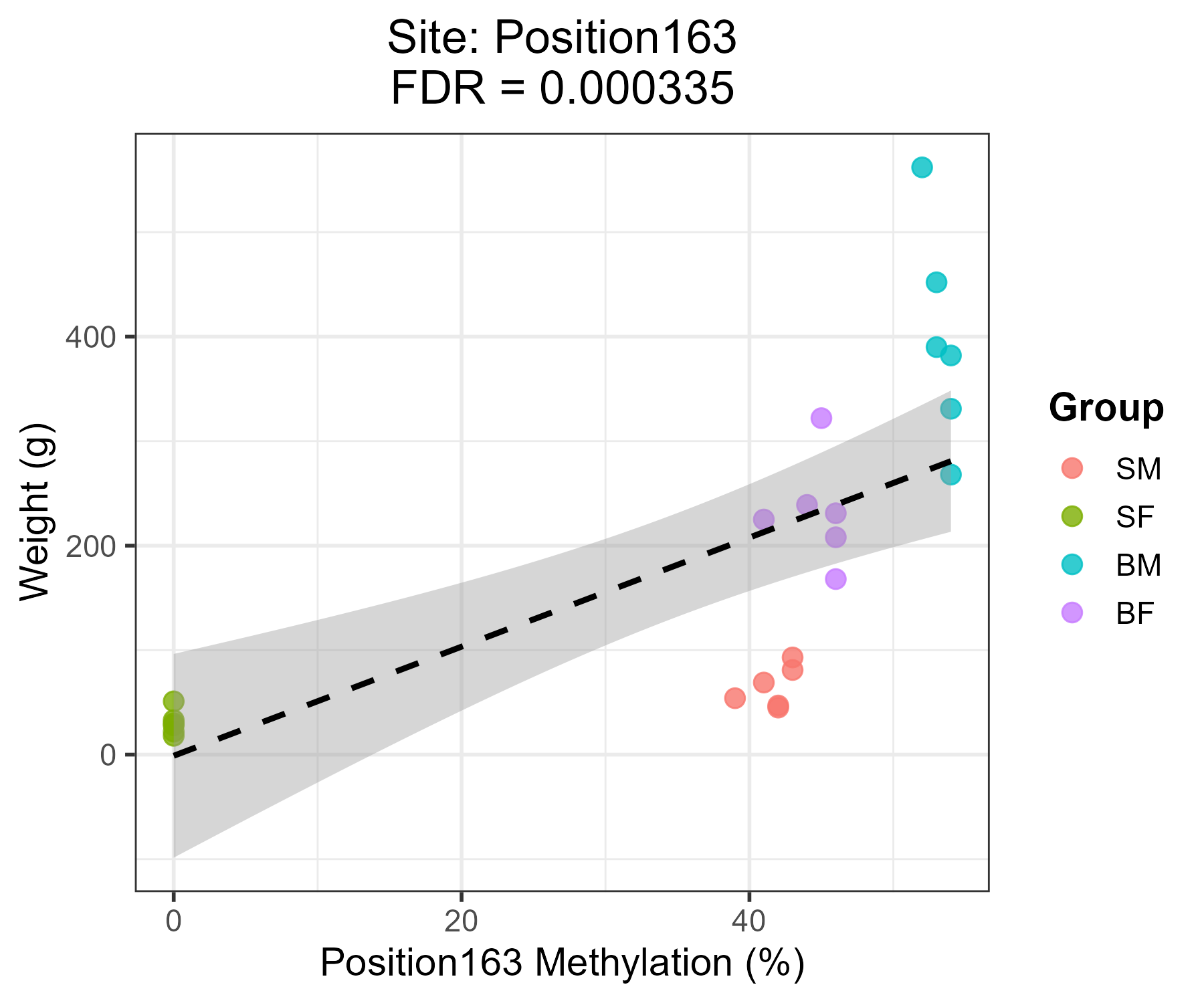

Supplement: Supplementary file 4 [file DataSheet2.zip › Regression_Minus_Strand/Position163_regression.tiff]

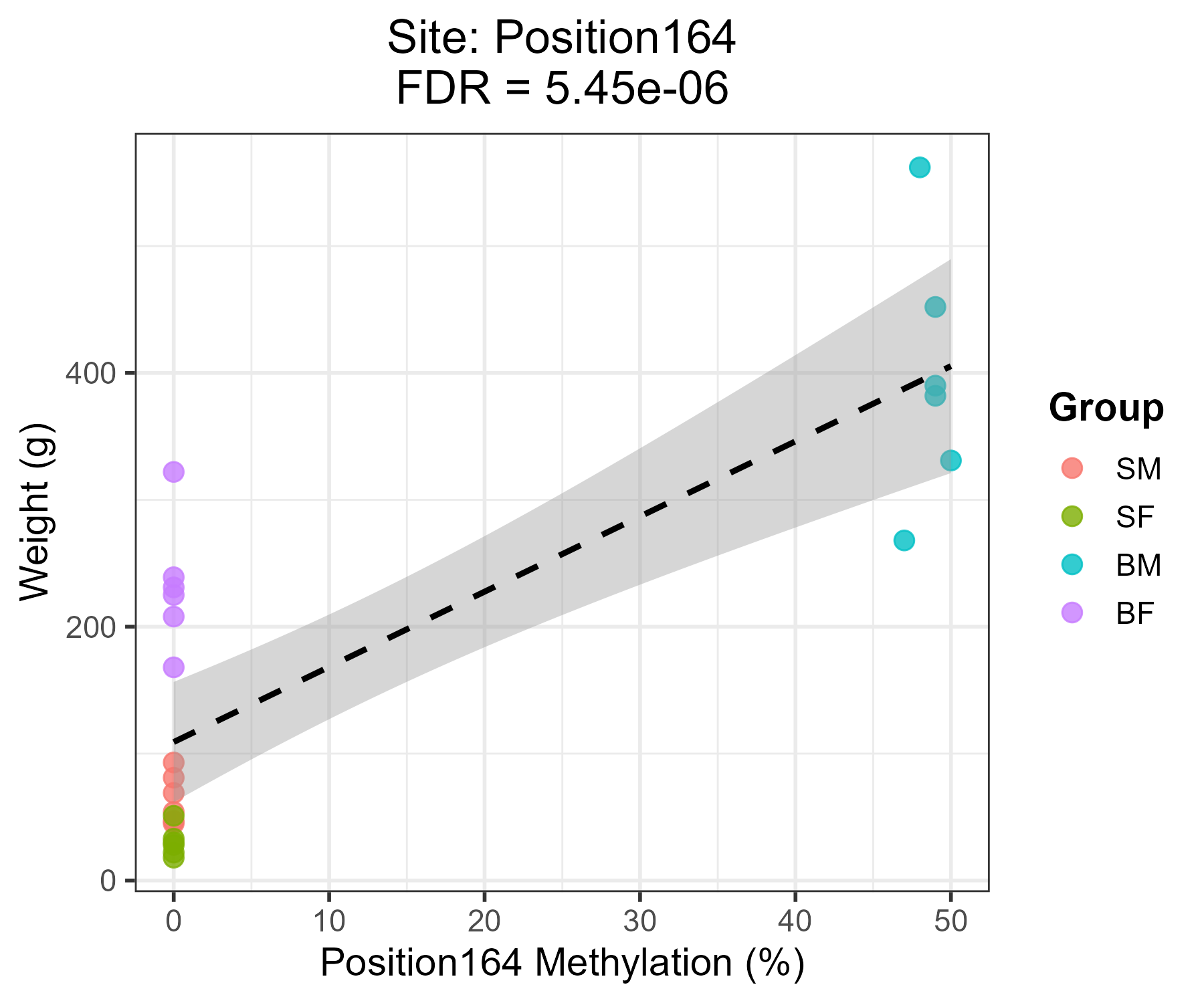

Supplement: Supplementary file 4 [file DataSheet2.zip › Regression_Minus_Strand/Position164_regression.tiff]

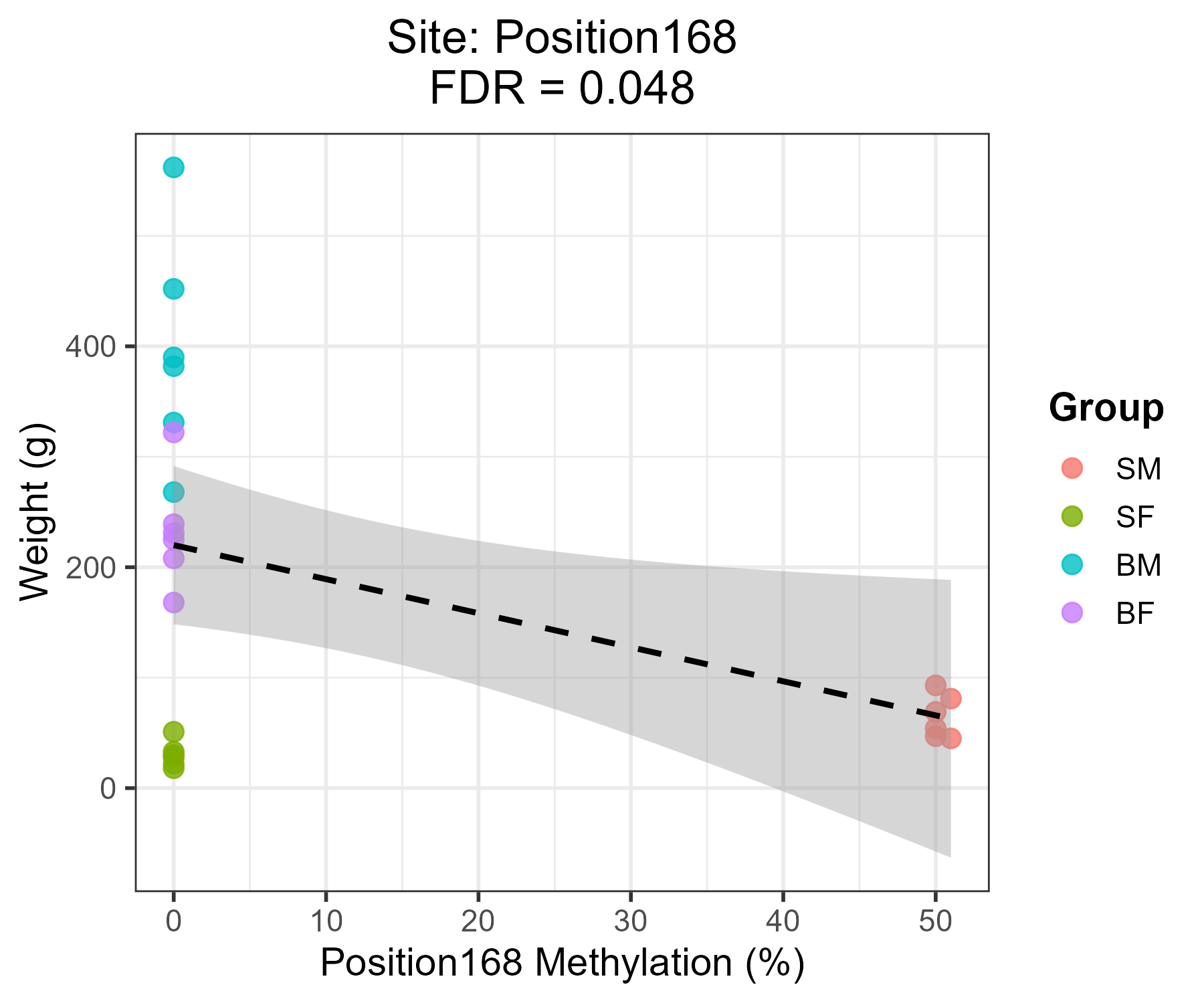

Supplement: Supplementary file 4 [file DataSheet2.zip › Regression_Minus_Strand/Position168_regression.tiff]

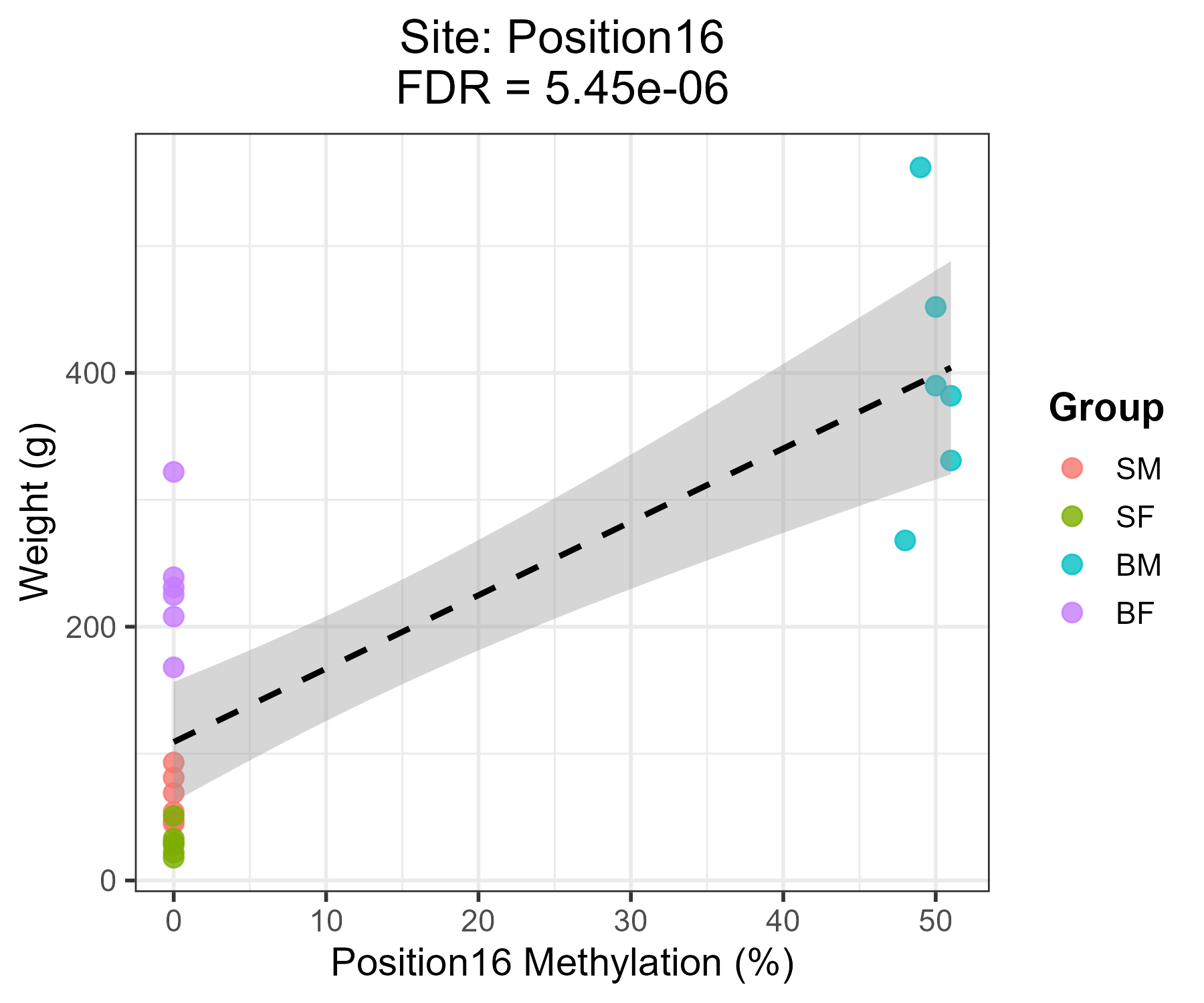

Supplement: Supplementary file 4 [file DataSheet2.zip › Regression_Minus_Strand/Position16_regression.tiff]

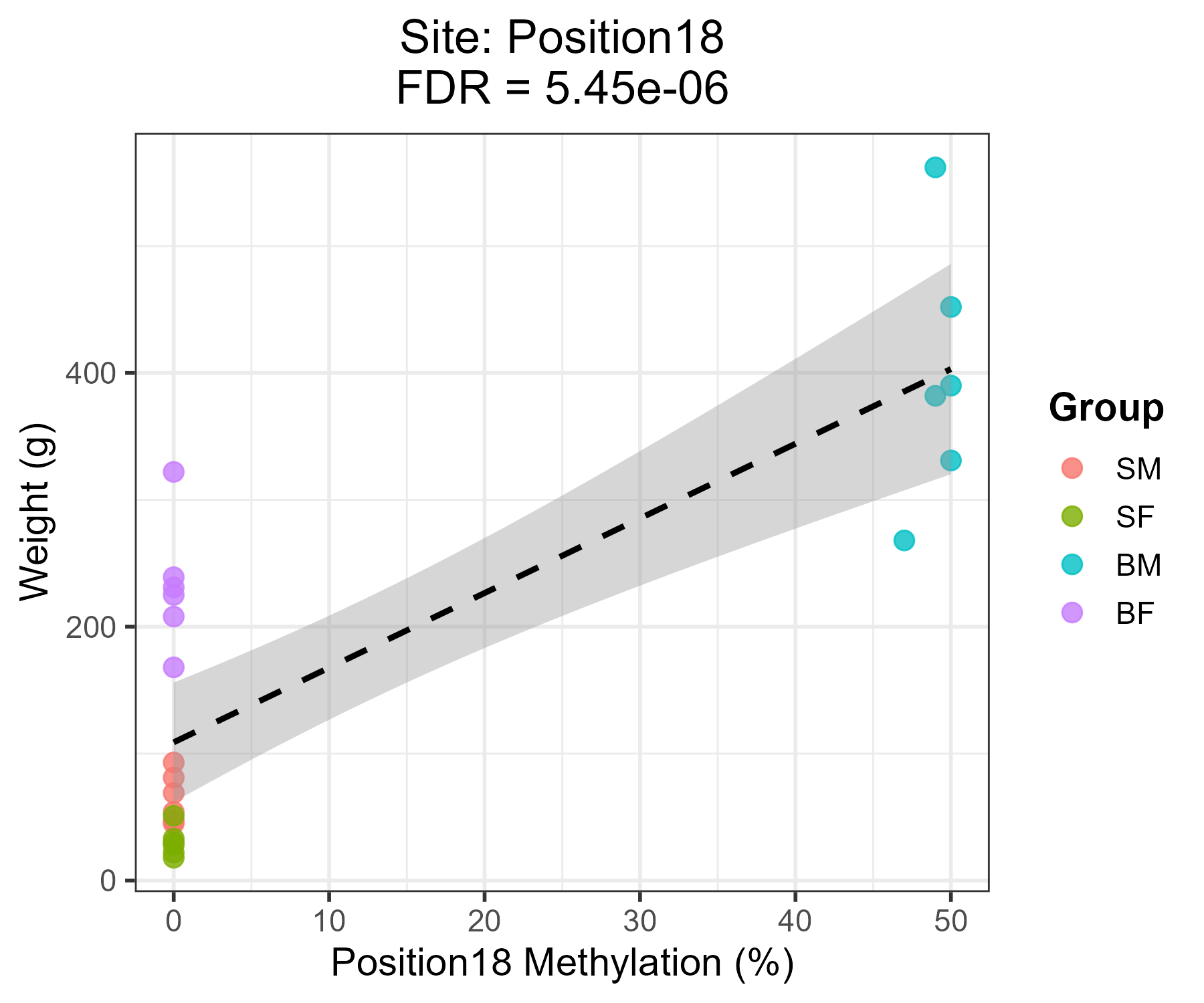

Supplement: Supplementary file 4 [file DataSheet2.zip › Regression_Minus_Strand/Position18_regression.tiff]

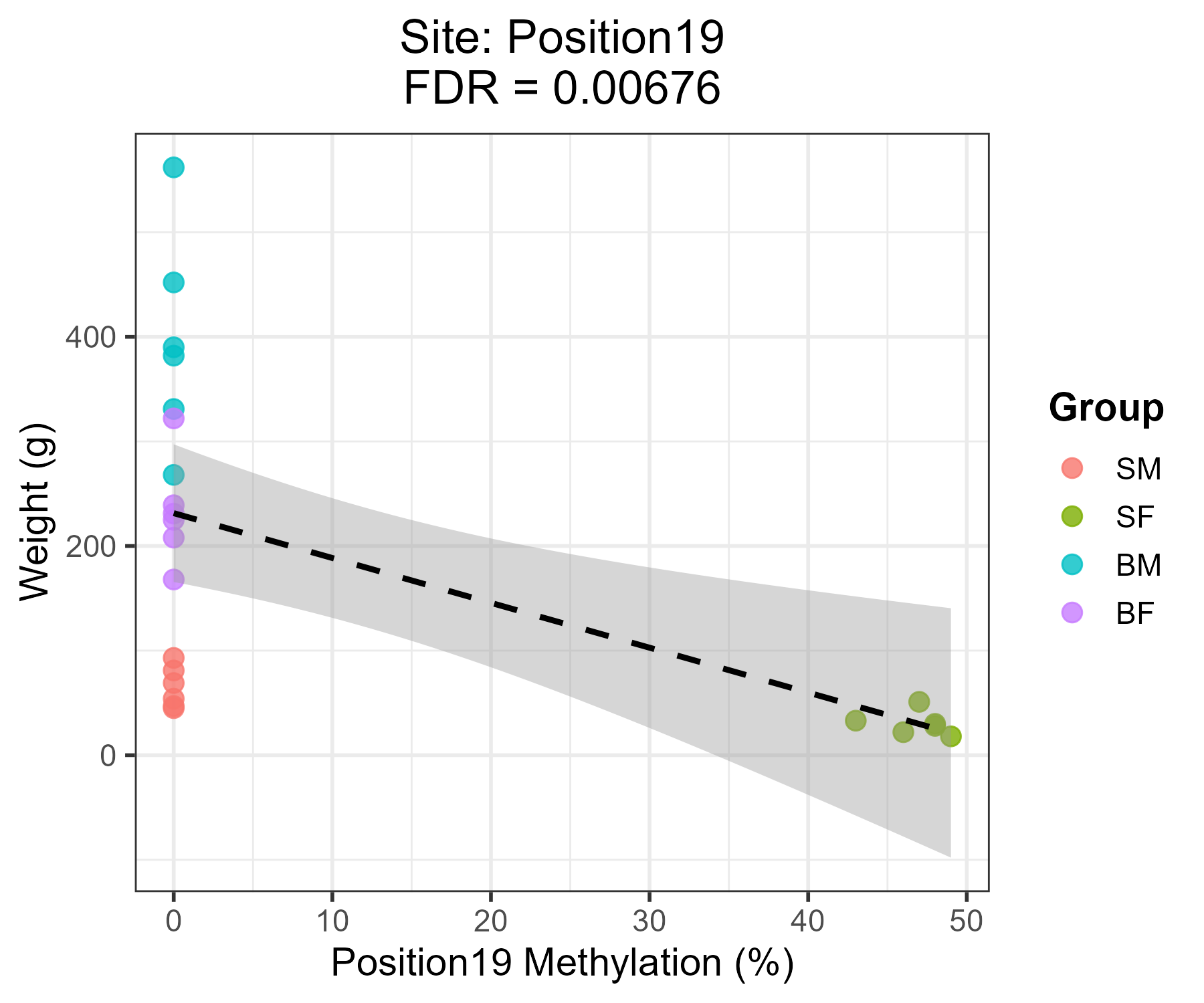

Supplement: Supplementary file 4 [file DataSheet2.zip › Regression_Minus_Strand/Position19_regression.tiff]

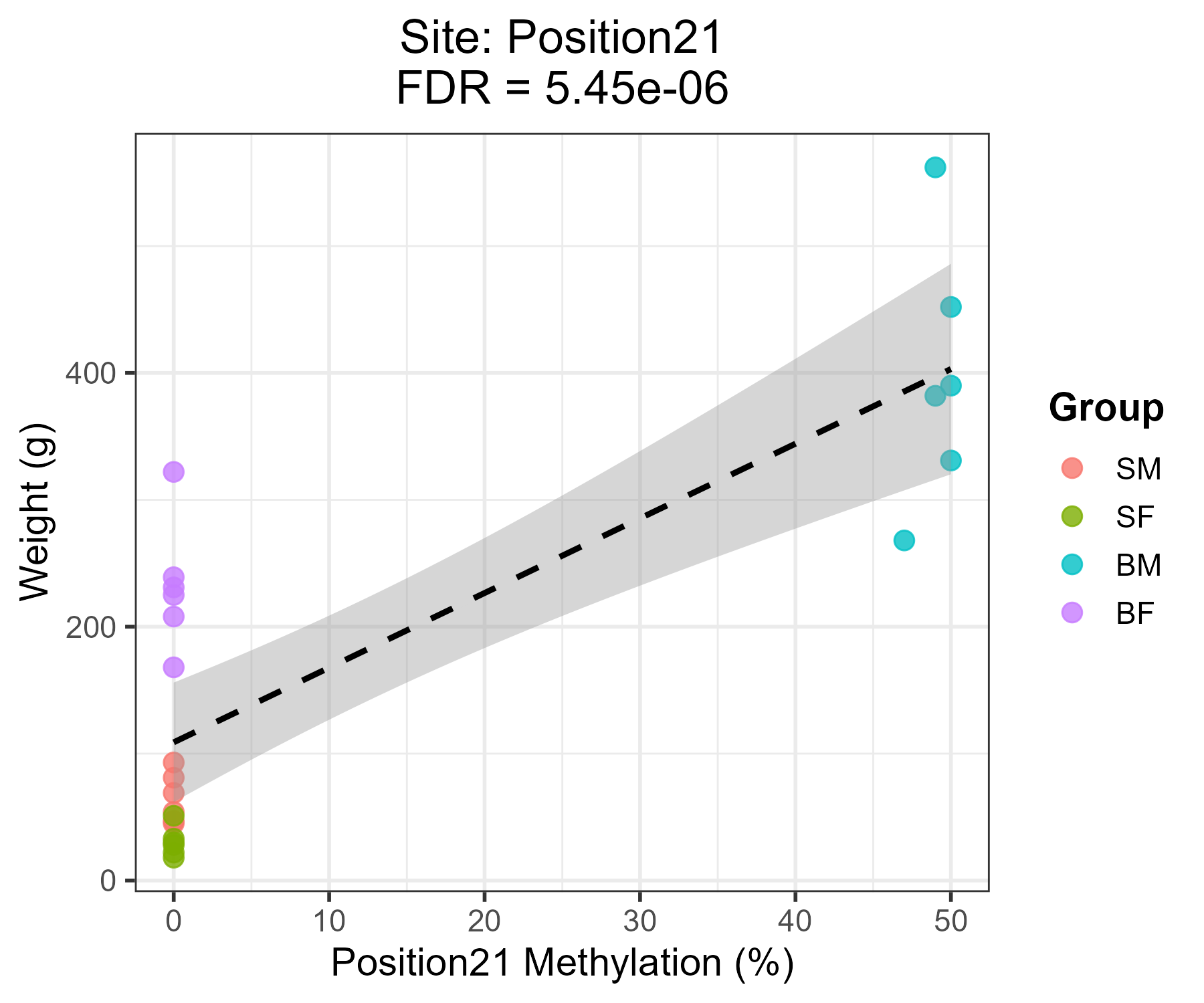

Supplement: Supplementary file 4 [file DataSheet2.zip › Regression_Minus_Strand/Position21_regression.tiff]

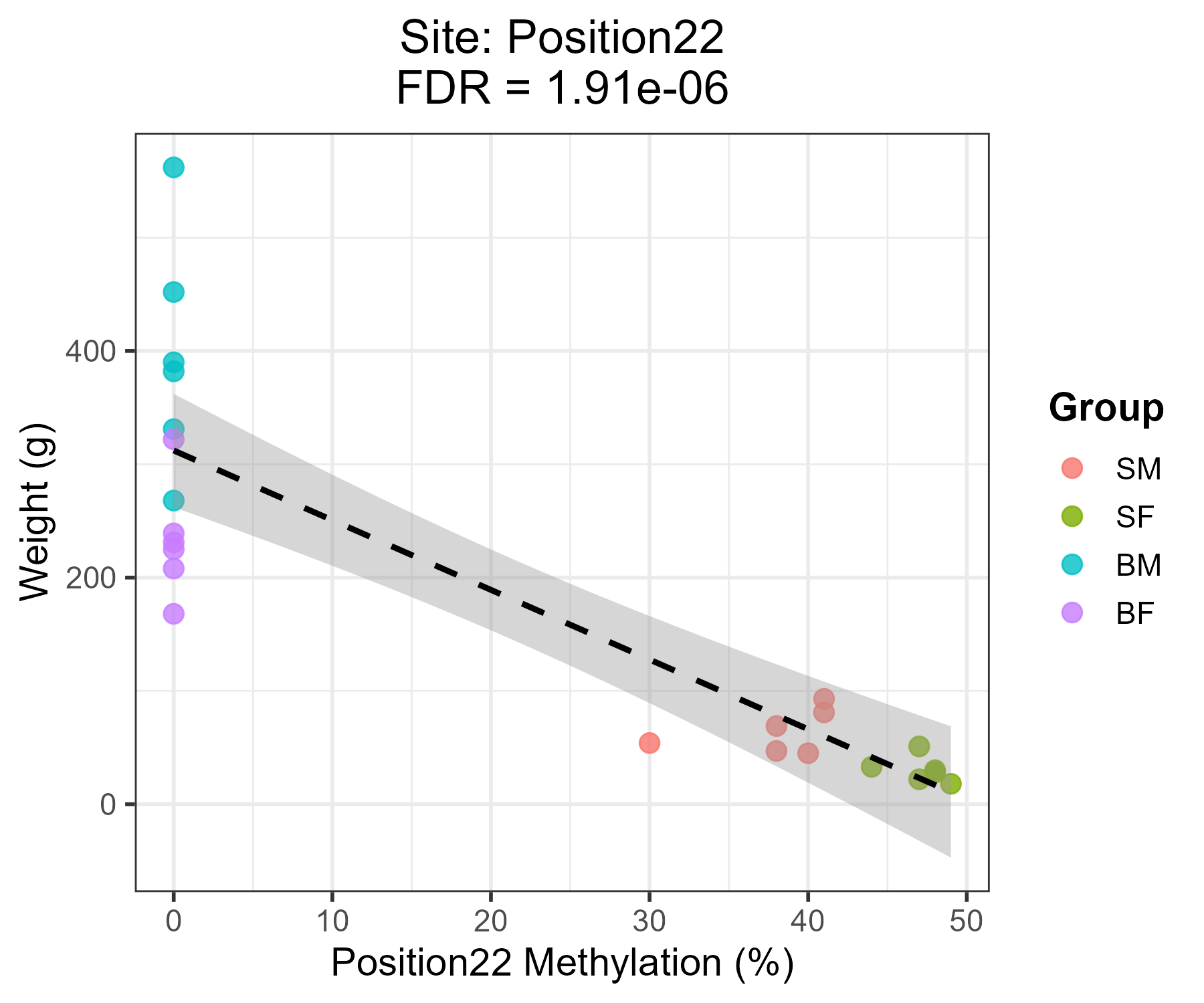

Supplement: Supplementary file 4 [file DataSheet2.zip › Regression_Minus_Strand/Position22_regression.tiff]

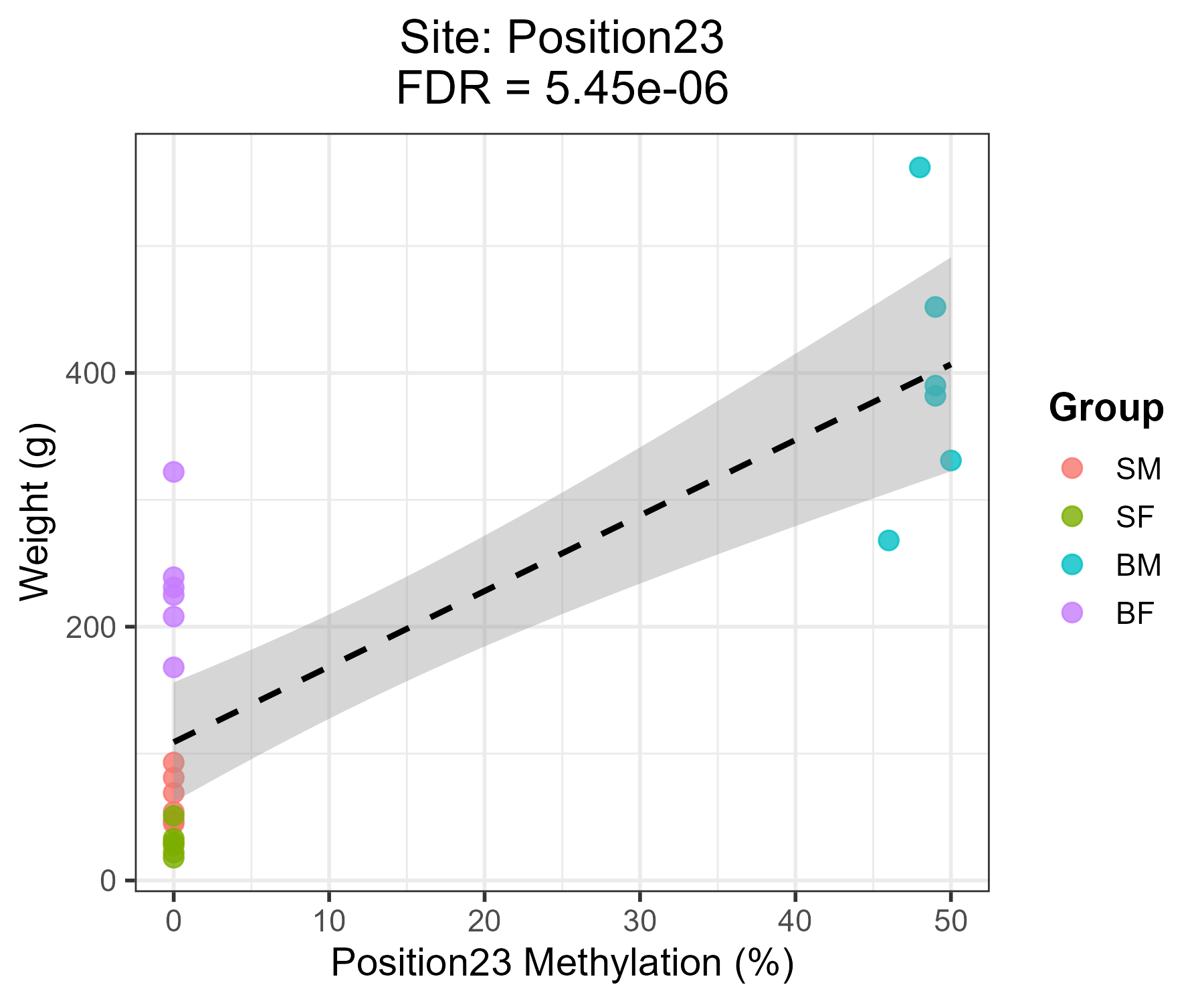

Supplement: Supplementary file 4 [file DataSheet2.zip › Regression_Minus_Strand/Position23_regression.tiff]

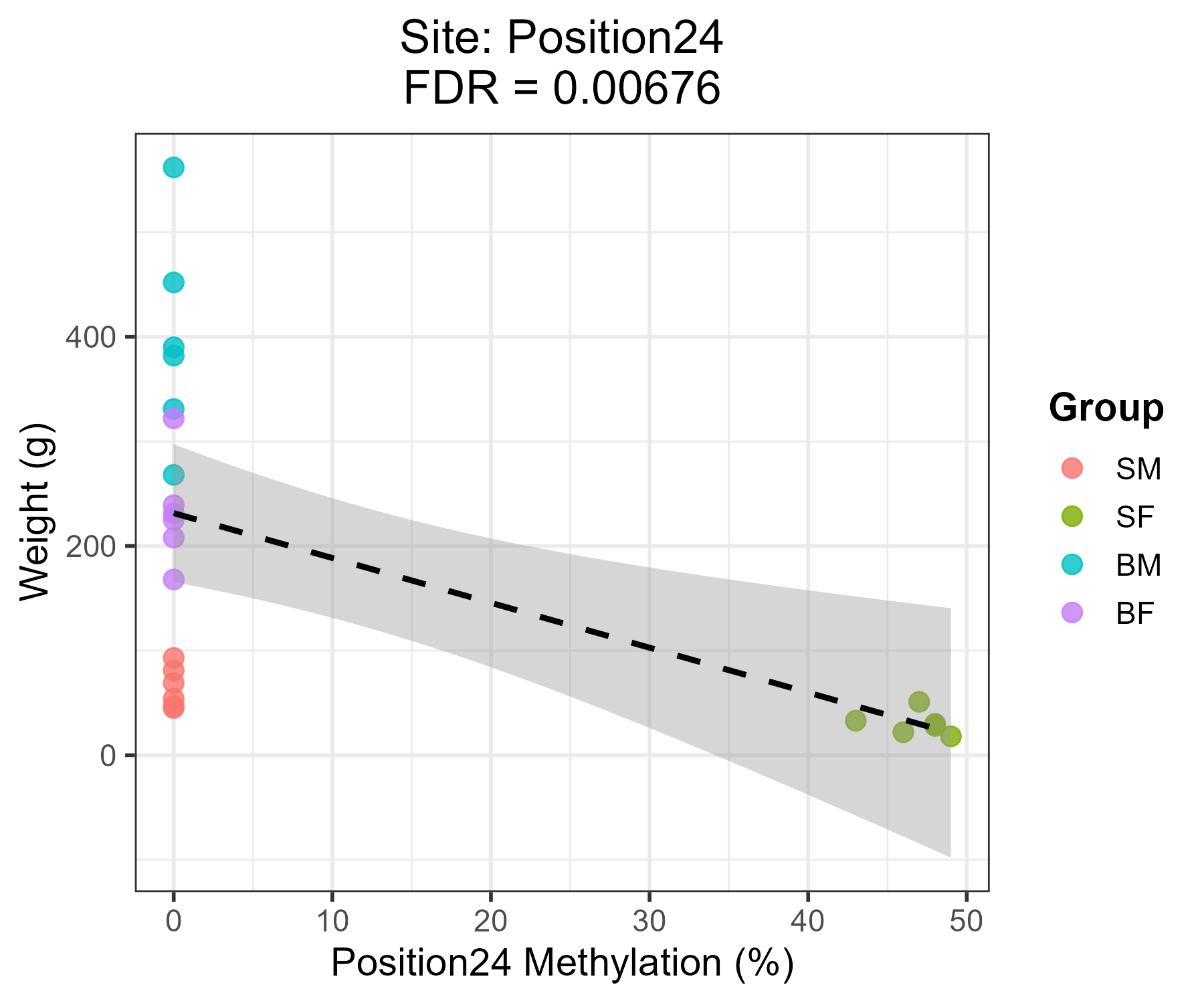

Supplement: Supplementary file 4 [file DataSheet2.zip › Regression_Minus_Strand/Position24_regression.tiff]

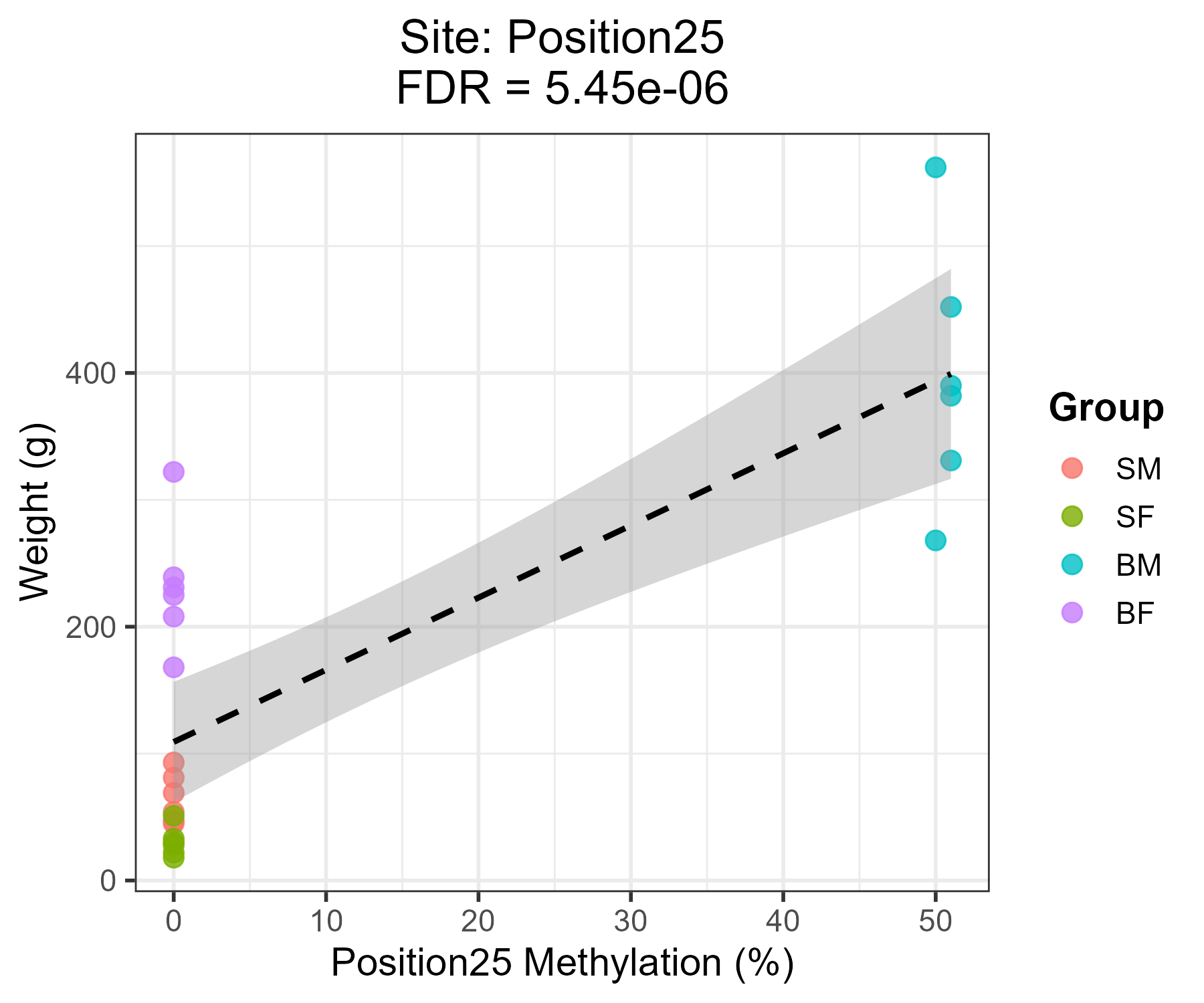

Supplement: Supplementary file 4 [file DataSheet2.zip › Regression_Minus_Strand/Position25_regression.tiff]

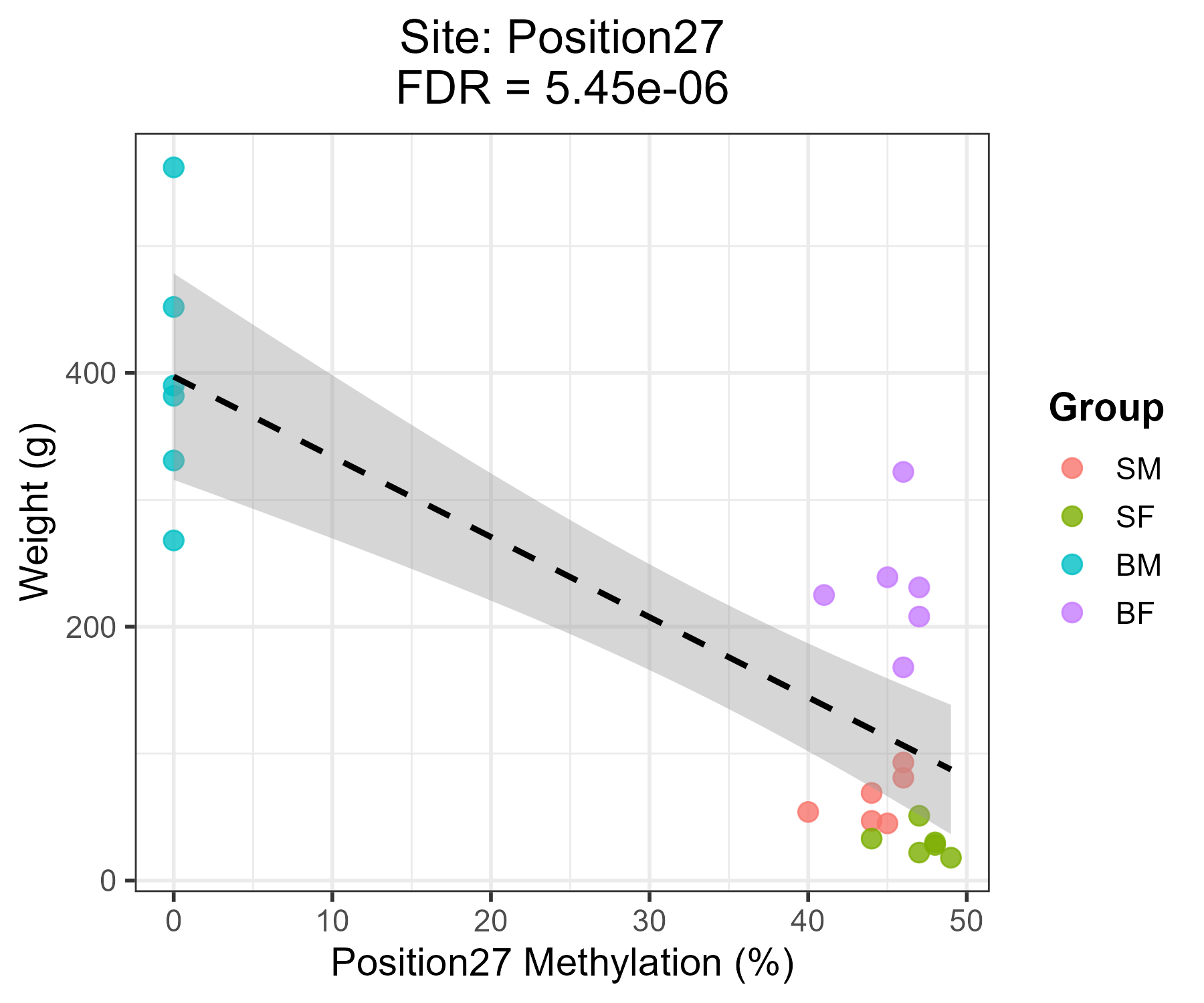

Supplement: Supplementary file 4 [file DataSheet2.zip › Regression_Minus_Strand/Position27_regression.tiff]

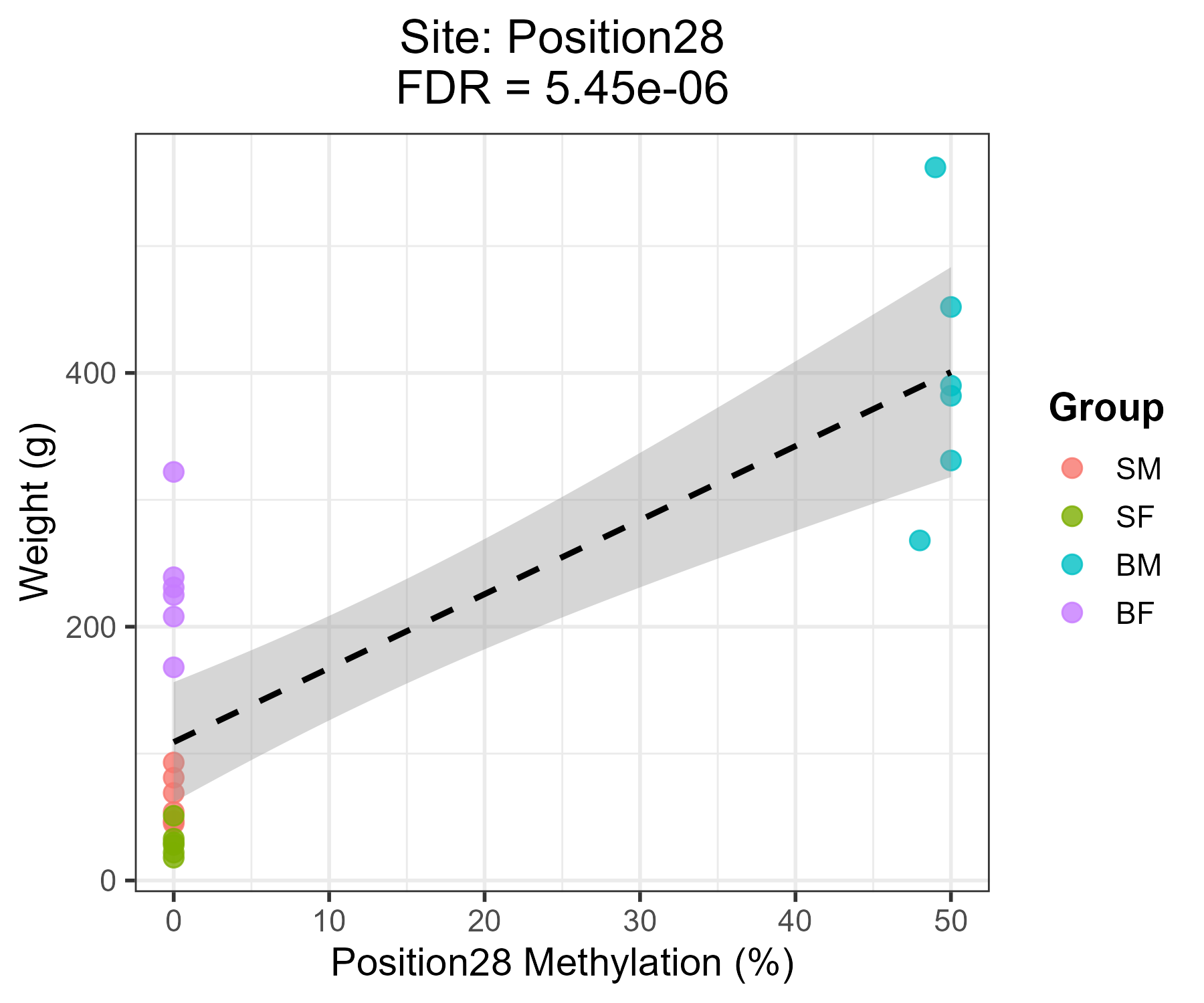

Supplement: Supplementary file 4 [file DataSheet2.zip › Regression_Minus_Strand/Position28_regression.tiff]

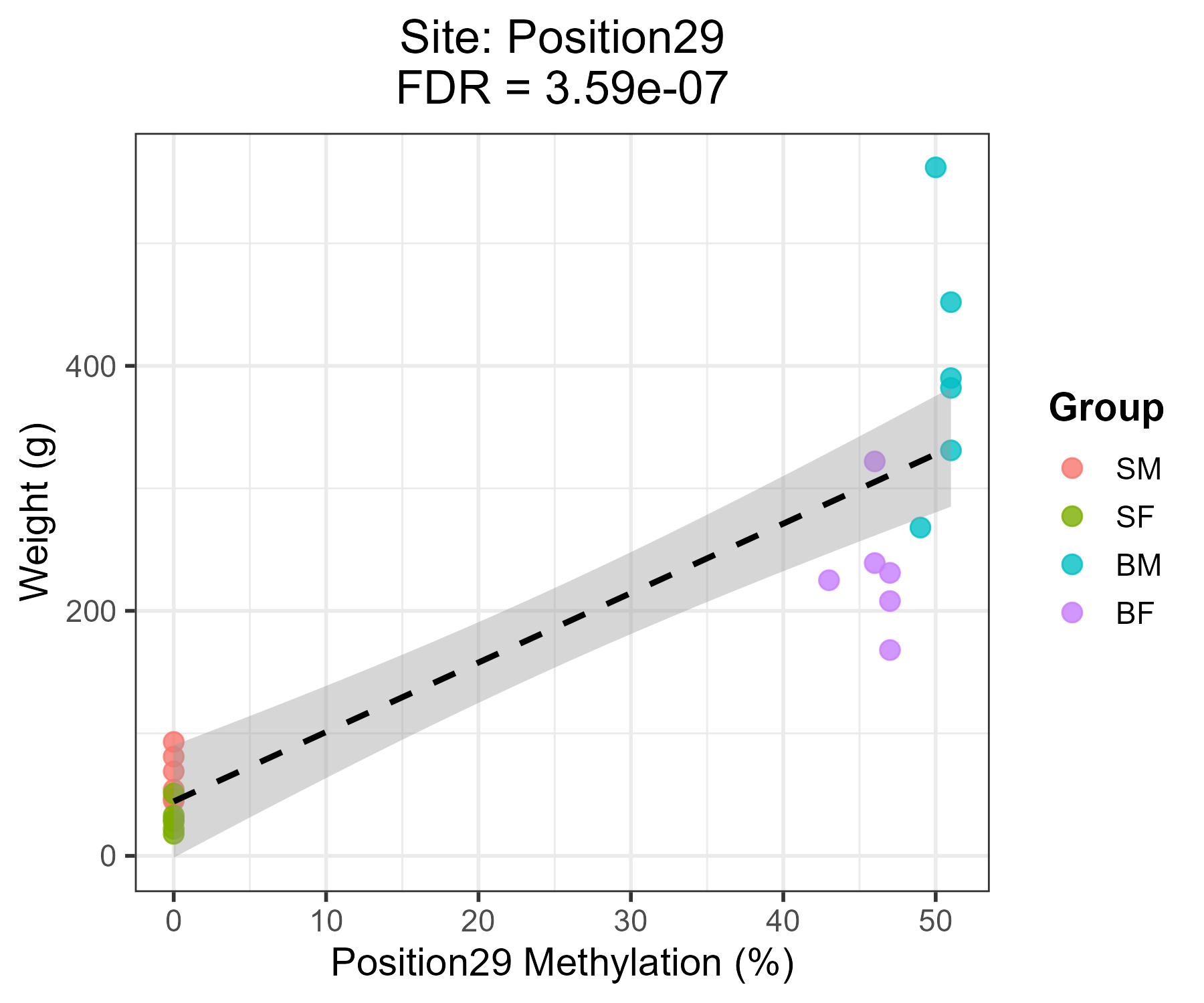

Supplement: Supplementary file 4 [file DataSheet2.zip › Regression_Minus_Strand/Position29_regression.tiff]

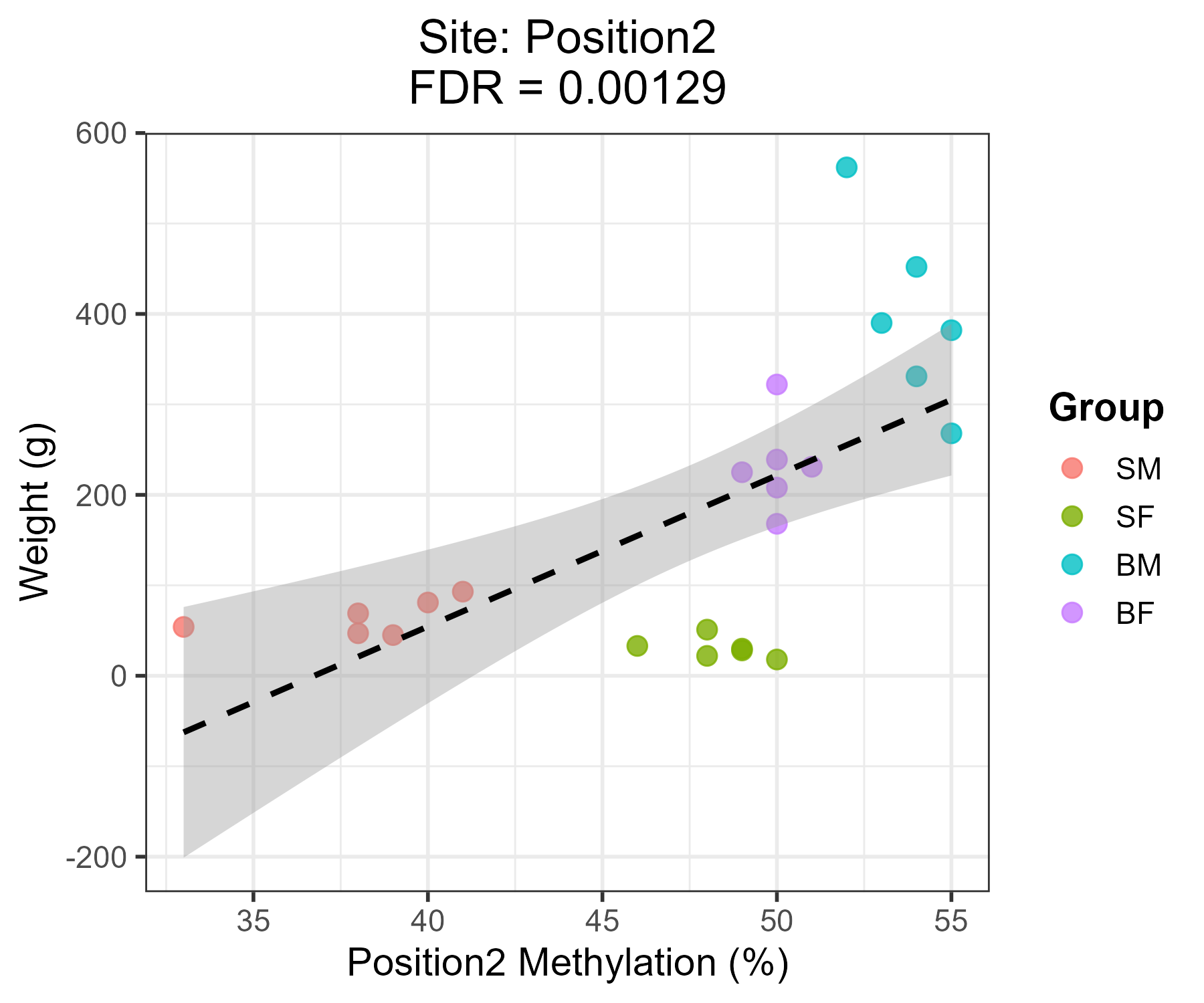

Supplement: Supplementary file 4 [file DataSheet2.zip › Regression_Minus_Strand/Position2_regression.tiff]

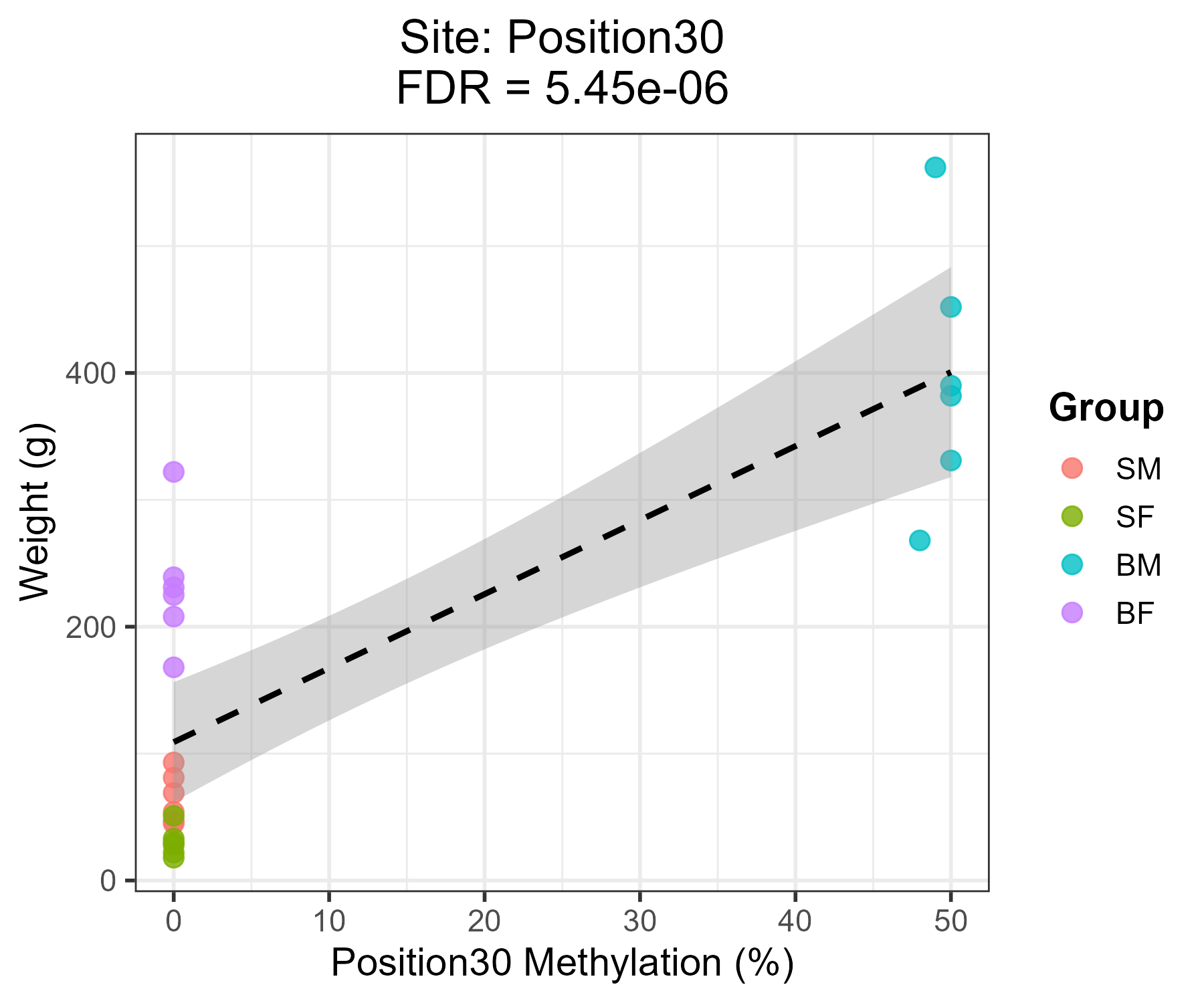

Supplement: Supplementary file 4 [file DataSheet2.zip › Regression_Minus_Strand/Position30_regression.tiff]

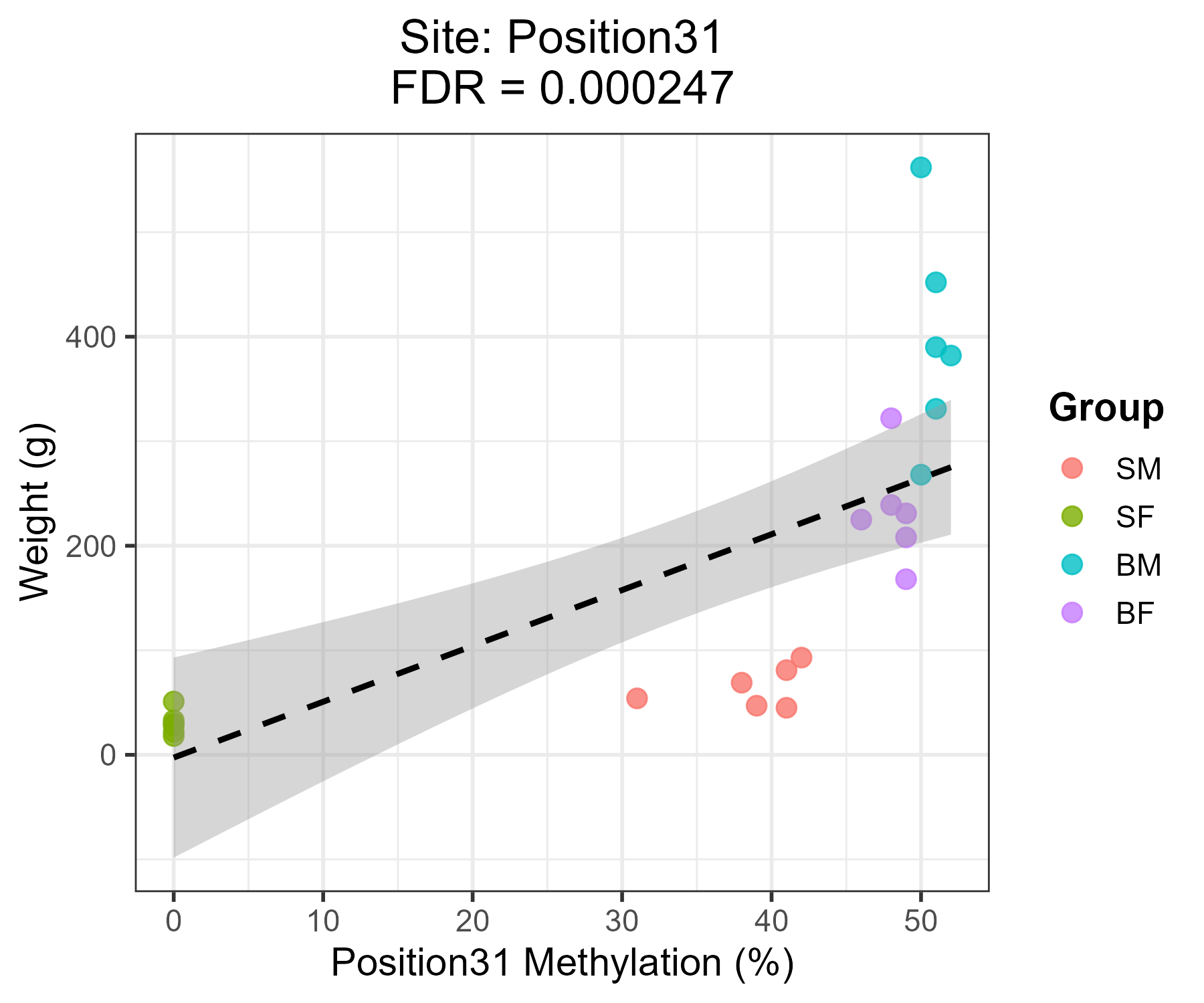

Supplement: Supplementary file 4 [file DataSheet2.zip › Regression_Minus_Strand/Position31_regression.tiff]

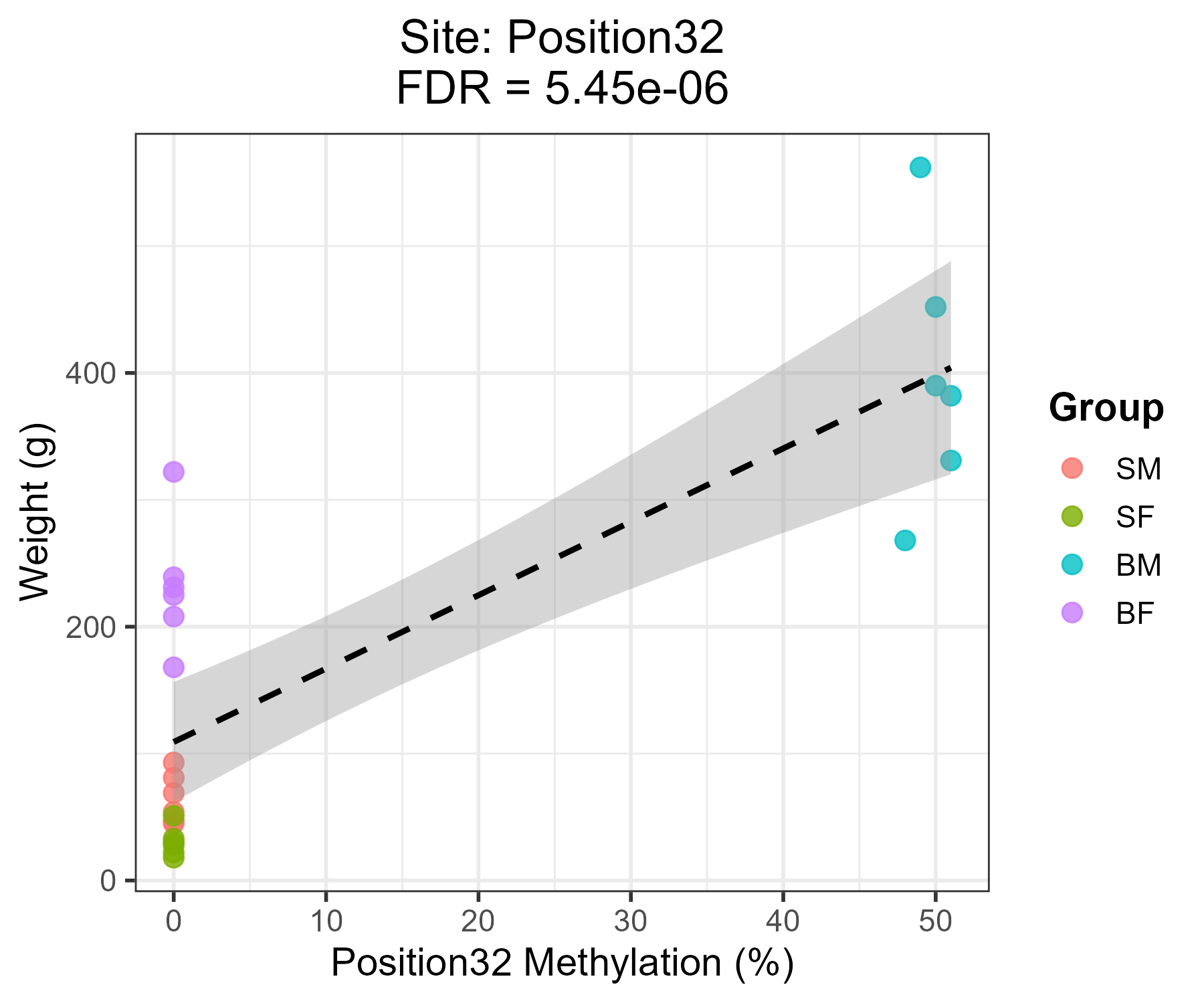

Supplement: Supplementary file 4 [file DataSheet2.zip › Regression_Minus_Strand/Position32_regression.tiff]

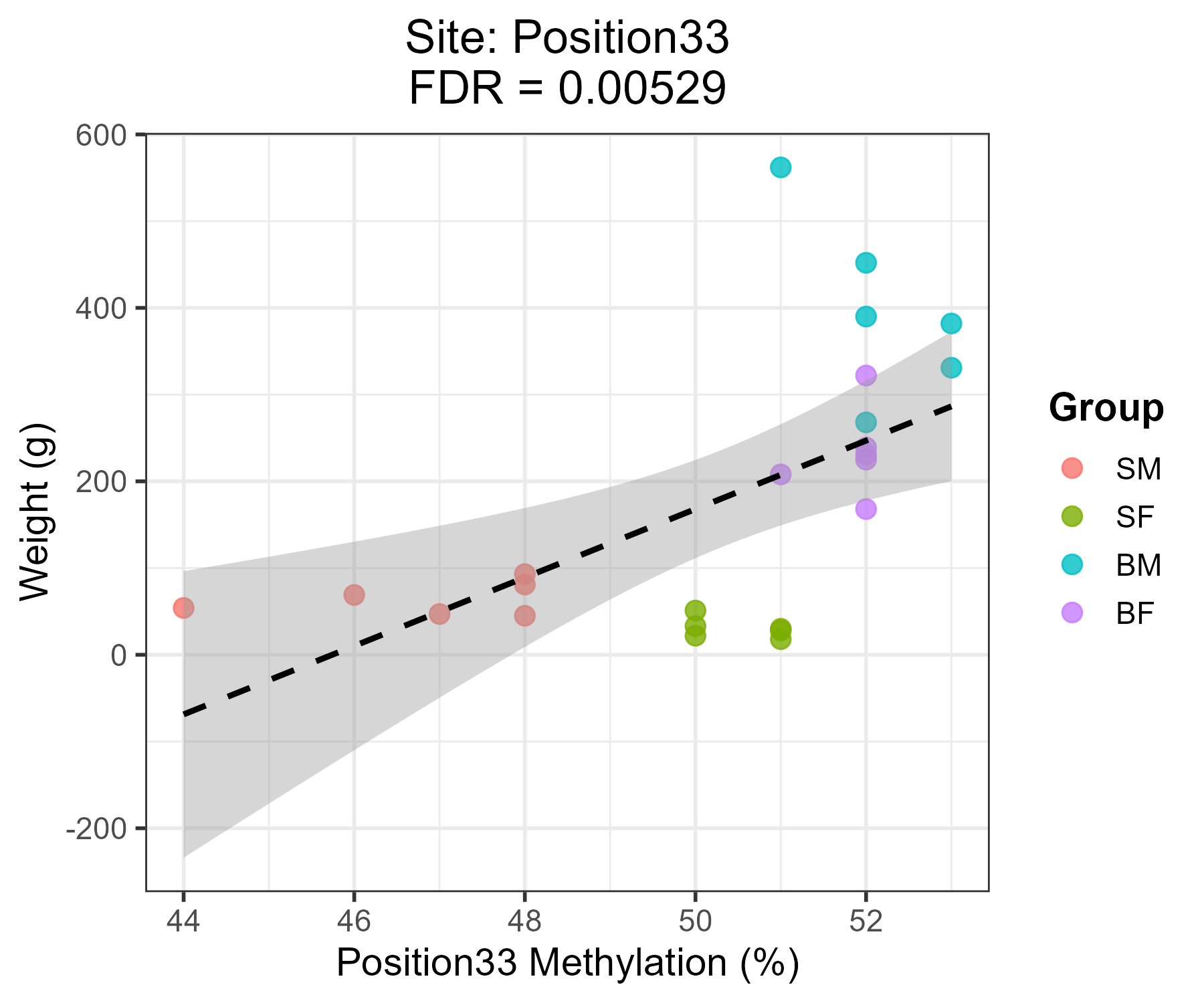

Supplement: Supplementary file 4 [file DataSheet2.zip › Regression_Minus_Strand/Position33_regression.tiff]

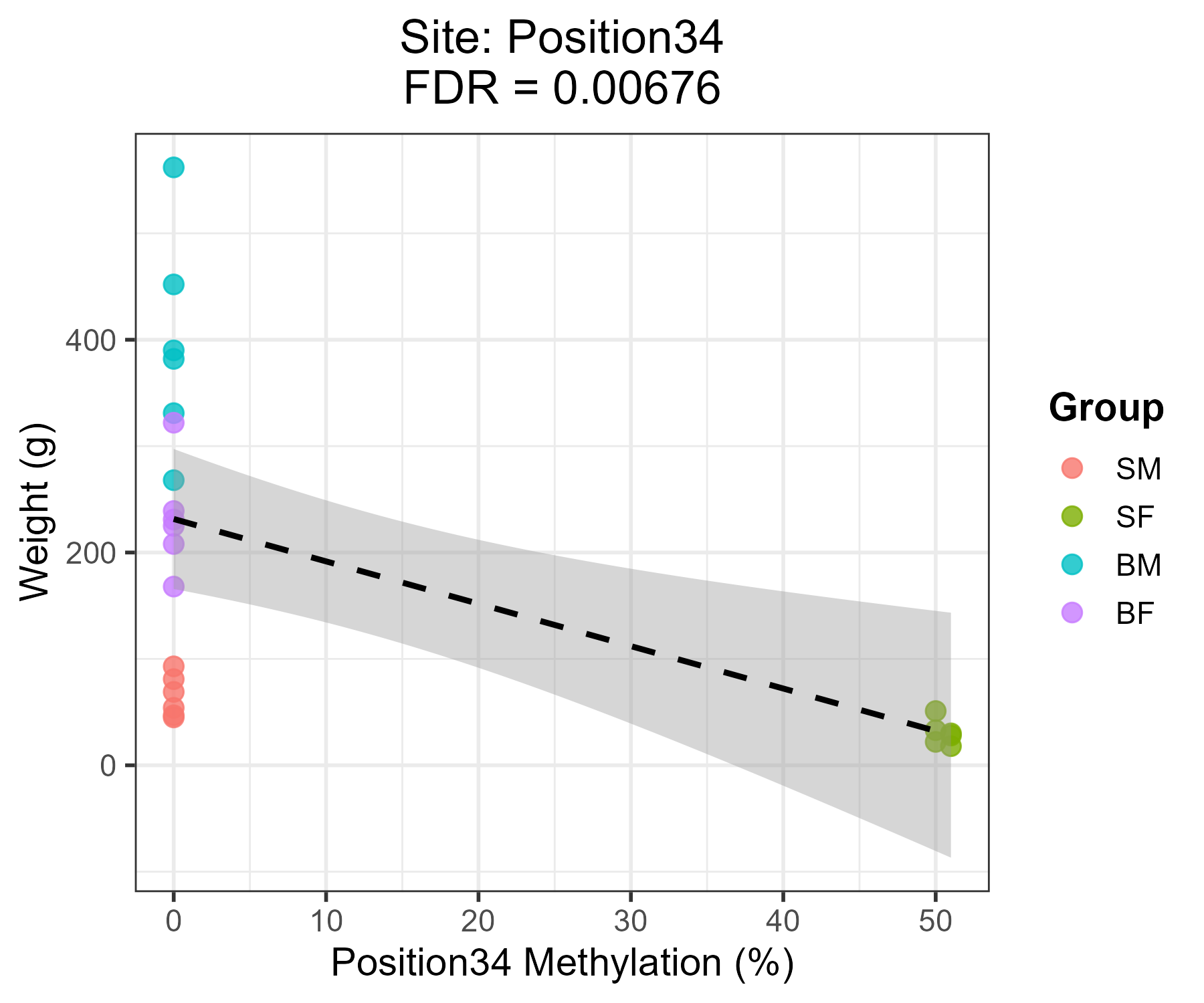

Supplement: Supplementary file 4 [file DataSheet2.zip › Regression_Minus_Strand/Position34_regression.tiff]

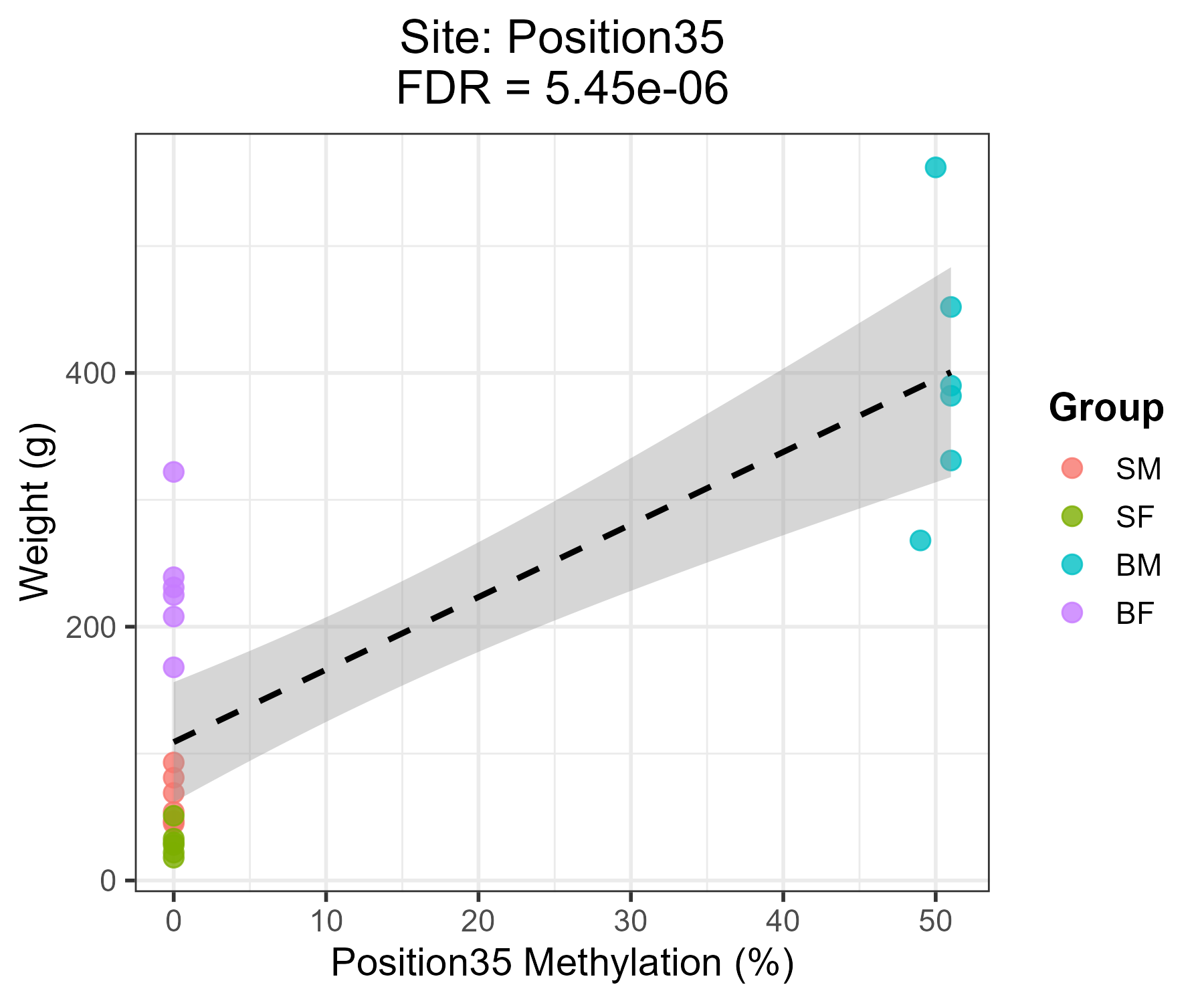

Supplement: Supplementary file 4 [file DataSheet2.zip › Regression_Minus_Strand/Position35_regression.tiff]

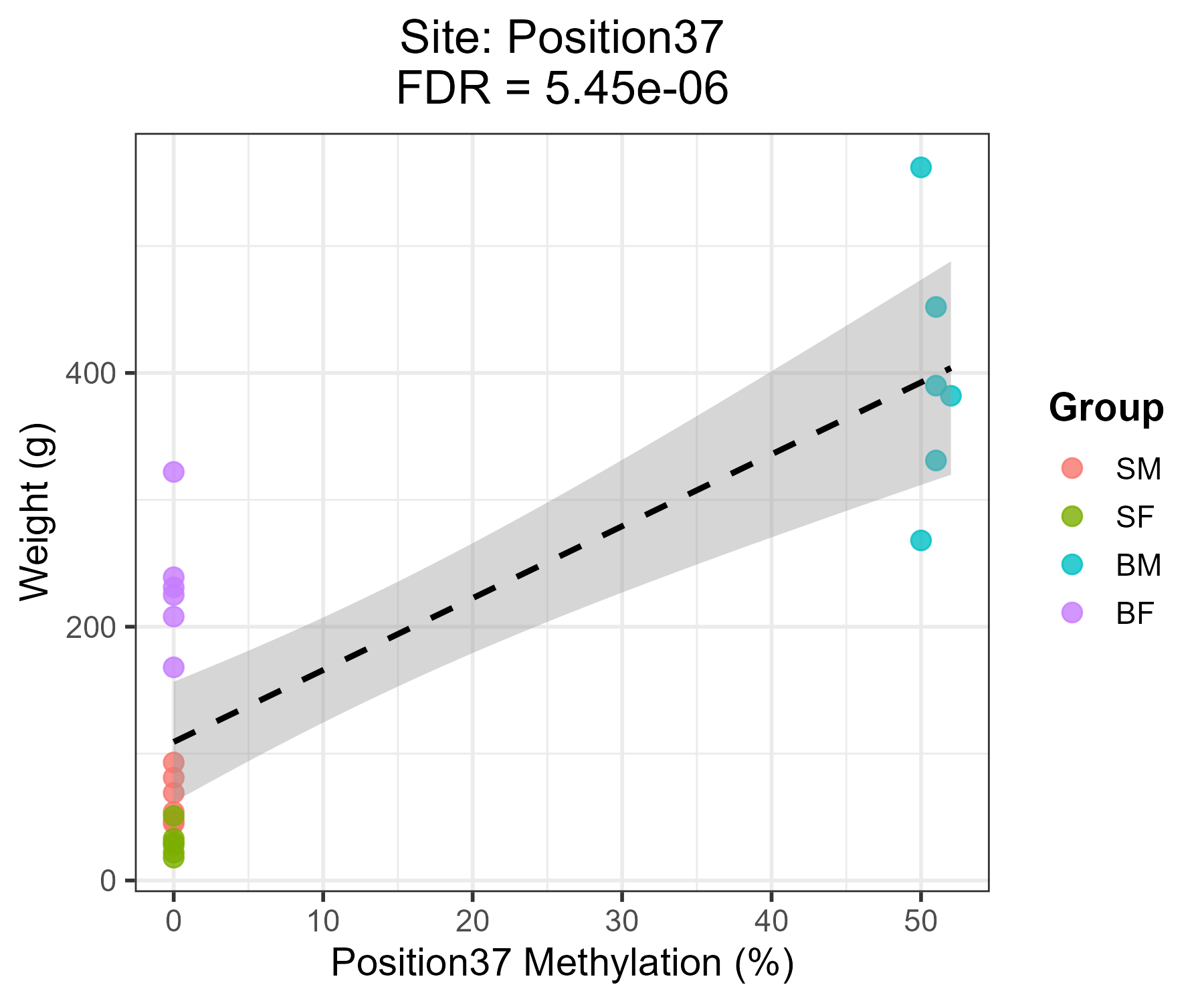

Supplement: Supplementary file 4 [file DataSheet2.zip › Regression_Minus_Strand/Position37_regression.tiff]

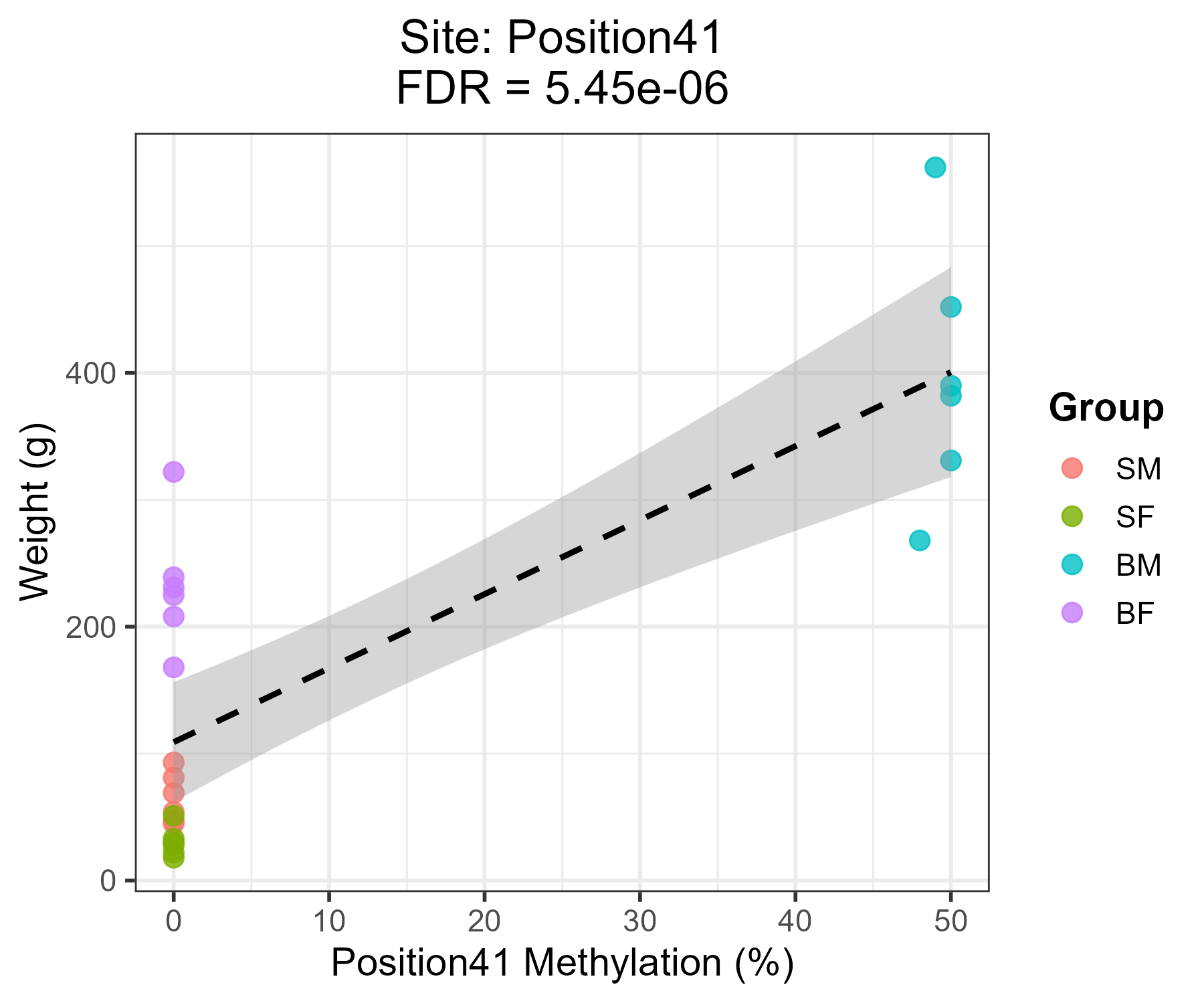

Supplement: Supplementary file 4 [file DataSheet2.zip › Regression_Minus_Strand/Position41_regression.tiff]

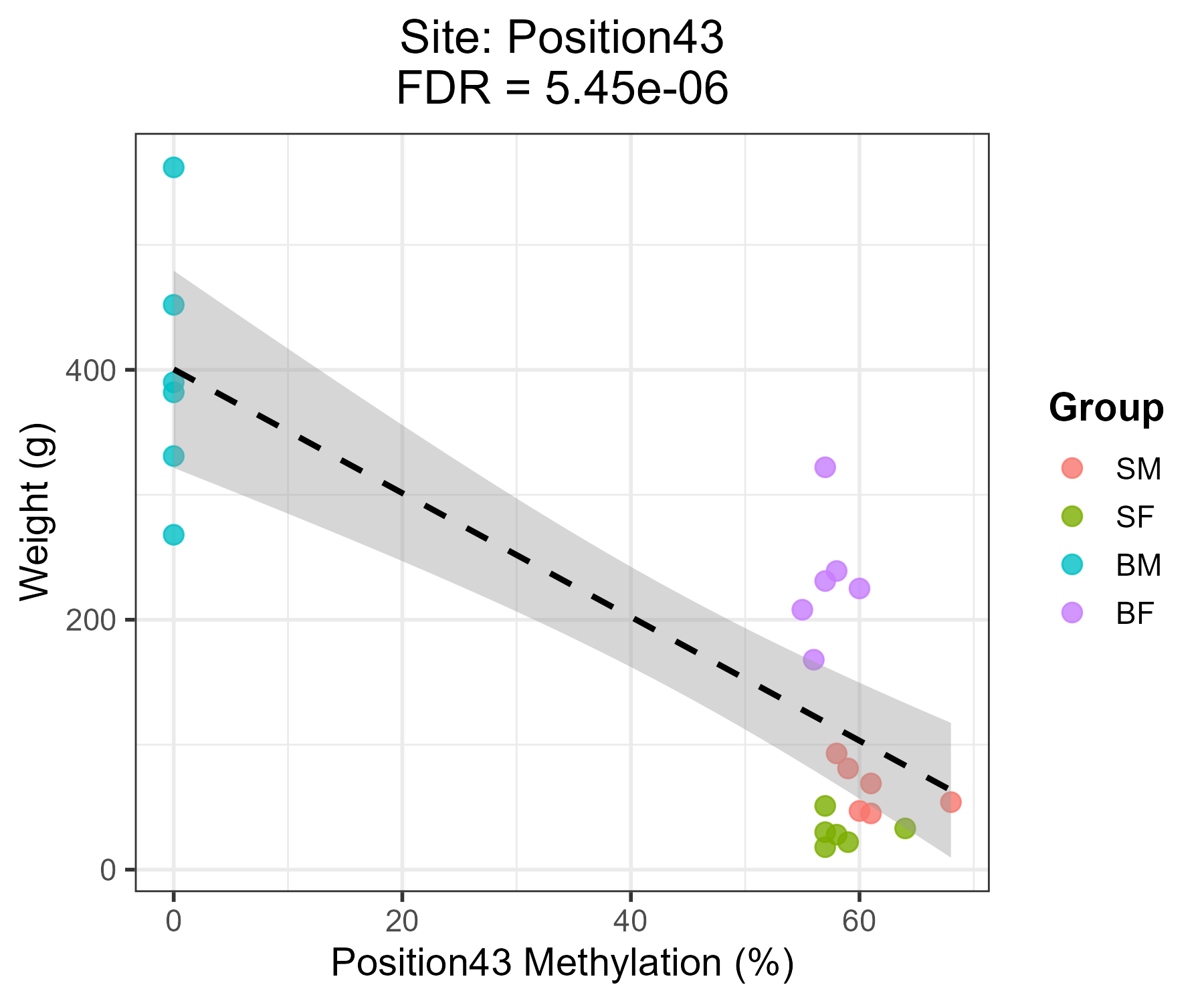

Supplement: Supplementary file 4 [file DataSheet2.zip › Regression_Minus_Strand/Position43_regression.tiff]

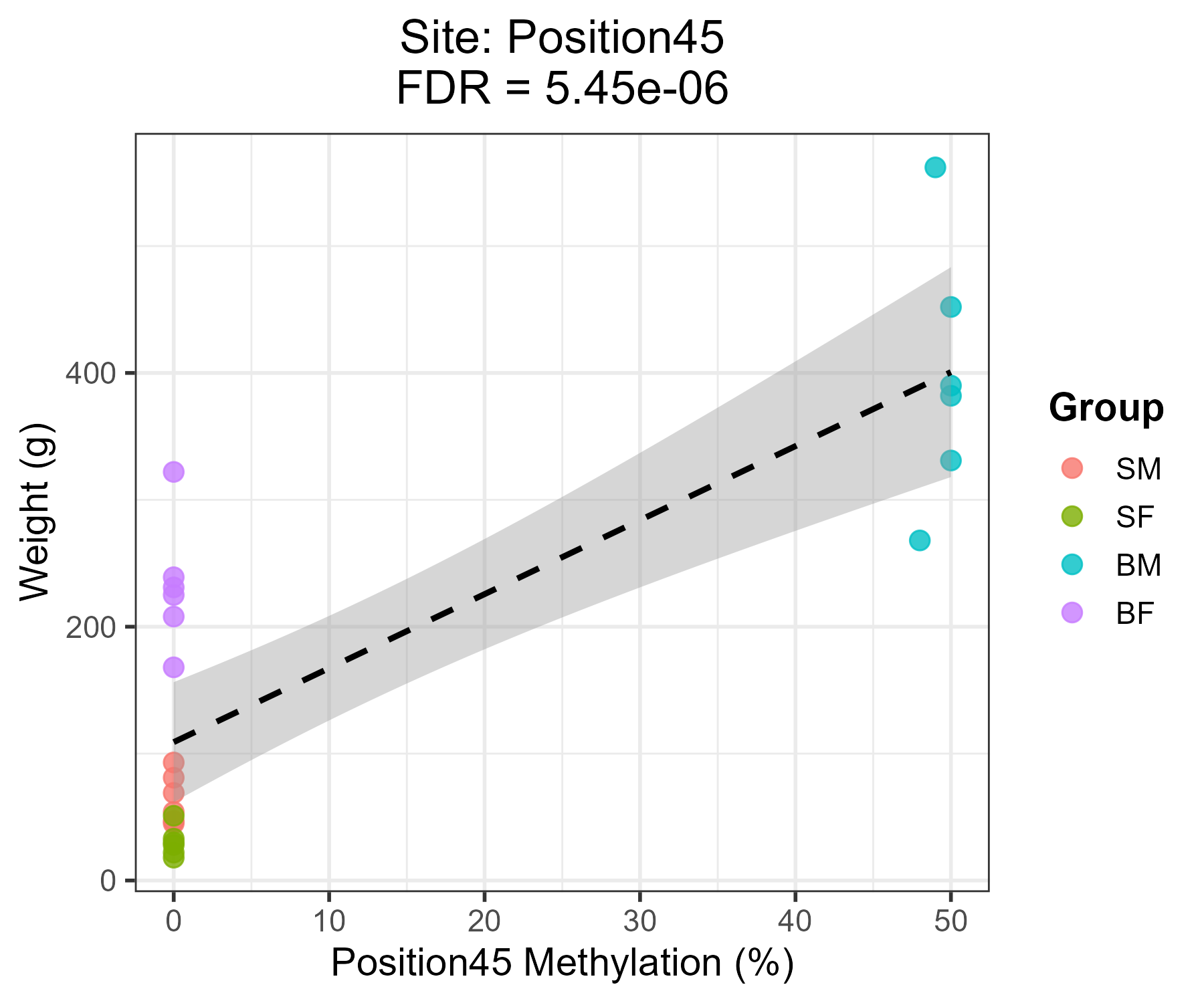

Supplement: Supplementary file 4 [file DataSheet2.zip › Regression_Minus_Strand/Position45_regression.tiff]

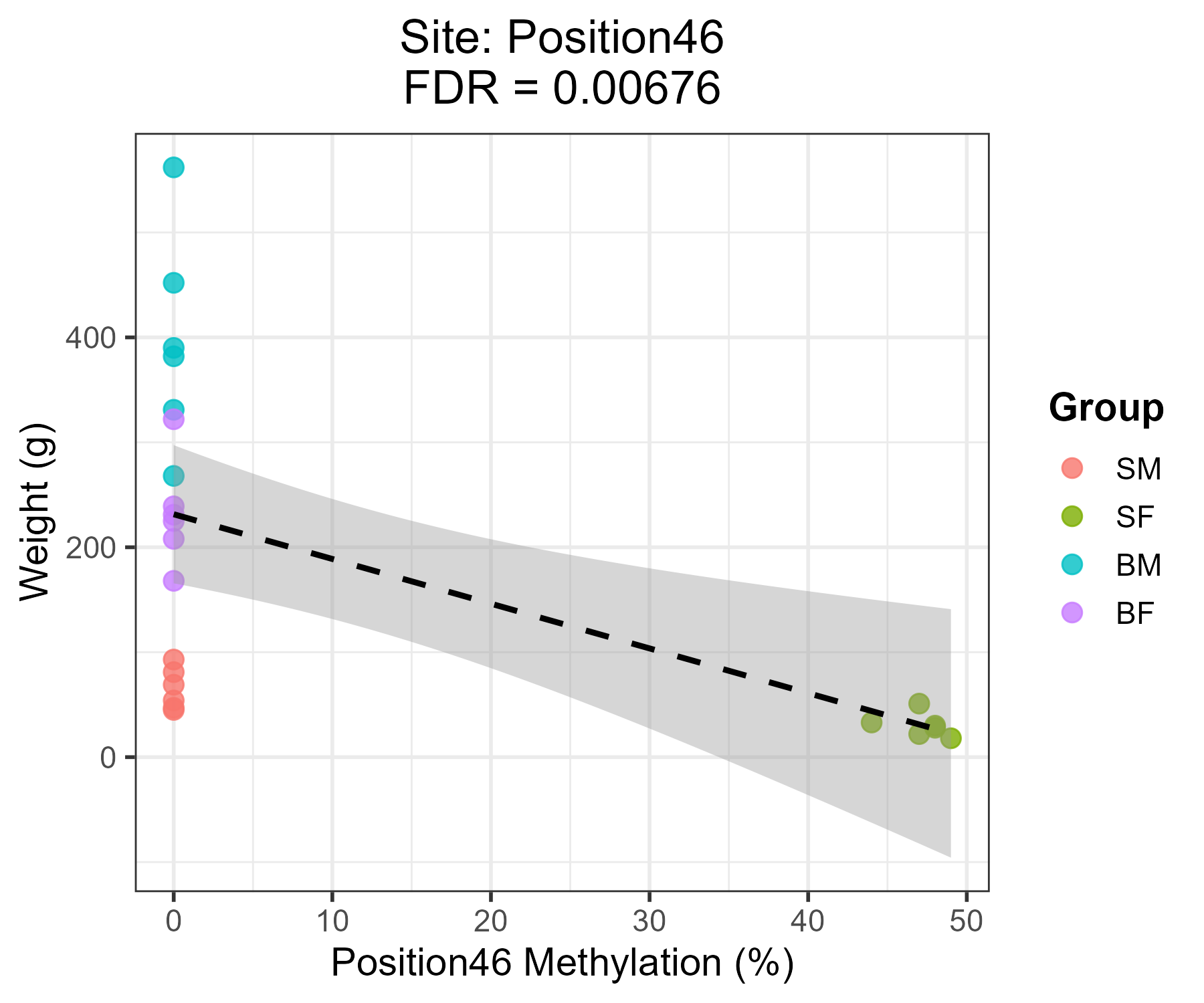

Supplement: Supplementary file 4 [file DataSheet2.zip › Regression_Minus_Strand/Position46_regression.tiff]

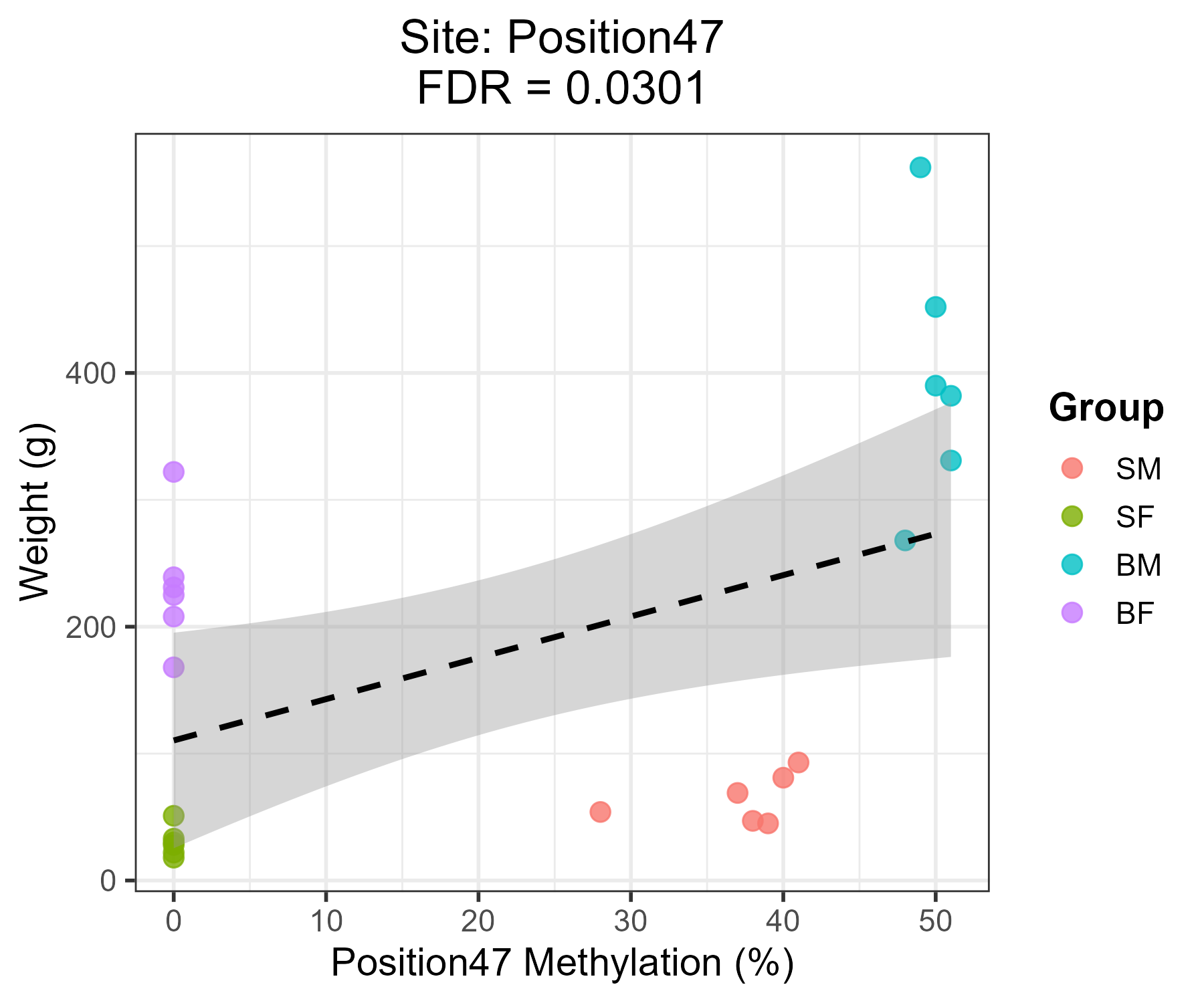

Supplement: Supplementary file 4 [file DataSheet2.zip › Regression_Minus_Strand/Position47_regression.tiff]

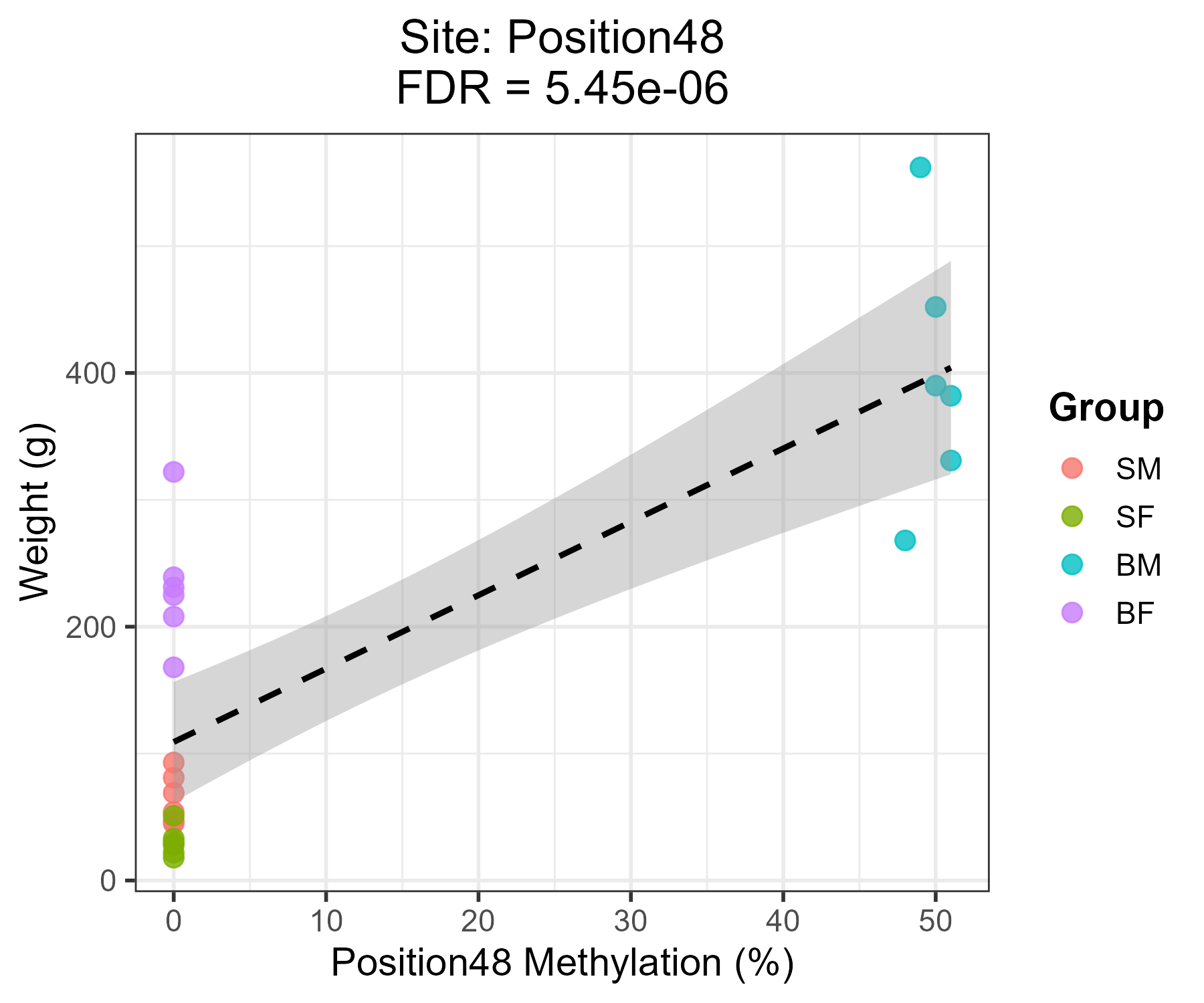

Supplement: Supplementary file 4 [file DataSheet2.zip › Regression_Minus_Strand/Position48_regression.tiff]

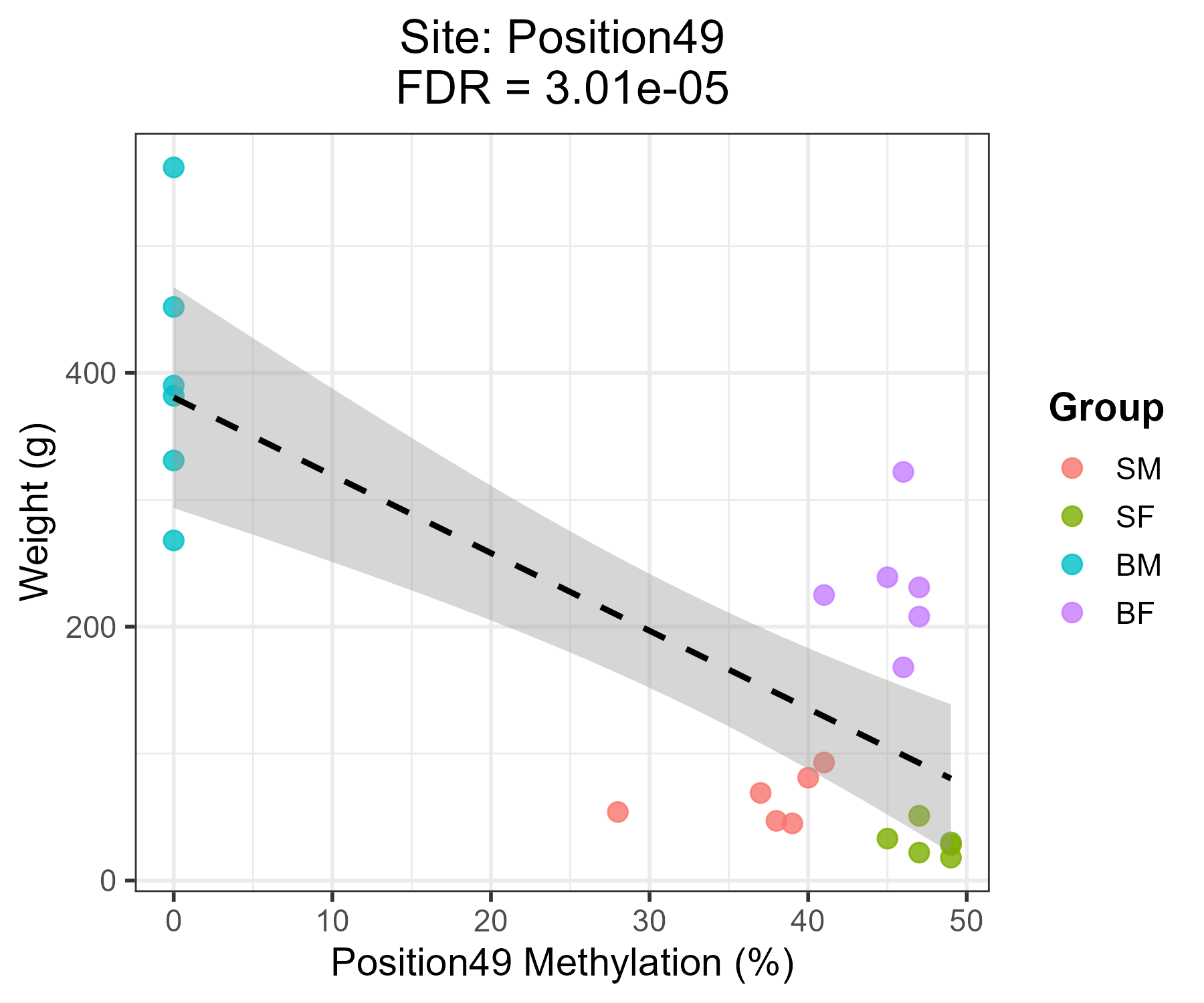

Supplement: Supplementary file 4 [file DataSheet2.zip › Regression_Minus_Strand/Position49_regression.tiff]

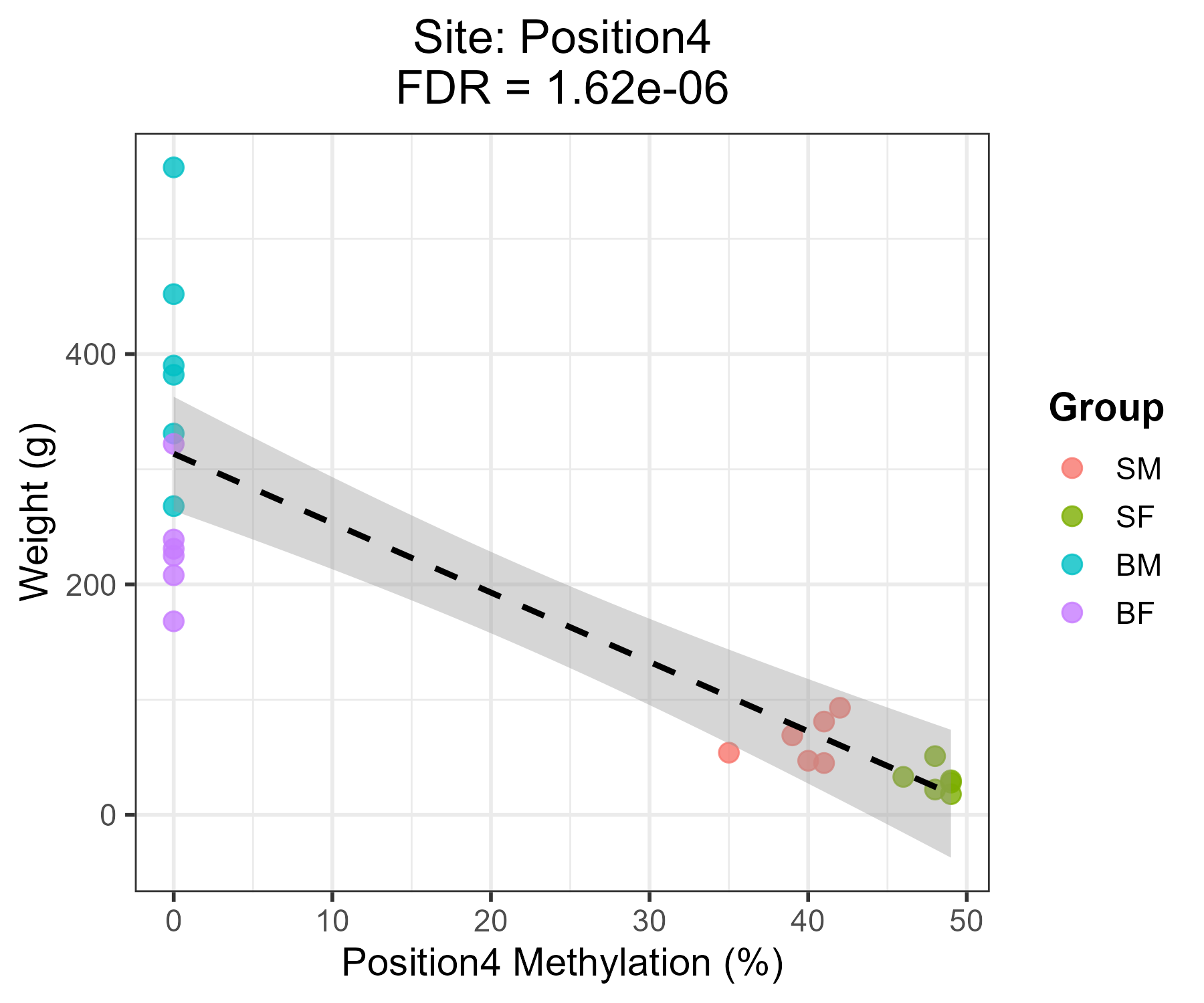

Supplement: Supplementary file 4 [file DataSheet2.zip › Regression_Minus_Strand/Position4_regression.tiff]

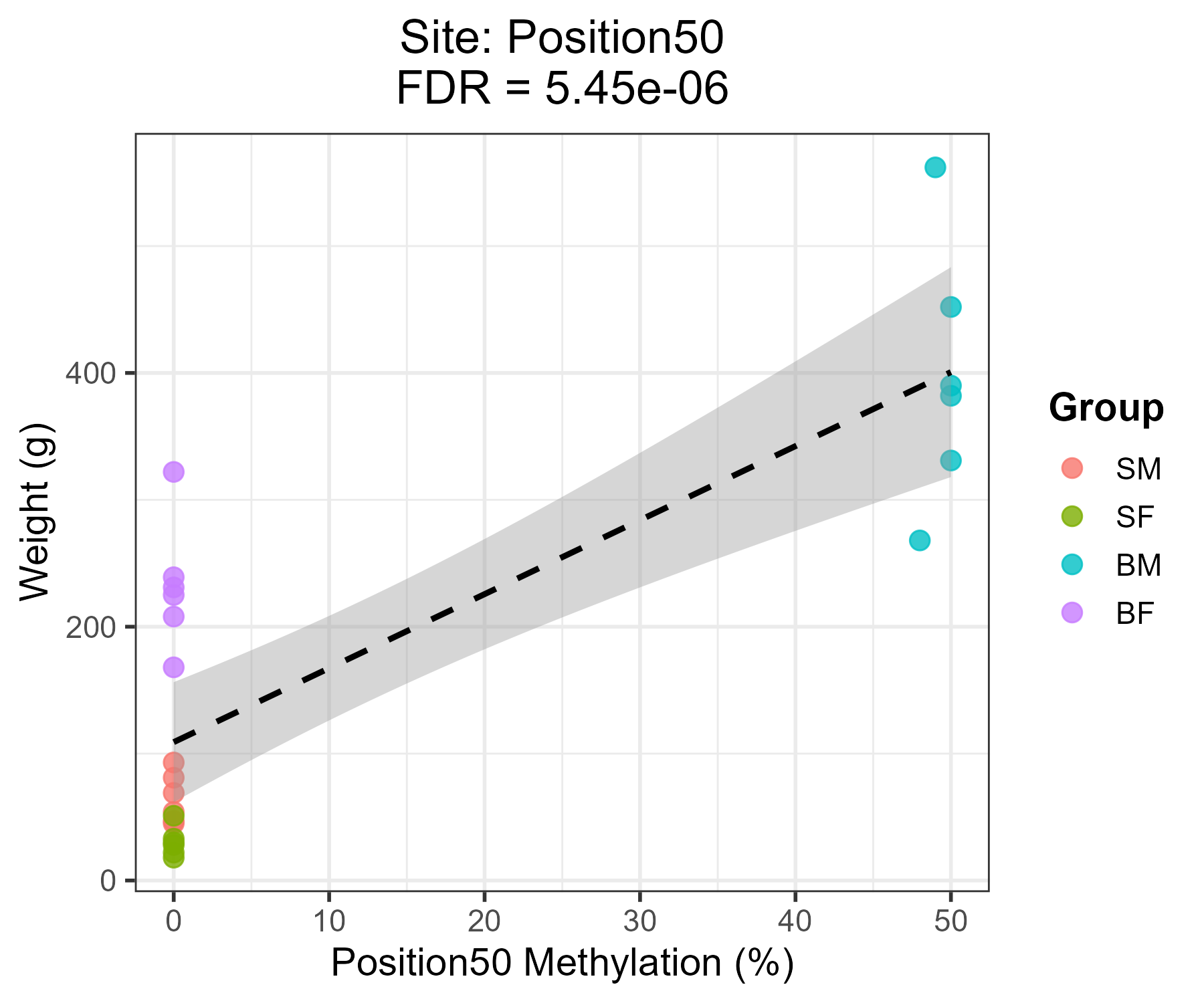

Supplement: Supplementary file 4 [file DataSheet2.zip › Regression_Minus_Strand/Position50_regression.tiff]

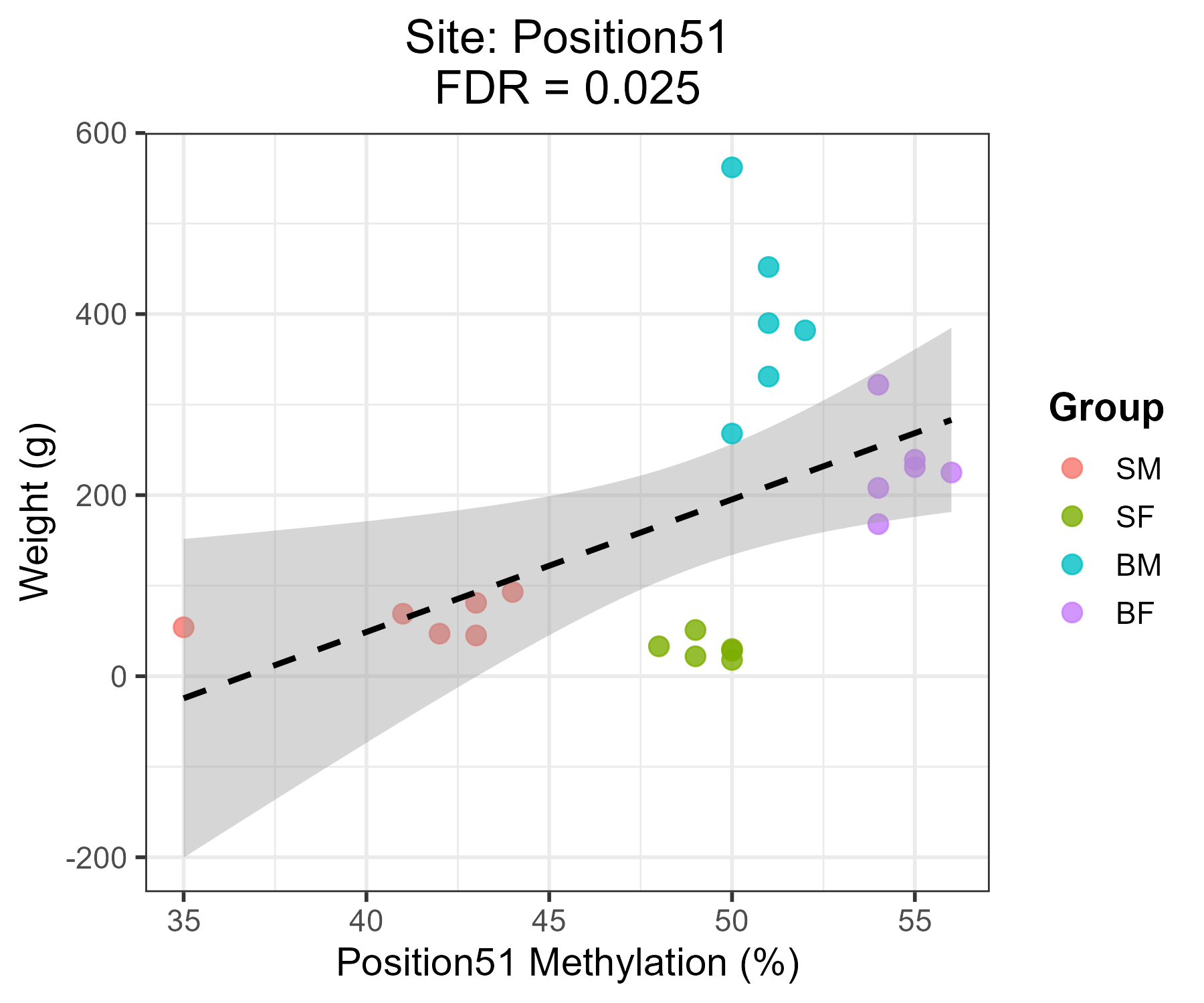

Supplement: Supplementary file 4 [file DataSheet2.zip › Regression_Minus_Strand/Position51_regression.tiff]

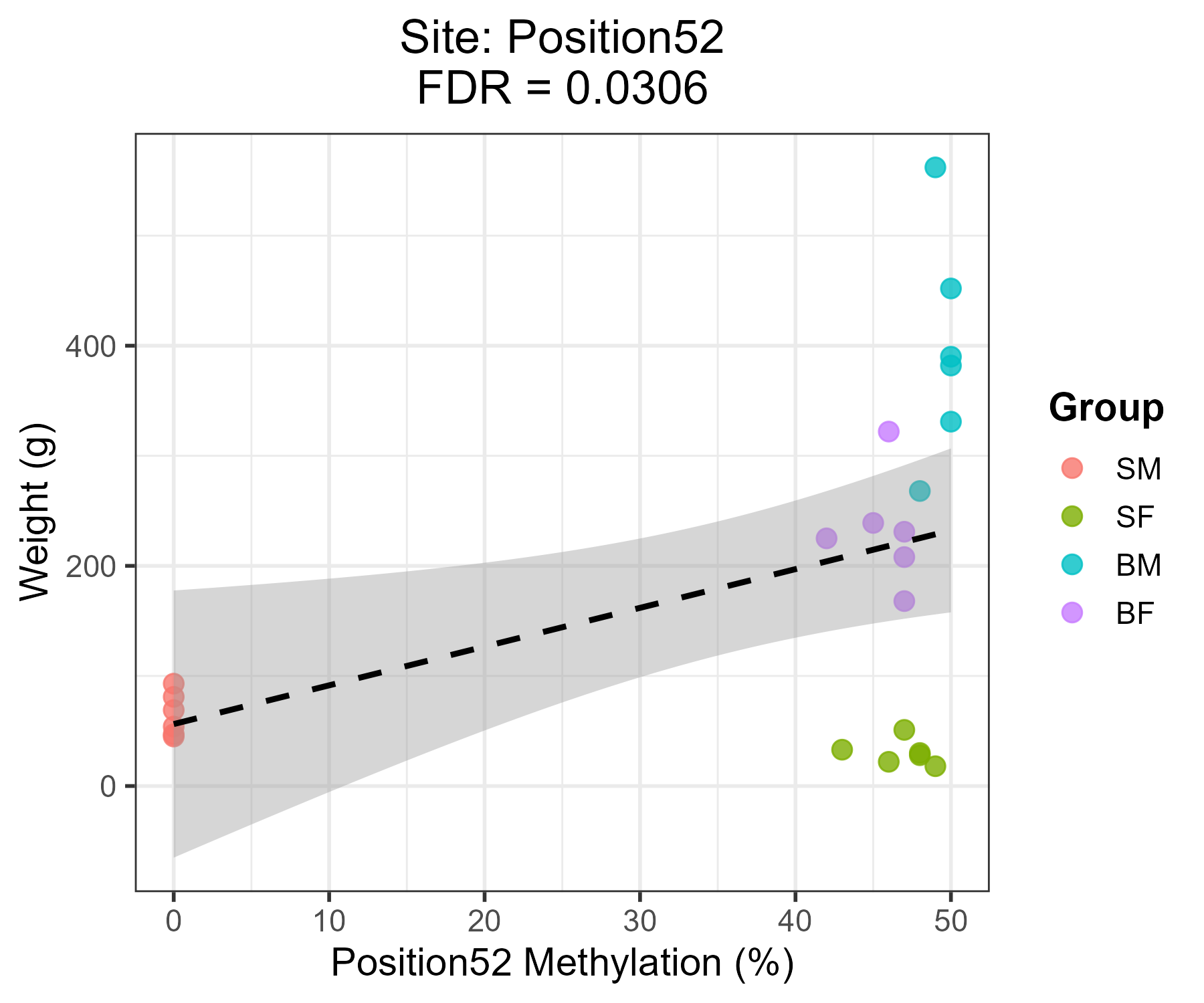

Supplement: Supplementary file 4 [file DataSheet2.zip › Regression_Minus_Strand/Position52_regression.tiff]

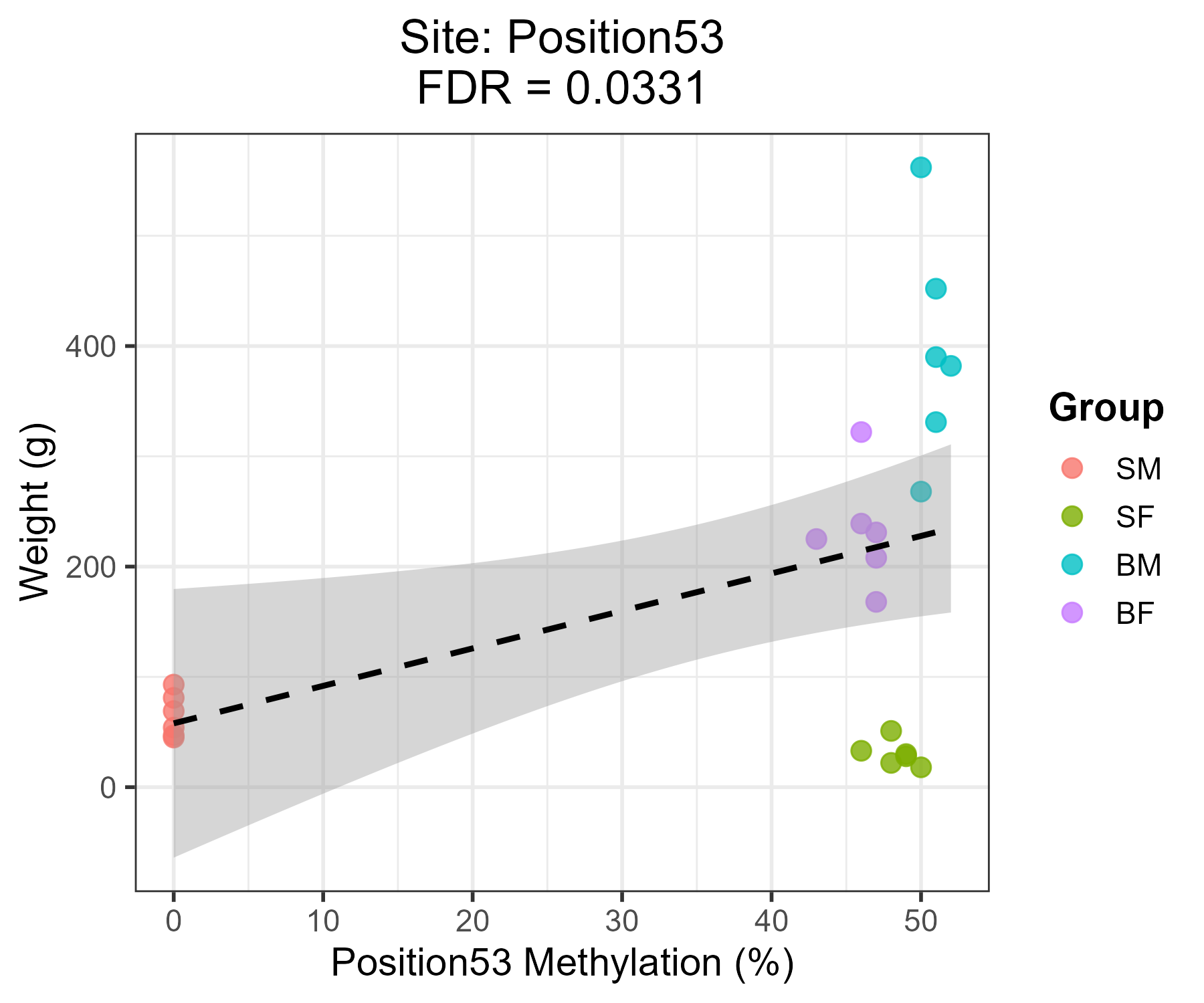

Supplement: Supplementary file 4 [file DataSheet2.zip › Regression_Minus_Strand/Position53_regression.tiff]

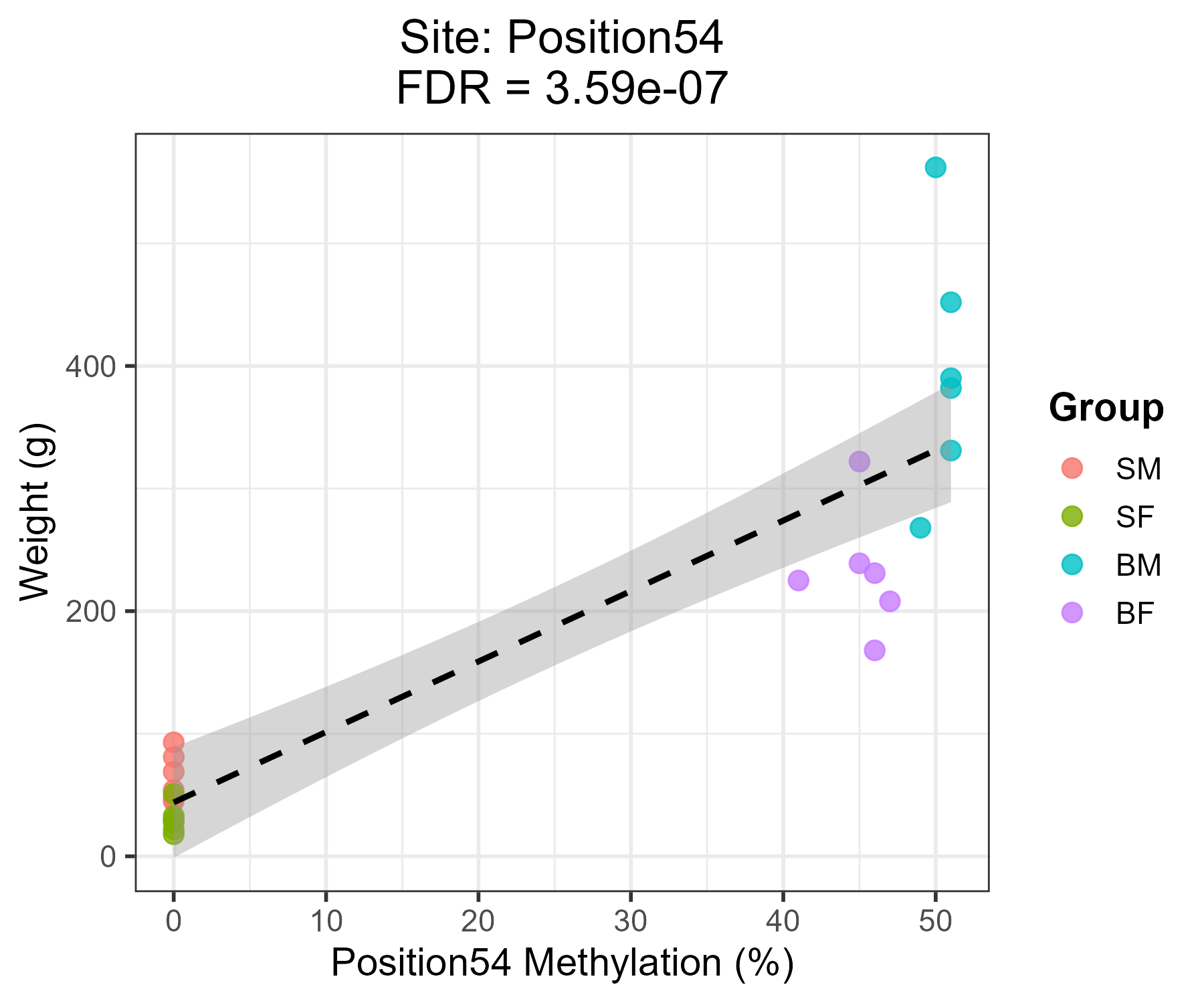

Supplement: Supplementary file 4 [file DataSheet2.zip › Regression_Minus_Strand/Position54_regression.tiff]

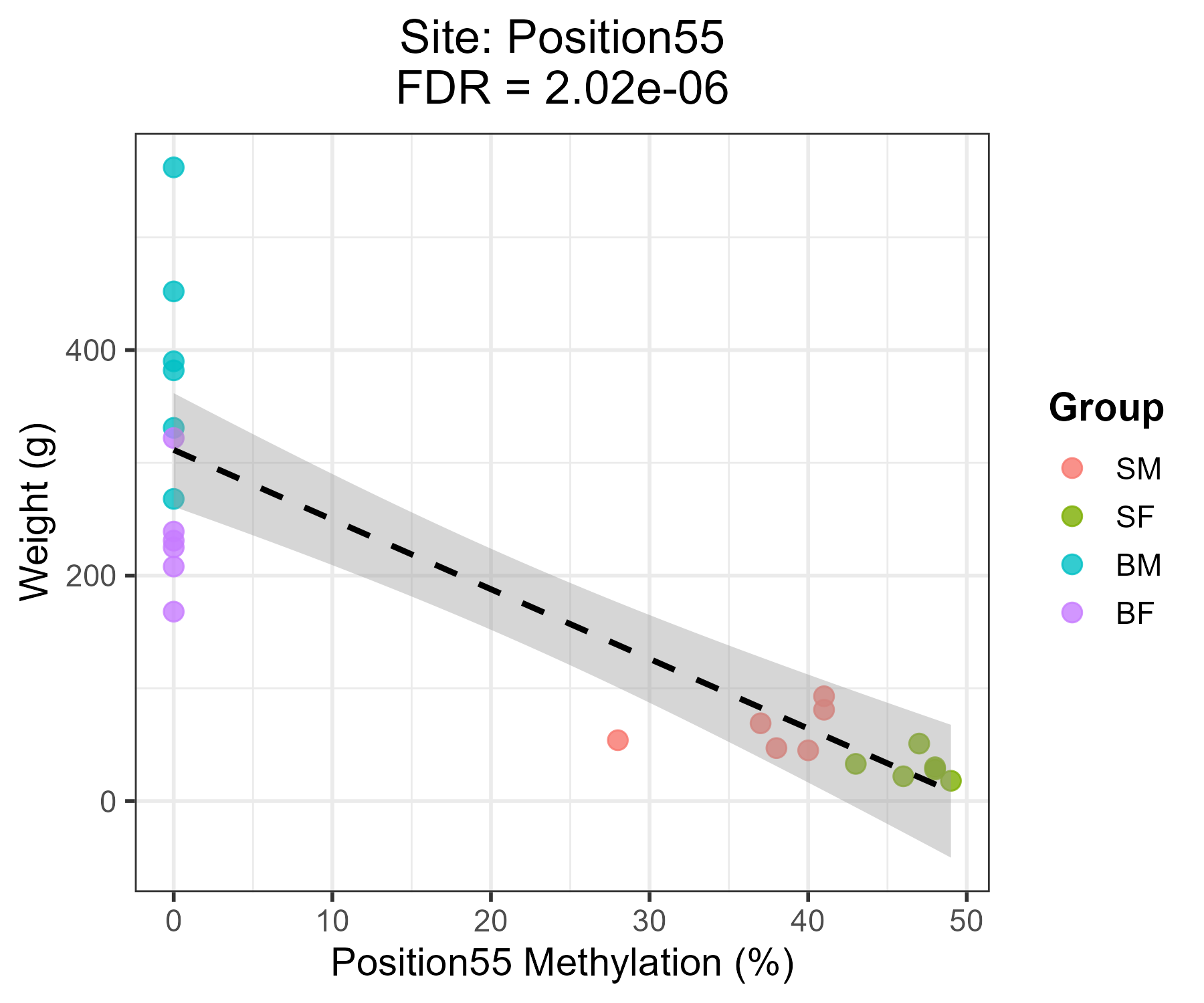

Supplement: Supplementary file 4 [file DataSheet2.zip › Regression_Minus_Strand/Position55_regression.tiff]

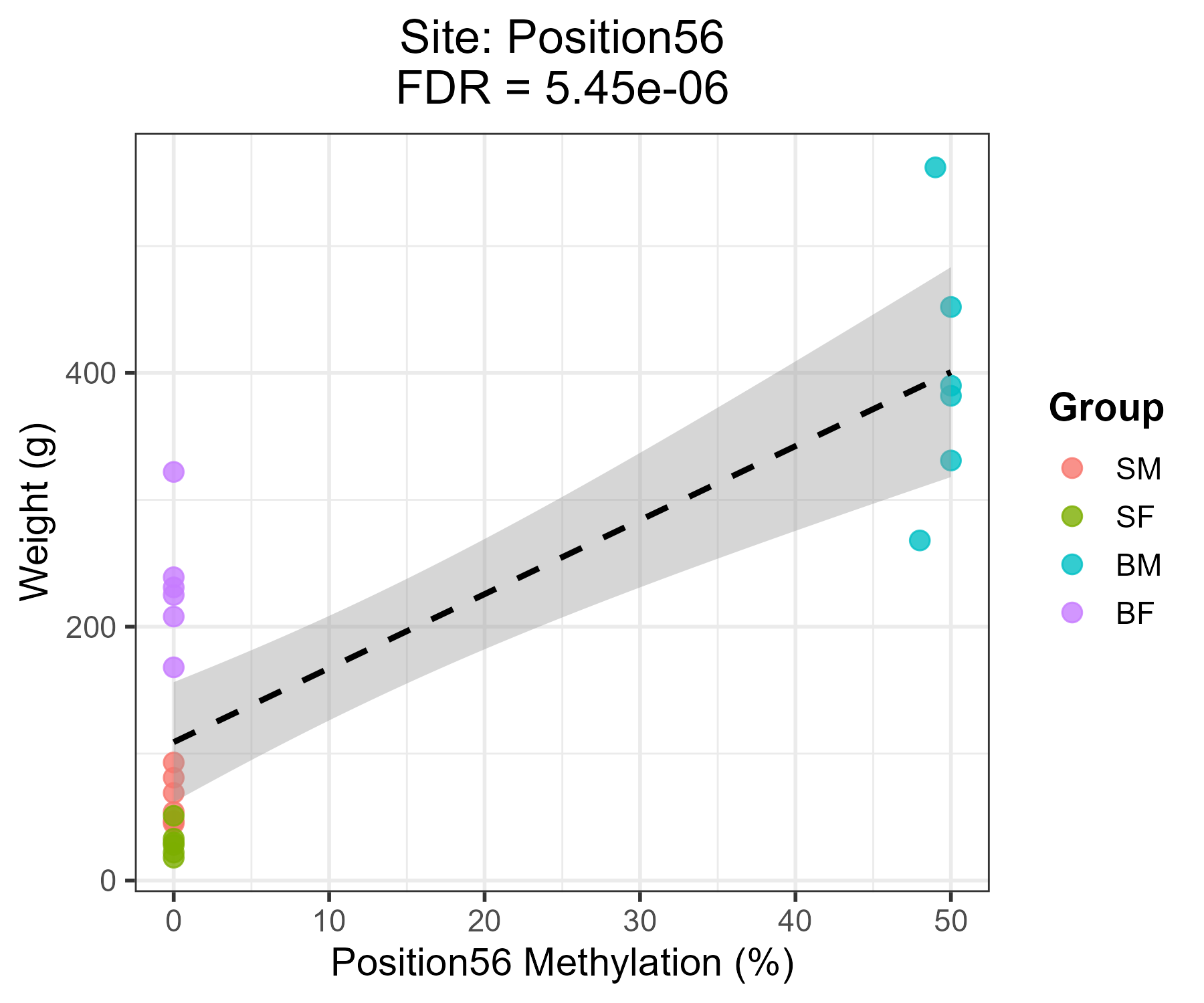

Supplement: Supplementary file 4 [file DataSheet2.zip › Regression_Minus_Strand/Position56_regression.tiff]

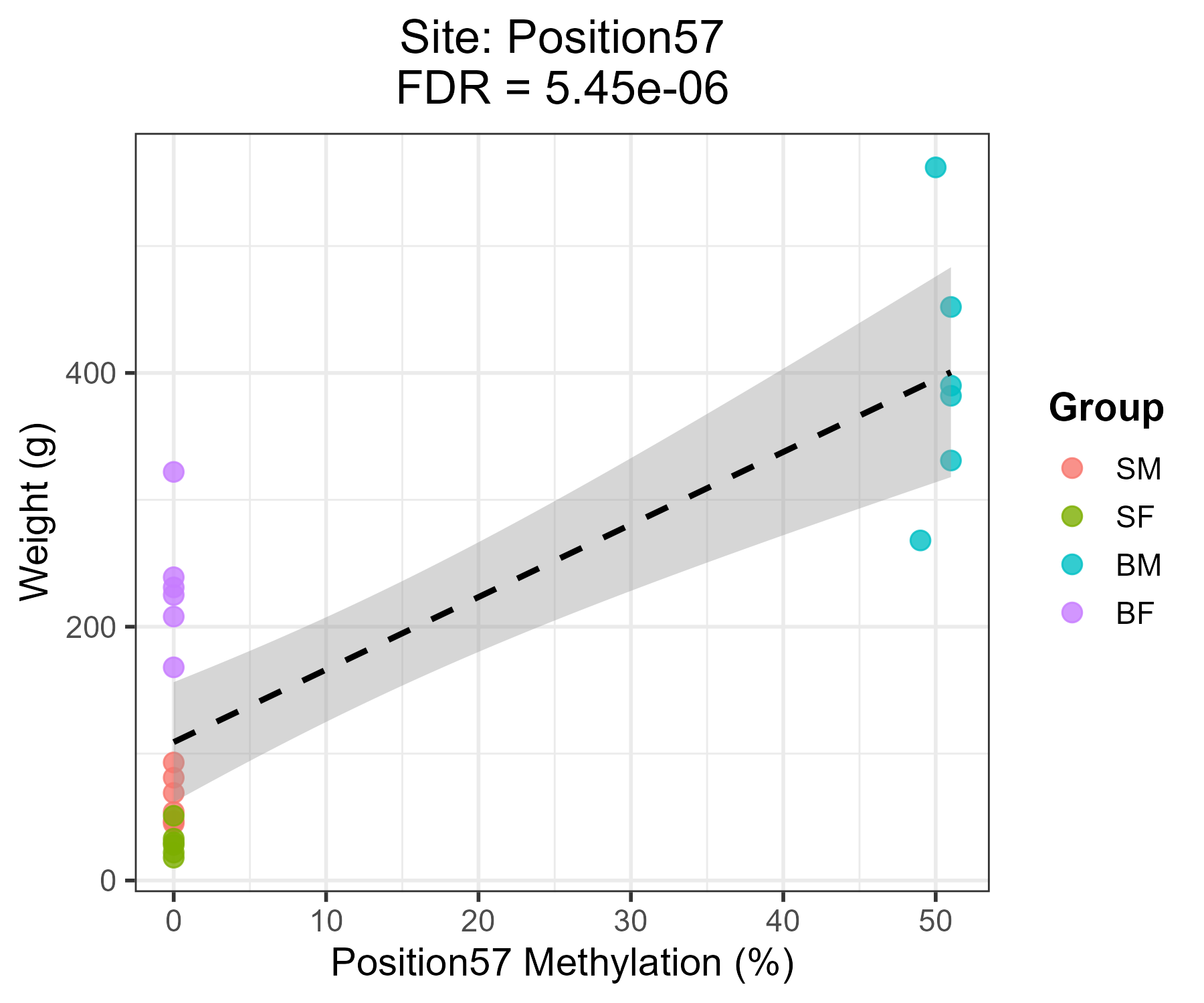

Supplement: Supplementary file 4 [file DataSheet2.zip › Regression_Minus_Strand/Position57_regression.tiff]

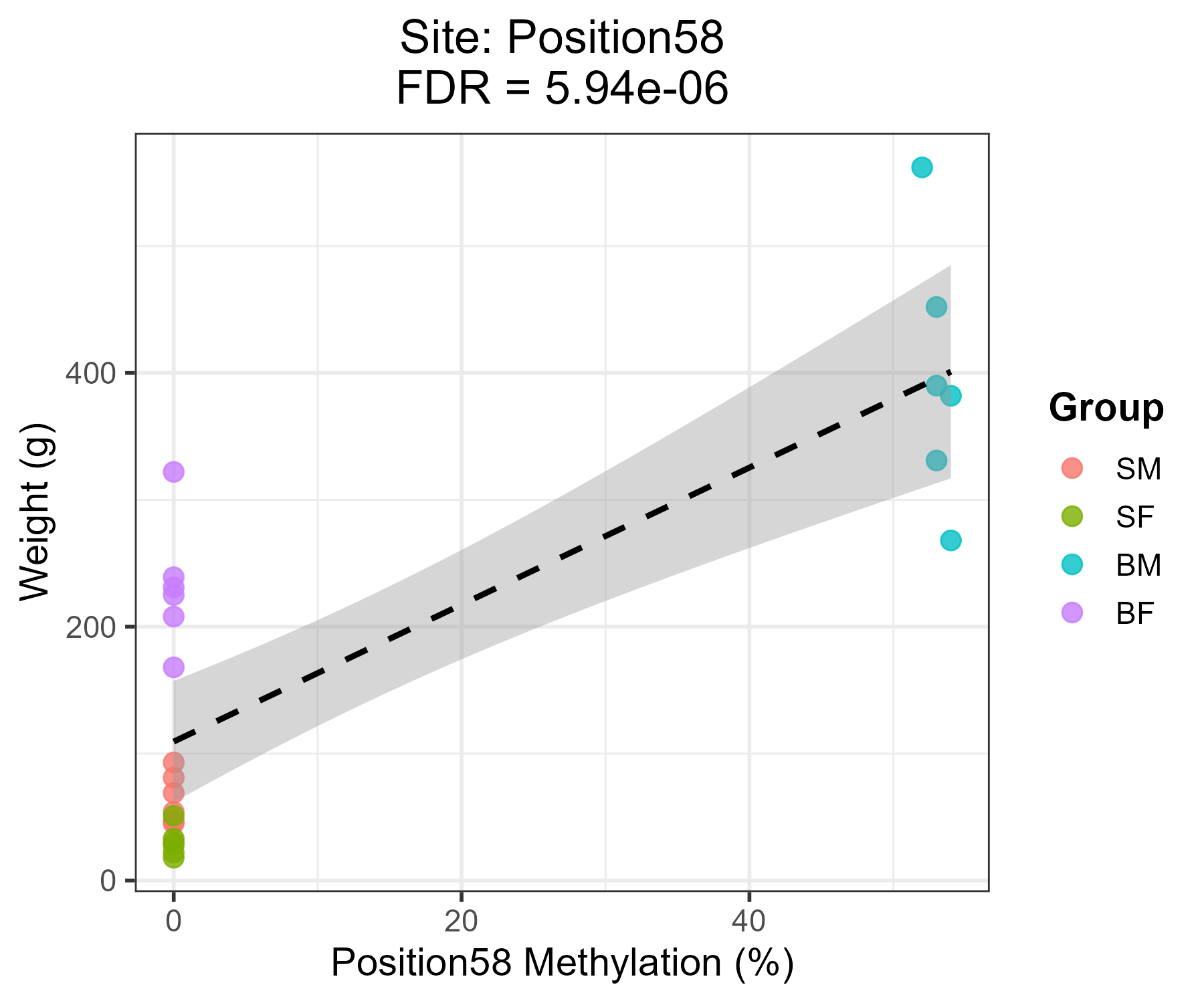

Supplement: Supplementary file 4 [file DataSheet2.zip › Regression_Minus_Strand/Position58_regression.tiff]

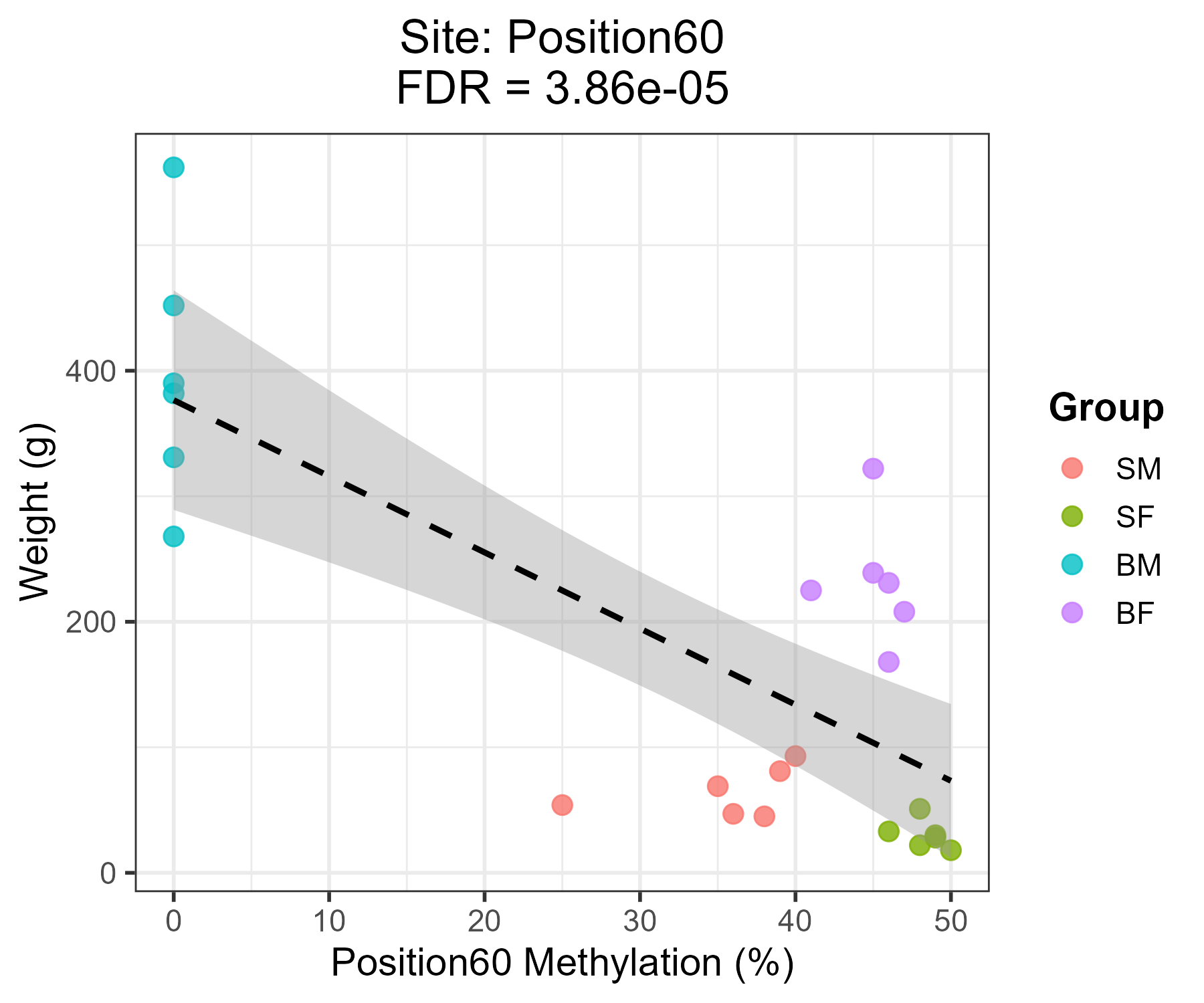

Supplement: Supplementary file 4 [file DataSheet2.zip › Regression_Minus_Strand/Position60_regression.tiff]

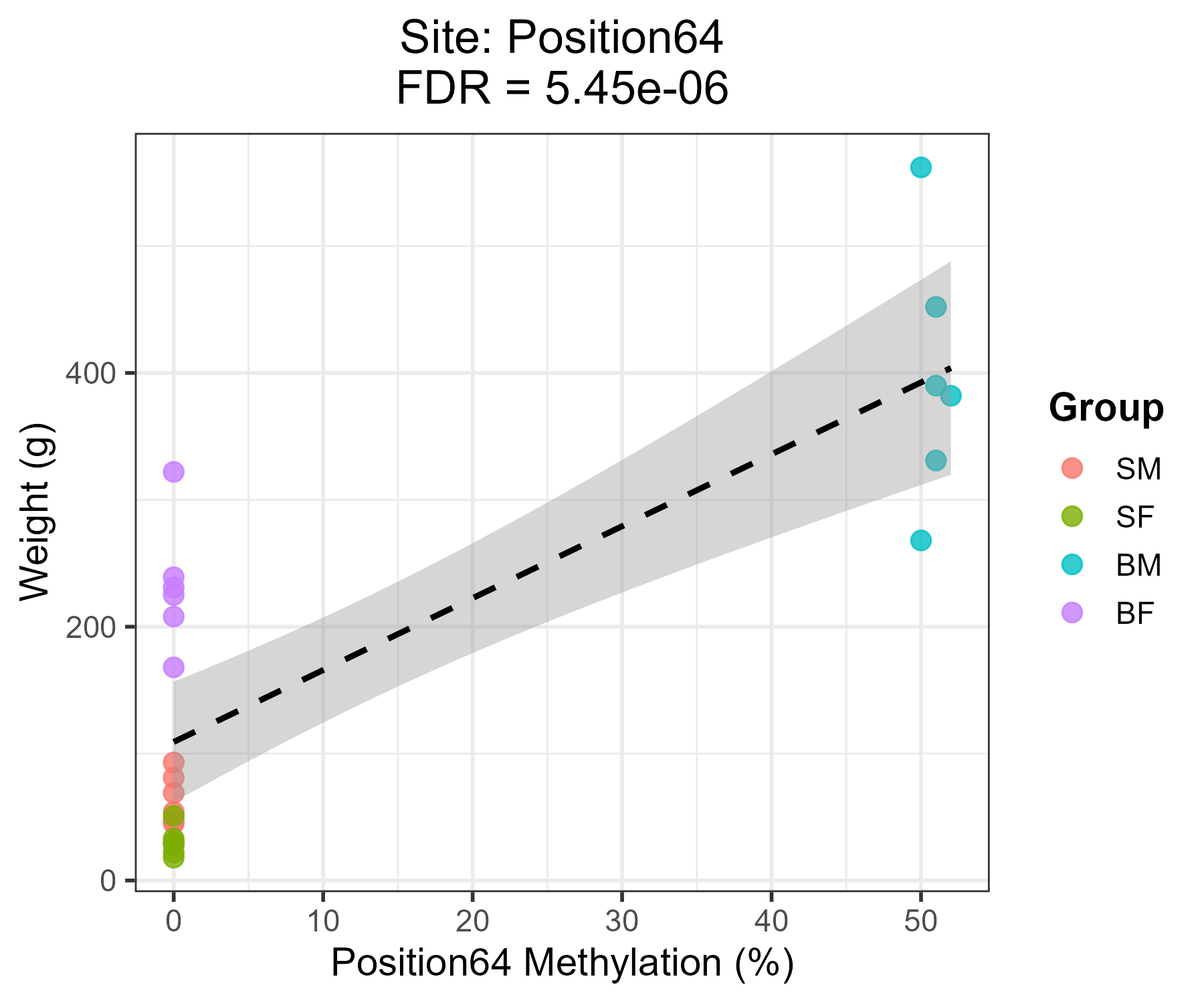

Supplement: Supplementary file 4 [file DataSheet2.zip › Regression_Minus_Strand/Position64_regression.tiff]

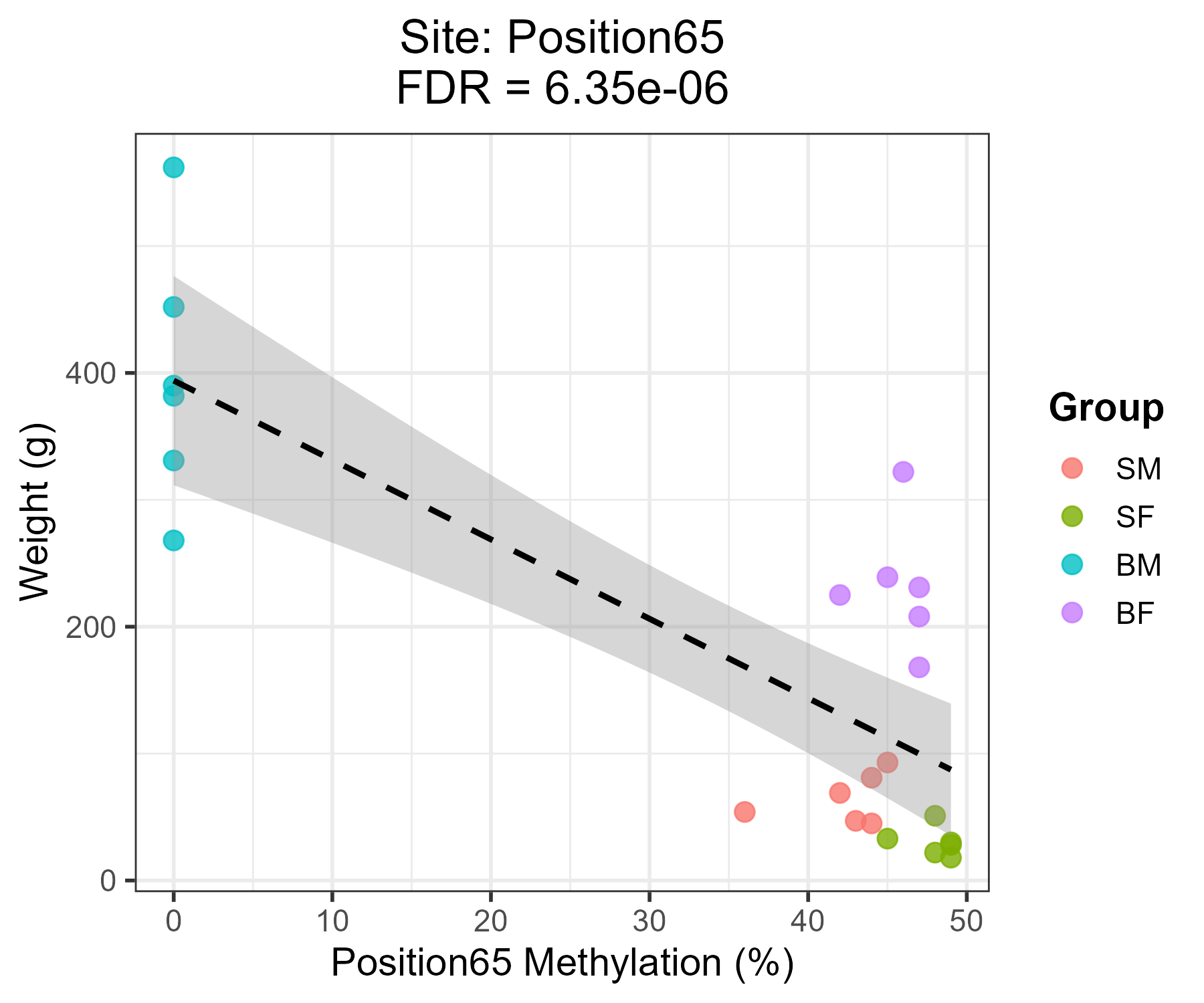

Supplement: Supplementary file 4 [file DataSheet2.zip › Regression_Minus_Strand/Position65_regression.tiff]

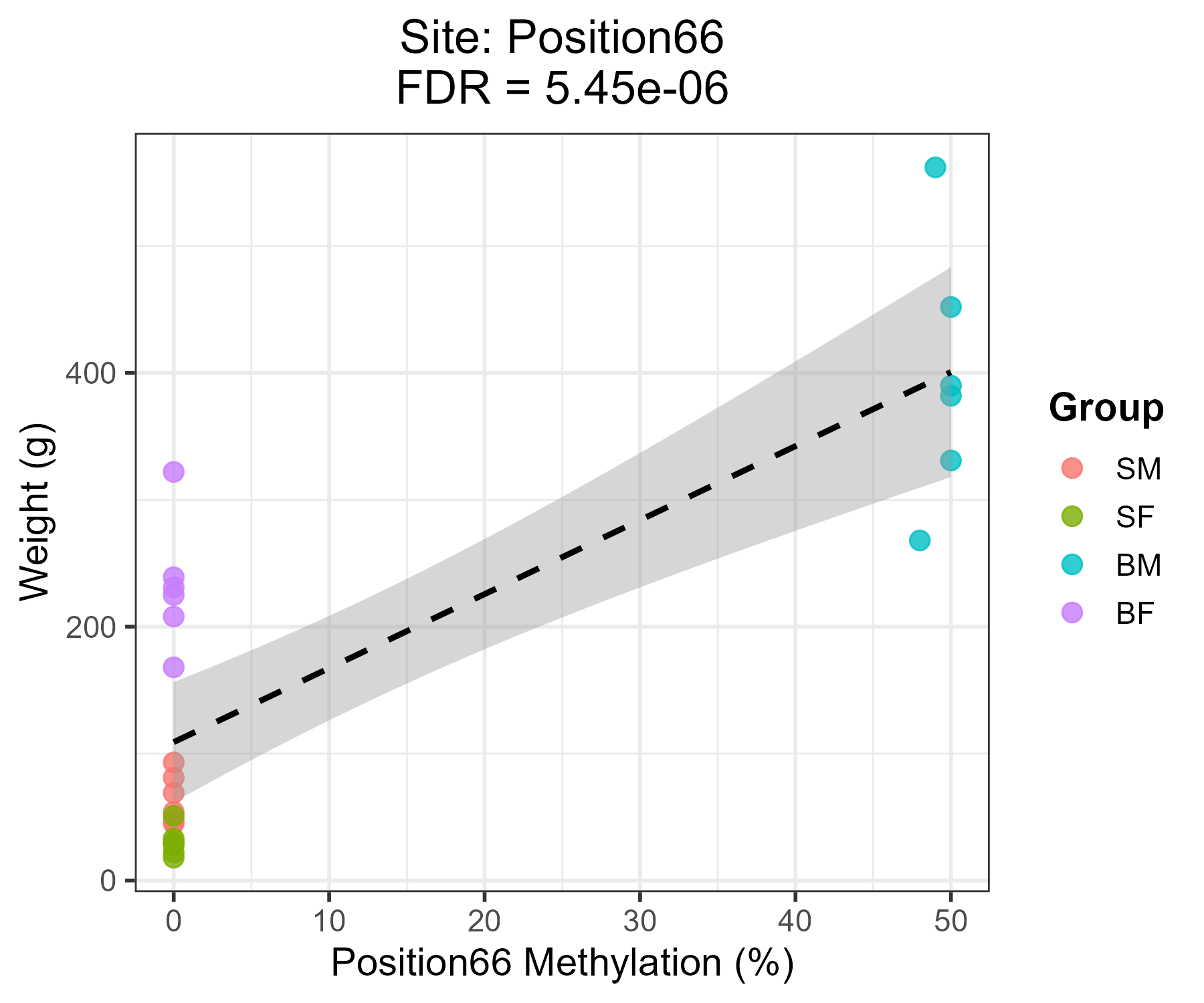

Supplement: Supplementary file 4 [file DataSheet2.zip › Regression_Minus_Strand/Position66_regression.tiff]

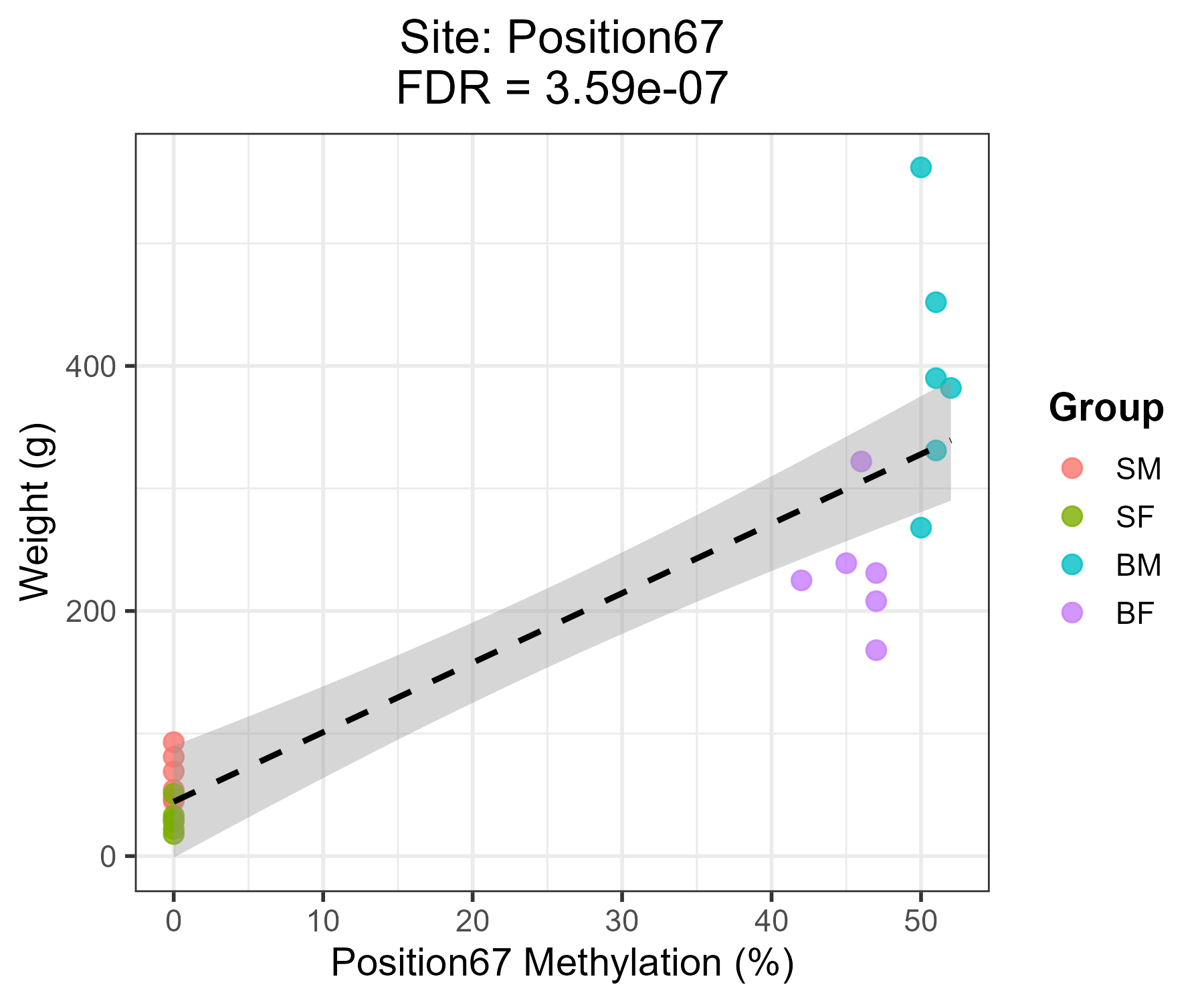

Supplement: Supplementary file 4 [file DataSheet2.zip › Regression_Minus_Strand/Position67_regression.tiff]

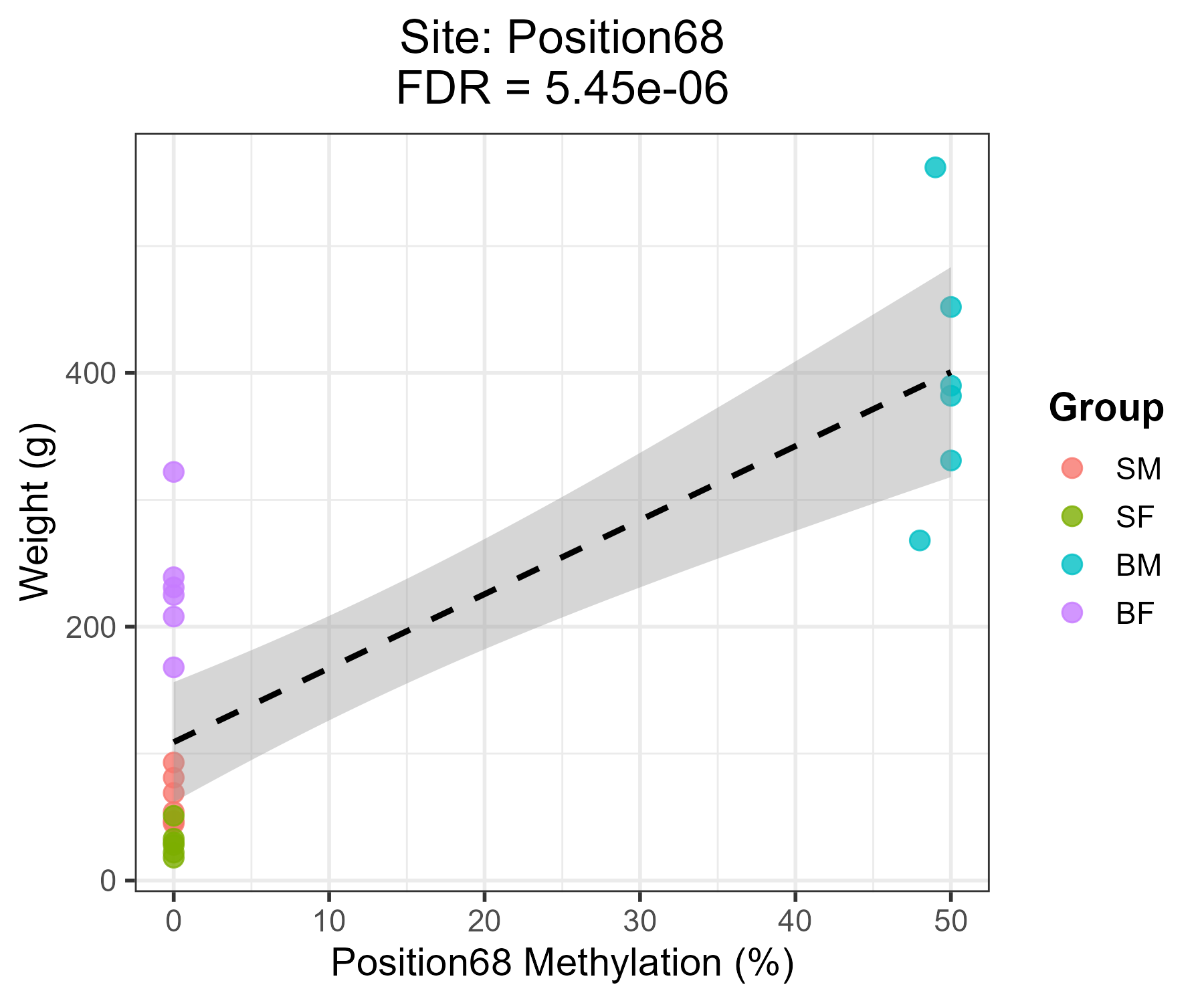

Supplement: Supplementary file 4 [file DataSheet2.zip › Regression_Minus_Strand/Position68_regression.tiff]

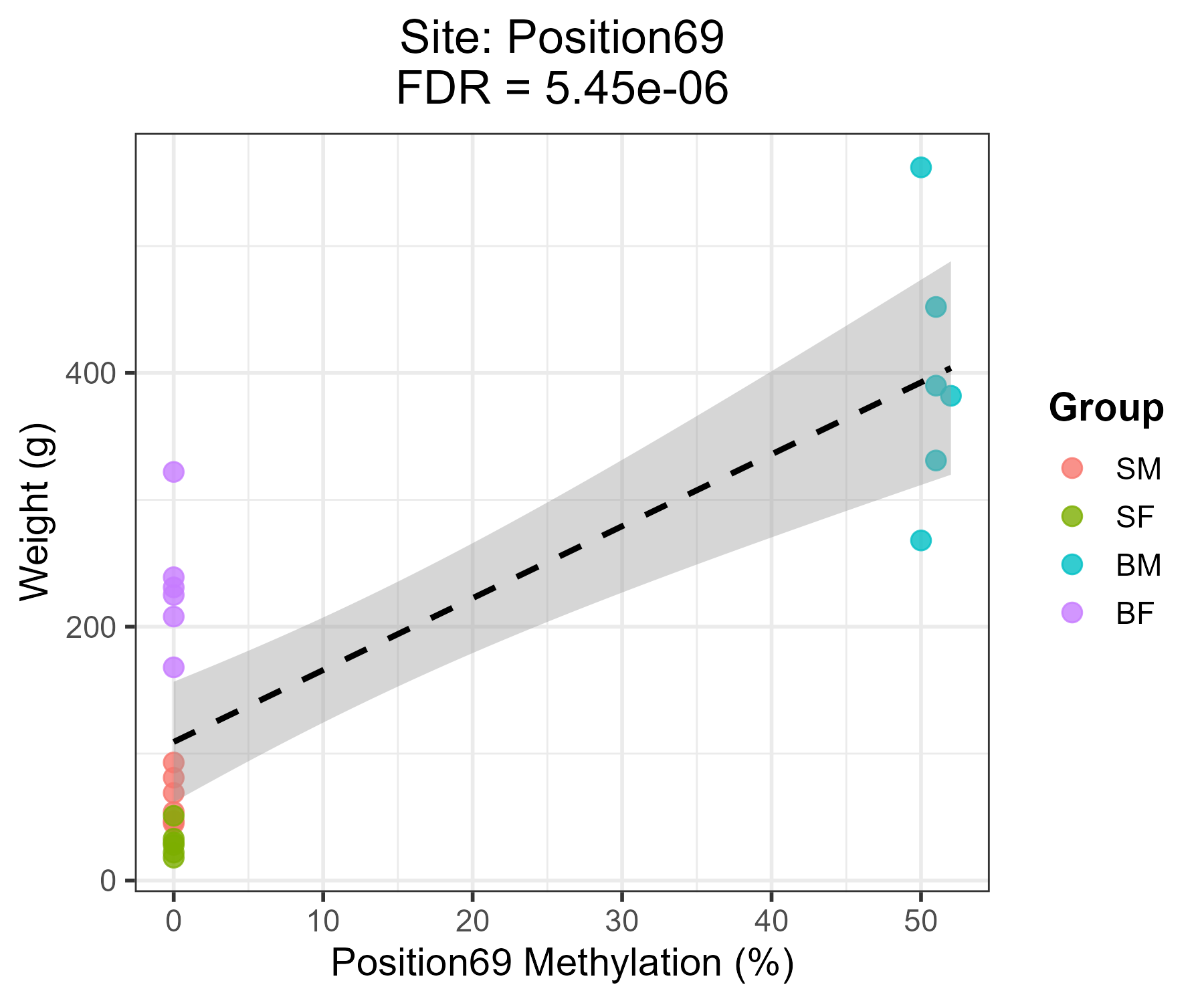

Supplement: Supplementary file 4 [file DataSheet2.zip › Regression_Minus_Strand/Position69_regression.tiff]

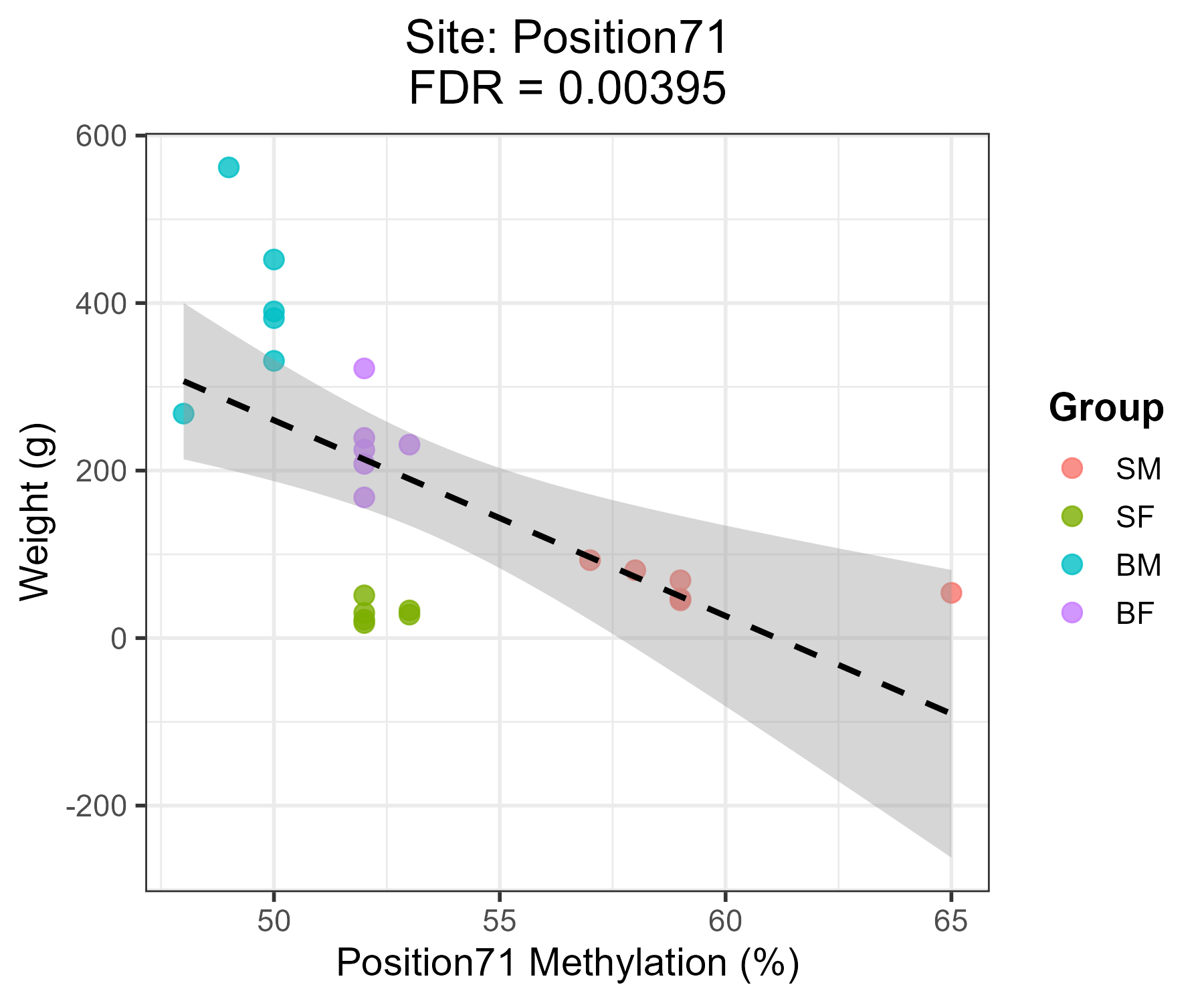

Supplement: Supplementary file 4 [file DataSheet2.zip › Regression_Minus_Strand/Position71_regression.tiff]

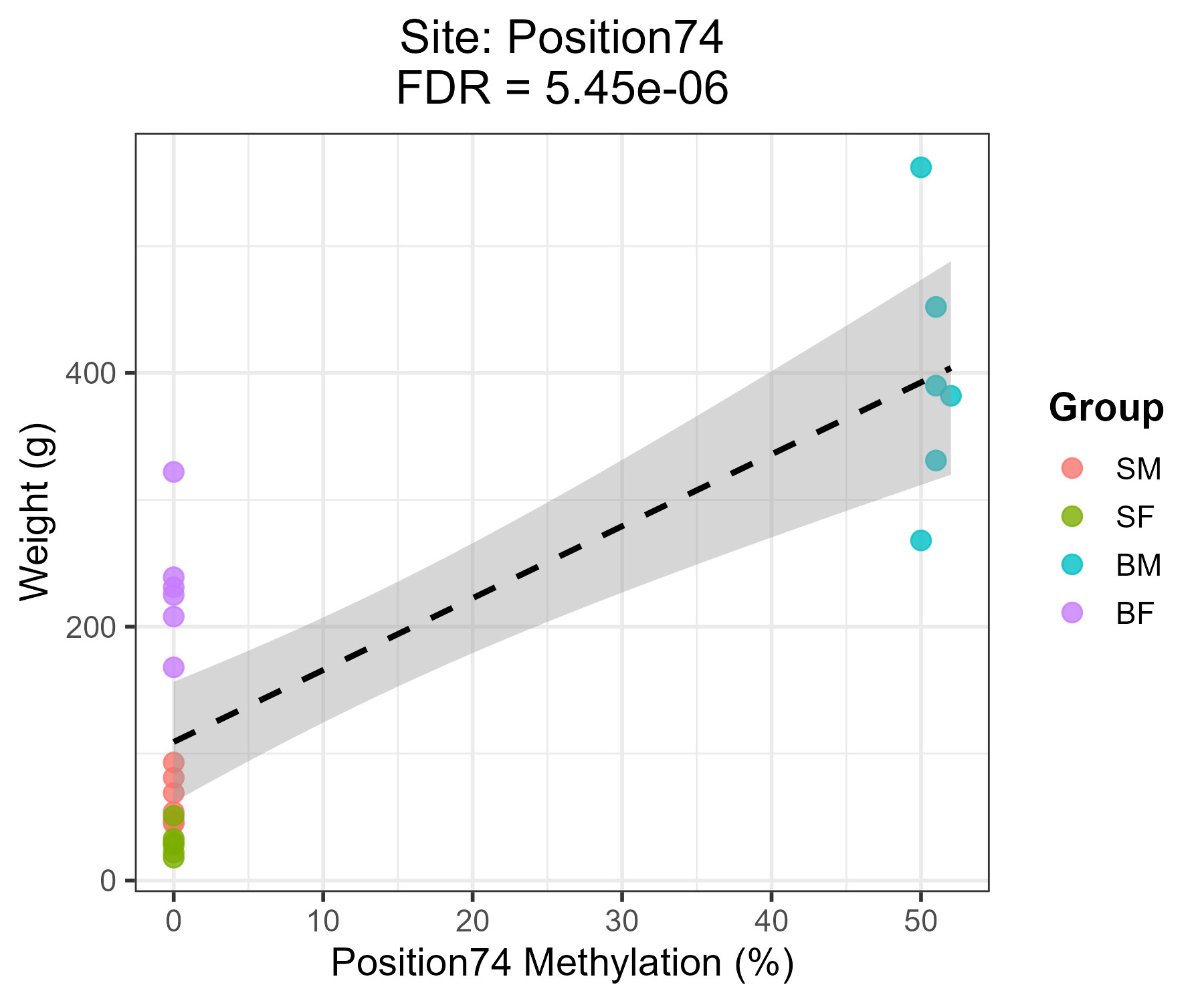

Supplement: Supplementary file 4 [file DataSheet2.zip › Regression_Minus_Strand/Position74_regression.tiff]

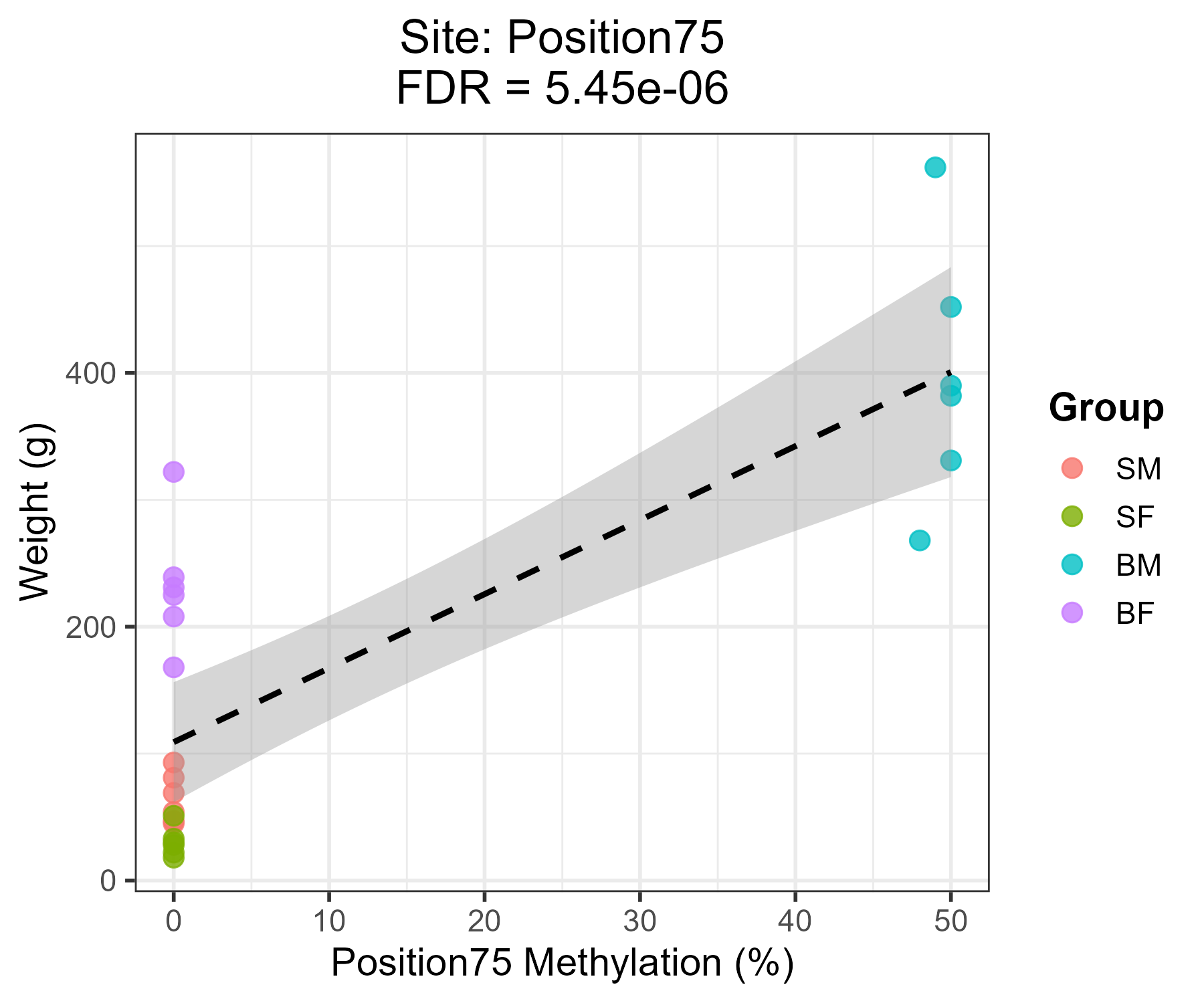

Supplement: Supplementary file 4 [file DataSheet2.zip › Regression_Minus_Strand/Position75_regression.tiff]

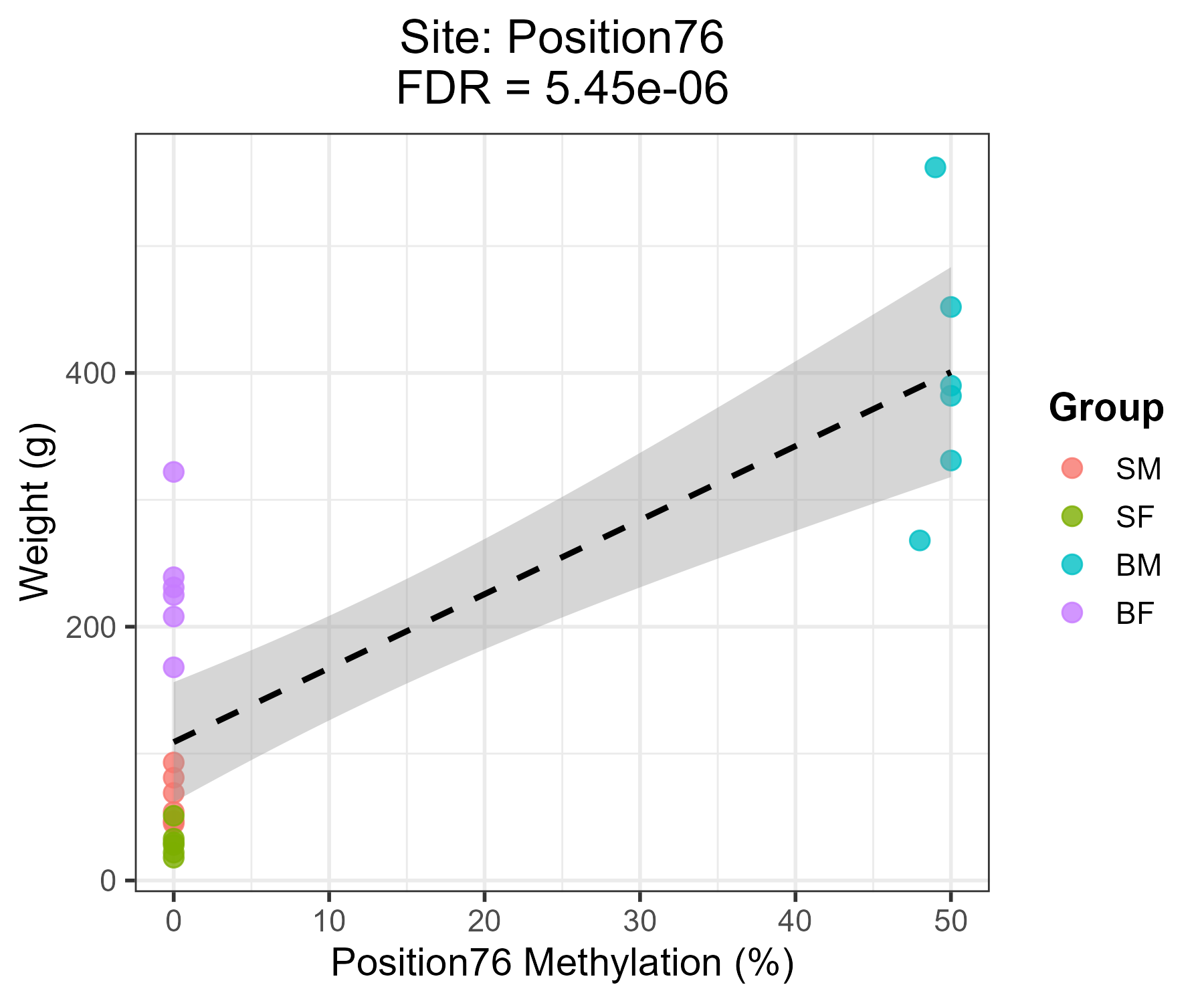

Supplement: Supplementary file 4 [file DataSheet2.zip › Regression_Minus_Strand/Position76_regression.tiff]

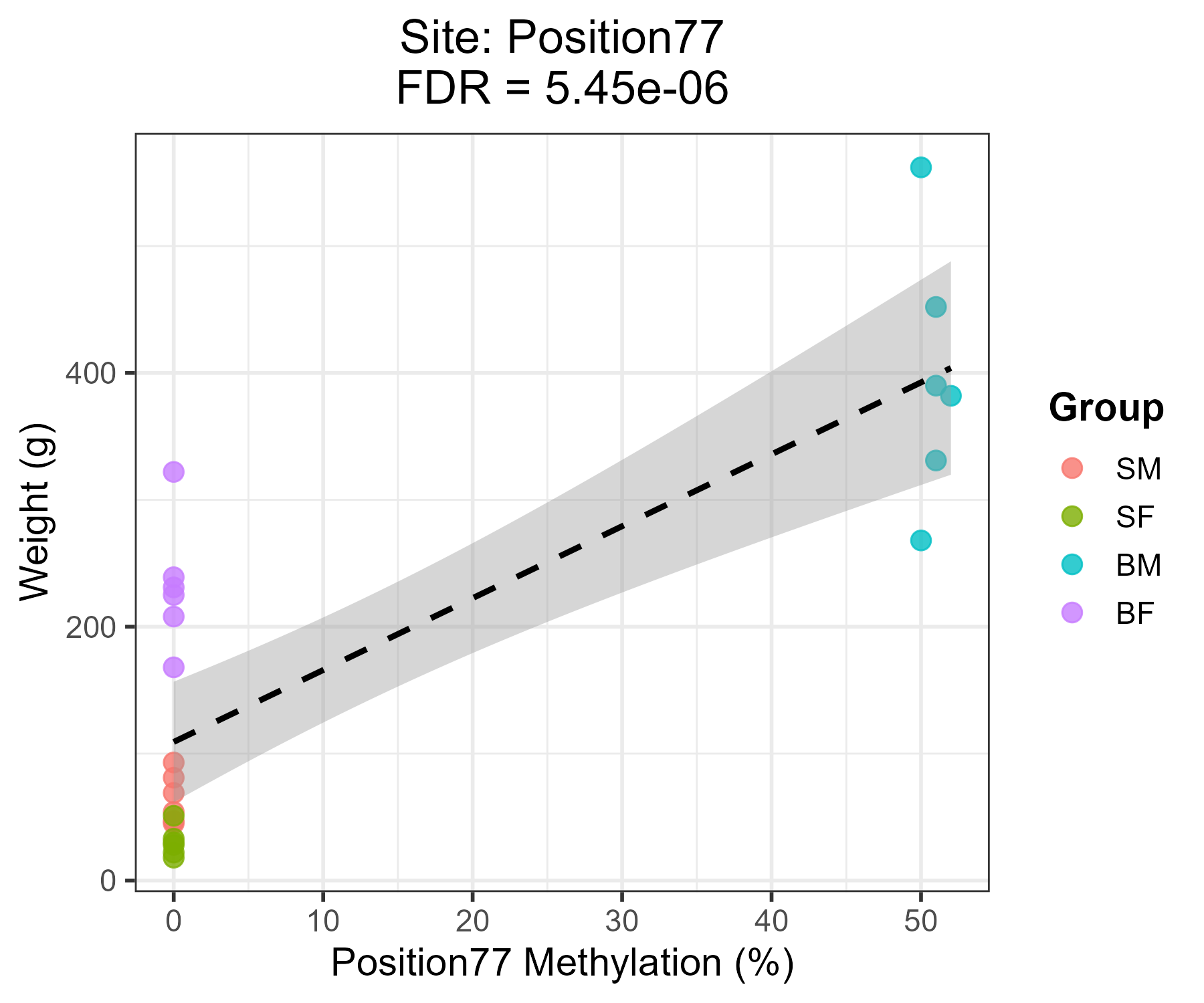

Supplement: Supplementary file 4 [file DataSheet2.zip › Regression_Minus_Strand/Position77_regression.tiff]

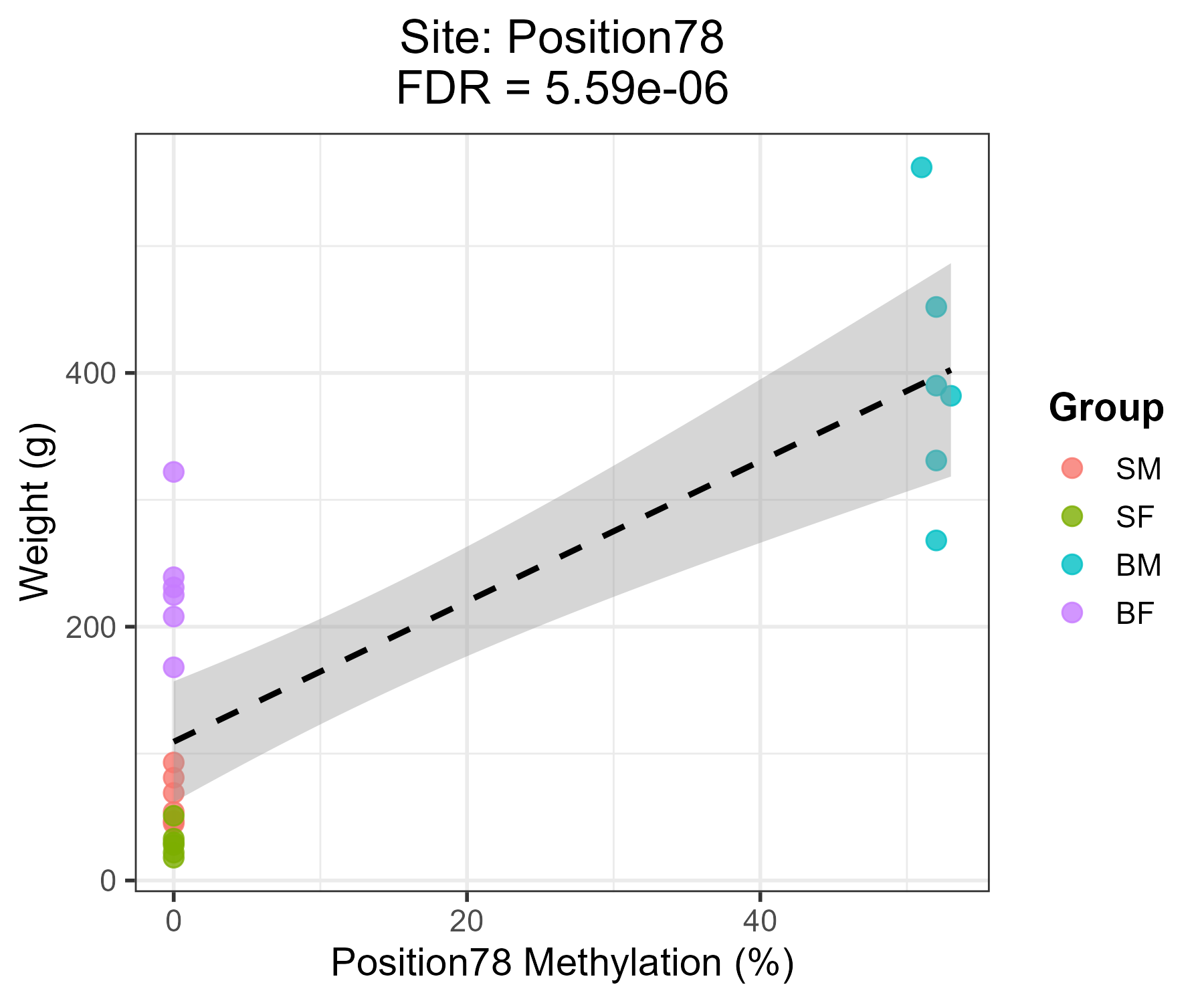

Supplement: Supplementary file 4 [file DataSheet2.zip › Regression_Minus_Strand/Position78_regression.tiff]

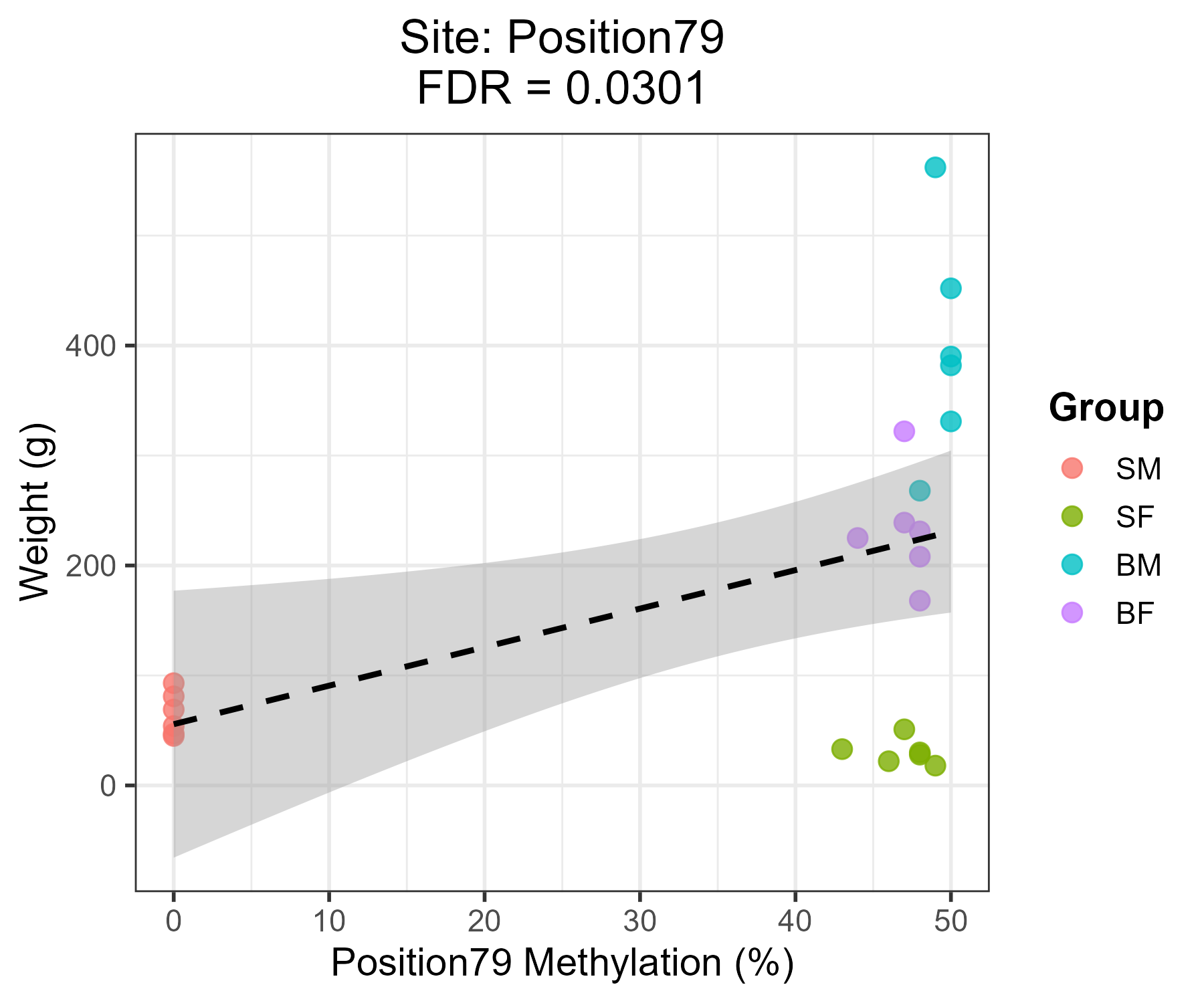

Supplement: Supplementary file 4 [file DataSheet2.zip › Regression_Minus_Strand/Position79_regression.tiff]

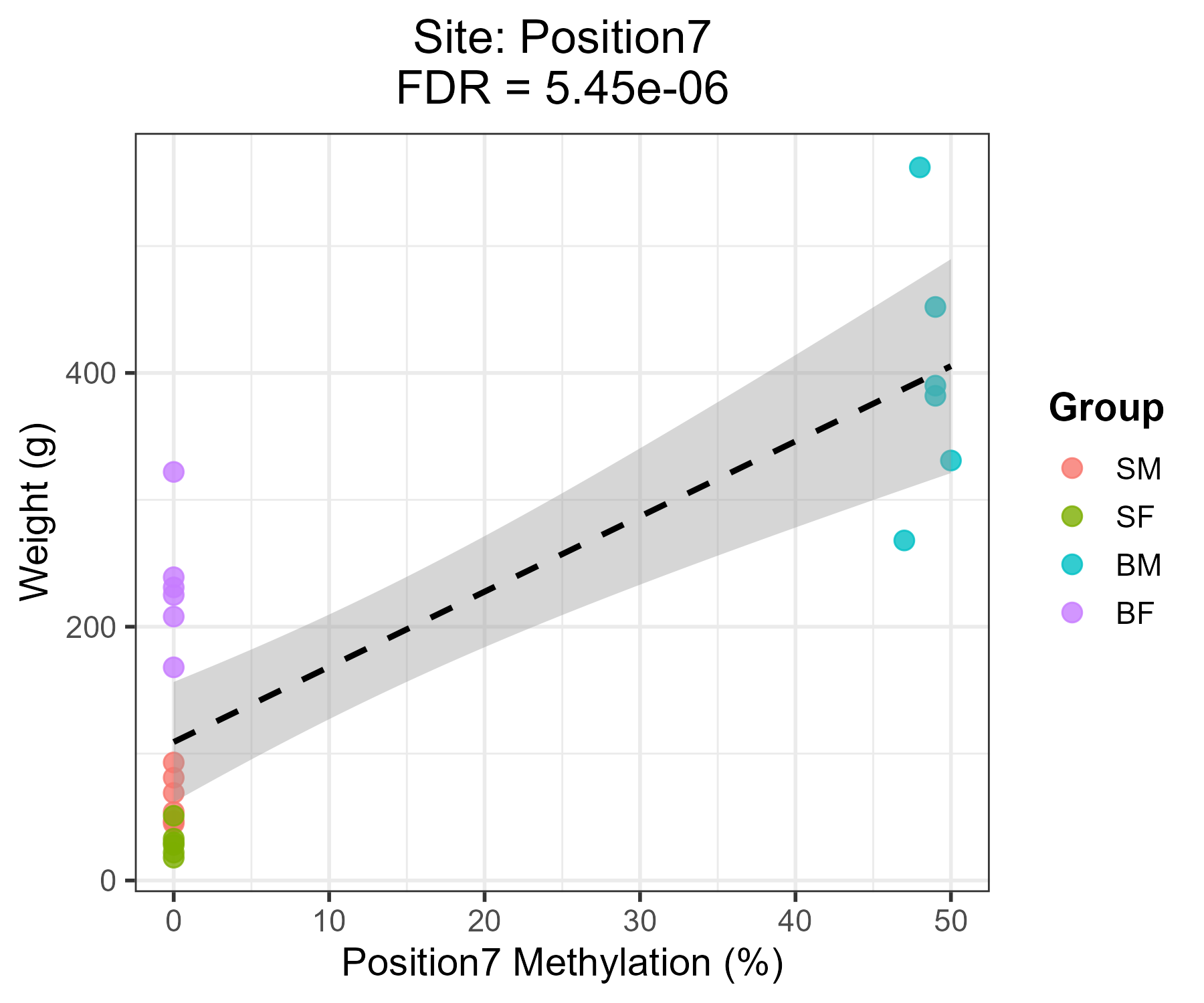

Supplement: Supplementary file 4 [file DataSheet2.zip › Regression_Minus_Strand/Position7_regression.tiff]

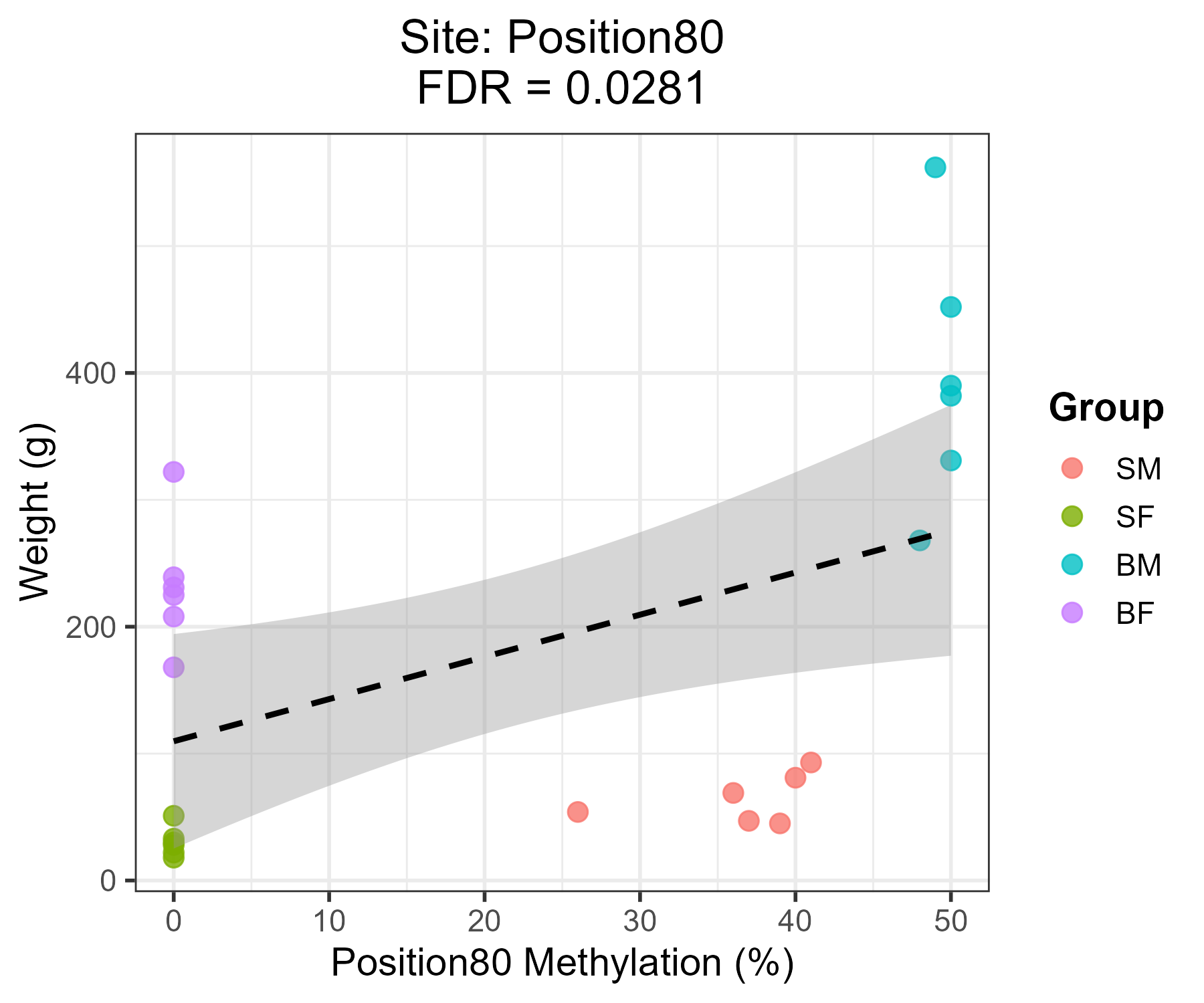

Supplement: Supplementary file 4 [file DataSheet2.zip › Regression_Minus_Strand/Position80_regression.tiff]

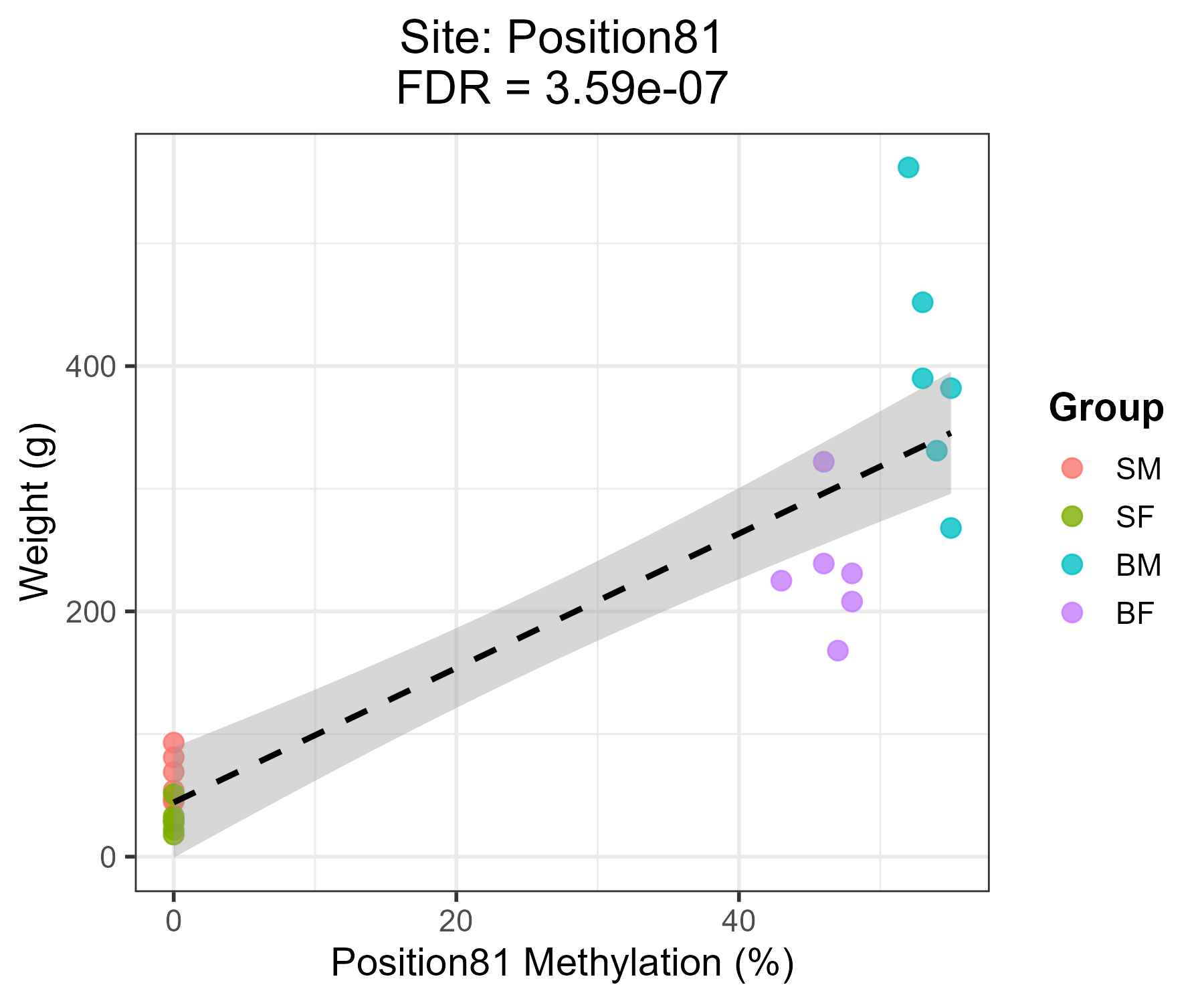

Supplement: Supplementary file 4 [file DataSheet2.zip › Regression_Minus_Strand/Position81_regression.tiff]

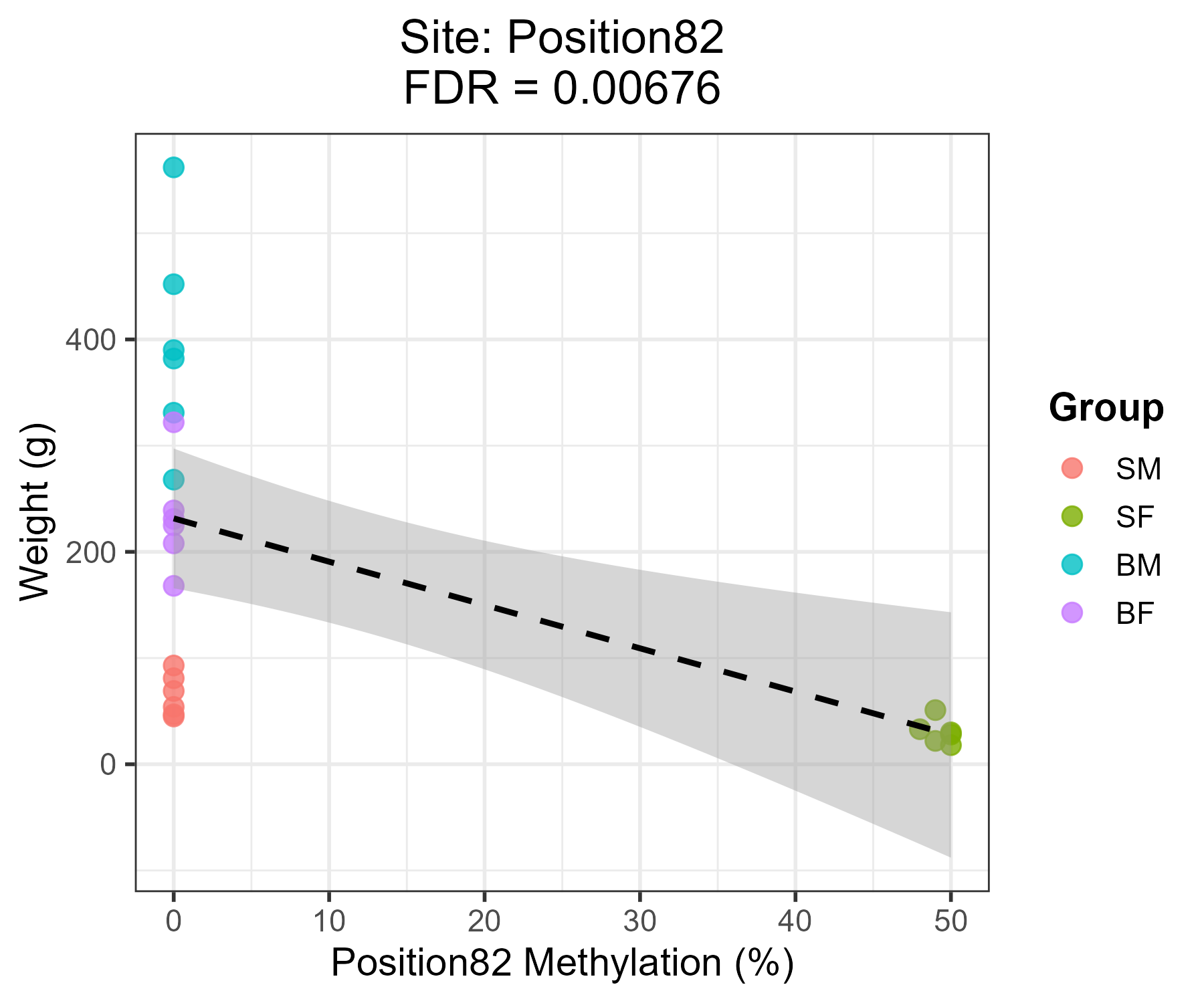

Supplement: Supplementary file 4 [file DataSheet2.zip › Regression_Minus_Strand/Position82_regression.tiff]

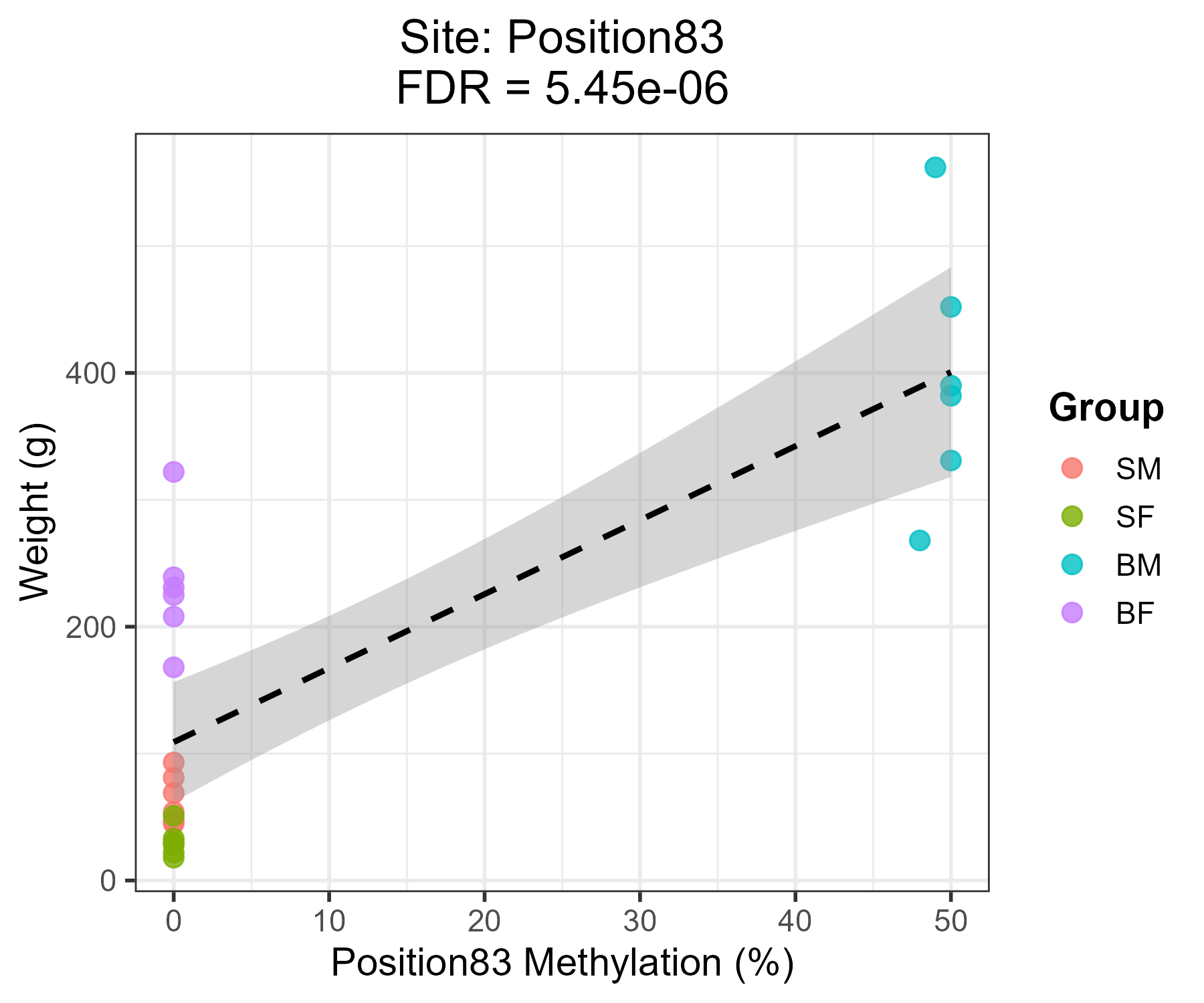

Supplement: Supplementary file 4 [file DataSheet2.zip › Regression_Minus_Strand/Position83_regression.tiff]

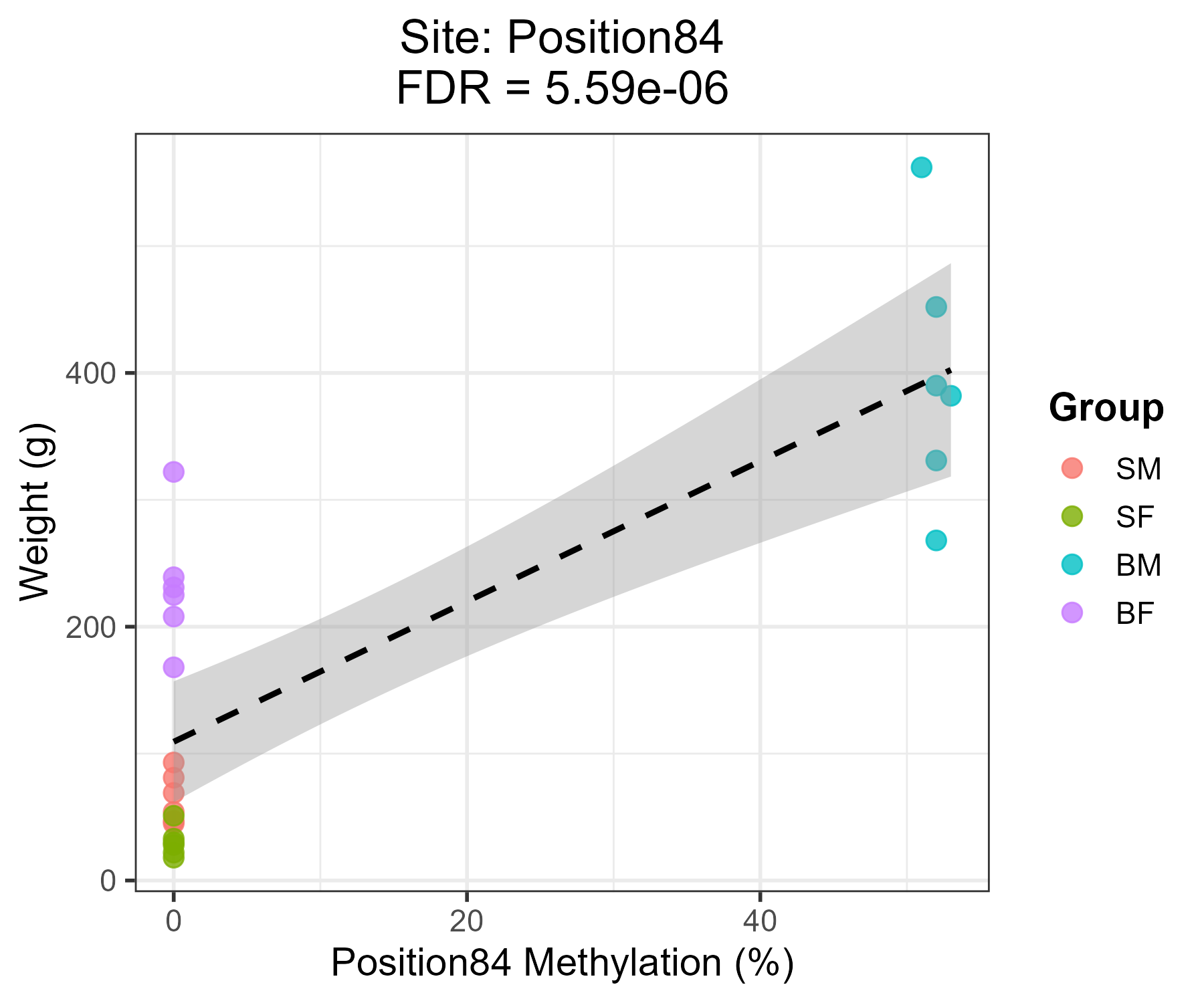

Supplement: Supplementary file 4 [file DataSheet2.zip › Regression_Minus_Strand/Position84_regression.tiff]
